# Analysis of Gene Expression Profiles in the Human Brain Stem, Cerebellum and Cerebral Cortex

**DOI:** 10.1371/journal.pone.0159395

**Published:** 2016-07-19

**Authors:** Lei Chen, Chen Chu, Yu-Hang Zhang, Changming Zhu, Xiangyin Kong, Tao Huang, Yu-Dong Cai

**Affiliations:** 1 School of Life Sciences, Shanghai University, Shanghai, 200444, China; 2 College of Information Engineering, Shanghai Maritime University, Shanghai, 201306, China; 3 Institute of Biochemistry and Cell Biology, Shanghai Institutes for Biological Sciences, Chinese Academy of Sciences, Shanghai, 200031, China; 4 Institute of Health Sciences, Shanghai Institutes for Biological Sciences, Chinese Academy of Sciences, Shanghai, 200031, China; Medical College of Wisconsin, UNITED STATES

## Abstract

The human brain is one of the most mysterious tissues in the body. Our knowledge of the human brain is limited due to the complexity of its structure and the microscopic nature of connections between brain regions and other tissues in the body. In this study, we analyzed the gene expression profiles of three brain regions—the brain stem, cerebellum and cerebral cortex—to identify genes that are differentially expressed among these different brain regions in humans and to obtain a list of robust, region-specific, differentially expressed genes by comparing the expression signatures from different individuals. Feature selection methods, specifically minimum redundancy maximum relevance and incremental feature selection, were employed to analyze the gene expression profiles. Sequential minimal optimization, a machine-learning algorithm, was employed to examine the utility of selected genes. We also performed a literature search, and we discuss the experimental evidence for the important physiological functions of several highly ranked genes, including NR2E1, DAO, and LRRC7, and we give our analyses on a gene (TFAP2B) that have not been investigated or experimentally validated. As a whole, the results of our study will improve our ability to predict and understand genes related to brain regionalization and function.

## Introduction

Human brains are distinguished from those of all other species by their incomparable cognitive capacities. Throughout human history, people have questioned how this mysterious and powerful organ functions in such a highly orchestrated manner. Studies of the human brain using cellular and molecular biological techniques have been undertaken for generations, but the mechanisms underlying the development, differentiation and function of the human brain remain quite elusive. It is widely accepted that fine-tuned spatiotemporal gene expression contributes to the proper function of individual tissues [1]. With the advancement of high-throughput technologies, brain transcriptomics studies have gained more attention and have given rise to a large amount of brain gene expression data over the past decade. Comparative studies of gene expression in the brains of different species have shown that the divergence in brain gene expression patterns has contributed to the changes in human brain function during evolution [2], and some human-specific brain gene expression patterns have been gradually revealed [3–5]. Spatially regulated gene expression is another feature of the human brain that is closely related to its development, differentiation and region-specific functions. Previous studies compared brain regional transcriptomes among different species and revealed a correlation between brain region-specific gene expression and function from an evolutionary perspective [6–8]. However, much less is known about intra-specific brain gene expression in humans and how the divergence in gene expression patterns of human brains contributes to differences based on age, race, sex, and other factors. Financial limitations and the limited availability of human samples usually impede the amount of data acquired or analyzed in a given study, and therefore, it is of great significance to perform data mining of previous datasets [9].

In 2013, the Human Brain Project (HBP), a large, 10-year research project, was established to unravel the mystery of human brains. One of its goals is to organize neuroscience data using a variety of computational models and analytical tools and to better serve the integration and analysis of increasingly large-volume, high-dimensional, and fine-grained experimental data. Despite the progress the HBP has made in the past two years, many obstacles in the methodology (data integration, computation, engineering, *etc*.) and overall research paradigm must still be addressed [10]. In addition to the HBP, several other previous or ongoing research projects also provide information regarding human brains. For example, the Allen Human Brain Atlas serves as an integrative and powerful database of brain gene expression data with high anatomical resolution [11] and has provided multimodal data-mining resources for many previous studies [9, 12, 13].

In this study, we further explore the universalities of regional gene expression of human brains, as well as the differential gene expression patterns among individuals of different ages, races and sexes. Compared to previous studies, we sought to better explore region-specific brain gene expression by minimizing the differences among samples. Considering data availability and reliability, we obtained gene expression data from the Allen Human Brain Atlas database. In this study, we employed several feature-selection methods, including minimum redundancy maximum relevance (mRMR) and incremental feature selection (IFS) [14], and a machine learning method, the sequential minimal optimization (SMO) algorithm, [15, 16] to analyze the gene expression profiles of the brain stem (BS), cerebellum (CB) and cerebral cortex (CC) in the brains of six different people. We also performed a literature search to gather evidence to support our analysis.

## Materials and Methods

### Materials

We downloaded the gene expression profiles of BS, CB and CC samples from six people (H0351.2001, H0351.2002, H0351.1009, H0351.1012, H0351.1015 and H0351.1016) from the Allen Brain Atlas [11] (http://human.brain-map.org/static/download). Detailed information regarding these six individuals is provided in S1 Text, which can be downloaded from http://help.brain-map.org/download/attachments/2818165/CaseQual_and_DonorProfiles.pdf?version=1&modificationDate=1382051848013. The number of samples from each region available for each person can be found in **Table 1**. Depending on the brain region from which the sample was obtained, samples from one person can be divided into three classes, BS, CB and CC, and comprise a dataset. For each sample, the expression levels of 20,782 genes were measured using microarray analysis. And the expression level of each gene was the feature used for classifying the samples from different brain regions.

**Table 1:** The distribution of samples, obtained from six individuals, among three regions of the human brain.

**Individual identification code**	**Number of samples from each region of the human brain**			**Total number of samples**
	**Brain stem (BS)**	**Cerebellum (CB)**	**Cerebral cortex (CC)**	
H0351.1009	26	42	295	363
H0351.1012	80	48	401	529
H0351.1015	79	62	329	470
H0351.1016	59	80	362	501
H0351.2001	154	53	739	946
H0351.2002	188	83	622	893

The goal of this study was to identify the differentially expressed genes among different brain regions in each person and then obtain a list of robust, region-specific, differentially expressed genes by comparing the expression signatures from different persons. Each of the six datasets was used as the training dataset for one analysis in which the remaining five datasets were used as test datasets.

For each individual, there were samples from three brain regions including BS, CB and CC. And we try to identify the genes that can classify them.

For cross-individual analysis, we used the discriminative genes identified from one individual to classify the samples in other five individuals. The cross-individual analysis could measure the robustness of the discriminative genes and obtain a brain region specific, but not individual specific discriminative gene list. The individual differences could be a confounding factor in brain region specific analysis.

### mRMR method

To identify differentially expressed genes, we used a popular feature selection method, mRMR, to analyze the gene expression profiles of three regions in the human brain. The mRMR method, proposed by Peng *et al*. [14], was developed based on two criteria: Max-Relevance and Min-Redundancy. The output of the mRMR program contains two feature lists, the MaxRel feature list and the mRMR feature list, in which all features are sorted. Max-Relevance guarantees that the features that correlate strongly with the target variable receive high ranks, while Min-Redundancy guarantees that a feature with low redundancy to features already in the list is selected in the next round. For the two obtained feature lists, the MaxRel feature list was produced based only on the Max-Relevance criterion, and the mRMR feature list was produced based on both criteria. We define these two feature lists as follows:

$$\left\{ \begin{array}{l} \text{MaxRel features list}\;:\;F_{\text{MaxRel}}=[f_{1}^{M},f_{2}^{M},\cdots ,f_{N}^{M}]\\ \text{mRMR features list}\;:\;F_{\text{mRMR}}=[f_{1}^{m},f_{2}^{m},\cdots ,f_{N}^{m}] \end{array}\right.$$ (1)

The MaxRel feature list can provide clues to assess which features are important for distinguishing samples from different classes. However, the combination of a number of top features in this list is not always an optimal combination for classification because redundancies may occur between them. On the other hand, the mRMR feature list considers these factors. Thus, the mRMR feature list is more appropriate for building an optimal classification model and extracting an optimal combination of features. MaxRel and mRMR feature lists have been widely used to address a variety of biological problems [17–26].

### Prediction engine

The mRMR method described above only provides lists of features. To extract important features (genes), a prediction engine is necessary, and it, together with the mRMR feature list, was used according to the method described below. In this study, we adopted a powerful machine learning method, SMO, as the prediction engine. SMO is proposed by John Platt [15, 16] and is one of the most popular methods for solving support vector machines (SVMs) in the dual space. It is a type of decomposition method and always uses the smallest possible working set, which contains two dual variables and can be updated very effectively. The optimization problem is divided into a number of smallest possible sub-problems, which are solved analytically [27]. Nowadays, it has been used in many algorithms, especially for C-SVM (SVM for classification) [28–30]. Some published papers have validated the convergence behavior of SMO [31–33]. All of these indicate that SMO is a good method to optimize solving procedures of SVM. Because this study involved three classes, pair-wise coupling [34] was applied to build the multi-class classifier.

In Weka [35], a classifier called SMO implements the SMO method described above. For convenience, this classifier was directly employed as the prediction engine and was used with its default parameters.

### Revised IFS method

The original IFS method, which was proposed by Peng *et al*. [14], used the mRMR feature list and a basic prediction engine to extract important features and develop an optimal prediction. All of these procedures are executed only on the training dataset. In this study, we mainly focused on finding differentially expressed genes identified from one individual to classify the samples in other five individuals, but not individual specific differentially expressed genes. We modified the original IFS method by executing it on both the training set and five test datasets. A detailed description is presented below:

1. According to the mRMR feature list $F_{mRMR}=[f_{1}^{m},f_{2}^{m},\ldots ,f_{N}^{m}]$, *N* feature sets, denoted as *F*_1_,*F*_2_,…,*F*_*N*_, can be constructed as $F_{i}=\{f_{1}^{m},f_{2}^{m},\ldots ,f_{i}^{m}\}(i=1,2,\ldots ,N)$, *i*.*e*., *F*_*i*_ contains the first *i* features in the mRMR feature list.
2. For each *F*_*i*_, samples in the training dataset and five test datasets were all represented by features in *F*_*i*_. Then, the classifier SMO was trained on the training set, and its performance was evaluated using five test datasets. The predicted results for each of the test datasets were counted as the total prediction accuracy and accuracy of each class.
3. For each test dataset, an IFS curve was plotted by setting the total prediction accuracy as the Y-axis and the number of features used as the X-axis.

Theoretically, for a training dataset and a test dataset, we want to find the optimal combination of genes that can accurately evaluate the differences between two people. Thus, a feature set that results in the maximum total prediction accuracy on the test dataset represents the optimal combination of genes for which we are searching. However, this feature set always contains an extremely large number of features (genes), which complicates the analysis. In this study, an inflection point was identified on each IFS curve. The inflection point is defined as the first point on the curve for which the total prediction accuracy at this point is greater than or equal to the total prediction accuracy at the point prior to this point and for which the total prediction accuracy is greater than the total prediction accuracy at the point posterior to this point. Features in the feature set corresponding to this point were deemed to be significant for the problem addressed in this study.

## Results and Discussion

### Results of the mRMR and revised IFS methods

The gene expression profiles of three brain regions from six individuals were examined in this study, thereby comprising six datasets. The mRMR method was executed on each of these six datasets and yielded the MaxRel feature list and mRMR feature list. Due to the limitations of our computational power, we only required output of the first 500 features in each of the two lists. The obtained lists are provided in S1 Table.

**Fig 1:**
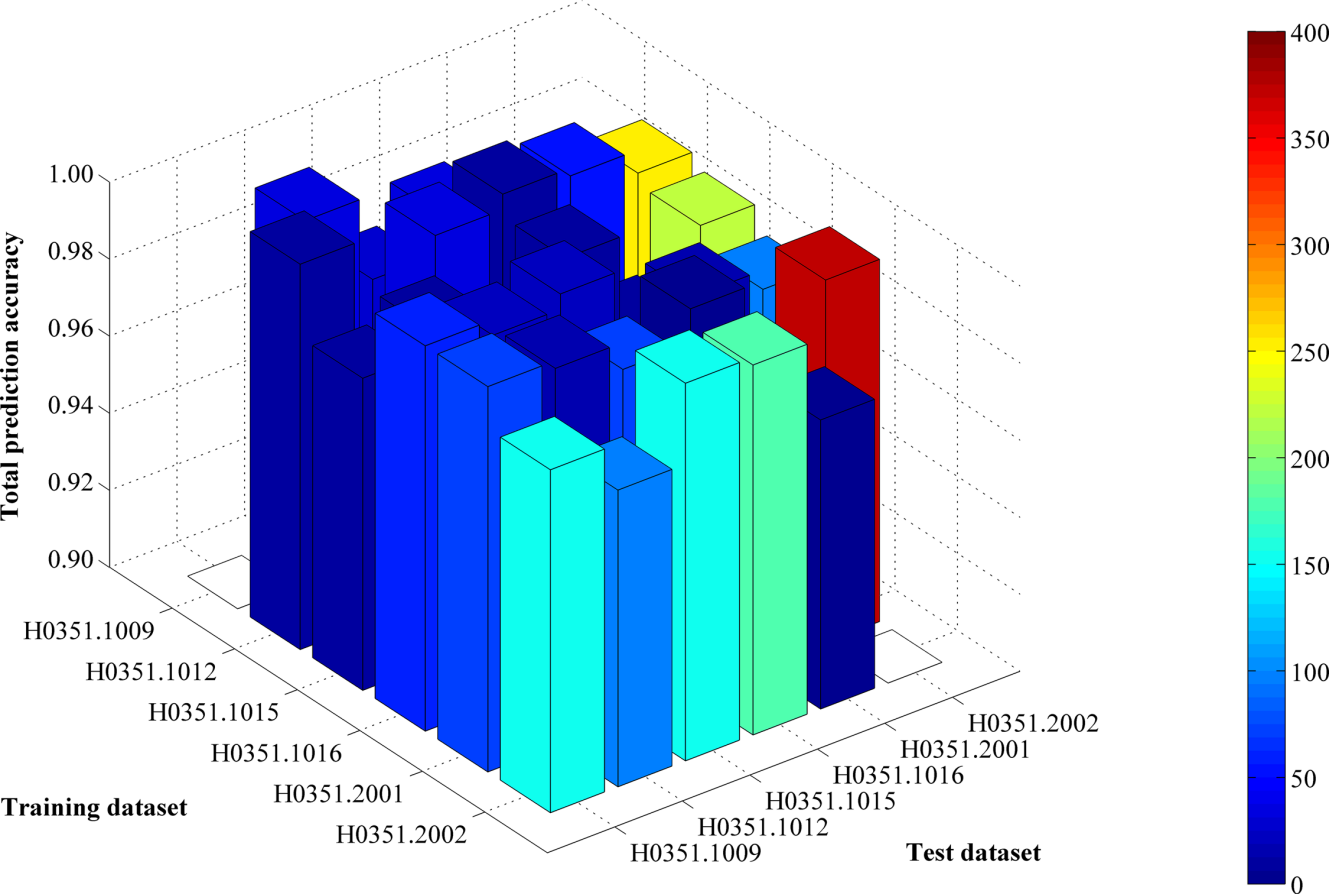
A 3-D histogram illustrating the number of features in the feature set yielding the maximum total prediction accuracy and the corresponding total prediction accuracy. The height of the bar represents the maximum total prediction accuracy, whereas the color of the bar represents the number of features in the feature set yielding the maximum total prediction accuracy.

Each of the six datasets was used as a training dataset, with the other five datasets used as test datasets. Thus, the revised IFS method described in Section “Revised IFS method” was executed six times. Each time, the IFS method constructed 500 feature sets according to the mRMR feature list. The features in each set were used to represent samples in the training dataset and five test datasets. The prediction engine SMO was trained on the training dataset, and its performance was evaluated on the five test datasets. For each test dataset, a series of total prediction accuracies and accuracies of three classes were obtained, all of which are provided in S2 Table. According to the third step of the revised IFS method, the predicted results of each test dataset can produce an IFS curve. Thus, for one training dataset and five test datasets, we can obtain five IFS curves. Six groups of IFS curves are illustrated in S1–S6 Figs. These curves show that for a given training dataset and test dataset, the feature set yielding the maximum value for total prediction accuracy on the test dataset can be obtained, and the numbers of features in these sets are listed in S3 Table. A 3-D histogram was plotted in **Fig 1** to show the number of features in the feature set yielding the maximum total prediction accuracy and the corresponding total prediction accuracy.

**Fig 2:**
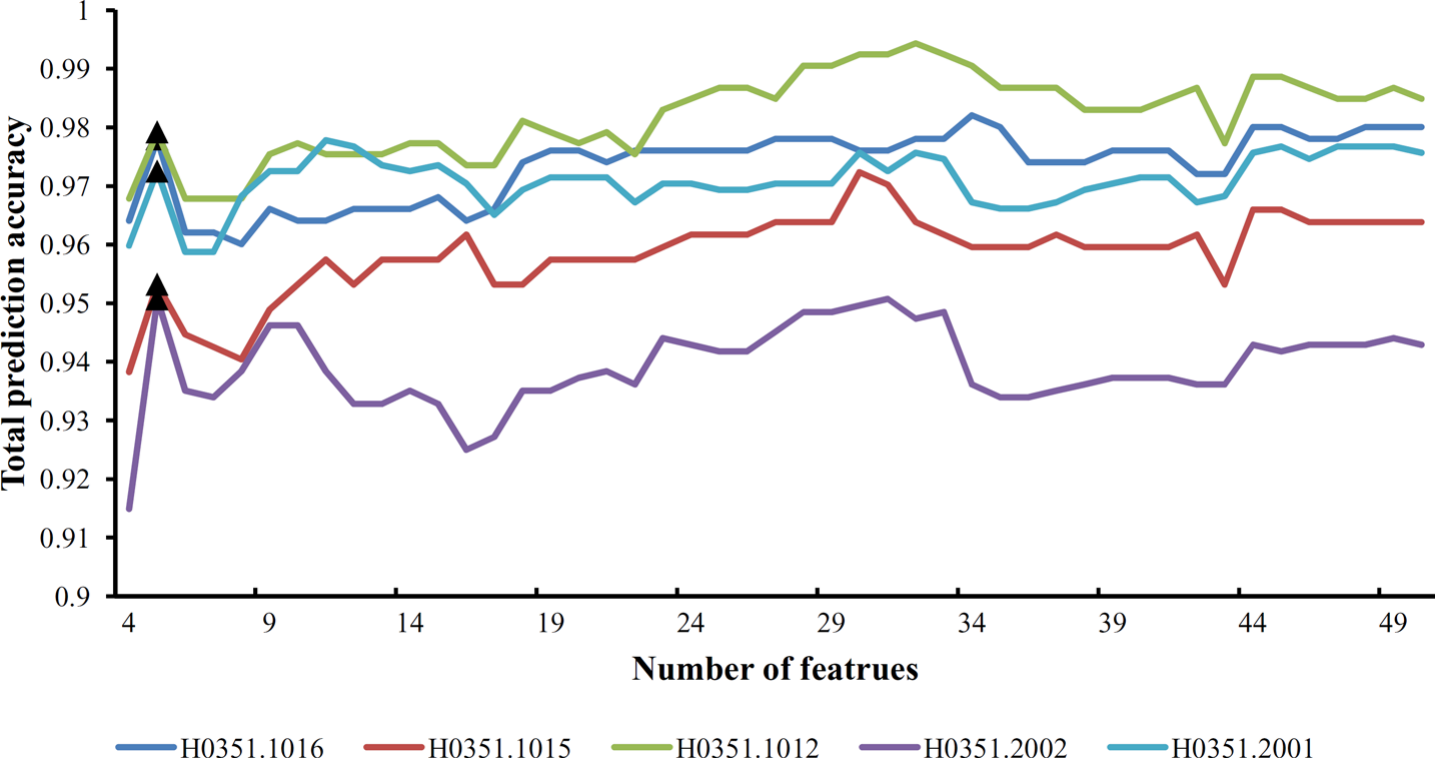
Parts of five IFS-curves using the data of H0351.1009 as the training dataset and the data from other people as the test dataset. The triangle in each curve represents the inflection point.

**Fig 3:**
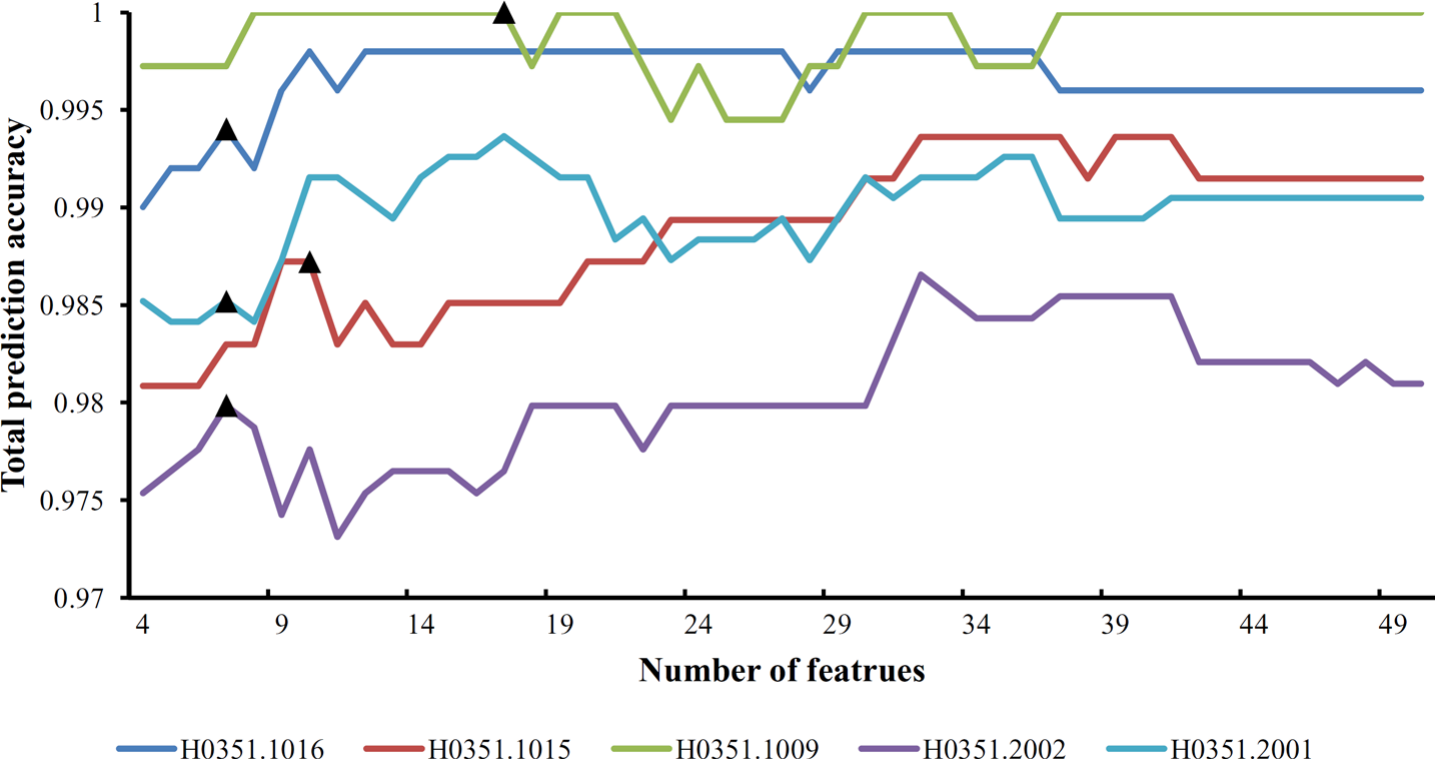
Parts of five IFS-curves using the data of H0351.1012 as the training dataset and the data from other people as the test dataset. The triangle in each curve represents the inflection point.

**Fig 4:**
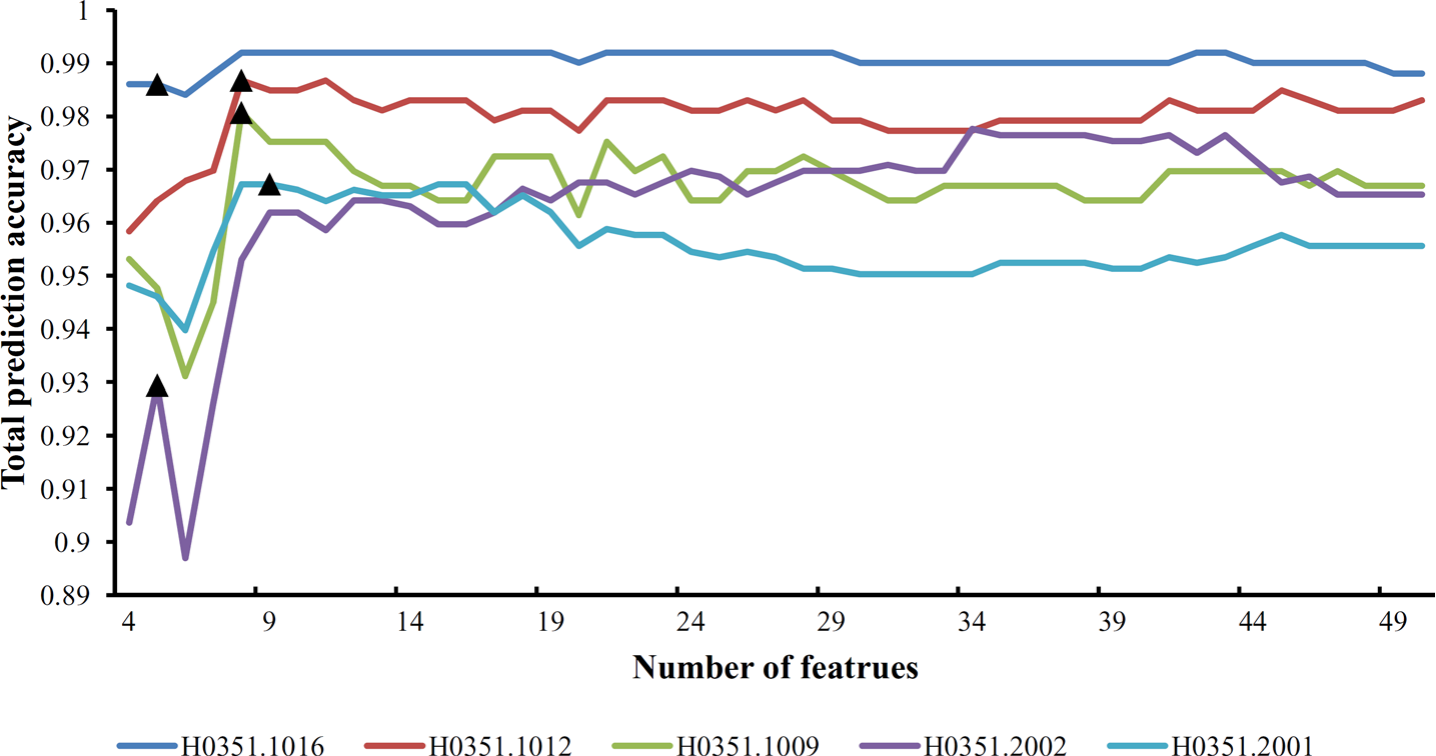
Parts of five IFS-curves using the data of H0351.1015 as the training dataset and the data from other people as the test dataset. The triangle in each curve represents the inflection point.

**Fig 5:**
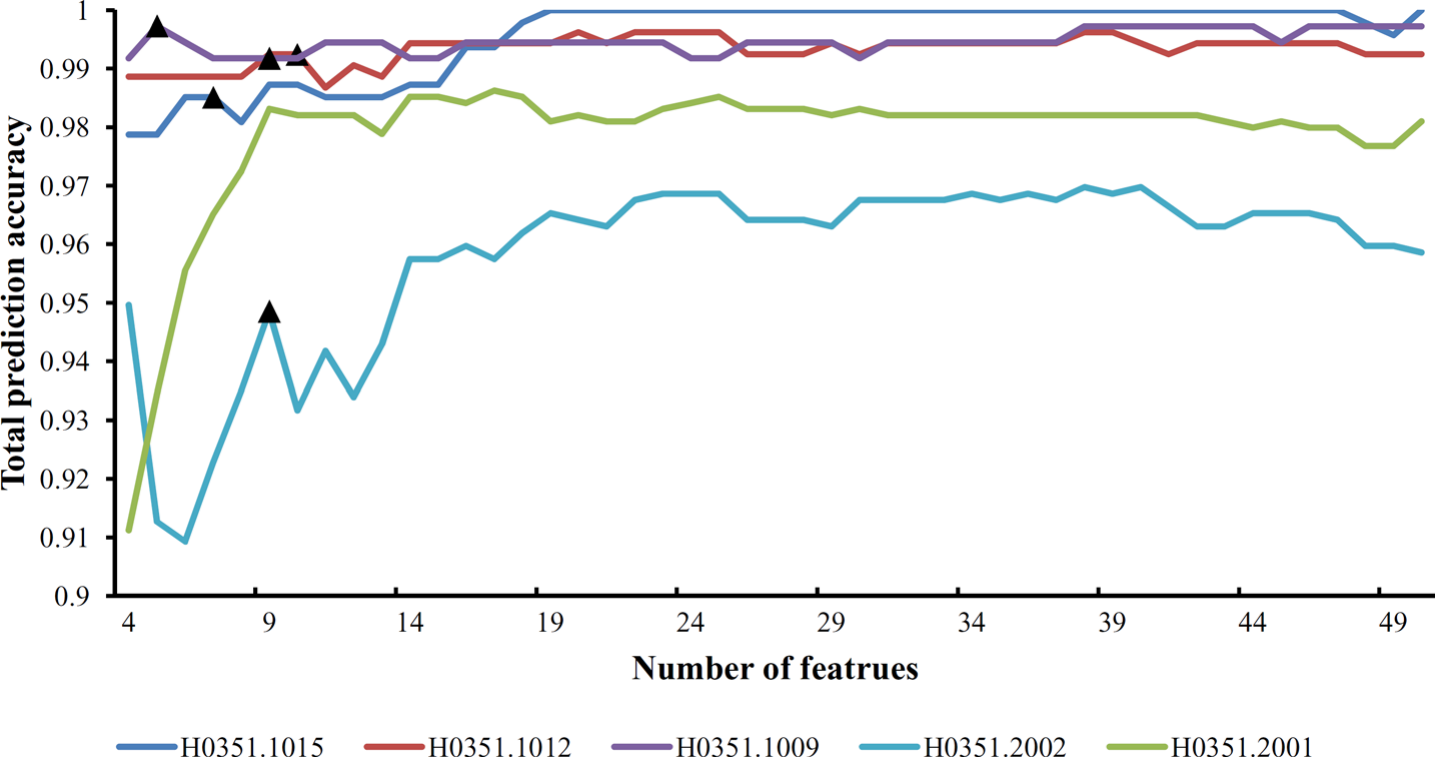
Parts of five IFS-curves using the data of H0351.1016 as the training dataset and the data from other people as the test dataset. The triangle in each curve represents the inflection point.

**Fig 6:**
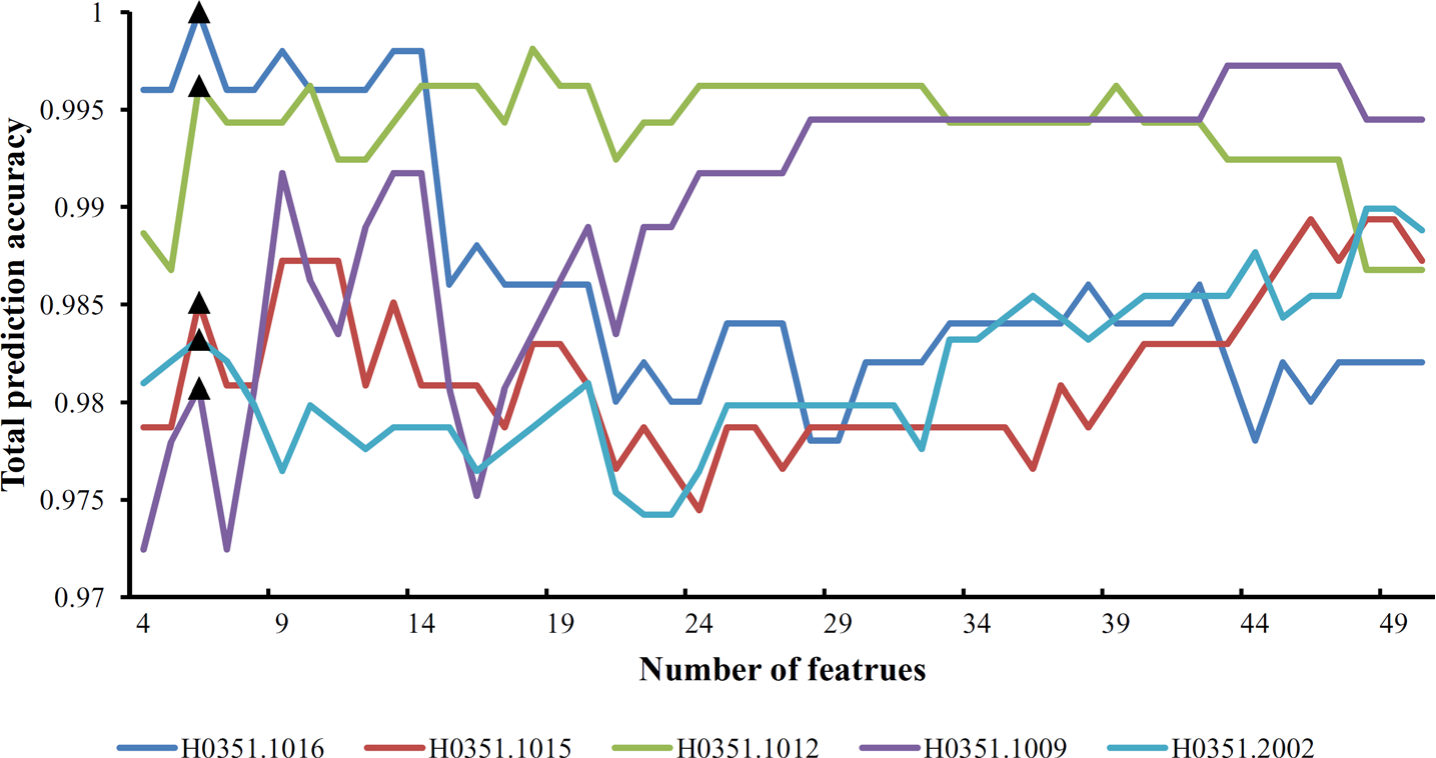
Parts of five IFS-curves using the data of H0351.2001 as the training dataset and the data from other people as the test dataset. The triangle in each curve represents the inflection point.

**Fig 7:**
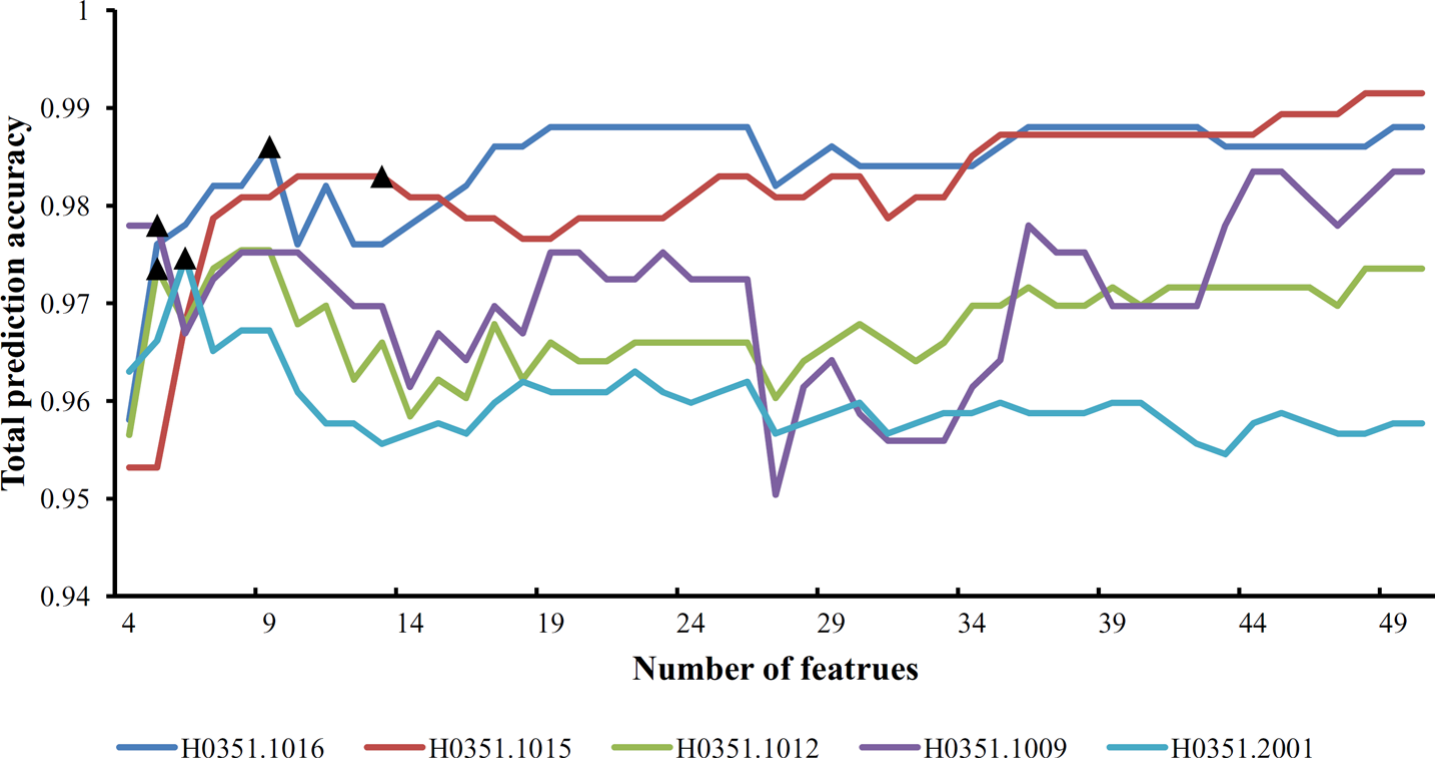
Parts of five IFS-curves using the data of H0351.2002 as the training dataset and the data from other people as the test dataset. The triangle in each curve represents the inflection point.

**Fig 8:**
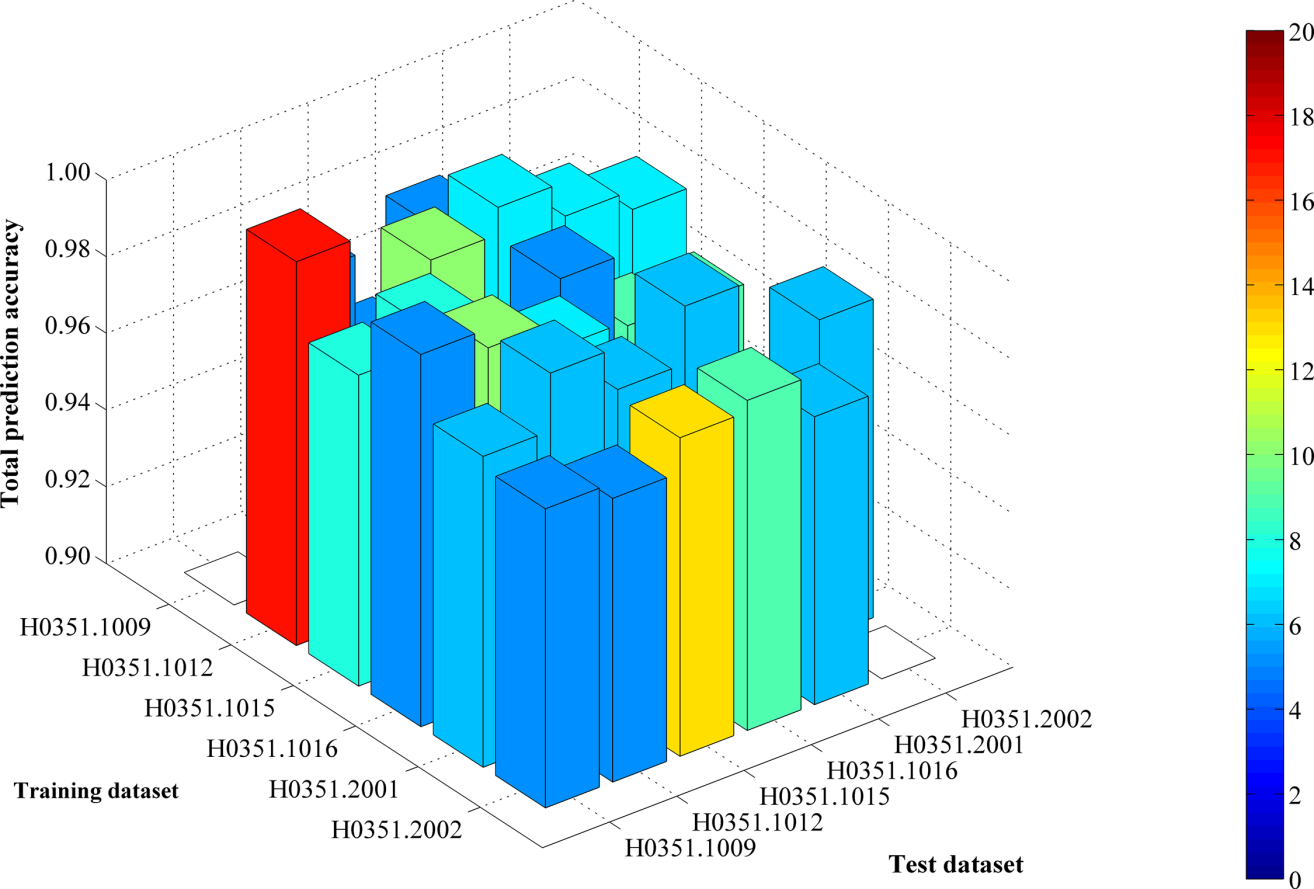
A 3-D histogram illustrating the number of features of each inflection point and the corresponding total prediction accuracy. The height of the bar represents the total prediction accuracy, whereas the color of the bar represents the number of features of each inflection point.

From S3 Table and **Fig 1**, we can see that the feature set yielding the maximum total prediction accuracy always contains a large number of features (genes), which makes it difficult to further analyze their importance. Thus, as mentioned in Section “Revised IFS method”, we selected an inflection point in each IFS curve. To do that, we amplified each IFS curve between 4 and 50 on the X-axis, as illustrated in **Figs 2–7**. The obtained inflection points and their corresponding total prediction accuracies are presented in S4 Table. Additionally, a 3-D histogram was plotted in **Fig 8** to show the number of features of each inflection point and the corresponding total prediction accuracy. It can be observed from S2 Table and **Fig 8** that the number of selected features (genes) at the inflection point was, in most cases, greatly reduced.

Because there is one mRMR feature list for a given training dataset and five inflection points for five test datasets, *i*.*e*., we can obtain five sets of the selected features, we took the intersection operation of these sets to extract important features for a given training dataset. Thus, six sets of important features were obtained, involving 20 features (genes), which are listed in column 1 of **Table 2**. To further demonstrate the importance of these 20 genes, the frequency at which each gene occurred in the six important feature sets was counted, as shown in column 3 of **Table 2**. It can be seen that some genes, such as NR2E1, DAO, and LRRC7, occurred several times, indicating that these genes are the most important for evaluating the differences between the brain regions of different people. The following section provides evidence supporting our results, indicating that our method is effective.

**Table 2:** Frequencies of the selected genes.

**Gene symbol**	**Description**	**Frequency**
NR2E1	Nuclear Receptor Subfamily 2, Group E, Member 1	5
DAO	D-Amino-Acid Oxidase	4
LRRC7	Leucine Rich Repeat Containing 7	3
HOXA2	Homeobox A2	2
PAX3	Paired Box 3	2
PCP2	Purkinje Cell Protein 2	2
TFAP2B	Transcription Factor AP-2 Beta (Activating Enhancer Binding Protein 2 Beta)	2
ABLIM1	Actin Binding LIM Protein 1	1
ARHGAP4	Rho GTPase Activating Protein 4	1
FLJ43663	Estrogen Receptor 1	1
HOXA3	Homeobox A3	1
HOXB2	Homeobox B2	1
KHDRBS1	KH Domain Containing, RNA Binding, Signal Transduction Associated 1	1
KLK8	Kallikrein-Related Peptidase 8	1
LOC100287521	—	1
NECAB1	N-Terminal EF-Hand Calcium Binding Protein 1	1
SEC13	Homolog of SEC13 (*S*. *cerevisiae*)	1
STON1	Stonin 1	1
TRAF3IP1	TNF Receptor-Associated Factor 3 Interacting Protein 1	1
WDR48	WD Repeat Domain 48	1

### Comparison with previous Allen Human Brain Atlas analysis

The Allen Human Brain Atlas dataset has been analyzed by Myers *et al*. [9]. Comparing with their study, there were several major improvements in our work.

First, they only used t-test to identify the differentially expressed genes. We adopted the state-of-art machine learning based feature selection method to select the optimal discriminative genes.

Second, they only did statistical test without prediction model. Beside the discriminative genes, the brain region prediction models were constructed in this work.

Third, they did not do cross-individual analysis which is very important for measuring the robustness of selected genes and for analyzing the subtle difference among individuals.

### Analysis of the selected genes

In this study, we analyzed the gene expression profiles from three brain regions of six people of different races, ages and sexes. To explore general trends in regionally expressed genes in human brains, we used feature selection methods, specifically the mRMR method and the IFS method, to analyze the gene expression profiles. As a result, 20 genes, along with their frequencies, were identified and are listed in **Table 2**. Here, we provide evidence to confirm our finding that these genes are differentially expressed in the brain and important for evaluating the differences between the brain regions of different people.

#### NR2E1

NR2E1 (nuclear receptor subfamily 2, group E, member 1) was ranked first on our list with a ‘frequency’ of 5. It has been shown to be expressed in a highly regionalized pattern in the brain, and it has important physiological functions in brain development and patterning [36, 37]. Deletion of the *Nr2e1* gene in mouse models results in disorders in brain (hypoplasia of cerebrum and olfactory lobes, abnormal synaptic plasticity and dendritic structure in the mouse dentate gyrus) and eye development and in behavioral abnormalities [38, 39]. Other studies have also suggested that NR2E1 controls the self-renewal of neural stem cells (NSCs) and is involved in the initiation of brain tumors [40].

#### DAO

DAO (D-amino-acid oxidase) had a ‘frequency’ of 4 and ranked second on our list. The regional expression and distribution of the DAO protein in the rat brain has been investigated and reported in several previous studies [41, 42]. Hindbrain neurons, especially Golgi and Purkinje cells, have been shown to express higher levels of DAO than forebrain neurons. In our study, DAO was also shown to be expressed in a region-specific manner in all 6 human brains. The DAO protein in the rat brain was postulated to function in the elimination of its substrate, D-serine [43], which has been shown to be a major endogenous coagonist of the N-methyl D-aspartate (NMDA) type of glutamate receptors [44]. Because a higher level of DAO is found in the hindbrain, D-serine is more abundant in the forebrain [45]. Previous studies have shown that the hypofunction of the N-Methyl-D-Aspartate (NMDA) type of glutamate receptors in the brain might lead to symptoms similar to those seen in schizophrenia [46].

#### LRRC7

LRRC7 (leucine rich repeat containing 7, also known as DENSIN) had a ‘frequency’ of 3 and ranked third on our list. The LRRC7 protein was first purified from rat brains and was identified as a brain-specific synaptic protein of the O-sialoglycoprotein family [47]. Rat densin was more abundant in the forebrain than in the cerebellum [47], which was consistent with our finding that LRRC7 was differentially expressed in the human brain. In the postsynaptic density (PSD), densin can form a high-affinity functional complex with αCaMKII and α-actinin [48]. Loss of densin results in reduced levels of alpha-actinin in the brain and reduced localization of mGluR5 and DISC1 in the PSD fraction, which may play a role in the behavioral endophenotypes of mental illness, as revealed in *Lrrc7* null-mutation mouse models [48]. Interestingly, LRRC7 was observed to be regionally expressed in the brains of the two African American men and the Hispanic woman but not in the three Caucasian men, which suggested that the differential brain expression of LRRC7 might also somewhat be related to race.

#### Hox family genes

Three Hox (homeobox) family genes were on our list, including HOXA2, with a ‘frequency’ of 2, and HOXA3 and HOXB2, each with a ‘frequency’ of 1. Differential expression of these Hox family genes was observed in some of the human brains (*e*.*g*., HOXA2 and HOXB2 in the 31-year-old Caucasian and 24-year-old African American men, HOXA3 in the 39-year-old African American man, and HOXA4 in the 57-year-old Caucasian man). Hox genes play pivotal roles in postembryonic brain development and regionalization through their spatiotemporal expression in the central nervous system [49, 50]. Similarly, in our study, HOXA2 and HOXB2 were among the top differentially expressed genes in younger people (age 24 and 31), irrespective of race. In contrast, other Hox family genes were differentially expressed in older people (age 39 and 57).

Besides the above genes, we also found experimental results supporting the regional expression pattern of several other genes in our list, including PAX3 [51, 52], PCP2 [53], ARHGAP4 [54] and NECAB1 [55]. These lines of evidence indicated the effectiveness of our method.

Using our method, we have also identified genes with previously unknown regional expression patterns in the brain, such as TFAP2B, ABLIM1, KLK8, SEC13 and STON1. Our study shed light on the potential important biological functions of these genes in the human brain. Here, we took TFAP2B as an example and gave our analyses.

#### TFAP2B

The transcription factor AP-2 beta (TFAP2B, also known as AP-2B or AP2-B) was another potential race-specific gene, and it was only observed to be differentially expressed in the brains of the two Caucasians (age 31 and 57) and not the two African American men or the Hispanic woman. TFAP2B encodes a transcription factor expressed in neural crest cells, stimulating cell proliferation and suppressing terminal differentiation [56]. Interestingly, loss of TFAP2B has been reported to be linked to several race-specific syndromes and diseases. For example, mutation of TFAP2B has been shown to be related to Char syndrome, a familial form of facial dysmorphism, as well as to patent ductus arteriosus (PDA) and hand anomalies (aplasia or hypoplasia of the middle phalanges of the fifth fingers) [57]. Other studies observed mutations of TFAP2B in similar congenital heart disease patients in southern China [58] and south India [59]. These lines of evidence suggest that genetic polymorphism of TFAP2B plays a role in various diseases.

## Conclusions

This study investigated the gene expression profiles of three human brain regions from six people of different races, ages, and sexes. Utilizing two major feature selection methods, a number of key genes were identified. These findings may contribute to further studies aimed at elucidating the mechanisms of development of the human brain from a genomics perspective.

## Supporting Information

S1 Fig Five IFS curves using the data of H0351.1009 as the training dataset and the data from other people as the test dataset. (TIF)

S2 Fig Five IFS curves using the data of H0351.1012 as the training dataset and the data from other people as the test dataset. (TIF)

S3 Fig Five IFS curves using the data of H0351.1015 as the training dataset and the data from other people as the test dataset. (TIF)

S4 Fig Five IFS curves using the data of H0351.1016 as the training dataset and the data from other people as the test dataset. (TIF)

S5 Fig Five IFS curves using the data of H0351.2001 as the training dataset and the data from other people as the test dataset. (TIF)

S6 Fig Five IFS curves using the data of H0351.2002 as the training dataset and the data from other people as the test dataset. (TIF)

S1 Table mRMR results for the gene expression profiles of human brains from six individuals. (DOCX)

S2 Table The accuracies obtained using the revised IFS method. (DOCX)

S3 Table The numbers of features (genes) yielding the maximum total prediction accuracy for pairs of training and test datasets. (DOCX)

S4 Table The inflection point for each IFS curve and its corresponding total prediction accuracy. (DOCX)

S1 Text Detailed information about the six individuals whose samples were studied. (PDF)
