# Supplementary figures and images for: Analysis of Gene Expression Profiles in the Human Brain Stem, Cerebellum and Cerebral Cortex
Source: PLoS One. 2016 Jul 19;11(7):e0159395. doi: 10.1371/journal.pone.0159395 (PMC4951119; doi:10.1371/journal.pone.0159395)

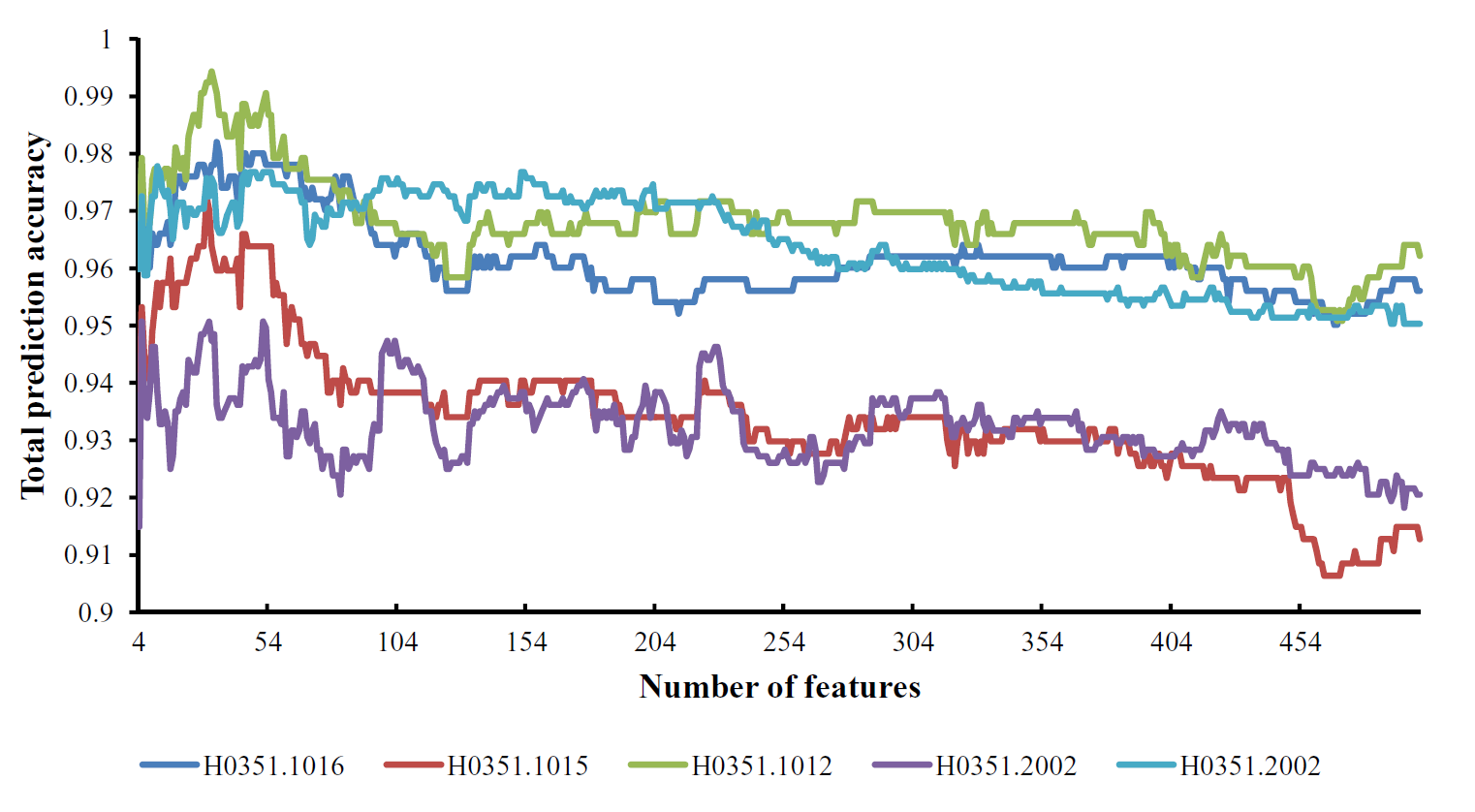

Supplement: S1 Fig — (TIF) [file pone.0159395.s001.tif]

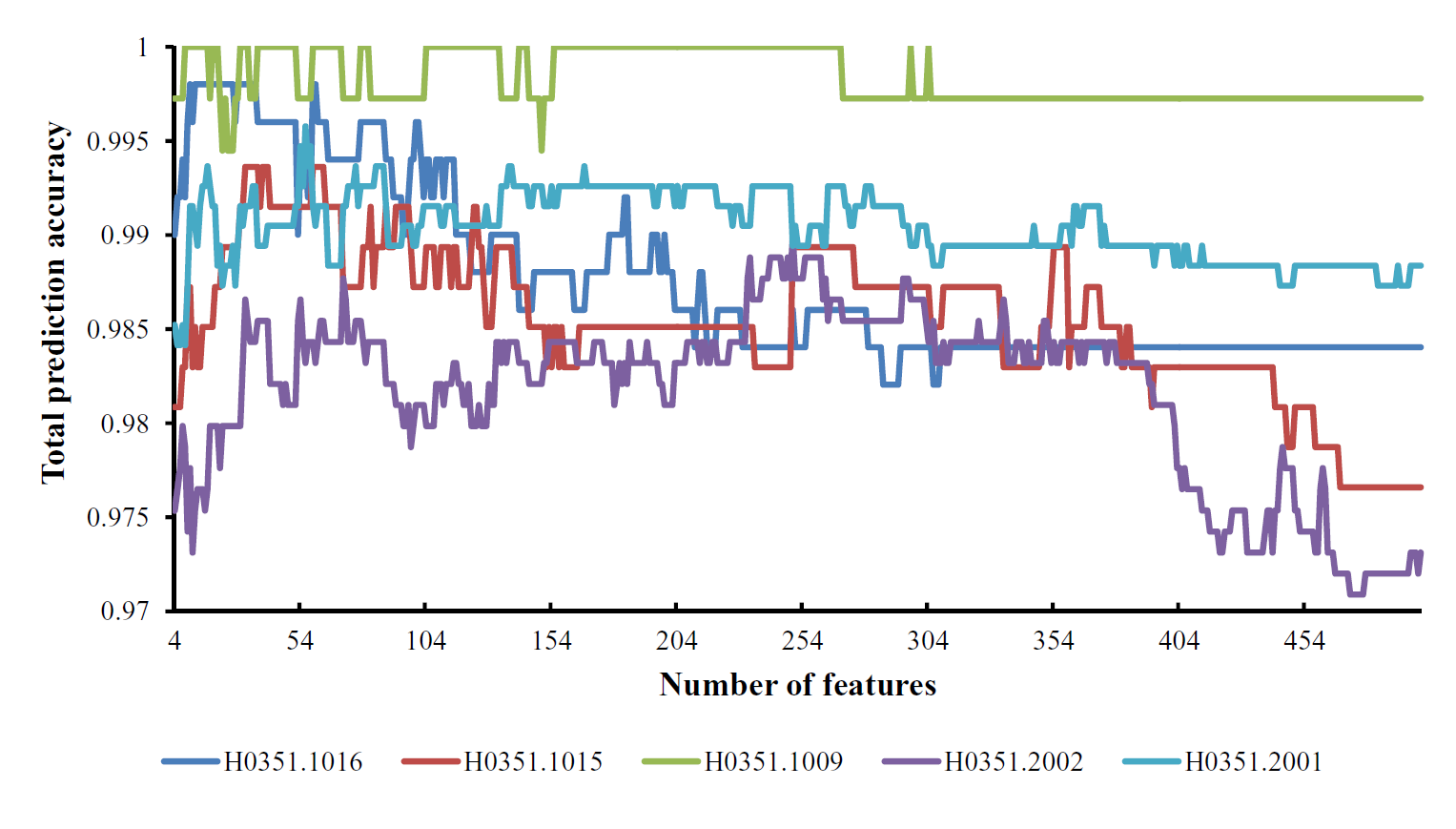

Supplement: S2 Fig — (TIF) [file pone.0159395.s002.tif]

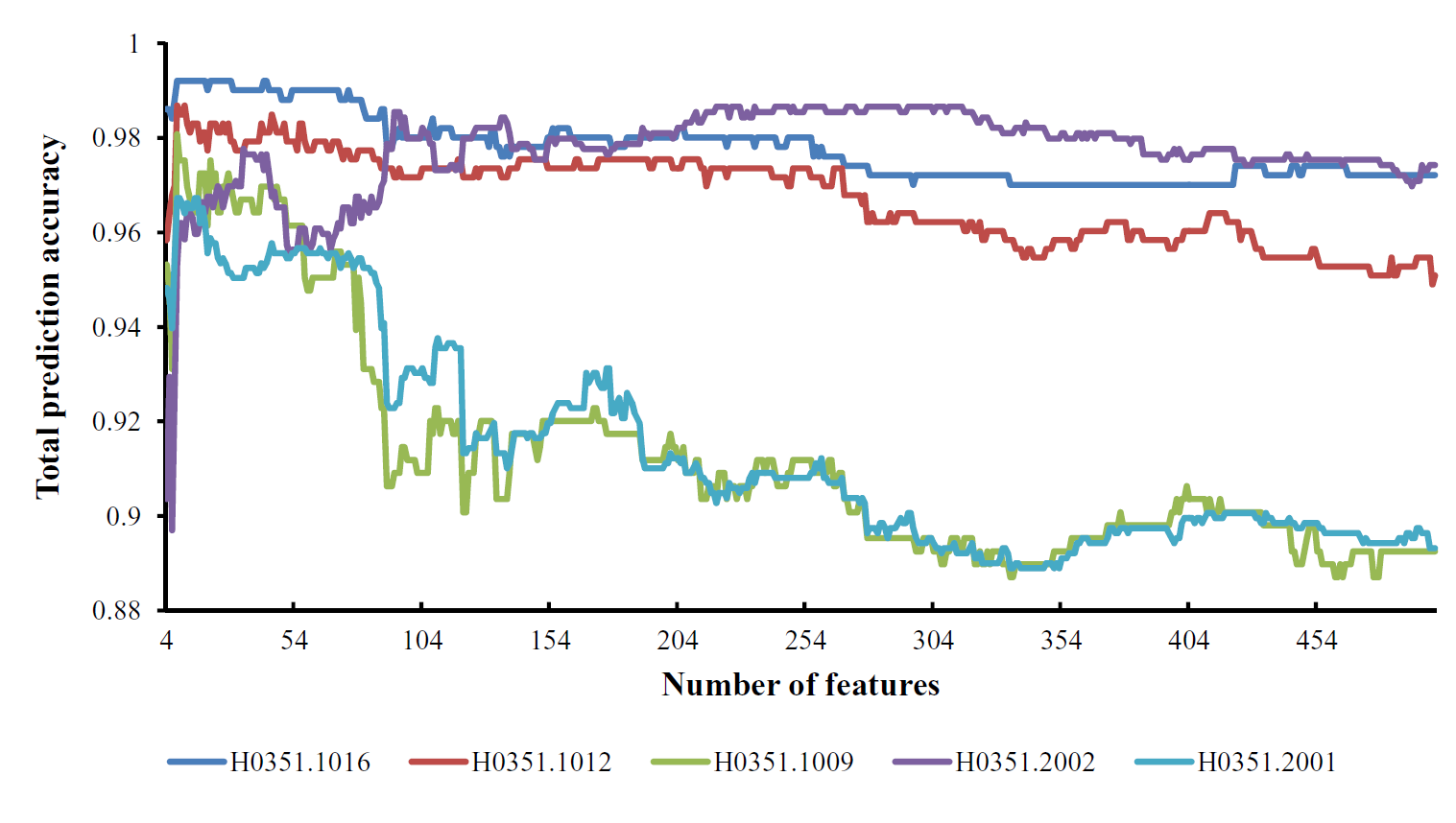

Supplement: S3 Fig — (TIF) [file pone.0159395.s003.tif]

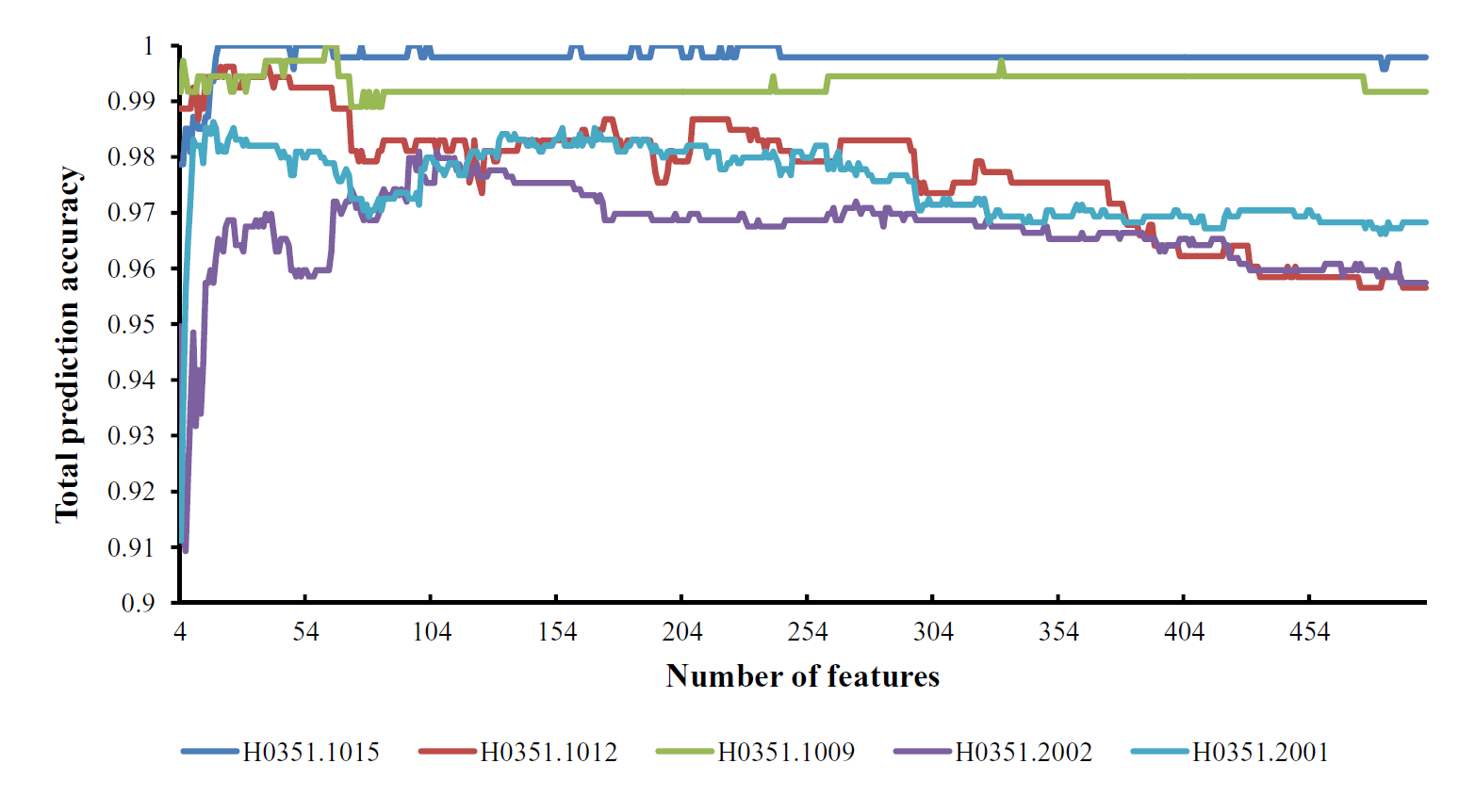

Supplement: S4 Fig — (TIF) [file pone.0159395.s004.tif]

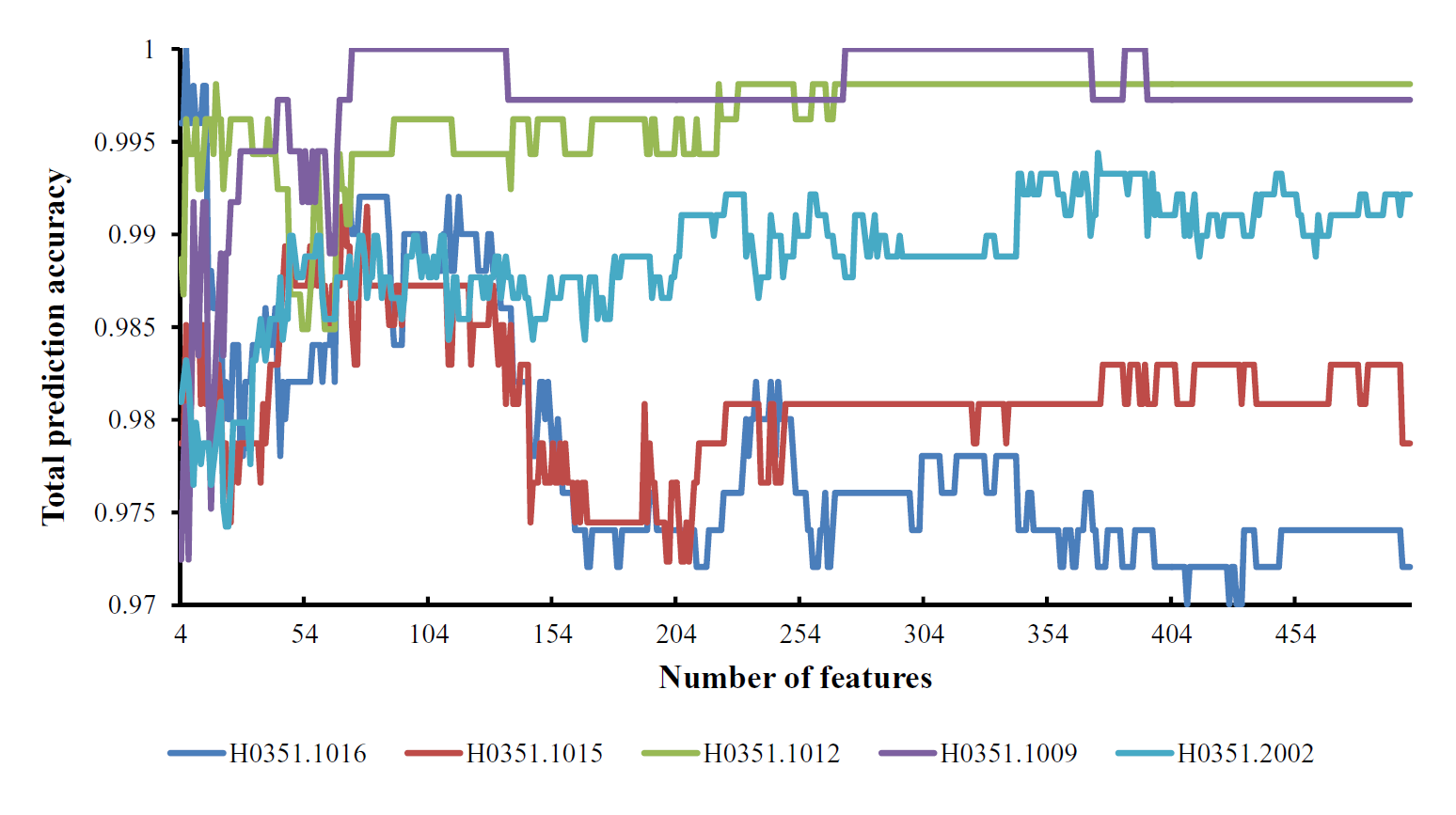

Supplement: S5 Fig — (TIF) [file pone.0159395.s005.tif]

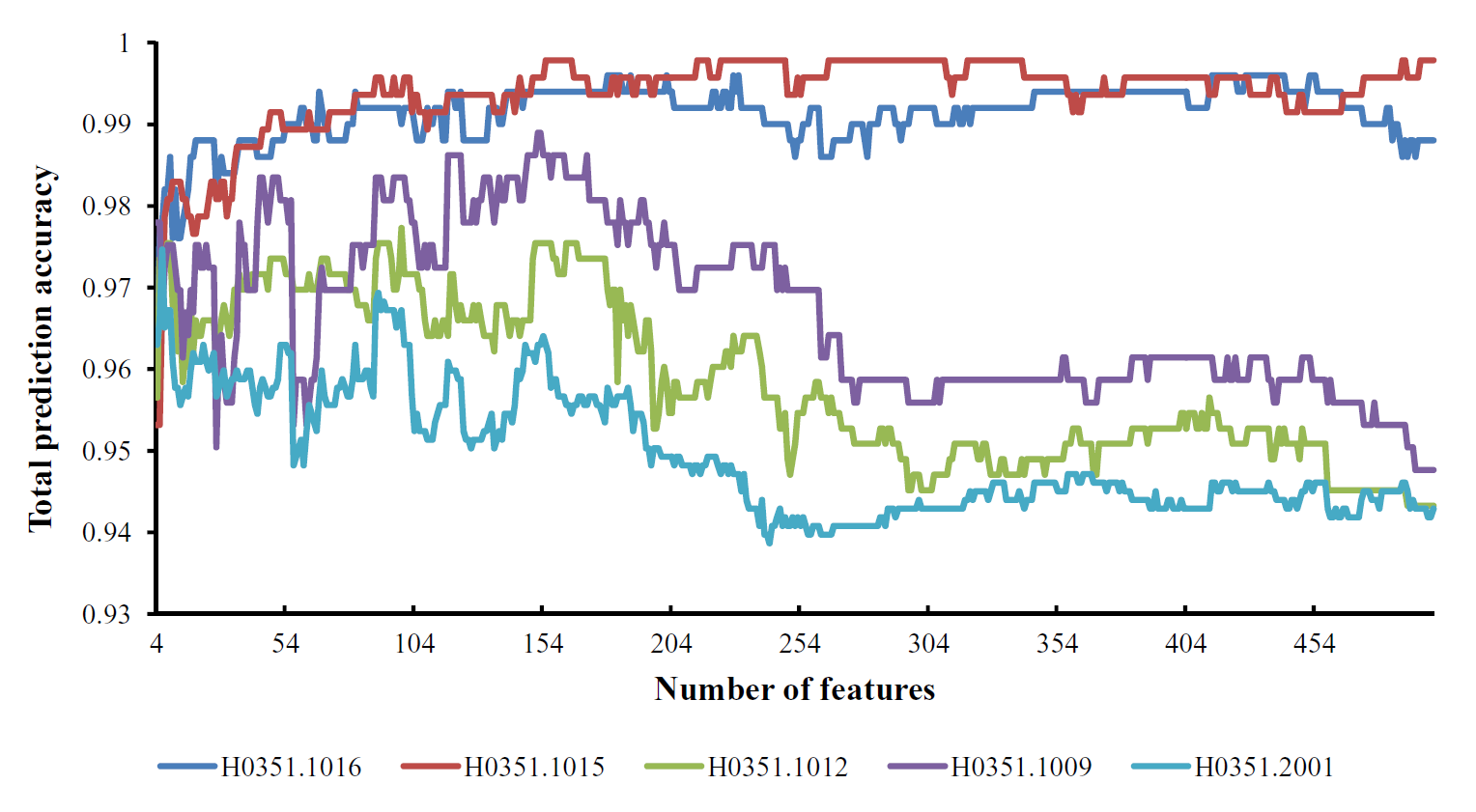

Supplement: S6 Fig — (TIF) [file pone.0159395.s006.tif]
