# Supplementary material for: Analysis of Gene Expression Profiles in the Human Brain Stem, Cerebellum and Cerebral Cortex
Source: PLoS One. 2016 Jul 19;11(7):e0159395. doi: 10.1371/journal.pone.0159395 (PMC4951119; doi:10.1371/journal.pone.0159395)
Supplement: S1 Table — (DOCX) [file pone.0159395.s007.docx]

**S1 Table.** mRMR results for the gene expression profiles of human brains from six individuals.

1. **H0351.1009**

**(I) MaxRel feature list**

| Order | Name |
| --- | --- |
| 1 | NR2E1 |
| 2 | DAO |
| 3 | MEIS1 |
| 4 | LRRC7 |
| 5 | FLJ42875 |
| 6 | NISCH |
| 7 | GABRA5 |
| 8 | KHDRBS1 |
| 9 | NRGN |
| 10 | RAB26 |
| 11 | SIPA1L2 |
| 12 | TUBGCP6 |
| 13 | SLC1A6 |
| 14 | CHD7 |
| 15 | SLC26A10 |
| 16 | CCNG2 |
| 17 | ABTB1 |
| 18 | ZIC4 |
| 19 | TARBP2 |
| 20 | PLK2 |
| 21 | DNMT1 |
| 22 | SEL1L3 |
| 23 | NECAB2 |
| 24 | KIF17 |
| 25 | STK10 |
| 26 | CNDP2 |
| 27 | LHX1 |
| 28 | SYNE4 |
| 29 | PDK2 |
| 30 | CCK |
| 31 | SPSB3 |
| 32 | KIAA1456 |
| 33 | GAS2 |
| 34 | PCDHA8 |
| 35 | ANK1 |
| 36 | KHDRBS2 |
| 37 | BARHL1 |
| 38 | SRGAP2 |
| 39 | CLK4 |
| 40 | RBP4 |
| 41 | STON1 |
| 42 | XAF1 |
| 43 | AMT |
| 44 | CBLN1 |
| 45 | ZNF521 |
| 46 | SLC8A2 |
| 47 | MLL4 |
| 48 | MPP3 |
| 49 | ETV1 |
| 50 | MX1 |
| 51 | HINFP |
| 52 | FGFR1 |
| 53 | CDC25B |
| 54 | PTK2B |
| 55 | UNCX |
| 56 | SETDB1 |
| 57 | INADL |
| 58 | CAST |
| 59 | RBFOX3 |
| 60 | PDE2A |
| 61 | GLUD1 |
| 62 | NOL11 |
| 63 | SDHAF1 |
| 64 | NUP85 |
| 65 | CHRD |
| 66 | PCSK9 |
| 67 | COL27A1 |
| 68 | FAM81A |
| 69 | KALRN |
| 70 | UTRN |
| 71 | RBM4 |
| 72 | MMD |
| 73 | HSPB8 |
| 74 | GLCE |
| 75 | ARHGAP4 |
| 76 | ALCAM |
| 77 | FOXA2 |
| 78 | DLX6-AS1 |
| 79 | C16orf11 |
| 80 | TFAP2B |
| 81 | ILF3 |
| 82 | PKIB |
| 83 | SERPINI1 |
| 84 | CDH8 |
| 85 | ABR |
| 86 | CCT7 |
| 87 | C11orf58 |
| 88 | CAMK4 |
| 89 | AK5 |
| 90 | NXF1 |
| 91 | PODXL |
| 92 | RASAL1 |
| 93 | RNF112 |
| 94 | NIPA1 |
| 95 | HPCAL1 |
| 96 | TMEM61 |
| 97 | RCAN3 |
| 98 | CADPS2 |
| 99 | SST |
| 100 | SPATA5L1 |
| 101 | CREG2 |
| 102 | DUSP12 |
| 103 | NEUROD1 |
| 104 | PPIP5K1 |
| 105 | DNAH7 |
| 106 | FAT2 |
| 107 | GNB3 |
| 108 | COL13A1 |
| 109 | LINC00473 |
| 110 | GDA |
| 111 | RNF115 |
| 112 | CAMKK2 |
| 113 | RBMS3 |
| 114 | DLX1 |
| 115 | SYT5 |
| 116 | FOXS1 |
| 117 | KIAA1024 |
| 118 | PAX3 |
| 119 | NECAB1 |
| 120 | RGS4 |
| 121 | LHX6 |
| 122 | RHOQ |
| 123 | BCAS1 |
| 124 | C20orf3 |
| 125 | ABLIM1 |
| 126 | GALNT7 |
| 127 | AMICA1 |
| 128 | LOC100287005 |
| 129 | SYTL1 |
| 130 | MAMDC4 |
| 131 | ARHGAP29 |
| 132 | CEP76 |
| 133 | IL16 |
| 134 | TIMP2 |
| 135 | ZDHHC24 |
| 136 | BAIAP3 |
| 137 | DDA1 |
| 138 | THUMPD2 |
| 139 | EEF1DP3 |
| 140 | DHPS |
| 141 | MFSD10 |
| 142 | ZNF384 |
| 143 | CDH22 |
| 144 | PCP2 |
| 145 | RASGRF2 |
| 146 | SLC35F4 |
| 147 | HTR2A |
| 148 | LBH |
| 149 | NGEF |
| 150 | GTDC2 |
| 151 | KCNF1 |
| 152 | CMTM7 |
| 153 | VSIG8 |
| 154 | KCNJ3 |
| 155 | LDLRAP1 |
| 156 | EGLN3 |
| 157 | VPS45 |
| 158 | GNAL |
| 159 | GFOD2 |
| 160 | SHC3 |
| 161 | MCF2 |
| 162 | TRAF3IP1 |
| 163 | ZNF187 |
| 164 | LDB2 |
| 165 | B9D2 |
| 166 | TIMP4 |
| 167 | NELF |
| 168 | SLC22A23 |
| 169 | BTN2A2 |
| 170 | TPCN2 |
| 171 | FLRT2 |
| 172 | WSCD2 |
| 173 | CSNK2B |
| 174 | FBXL5 |
| 175 | TMEM229B |
| 176 | C16orf79 |
| 177 | CRTAM |
| 178 | NCALD |
| 179 | PRPF6 |
| 180 | PISD |
| 181 | DUSP22 |
| 182 | C2orf80 |
| 183 | CCDC152 |
| 184 | ZP1 |
| 185 | USP45 |
| 186 | RRAGD |
| 187 | PPT2 |
| 188 | CDIPT |
| 189 | SPP1 |
| 190 | CPT1A |
| 191 | RNFT2 |
| 192 | LOC541471 |
| 193 | PRPSAP2 |
| 194 | EPS8L2 |
| 195 | EPB41L4A |
| 196 | MAB21L1 |
| 197 | LOC643037 |
| 198 | LOC283174 |
| 199 | CCDC107 |
| 200 | SELO |
| 201 | SHROOM3 |
| 202 | LINC00152 |
| 203 | ATP4A |
| 204 | PRORSD1P |
| 205 | GTF3C5 |
| 206 | SLIT2 |
| 207 | CBLB |
| 208 | DOPEY2 |
| 209 | CAMKV |
| 210 | SPTBN5 |
| 211 | COL11A1 |
| 212 | ZNF266 |
| 213 | TOP1MT |
| 214 | WNT10B |
| 215 | FRMD6 |
| 216 | CRIP3 |
| 217 | OTX1 |
| 218 | CXCL14 |
| 219 | SYT17 |
| 220 | LAMA5 |
| 221 | STOX1 |
| 222 | BTG1 |
| 223 | GRID2 |
| 224 | MTO1 |
| 225 | UG0898H09 |
| 226 | PTCHD1 |
| 227 | DECR2 |
| 228 | ADAMTS19 |
| 229 | TNS1 |
| 230 | NETO1 |
| 231 | ARMC8 |
| 232 | PCDH11Y |
| 233 | RELN |
| 234 | C16orf93 |
| 235 | KIAA1598 |
| 236 | ZIC1 |
| 237 | CTDSPL2 |
| 238 | LOC150622 |
| 239 | NEFM |
| 240 | DYSF |
| 241 | RGS14 |
| 242 | C10orf54 |
| 243 | SNORA70 |
| 244 | LRCH1 |
| 245 | CCDC88A |
| 246 | FAM5B |
| 247 | EIF5A2 |
| 248 | CHRM3 |
| 249 | PAXIP1 |
| 250 | ENY2 |
| 251 | LOC100287291 |
| 252 | LINC00176 |
| 253 | RMND5A |
| 254 | JMJD7 |
| 255 | LOC100287347 |
| 256 | B9D1 |
| 257 | QSOX2 |
| 258 | EPB41L4A-AS1 |
| 259 | LIMA1 |
| 260 | COL6A1 |
| 261 | KIAA0247 |
| 262 | GSTT2 |
| 263 | NUDT14 |
| 264 | MGARP |
| 265 | LTA4H |
| 266 | LHX2 |
| 267 | PPFIA2 |
| 268 | RIIAD1 |
| 269 | ZNF385D |
| 270 | NRG3 |
| 271 | PILRA |
| 272 | C8orf46 |
| 273 | AGPAT6 |
| 274 | MBP |
| 275 | PRICKLE2 |
| 276 | TPRA1 |
| 277 | RANBP3L |
| 278 | HRH1 |
| 279 | ZFAND2B |
| 280 | NLK |
| 281 | MDGA1 |
| 282 | MICAL2 |
| 283 | BCAT1 |
| 284 | RASL10A |
| 285 | PPFIBP1 |
| 286 | MAST3 |
| 287 | RNF152 |
| 288 | PLK5 |
| 289 | LOC729683 |
| 290 | ARHGEF10L |
| 291 | TRIM50 |
| 292 | CCDC14 |
| 293 | RALB |
| 294 | KIAA0895L |
| 295 | ELP4 |
| 296 | CLK2 |
| 297 | DDX46 |
| 298 | MRVI1 |
| 299 | QPCT |
| 300 | ICAM5 |
| 301 | LRRC4C |
| 302 | GRM4 |
| 303 | FAM131C |
| 304 | EPB41L1 |
| 305 | THOC3 |
| 306 | PCSK1 |
| 307 | OR7E156P |
| 308 | IL11RA |
| 309 | HPS4 |
| 310 | SIRT7 |
| 311 | RBM5 |
| 312 | HRASLS |
| 313 | EDIL3 |
| 314 | GBP4 |
| 315 | GAB2 |
| 316 | ZNF503 |
| 317 | EIF3E |
| 318 | C10orf107 |
| 319 | TLL1 |
| 320 | ITPKA |
| 321 | CLIP1 |
| 322 | SHF |
| 323 | TDRD6 |
| 324 | GPRC5C |
| 325 | TH1L |
| 326 | PHF17 |
| 327 | PDLIM2 |
| 328 | PILRB |
| 329 | CNPY1 |
| 330 | SLC48A1 |
| 331 | ABRACL |
| 332 | C9orf72 |
| 333 | DGKD |
| 334 | LRRTM4 |
| 335 | TAC3 |
| 336 | PDZK1P1 |
| 337 | H2BFXP |
| 338 | POLB |
| 339 | TMSB10 |
| 340 | ATRNL1 |
| 341 | SLC17A6 |
| 342 | SUV420H2 |
| 343 | RRAS |
| 344 | DPF3 |
| 345 | FHOD3 |
| 346 | TMEM163 |
| 347 | USP47 |
| 348 | APOO |
| 349 | CCDC12 |
| 350 | GABRA6 |
| 351 | FAM123C |
| 352 | HDAC1 |
| 353 | TMEM62 |
| 354 | TMEM249 |
| 355 | EPB41L2 |
| 356 | CNTLN |
| 357 | ZNF134 |
| 358 | FAM161B |
| 359 | SKIL |
| 360 | TLE2 |
| 361 | LOC100133299 |
| 362 | LPPR5 |
| 363 | FSTL5 |
| 364 | SPSB1 |
| 365 | LCOR |
| 366 | CPM |
| 367 | TNFRSF6B |
| 368 | RORB |
| 369 | ANLN |
| 370 | TIAM1 |
| 371 | CRIP2 |
| 372 | B3GALT2 |
| 373 | LOC100287987 |
| 374 | WDR83 |
| 375 | CPNE4 |
| 376 | FGF5 |
| 377 | LEPREL1 |
| 378 | NEFL |
| 379 | LEPREL2 |
| 380 | ARNT2 |
| 381 | TRIM67 |
| 382 | TNFRSF25 |
| 383 | CCDC88B |
| 384 | CGN |
| 385 | BACE2 |
| 386 | APEX2 |
| 387 | RSAD2 |
| 388 | EPB41L5 |
| 389 | LINC00115 |
| 390 | ZEB2 |
| 391 | BEST3 |
| 392 | EIF3L |
| 393 | NBPF15 |
| 394 | SYT2 |
| 395 | HIST1H4A |
| 396 | ADAMTS16 |
| 397 | SYT6 |
| 398 | MATK |
| 399 | STAC |
| 400 | ZNF143 |
| 401 | ADAMTS8 |
| 402 | ARSJ |
| 403 | ZNF800 |
| 404 | LOC439914 |
| 405 | LRRC8C |
| 406 | USP44 |
| 407 | FLT3 |
| 408 | ING3 |
| 409 | ZIC5 |
| 410 | MGC16384 |
| 411 | LUZP2 |
| 412 | AKAP13 |
| 413 | TRIM72 |
| 414 | SPA17 |
| 415 | DARC |
| 416 | LZTS1 |
| 417 | UBTD2 |
| 418 | ALS2 |
| 419 | CDKN1C |
| 420 | FAM49A |
| 421 | PAX6 |
| 422 | JMJD1C |
| 423 | EXPH5 |
| 424 | FOXN3 |
| 425 | ANKRD18B |
| 426 | ZNF702P |
| 427 | IQCA1 |
| 428 | C2orf72 |
| 429 | ANO3 |
| 430 | C15orf27 |
| 431 | CST6 |
| 432 | PLA2R1 |
| 433 | CLEC4G |
| 434 | CGNL1 |
| 435 | PTPRN2 |
| 436 | CA10 |
| 437 | LINC00599 |
| 438 | SNRK |
| 439 | SFI1 |
| 440 | KRT18 |
| 441 | SNHG7 |
| 442 | ZNF671 |
| 443 | GLE1 |
| 444 | NRXN2 |
| 445 | TRIM11 |
| 446 | C17orf97 |
| 447 | FCGRT |
| 448 | C5orf46 |
| 449 | CA4 |
| 450 | RSBN1 |
| 451 | DHX38 |
| 452 | CARTPT |
| 453 | SVEP1 |
| 454 | CHN2 |
| 455 | ASTN1 |
| 456 | AMY2A |
| 457 | SLC22A31 |
| 458 | P2RX4 |
| 459 | ELFN2 |
| 460 | PCDH20 |
| 461 | ZNF567 |
| 462 | CCDC111 |
| 463 | PDE8B |
| 464 | PHF11 |
| 465 | FBXL6 |
| 466 | GCOM1 |
| 467 | C1orf198 |
| 468 | CPPED1 |
| 469 | VSTM2L |
| 470 | TFAP2E |
| 471 | LPPR3 |
| 472 | CBS |
| 473 | NRXN3 |
| 474 | B3GAT2 |
| 475 | GALNT12 |
| 476 | SGCA |
| 477 | EBF1 |
| 478 | APBB2 |
| 479 | CNTD2 |
| 480 | CCDC39 |
| 481 | KCTD4 |
| 482 | FDXR |
| 483 | FREM1 |
| 484 | LOC100128822 |
| 485 | PAK7 |
| 486 | MALT1 |
| 487 | SLC25A41 |
| 488 | C16orf86 |
| 489 | TRIM58 |
| 490 | SOX5 |
| 491 | RSAD1 |
| 492 | GRIN2C |
| 493 | CNOT6 |
| 494 | WIPF1 |
| 495 | ARID2 |
| 496 | CERKL |
| 497 | GPR137C |
| 498 | BARHL2 |
| 499 | PCDH10 |
| 500 | ENPP2 |

**(II) mRMR feature list**

| Order | Name |
| --- | --- |
| 1 | NR2E1 |
| 2 | SEC13 |
| 3 | DAO |
| 4 | TFAP2B |
| 5 | KHDRBS1 |
| 6 | HOXA4 |
| 7 | BARHL1 |
| 8 | ILF3 |
| 9 | MEIS1 |
| 10 | PDK2 |
| 11 | STON1 |
| 12 | CSNK2B |
| 13 | CNDP2 |
| 14 | HOXB2 |
| 15 | TARBP2 |
| 16 | PAX3 |
| 17 | FAM129B |
| 18 | NECAB2 |
| 19 | TMEM229B |
| 20 | SPSB3 |
| 21 | HOXA7 |
| 22 | KAZN |
| 23 | STOX1 |
| 24 | RBM4 |
| 25 | HOXA3 |
| 26 | TUBGCP6 |
| 27 | TDRD6 |
| 28 | KIAA1024 |
| 29 | SPATA5L1 |
| 30 | PAX2 |
| 31 | DECR2 |
| 32 | CCDC89 |
| 33 | ANK1 |
| 34 | FBXL22 |
| 35 | OTX1 |
| 36 | TRA2B |
| 37 | TMEM61 |
| 38 | HOXB3 |
| 39 | COL27A1 |
| 40 | ARHGAP29 |
| 41 | ITM2C |
| 42 | KALRN |
| 43 | PRORSD1P |
| 44 | PCP2 |
| 45 | B9D2 |
| 46 | DIS3 |
| 47 | FGFR1 |
| 48 | C16orf11 |
| 49 | HOXD3 |
| 50 | MPP3 |
| 51 | WDR13 |
| 52 | ZP1 |
| 53 | NISCH |
| 54 | TRAF3IP1 |
| 55 | NIM1 |
| 56 | FBXL5 |
| 57 | PCSK9 |
| 58 | HOXA6 |
| 59 | GAS2 |
| 60 | MLL4 |
| 61 | CISD3 |
| 62 | SETDB1 |
| 63 | TFAP2A |
| 64 | EIF5A2 |
| 65 | C11orf58 |
| 66 | HOXA2 |
| 67 | C10orf107 |
| 68 | ABLIM1 |
| 69 | C3orf17 |
| 70 | SYT2 |
| 71 | EIF3E |
| 72 | STK10 |
| 73 | CBLN1 |
| 74 | WDR18 |
| 75 | MSX2P1 |
| 76 | PILRB |
| 77 | LOXL2 |
| 78 | RAB11B |
| 79 | LEPREL1 |
| 80 | LRRC7 |
| 81 | CRTAM |
| 82 | GTF3C5 |
| 83 | PRPH |
| 84 | SPSB1 |
| 85 | B9D1 |
| 86 | PI4KB |
| 87 | DNMT1 |
| 88 | USP47 |
| 89 | SLCO1C1 |
| 90 | SKIL |
| 91 | FOXS1 |
| 92 | ELP4 |
| 93 | ZNF33A |
| 94 | SH2B1 |
| 95 | KLK8 |
| 96 | LHX1 |
| 97 | LTA4H |
| 98 | SPOP |
| 99 | HOXB5 |
| 100 | SRGAP2 |
| 101 | ACPL2 |
| 102 | CHD7 |
| 103 | C17orf70 |
| 104 | NECAB1 |
| 105 | DUSP22 |
| 106 | CAST |
| 107 | QSOX2 |
| 108 | NELF |
| 109 | VSIG8 |
| 110 | TTBK2 |
| 111 | RRAS |
| 112 | LINC00152 |
| 113 | C9orf72 |
| 114 | VPS45 |
| 115 | TM9SF2 |
| 116 | TCTN1 |
| 117 | CSK |
| 118 | IL11RA |
| 119 | CRIP2 |
| 120 | NUP210 |
| 121 | GALNT7 |
| 122 | CARD10 |
| 123 | JMJD7 |
| 124 | GABRA5 |
| 125 | ADAM33 |
| 126 | BTG1 |
| 127 | THUMPD2 |
| 128 | SIPA1L2 |
| 129 | ARHGAP4 |
| 130 | GBP4 |
| 131 | CNOT6 |
| 132 | GLRA1 |
| 133 | SEL1L3 |
| 134 | CNPY1 |
| 135 | ZNF384 |
| 136 | HOXC6 |
| 137 | LAMA5 |
| 138 | MX1 |
| 139 | ZNF800 |
| 140 | ZNF491 |
| 141 | C20orf3 |
| 142 | ZNF57 |
| 143 | CGN |
| 144 | ZNF503 |
| 145 | NOL6 |
| 146 | KIAA1456 |
| 147 | ZNF266 |
| 148 | MAB21L2 |
| 149 | RIIAD1 |
| 150 | ZNF187 |
| 151 | LBH |
| 152 | GTDC2 |
| 153 | COL6A1 |
| 154 | SETD8 |
| 155 | ALCAM |
| 156 | RANBP3L |
| 157 | CRIP3 |
| 158 | KRT18 |
| 159 | IFNAR1 |
| 160 | PLCXD3 |
| 161 | DHX38 |
| 162 | C10orf10 |
| 163 | LINC00176 |
| 164 | KRT24 |
| 165 | ZNF75D |
| 166 | CIB1 |
| 167 | PODXL |
| 168 | RALB |
| 169 | MED17 |
| 170 | OCIAD2 |
| 171 | ABR |
| 172 | CHST12 |
| 173 | CLK4 |
| 174 | GCOM1 |
| 175 | SLC22A23 |
| 176 | TPRA1 |
| 177 | UBASH3B |
| 178 | RNF115 |
| 179 | CCDC12 |
| 180 | STON1-GTF2A1L |
| 181 | EHF |
| 182 | GPSM3 |
| 183 | FLJ42875 |
| 184 | LOC439914 |
| 185 | NOL11 |
| 186 | USP45 |
| 187 | RBM5 |
| 188 | TLL2 |
| 189 | CCDC107 |
| 190 | FANCE |
| 191 | KCNF1 |
| 192 | OR13C5 |
| 193 | LCOR |
| 194 | DNAH7 |
| 195 | FAT2 |
| 196 | UTRN |
| 197 | NCAPG |
| 198 | TMSB10 |
| 199 | CCDC111 |
| 200 | DLX1 |
| 201 | APOL1 |
| 202 | PISD |
| 203 | NXF1 |
| 204 | ST7 |
| 205 | HOXA5 |
| 206 | PTPN13 |
| 207 | SNX1 |
| 208 | RSBN1 |
| 209 | GLCE |
| 210 | MYO1C |
| 211 | CYB5R3 |
| 212 | CHCHD10 |
| 213 | VAMP1 |
| 214 | LOC654433 |
| 215 | FAM160A2 |
| 216 | PACRGL |
| 217 | DLX6-AS1 |
| 218 | CBLB |
| 219 | UG0898H09 |
| 220 | EEF1DP3 |
| 221 | ERVV-1 |
| 222 | TRIM45 |
| 223 | KHDRBS2 |
| 224 | FGF20 |
| 225 | DDAH1 |
| 226 | POLB |
| 227 | KCNMB4 |
| 228 | WAC |
| 229 | TOP1MT |
| 230 | TSTD1 |
| 231 | EPC1 |
| 232 | MON1B |
| 233 | TMEM47 |
| 234 | SLC25A34 |
| 235 | ABLIM3 |
| 236 | SPP1 |
| 237 | SFI1 |
| 238 | ERCC3 |
| 239 | RPS6KA2 |
| 240 | AGPAT6 |
| 241 | FLRT2 |
| 242 | C16orf57 |
| 243 | LRFN4 |
| 244 | MGARP |
| 245 | SKOR1 |
| 246 | CLIP1 |
| 247 | CDC25B |
| 248 | KCNQ1OT1 |
| 249 | SUV420H2 |
| 250 | ZNF143 |
| 251 | SOX17 |
| 252 | ING3 |
| 253 | XAF1 |
| 254 | MGC16384 |
| 255 | TPRKB |
| 256 | WDR83 |
| 257 | FGF5 |
| 258 | RNF152 |
| 259 | NCOA1 |
| 260 | KCTD14 |
| 261 | ADAMTS19 |
| 262 | FAM131C |
| 263 | CRB1 |
| 264 | PPT2 |
| 265 | MAP3K3 |
| 266 | CCDC14 |
| 267 | FAM161B |
| 268 | HCFC2 |
| 269 | ZNF646 |
| 270 | EPB41L4A-AS1 |
| 271 | ZNF148 |
| 272 | CAMKV |
| 273 | NRG2 |
| 274 | MALT1 |
| 275 | KRT33B |
| 276 | HINFP |
| 277 | YKT6 |
| 278 | PDE6B |
| 279 | C4orf3 |
| 280 | FRMD6 |
| 281 | DENND4C |
| 282 | ZFAND2B |
| 283 | CA9 |
| 284 | PLA2R1 |
| 285 | AMT |
| 286 | CDR2L |
| 287 | NRK |
| 288 | PRB3 |
| 289 | ZNF442 |
| 290 | SOD1 |
| 291 | SDHAF1 |
| 292 | USP44 |
| 293 | ADRM1 |
| 294 | LOC100288147 |
| 295 | HRASLS |
| 296 | LOC100129201 |
| 297 | AMY2A |
| 298 | KIF17 |
| 299 | RNASEH2C |
| 300 | TPCN2 |
| 301 | NPR2 |
| 302 | PIGH |
| 303 | KRT18P26 |
| 304 | TRIM11 |
| 305 | RSPO4 |
| 306 | HCN2 |
| 307 | FHOD3 |
| 308 | FTH1P20 |
| 309 | FOXA2 |
| 310 | ST13 |
| 311 | PRPSAP2 |
| 312 | EPB41L5 |
| 313 | BEND7 |
| 314 | PDLIM2 |
| 315 | TH1L |
| 316 | PCDHA1 |
| 317 | NXPH4 |
| 318 | NEFH |
| 319 | KLF6 |
| 320 | SCARA3 |
| 321 | PILRA |
| 322 | RNFT2 |
| 323 | C2CD4A |
| 324 | ZNF134 |
| 325 | ARID2 |
| 326 | PAXIP1 |
| 327 | EPB41L1 |
| 328 | SORBS3 |
| 329 | SLC35F4 |
| 330 | CLPP |
| 331 | BEST3 |
| 332 | SYNE4 |
| 333 | FBXO5 |
| 334 | FBXL6 |
| 335 | CHD2 |
| 336 | RGS5 |
| 337 | CST6 |
| 338 | CCT7 |
| 339 | SOCS5 |
| 340 | HSPB8 |
| 341 | FLJ10661 |
| 342 | CCDC152 |
| 343 | GFRA3 |
| 344 | DKK3 |
| 345 | TPPP |
| 346 | TNFRSF6B |
| 347 | CRAMP1L |
| 348 | MIOS |
| 349 | PGM5 |
| 350 | SLC16A5 |
| 351 | CPLX4 |
| 352 | CPT1A |
| 353 | FLT3 |
| 354 | TRIM72 |
| 355 | GPR12 |
| 356 | LOC220729 |
| 357 | FLJ43663 |
| 358 | LOC100129917 |
| 359 | CCNG2 |
| 360 | ARHGAP22 |
| 361 | HPS4 |
| 362 | CREG1 |
| 363 | ENY2 |
| 364 | ZNF335 |
| 365 | CAB39L |
| 366 | SNHG8 |
| 367 | RASAL1 |
| 368 | FREM1 |
| 369 | RNF112 |
| 370 | TPGS1 |
| 371 | BAZ2A |
| 372 | C2orf15 |
| 373 | SLC26A5 |
| 374 | PRMT1 |
| 375 | CARNS1 |
| 376 | SELO |
| 377 | GPATCH8 |
| 378 | NIPA1 |
| 379 | LOC442249 |
| 380 | TBC1D20 |
| 381 | TRIM14 |
| 382 | MAMDC4 |
| 383 | SLMO1 |
| 384 | ZNF273 |
| 385 | GUK1 |
| 386 | C5orf46 |
| 387 | MPG |
| 388 | CDH8 |
| 389 | RSAD1 |
| 390 | MINA |
| 391 | RELN |
| 392 | GABRA6 |
| 393 | ANAPC13 |
| 394 | ARMC8 |
| 395 | MORN4 |
| 396 | TRIM68 |
| 397 | SNRK |
| 398 | UNCX |
| 399 | ALG1L |
| 400 | RASAL2 |
| 401 | MAP3K5 |
| 402 | CLK2 |
| 403 | FAM63B |
| 404 | FGF17 |
| 405 | ACLY |
| 406 | CLCN5 |
| 407 | KCTD2 |
| 408 | ITGA2 |
| 409 | TMEM191A |
| 410 | ZNF544 |
| 411 | DPH2 |
| 412 | ZNF593 |
| 413 | FAM213A |
| 414 | GPCPD1 |
| 415 | NGRN |
| 416 | ETV1 |
| 417 | ABHD14A |
| 418 | PTK2B |
| 419 | DPF3 |
| 420 | DDX46 |
| 421 | LTBP4 |
| 422 | DUSP12 |
| 423 | NYNRIN |
| 424 | ANKRD10 |
| 425 | UBTD2 |
| 426 | TSEN54 |
| 427 | KCNS3 |
| 428 | SNRNP200 |
| 429 | GRID2 |
| 430 | LPPR5 |
| 431 | SRM |
| 432 | RAB3IP |
| 433 | HFM1 |
| 434 | SCARNA17 |
| 435 | RBP4 |
| 436 | CYP2W1 |
| 437 | MTHFD1L |
| 438 | ZNF337 |
| 439 | TRIM67 |
| 440 | ABLIM2 |
| 441 | HOXB4 |
| 442 | LIMA1 |
| 443 | EIF3L |
| 444 | CXorf24 |
| 445 | C11orf31 |
| 446 | MICU1 |
| 447 | RMND5A |
| 448 | JARID2 |
| 449 | ASTN1 |
| 450 | SPATA7 |
| 451 | MIIP |
| 452 | PPP1CC |
| 453 | PHF11 |
| 454 | TMEFF2 |
| 455 | NHEJ1 |
| 456 | DYSF |
| 457 | USP1 |
| 458 | NOP16 |
| 459 | PCDHA8 |
| 460 | AR |
| 461 | CDK8 |
| 462 | CEP76 |
| 463 | ANKRD18B |
| 464 | ADAM15 |
| 465 | GPR89B |
| 466 | TIMP4 |
| 467 | C5orf54 |
| 468 | ZNF600 |
| 469 | TRIM50 |
| 470 | CDK5R2 |
| 471 | SLC25A27 |
| 472 | ZKSCAN1 |
| 473 | LINC00115 |
| 474 | STAC |
| 475 | ARX |
| 476 | PRELID2 |
| 477 | RUFY2 |
| 478 | ARHGEF10L |
| 479 | C5orf58 |
| 480 | KRT34 |
| 481 | PLD1 |
| 482 | LOC441728 |
| 483 | LOC100127899 |
| 484 | CHD4 |
| 485 | PARP16 |
| 486 | NUP98 |
| 487 | SLC1A6 |
| 488 | DCUN1D2 |
| 489 | MBTPS2 |
| 490 | LEMD1 |
| 491 | DUSP5 |
| 492 | SLC9A3R2 |
| 493 | SGCA |
| 494 | DSCC1 |
| 495 | LOC147727 |
| 496 | RABEPK |
| 497 | SPEF1 |
| 498 | SYTL1 |
| 499 | DGKD |
| 500 | LONRF1 |

1. **H0351.1012**

**(I) MaxRel feature list**

| Order | Name |
| --- | --- |
| 1 | DAO |
| 2 | NR2E1 |
| 3 | LRRC7 |
| 4 | FLJ42875 |
| 5 | PDE2A |
| 6 | GABRA5 |
| 7 | STON1 |
| 8 | MEIS1 |
| 9 | PAX3 |
| 10 | NRGN |
| 11 | CCNG2 |
| 12 | IRX2 |
| 13 | RASL10A |
| 14 | CLK4 |
| 15 | CTDSPL |
| 16 | ARHGAP4 |
| 17 | MX1 |
| 18 | KHDRBS2 |
| 19 | SYNE4 |
| 20 | RCAN3 |
| 21 | CCK |
| 22 | FLRT2 |
| 23 | CREG2 |
| 24 | ABR |
| 25 | DUSP5 |
| 26 | CAMK4 |
| 27 | ACTN1 |
| 28 | LHX2 |
| 29 | SYT5 |
| 30 | LDB2 |
| 31 | ARHGAP24 |
| 32 | DDN |
| 33 | BCAT1 |
| 34 | CBLN1 |
| 35 | SRGAP2 |
| 36 | CAMKV |
| 37 | SEL1L3 |
| 38 | CRB1 |
| 39 | VAMP1 |
| 40 | ETV1 |
| 41 | ZNF521 |
| 42 | NECAB1 |
| 43 | CHD7 |
| 44 | MPP4 |
| 45 | LHX6 |
| 46 | LOC100131342 |
| 47 | GLT8D2 |
| 48 | CPNE4 |
| 49 | HOXB2 |
| 50 | TAC3 |
| 51 | GAS2 |
| 52 | KIAA1024 |
| 53 | CAMKK2 |
| 54 | LINC00473 |
| 55 | NEUROD1 |
| 56 | PLK2 |
| 57 | ITPKA |
| 58 | TIMP4 |
| 59 | NRG3 |
| 60 | LBH |
| 61 | EXPH5 |
| 62 | ICAM5 |
| 63 | KIF17 |
| 64 | FOXA2 |
| 65 | TRIM58 |
| 66 | LPCAT2 |
| 67 | SIPA1L2 |
| 68 | UG0898H09 |
| 69 | RNF152 |
| 70 | CDH9 |
| 71 | FAM81A |
| 72 | STOX1 |
| 73 | BARHL1 |
| 74 | EMX2 |
| 75 | NPTX2 |
| 76 | TIMP2 |
| 77 | STK10 |
| 78 | FAT2 |
| 79 | MXD4 |
| 80 | EGR3 |
| 81 | CXCL14 |
| 82 | FAM49A |
| 83 | DLGAP2 |
| 84 | BTN2A2 |
| 85 | COL13A1 |
| 86 | ZNF564 |
| 87 | KALRN |
| 88 | RBFOX3 |
| 89 | CTNNA2 |
| 90 | VPS45 |
| 91 | EPS8L2 |
| 92 | RGS4 |
| 93 | PADI2 |
| 94 | GDA |
| 95 | ABRACL |
| 96 | FGFR1 |
| 97 | PRORSD1P |
| 98 | TMEM132B |
| 99 | PODXL |
| 100 | DGAT2 |
| 101 | KCNF1 |
| 102 | WIPF1 |
| 103 | RANBP3L |
| 104 | AK5 |
| 105 | EMC10 |
| 106 | FAM5B |
| 107 | PTK2B |
| 108 | RMND5A |
| 109 | LHX1 |
| 110 | LDLRAP1 |
| 111 | LZTS1 |
| 112 | C2orf55 |
| 113 | VSIG8 |
| 114 | PLCB1 |
| 115 | MMD |
| 116 | DDX26B |
| 117 | HOXD1 |
| 118 | MKL2 |
| 119 | EXTL1 |
| 120 | SLCO1C1 |
| 121 | STX1A |
| 122 | ENC1 |
| 123 | C15orf27 |
| 124 | MMP17 |
| 125 | SYT16 |
| 126 | VIT |
| 127 | NEUROD6 |
| 128 | FLT3 |
| 129 | STBD1 |
| 130 | OTX1 |
| 131 | DLX1 |
| 132 | INADL |
| 133 | ITM2C |
| 134 | AKAP5 |
| 135 | CHRM3 |
| 136 | CAMK2A |
| 137 | CAMK2N1 |
| 138 | CMTM7 |
| 139 | PKIB |
| 140 | LINC00176 |
| 141 | PCDH20 |
| 142 | SGK223 |
| 143 | VWA5A |
| 144 | RRAGD |
| 145 | KRT18 |
| 146 | GNAL |
| 147 | DGKB |
| 148 | TFAP2B |
| 149 | RNFT2 |
| 150 | ZFHX3 |
| 151 | NKAIN3 |
| 152 | LOC283174 |
| 153 | SNHG8 |
| 154 | GRIN2B |
| 155 | SNHG10 |
| 156 | CBLB |
| 157 | RAB26 |
| 158 | RSPO4 |
| 159 | LOC100288147 |
| 160 | LNX1 |
| 161 | HOMER1 |
| 162 | SH2D5 |
| 163 | MICAL2 |
| 164 | GABRA4 |
| 165 | RASAL1 |
| 166 | NETO1 |
| 167 | SNX32 |
| 168 | FSTL5 |
| 169 | CYB5R3 |
| 170 | PLCB4 |
| 171 | PAX6 |
| 172 | B9D1 |
| 173 | OSBPL3 |
| 174 | CADPS2 |
| 175 | RBMS3 |
| 176 | EPHA4 |
| 177 | NUAK2 |
| 178 | EIF5A2 |
| 179 | UHRF2 |
| 180 | PARP16 |
| 181 | TTC39A |
| 182 | XAF1 |
| 183 | SYNJ2 |
| 184 | ZIC4 |
| 185 | CACNB4 |
| 186 | RPS6KA1 |
| 187 | SLC26A10 |
| 188 | OLR1 |
| 189 | ZNF219 |
| 190 | SHC3 |
| 191 | RPRML |
| 192 | CDH23 |
| 193 | CTDSPL2 |
| 194 | TMEM44 |
| 195 | TMEM61 |
| 196 | NCALD |
| 197 | WSCD2 |
| 198 | PLA2R1 |
| 199 | IRX3 |
| 200 | RHOQ |
| 201 | CHRD |
| 202 | NECAB2 |
| 203 | PDE1A |
| 204 | MPP3 |
| 205 | FOXG1 |
| 206 | RGS20 |
| 207 | CX3CL1 |
| 208 | RASGRF2 |
| 209 | LINC00599 |
| 210 | PALM2-AKAP2 |
| 211 | PRSS55 |
| 212 | CA4 |
| 213 | EBF1 |
| 214 | CLIP1 |
| 215 | ZNF800 |
| 216 | MOXD1 |
| 217 | KCNJ2 |
| 218 | NEFM |
| 219 | SLC16A5 |
| 220 | LOC729683 |
| 221 | MAST3 |
| 222 | C11orf58 |
| 223 | NEFL |
| 224 | SERPINI1 |
| 225 | DCAF6 |
| 226 | OR14I1 |
| 227 | H2BFXP |
| 228 | EHD1 |
| 229 | ATRNL1 |
| 230 | LOC100287347 |
| 231 | ZNF77 |
| 232 | GALNT12 |
| 233 | BACE2 |
| 234 | RAB11B |
| 235 | SYT2 |
| 236 | SLC1A6 |
| 237 | KCNV1 |
| 238 | DNAH7 |
| 239 | DDX46 |
| 240 | ATP2B1 |
| 241 | RGS14 |
| 242 | CGN |
| 243 | DLX6-AS1 |
| 244 | C8orf46 |
| 245 | CABP1 |
| 246 | PTPN5 |
| 247 | LOC158696 |
| 248 | USP45 |
| 249 | PPP2R2D |
| 250 | RAPGEF4 |
| 251 | PCP2 |
| 252 | FAM153A |
| 253 | PVRL3 |
| 254 | DYSF |
| 255 | CDC7 |
| 256 | RBM24 |
| 257 | NOL11 |
| 258 | EPB41L5 |
| 259 | HOOK1 |
| 260 | C14orf23 |
| 261 | UTRN |
| 262 | NDP |
| 263 | HPCA |
| 264 | CHSY3 |
| 265 | FHL2 |
| 266 | FAM84A |
| 267 | C17orf108 |
| 268 | SLC8A2 |
| 269 | FEZF2 |
| 270 | HTR2A |
| 271 | DLX2 |
| 272 | EIF3L |
| 273 | SCARA3 |
| 274 | TMEM241 |
| 275 | C11orf80 |
| 276 | SPSB1 |
| 277 | NGEF |
| 278 | PRDM16 |
| 279 | PPFIA2 |
| 280 | USP1 |
| 281 | LOC100287987 |
| 282 | SFRP1 |
| 283 | GALNT9 |
| 284 | SNHG7 |
| 285 | LAMB1 |
| 286 | FAM171B |
| 287 | ANK1 |
| 288 | LOC100131289 |
| 289 | LRRC16A |
| 290 | EIF3E |
| 291 | PTPRR |
| 292 | ENGASE |
| 293 | MBP |
| 294 | PCSK9 |
| 295 | PCDHA8 |
| 296 | HS6ST3 |
| 297 | ALCAM |
| 298 | CACNG3 |
| 299 | LOC151009 |
| 300 | THRB |
| 301 | ARSJ |
| 302 | MATK |
| 303 | CLCNKB |
| 304 | HMG20B |
| 305 | GRIA2 |
| 306 | RPL3P2 |
| 307 | B3GALT2 |
| 308 | RHOBTB2 |
| 309 | LOC643037 |
| 310 | GRASP |
| 311 | GAB2 |
| 312 | WIF1 |
| 313 | FILIP1 |
| 314 | CLK2 |
| 315 | PDE8B |
| 316 | CORT |
| 317 | CAMK1G |
| 318 | ZNF187 |
| 319 | SPON2 |
| 320 | DUSP22 |
| 321 | COL6A1 |
| 322 | PRSS35 |
| 323 | NLK |
| 324 | SLIT1 |
| 325 | LIMA1 |
| 326 | MYLK |
| 327 | WASF1 |
| 328 | PRICKLE2 |
| 329 | EDIL3 |
| 330 | C16orf11 |
| 331 | GABRA6 |
| 332 | IL13RA1 |
| 333 | LMO7 |
| 334 | CHST1 |
| 335 | RNF112 |
| 336 | LPPR4 |
| 337 | FAM190A |
| 338 | PMEPA1 |
| 339 | RBP4 |
| 340 | NPAS2 |
| 341 | PSD3 |
| 342 | LINS |
| 343 | ZDHHC23 |
| 344 | MCTP1 |
| 345 | NELF |
| 346 | KCNB1 |
| 347 | C2orf80 |
| 348 | ZDHHC2 |
| 349 | LDLRAD3 |
| 350 | SHPRH |
| 351 | TIAM1 |
| 352 | LRRTM4 |
| 353 | ANLN |
| 354 | TRIM48 |
| 355 | CAST |
| 356 | SPTLC2 |
| 357 | SERPINF1 |
| 358 | LRRC8B |
| 359 | AVIL |
| 360 | NIM1 |
| 361 | LGALS1 |
| 362 | CTXN1 |
| 363 | SCN3B |
| 364 | MCF2 |
| 365 | ZNF238 |
| 366 | FAM131C |
| 367 | ATP4A |
| 368 | TRIM9 |
| 369 | LACC1 |
| 370 | NUP85 |
| 371 | BAI2 |
| 372 | EMX2OS |
| 373 | KIAA1456 |
| 374 | CLDN3 |
| 375 | BCL6 |
| 376 | KCTD16 |
| 377 | GRM5 |
| 378 | WWOX |
| 379 | SEMA3C |
| 380 | NPNT |
| 381 | TNS1 |
| 382 | DIO2 |
| 383 | ARHGAP32 |
| 384 | AGBL4 |
| 385 | CHN1 |
| 386 | PLGLB1 |
| 387 | 10-Sep |
| 388 | C1orf198 |
| 389 | C1orf115 |
| 390 | FAM125B |
| 391 | PIRT |
| 392 | ARHGEF4 |
| 393 | CCDC3 |
| 394 | LOC646627 |
| 395 | HSPB8 |
| 396 | PCDHA11 |
| 397 | EPHB6 |
| 398 | ADAMTS19 |
| 399 | UBASH3B |
| 400 | CDKL5 |
| 401 | CRNDE |
| 402 | RELN |
| 403 | OR7E36P |
| 404 | RNF148 |
| 405 | PCLO |
| 406 | PRSS22 |
| 407 | FAP |
| 408 | STYK1 |
| 409 | SOX11 |
| 410 | SYDE2 |
| 411 | EMX1 |
| 412 | DACT1 |
| 413 | SLC22A23 |
| 414 | SAMD4A |
| 415 | GRM4 |
| 416 | MAB21L1 |
| 417 | P2RX4 |
| 418 | ALS2 |
| 419 | PRPS2 |
| 420 | PTPRO |
| 421 | LRRC4C |
| 422 | C18orf42 |
| 423 | SRP14P1 |
| 424 | GPR26 |
| 425 | LPPR5 |
| 426 | SLC35F4 |
| 427 | LRRC73 |
| 428 | APBB2 |
| 429 | GLTP |
| 430 | LOC439914 |
| 431 | HINFP |
| 432 | ARHGEF10L |
| 433 | BEND7 |
| 434 | ODZ2 |
| 435 | COQ10A |
| 436 | TRIM11 |
| 437 | PCDH8 |
| 438 | KANK4 |
| 439 | SPP1 |
| 440 | FBXL16 |
| 441 | NCAN |
| 442 | RIMBP2 |
| 443 | KLK6 |
| 444 | AGPAT9 |
| 445 | C1orf194 |
| 446 | NYNRIN |
| 447 | ANKRD42 |
| 448 | TXNRD2 |
| 449 | LOC150622 |
| 450 | GRIK2 |
| 451 | C17orf110 |
| 452 | MIR7-3HG |
| 453 | CHADL |
| 454 | SOX8 |
| 455 | ICA1L |
| 456 | GABRB2 |
| 457 | DHRS13 |
| 458 | C1orf38 |
| 459 | STON1-GTF2A1L |
| 460 | THOC3 |
| 461 | ANKRD29 |
| 462 | ZBBX |
| 463 | ZNF611 |
| 464 | RPL18 |
| 465 | ZEB2 |
| 466 | CDH18 |
| 467 | MPPED1 |
| 468 | MTPAP |
| 469 | KCNJ4 |
| 470 | PDYN |
| 471 | ACHE |
| 472 | TMEM249 |
| 473 | NUP54 |
| 474 | UNCX |
| 475 | FKBP1A |
| 476 | TLE2 |
| 477 | HRK |
| 478 | PHACTR1 |
| 479 | UGT8 |
| 480 | RFFL |
| 481 | NEK10 |
| 482 | KCNE4 |
| 483 | ARNTL |
| 484 | RIN1 |
| 485 | CNPY1 |
| 486 | CEP76 |
| 487 | SLC1A2 |
| 488 | KCNMA1 |
| 489 | MGARP |
| 490 | ARHGEF40 |
| 491 | CCDC111 |
| 492 | RORB |
| 493 | JPH1 |
| 494 | HOXA3 |
| 495 | CSGALNACT1 |
| 496 | C3orf80 |
| 497 | MIRLET7BHG |
| 498 | STEAP3 |
| 499 | GLCE |
| 500 | SOX2-OT |

**(II) mRMR feature list**

| Order | Name |
| --- | --- |
| 1 | DAO |
| 2 | HOXA2 |
| 3 | PCP2 |
| 4 | NR2E1 |
| 5 | ARHGAP4 |
| 6 | HOXB2 |
| 7 | TFAP2B |
| 8 | PAX3 |
| 9 | STON1 |
| 10 | CLK4 |
| 11 | MEIS1 |
| 12 | HOXA4 |
| 13 | NIM1 |
| 14 | STK10 |
| 15 | NKAIN3 |
| 16 | EMX2 |
| 17 | HOXB3 |
| 18 | CNPY1 |
| 19 | SRGAP2 |
| 20 | CYB5R3 |
| 21 | STOX1 |
| 22 | ZNF521 |
| 23 | HOXA3 |
| 24 | TIMP4 |
| 25 | NECAB1 |
| 26 | LOC100131342 |
| 27 | HOXB5 |
| 28 | GABRA5 |
| 29 | OTX1 |
| 30 | ZNF77 |
| 31 | RANBP3L |
| 32 | SCARA3 |
| 33 | LBH |
| 34 | VAMP1 |
| 35 | CLIP1 |
| 36 | SAMD4A |
| 37 | STON1-GTF2A1L |
| 38 | FAT2 |
| 39 | GLRA1 |
| 40 | C11orf58 |
| 41 | ACTN1 |
| 42 | RNFT2 |
| 43 | VSIG8 |
| 44 | RASL10A |
| 45 | HOXD3 |
| 46 | VPS45 |
| 47 | COL6A1 |
| 48 | LHX1 |
| 49 | UG0898H09 |
| 50 | HOOK1 |
| 51 | DDX26B |
| 52 | FLJ42875 |
| 53 | PAX8 |
| 54 | DUSP5 |
| 55 | EIF3E |
| 56 | LRRC7 |
| 57 | CCDC104 |
| 58 | PODXL |
| 59 | PAX2 |
| 60 | ZNF219 |
| 61 | MBIP |
| 62 | ABR |
| 63 | PRORSD1P |
| 64 | KRT18 |
| 65 | SIGIRR |
| 66 | SKOR1 |
| 67 | RAPGEF4 |
| 68 | NKD2 |
| 69 | GLT8D2 |
| 70 | UTRN |
| 71 | EMX2OS |
| 72 | PRDM16 |
| 73 | ZFY |
| 74 | HOXD1 |
| 75 | IRX2 |
| 76 | ITM2C |
| 77 | SNHG8 |
| 78 | NTN1 |
| 79 | ETV2 |
| 80 | PPM1B |
| 81 | SYT2 |
| 82 | NCAN |
| 83 | RAB11B |
| 84 | SLCO1C1 |
| 85 | ZNF57 |
| 86 | TMEM44 |
| 87 | LOC100288147 |
| 88 | PLBD2 |
| 89 | NPTX2 |
| 90 | MSX2 |
| 91 | ETV1 |
| 92 | GPR89B |
| 93 | MPP4 |
| 94 | ANK1 |
| 95 | HOXA7 |
| 96 | CRB1 |
| 97 | USP1 |
| 98 | GABRA4 |
| 99 | LOXL2 |
| 100 | MX1 |
| 101 | HNRNPH1 |
| 102 | FAM125B |
| 103 | LTA4H |
| 104 | LINS |
| 105 | DKFZp779M0652 |
| 106 | SIPA1L2 |
| 107 | SLC16A5 |
| 108 | KLK8 |
| 109 | LOC400043 |
| 110 | KALRN |
| 111 | PRR5 |
| 112 | TRIM58 |
| 113 | CANX |
| 114 | PCSK9 |
| 115 | MTPAP |
| 116 | EPPK1 |
| 117 | SLC45A3 |
| 118 | KCNF1 |
| 119 | ROR1 |
| 120 | PLA2R1 |
| 121 | HOXA6 |
| 122 | NOL11 |
| 123 | PDLIM2 |
| 124 | CTNNA2 |
| 125 | PALM2-AKAP2 |
| 126 | FAM171B |
| 127 | LGALS3 |
| 128 | CHD7 |
| 129 | TMC2 |
| 130 | CBLN1 |
| 131 | ERRFI1 |
| 132 | FAM5B |
| 133 | GABRA6 |
| 134 | DLX1 |
| 135 | ABLIM1 |
| 136 | LDLRAD3 |
| 137 | HOXB4 |
| 138 | STBD1 |
| 139 | ZC3H12A |
| 140 | BARHL1 |
| 141 | KCNE4 |
| 142 | FAM213B |
| 143 | GAB2 |
| 144 | EPHA4 |
| 145 | CCDC111 |
| 146 | GAS2 |
| 147 | CAMKV |
| 148 | RRAGD |
| 149 | TDRD6 |
| 150 | NPNT |
| 151 | C11orf80 |
| 152 | KCNS3 |
| 153 | NRG3 |
| 154 | EMC10 |
| 155 | FKBP14 |
| 156 | NPAS2 |
| 157 | MAB21L1 |
| 158 | SWAP70 |
| 159 | GABRB2 |
| 160 | EED |
| 161 | KRT18P55 |
| 162 | CCNG2 |
| 163 | B4GALT2 |
| 164 | ZNRF3 |
| 165 | RPS6KA1 |
| 166 | FLRT2 |
| 167 | GPRC5C |
| 168 | FBXO4 |
| 169 | PPFIA2 |
| 170 | PADI2 |
| 171 | SHPRH |
| 172 | FKBP1A |
| 173 | CCDC101 |
| 174 | LOC100133008 |
| 175 | ZC3H18 |
| 176 | KHDRBS2 |
| 177 | NXPH4 |
| 178 | EIF5A2 |
| 179 | FAM49A |
| 180 | FAM57A |
| 181 | DZANK1 |
| 182 | CX3CL1 |
| 183 | RMND5A |
| 184 | NECAB2 |
| 185 | NIN |
| 186 | KIAA1024 |
| 187 | FKBP9 |
| 188 | UBASH3B |
| 189 | PARP16 |
| 190 | C1orf38 |
| 191 | LOC100288144 |
| 192 | EHD1 |
| 193 | SYF2 |
| 194 | BCAT1 |
| 195 | SERHL2 |
| 196 | TFAP2A |
| 197 | C17orf89 |
| 198 | SYT5 |
| 199 | BEND3 |
| 200 | IRX3 |
| 201 | CACNB4 |
| 202 | HOXA5 |
| 203 | ENGASE |
| 204 | ABLIM3 |
| 205 | B9D1 |
| 206 | C1orf150 |
| 207 | PRIMA1 |
| 208 | C17orf70 |
| 209 | PDE2A |
| 210 | EPB41L5 |
| 211 | CDC7 |
| 212 | FLJ41350 |
| 213 | MYO1A |
| 214 | CTDSPL2 |
| 215 | CDKL5 |
| 216 | LOC100132317 |
| 217 | CTDSPL |
| 218 | CHADL |
| 219 | IL1RAP |
| 220 | CSK |
| 221 | SGK223 |
| 222 | SLC25A27 |
| 223 | PRSS35 |
| 224 | IQCC |
| 225 | MMP17 |
| 226 | NUP54 |
| 227 | AGPAT4 |
| 228 | EBF1 |
| 229 | SNHG10 |
| 230 | FAM160B2 |
| 231 | GPR56 |
| 232 | MON2 |
| 233 | SNHG7 |
| 234 | NUAK2 |
| 235 | VSIG4 |
| 236 | CBLN3 |
| 237 | SIRPA |
| 238 | TMEM61 |
| 239 | ANGPT1 |
| 240 | MLLT4-AS1 |
| 241 | ZNF337 |
| 242 | WIPF1 |
| 243 | ZNF28 |
| 244 | FAM131C |
| 245 | MPP3 |
| 246 | NELF |
| 247 | ZIK1 |
| 248 | CASP7 |
| 249 | ANKS1B |
| 250 | C1orf51 |
| 251 | HMG20B |
| 252 | ZNF556 |
| 253 | SFRP1 |
| 254 | PPP2R2D |
| 255 | CD99L2 |
| 256 | LDB2 |
| 257 | RPL18 |
| 258 | COL8A2 |
| 259 | SLC22A13 |
| 260 | CGN |
| 261 | PCDHA11 |
| 262 | CALCRL |
| 263 | INADL |
| 264 | CBLB |
| 265 | SOGA1 |
| 266 | FAM70A |
| 267 | SPSB1 |
| 268 | YY2 |
| 269 | RASAL1 |
| 270 | UHRF2 |
| 271 | ARX |
| 272 | FBXO5 |
| 273 | CXorf24 |
| 274 | ARHGEF40 |
| 275 | NKX6-1 |
| 276 | VWA5A |
| 277 | YEATS4 |
| 278 | C14orf23 |
| 279 | SOD1 |
| 280 | POLD2 |
| 281 | SMC5 |
| 282 | RBM24 |
| 283 | EMILIN2 |
| 284 | NACA |
| 285 | FAT3 |
| 286 | LINC00312 |
| 287 | ATP2B1 |
| 288 | SPINK6 |
| 289 | ACTC1 |
| 290 | KLHL23 |
| 291 | PTRH1 |
| 292 | MOXD1 |
| 293 | LPCAT2 |
| 294 | C14orf105 |
| 295 | RELN |
| 296 | PRPS2 |
| 297 | C16orf7 |
| 298 | SEL1L3 |
| 299 | RELL1 |
| 300 | DENND4C |
| 301 | NYNRIN |
| 302 | RIIAD1 |
| 303 | SYNE4 |
| 304 | KIN |
| 305 | SLC2A13 |
| 306 | SRP14P1 |
| 307 | GALNT7 |
| 308 | SNX32 |
| 309 | LINC00473 |
| 310 | MEX3D |
| 311 | PPP1R3D |
| 312 | PTPN2 |
| 313 | LIMA1 |
| 314 | ZNF23 |
| 315 | RUNX1T1 |
| 316 | NTRK2 |
| 317 | ELP4 |
| 318 | LINC00162 |
| 319 | FGFR1 |
| 320 | CDKN1B |
| 321 | EPB41L1 |
| 322 | DLX2 |
| 323 | SRGAP2C |
| 324 | LEMD1 |
| 325 | MMP28 |
| 326 | KCNMB4 |
| 327 | CNOT6 |
| 328 | KCNJ2 |
| 329 | TIAM1 |
| 330 | RARB |
| 331 | KANSL2 |
| 332 | DUSP22 |
| 333 | USP11 |
| 334 | DCAF6 |
| 335 | CLCNKB |
| 336 | NGFR |
| 337 | ZNF187 |
| 338 | KCNB1 |
| 339 | HCFC2 |
| 340 | MBOAT1 |
| 341 | SPATA5L1 |
| 342 | CDH9 |
| 343 | ADCY7 |
| 344 | FASTKD3 |
| 345 | TRIM32 |
| 346 | MYBL1 |
| 347 | CCDC155 |
| 348 | TRIM9 |
| 349 | TP53BP2 |
| 350 | ARHGEF10L |
| 351 | CAMKK2 |
| 352 | THUMPD2 |
| 353 | HABP2 |
| 354 | SLC1A2 |
| 355 | CDH23 |
| 356 | MBD4 |
| 357 | IPW |
| 358 | PCDHA8 |
| 359 | SOX8 |
| 360 | LOC100131699 |
| 361 | BTN2A2 |
| 362 | IGSF22 |
| 363 | ZNF782 |
| 364 | APLN |
| 365 | DNAH7 |
| 366 | RSPO4 |
| 367 | IMMP1L |
| 368 | PTK2B |
| 369 | UROS |
| 370 | TACC1 |
| 371 | EBF3 |
| 372 | SETD8 |
| 373 | CD83 |
| 374 | FOXA2 |
| 375 | TRIM55 |
| 376 | EPS8L2 |
| 377 | HOXC6 |
| 378 | POU2F1 |
| 379 | THRB |
| 380 | MICALCL |
| 381 | ZNF330 |
| 382 | GPD1 |
| 383 | CA9 |
| 384 | PLCB1 |
| 385 | KANK4 |
| 386 | C16orf57 |
| 387 | LOC100127899 |
| 388 | PDE8B |
| 389 | TRIM14 |
| 390 | C2orf77 |
| 391 | ANKRD29 |
| 392 | ACSBG2 |
| 393 | EIF3L |
| 394 | OR2A9P |
| 395 | ZNF611 |
| 396 | FOXG1 |
| 397 | KIAA0247 |
| 398 | MBP |
| 399 | RIN1 |
| 400 | KRT18P26 |
| 401 | SLC22A31 |
| 402 | MYEF2 |
| 403 | DLGAP2 |
| 404 | ADAM9 |
| 405 | PPFIBP1 |
| 406 | MTPN |
| 407 | NEFM |
| 408 | MVK |
| 409 | RHPN1 |
| 410 | NCALD |
| 411 | ZNF792 |
| 412 | WWOX |
| 413 | FAM190A |
| 414 | QRSL1 |
| 415 | LZTS1 |
| 416 | ZNF669 |
| 417 | SPTLC2 |
| 418 | RNF133 |
| 419 | APOL1 |
| 420 | FSTL5 |
| 421 | ANGPTL2 |
| 422 | KCNK9 |
| 423 | PIF1 |
| 424 | ZNF439 |
| 425 | OR14I1 |
| 426 | MYLK |
| 427 | OVOS |
| 428 | TNFRSF12A |
| 429 | TRIM67 |
| 430 | GRM4 |
| 431 | MRPS30 |
| 432 | LPPR4 |
| 433 | ARHGAP24 |
| 434 | SMCP |
| 435 | LRRC39 |
| 436 | GALNT12 |
| 437 | CNOT2 |
| 438 | SLC6A5 |
| 439 | OLR1 |
| 440 | ZNF503 |
| 441 | CAMK4 |
| 442 | KBTBD10 |
| 443 | CYP46A1 |
| 444 | RNF115 |
| 445 | ICA1L |
| 446 | C1orf194 |
| 447 | GCOM1 |
| 448 | CERK |
| 449 | ZNF671 |
| 450 | P2RX4 |
| 451 | C17orf110 |
| 452 | EHF |
| 453 | ING3 |
| 454 | S100A6 |
| 455 | PRSS55 |
| 456 | ZDHHC2 |
| 457 | HOXD-AS1 |
| 458 | IL28RA |
| 459 | CMTM7 |
| 460 | PRPF38B |
| 461 | NRGN |
| 462 | CCT7 |
| 463 | MCM5 |
| 464 | OSBPL3 |
| 465 | MFNG |
| 466 | SLC26A11 |
| 467 | CXCL14 |
| 468 | CNOT6L |
| 469 | FAM101A |
| 470 | LDLRAP1 |
| 471 | MTBP |
| 472 | ANO10 |
| 473 | GGTA1P |
| 474 | MINA |
| 475 | ZNF564 |
| 476 | SORBS3 |
| 477 | ELFN2 |
| 478 | SWSAP1 |
| 479 | BACE2 |
| 480 | LOC646043 |
| 481 | AKAP5 |
| 482 | NRK |
| 483 | PALM3 |
| 484 | USP44 |
| 485 | RNF152 |
| 486 | MSR1 |
| 487 | RPS21 |
| 488 | ZNF256 |
| 489 | KLK6 |
| 490 | DMWD |
| 491 | AVIL |
| 492 | DLX6-AS1 |
| 493 | LPCAT3 |
| 494 | PHOX2B |
| 495 | PDZK1P1 |
| 496 | LOC727938 |
| 497 | TMEM229B |
| 498 | KBTBD8 |
| 499 | PLCB4 |
| 500 | KDM4C |

1. **H0351.1015**

**(I) MaxRel feature list**

| Order | Name |
| --- | --- |
| 1 | LRRC7 |
| 2 | DAO |
| 3 | ZNF521 |
| 4 | GABRA5 |
| 5 | ABLIM1 |
| 6 | ARHGEF6 |
| 7 | RASAL1 |
| 8 | NECAB1 |
| 9 | DLX6-AS1 |
| 10 | TIMP4 |
| 11 | CCNG2 |
| 12 | ITPKA |
| 13 | LDB2 |
| 14 | PDE2A |
| 15 | CAMK4 |
| 16 | STON1 |
| 17 | IRX2 |
| 18 | LINC00473 |
| 19 | PLK2 |
| 20 | CREG2 |
| 21 | AK5 |
| 22 | FAM81A |
| 23 | GPRC5C |
| 24 | NRGN |
| 25 | DDN |
| 26 | CHN1 |
| 27 | KIF17 |
| 28 | LOC100287347 |
| 29 | CCK |
| 30 | RGS4 |
| 31 | GRIN2B |
| 32 | CHRM3 |
| 33 | MAST3 |
| 34 | ABR |
| 35 | NEUROD1 |
| 36 | RCAN3 |
| 37 | GALNTL5 |
| 38 | NR2E1 |
| 39 | BTN2A2 |
| 40 | PAX3 |
| 41 | SLC26A10 |
| 42 | NEK10 |
| 43 | CAMKV |
| 44 | TRIM58 |
| 45 | CBLB |
| 46 | CPNE4 |
| 47 | LHX6 |
| 48 | NETO1 |
| 49 | GDA |
| 50 | PCDHA5 |
| 51 | MEIS1 |
| 52 | MX1 |
| 53 | PTPRR |
| 54 | INADL |
| 55 | FAM153B |
| 56 | RGS14 |
| 57 | RMND5A |
| 58 | PRB2 |
| 59 | SGK223 |
| 60 | DUSP5 |
| 61 | CAMKK2 |
| 62 | CCDC103 |
| 63 | CBLN1 |
| 64 | FAM49A |
| 65 | LINC00599 |
| 66 | KCNF1 |
| 67 | TNFRSF25 |
| 68 | PPFIBP1 |
| 69 | DDX46 |
| 70 | SYNE4 |
| 71 | CLK4 |
| 72 | PLA2R1 |
| 73 | EPS8L2 |
| 74 | VWA5A |
| 75 | ABRACL |
| 76 | KALRN |
| 77 | RHOQ |
| 78 | TIAM2 |
| 79 | PCDH20 |
| 80 | ZBTB8A |
| 81 | NELF |
| 82 | DLGAP2 |
| 83 | MMD |
| 84 | ARHGAP24 |
| 85 | HSPB8 |
| 86 | ZNF219 |
| 87 | WSCD2 |
| 88 | COL13A1 |
| 89 | C8orf46 |
| 90 | PCID2 |
| 91 | RAB26 |
| 92 | PCDHA1 |
| 93 | SEL1L3 |
| 94 | THBS4 |
| 95 | KCNV1 |
| 96 | ATP4A |
| 97 | FAM161B |
| 98 | KHDRBS2 |
| 99 | HPSE2 |
| 100 | TTC39A |
| 101 | C14orf23 |
| 102 | FSTL5 |
| 103 | C15orf27 |
| 104 | EBF1 |
| 105 | CAMK2A |
| 106 | DGAT2 |
| 107 | FSCN1 |
| 108 | DLX1 |
| 109 | ICAM5 |
| 110 | CORT |
| 111 | ATRNL1 |
| 112 | PDE8B |
| 113 | LOC158696 |
| 114 | NPTX2 |
| 115 | CHD7 |
| 116 | RBFOX3 |
| 117 | KLHL3 |
| 118 | ARHGEF10L |
| 119 | RASL10A |
| 120 | TRPM3 |
| 121 | SLC25A33 |
| 122 | B3GAT2 |
| 123 | CTDSPL |
| 124 | LOC440084 |
| 125 | PRRT2 |
| 126 | RPRML |
| 127 | TMEM132B |
| 128 | PVRL3 |
| 129 | ARHGEF4 |
| 130 | REEP1 |
| 131 | CHSY3 |
| 132 | SYT5 |
| 133 | BCAT1 |
| 134 | FKBP9L |
| 135 | CADPS2 |
| 136 | CHI3L1 |
| 137 | CTDSPL2 |
| 138 | TRIM48 |
| 139 | CRISPLD1 |
| 140 | UNCX |
| 141 | ZBBX |
| 142 | TRIM9 |
| 143 | RNF152 |
| 144 | FLRT2 |
| 145 | RASGRF2 |
| 146 | SCARB2 |
| 147 | KLF13 |
| 148 | SRGAP2 |
| 149 | LOC729683 |
| 150 | NIPA1 |
| 151 | CAMK1G |
| 152 | FAM5B |
| 153 | BEND7 |
| 154 | CDH9 |
| 155 | SH2D5 |
| 156 | MAB21L1 |
| 157 | HNRNPU-AS1 |
| 158 | ADAMTS8 |
| 159 | GAS2 |
| 160 | DGCR5 |
| 161 | KIAA1456 |
| 162 | CHST1 |
| 163 | MXD4 |
| 164 | PCDH8 |
| 165 | TAC3 |
| 166 | SLC1A6 |
| 167 | KIAA1024 |
| 168 | DDX26B |
| 169 | NUP85 |
| 170 | ISOC1 |
| 171 | GRM5 |
| 172 | SOCS7 |
| 173 | PLCB4 |
| 174 | NEUROD6 |
| 175 | GLUD2 |
| 176 | PTPN5 |
| 177 | PPM1B |
| 178 | RBM5 |
| 179 | MGARP |
| 180 | ANKS1B |
| 181 | NECAB2 |
| 182 | GSTT2 |
| 183 | TIAM1 |
| 184 | LOC283174 |
| 185 | STK32C |
| 186 | SIPA1L2 |
| 187 | LRRC8B |
| 188 | AGAP4 |
| 189 | NRXN2 |
| 190 | EPHA4 |
| 191 | ODZ2 |
| 192 | ZFAND4 |
| 193 | LHX1 |
| 194 | ETV1 |
| 195 | LMO3 |
| 196 | ZIC4 |
| 197 | LZTS1 |
| 198 | DYNLT3 |
| 199 | PAK7 |
| 200 | PKIB |
| 201 | MPP3 |
| 202 | VPS41 |
| 203 | CDH8 |
| 204 | SLC8A2 |
| 205 | DHRS13 |
| 206 | CRH |
| 207 | C9orf91 |
| 208 | CDC25B |
| 209 | IDS |
| 210 | B3GALT2 |
| 211 | GABRA6 |
| 212 | PCDHA11 |
| 213 | LRRC6 |
| 214 | CHRD |
| 215 | ADAMTS19 |
| 216 | LONRF3 |
| 217 | SERPINI1 |
| 218 | HTR4 |
| 219 | SASH1 |
| 220 | LRRTM4 |
| 221 | AMICA1 |
| 222 | CPNE5 |
| 223 | GSDMB |
| 224 | TNS1 |
| 225 | COL6A1 |
| 226 | SOX11 |
| 227 | ENC1 |
| 228 | CABP1 |
| 229 | CTNNA2 |
| 230 | KCTD17 |
| 231 | CPT1A |
| 232 | TXNRD2 |
| 233 | SPA17 |
| 234 | NDST1 |
| 235 | ZIC1 |
| 236 | C18orf42 |
| 237 | C17orf108 |
| 238 | PODXL |
| 239 | LCOR |
| 240 | CNPY1 |
| 241 | C4orf50 |
| 242 | EBF3 |
| 243 | RFK |
| 244 | PALM2 |
| 245 | LOC150622 |
| 246 | ABTB1 |
| 247 | PTCHD1 |
| 248 | MMP17 |
| 249 | PEBP4 |
| 250 | C3orf80 |
| 251 | MYT1 |
| 252 | UCKL1 |
| 253 | H2BFXP |
| 254 | TRIM67 |
| 255 | LAMB1 |
| 256 | REV3L |
| 257 | MPP4 |
| 258 | HTR2A |
| 259 | MAL2 |
| 260 | SST |
| 261 | FAR2 |
| 262 | EGR3 |
| 263 | ZNF831 |
| 264 | NCALD |
| 265 | LDLRAP1 |
| 266 | C2orf80 |
| 267 | LNX1 |
| 268 | PELI2 |
| 269 | TSTD1 |
| 270 | C11orf58 |
| 271 | ITM2C |
| 272 | CRHR1 |
| 273 | CEP76 |
| 274 | MYBPHL |
| 275 | STK10 |
| 276 | NYNRIN |
| 277 | GABRA4 |
| 278 | USP45 |
| 279 | WEE1 |
| 280 | STBD1 |
| 281 | MGC16384 |
| 282 | LPPR5 |
| 283 | NGEF |
| 284 | SHC3 |
| 285 | RRAGD |
| 286 | BARHL1 |
| 287 | LPPR3 |
| 288 | ADAMTS3 |
| 289 | KCTD16 |
| 290 | KCNQ3 |
| 291 | SH3BGRL |
| 292 | ALS2 |
| 293 | SHPRH |
| 294 | ETS2 |
| 295 | FEZF2 |
| 296 | B9D1 |
| 297 | FLT3 |
| 298 | NFIA |
| 299 | LOC643037 |
| 300 | CCDC152 |
| 301 | SYT16 |
| 302 | POU3F2 |
| 303 | CMTM7 |
| 304 | SCN2A |
| 305 | ZNF551 |
| 306 | SAMD4A |
| 307 | ZDHHC23 |
| 308 | UNC13A |
| 309 | S100A1 |
| 310 | CNOT6 |
| 311 | FGF14 |
| 312 | TPCN2 |
| 313 | ANKRD30BL |
| 314 | MKL2 |
| 315 | CTXN1 |
| 316 | CATSPER2 |
| 317 | NRG3 |
| 318 | RUNX1T1 |
| 319 | TFAP2B |
| 320 | WDR16 |
| 321 | THUMPD2 |
| 322 | DUSP22 |
| 323 | CACNA1A |
| 324 | LIMA1 |
| 325 | HSPA4 |
| 326 | DLGAP1 |
| 327 | PLEKHA2 |
| 328 | LPPR4 |
| 329 | CHN2 |
| 330 | LCN8 |
| 331 | THRB |
| 332 | MCM3AP-AS1 |
| 333 | LRRC73 |
| 334 | RNFT2 |
| 335 | CRTAM |
| 336 | ALDH1A1 |
| 337 | ZNF823 |
| 338 | LINC00312 |
| 339 | RBMS3 |
| 340 | STX1A |
| 341 | PGAM2 |
| 342 | C2CD2 |
| 343 | GRIN2C |
| 344 | DYSF |
| 345 | TRIM50 |
| 346 | RNF148 |
| 347 | NUAK2 |
| 348 | FOXN3 |
| 349 | CSRNP3 |
| 350 | CRYGD |
| 351 | NINL |
| 352 | MOXD1 |
| 353 | FOXG1 |
| 354 | VIT |
| 355 | GNAL |
| 356 | USP42 |
| 357 | FBXL16 |
| 358 | GALNT12 |
| 359 | DLX2 |
| 360 | EGLN3 |
| 361 | KIAA0895L |
| 362 | GRIA2 |
| 363 | LGR6 |
| 364 | ZNF12 |
| 365 | CYP46A1 |
| 366 | XAF1 |
| 367 | CCDC92 |
| 368 | GALNT7 |
| 369 | PPIP5K1 |
| 370 | CACNG3 |
| 371 | C2orf55 |
| 372 | ARHGAP4 |
| 373 | LRRC4C |
| 374 | QPRT |
| 375 | LOC646627 |
| 376 | RPL3P2 |
| 377 | DGKB |
| 378 | RSAD2 |
| 379 | RORB |
| 380 | CLCNKA |
| 381 | LOC100288147 |
| 382 | P2RX4 |
| 383 | TOR2A |
| 384 | PDZK1 |
| 385 | MAP2K1 |
| 386 | KCNJ16 |
| 387 | RBP4 |
| 388 | 5-Sep |
| 389 | TSPAN1 |
| 390 | NEUROD2 |
| 391 | C16orf79 |
| 392 | OPLAH |
| 393 | GALNT9 |
| 394 | LRTM2 |
| 395 | TNAP |
| 396 | MRVI1 |
| 397 | SRRM4 |
| 398 | LOC100289230 |
| 399 | FAM19A1 |
| 400 | KCNIP3 |
| 401 | TMEM51 |
| 402 | DOCK11 |
| 403 | TIMP2 |
| 404 | ANXA11 |
| 405 | RNF128 |
| 406 | EXPH5 |
| 407 | FHL2 |
| 408 | FGF13 |
| 409 | EXTL1 |
| 410 | CX3CL1 |
| 411 | MDGA1 |
| 412 | LOC100131342 |
| 413 | IFIT3 |
| 414 | PRICKLE2 |
| 415 | CDH13 |
| 416 | FAP |
| 417 | LOC730091 |
| 418 | CDH23 |
| 419 | CNTLN |
| 420 | FAM153A |
| 421 | LOC100290023 |
| 422 | AGT |
| 423 | TMEM44 |
| 424 | ARSJ |
| 425 | PPP2R2C |
| 426 | DNMT1 |
| 427 | GRAMD1C |
| 428 | OCIAD2 |
| 429 | RAP2B |
| 430 | SNX32 |
| 431 | PPP1R3C |
| 432 | CD109 |
| 433 | CBS |
| 434 | NEFL |
| 435 | TMEM241 |
| 436 | STRC |
| 437 | CXCL14 |
| 438 | LACTB2 |
| 439 | PPP1R3D |
| 440 | GLT8D2 |
| 441 | FKBP9 |
| 442 | CA8 |
| 443 | GLCE |
| 444 | PDYN |
| 445 | LOC441204 |
| 446 | KCNA4 |
| 447 | USP1 |
| 448 | C1orf115 |
| 449 | ANO6 |
| 450 | EXOC6B |
| 451 | HPCA |
| 452 | FAM106A |
| 453 | PLK5 |
| 454 | MUS81 |
| 455 | JPH1 |
| 456 | RNF112 |
| 457 | ELP4 |
| 458 | WNT10B |
| 459 | ETV2 |
| 460 | PCDH10 |
| 461 | PTPRN2 |
| 462 | AFG3L1P |
| 463 | GRIK2 |
| 464 | POPDC3 |
| 465 | INO80D |
| 466 | LUZP2 |
| 467 | PLXDC2 |
| 468 | KIAA0664L3 |
| 469 | CPPED1 |
| 470 | PIRT |
| 471 | SPHKAP |
| 472 | PCDHA9 |
| 473 | TRANK1 |
| 474 | STYK1 |
| 475 | PLGLB1 |
| 476 | PRKAR2B |
| 477 | ANKRD30BP2 |
| 478 | AKAP5 |
| 479 | KCTD1 |
| 480 | USP3 |
| 481 | CAST |
| 482 | GRASP |
| 483 | UG0898H09 |
| 484 | YJEFN3 |
| 485 | ADAM11 |
| 486 | CALML4 |
| 487 | OTX1 |
| 488 | PDLIM2 |
| 489 | TRIM36 |
| 490 | TMEM155 |
| 491 | LOC100133299 |
| 492 | NT5E |
| 493 | FGF20 |
| 494 | GNB3 |
| 495 | DRD1 |
| 496 | CXADR |
| 497 | BACE2 |
| 498 | ABHD3 |
| 499 | FGFR1 |
| 500 | ARHGEF40 |

**(II) mRMR feature list**

| Order | Name |
| --- | --- |
| 1 | LRRC7 |
| 2 | TRAF3IP1 |
| 3 | STON1 |
| 4 | ABLIM1 |
| 5 | PAX3 |
| 6 | FAM161B |
| 7 | DAO |
| 8 | NR2E1 |
| 9 | PPM1B |
| 10 | ARHGEF6 |
| 11 | HOXA2 |
| 12 | FSCN1 |
| 13 | ZNF521 |
| 14 | PLA2R1 |
| 15 | GPRC5C |
| 16 | TIMP4 |
| 17 | DLX1 |
| 18 | PAX2 |
| 19 | NECAB1 |
| 20 | C11orf58 |
| 21 | IRX2 |
| 22 | SRGAP2 |
| 23 | HOXB2 |
| 24 | HINFP |
| 25 | RASAL1 |
| 26 | CHI3L1 |
| 27 | COL6A1 |
| 28 | DDX46 |
| 29 | GPR89B |
| 30 | GLRA1 |
| 31 | CCDC103 |
| 32 | CTDSPL2 |
| 33 | ANKS1B |
| 34 | PCP2 |
| 35 | SAMD4A |
| 36 | CLK4 |
| 37 | THUMPD2 |
| 38 | HOXA4 |
| 39 | TRPM3 |
| 40 | DLX6-AS1 |
| 41 | FOXJ2 |
| 42 | ARHGEF10L |
| 43 | GABRA5 |
| 44 | IPW |
| 45 | ZNF219 |
| 46 | PANK4 |
| 47 | MUS81 |
| 48 | MAB21L1 |
| 49 | RMND5A |
| 50 | USP45 |
| 51 | CXADR |
| 52 | CAST |
| 53 | THBS4 |
| 54 | CCNG2 |
| 55 | MEIS1 |
| 56 | TMEM51 |
| 57 | GRPEL1 |
| 58 | LGR6 |
| 59 | REV3L |
| 60 | GABRA6 |
| 61 | BTN2A2 |
| 62 | PRPH |
| 63 | TMEM191A |
| 64 | ZNF551 |
| 65 | ARHGAP4 |
| 66 | DLX2 |
| 67 | SHPRH |
| 68 | DUSP5 |
| 69 | HOXA3 |
| 70 | TXNRD2 |
| 71 | TFAP2B |
| 72 | KCNJ16 |
| 73 | CNOT6 |
| 74 | NPTX2 |
| 75 | C17orf108 |
| 76 | PCID2 |
| 77 | TTBK2 |
| 78 | ITPA |
| 79 | NYNRIN |
| 80 | ABR |
| 81 | ZNF12 |
| 82 | PPFIA2 |
| 83 | KCNF1 |
| 84 | KDM5C |
| 85 | NUP85 |
| 86 | IL1RAPL1 |
| 87 | RRAGD |
| 88 | APLN |
| 89 | PALM2 |
| 90 | OCIAD2 |
| 91 | GRIN2B |
| 92 | RAB11B |
| 93 | CNPY1 |
| 94 | NIM1 |
| 95 | VSTM2B |
| 96 | RNFT2 |
| 97 | PCDHA5 |
| 98 | TFAP2A |
| 99 | PPP1R3D |
| 100 | EXOC6B |
| 101 | SMPDL3A |
| 102 | SLC25A33 |
| 103 | DDX26B |
| 104 | HOOK1 |
| 105 | ALDH1A1 |
| 106 | OTX1 |
| 107 | KIAA1432 |
| 108 | WWOX |
| 109 | CBLB |
| 110 | HOXB3 |
| 111 | LHX1 |
| 112 | CCDC92 |
| 113 | LOC100287347 |
| 114 | ZBTB8A |
| 115 | CEP76 |
| 116 | CHST1 |
| 117 | PLIN4 |
| 118 | TRIM67 |
| 119 | CPT1A |
| 120 | NOL11 |
| 121 | BEND7 |
| 122 | NRXN2 |
| 123 | CAMKV |
| 124 | DNMT1 |
| 125 | TRIM58 |
| 126 | SCARA3 |
| 127 | CRTAM |
| 128 | KIAA1024 |
| 129 | ANO6 |
| 130 | FLJ42875 |
| 131 | EBF1 |
| 132 | GABRA4 |
| 133 | SNHG8 |
| 134 | LCOR |
| 135 | HPSE2 |
| 136 | CHN2 |
| 137 | OGDH |
| 138 | LINC00312 |
| 139 | RBM24 |
| 140 | FSTL5 |
| 141 | PDK2 |
| 142 | CLCNKA |
| 143 | GSTO2 |
| 144 | GLUD2 |
| 145 | CYP46A1 |
| 146 | FOXN3 |
| 147 | FKBP9L |
| 148 | THRSP |
| 149 | PDLIM2 |
| 150 | TSTD1 |
| 151 | TMEM44 |
| 152 | SLC38A3 |
| 153 | LOC654433 |
| 154 | KHDRBS2 |
| 155 | NTN1 |
| 156 | CYB5B |
| 157 | ZNF266 |
| 158 | PPIP5K2 |
| 159 | TIAM1 |
| 160 | LOC729683 |
| 161 | KLF13 |
| 162 | ADAMTS19 |
| 163 | ABLIM3 |
| 164 | ARHGEF4 |
| 165 | RBM5 |
| 166 | EBNA1BP2 |
| 167 | B4GALT2 |
| 168 | SYT7 |
| 169 | C9orf89 |
| 170 | RHBG |
| 171 | TNS1 |
| 172 | LOC158696 |
| 173 | GTF3C5 |
| 174 | CHN1 |
| 175 | GRAMD1C |
| 176 | OSBPL2 |
| 177 | MIR100HG |
| 178 | JTB |
| 179 | USP42 |
| 180 | BARHL1 |
| 181 | SNRNP48 |
| 182 | KALRN |
| 183 | SLC25A27 |
| 184 | PRDX6 |
| 185 | CRISPLD1 |
| 186 | HOXB5 |
| 187 | FAM49A |
| 188 | P2RX4 |
| 189 | STK32C |
| 190 | LOC441722 |
| 191 | ZBED1 |
| 192 | LRRC6 |
| 193 | TARBP2 |
| 194 | TIAM2 |
| 195 | SIPA1L2 |
| 196 | LRRC73 |
| 197 | CAB39 |
| 198 | EPS8L2 |
| 199 | UCKL1 |
| 200 | ASTN1 |
| 201 | PRDM5 |
| 202 | FAR2 |
| 203 | ANO10 |
| 204 | FLJ41350 |
| 205 | TOR2A |
| 206 | ACTC1 |
| 207 | CDH8 |
| 208 | STK10 |
| 209 | WEE1 |
| 210 | PCDH20 |
| 211 | NECAB2 |
| 212 | FERMT1 |
| 213 | RPS21 |
| 214 | KRT18P55 |
| 215 | MAST3 |
| 216 | SKOR1 |
| 217 | DDIT4L |
| 218 | TOP2B |
| 219 | SGK223 |
| 220 | EBF3 |
| 221 | KHDRBS1 |
| 222 | PTPRR |
| 223 | GLUD1 |
| 224 | WAC |
| 225 | MMP17 |
| 226 | FBXO30 |
| 227 | EMX2OS |
| 228 | CD109 |
| 229 | INF2 |
| 230 | LPPR5 |
| 231 | PP7080 |
| 232 | PODXL |
| 233 | SEPHS1 |
| 234 | PCDHA11 |
| 235 | ZFC3H1 |
| 236 | TTC39B |
| 237 | ODZ2 |
| 238 | DDN |
| 239 | GALNT7 |
| 240 | ANKRD12 |
| 241 | NEK10 |
| 242 | ITPR2 |
| 243 | HOGA1 |
| 244 | ZNF337 |
| 245 | HOXA7 |
| 246 | PPFIBP1 |
| 247 | CDC25B |
| 248 | KRT18P26 |
| 249 | PRB2 |
| 250 | LOC100129201 |
| 251 | HSPB8 |
| 252 | SLC26A5 |
| 253 | MAL2 |
| 254 | IGSF22 |
| 255 | FAT2 |
| 256 | ABRACL |
| 257 | MFSD2A |
| 258 | LRRTM2 |
| 259 | LDB2 |
| 260 | LCN8 |
| 261 | YJEFN3 |
| 262 | GRB7 |
| 263 | CCDC152 |
| 264 | PCDHA1 |
| 265 | MSX2P1 |
| 266 | KLHL3 |
| 267 | ETV2 |
| 268 | WSCD2 |
| 269 | RPS3A |
| 270 | B9D1 |
| 271 | ATP1B2 |
| 272 | SPSB3 |
| 273 | MPP3 |
| 274 | RHOQ |
| 275 | KLK8 |
| 276 | SLC16A5 |
| 277 | ANKRD30BL |
| 278 | ERGIC2 |
| 279 | FGF13 |
| 280 | GUK1 |
| 281 | KIF21A |
| 282 | ALS2 |
| 283 | LOC100288147 |
| 284 | AMICA1 |
| 285 | UHRF2 |
| 286 | HTR4 |
| 287 | FLJ31485 |
| 288 | TRIM50 |
| 289 | PDSS1 |
| 290 | PDE1A |
| 291 | ARHGEF40 |
| 292 | CCNL2 |
| 293 | DPYSL4 |
| 294 | INO80D |
| 295 | NDST1 |
| 296 | NINL |
| 297 | FZD6 |
| 298 | INADL |
| 299 | HIPK1 |
| 300 | ATPAF1 |
| 301 | PDE10A |
| 302 | SCARB2 |
| 303 | HOXA5 |
| 304 | CSPG4 |
| 305 | CDC42SE2 |
| 306 | RBMS3 |
| 307 | FGFR1 |
| 308 | DLX6 |
| 309 | PTCHD1 |
| 310 | CCDC155 |
| 311 | EPHA4 |
| 312 | SH3BP5 |
| 313 | ANAPC15 |
| 314 | PEBP4 |
| 315 | LPPR4 |
| 316 | PDZK1P1 |
| 317 | CDKL5 |
| 318 | SNHG10 |
| 319 | SPA17 |
| 320 | MRFAP1 |
| 321 | CHD7 |
| 322 | MX1 |
| 323 | MOB3C |
| 324 | LBH |
| 325 | MTHFD1L |
| 326 | PAK7 |
| 327 | ANGPT1 |
| 328 | PILRA |
| 329 | SYT2 |
| 330 | USP44 |
| 331 | GALNTL5 |
| 332 | GRM5 |
| 333 | TPCN2 |
| 334 | C12orf26 |
| 335 | MMD |
| 336 | ZC3H18 |
| 337 | XAF1 |
| 338 | TRIM55 |
| 339 | LOC100288929 |
| 340 | TOB2P1 |
| 341 | ILF3 |
| 342 | ITPKA |
| 343 | PPP1R3C |
| 344 | CACNB4 |
| 345 | GALNT12 |
| 346 | B4GALT1 |
| 347 | KCTD13 |
| 348 | PFDN1 |
| 349 | MGARP |
| 350 | EYA2 |
| 351 | RNF152 |
| 352 | STBD1 |
| 353 | ZNF653 |
| 354 | SH2D5 |
| 355 | TMEFF2 |
| 356 | AGAP4 |
| 357 | EMX2 |
| 358 | ARX |
| 359 | GRIN2C |
| 360 | FXR1 |
| 361 | PCDH10 |
| 362 | TRANK1 |
| 363 | DGAT2 |
| 364 | LONRF3 |
| 365 | NAV1 |
| 366 | IDS |
| 367 | CLK2 |
| 368 | NFIA |
| 369 | SORBS3 |
| 370 | BAHCC1 |
| 371 | CRHR1 |
| 372 | UNCX |
| 373 | PICK1 |
| 374 | RAB22A |
| 375 | PDE2A |
| 376 | CD83 |
| 377 | NELF |
| 378 | JMJD1C |
| 379 | LAMB1 |
| 380 | SMYD4 |
| 381 | CD2AP |
| 382 | GABRB2 |
| 383 | SUB1 |
| 384 | AKAP13 |
| 385 | CMTM7 |
| 386 | STK32A |
| 387 | TDRD6 |
| 388 | ATAD5 |
| 389 | PTK2B |
| 390 | REEP1 |
| 391 | FLT3 |
| 392 | ZNF552 |
| 393 | SH3BGRL |
| 394 | CSRNP3 |
| 395 | NCOA6 |
| 396 | SHISA6 |
| 397 | LRRC8C |
| 398 | CAMK1G |
| 399 | BRAP |
| 400 | CPNE4 |
| 401 | SOD2 |
| 402 | SMC1A |
| 403 | FKBP9 |
| 404 | CBLN1 |
| 405 | MTPN |
| 406 | LOC202781 |
| 407 | NISCH |
| 408 | SLC26A10 |
| 409 | CROCCP2 |
| 410 | ZNF33A |
| 411 | LOC646043 |
| 412 | MOXD1 |
| 413 | AHCYL1 |
| 414 | STRC |
| 415 | C12orf39 |
| 416 | C17orf70 |
| 417 | GLCE |
| 418 | KCNMB4 |
| 419 | DKFZP434I0714 |
| 420 | CAMKK2 |
| 421 | VGF |
| 422 | HOXA6 |
| 423 | SASH1 |
| 424 | PRPF6 |
| 425 | LHX6 |
| 426 | LOC727982 |
| 427 | HSPA9 |
| 428 | NUDT14 |
| 429 | SOCS7 |
| 430 | MSX2 |
| 431 | C5orf38 |
| 432 | TRIM11 |
| 433 | ACSBG1 |
| 434 | KRT18 |
| 435 | ABCD2 |
| 436 | LRRC10B |
| 437 | B3GAT2 |
| 438 | ITPRIPL1 |
| 439 | SYT5 |
| 440 | UBA3 |
| 441 | JARID2 |
| 442 | PRORSD1P |
| 443 | CAMK4 |
| 444 | GRHL3 |
| 445 | ACN9 |
| 446 | POLB |
| 447 | CGNL1 |
| 448 | RYBP |
| 449 | KCTD17 |
| 450 | VWA5A |
| 451 | ZNF596 |
| 452 | ITGA7 |
| 453 | ABTB1 |
| 454 | PAG1 |
| 455 | NPAS2 |
| 456 | PTRH1 |
| 457 | ANK2 |
| 458 | DUSP22 |
| 459 | RBM10 |
| 460 | RTN4RL1 |
| 461 | RIIAD1 |
| 462 | MCM3AP-AS1 |
| 463 | SPECC1L |
| 464 | SDC3 |
| 465 | TMSB10 |
| 466 | CDH23 |
| 467 | PADI2 |
| 468 | RNF115 |
| 469 | ANKRD55 |
| 470 | UNC45A |
| 471 | DHRS13 |
| 472 | ARHGAP29 |
| 473 | GFAP |
| 474 | LOC100289230 |
| 475 | B3GALT2 |
| 476 | SPHAR |
| 477 | CEP85L |
| 478 | LOC646627 |
| 479 | FHOD3 |
| 480 | LMCD1 |
| 481 | FUT2 |
| 482 | DYSF |
| 483 | LY6K |
| 484 | ARID2 |
| 485 | ZDHHC16 |
| 486 | PCSK9 |
| 487 | SPINK6 |
| 488 | MAP2K1 |
| 489 | LMBR1L |
| 490 | ANGPTL2 |
| 491 | LPPR3 |
| 492 | EOMES |
| 493 | LEF1 |
| 494 | MKL2 |
| 495 | EN2 |
| 496 | SWAP70 |
| 497 | CALML4 |
| 498 | LOC728470 |
| 499 | C4orf19 |
| 500 | COL13A1 |

1. **H0351.1016**

**(I) MaxRel feature list**

| Order | Name |
| --- | --- |
| 1 | DAO |
| 2 | PAX3 |
| 3 | LRRC7 |
| 4 | STON1 |
| 5 | NR2E1 |
| 6 | GABRA5 |
| 7 | TIMP4 |
| 8 | KIF17 |
| 9 | NRGN |
| 10 | ZNF521 |
| 11 | FAM81A |
| 12 | ABLIM1 |
| 13 | CCNG2 |
| 14 | CBLN1 |
| 15 | MEIS1 |
| 16 | HSPB8 |
| 17 | AK5 |
| 18 | RASAL1 |
| 19 | CREG2 |
| 20 | CTDSPL2 |
| 21 | NIPA1 |
| 22 | LINC00473 |
| 23 | PDE2A |
| 24 | NECAB1 |
| 25 | SRGAP2 |
| 26 | LDB2 |
| 27 | SYNE4 |
| 28 | C11orf58 |
| 29 | NELF |
| 30 | DLGAP2 |
| 31 | NETO1 |
| 32 | MAB21L1 |
| 33 | PLK2 |
| 34 | CCK |
| 35 | CHRM3 |
| 36 | DLX6-AS1 |
| 37 | CAMKV |
| 38 | CTNNA2 |
| 39 | CDH8 |
| 40 | KHDRBS1 |
| 41 | INADL |
| 42 | MMD |
| 43 | MAST3 |
| 44 | SEL1L3 |
| 45 | FRAS1 |
| 46 | CLK4 |
| 47 | KHDRBS2 |
| 48 | EBF1 |
| 49 | GALNT12 |
| 50 | CADPS2 |
| 51 | RNF152 |
| 52 | CAMK4 |
| 53 | MGARP |
| 54 | PODXL |
| 55 | HINFP |
| 56 | RGS4 |
| 57 | SLC1A6 |
| 58 | XAF1 |
| 59 | NUP85 |
| 60 | FLJ42875 |
| 61 | ICAM5 |
| 62 | DUSP5 |
| 63 | GDA |
| 64 | SUMO2 |
| 65 | LHX1 |
| 66 | PRICKLE2 |
| 67 | CBLB |
| 68 | SLC26A10 |
| 69 | GRID2 |
| 70 | ZFAND4 |
| 71 | GPRC5C |
| 72 | CAST |
| 73 | RHOQ |
| 74 | SYT5 |
| 75 | LOC440084 |
| 76 | TIAM1 |
| 77 | BARHL1 |
| 78 | SIPA1L2 |
| 79 | FAM5B |
| 80 | CHD7 |
| 81 | TNS1 |
| 82 | ZIC4 |
| 83 | COL13A1 |
| 84 | C15orf27 |
| 85 | SLC35F4 |
| 86 | KCNV1 |
| 87 | LHX6 |
| 88 | FOXA2 |
| 89 | KIAA1024 |
| 90 | ETV1 |
| 91 | LINC00599 |
| 92 | CPNE4 |
| 93 | RPS3A |
| 94 | NDST1 |
| 95 | TMEM132B |
| 96 | CEP76 |
| 97 | RGS14 |
| 98 | TMEM44 |
| 99 | SLC6A7 |
| 100 | KIAA1456 |
| 101 | ZNF823 |
| 102 | IRX2 |
| 103 | PCDH20 |
| 104 | WSCD2 |
| 105 | RASL10A |
| 106 | FKBP9L |
| 107 | CNPY1 |
| 108 | GRIN2B |
| 109 | ATP4A |
| 110 | RAB11B |
| 111 | C10orf107 |
| 112 | TRIM58 |
| 113 | BCL6 |
| 114 | VIT |
| 115 | ITPKA |
| 116 | C12orf26 |
| 117 | APBB2 |
| 118 | DGAT2 |
| 119 | CHI3L1 |
| 120 | FLRT2 |
| 121 | TIMP2 |
| 122 | PLA2R1 |
| 123 | PLCB1 |
| 124 | TFAP2B |
| 125 | NEFM |
| 126 | COQ10A |
| 127 | C8orf46 |
| 128 | LOC202781 |
| 129 | TARBP2 |
| 130 | KCNC2 |
| 131 | LOC100287347 |
| 132 | KCNF1 |
| 133 | EMX2 |
| 134 | NRG3 |
| 135 | DLX2 |
| 136 | LOC643037 |
| 137 | ARVCF |
| 138 | DDX46 |
| 139 | NECAB2 |
| 140 | GLCE |
| 141 | PKIB |
| 142 | KALRN |
| 143 | AMICA1 |
| 144 | USP45 |
| 145 | NEUROD1 |
| 146 | GABRA6 |
| 147 | RASGRF2 |
| 148 | PPFIBP1 |
| 149 | TAC3 |
| 150 | PRSS55 |
| 151 | CHSY3 |
| 152 | ADAMTS19 |
| 153 | PCP2 |
| 154 | FLT3 |
| 155 | RBFOX3 |
| 156 | ODZ2 |
| 157 | SCARB2 |
| 158 | ABR |
| 159 | DUSP12 |
| 160 | PAK7 |
| 161 | LOC654433 |
| 162 | FHL2 |
| 163 | LOC100287987 |
| 164 | RMND5A |
| 165 | S100A1 |
| 166 | ACTN1 |
| 167 | STYK1 |
| 168 | UNCX |
| 169 | ISOC1 |
| 170 | SNHG8 |
| 171 | EXPH5 |
| 172 | EEF1DP3 |
| 173 | CCT7 |
| 174 | CTXN1 |
| 175 | GCOM1 |
| 176 | SYT6 |
| 177 | RCAN3 |
| 178 | GFOD2 |
| 179 | CGNL1 |
| 180 | MYT1 |
| 181 | SERPINI1 |
| 182 | ZBBX |
| 183 | PCDHA8 |
| 184 | ZFHX3 |
| 185 | CDKL5 |
| 186 | NFE2L1 |
| 187 | PRB2 |
| 188 | LOC283174 |
| 189 | MCTP1 |
| 190 | ZNF385D |
| 191 | FAM49A |
| 192 | C14orf23 |
| 193 | VWA5A |
| 194 | CHRD |
| 195 | B3GALT2 |
| 196 | KIAA0895L |
| 197 | C2orf72 |
| 198 | GSTT2 |
| 199 | TMEM150C |
| 200 | LOC100288105 |
| 201 | SPINK6 |
| 202 | B3GAT2 |
| 203 | RELN |
| 204 | ZNF800 |
| 205 | CAMK1G |
| 206 | RASAL2 |
| 207 | BTN2A2 |
| 208 | TNAP |
| 209 | CORT |
| 210 | MPP3 |
| 211 | LOC729683 |
| 212 | H2BFXP |
| 213 | GLT8D2 |
| 214 | GRIN2C |
| 215 | SASH1 |
| 216 | ATRNL1 |
| 217 | CHST1 |
| 218 | SRGAP2C |
| 219 | EDIL3 |
| 220 | CHN1 |
| 221 | ARHGAP29 |
| 222 | TRIM67 |
| 223 | SLITRK1 |
| 224 | RHOBTB2 |
| 225 | ARHGAP24 |
| 226 | KRT19 |
| 227 | C2orf80 |
| 228 | DOCK11 |
| 229 | CAMK2A |
| 230 | LOC100287005 |
| 231 | PLK5 |
| 232 | CRISPLD1 |
| 233 | PPT2 |
| 234 | ZIC1 |
| 235 | MPP4 |
| 236 | MXD4 |
| 237 | EPS8L2 |
| 238 | MOXD1 |
| 239 | OSBPL3 |
| 240 | RNF148 |
| 241 | ELP4 |
| 242 | FGFR1 |
| 243 | GLUD1 |
| 244 | B9D2 |
| 245 | SNHG1 |
| 246 | THUMPD2 |
| 247 | CRTAM |
| 248 | ALDH1A1 |
| 249 | RPL3P2 |
| 250 | GAB2 |
| 251 | STK10 |
| 252 | DDN |
| 253 | ARHGEF4 |
| 254 | SNHG10 |
| 255 | LINC00312 |
| 256 | TMEM61 |
| 257 | NPM3 |
| 258 | STK32C |
| 259 | MATK |
| 260 | STBD1 |
| 261 | LCN8 |
| 262 | RBP4 |
| 263 | EXTL1 |
| 264 | USP3 |
| 265 | ARHGAP4 |
| 266 | LOC644950 |
| 267 | ZNF238 |
| 268 | B9D1 |
| 269 | ARNT2 |
| 270 | ALK |
| 271 | UBASH3B |
| 272 | IFIT3 |
| 273 | PLEKHA2 |
| 274 | EBF3 |
| 275 | SHF |
| 276 | FKBP9 |
| 277 | TNFRSF25 |
| 278 | ADAMTS8 |
| 279 | SCARA3 |
| 280 | MBP |
| 281 | CYB5A |
| 282 | CDH13 |
| 283 | IRF6 |
| 284 | DOPEY2 |
| 285 | NT5E |
| 286 | KIAA1598 |
| 287 | TSTD1 |
| 288 | PLCB4 |
| 289 | PCDH8 |
| 290 | CRH |
| 291 | REL |
| 292 | LUZP2 |
| 293 | EGR3 |
| 294 | RHBG |
| 295 | CNTN6 |
| 296 | MAOB |
| 297 | POU3F2 |
| 298 | PELI2 |
| 299 | DNMT1 |
| 300 | NEUROD6 |
| 301 | TFAP2A |
| 302 | SPON2 |
| 303 | MKL2 |
| 304 | PTPN5 |
| 305 | ALS2 |
| 306 | LIMA1 |
| 307 | LPCAT2 |
| 308 | C3orf35 |
| 309 | SLC22A31 |
| 310 | LOC439914 |
| 311 | PAX6 |
| 312 | SNRNP48 |
| 313 | ACLY |
| 314 | KCNJ3 |
| 315 | CIDEB |
| 316 | EIF3L |
| 317 | FSTL5 |
| 318 | UBTD2 |
| 319 | LRRC73 |
| 320 | FAP |
| 321 | ELFN2 |
| 322 | SORT1 |
| 323 | CCDC152 |
| 324 | MX1 |
| 325 | NPTX2 |
| 326 | TLN2 |
| 327 | TMEM155 |
| 328 | NFIA |
| 329 | ZNF330 |
| 330 | LARP1 |
| 331 | FUT2 |
| 332 | FDX1L |
| 333 | KLHL3 |
| 334 | FAM213A |
| 335 | ASAP1 |
| 336 | LRRC8C |
| 337 | KLF11 |
| 338 | LOC100290023 |
| 339 | ELL3 |
| 340 | RIIAD1 |
| 341 | DYSF |
| 342 | LOC100131289 |
| 343 | HSPA4 |
| 344 | CA4 |
| 345 | USP14 |
| 346 | KCNQ3 |
| 347 | KCNK1 |
| 348 | PTPRR |
| 349 | NGEF |
| 350 | PMEPA1 |
| 351 | GRK4 |
| 352 | LOXL2 |
| 353 | USP44 |
| 354 | HDAC1 |
| 355 | SHC3 |
| 356 | CYP46A1 |
| 357 | ATP2B1 |
| 358 | ZNF702P |
| 359 | KDM4D |
| 360 | SOX11 |
| 361 | EIF3E |
| 362 | LRRC8B |
| 363 | C9orf91 |
| 364 | GALNTL5 |
| 365 | FAM19A1 |
| 366 | CERKL |
| 367 | C17orf108 |
| 368 | SH2D5 |
| 369 | CARTPT |
| 370 | DHRS13 |
| 371 | ARHGEF10L |
| 372 | NRK |
| 373 | SGCA |
| 374 | SHROOM3 |
| 375 | USP1 |
| 376 | PPP2R2B |
| 377 | ABRACL |
| 378 | TMSB10 |
| 379 | LPPR3 |
| 380 | EPC1 |
| 381 | MYO5C |
| 382 | PCSK9 |
| 383 | SPTBN5 |
| 384 | SLIT2 |
| 385 | POU2F1 |
| 386 | FGF20 |
| 387 | KCTD16 |
| 388 | CDH15 |
| 389 | ENC1 |
| 390 | NCRNA00185 |
| 391 | LGR6 |
| 392 | EPB41L4A-AS1 |
| 393 | HTR2A |
| 394 | DLX1 |
| 395 | LONRF3 |
| 396 | EPAS1 |
| 397 | JMJD7 |
| 398 | LOC158696 |
| 399 | PDE8B |
| 400 | POLB |
| 401 | TXNRD2 |
| 402 | RBMS3 |
| 403 | LOC100128822 |
| 404 | DGKB |
| 405 | CLCNKB |
| 406 | MEGF11 |
| 407 | CA9 |
| 408 | NGDN |
| 409 | RAB26 |
| 410 | RSAD2 |
| 411 | NOL11 |
| 412 | SOGA2 |
| 413 | C20orf3 |
| 414 | ARHGAP32 |
| 415 | LINC00526 |
| 416 | VAMP1 |
| 417 | FBXO5 |
| 418 | MTHFD1L |
| 419 | ALCAM |
| 420 | RPRML |
| 421 | ZDHHC2 |
| 422 | ANO3 |
| 423 | FLJ43663 |
| 424 | ANKS1B |
| 425 | AGBL4 |
| 426 | HIST1H1D |
| 427 | AMT |
| 428 | NXPH3 |
| 429 | ZBED5 |
| 430 | PRSS22 |
| 431 | TMEM91 |
| 432 | KLK8 |
| 433 | ZNF663 |
| 434 | TANC2 |
| 435 | LDLRAP1 |
| 436 | LMO3 |
| 437 | EPB41L5 |
| 438 | PDZK1 |
| 439 | PCDH7 |
| 440 | P2RX4 |
| 441 | HPSE2 |
| 442 | CMTM7 |
| 443 | PLCB2 |
| 444 | SHISA9 |
| 445 | BCAS1 |
| 446 | POLR2H |
| 447 | RSPO4 |
| 448 | CDH9 |
| 449 | C10orf105 |
| 450 | DACH2 |
| 451 | PRRG1 |
| 452 | GSTO2 |
| 453 | CNOT6 |
| 454 | ZNF143 |
| 455 | CPNE5 |
| 456 | FREM1 |
| 457 | KCNA4 |
| 458 | PALM2 |
| 459 | EOMES |
| 460 | PCDH10 |
| 461 | LTA4H |
| 462 | GNAL |
| 463 | SYNE2 |
| 464 | PCSK1 |
| 465 | LCOR |
| 466 | ZNF671 |
| 467 | COL11A1 |
| 468 | ZP2 |
| 469 | SYT16 |
| 470 | EPB41L2 |
| 471 | ASXL3 |
| 472 | CTDSPL |
| 473 | CXCL14 |
| 474 | BUB1B |
| 475 | SH3KBP1 |
| 476 | LHX2 |
| 477 | TMCO3 |
| 478 | RPL17 |
| 479 | LMNB1 |
| 480 | GABRD |
| 481 | PPFIA2 |
| 482 | KIAA0930 |
| 483 | ABLIM3 |
| 484 | PAG1 |
| 485 | RNF115 |
| 486 | MAN2B2 |
| 487 | C18orf42 |
| 488 | RNF43 |
| 489 | DUSP22 |
| 490 | LZTS1 |
| 491 | CCDC88A |
| 492 | CHST15 |
| 493 | BACE2 |
| 494 | ACD |
| 495 | NIM1 |
| 496 | AGMAT |
| 497 | CA8 |
| 498 | NUAK2 |
| 499 | LHFP |
| 500 | EMX2OS |

**(II) mRMR feature list**

| Order | Name |
| --- | --- |
| 1 | DAO |
| 2 | FLJ43663 |
| 3 | NR2E1 |
| 4 | PAX3 |
| 5 | LOC100287521 |
| 6 | STON1 |
| 7 | CCT7 |
| 8 | SUMO2 |
| 9 | PCP2 |
| 10 | NPM3 |
| 11 | KDM4D |
| 12 | CNPY1 |
| 13 | ABLIM1 |
| 14 | HOXB2 |
| 15 | MEIS1 |
| 16 | MAB21L1 |
| 17 | KHDRBS1 |
| 18 | PAX2 |
| 19 | GLRA1 |
| 20 | TIMP4 |
| 21 | RASAL1 |
| 22 | NELF |
| 23 | TARBP2 |
| 24 | NECAB1 |
| 25 | SRGAP2 |
| 26 | LOXL2 |
| 27 | PPT2 |
| 28 | HOXA3 |
| 29 | CTDSPL2 |
| 30 | GSTO2 |
| 31 | SCARB2 |
| 32 | TFAP2B |
| 33 | CEP76 |
| 34 | MGARP |
| 35 | ANKS1B |
| 36 | NIPA1 |
| 37 | IFIT3 |
| 38 | LOC644950 |
| 39 | PODXL |
| 40 | HSPB8 |
| 41 | PLA2R1 |
| 42 | CDKL5 |
| 43 | FAM161B |
| 44 | TFAP2A |
| 45 | CAST |
| 46 | ZNF800 |
| 47 | CBLN1 |
| 48 | ASAH1 |
| 49 | HINFP |
| 50 | SLC35F4 |
| 51 | NIM1 |
| 52 | RAB22A |
| 53 | C11orf58 |
| 54 | HOXA2 |
| 55 | GABRA5 |
| 56 | DLX2 |
| 57 | GALNT12 |
| 58 | RAB11B |
| 59 | CHI3L1 |
| 60 | ANO10 |
| 61 | RNF152 |
| 62 | CCDC12 |
| 63 | NUP85 |
| 64 | GPR89C |
| 65 | LOC654433 |
| 66 | TRAF3IP1 |
| 67 | CDH8 |
| 68 | OTX1 |
| 69 | C10orf107 |
| 70 | IRX2 |
| 71 | LOC100131754 |
| 72 | KATNB1 |
| 73 | SYT2 |
| 74 | THUMPD2 |
| 75 | FKBP9L |
| 76 | TMEM44 |
| 77 | UBASH3B |
| 78 | ZNF33A |
| 79 | CTNNA2 |
| 80 | LHX1 |
| 81 | HOXB3 |
| 82 | ZNF521 |
| 83 | GSS |
| 84 | KIAA1024 |
| 85 | KLK8 |
| 86 | C11orf31 |
| 87 | LRRC7 |
| 88 | SPTBN1 |
| 89 | SPINK6 |
| 90 | SSFA2 |
| 91 | ARVCF |
| 92 | EBF1 |
| 93 | ARHGAP4 |
| 94 | KIF17 |
| 95 | C14orf105 |
| 96 | USP45 |
| 97 | DNMT1 |
| 98 | EMX2 |
| 99 | HOXA4 |
| 100 | F8 |
| 101 | TIAM1 |
| 102 | RIIAD1 |
| 103 | INADL |
| 104 | TMEM8B |
| 105 | GABRA6 |
| 106 | EMX2OS |
| 107 | CAMKV |
| 108 | USP14 |
| 109 | TERF1 |
| 110 | FRAS1 |
| 111 | NRK |
| 112 | KCNF1 |
| 113 | NFE2L1 |
| 114 | GPR89B |
| 115 | SNHG8 |
| 116 | DDX59 |
| 117 | GPRC5C |
| 118 | STBD1 |
| 119 | LGR6 |
| 120 | ZNF79 |
| 121 | PRKAA1 |
| 122 | PCSK9 |
| 123 | PNPLA7 |
| 124 | POLB |
| 125 | DLX6-AS1 |
| 126 | GLUD1 |
| 127 | B9D1 |
| 128 | URI1 |
| 129 | ACLY |
| 130 | EPB41L4A-AS1 |
| 131 | LMBR1L |
| 132 | DUSP5 |
| 133 | CDK8 |
| 134 | ABLIM3 |
| 135 | RASL10A |
| 136 | SH3BGRL |
| 137 | SAMD4A |
| 138 | TOR2A |
| 139 | CCNG2 |
| 140 | AMICA1 |
| 141 | ACTN1 |
| 142 | SLC22A31 |
| 143 | VAMP1 |
| 144 | RP2 |
| 145 | EBF3 |
| 146 | C12orf26 |
| 147 | GRID2 |
| 148 | TNS1 |
| 149 | PPP1R35 |
| 150 | UBA3 |
| 151 | ADAMTS19 |
| 152 | B9D2 |
| 153 | NOL11 |
| 154 | KCNJ16 |
| 155 | LCN8 |
| 156 | TMSB10 |
| 157 | OR13J1 |
| 158 | MAST3 |
| 159 | PRPH |
| 160 | ZDHHC20 |
| 161 | TMCO3 |
| 162 | BARHL1 |
| 163 | LINC00312 |
| 164 | DLGAP2 |
| 165 | SPATA5L1 |
| 166 | WWOX |
| 167 | PDK2 |
| 168 | FLJ42875 |
| 169 | JMJD7 |
| 170 | FAM5B |
| 171 | HPX |
| 172 | GXYLT1 |
| 173 | NECAB2 |
| 174 | ZNF567 |
| 175 | CHN1 |
| 176 | MSX2 |
| 177 | FEM1A |
| 178 | PRPF6 |
| 179 | PCDH20 |
| 180 | UTRN |
| 181 | OGFRL1 |
| 182 | IDS |
| 183 | ACBD6 |
| 184 | CLK4 |
| 185 | FOXA2 |
| 186 | NGDN |
| 187 | PRSS55 |
| 188 | TTBK2 |
| 189 | GFOD2 |
| 190 | PPP4R1 |
| 191 | RHBG |
| 192 | PRDM5 |
| 193 | ZFAND4 |
| 194 | EXOC6B |
| 195 | RASAL2 |
| 196 | SHPRH |
| 197 | ANGPT1 |
| 198 | COL6A1 |
| 199 | TSTD1 |
| 200 | NRG4 |
| 201 | FBXO5 |
| 202 | HOXA7 |
| 203 | LOC100128822 |
| 204 | ODZ2 |
| 205 | BCL6 |
| 206 | GCOM1 |
| 207 | DNAJB5 |
| 208 | DLX1 |
| 209 | FUT2 |
| 210 | ITPR2 |
| 211 | GLCE |
| 212 | TRIM58 |
| 213 | ALDH1A1 |
| 214 | GUK1 |
| 215 | HIPK1 |
| 216 | RSRC2 |
| 217 | CEP41 |
| 218 | FKBP9 |
| 219 | GPRIN3 |
| 220 | ARHGAP29 |
| 221 | FLJ41350 |
| 222 | LDB2 |
| 223 | IPW |
| 224 | SOCS5 |
| 225 | FGFR1 |
| 226 | UHMK1 |
| 227 | DZANK1 |
| 228 | ZNF823 |
| 229 | CBLB |
| 230 | KIAA1614 |
| 231 | RAB21 |
| 232 | RALGAPA1 |
| 233 | PRSS22 |
| 234 | WEE1 |
| 235 | USP44 |
| 236 | SMOC2 |
| 237 | LIPG |
| 238 | TRIM67 |
| 239 | CYB5B |
| 240 | CYP46A1 |
| 241 | GAB2 |
| 242 | CDIPT |
| 243 | WDR13 |
| 244 | MTHFD1L |
| 245 | TRPM3 |
| 246 | HSPA4 |
| 247 | XAF1 |
| 248 | TUBE1 |
| 249 | ASAP1 |
| 250 | SGCA |
| 251 | KIAA0564 |
| 252 | ACSBG1 |
| 253 | ZNF330 |
| 254 | LAMC1 |
| 255 | CCDC152 |
| 256 | UBXN2A |
| 257 | TMEM249 |
| 258 | FAM81A |
| 259 | EPB41L1 |
| 260 | PPFIA2 |
| 261 | WSCD2 |
| 262 | EN2 |
| 263 | NDST1 |
| 264 | KALRN |
| 265 | HIST1H1B |
| 266 | EPAS1 |
| 267 | OCIAD2 |
| 268 | LARP1 |
| 269 | RCE1 |
| 270 | MOXD1 |
| 271 | CMTM7 |
| 272 | KIF21A |
| 273 | ALCAM |
| 274 | FSCN1 |
| 275 | ZCCHC9 |
| 276 | CCDC144NL |
| 277 | SCARA3 |
| 278 | C17orf108 |
| 279 | EPHA4 |
| 280 | POU2F1 |
| 281 | EOMES |
| 282 | ABHD3 |
| 283 | ARHGEF6 |
| 284 | APBB2 |
| 285 | SLAIN2 |
| 286 | NEFM |
| 287 | ZNF143 |
| 288 | BZW2 |
| 289 | C9orf72 |
| 290 | SLC6A7 |
| 291 | TMEM150C |
| 292 | DPH2 |
| 293 | ABR |
| 294 | IRF6 |
| 295 | PRKCDBP |
| 296 | RHOQ |
| 297 | KRT33B |
| 298 | ZNRD1 |
| 299 | CTXN1 |
| 300 | STAT5B |
| 301 | SKOR1 |
| 302 | NGRN |
| 303 | MMD |
| 304 | MON1A |
| 305 | SNX1 |
| 306 | VSIG8 |
| 307 | DDX46 |
| 308 | HOXB5 |
| 309 | ARMC9 |
| 310 | MRPS31 |
| 311 | LHX6 |
| 312 | USP3 |
| 313 | ACBD3 |
| 314 | CNTD2 |
| 315 | ZFHX3 |
| 316 | LOC100288147 |
| 317 | KLF11 |
| 318 | CLPP |
| 319 | CA9 |
| 320 | ELK3 |
| 321 | NDNF |
| 322 | LOC100287347 |
| 323 | APPL1 |
| 324 | FLT3 |
| 325 | SLC26A2 |
| 326 | MAGOH |
| 327 | KHDRBS2 |
| 328 | ZNF845 |
| 329 | HIST1H1D |
| 330 | DGAT2 |
| 331 | GNS |
| 332 | PPFIBP1 |
| 333 | DHPS |
| 334 | KRT18P55 |
| 335 | RELN |
| 336 | NXPH4 |
| 337 | FLJ31485 |
| 338 | SWAP70 |
| 339 | MIPOL1 |
| 340 | RMND5A |
| 341 | NPAS2 |
| 342 | TLN2 |
| 343 | KRT19 |
| 344 | LRRC73 |
| 345 | DACT3 |
| 346 | LRFN4 |
| 347 | SIPA1L2 |
| 348 | TLR9 |
| 349 | SYNE4 |
| 350 | LINC00526 |
| 351 | COL8A2 |
| 352 | IP6K2 |
| 353 | LOC202781 |
| 354 | CGNL1 |
| 355 | CIRH1A |
| 356 | BUB1B |
| 357 | EEF1DP3 |
| 358 | LOC440084 |
| 359 | TEX2 |
| 360 | CASP12 |
| 361 | ELP4 |
| 362 | ZUFSP |
| 363 | PLK5 |
| 364 | ZNF100 |
| 365 | STK10 |
| 366 | GRIN2B |
| 367 | ELFN2 |
| 368 | LOC646808 |
| 369 | JMJD1C |
| 370 | HOXD3 |
| 371 | NCRNA00185 |
| 372 | FGL2 |
| 373 | RPS3A |
| 374 | ZNF544 |
| 375 | GABRA4 |
| 376 | ST13P18 |
| 377 | CRTAM |
| 378 | LRRC8C |
| 379 | OR10K1 |
| 380 | COPS2 |
| 381 | CHST1 |
| 382 | NRGN |
| 383 | RAB27A |
| 384 | SNRNP48 |
| 385 | EIF4A1P4 |
| 386 | PDLIM2 |
| 387 | SLC26A10 |
| 388 | SOD1 |
| 389 | CALD1 |
| 390 | SCARNA17 |
| 391 | CNOT2 |
| 392 | STK32A |
| 393 | KCTD17 |
| 394 | FOXS1 |
| 395 | RNF115 |
| 396 | GDAP2 |
| 397 | AIM2 |
| 398 | SEL1L3 |
| 399 | IMMP1L |
| 400 | CNO |
| 401 | EPS8L2 |
| 402 | KCNQ3 |
| 403 | C1orf150 |
| 404 | ARFGEF2 |
| 405 | PRICKLE2 |
| 406 | C1orf162 |
| 407 | KCNMB4 |
| 408 | TGFBR1 |
| 409 | LTA4H |
| 410 | BEND3 |
| 411 | LIMCH1 |
| 412 | ZC3H6 |
| 413 | LONRF3 |
| 414 | LEPREL1 |
| 415 | POU3F2 |
| 416 | POLK |
| 417 | EPC1 |
| 418 | COBLL1 |
| 419 | PDE2A |
| 420 | H3.Y |
| 421 | QRICH1 |
| 422 | MYNN |
| 423 | PLCB4 |
| 424 | UBTD2 |
| 425 | CDV3 |
| 426 | CCNY |
| 427 | L3MBTL3 |
| 428 | RHOBTB2 |
| 429 | CHD7 |
| 430 | 1-Mar |
| 431 | NDN |
| 432 | LOC729683 |
| 433 | ZP2 |
| 434 | PANK4 |
| 435 | HIST1H1C |
| 436 | SLC1A6 |
| 437 | LCOR |
| 438 | KRT25 |
| 439 | LOC344595 |
| 440 | RERG |
| 441 | RPGRIP1L |
| 442 | TRIM43 |
| 443 | PAX8 |
| 444 | ABTB1 |
| 445 | LEO1 |
| 446 | CX3CL1 |
| 447 | PAN3 |
| 448 | DEFB1 |
| 449 | TM9SF3 |
| 450 | MPP3 |
| 451 | PELI2 |
| 452 | CAMK4 |
| 453 | ARMC8 |
| 454 | POLE4 |
| 455 | SNHG10 |
| 456 | WDR83 |
| 457 | SYT5 |
| 458 | MRPL24 |
| 459 | C1orf74 |
| 460 | FXR1 |
| 461 | CCK |
| 462 | GPATCH2 |
| 463 | SLC2A13 |
| 464 | RPL18AP3 |
| 465 | RBMS3 |
| 466 | CLCNKB |
| 467 | PCDHA8 |
| 468 | LOC400043 |
| 469 | ZDHHC2 |
| 470 | FAM213A |
| 471 | PPIH |
| 472 | TCF19 |
| 473 | ARHGEF4 |
| 474 | PIAS2 |
| 475 | SLC26A5 |
| 476 | RPL17 |
| 477 | GLUD2 |
| 478 | PDCD6IP |
| 479 | SNHG1 |
| 480 | COQ10A |
| 481 | FAM63B |
| 482 | DOCK11 |
| 483 | ZNF442 |
| 484 | CPNE4 |
| 485 | COL27A1 |
| 486 | ZBED1 |
| 487 | ATF7IP |
| 488 | PRPF38B |
| 489 | KIAA0664L3 |
| 490 | OR14I1 |
| 491 | LOC100287005 |
| 492 | C11orf46 |
| 493 | SETDB1 |
| 494 | TTC37 |
| 495 | RNF146 |
| 496 | ZNF232 |
| 497 | C1QL2 |
| 498 | UNCX |
| 499 | WDR78 |
| 500 | LOC340947 |

1. **H0351.2001**

**(I) MaxRel feature list**

| Order | Name |
| --- | --- |
| 1 | NR2E1 |
| 2 | DAO |
| 3 | LRRC7 |
| 4 | GABRA5 |
| 5 | STON1 |
| 6 | CPNE4 |
| 7 | FLJ42875 |
| 8 | OTX1 |
| 9 | PLK2 |
| 10 | RGS20 |
| 11 | CAMK4 |
| 12 | EMX2 |
| 13 | NRGN |
| 14 | KALRN |
| 15 | MEIS1 |
| 16 | MX1 |
| 17 | CTNNA2 |
| 18 | GRIN2B |
| 19 | CAMKV |
| 20 | PLCB1 |
| 21 | CCNG2 |
| 22 | LHX2 |
| 23 | IRX2 |
| 24 | KHDRBS2 |
| 25 | SYNE4 |
| 26 | SEL1L3 |
| 27 | ADAMTS19 |
| 28 | ABR |
| 29 | PDE2A |
| 30 | THRB |
| 31 | AK5 |
| 32 | LMO7 |
| 33 | LDB2 |
| 34 | LHX6 |
| 35 | CDKL5 |
| 36 | CAMK2A |
| 37 | PAK7 |
| 38 | LINC00599 |
| 39 | RASL10A |
| 40 | KCNF1 |
| 41 | LOC283174 |
| 42 | MOXD1 |
| 43 | LINC00473 |
| 44 | SLC26A10 |
| 45 | MCTP1 |
| 46 | LRRTM1 |
| 47 | ATP2B1 |
| 48 | FAM49A |
| 49 | EMX2OS |
| 50 | NETO1 |
| 51 | DLX6-AS1 |
| 52 | RAPGEF4 |
| 53 | FLRT2 |
| 54 | HOMER1 |
| 55 | GRM5 |
| 56 | FOXG1 |
| 57 | WIF1 |
| 58 | NPTX2 |
| 59 | CREG2 |
| 60 | EPHA4 |
| 61 | GDA |
| 62 | MKL2 |
| 63 | C14orf23 |
| 64 | FAM81A |
| 65 | STX1A |
| 66 | MMD |
| 67 | SYT16 |
| 68 | FHL2 |
| 69 | PRICKLE2 |
| 70 | TIAM2 |
| 71 | AKAP5 |
| 72 | CRNDE |
| 73 | GABRA4 |
| 74 | GPR98 |
| 75 | NEUROD1 |
| 76 | ICAM5 |
| 77 | KLHL3 |
| 78 | ETV1 |
| 79 | C4orf50 |
| 80 | NRG3 |
| 81 | CDH9 |
| 82 | LHX1 |
| 83 | CAMK1 |
| 84 | TMEM132B |
| 85 | RGS4 |
| 86 | SLCO1C1 |
| 87 | CHSY3 |
| 88 | PTPRR |
| 89 | RCAN3 |
| 90 | ZNF521 |
| 91 | WASF1 |
| 92 | DUSP5 |
| 93 | DPF1 |
| 94 | CHRM3 |
| 95 | CMTM7 |
| 96 | CECR6 |
| 97 | GRIK2 |
| 98 | STOX1 |
| 99 | KIF17 |
| 100 | FBXL16 |
| 101 | ITPKA |
| 102 | GABRA2 |
| 103 | MAL2 |
| 104 | ZIC4 |
| 105 | GABRB3 |
| 106 | SYT5 |
| 107 | DLGAP1 |
| 108 | FEZF2 |
| 109 | PRKCG |
| 110 | CCK |
| 111 | LOC646627 |
| 112 | LOC729722 |
| 113 | RORB |
| 114 | LOC344595 |
| 115 | LDLRAP1 |
| 116 | NEUROD6 |
| 117 | PIRT |
| 118 | DCAF6 |
| 119 | RANBP3L |
| 120 | DYSF |
| 121 | KCNB1 |
| 122 | NCALD |
| 123 | 5-Sep |
| 124 | SAMD4A |
| 125 | FRMPD4 |
| 126 | PCP2 |
| 127 | ACTR3B |
| 128 | DUSP6 |
| 129 | SCN3B |
| 130 | CLDN10 |
| 131 | NGEF |
| 132 | RGS7BP |
| 133 | OSBPL3 |
| 134 | MPP3 |
| 135 | RIMS1 |
| 136 | KIAA1456 |
| 137 | NELF |
| 138 | LINGO1 |
| 139 | PHACTR1 |
| 140 | ARHGAP32 |
| 141 | KCNMA1 |
| 142 | DLGAP4 |
| 143 | C1orf115 |
| 144 | CACNB4 |
| 145 | EGR3 |
| 146 | CAMK1D |
| 147 | NIM1 |
| 148 | ENC1 |
| 149 | RGS14 |
| 150 | C2orf55 |
| 151 | PRKCB |
| 152 | FAM5B |
| 153 | CAMK2N1 |
| 154 | KCTD16 |
| 155 | MICAL2 |
| 156 | KIAA1024 |
| 157 | HN1L |
| 158 | FILIP1 |
| 159 | PCDH20 |
| 160 | ZDHHC23 |
| 161 | CDC40 |
| 162 | FAM171B |
| 163 | ZNF831 |
| 164 | ABRACL |
| 165 | CYP46A1 |
| 166 | SYNGAP1 |
| 167 | DLGAP2 |
| 168 | NEK10 |
| 169 | BSN |
| 170 | LMO3 |
| 171 | PDLIM2 |
| 172 | SH3RF3 |
| 173 | CABP1 |
| 174 | SGK223 |
| 175 | CX3CL1 |
| 176 | PSD |
| 177 | CAMKK2 |
| 178 | OR14I1 |
| 179 | CACNG3 |
| 180 | CDH22 |
| 181 | DLX1 |
| 182 | ACTN1 |
| 183 | CCDC3 |
| 184 | GNPTAB |
| 185 | WSCD2 |
| 186 | LRRC39 |
| 187 | C5orf38 |
| 188 | JAKMIP1 |
| 189 | DRP2 |
| 190 | CADPS2 |
| 191 | ANKS1B |
| 192 | HRH1 |
| 193 | CSGALNACT1 |
| 194 | SPACA3 |
| 195 | HOXB2 |
| 196 | ARX |
| 197 | KLK10 |
| 198 | TAC3 |
| 199 | ANKRD30BP2 |
| 200 | SCN2A |
| 201 | ARNTL2 |
| 202 | GRASP |
| 203 | LPPR5 |
| 204 | BZW2 |
| 205 | SH3RF1 |
| 206 | LAMB1 |
| 207 | GRIN2A |
| 208 | ANKRD30BL |
| 209 | CRH |
| 210 | GABRD |
| 211 | CAP2 |
| 212 | LOC440084 |
| 213 | PCDHGC5 |
| 214 | ITPR1 |
| 215 | KCNV1 |
| 216 | SYNE1 |
| 217 | SLC6A7 |
| 218 | IRX3 |
| 219 | GALNTL5 |
| 220 | DAPK1 |
| 221 | TBC1D26 |
| 222 | HOXA2 |
| 223 | RBFOX3 |
| 224 | EPHB6 |
| 225 | NAGPA |
| 226 | GPRIN2 |
| 227 | SRPX |
| 228 | EBF1 |
| 229 | ARNT2 |
| 230 | FAR2 |
| 231 | LRRTM4 |
| 232 | CHN1 |
| 233 | BACE2 |
| 234 | NNT |
| 235 | ANK1 |
| 236 | FSTL5 |
| 237 | PPP2R2C |
| 238 | KCND3 |
| 239 | SIRPA |
| 240 | CD44 |
| 241 | LOC729683 |
| 242 | LZTS1 |
| 243 | ODZ2 |
| 244 | NCS1 |
| 245 | FLT3 |
| 246 | HOXD3 |
| 247 | FAT3 |
| 248 | AQP1 |
| 249 | BAI2 |
| 250 | MAST3 |
| 251 | FBXO7 |
| 252 | ATRNL1 |
| 253 | GABBR2 |
| 254 | DHRS13 |
| 255 | ATXN1 |
| 256 | DPYSL4 |
| 257 | IDS |
| 258 | HS6ST3 |
| 259 | MCAM |
| 260 | RASGRF2 |
| 261 | GLCE |
| 262 | SLC27A6 |
| 263 | SLIT1 |
| 264 | PTPRO |
| 265 | R3HDM1 |
| 266 | CACNB1 |
| 267 | CHRDL1 |
| 268 | SNX32 |
| 269 | PPFIA4 |
| 270 | C11orf41 |
| 271 | EPB49 |
| 272 | SLC39A10 |
| 273 | PLCB4 |
| 274 | NRXN2 |
| 275 | PNMA2 |
| 276 | KANK4 |
| 277 | RAPGEFL1 |
| 278 | RNF152 |
| 279 | HTR4 |
| 280 | BIRC3 |
| 281 | FAM107A |
| 282 | DUSP19 |
| 283 | SNHG1 |
| 284 | BCL11B |
| 285 | LOC730091 |
| 286 | CFL2 |
| 287 | LSS |
| 288 | LRRC6 |
| 289 | C3orf80 |
| 290 | SATB2 |
| 291 | CIT |
| 292 | RASAL1 |
| 293 | EPS8L2 |
| 294 | NTN1 |
| 295 | LOC100133686 |
| 296 | INADL |
| 297 | PAK6 |
| 298 | HOXB3 |
| 299 | KCNIP3 |
| 300 | CSMD1 |
| 301 | LOC400043 |
| 302 | S100A6 |
| 303 | SYT7 |
| 304 | GPM6A |
| 305 | XAF1 |
| 306 | TESPA1 |
| 307 | CCDC160 |
| 308 | CHL1 |
| 309 | SLC35F1 |
| 310 | GUK1 |
| 311 | PRMT8 |
| 312 | SRRM4 |
| 313 | RXFP1 |
| 314 | HLA-DPB1 |
| 315 | CLK4 |
| 316 | RELL1 |
| 317 | STK32C |
| 318 | TNFRSF12A |
| 319 | ACLY |
| 320 | CPXM2 |
| 321 | HRK |
| 322 | DIRAS2 |
| 323 | CXCL14 |
| 324 | PSD3 |
| 325 | JPH3 |
| 326 | HTR2A |
| 327 | BTN2A2 |
| 328 | SCAI |
| 329 | GABRB2 |
| 330 | PITPNM2 |
| 331 | NPAS2 |
| 332 | GLT8D2 |
| 333 | PDP1 |
| 334 | PAX3 |
| 335 | SHC3 |
| 336 | CGN |
| 337 | KIF3B |
| 338 | TSPAN8 |
| 339 | HOOK1 |
| 340 | LPPR4 |
| 341 | LNX1 |
| 342 | SERPINF1 |
| 343 | ALS2 |
| 344 | RMI1 |
| 345 | RND2 |
| 346 | FKBP9 |
| 347 | ARHGAP24 |
| 348 | MEF2C |
| 349 | ABI2 |
| 350 | PCDH8 |
| 351 | GRIA2 |
| 352 | CEP76 |
| 353 | SLC26A4 |
| 354 | LRTM2 |
| 355 | LOXL2 |
| 356 | CAMK2B |
| 357 | TMEM155 |
| 358 | SPINK6 |
| 359 | COL24A1 |
| 360 | RTKN2 |
| 361 | CERS2 |
| 362 | FAM212B |
| 363 | ST6GAL2 |
| 364 | SPARCL1 |
| 365 | ADCY2 |
| 366 | LRRC8B |
| 367 | SLC1A2 |
| 368 | LOC158696 |
| 369 | TF |
| 370 | CPNE5 |
| 371 | CHD7 |
| 372 | CHRD |
| 373 | GAS2 |
| 374 | UNC13A |
| 375 | PRDM16 |
| 376 | CNKSR2 |
| 377 | GLTPD2 |
| 378 | CBLB |
| 379 | C16orf87 |
| 380 | ANKRD30B |
| 381 | RFPL1-AS1 |
| 382 | WNT11 |
| 383 | WBSCR17 |
| 384 | CBLN1 |
| 385 | ZNF702P |
| 386 | PTPN3 |
| 387 | NUDT4P1 |
| 388 | LPPR3 |
| 389 | LOC646043 |
| 390 | OPCML |
| 391 | ANGPTL2 |
| 392 | RASAL2 |
| 393 | RSPO4 |
| 394 | RBP4 |
| 395 | TBR1 |
| 396 | ST6GALNAC5 |
| 397 | LOC151009 |
| 398 | FAM153A |
| 399 | SYNPR |
| 400 | RGR |
| 401 | CLCN5 |
| 402 | TIMP2 |
| 403 | DGKI |
| 404 | PCSK1 |
| 405 | PLK5 |
| 406 | MEF2A |
| 407 | DIP2B |
| 408 | ST8SIA3 |
| 409 | KCNH3 |
| 410 | DGKB |
| 411 | CCDC103 |
| 412 | RPRML |
| 413 | EFCAB1 |
| 414 | PPA1 |
| 415 | C17orf96 |
| 416 | ASTN1 |
| 417 | LRRTM2 |
| 418 | LOC100131342 |
| 419 | VSTM2A |
| 420 | YPEL1 |
| 421 | SLC30A3 |
| 422 | CCND2 |
| 423 | PDZD4 |
| 424 | NCAN |
| 425 | VIP |
| 426 | PPFIA2 |
| 427 | SEMA5B |
| 428 | SNTG1 |
| 429 | CACNA2D3 |
| 430 | C8orf46 |
| 431 | SLC20A2 |
| 432 | LRFN2 |
| 433 | C2orf80 |
| 434 | PCDH11Y |
| 435 | B3GALT2 |
| 436 | FAM153B |
| 437 | FAM70A |
| 438 | VWA5A |
| 439 | LCE3C |
| 440 | RFFL |
| 441 | KCNA4 |
| 442 | LURAP1L |
| 443 | ANO3 |
| 444 | MFSD4 |
| 445 | C1orf95 |
| 446 | VAMP1 |
| 447 | FOXP2 |
| 448 | NCR2 |
| 449 | KCTD1 |
| 450 | KCNK2 |
| 451 | NTRK2 |
| 452 | RHOQ |
| 453 | NPY1R |
| 454 | GRIA3 |
| 455 | CELF1 |
| 456 | LOC100287347 |
| 457 | CDH23 |
| 458 | INSIG1 |
| 459 | SLC26A4-AS1 |
| 460 | SMPD3 |
| 461 | TTC39A |
| 462 | LEPREL2 |
| 463 | SLC1A6 |
| 464 | KIAA1045 |
| 465 | ZNF646 |
| 466 | SYDE2 |
| 467 | CA11 |
| 468 | STK33 |
| 469 | ATL2 |
| 470 | GPR26 |
| 471 | CSRNP3 |
| 472 | GALNT12 |
| 473 | NUPR1 |
| 474 | ZNF238 |
| 475 | AGBL4 |
| 476 | DLGAP3 |
| 477 | SVIP |
| 478 | PRICKLE1 |
| 479 | KLHL2 |
| 480 | HPCA |
| 481 | FKBP1A |
| 482 | WDR16 |
| 483 | ZBTB16 |
| 484 | KCNQ5 |
| 485 | OAF |
| 486 | ISOC1 |
| 487 | KIAA0355 |
| 488 | ERICH1-AS1 |
| 489 | CDK5R1 |
| 490 | TJP2 |
| 491 | MYBPHL |
| 492 | DGKA |
| 493 | ZNF184 |
| 494 | TIMP4 |
| 495 | CLPSL2 |
| 496 | EGR1 |
| 497 | NPC2 |
| 498 | PBX3 |
| 499 | AVEN |
| 500 | SLC8A2 |

**(II) mRMR feature list**

| Order | Name |
| --- | --- |
| 1 | NR2E1 |
| 2 | KLK8 |
| 3 | DAO |
| 4 | HOXA2 |
| 5 | LRRC7 |
| 6 | PCP2 |
| 7 | OTX1 |
| 8 | EMX2 |
| 9 | STON1 |
| 10 | HOXB2 |
| 11 | SPINK6 |
| 12 | EMX2OS |
| 13 | RASL10A |
| 14 | MEIS1 |
| 15 | MAB21L2 |
| 16 | ADAMTS19 |
| 17 | NIM1 |
| 18 | DUSP5 |
| 19 | HOXB3 |
| 20 | FLJ42875 |
| 21 | NNT |
| 22 | CCNG2 |
| 23 | STOX1 |
| 24 | S100A6 |
| 25 | GABRA5 |
| 26 | HOXA3 |
| 27 | PPP2R2C |
| 28 | PAX3 |
| 29 | CTNNA2 |
| 30 | HOXA4 |
| 31 | RGS20 |
| 32 | PPA1 |
| 33 | TFAP2B |
| 34 | MX1 |
| 35 | ETV1 |
| 36 | HOXD3 |
| 37 | KALRN |
| 38 | MTF2 |
| 39 | LHX1 |
| 40 | ARX |
| 41 | RANBP3L |
| 42 | MEF2A |
| 43 | ZNF503 |
| 44 | LOC400043 |
| 45 | PLCB1 |
| 46 | PDLIM2 |
| 47 | DPYSL4 |
| 48 | LOC344595 |
| 49 | PRDM16 |
| 50 | ANKS1B |
| 51 | SEL1L3 |
| 52 | ABR |
| 53 | MPP3 |
| 54 | CMTM7 |
| 55 | KLHL3 |
| 56 | HOXA7 |
| 57 | SAMD4A |
| 58 | PIF1 |
| 59 | KHDRBS2 |
| 60 | FAT3 |
| 61 | SPACA3 |
| 62 | ANK1 |
| 63 | CDKL5 |
| 64 | SKOR1 |
| 65 | LRRC39 |
| 66 | NRXN2 |
| 67 | FAM107A |
| 68 | LDLRAP1 |
| 69 | KLHDC7A |
| 70 | KLK10 |
| 71 | RAPGEF4 |
| 72 | THUMPD2 |
| 73 | IL1RAP |
| 74 | KIF3B |
| 75 | CPNE4 |
| 76 | PAX2 |
| 77 | TOP2B |
| 78 | ATP2B1 |
| 79 | KCNK2 |
| 80 | LSS |
| 81 | CEP76 |
| 82 | ST3GAL6 |
| 83 | CLDN10 |
| 84 | KIAA1024 |
| 85 | KIAA1456 |
| 86 | HOXB5 |
| 87 | CAMK4 |
| 88 | LURAP1L |
| 89 | CLCN5 |
| 90 | FKBP3 |
| 91 | SIRPA |
| 92 | LRRTM1 |
| 93 | CBLB |
| 94 | CGB5 |
| 95 | FLT3 |
| 96 | HOXB4 |
| 97 | LOC729722 |
| 98 | GLRA1 |
| 99 | NRG3 |
| 100 | NYNRIN |
| 101 | IRX2 |
| 102 | SOD1 |
| 103 | SLC6A4 |
| 104 | FAM49A |
| 105 | LOXL2 |
| 106 | TNFRSF12A |
| 107 | ZNF521 |
| 108 | UG0898H09 |
| 109 | DLGAP4 |
| 110 | RPS21 |
| 111 | SYNE1 |
| 112 | R3HDM4 |
| 113 | CARKD |
| 114 | CAMKV |
| 115 | INSIG1 |
| 116 | GALNT12 |
| 117 | MOXD1 |
| 118 | CTNND2 |
| 119 | CACNB4 |
| 120 | DUSP6 |
| 121 | THRSP |
| 122 | HOXA5 |
| 123 | ASTN1 |
| 124 | SLC26A10 |
| 125 | DHRS13 |
| 126 | NTRK2 |
| 127 | HIST1H2AE |
| 128 | OSBPL3 |
| 129 | NELF |
| 130 | GRIK2 |
| 131 | ASCL2 |
| 132 | COL8A2 |
| 133 | SYNE4 |
| 134 | FAM70A |
| 135 | NCAN |
| 136 | KBTBD12 |
| 137 | FKBP9 |
| 138 | PAX8 |
| 139 | GRM5 |
| 140 | ANKRD30BL |
| 141 | PCDHB8 |
| 142 | SLC25A18 |
| 143 | THRB |
| 144 | LOC100288147 |
| 145 | NTPCR |
| 146 | SNHG1 |
| 147 | SLC20A2 |
| 148 | LOC646043 |
| 149 | MAPK1 |
| 150 | SLCO1C1 |
| 151 | ACLY |
| 152 | KCND3 |
| 153 | PDP1 |
| 154 | ZKSCAN4 |
| 155 | NPTX2 |
| 156 | ACTR3BP2 |
| 157 | GNG13 |
| 158 | SGK223 |
| 159 | PRKCDBP |
| 160 | CLK4 |
| 161 | ACAP3 |
| 162 | C16orf87 |
| 163 | RALGAPA2 |
| 164 | LBH |
| 165 | DDAH1 |
| 166 | FRMPD4 |
| 167 | ABLIM1 |
| 168 | WDR26 |
| 169 | CELF1 |
| 170 | CBLN1 |
| 171 | KIAA0355 |
| 172 | PLCH2 |
| 173 | FAM101A |
| 174 | GUK1 |
| 175 | SLC1A2 |
| 176 | GRID2 |
| 177 | AVEN |
| 178 | PRODH |
| 179 | CCDC103 |
| 180 | ATXN1 |
| 181 | LOC283174 |
| 182 | NCAPG |
| 183 | GRIN2B |
| 184 | ATRX |
| 185 | SVIP |
| 186 | PPM1B |
| 187 | LINC00473 |
| 188 | MT3 |
| 189 | DLGAP3 |
| 190 | IL28RA |
| 191 | NCR2 |
| 192 | PAPLN |
| 193 | EBF3 |
| 194 | FAM199X |
| 195 | GNPTAB |
| 196 | FOXP2 |
| 197 | NCS1 |
| 198 | PDZK1 |
| 199 | EPHA4 |
| 200 | HRASLS |
| 201 | TLL2 |
| 202 | GOLGA8A |
| 203 | EIF3E |
| 204 | CYP46A1 |
| 205 | GJA8 |
| 206 | SLC35F4 |
| 207 | NEK10 |
| 208 | SYT7 |
| 209 | SH3RF1 |
| 210 | HOXC6 |
| 211 | DLX6-AS1 |
| 212 | SOX5 |
| 213 | B4GALT1 |
| 214 | RGS7BP |
| 215 | VAMP1 |
| 216 | FAM149A |
| 217 | F5 |
| 218 | ALS2 |
| 219 | PLK2 |
| 220 | TSPAN1 |
| 221 | NTN1 |
| 222 | EPM2AIP1 |
| 223 | NMUR1 |
| 224 | FOXG1 |
| 225 | ANP32A |
| 226 | LRRC6 |
| 227 | CALCRL |
| 228 | FAM171B |
| 229 | ILDR2 |
| 230 | TRIM67 |
| 231 | LOC400657 |
| 232 | LPCAT2 |
| 233 | CAMK2N1 |
| 234 | GCHFR |
| 235 | HN1L |
| 236 | HNRNPD |
| 237 | LMO7 |
| 238 | SYCP2L |
| 239 | LOC654433 |
| 240 | SYT2 |
| 241 | PLK5 |
| 242 | SHROOM1 |
| 243 | FSTL5 |
| 244 | ANGPT1 |
| 245 | NOVA2 |
| 246 | MCTP1 |
| 247 | OXTR |
| 248 | RGR |
| 249 | PPFIA2 |
| 250 | KANK4 |
| 251 | ABLIM3 |
| 252 | SPPL2C |
| 253 | GABRA4 |
| 254 | CD70 |
| 255 | KCNF1 |
| 256 | C10orf107 |
| 257 | TF |
| 258 | NOVA1 |
| 259 | EBF1 |
| 260 | HSD17B6 |
| 261 | SPTLC2 |
| 262 | FAR2 |
| 263 | PCSK9 |
| 264 | HLA-DPB1 |
| 265 | GABRA2 |
| 266 | MAPKBP1 |
| 267 | C14orf105 |
| 268 | LDB2 |
| 269 | RNF133 |
| 270 | NRG1 |
| 271 | NTM |
| 272 | LHX2 |
| 273 | FAM160B2 |
| 274 | NXPH4 |
| 275 | ODZ2 |
| 276 | NLRP11 |
| 277 | NRGN |
| 278 | CGN |
| 279 | RHBG |
| 280 | SDCCAG8 |
| 281 | MMD |
| 282 | SRGAP2C |
| 283 | MMEL1 |
| 284 | EPB49 |
| 285 | RSPO4 |
| 286 | ANKRD30BP2 |
| 287 | MVK |
| 288 | BZW2 |
| 289 | CA9 |
| 290 | RFFL |
| 291 | TRIM13 |
| 292 | CCDC14 |
| 293 | DCAF6 |
| 294 | ARHGEF4 |
| 295 | PHOX2B |
| 296 | WNT11 |
| 297 | BTG1 |
| 298 | PRKCG |
| 299 | ATAD2 |
| 300 | DGKI |
| 301 | SGCA |
| 302 | TMEM189 |
| 303 | KRT24 |
| 304 | NPAS2 |
| 305 | ARNT2 |
| 306 | MINA |
| 307 | C1orf95 |
| 308 | PCDH10 |
| 309 | PRPH |
| 310 | DYSF |
| 311 | ATP8B3 |
| 312 | INADL |
| 313 | DNAH7 |
| 314 | SC5DL |
| 315 | LHX6 |
| 316 | EMILIN2 |
| 317 | SLC8A1 |
| 318 | TSPO |
| 319 | PID1 |
| 320 | ZNF883 |
| 321 | GABBR2 |
| 322 | PLS3 |
| 323 | NGFR |
| 324 | LINC00599 |
| 325 | TNIK |
| 326 | ZNF536 |
| 327 | TIAM2 |
| 328 | CNOT2 |
| 329 | SQLE |
| 330 | CDR2L |
| 331 | CX3CL1 |
| 332 | PACRGL |
| 333 | TLX3 |
| 334 | GABRB2 |
| 335 | NCALD |
| 336 | LIG1 |
| 337 | CRTAM |
| 338 | CHRM4 |
| 339 | GABRB3 |
| 340 | ACTC1 |
| 341 | FLRT2 |
| 342 | ZCCHC16 |
| 343 | LAMB1 |
| 344 | YIPF7 |
| 345 | KLHL1 |
| 346 | FBXO7 |
| 347 | SHISA9 |
| 348 | FXYD7 |
| 349 | IDS |
| 350 | HOXA6 |
| 351 | SVEP1 |
| 352 | TBC1D19 |
| 353 | GPRIN2 |
| 354 | TMC7 |
| 355 | DUSP7 |
| 356 | LPPR3 |
| 357 | CIT |
| 358 | POLR3D |
| 359 | SLC6A7 |
| 360 | ZDHHC2 |
| 361 | ALOX5 |
| 362 | GLTPD2 |
| 363 | DRP2 |
| 364 | ZNF549 |
| 365 | LOC100292648 |
| 366 | GAS2 |
| 367 | CBLN3 |
| 368 | CHL1 |
| 369 | PTPRJ |
| 370 | PRMT8 |
| 371 | BCAT1 |
| 372 | MLEC |
| 373 | PRICKLE2 |
| 374 | FAM43A |
| 375 | MAB21L1 |
| 376 | POMP |
| 377 | DIP2B |
| 378 | RASAL2 |
| 379 | RMI1 |
| 380 | RPH3AL |
| 381 | IFNGR1 |
| 382 | COL6A1 |
| 383 | FAM5B |
| 384 | FAM185A |
| 385 | HOOK1 |
| 386 | GK5 |
| 387 | C14orf23 |
| 388 | KRT18P55 |
| 389 | HS6ST3 |
| 390 | CNPY1 |
| 391 | LOC100133686 |
| 392 | ADORA2B |
| 393 | PAXIP1 |
| 394 | TSPAN8 |
| 395 | BTF3 |
| 396 | PRSS35 |
| 397 | MYCT1 |
| 398 | VAX2 |
| 399 | GPD1 |
| 400 | EEF2K |
| 401 | IFIT5 |
| 402 | MAL2 |
| 403 | PRSS22 |
| 404 | CXCL14 |
| 405 | NECAB2 |
| 406 | NKX6-1 |
| 407 | PIK3R1 |
| 408 | CCDC160 |
| 409 | EMID1 |
| 410 | TAGLN2 |
| 411 | C4orf50 |
| 412 | GABRA6 |
| 413 | LRRTM2 |
| 414 | GRHL3 |
| 415 | KRT18 |
| 416 | GLCE |
| 417 | UFD1L |
| 418 | VWA5A |
| 419 | SUPT3H |
| 420 | RHOQ |
| 421 | SH3RF3 |
| 422 | NRK |
| 423 | RET |
| 424 | LOC730658 |
| 425 | C2CD2L |
| 426 | FGFR1 |
| 427 | TMEM41B |
| 428 | KIAA1522 |
| 429 | SYNPR |
| 430 | CNOT6 |
| 431 | GSTK1 |
| 432 | 5-Sep |
| 433 | SIRPG |
| 434 | EPPK1 |
| 435 | SYNGAP1 |
| 436 | BST2 |
| 437 | LOC100133337 |
| 438 | C17orf110 |
| 439 | CHN2 |
| 440 | GLDC |
| 441 | SNX3 |
| 442 | SRPK1 |
| 443 | CSRNP3 |
| 444 | HRK |
| 445 | GALNT7 |
| 446 | KRTAP5-8 |
| 447 | PDE2A |
| 448 | GPR56 |
| 449 | REXO2 |
| 450 | ZNF17 |
| 451 | STON2 |
| 452 | CAMK1 |
| 453 | RAB11FIP4 |
| 454 | KLF11 |
| 455 | PCDH12 |
| 456 | FGF13 |
| 457 | USP46 |
| 458 | SLC25A27 |
| 459 | FAM81A |
| 460 | HNRNPU-AS1 |
| 461 | SLC27A6 |
| 462 | LOC100288346 |
| 463 | ACTN1 |
| 464 | LRRC37A4P |
| 465 | FNTB |
| 466 | CEP170 |
| 467 | PAK7 |
| 468 | TIGD2 |
| 469 | PPAT |
| 470 | KCNH2 |
| 471 | CPPED1 |
| 472 | CHODL |
| 473 | LRTM2 |
| 474 | ARHGAP4 |
| 475 | AK5 |
| 476 | DIS3 |
| 477 | MGC39372 |
| 478 | RNF152 |
| 479 | OPCML |
| 480 | SNHG7 |
| 481 | PLA2R1 |
| 482 | VSTM2B |
| 483 | PARD6G-AS1 |
| 484 | PTPRN2 |
| 485 | TKT |
| 486 | C5orf38 |
| 487 | TSC1 |
| 488 | ESYT1 |
| 489 | SPARCL1 |
| 490 | C1orf150 |
| 491 | WIF1 |
| 492 | MPP4 |
| 493 | L2HGDH |
| 494 | ACTR3B |
| 495 | CBFA2T3 |
| 496 | TMEM61 |
| 497 | GORASP1 |
| 498 | PILRB |
| 499 | PYCARD |
| 500 | PPP1R9B |

1. **H0351.2002**

**(I) MaxRel feature list**

| Order | Name |
| --- | --- |
| 1 | LRRC7 |
| 2 | DAO |
| 3 | NR2E1 |
| 4 | NRGN |
| 5 | GABRA5 |
| 6 | PLK2 |
| 7 | CAMKV |
| 8 | SYNE4 |
| 9 | LHX6 |
| 10 | RHOQ |
| 11 | IRX2 |
| 12 | CCK |
| 13 | FAM5B |
| 14 | SLC26A10 |
| 15 | FLJ42875 |
| 16 | SLC8A2 |
| 17 | CPNE4 |
| 18 | RASAL1 |
| 19 | CCNG2 |
| 20 | ZNF521 |
| 21 | ETV1 |
| 22 | MEIS1 |
| 23 | RBFOX3 |
| 24 | STOX1 |
| 25 | CREG2 |
| 26 | STON1 |
| 27 | KALRN |
| 28 | MMD |
| 29 | KCTD16 |
| 30 | ATP2B1 |
| 31 | AK5 |
| 32 | GRIN2B |
| 33 | KCNF1 |
| 34 | PTK2B |
| 35 | ITPKA |
| 36 | ABTB1 |
| 37 | RGS14 |
| 38 | CAMK4 |
| 39 | KIF21A |
| 40 | DLGAP2 |
| 41 | FAM81A |
| 42 | FLRT2 |
| 43 | FAM49A |
| 44 | NUP85 |
| 45 | CAMKK2 |
| 46 | RCAN3 |
| 47 | CTNNA2 |
| 48 | KHDRBS2 |
| 49 | PRRT2 |
| 50 | MICAL2 |
| 51 | CAMK2A |
| 52 | LDB2 |
| 53 | LHX1 |
| 54 | AMT |
| 55 | KIF17 |
| 56 | B9D1 |
| 57 | ITPR1 |
| 58 | EBF1 |
| 59 | RASL10A |
| 60 | BCAT1 |
| 61 | CLIP1 |
| 62 | MX1 |
| 63 | ZFAND4 |
| 64 | NECAB1 |
| 65 | CELF1 |
| 66 | ABR |
| 67 | LINC00473 |
| 68 | RGS4 |
| 69 | PDE8B |
| 70 | MOXD1 |
| 71 | DLX1 |
| 72 | ATRNL1 |
| 73 | RAB26 |
| 74 | KIAA1456 |
| 75 | CAST |
| 76 | RNF152 |
| 77 | NETO1 |
| 78 | PDE2A |
| 79 | FILIP1 |
| 80 | PDK2 |
| 81 | CHRM3 |
| 82 | BARHL1 |
| 83 | FOXA2 |
| 84 | GALNT9 |
| 85 | ITM2C |
| 86 | HINFP |
| 87 | ARSJ |
| 88 | PLCB1 |
| 89 | LOC100287347 |
| 90 | EMX2 |
| 91 | RGS20 |
| 92 | MAPKBP1 |
| 93 | SYT5 |
| 94 | SYT16 |
| 95 | LHX2 |
| 96 | RBMS3 |
| 97 | CBLN1 |
| 98 | MAST3 |
| 99 | AKAP5 |
| 100 | LOC283174 |
| 101 | RBM24 |
| 102 | ADAM11 |
| 103 | NIPA1 |
| 104 | LMO3 |
| 105 | RNF112 |
| 106 | SEL1L3 |
| 107 | MKL2 |
| 108 | SLCO1C1 |
| 109 | PCDH20 |
| 110 | ABRACL |
| 111 | LRRC8B |
| 112 | CHN1 |
| 113 | RRAGD |
| 114 | NRG3 |
| 115 | FGFR1 |
| 116 | FLT3 |
| 117 | CPT1A |
| 118 | GDA |
| 119 | C15orf27 |
| 120 | LZTS1 |
| 121 | ANKS1B |
| 122 | NCALD |
| 123 | TNFRSF25 |
| 124 | NEUROD1 |
| 125 | FOXG1 |
| 126 | CAP2 |
| 127 | C2orf55 |
| 128 | CDKL5 |
| 129 | SGK223 |
| 130 | CHRD |
| 131 | CHST1 |
| 132 | RASAL2 |
| 133 | FGF14 |
| 134 | GABRA4 |
| 135 | LINC00599 |
| 136 | SLC1A6 |
| 137 | CHD7 |
| 138 | UNC13A |
| 139 | CAMK2N1 |
| 140 | SH3RF1 |
| 141 | UBASH3B |
| 142 | NELF |
| 143 | TMEM132B |
| 144 | NECAB2 |
| 145 | DLX6-AS1 |
| 146 | MXD4 |
| 147 | PLCB4 |
| 148 | FKBP9L |
| 149 | CGN |
| 150 | KLF13 |
| 151 | ADCK4 |
| 152 | EXPH5 |
| 153 | RRM2B |
| 154 | NEIL1 |
| 155 | FAM153B |
| 156 | TRIM58 |
| 157 | APOLD1 |
| 158 | LPPR4 |
| 159 | ARHGAP4 |
| 160 | TNS1 |
| 161 | LPPR3 |
| 162 | KIAA0895L |
| 163 | INADL |
| 164 | CLK4 |
| 165 | PRICKLE2 |
| 166 | FHL2 |
| 167 | NGEF |
| 168 | KCTD10 |
| 169 | CPPED1 |
| 170 | WSCD2 |
| 171 | CCDC152 |
| 172 | C1orf51 |
| 173 | MAB21L1 |
| 174 | EBF3 |
| 175 | ARHGAP24 |
| 176 | PILRB |
| 177 | FLJ10038 |
| 178 | DDN |
| 179 | ICAM5 |
| 180 | WDR16 |
| 181 | CMTM7 |
| 182 | CNPY1 |
| 183 | ZNF831 |
| 184 | TUBGCP6 |
| 185 | DGKB |
| 186 | SLC25A33 |
| 187 | LOC730173 |
| 188 | KIAA0664L3 |
| 189 | LRRTM4 |
| 190 | BTN2A2 |
| 191 | LOC646627 |
| 192 | KCNV1 |
| 193 | DPF1 |
| 194 | PKIB |
| 195 | CYP46A1 |
| 196 | ACTN1 |
| 197 | LMO7 |
| 198 | C1orf115 |
| 199 | CADPS2 |
| 200 | IMPACT |
| 201 | 10-Sep |
| 202 | HSPB8 |
| 203 | KIAA1024 |
| 204 | TTC39A |
| 205 | CDH9 |
| 206 | TIAM2 |
| 207 | CCNL2 |
| 208 | KLHL1 |
| 209 | ACAP3 |
| 210 | KPNA1 |
| 211 | STX1A |
| 212 | TAC3 |
| 213 | FRAS1 |
| 214 | LOC100290023 |
| 215 | C16orf11 |
| 216 | STK32C |
| 217 | PRKAR2B |
| 218 | ZIC4 |
| 219 | LAT |
| 220 | HTR2A |
| 221 | SIAE |
| 222 | LOC643037 |
| 223 | TPCN2 |
| 224 | GLE1 |
| 225 | C14orf23 |
| 226 | IRX3 |
| 227 | HPSE2 |
| 228 | MAP3K12 |
| 229 | CDV3 |
| 230 | TCF4 |
| 231 | 5-Sep |
| 232 | KGFLP1 |
| 233 | CRB1 |
| 234 | VWA5A |
| 235 | B3GALT2 |
| 236 | DHRS13 |
| 237 | ACHE |
| 238 | PAX6 |
| 239 | SETBP1 |
| 240 | EPHA4 |
| 241 | SIPA1L2 |
| 242 | ARHGEF6 |
| 243 | GSDMB |
| 244 | DPH3P1 |
| 245 | CXCL14 |
| 246 | GAS2 |
| 247 | PAK7 |
| 248 | RPRML |
| 249 | ARHGEF4 |
| 250 | LPCAT2 |
| 251 | NS3BP |
| 252 | SLC35F4 |
| 253 | DGAT2 |
| 254 | CAMK2B |
| 255 | RANBP3L |
| 256 | NPAS2 |
| 257 | DLGAP4 |
| 258 | FLCN |
| 259 | DNAH7 |
| 260 | RFPL1-AS1 |
| 261 | C9orf72 |
| 262 | GRM5 |
| 263 | DLX2 |
| 264 | MLL4 |
| 265 | EPS8L2 |
| 266 | C17orf108 |
| 267 | CACNG3 |
| 268 | RBP4 |
| 269 | USP42 |
| 270 | SHC3 |
| 271 | TPD52L1 |
| 272 | OR14I1 |
| 273 | FEZF2 |
| 274 | NTM |
| 275 | PCDH8 |
| 276 | C11orf41 |
| 277 | RORB |
| 278 | MYBPHL |
| 279 | AVIL |
| 280 | KRT33B |
| 281 | ABLIM3 |
| 282 | SCARB2 |
| 283 | MGARP |
| 284 | ZDHHC23 |
| 285 | LRRC73 |
| 286 | PHACTR1 |
| 287 | TSEN54 |
| 288 | SYDE2 |
| 289 | GABRD |
| 290 | S100A6 |
| 291 | ABCC8 |
| 292 | MCF2 |
| 293 | STH |
| 294 | PSD |
| 295 | ARHGAP29 |
| 296 | USP45 |
| 297 | OAF |
| 298 | NRXN2 |
| 299 | ZBTB8A |
| 300 | ZNF653 |
| 301 | ENC1 |
| 302 | FAT3 |
| 303 | ADAMTS8 |
| 304 | EDIL3 |
| 305 | PSD3 |
| 306 | LOC100131342 |
| 307 | CDC25B |
| 308 | PLXNB2 |
| 309 | SEC31B |
| 310 | ZNF148 |
| 311 | TFAP2B |
| 312 | RAB6A |
| 313 | CRNDE |
| 314 | FBXL16 |
| 315 | C16orf79 |
| 316 | TMEM61 |
| 317 | GLCCI1 |
| 318 | C5orf45 |
| 319 | LUZP2 |
| 320 | SRGAP2 |
| 321 | OTX1 |
| 322 | WIF1 |
| 323 | TRIM50 |
| 324 | TIMP2 |
| 325 | THRB |
| 326 | PPFIA4 |
| 327 | EGR3 |
| 328 | RGS2 |
| 329 | CATSPER2 |
| 330 | SST |
| 331 | NEUROD2 |
| 332 | TIMP4 |
| 333 | ANGPTL2 |
| 334 | RIIAD1 |
| 335 | MIR7-3HG |
| 336 | LDLRAP1 |
| 337 | OLR1 |
| 338 | ATP4A |
| 339 | RIN1 |
| 340 | FKBP9 |
| 341 | MPP3 |
| 342 | FAR2 |
| 343 | PCP2 |
| 344 | MAMDC4 |
| 345 | IPW |
| 346 | SPON2 |
| 347 | KCNJ12 |
| 348 | ABLIM1 |
| 349 | KCND3 |
| 350 | LAMB1 |
| 351 | GLRA1 |
| 352 | CDH8 |
| 353 | AGPAT9 |
| 354 | TXNRD2 |
| 355 | FAP |
| 356 | ANO3 |
| 357 | BZW2 |
| 358 | LRRC6 |
| 359 | SETD1A |
| 360 | KCNC2 |
| 361 | DNMT1 |
| 362 | RBM10 |
| 363 | C2orf80 |
| 364 | PCDHA5 |
| 365 | RNF115 |
| 366 | NTN1 |
| 367 | XAF1 |
| 368 | CHSY3 |
| 369 | NPY |
| 370 | CORT |
| 371 | DGCR5 |
| 372 | CCL27 |
| 373 | C10orf107 |
| 374 | SVIP |
| 375 | NKIRAS2 |
| 376 | CLDN3 |
| 377 | KCNJ16 |
| 378 | BACE2 |
| 379 | GABRA2 |
| 380 | ALS2 |
| 381 | LOC202181 |
| 382 | PAX2 |
| 383 | ODZ2 |
| 384 | CABP1 |
| 385 | C5orf38 |
| 386 | SNX32 |
| 387 | JAKMIP1 |
| 388 | NTN4 |
| 389 | ARNT2 |
| 390 | HOXA3 |
| 391 | TANC2 |
| 392 | PTPRC |
| 393 | MEF2C |
| 394 | ETV2 |
| 395 | STAC |
| 396 | C4orf50 |
| 397 | KCNMB4 |
| 398 | LRTM2 |
| 399 | ZNF551 |
| 400 | C9orf171 |
| 401 | KCNQ5 |
| 402 | KCNMA1 |
| 403 | STRC |
| 404 | CHRDL1 |
| 405 | PKD1 |
| 406 | FAM153A |
| 407 | MATK |
| 408 | SLC27A6 |
| 409 | LOC100288147 |
| 410 | SATB2 |
| 411 | LIMA1 |
| 412 | PLGLB1 |
| 413 | PRKCA |
| 414 | AKAP12 |
| 415 | NIM1 |
| 416 | KCNQ3 |
| 417 | LOC100130691 |
| 418 | NINL |
| 419 | LNX1 |
| 420 | HOXB2 |
| 421 | CALML4 |
| 422 | GALNTL4 |
| 423 | CA11 |
| 424 | SLC1A2 |
| 425 | SLC25A18 |
| 426 | DRD5 |
| 427 | CSGALNACT1 |
| 428 | NAGPA |
| 429 | C2orf63 |
| 430 | MORC2-AS1 |
| 431 | PTPRR |
| 432 | IL13RA1 |
| 433 | PBX3 |
| 434 | HOMER1 |
| 435 | CBLB |
| 436 | PRKCG |
| 437 | CAMK1D |
| 438 | UHMK1 |
| 439 | ZDHHC20 |
| 440 | EPHB6 |
| 441 | DUSP19 |
| 442 | BEND7 |
| 443 | PRORSD1P |
| 444 | BAI2 |
| 445 | PAX3 |
| 446 | MPP5 |
| 447 | GPR26 |
| 448 | HS6ST3 |
| 449 | FYB |
| 450 | ACTC1 |
| 451 | TMEM155 |
| 452 | SPSB3 |
| 453 | NKAIN3 |
| 454 | KRT33A |
| 455 | ISOC1 |
| 456 | CIC |
| 457 | SLITRK1 |
| 458 | USP28 |
| 459 | C8orf46 |
| 460 | TRIM36 |
| 461 | TSTD1 |
| 462 | CTDSPL |
| 463 | GLT8D2 |
| 464 | RSPO4 |
| 465 | SSPO |
| 466 | CPNE5 |
| 467 | SUN1 |
| 468 | GRID2 |
| 469 | MAL2 |
| 470 | RAPGEF4 |
| 471 | TRIM17 |
| 472 | FAM131A |
| 473 | UROS |
| 474 | ST6GALNAC5 |
| 475 | VN1R1 |
| 476 | DLGAP1 |
| 477 | PRKCZ |
| 478 | CECR6 |
| 479 | PVRL3 |
| 480 | PCDH10 |
| 481 | WASF1 |
| 482 | CA8 |
| 483 | LPCAT3 |
| 484 | NPTX2 |
| 485 | SOX5 |
| 486 | NEFL |
| 487 | LINGO1 |
| 488 | MMEL1 |
| 489 | FAT2 |
| 490 | FKBP1A |
| 491 | PPP2R2C |
| 492 | KLHL3 |
| 493 | LEPREL2 |
| 494 | KANK4 |
| 495 | TNFRSF6B |
| 496 | WEE1 |
| 497 | ARX |
| 498 | KCNH3 |
| 499 | ZNRF3 |
| 500 | ZNF266 |

**(II) mRMR feature list**

| Order | Name |
| --- | --- |
| 1 | LRRC7 |
| 2 | WDR48 |
| 3 | NR2E1 |
| 4 | NECAB1 |
| 5 | HOXA3 |
| 6 | DAO |
| 7 | HINFP |
| 8 | STON1 |
| 9 | ITM2C |
| 10 | GLRA1 |
| 11 | EBF1 |
| 12 | STOX1 |
| 13 | ARHGAP4 |
| 14 | CDV3 |
| 15 | RASAL1 |
| 16 | PTK2B |
| 17 | TFAP2B |
| 18 | UBASH3B |
| 19 | FAM5B |
| 20 | ABLIM3 |
| 21 | HOXB2 |
| 22 | IRX2 |
| 23 | CLIP1 |
| 24 | KIF21A |
| 25 | PAX2 |
| 26 | TRIM58 |
| 27 | B9D1 |
| 28 | ETV1 |
| 29 | NTN1 |
| 30 | MEIS1 |
| 31 | MAB21L1 |
| 32 | RHOQ |
| 33 | FLJ42875 |
| 34 | S100A6 |
| 35 | AMT |
| 36 | DLX1 |
| 37 | BARHL1 |
| 38 | KCTD10 |
| 39 | FOXQ1 |
| 40 | LHX1 |
| 41 | SLCO1C1 |
| 42 | FKBP9L |
| 43 | LAT |
| 44 | KALRN |
| 45 | HOXA2 |
| 46 | KHDRBS2 |
| 47 | IPW |
| 48 | CNPY1 |
| 49 | CRB1 |
| 50 | GRIN2B |
| 51 | ABLIM1 |
| 52 | ARX |
| 53 | KRT33B |
| 54 | RGS20 |
| 55 | NUP85 |
| 56 | CAMKV |
| 57 | UBE2D2 |
| 58 | HOXB3 |
| 59 | SLC26A10 |
| 60 | SRGAP2 |
| 61 | ANKS1B |
| 62 | CGN |
| 63 | KCNF1 |
| 64 | ENOX1 |
| 65 | RRAGD |
| 66 | CAST |
| 67 | TSEN54 |
| 68 | EBF3 |
| 69 | USP45 |
| 70 | CDKL5 |
| 71 | MAMDC4 |
| 72 | EMX2 |
| 73 | MAP1LC3B |
| 74 | SLC35F4 |
| 75 | SYNE4 |
| 76 | HOXB5 |
| 77 | GABRA5 |
| 78 | KGFLP1 |
| 79 | DLX2 |
| 80 | PAX3 |
| 81 | FAM70A |
| 82 | OTX1 |
| 83 | FOXA2 |
| 84 | CBLN1 |
| 85 | RASL10A |
| 86 | HABP2 |
| 87 | CLCN3 |
| 88 | NECAB2 |
| 89 | BHLHE23 |
| 90 | ARHGAP29 |
| 91 | ETV2 |
| 92 | RBMS3 |
| 93 | FAT3 |
| 94 | CCNL2 |
| 95 | SCARB2 |
| 96 | NRG1 |
| 97 | RBM24 |
| 98 | FLJ10038 |
| 99 | APOLD1 |
| 100 | LOC100288147 |
| 101 | RIIAD1 |
| 102 | MOXD1 |
| 103 | FGFR1 |
| 104 | LINC00176 |
| 105 | KCTD16 |
| 106 | AR |
| 107 | ACTC1 |
| 108 | RASAL2 |
| 109 | FLT3 |
| 110 | ADCK4 |
| 111 | TNS1 |
| 112 | GLE1 |
| 113 | LHX6 |
| 114 | RRM2B |
| 115 | WWOX |
| 116 | CMTM7 |
| 117 | MMEL1 |
| 118 | SLC25A18 |
| 119 | ZNF521 |
| 120 | TTBK2 |
| 121 | FAM49A |
| 122 | KCNK2 |
| 123 | LTK |
| 124 | RNF152 |
| 125 | DLGAP4 |
| 126 | DDX46 |
| 127 | RANBP3L |
| 128 | NPAS2 |
| 129 | CPT1A |
| 130 | NELF |
| 131 | C10orf107 |
| 132 | SORD |
| 133 | AVIL |
| 134 | DLX6-AS1 |
| 135 | TFAP2A |
| 136 | ARSJ |
| 137 | LOC400043 |
| 138 | NIPA1 |
| 139 | PRPH |
| 140 | KIAA0664L3 |
| 141 | CCNG2 |
| 142 | C9orf72 |
| 143 | IMPA2 |
| 144 | KLK8 |
| 145 | MMD |
| 146 | KANK4 |
| 147 | FAR2 |
| 148 | NDP |
| 149 | PILRB |
| 150 | EPC2 |
| 151 | HOXA4 |
| 152 | C2orf63 |
| 153 | CTNNA2 |
| 154 | ZNF266 |
| 155 | PPP1R3D |
| 156 | NTRK2 |
| 157 | LOC441722 |
| 158 | KRT33A |
| 159 | UROS |
| 160 | MX1 |
| 161 | ZFAND4 |
| 162 | C1orf51 |
| 163 | KLHL1 |
| 164 | PFKFB3 |
| 165 | C17orf108 |
| 166 | CBLB |
| 167 | VAMP1 |
| 168 | DRD5 |
| 169 | CELF1 |
| 170 | PODXL |
| 171 | KIAA1024 |
| 172 | BRAP |
| 173 | LPCAT3 |
| 174 | ANGPTL2 |
| 175 | SIAE |
| 176 | EPS8L2 |
| 177 | LRRC73 |
| 178 | KPNA1 |
| 179 | ONECUT1 |
| 180 | ACAP3 |
| 181 | MED17 |
| 182 | ATP2B1 |
| 183 | NXPH4 |
| 184 | ACSBG1 |
| 185 | RIN1 |
| 186 | LBH |
| 187 | GABRA4 |
| 188 | LMBR1L |
| 189 | PDE8B |
| 190 | HOXD3 |
| 191 | CLK4 |
| 192 | PDK2 |
| 193 | PRDM16 |
| 194 | FAM129B |
| 195 | CHD7 |
| 196 | KRT18 |
| 197 | OSBP |
| 198 | RNF112 |
| 199 | RAPGEF4 |
| 200 | MAP3K12 |
| 201 | C10orf32 |
| 202 | REC8 |
| 203 | LOC727982 |
| 204 | FLRT2 |
| 205 | CCL27 |
| 206 | CAMK2N1 |
| 207 | SNHG8 |
| 208 | MED13L |
| 209 | TIAM2 |
| 210 | SAMD4A |
| 211 | ZBTB6 |
| 212 | FOXG1 |
| 213 | GRID2 |
| 214 | SOD2 |
| 215 | ABTB1 |
| 216 | TNIK |
| 217 | ABRACL |
| 218 | SNRNP48 |
| 219 | FOXS1 |
| 220 | CAB39 |
| 221 | DPF1 |
| 222 | PRCD |
| 223 | PCDH20 |
| 224 | EMX2OS |
| 225 | TRIM50 |
| 226 | TDG |
| 227 | C11orf31 |
| 228 | CHRDL1 |
| 229 | STAC |
| 230 | DNAH7 |
| 231 | TSTD2 |
| 232 | WEE1 |
| 233 | PCSK9 |
| 234 | STK32C |
| 235 | MAPKBP1 |
| 236 | SOX17 |
| 237 | EPB41L1 |
| 238 | NTM |
| 239 | NCAPG |
| 240 | C16orf95 |
| 241 | PRICKLE2 |
| 242 | C11orf58 |
| 243 | KIAA1456 |
| 244 | PLCB1 |
| 245 | PLA2R1 |
| 246 | PIK3CB |
| 247 | LOC100132585 |
| 248 | KIF17 |
| 249 | CSDA |
| 250 | ZNF337 |
| 251 | CCDC152 |
| 252 | WDR66 |
| 253 | ZNF500 |
| 254 | TMEM61 |
| 255 | CPNE4 |
| 256 | ZNF398 |
| 257 | CDC25B |
| 258 | CAP2 |
| 259 | OCIAD2 |
| 260 | REPS1 |
| 261 | KCNMB4 |
| 262 | C2orf72 |
| 263 | PRRT2 |
| 264 | PFN2 |
| 265 | ZNF831 |
| 266 | LOC728543 |
| 267 | CD6 |
| 268 | OLR1 |
| 269 | HSPB8 |
| 270 | TPCN2 |
| 271 | CYP46A1 |
| 272 | NINL |
| 273 | LOC100288972 |
| 274 | TIMP4 |
| 275 | LOC100131342 |
| 276 | SLC27A6 |
| 277 | RGL2 |
| 278 | MKL2 |
| 279 | PSMD12 |
| 280 | POU4F1 |
| 281 | PCP2 |
| 282 | PARP6 |
| 283 | FGF14 |
| 284 | EHD1 |
| 285 | SIRPA |
| 286 | RPS6KA1 |
| 287 | LZTS1 |
| 288 | KLHL3 |
| 289 | TMEM51 |
| 290 | HINT1 |
| 291 | PDGFD |
| 292 | ZNF551 |
| 293 | AKAP5 |
| 294 | LOC388630 |
| 295 | KLF13 |
| 296 | FKBP3 |
| 297 | DGKB |
| 298 | CGNL1 |
| 299 | GSTO2 |
| 300 | ITPKA |
| 301 | CCDC142 |
| 302 | RPH3AL |
| 303 | CHST1 |
| 304 | SLC20A2 |
| 305 | SOX5 |
| 306 | LPPR4 |
| 307 | ANKRD55 |
| 308 | FGF7 |
| 309 | OSGEP |
| 310 | DLGAP2 |
| 311 | POLB |
| 312 | SYT16 |
| 313 | FHIT |
| 314 | STRC |
| 315 | ACHE |
| 316 | HCFC2 |
| 317 | LINC00461 |
| 318 | FRAS1 |
| 319 | NRG3 |
| 320 | EIF5A2 |
| 321 | CAMKK2 |
| 322 | SEL1L3 |
| 323 | ADORA2B |
| 324 | KIAA0930 |
| 325 | CHN1 |
| 326 | UBTF |
| 327 | SGK223 |
| 328 | SERPINH1 |
| 329 | CBS |
| 330 | ASB3 |
| 331 | NKAIN3 |
| 332 | EPHA4 |
| 333 | BCAT1 |
| 334 | ANKRD50 |
| 335 | PRORSD1P |
| 336 | NIM1 |
| 337 | GALNT9 |
| 338 | FLJ41350 |
| 339 | BEND7 |
| 340 | KCNJ16 |
| 341 | ZMPSTE24 |
| 342 | CCK |
| 343 | HSD17B6 |
| 344 | TAF1C |
| 345 | RFPL1-AS1 |
| 346 | COL27A1 |
| 347 | MPP5 |
| 348 | LINC00473 |
| 349 | CLDN3 |
| 350 | NKX6-1 |
| 351 | SPATA5L1 |
| 352 | AGPAT9 |
| 353 | SYT5 |
| 354 | NPR2 |
| 355 | LOC283174 |
| 356 | SLC6A4 |
| 357 | MAL2 |
| 358 | SNHG12 |
| 359 | JUP |
| 360 | LOC442249 |
| 361 | UBE2Q2 |
| 362 | MYBPHL |
| 363 | EEF2K |
| 364 | ACTN1 |
| 365 | PDZD8 |
| 366 | GABRA6 |
| 367 | HOXA5 |
| 368 | MMP24 |
| 369 | CEP76 |
| 370 | HOXD1 |
| 371 | REEP1 |
| 372 | FILIP1 |
| 373 | ARID3A |
| 374 | LRTM2 |
| 375 | SLC25A33 |
| 376 | INADL |
| 377 | DNMT1 |
| 378 | RNF113A |
| 379 | LDB2 |
| 380 | CALML4 |
| 381 | NPTX2 |
| 382 | SIPA1L2 |
| 383 | C16orf11 |
| 384 | PPP1R35 |
| 385 | 10-Sep |
| 386 | DDAH1 |
| 387 | THUMPD2 |
| 388 | WSCD2 |
| 389 | NCAN |
| 390 | CLDN9 |
| 391 | DPH3P1 |
| 392 | CHI3L1 |
| 393 | ZBTB8A |
| 394 | JARID2 |
| 395 | FUK |
| 396 | RGS14 |
| 397 | KCNS3 |
| 398 | COL11A2 |
| 399 | MRC1 |
| 400 | TBC1D19 |
| 401 | UNC13A |
| 402 | LOC100134173 |
| 403 | OR2A9P |
| 404 | LOC100287347 |
| 405 | EPPK1 |
| 406 | TIAM1 |
| 407 | BZRAP1 |
| 408 | CDH8 |
| 409 | RCAN3 |
| 410 | C17orf70 |
| 411 | ASTN1 |
| 412 | OAF |
| 413 | FSTL5 |
| 414 | LGALS3 |
| 415 | LOC644613 |
| 416 | IL15 |
| 417 | LDOC1L |
| 418 | PAX6 |
| 419 | C16orf79 |
| 420 | CAMK2A |
| 421 | HOXA7 |
| 422 | LPPR3 |
| 423 | FKBP9 |
| 424 | LOC100289230 |
| 425 | LINGO1 |
| 426 | FAM198B |
| 427 | ADAM28 |
| 428 | DENND4C |
| 429 | FKBP1A |
| 430 | NKIRAS2 |
| 431 | UHMK1 |
| 432 | HOXA6 |
| 433 | SLC8A2 |
| 434 | C11orf93 |
| 435 | ENO3 |
| 436 | OR14I1 |
| 437 | SNX1 |
| 438 | VWA5A |
| 439 | ZNF671 |
| 440 | C9orf147 |
| 441 | MPP3 |
| 442 | GALNTL4 |
| 443 | LOC344595 |
| 444 | EOMES |
| 445 | NRGN |
| 446 | TBL1XR1 |
| 447 | CCDC154 |
| 448 | LOC646627 |
| 449 | NKAIN1 |
| 450 | ALDH1L1 |
| 451 | RGS2 |
| 452 | GALNT12 |
| 453 | PSD |
| 454 | LOC100128343 |
| 455 | SH3RF1 |
| 456 | THBS3 |
| 457 | AMY1B |
| 458 | CPPED1 |
| 459 | MRPS18C |
| 460 | BZW2 |
| 461 | NUDT3 |
| 462 | PILRA |
| 463 | IFIT5 |
| 464 | GAS2 |
| 465 | ABHD11 |
| 466 | CRHR1 |
| 467 | ARHGAP24 |
| 468 | ZNF148 |
| 469 | POP1 |
| 470 | PPP1R1B |
| 471 | PDLIM2 |
| 472 | ABR |
| 473 | ALG1 |
| 474 | LPCAT2 |
| 475 | PAMR1 |
| 476 | INPP5F |
| 477 | TUBGCP6 |
| 478 | ALS2 |
| 479 | ZBED1 |
| 480 | KRT18P55 |
| 481 | EPCAM |
| 482 | MAST3 |
| 483 | WIF1 |
| 484 | PRSS22 |
| 485 | SHROOM1 |
| 486 | PPT2 |
| 487 | MYO1B |
| 488 | ISLR2 |
| 489 | CAMK2B |
| 490 | PP13 |
| 491 | BAHCC1 |
| 492 | TNFRSF25 |
| 493 | NYNRIN |
| 494 | BACE2 |
| 495 | C9orf171 |
| 496 | FRMPD4 |
| 497 | ACLY |
| 498 | HRASLS |
| 499 | TSTD1 |
| 500 | NCALD |
