# Supplementary material for: Analysis of Gene Expression Profiles in the Human Brain Stem, Cerebellum and Cerebral Cortex
Source: PLoS One. 2016 Jul 19;11(7):e0159395. doi: 10.1371/journal.pone.0159395 (PMC4951119; doi:10.1371/journal.pone.0159395)
Supplement: S2 Table — (DOCX) [file pone.0159395.s008.docx]

**S2 Table.** The accuracies obtained using the revised IFS method

1. The dataset of H0351.1009 was selected as the training dataset

| Number of features | H0351.1012 | | | | H0351.1015 | | | | H0351.1016 | | | | H0351.2001 | | | | H0351.2002 | | | |
| --- | --- | --- | --- | --- | --- | --- | --- | --- | --- | --- | --- | --- | --- | --- | --- | --- | --- | --- | --- | --- |
|  | class1 | class2 | class3 | total | class1 | class2 | class3 | total | class1 | class2 | class3 | total | class1 | class2 | class3 | total | class1 | class2 | class3 | total |
| 4 | 0.875 | 0.917 | 0.993 | 0.968 | 0.684 | 0.968 | 0.994 | 0.938 | 0.763 | 0.950 | 1.000 | 0.964 | 0.844 | 0.736 | 1.000 | 0.960 | 0.638 | 0.904 | 1.000 | 0.915 |
| 5 | 0.950 | 0.917 | 0.993 | 0.979 | 0.772 | 0.968 | 0.994 | 0.953 | 0.881 | 0.950 | 1.000 | 0.978 | 0.916 | 0.774 | 0.999 | 0.973 | 0.814 | 0.892 | 1.000 | 0.951 |
| 6 | 0.863 | 0.917 | 0.995 | 0.968 | 0.709 | 0.984 | 0.994 | 0.945 | 0.729 | 0.963 | 1.000 | 0.962 | 0.825 | 0.774 | 1.000 | 0.959 | 0.739 | 0.892 | 1.000 | 0.935 |
| 7 | 0.863 | 0.917 | 0.995 | 0.968 | 0.709 | 0.968 | 0.994 | 0.943 | 0.729 | 0.963 | 1.000 | 0.962 | 0.831 | 0.774 | 0.999 | 0.959 | 0.734 | 0.892 | 1.000 | 0.934 |
| 8 | 0.863 | 0.917 | 0.995 | 0.968 | 0.696 | 0.968 | 0.994 | 0.940 | 0.729 | 0.950 | 1.000 | 0.960 | 0.896 | 0.774 | 0.997 | 0.968 | 0.755 | 0.892 | 1.000 | 0.938 |
| 9 | 0.888 | 0.917 | 1.000 | 0.975 | 0.734 | 0.968 | 0.997 | 0.949 | 0.763 | 0.963 | 1.000 | 0.966 | 0.916 | 0.774 | 0.999 | 0.973 | 0.793 | 0.892 | 1.000 | 0.946 |
| 10 | 0.900 | 0.917 | 1.000 | 0.977 | 0.759 | 0.968 | 0.997 | 0.953 | 0.763 | 0.950 | 1.000 | 0.964 | 0.909 | 0.774 | 1.000 | 0.973 | 0.793 | 0.892 | 1.000 | 0.946 |
| 11 | 0.900 | 0.917 | 0.998 | 0.975 | 0.785 | 0.968 | 0.997 | 0.957 | 0.763 | 0.950 | 1.000 | 0.964 | 0.948 | 0.774 | 0.999 | 0.978 | 0.755 | 0.892 | 1.000 | 0.938 |
| 12 | 0.888 | 0.917 | 1.000 | 0.975 | 0.759 | 0.968 | 0.997 | 0.953 | 0.780 | 0.950 | 1.000 | 0.966 | 0.948 | 0.774 | 0.997 | 0.977 | 0.729 | 0.892 | 1.000 | 0.933 |
| 13 | 0.888 | 0.917 | 1.000 | 0.975 | 0.772 | 0.968 | 1.000 | 0.957 | 0.780 | 0.950 | 1.000 | 0.966 | 0.922 | 0.774 | 0.999 | 0.974 | 0.729 | 0.892 | 1.000 | 0.933 |
| 14 | 0.900 | 0.917 | 1.000 | 0.977 | 0.772 | 0.968 | 1.000 | 0.957 | 0.780 | 0.950 | 1.000 | 0.966 | 0.916 | 0.774 | 0.999 | 0.973 | 0.739 | 0.892 | 1.000 | 0.935 |
| 15 | 0.900 | 0.917 | 1.000 | 0.977 | 0.772 | 0.968 | 1.000 | 0.957 | 0.797 | 0.950 | 1.000 | 0.968 | 0.922 | 0.774 | 0.999 | 0.974 | 0.729 | 0.892 | 1.000 | 0.933 |
| 16 | 0.875 | 0.917 | 1.000 | 0.974 | 0.797 | 0.968 | 1.000 | 0.962 | 0.763 | 0.950 | 1.000 | 0.964 | 0.903 | 0.774 | 0.999 | 0.970 | 0.691 | 0.892 | 1.000 | 0.925 |
| 17 | 0.875 | 0.917 | 1.000 | 0.974 | 0.747 | 0.968 | 1.000 | 0.953 | 0.763 | 0.963 | 1.000 | 0.966 | 0.870 | 0.774 | 0.999 | 0.965 | 0.702 | 0.892 | 1.000 | 0.927 |
| 18 | 0.913 | 0.938 | 1.000 | 0.981 | 0.747 | 0.968 | 1.000 | 0.953 | 0.831 | 0.963 | 1.000 | 0.974 | 0.896 | 0.792 | 0.997 | 0.969 | 0.734 | 0.904 | 1.000 | 0.935 |
| 19 | 0.900 | 0.938 | 1.000 | 0.979 | 0.772 | 0.968 | 1.000 | 0.957 | 0.847 | 0.963 | 1.000 | 0.976 | 0.909 | 0.792 | 0.997 | 0.971 | 0.734 | 0.904 | 1.000 | 0.935 |
| 20 | 0.900 | 0.917 | 1.000 | 0.977 | 0.772 | 0.968 | 1.000 | 0.957 | 0.847 | 0.963 | 1.000 | 0.976 | 0.916 | 0.774 | 0.997 | 0.971 | 0.739 | 0.916 | 1.000 | 0.937 |
| 21 | 0.900 | 0.938 | 1.000 | 0.979 | 0.772 | 0.968 | 1.000 | 0.957 | 0.831 | 0.963 | 1.000 | 0.974 | 0.903 | 0.792 | 0.999 | 0.971 | 0.734 | 0.940 | 1.000 | 0.938 |
| 22 | 0.888 | 0.917 | 1.000 | 0.975 | 0.772 | 0.968 | 1.000 | 0.957 | 0.847 | 0.963 | 1.000 | 0.976 | 0.877 | 0.792 | 0.999 | 0.967 | 0.734 | 0.916 | 1.000 | 0.936 |
| 23 | 0.913 | 0.958 | 1.000 | 0.983 | 0.785 | 0.968 | 1.000 | 0.960 | 0.847 | 0.963 | 1.000 | 0.976 | 0.896 | 0.792 | 0.999 | 0.970 | 0.755 | 0.952 | 1.000 | 0.944 |
| 24 | 0.925 | 0.958 | 1.000 | 0.985 | 0.797 | 0.968 | 1.000 | 0.962 | 0.847 | 0.963 | 1.000 | 0.976 | 0.896 | 0.792 | 0.999 | 0.970 | 0.750 | 0.952 | 1.000 | 0.943 |
| 25 | 0.938 | 0.958 | 1.000 | 0.987 | 0.797 | 0.968 | 1.000 | 0.962 | 0.847 | 0.963 | 1.000 | 0.976 | 0.883 | 0.811 | 0.999 | 0.969 | 0.745 | 0.952 | 1.000 | 0.942 |
| 26 | 0.938 | 0.958 | 1.000 | 0.987 | 0.797 | 0.968 | 1.000 | 0.962 | 0.847 | 0.963 | 1.000 | 0.976 | 0.883 | 0.811 | 0.999 | 0.969 | 0.745 | 0.952 | 1.000 | 0.942 |
| 27 | 0.925 | 0.958 | 1.000 | 0.985 | 0.810 | 0.968 | 1.000 | 0.964 | 0.864 | 0.963 | 1.000 | 0.978 | 0.890 | 0.811 | 0.999 | 0.970 | 0.761 | 0.952 | 1.000 | 0.945 |
| 28 | 0.963 | 0.958 | 1.000 | 0.991 | 0.810 | 0.968 | 1.000 | 0.964 | 0.864 | 0.963 | 1.000 | 0.978 | 0.890 | 0.811 | 0.999 | 0.970 | 0.777 | 0.952 | 1.000 | 0.948 |
| 29 | 0.963 | 0.958 | 1.000 | 0.991 | 0.810 | 0.968 | 1.000 | 0.964 | 0.864 | 0.963 | 1.000 | 0.978 | 0.890 | 0.811 | 0.999 | 0.970 | 0.777 | 0.952 | 1.000 | 0.948 |
| 30 | 0.963 | 0.979 | 1.000 | 0.992 | 0.848 | 0.984 | 1.000 | 0.972 | 0.847 | 0.963 | 1.000 | 0.976 | 0.896 | 0.887 | 0.999 | 0.976 | 0.782 | 0.952 | 1.000 | 0.950 |
| 31 | 0.963 | 0.979 | 1.000 | 0.992 | 0.835 | 0.984 | 1.000 | 0.970 | 0.847 | 0.963 | 1.000 | 0.976 | 0.896 | 0.830 | 0.999 | 0.973 | 0.782 | 0.964 | 1.000 | 0.951 |
| 32 | 0.963 | 1.000 | 1.000 | 0.994 | 0.785 | 1.000 | 1.000 | 0.964 | 0.864 | 0.963 | 1.000 | 0.978 | 0.903 | 0.868 | 0.999 | 0.976 | 0.782 | 0.928 | 1.000 | 0.947 |
| 33 | 0.963 | 0.979 | 1.000 | 0.992 | 0.772 | 1.000 | 1.000 | 0.962 | 0.864 | 0.963 | 1.000 | 0.978 | 0.896 | 0.868 | 0.999 | 0.975 | 0.787 | 0.928 | 1.000 | 0.948 |
| 34 | 0.938 | 1.000 | 1.000 | 0.991 | 0.759 | 1.000 | 1.000 | 0.960 | 0.847 | 1.000 | 1.000 | 0.982 | 0.831 | 0.906 | 1.000 | 0.967 | 0.718 | 0.952 | 1.000 | 0.936 |
| 35 | 0.913 | 1.000 | 1.000 | 0.987 | 0.759 | 1.000 | 1.000 | 0.960 | 0.831 | 1.000 | 1.000 | 0.980 | 0.825 | 0.906 | 1.000 | 0.966 | 0.707 | 0.952 | 1.000 | 0.934 |
| 36 | 0.913 | 1.000 | 1.000 | 0.987 | 0.759 | 1.000 | 1.000 | 0.960 | 0.780 | 1.000 | 1.000 | 0.974 | 0.825 | 0.906 | 1.000 | 0.966 | 0.707 | 0.952 | 1.000 | 0.934 |
| 37 | 0.913 | 1.000 | 1.000 | 0.987 | 0.772 | 1.000 | 1.000 | 0.962 | 0.780 | 1.000 | 1.000 | 0.974 | 0.831 | 0.906 | 1.000 | 0.967 | 0.707 | 0.964 | 1.000 | 0.935 |
| 38 | 0.888 | 1.000 | 1.000 | 0.983 | 0.759 | 1.000 | 1.000 | 0.960 | 0.780 | 1.000 | 1.000 | 0.974 | 0.844 | 0.906 | 1.000 | 0.969 | 0.713 | 0.964 | 1.000 | 0.936 |
| 39 | 0.888 | 1.000 | 1.000 | 0.983 | 0.759 | 1.000 | 1.000 | 0.960 | 0.797 | 1.000 | 1.000 | 0.976 | 0.851 | 0.906 | 1.000 | 0.970 | 0.718 | 0.964 | 1.000 | 0.937 |
| 40 | 0.888 | 1.000 | 1.000 | 0.983 | 0.759 | 1.000 | 1.000 | 0.960 | 0.797 | 1.000 | 1.000 | 0.976 | 0.857 | 0.906 | 1.000 | 0.971 | 0.718 | 0.964 | 1.000 | 0.937 |
| 41 | 0.900 | 1.000 | 1.000 | 0.985 | 0.759 | 1.000 | 1.000 | 0.960 | 0.797 | 1.000 | 1.000 | 0.976 | 0.857 | 0.906 | 1.000 | 0.971 | 0.718 | 0.964 | 1.000 | 0.937 |
| 42 | 0.913 | 1.000 | 1.000 | 0.987 | 0.772 | 1.000 | 1.000 | 0.962 | 0.763 | 1.000 | 1.000 | 0.972 | 0.831 | 0.906 | 1.000 | 0.967 | 0.702 | 0.988 | 1.000 | 0.936 |
| 43 | 0.850 | 1.000 | 1.000 | 0.977 | 0.722 | 1.000 | 1.000 | 0.953 | 0.763 | 1.000 | 1.000 | 0.972 | 0.838 | 0.906 | 1.000 | 0.968 | 0.697 | 1.000 | 1.000 | 0.936 |
| 44 | 0.925 | 1.000 | 1.000 | 0.989 | 0.797 | 1.000 | 1.000 | 0.966 | 0.831 | 1.000 | 1.000 | 0.980 | 0.864 | 0.962 | 1.000 | 0.976 | 0.729 | 1.000 | 1.000 | 0.943 |
| 45 | 0.925 | 1.000 | 1.000 | 0.989 | 0.797 | 1.000 | 1.000 | 0.966 | 0.831 | 1.000 | 1.000 | 0.980 | 0.870 | 0.962 | 1.000 | 0.977 | 0.723 | 1.000 | 1.000 | 0.942 |
| 46 | 0.913 | 1.000 | 1.000 | 0.987 | 0.785 | 1.000 | 1.000 | 0.964 | 0.814 | 1.000 | 1.000 | 0.978 | 0.864 | 0.943 | 1.000 | 0.975 | 0.729 | 1.000 | 1.000 | 0.943 |
| 47 | 0.900 | 1.000 | 1.000 | 0.985 | 0.785 | 1.000 | 1.000 | 0.964 | 0.814 | 1.000 | 1.000 | 0.978 | 0.877 | 0.943 | 1.000 | 0.977 | 0.729 | 1.000 | 1.000 | 0.943 |
| 48 | 0.900 | 1.000 | 1.000 | 0.985 | 0.785 | 1.000 | 1.000 | 0.964 | 0.831 | 1.000 | 1.000 | 0.980 | 0.877 | 0.943 | 1.000 | 0.977 | 0.729 | 1.000 | 1.000 | 0.943 |
| 49 | 0.913 | 1.000 | 1.000 | 0.987 | 0.785 | 1.000 | 1.000 | 0.964 | 0.831 | 1.000 | 1.000 | 0.980 | 0.883 | 0.925 | 1.000 | 0.977 | 0.734 | 1.000 | 1.000 | 0.944 |
| 50 | 0.900 | 1.000 | 1.000 | 0.985 | 0.785 | 1.000 | 1.000 | 0.964 | 0.831 | 1.000 | 1.000 | 0.980 | 0.877 | 0.925 | 1.000 | 0.976 | 0.729 | 1.000 | 1.000 | 0.943 |
| 51 | 0.913 | 1.000 | 1.000 | 0.987 | 0.785 | 1.000 | 1.000 | 0.964 | 0.831 | 1.000 | 1.000 | 0.980 | 0.883 | 0.925 | 0.999 | 0.976 | 0.734 | 1.000 | 1.000 | 0.944 |
| 52 | 0.925 | 1.000 | 1.000 | 0.989 | 0.785 | 1.000 | 1.000 | 0.964 | 0.831 | 1.000 | 1.000 | 0.980 | 0.883 | 0.925 | 1.000 | 0.977 | 0.766 | 1.000 | 1.000 | 0.951 |
| 53 | 0.938 | 1.000 | 1.000 | 0.991 | 0.785 | 1.000 | 1.000 | 0.964 | 0.814 | 1.000 | 1.000 | 0.978 | 0.883 | 0.925 | 1.000 | 0.977 | 0.761 | 1.000 | 1.000 | 0.950 |
| 54 | 0.913 | 1.000 | 1.000 | 0.987 | 0.785 | 1.000 | 1.000 | 0.964 | 0.814 | 1.000 | 1.000 | 0.978 | 0.870 | 0.962 | 1.000 | 0.977 | 0.718 | 1.000 | 1.000 | 0.941 |
| 55 | 0.913 | 1.000 | 1.000 | 0.987 | 0.785 | 1.000 | 1.000 | 0.964 | 0.814 | 1.000 | 1.000 | 0.978 | 0.870 | 0.962 | 1.000 | 0.977 | 0.707 | 1.000 | 1.000 | 0.938 |
| 56 | 0.863 | 1.000 | 1.000 | 0.979 | 0.734 | 1.000 | 1.000 | 0.955 | 0.814 | 1.000 | 1.000 | 0.978 | 0.864 | 0.962 | 0.999 | 0.975 | 0.686 | 1.000 | 1.000 | 0.934 |
| 57 | 0.863 | 1.000 | 1.000 | 0.979 | 0.747 | 1.000 | 1.000 | 0.957 | 0.814 | 1.000 | 1.000 | 0.978 | 0.864 | 0.962 | 0.999 | 0.975 | 0.686 | 1.000 | 1.000 | 0.934 |
| 58 | 0.863 | 1.000 | 1.000 | 0.979 | 0.734 | 1.000 | 1.000 | 0.955 | 0.814 | 1.000 | 1.000 | 0.978 | 0.864 | 0.962 | 0.999 | 0.975 | 0.686 | 1.000 | 1.000 | 0.934 |
| 59 | 0.875 | 1.000 | 1.000 | 0.981 | 0.734 | 1.000 | 1.000 | 0.955 | 0.814 | 1.000 | 1.000 | 0.978 | 0.870 | 0.962 | 0.997 | 0.975 | 0.681 | 1.000 | 1.000 | 0.933 |
| 60 | 0.888 | 1.000 | 1.000 | 0.983 | 0.734 | 1.000 | 1.000 | 0.955 | 0.814 | 1.000 | 1.000 | 0.978 | 0.870 | 0.962 | 0.997 | 0.975 | 0.707 | 1.000 | 1.000 | 0.938 |
| 61 | 0.850 | 1.000 | 1.000 | 0.977 | 0.684 | 1.000 | 1.000 | 0.947 | 0.797 | 1.000 | 1.000 | 0.976 | 0.870 | 0.962 | 0.996 | 0.974 | 0.654 | 1.000 | 1.000 | 0.927 |
| 62 | 0.850 | 1.000 | 1.000 | 0.977 | 0.709 | 1.000 | 1.000 | 0.951 | 0.814 | 1.000 | 1.000 | 0.978 | 0.851 | 0.962 | 1.000 | 0.974 | 0.654 | 1.000 | 1.000 | 0.927 |
| 63 | 0.850 | 1.000 | 1.000 | 0.977 | 0.722 | 1.000 | 1.000 | 0.953 | 0.814 | 1.000 | 1.000 | 0.978 | 0.851 | 0.962 | 1.000 | 0.974 | 0.676 | 1.000 | 1.000 | 0.932 |
| 64 | 0.850 | 1.000 | 1.000 | 0.977 | 0.709 | 1.000 | 1.000 | 0.951 | 0.814 | 1.000 | 1.000 | 0.978 | 0.851 | 0.962 | 1.000 | 0.974 | 0.676 | 1.000 | 1.000 | 0.932 |
| 65 | 0.850 | 1.000 | 1.000 | 0.977 | 0.709 | 1.000 | 1.000 | 0.951 | 0.814 | 1.000 | 1.000 | 0.978 | 0.851 | 0.962 | 1.000 | 0.974 | 0.670 | 1.000 | 1.000 | 0.931 |
| 66 | 0.850 | 1.000 | 1.000 | 0.977 | 0.709 | 1.000 | 1.000 | 0.951 | 0.814 | 1.000 | 1.000 | 0.978 | 0.851 | 0.962 | 1.000 | 0.974 | 0.676 | 1.000 | 1.000 | 0.932 |
| 67 | 0.863 | 1.000 | 1.000 | 0.979 | 0.684 | 1.000 | 1.000 | 0.947 | 0.780 | 1.000 | 1.000 | 0.974 | 0.844 | 0.962 | 0.999 | 0.971 | 0.691 | 1.000 | 1.000 | 0.935 |
| 68 | 0.863 | 1.000 | 1.000 | 0.979 | 0.684 | 1.000 | 1.000 | 0.947 | 0.780 | 1.000 | 1.000 | 0.974 | 0.838 | 0.962 | 1.000 | 0.971 | 0.691 | 1.000 | 1.000 | 0.935 |
| 69 | 0.838 | 1.000 | 1.000 | 0.975 | 0.671 | 1.000 | 1.000 | 0.945 | 0.763 | 1.000 | 1.000 | 0.972 | 0.818 | 0.943 | 0.997 | 0.965 | 0.670 | 1.000 | 1.000 | 0.931 |
| 70 | 0.838 | 1.000 | 1.000 | 0.975 | 0.684 | 1.000 | 1.000 | 0.947 | 0.763 | 1.000 | 1.000 | 0.972 | 0.825 | 0.943 | 0.995 | 0.964 | 0.665 | 1.000 | 1.000 | 0.929 |
| 71 | 0.838 | 1.000 | 1.000 | 0.975 | 0.684 | 1.000 | 1.000 | 0.947 | 0.780 | 1.000 | 1.000 | 0.974 | 0.825 | 0.943 | 0.996 | 0.965 | 0.676 | 1.000 | 1.000 | 0.932 |
| 72 | 0.838 | 1.000 | 1.000 | 0.975 | 0.684 | 1.000 | 1.000 | 0.947 | 0.763 | 1.000 | 1.000 | 0.972 | 0.851 | 0.943 | 0.996 | 0.969 | 0.681 | 1.000 | 1.000 | 0.933 |
| 73 | 0.838 | 1.000 | 1.000 | 0.975 | 0.671 | 1.000 | 1.000 | 0.945 | 0.763 | 1.000 | 1.000 | 0.972 | 0.844 | 0.943 | 0.996 | 0.968 | 0.660 | 1.000 | 1.000 | 0.928 |
| 74 | 0.838 | 1.000 | 1.000 | 0.975 | 0.671 | 1.000 | 1.000 | 0.945 | 0.763 | 1.000 | 1.000 | 0.972 | 0.844 | 0.943 | 0.996 | 0.968 | 0.660 | 1.000 | 1.000 | 0.928 |
| 75 | 0.838 | 1.000 | 1.000 | 0.975 | 0.671 | 1.000 | 1.000 | 0.945 | 0.763 | 1.000 | 1.000 | 0.972 | 0.838 | 0.943 | 0.996 | 0.967 | 0.644 | 1.000 | 1.000 | 0.925 |
| 76 | 0.838 | 1.000 | 1.000 | 0.975 | 0.671 | 1.000 | 1.000 | 0.945 | 0.746 | 1.000 | 1.000 | 0.970 | 0.844 | 0.943 | 0.995 | 0.967 | 0.654 | 1.000 | 1.000 | 0.927 |
| 77 | 0.838 | 1.000 | 1.000 | 0.975 | 0.633 | 1.000 | 1.000 | 0.938 | 0.763 | 1.000 | 1.000 | 0.972 | 0.864 | 0.943 | 0.995 | 0.970 | 0.654 | 1.000 | 1.000 | 0.927 |
| 78 | 0.838 | 1.000 | 1.000 | 0.975 | 0.633 | 1.000 | 1.000 | 0.938 | 0.763 | 1.000 | 1.000 | 0.972 | 0.864 | 0.943 | 0.995 | 0.970 | 0.654 | 1.000 | 1.000 | 0.927 |
| 79 | 0.838 | 1.000 | 1.000 | 0.975 | 0.646 | 1.000 | 1.000 | 0.940 | 0.780 | 1.000 | 1.000 | 0.974 | 0.857 | 0.943 | 0.995 | 0.969 | 0.638 | 1.000 | 1.000 | 0.924 |
| 80 | 0.825 | 1.000 | 1.000 | 0.974 | 0.646 | 1.000 | 1.000 | 0.940 | 0.797 | 1.000 | 1.000 | 0.976 | 0.857 | 0.943 | 0.995 | 0.969 | 0.638 | 1.000 | 1.000 | 0.924 |
| 81 | 0.825 | 1.000 | 1.000 | 0.974 | 0.646 | 1.000 | 1.000 | 0.940 | 0.780 | 1.000 | 1.000 | 0.974 | 0.857 | 0.943 | 0.995 | 0.969 | 0.638 | 1.000 | 1.000 | 0.924 |
| 82 | 0.813 | 1.000 | 1.000 | 0.972 | 0.620 | 1.000 | 1.000 | 0.936 | 0.746 | 1.000 | 1.000 | 0.970 | 0.857 | 0.943 | 0.995 | 0.969 | 0.622 | 1.000 | 1.000 | 0.920 |
| 83 | 0.825 | 1.000 | 1.000 | 0.974 | 0.658 | 1.000 | 1.000 | 0.943 | 0.797 | 1.000 | 1.000 | 0.976 | 0.870 | 0.943 | 0.995 | 0.971 | 0.660 | 1.000 | 1.000 | 0.928 |
| 84 | 0.825 | 1.000 | 1.000 | 0.974 | 0.646 | 1.000 | 1.000 | 0.940 | 0.797 | 1.000 | 1.000 | 0.976 | 0.870 | 0.943 | 0.995 | 0.971 | 0.660 | 1.000 | 1.000 | 0.928 |
| 85 | 0.825 | 1.000 | 1.000 | 0.974 | 0.646 | 1.000 | 1.000 | 0.940 | 0.797 | 1.000 | 1.000 | 0.976 | 0.870 | 0.943 | 0.995 | 0.971 | 0.644 | 1.000 | 1.000 | 0.925 |
| 86 | 0.813 | 1.000 | 1.000 | 0.972 | 0.633 | 1.000 | 1.000 | 0.938 | 0.780 | 1.000 | 1.000 | 0.974 | 0.864 | 0.943 | 0.995 | 0.970 | 0.649 | 1.000 | 1.000 | 0.926 |
| 87 | 0.813 | 1.000 | 1.000 | 0.972 | 0.633 | 1.000 | 1.000 | 0.938 | 0.763 | 1.000 | 1.000 | 0.972 | 0.870 | 0.943 | 0.995 | 0.971 | 0.654 | 1.000 | 1.000 | 0.927 |
| 88 | 0.788 | 1.000 | 1.000 | 0.968 | 0.633 | 1.000 | 1.000 | 0.938 | 0.746 | 1.000 | 1.000 | 0.970 | 0.870 | 0.943 | 0.995 | 0.971 | 0.649 | 1.000 | 1.000 | 0.926 |
| 89 | 0.788 | 1.000 | 1.000 | 0.968 | 0.646 | 1.000 | 1.000 | 0.940 | 0.746 | 1.000 | 1.000 | 0.970 | 0.864 | 0.943 | 0.995 | 0.970 | 0.654 | 1.000 | 1.000 | 0.927 |
| 90 | 0.788 | 1.000 | 1.000 | 0.968 | 0.646 | 1.000 | 1.000 | 0.940 | 0.746 | 1.000 | 1.000 | 0.970 | 0.864 | 0.943 | 0.995 | 0.970 | 0.654 | 1.000 | 1.000 | 0.927 |
| 91 | 0.813 | 1.000 | 1.000 | 0.972 | 0.646 | 1.000 | 1.000 | 0.940 | 0.746 | 1.000 | 1.000 | 0.970 | 0.864 | 0.943 | 0.995 | 0.970 | 0.654 | 1.000 | 1.000 | 0.927 |
| 92 | 0.813 | 1.000 | 1.000 | 0.972 | 0.646 | 1.000 | 1.000 | 0.940 | 0.746 | 1.000 | 1.000 | 0.970 | 0.877 | 0.943 | 0.995 | 0.973 | 0.649 | 1.000 | 1.000 | 0.926 |
| 93 | 0.813 | 1.000 | 1.000 | 0.972 | 0.646 | 1.000 | 1.000 | 0.940 | 0.729 | 1.000 | 1.000 | 0.968 | 0.877 | 0.943 | 0.995 | 0.973 | 0.644 | 1.000 | 1.000 | 0.925 |
| 94 | 0.788 | 1.000 | 1.000 | 0.968 | 0.633 | 1.000 | 1.000 | 0.938 | 0.712 | 1.000 | 1.000 | 0.966 | 0.877 | 0.943 | 0.995 | 0.973 | 0.670 | 1.000 | 1.000 | 0.931 |
| 95 | 0.800 | 1.000 | 1.000 | 0.970 | 0.633 | 1.000 | 1.000 | 0.938 | 0.712 | 1.000 | 1.000 | 0.966 | 0.883 | 0.943 | 0.995 | 0.974 | 0.681 | 1.000 | 1.000 | 0.933 |
| 96 | 0.788 | 1.000 | 1.000 | 0.968 | 0.633 | 1.000 | 1.000 | 0.938 | 0.695 | 1.000 | 1.000 | 0.964 | 0.883 | 0.943 | 0.995 | 0.974 | 0.676 | 1.000 | 1.000 | 0.932 |
| 97 | 0.788 | 1.000 | 1.000 | 0.968 | 0.633 | 1.000 | 1.000 | 0.938 | 0.695 | 1.000 | 1.000 | 0.964 | 0.883 | 0.943 | 0.995 | 0.974 | 0.676 | 1.000 | 1.000 | 0.932 |
| 98 | 0.788 | 1.000 | 1.000 | 0.968 | 0.633 | 1.000 | 1.000 | 0.938 | 0.695 | 1.000 | 1.000 | 0.964 | 0.896 | 0.943 | 0.995 | 0.976 | 0.739 | 1.000 | 1.000 | 0.945 |
| 99 | 0.788 | 1.000 | 1.000 | 0.968 | 0.633 | 1.000 | 1.000 | 0.938 | 0.695 | 1.000 | 1.000 | 0.964 | 0.896 | 0.943 | 0.995 | 0.976 | 0.745 | 1.000 | 1.000 | 0.946 |
| 100 | 0.788 | 1.000 | 1.000 | 0.968 | 0.633 | 1.000 | 1.000 | 0.938 | 0.695 | 1.000 | 1.000 | 0.964 | 0.896 | 0.943 | 0.995 | 0.976 | 0.750 | 1.000 | 1.000 | 0.947 |
| 101 | 0.788 | 1.000 | 1.000 | 0.968 | 0.633 | 1.000 | 1.000 | 0.938 | 0.695 | 1.000 | 1.000 | 0.964 | 0.890 | 0.943 | 0.995 | 0.975 | 0.739 | 1.000 | 1.000 | 0.945 |
| 102 | 0.788 | 1.000 | 1.000 | 0.968 | 0.633 | 1.000 | 1.000 | 0.938 | 0.695 | 1.000 | 1.000 | 0.964 | 0.890 | 0.943 | 0.995 | 0.975 | 0.739 | 1.000 | 1.000 | 0.945 |
| 103 | 0.788 | 1.000 | 1.000 | 0.968 | 0.633 | 1.000 | 1.000 | 0.938 | 0.678 | 1.000 | 1.000 | 0.962 | 0.890 | 0.943 | 0.995 | 0.975 | 0.750 | 1.000 | 1.000 | 0.947 |
| 104 | 0.775 | 1.000 | 1.000 | 0.966 | 0.633 | 1.000 | 1.000 | 0.938 | 0.695 | 1.000 | 1.000 | 0.964 | 0.896 | 0.943 | 0.995 | 0.976 | 0.739 | 1.000 | 1.000 | 0.945 |
| 105 | 0.775 | 1.000 | 1.000 | 0.966 | 0.633 | 1.000 | 1.000 | 0.938 | 0.695 | 1.000 | 1.000 | 0.964 | 0.890 | 0.943 | 0.995 | 0.975 | 0.729 | 1.000 | 1.000 | 0.943 |
| 106 | 0.775 | 1.000 | 1.000 | 0.966 | 0.633 | 1.000 | 1.000 | 0.938 | 0.712 | 1.000 | 1.000 | 0.966 | 0.890 | 0.943 | 0.995 | 0.975 | 0.729 | 1.000 | 1.000 | 0.943 |
| 107 | 0.775 | 1.000 | 1.000 | 0.966 | 0.633 | 1.000 | 1.000 | 0.938 | 0.695 | 1.000 | 1.000 | 0.964 | 0.883 | 0.943 | 0.993 | 0.973 | 0.734 | 1.000 | 1.000 | 0.944 |
| 108 | 0.775 | 1.000 | 1.000 | 0.966 | 0.633 | 1.000 | 1.000 | 0.938 | 0.712 | 1.000 | 1.000 | 0.966 | 0.890 | 0.943 | 0.993 | 0.974 | 0.734 | 1.000 | 1.000 | 0.944 |
| 109 | 0.775 | 1.000 | 1.000 | 0.966 | 0.633 | 1.000 | 1.000 | 0.938 | 0.712 | 1.000 | 1.000 | 0.966 | 0.890 | 0.943 | 0.993 | 0.974 | 0.729 | 1.000 | 1.000 | 0.943 |
| 110 | 0.775 | 1.000 | 1.000 | 0.966 | 0.633 | 1.000 | 1.000 | 0.938 | 0.712 | 1.000 | 1.000 | 0.966 | 0.890 | 0.943 | 0.993 | 0.974 | 0.723 | 1.000 | 1.000 | 0.942 |
| 111 | 0.775 | 1.000 | 1.000 | 0.966 | 0.633 | 1.000 | 1.000 | 0.938 | 0.712 | 1.000 | 1.000 | 0.966 | 0.890 | 0.943 | 0.993 | 0.974 | 0.723 | 1.000 | 1.000 | 0.942 |
| 112 | 0.775 | 1.000 | 1.000 | 0.966 | 0.633 | 1.000 | 1.000 | 0.938 | 0.712 | 1.000 | 1.000 | 0.966 | 0.890 | 0.943 | 0.993 | 0.974 | 0.729 | 1.000 | 1.000 | 0.943 |
| 113 | 0.775 | 1.000 | 1.000 | 0.966 | 0.633 | 1.000 | 1.000 | 0.938 | 0.695 | 1.000 | 1.000 | 0.964 | 0.883 | 0.943 | 0.993 | 0.973 | 0.723 | 1.000 | 1.000 | 0.942 |
| 114 | 0.775 | 1.000 | 1.000 | 0.966 | 0.633 | 1.000 | 1.000 | 0.938 | 0.695 | 1.000 | 1.000 | 0.964 | 0.883 | 0.943 | 0.993 | 0.973 | 0.718 | 1.000 | 1.000 | 0.941 |
| 115 | 0.763 | 1.000 | 1.000 | 0.964 | 0.620 | 1.000 | 1.000 | 0.936 | 0.678 | 1.000 | 1.000 | 0.962 | 0.883 | 0.943 | 0.993 | 0.973 | 0.691 | 1.000 | 1.000 | 0.935 |
| 116 | 0.763 | 1.000 | 1.000 | 0.964 | 0.620 | 1.000 | 1.000 | 0.936 | 0.678 | 1.000 | 1.000 | 0.962 | 0.883 | 0.943 | 0.993 | 0.973 | 0.691 | 1.000 | 1.000 | 0.935 |
| 117 | 0.763 | 1.000 | 1.000 | 0.964 | 0.620 | 1.000 | 1.000 | 0.936 | 0.678 | 1.000 | 1.000 | 0.962 | 0.883 | 0.962 | 0.993 | 0.974 | 0.691 | 1.000 | 1.000 | 0.935 |
| 118 | 0.750 | 1.000 | 1.000 | 0.962 | 0.608 | 1.000 | 1.000 | 0.934 | 0.644 | 1.000 | 1.000 | 0.958 | 0.890 | 0.962 | 0.993 | 0.975 | 0.676 | 1.000 | 1.000 | 0.932 |
| 119 | 0.750 | 1.000 | 1.000 | 0.962 | 0.608 | 1.000 | 1.000 | 0.934 | 0.644 | 1.000 | 1.000 | 0.958 | 0.890 | 0.962 | 0.993 | 0.975 | 0.665 | 1.000 | 1.000 | 0.929 |
| 120 | 0.763 | 1.000 | 1.000 | 0.964 | 0.620 | 1.000 | 1.000 | 0.936 | 0.661 | 1.000 | 1.000 | 0.960 | 0.890 | 0.962 | 0.993 | 0.975 | 0.660 | 1.000 | 1.000 | 0.928 |
| 121 | 0.763 | 1.000 | 1.000 | 0.964 | 0.620 | 1.000 | 1.000 | 0.936 | 0.661 | 1.000 | 1.000 | 0.960 | 0.890 | 0.962 | 0.993 | 0.975 | 0.654 | 1.000 | 1.000 | 0.927 |
| 122 | 0.763 | 1.000 | 1.000 | 0.964 | 0.633 | 1.000 | 1.000 | 0.938 | 0.661 | 1.000 | 1.000 | 0.960 | 0.890 | 0.943 | 0.993 | 0.974 | 0.660 | 1.000 | 1.000 | 0.928 |
| 123 | 0.725 | 1.000 | 1.000 | 0.958 | 0.608 | 1.000 | 1.000 | 0.934 | 0.627 | 1.000 | 1.000 | 0.956 | 0.883 | 0.962 | 0.993 | 0.974 | 0.644 | 1.000 | 1.000 | 0.925 |
| 124 | 0.725 | 1.000 | 1.000 | 0.958 | 0.608 | 1.000 | 1.000 | 0.934 | 0.627 | 1.000 | 1.000 | 0.956 | 0.883 | 0.943 | 0.993 | 0.973 | 0.644 | 1.000 | 1.000 | 0.925 |
| 125 | 0.725 | 1.000 | 1.000 | 0.958 | 0.608 | 1.000 | 1.000 | 0.934 | 0.627 | 1.000 | 1.000 | 0.956 | 0.883 | 0.943 | 0.993 | 0.973 | 0.649 | 1.000 | 1.000 | 0.926 |
| 126 | 0.725 | 1.000 | 1.000 | 0.958 | 0.608 | 1.000 | 1.000 | 0.934 | 0.627 | 1.000 | 1.000 | 0.956 | 0.883 | 0.943 | 0.993 | 0.973 | 0.649 | 1.000 | 1.000 | 0.926 |
| 127 | 0.725 | 1.000 | 1.000 | 0.958 | 0.608 | 1.000 | 1.000 | 0.934 | 0.627 | 1.000 | 1.000 | 0.956 | 0.870 | 0.943 | 0.993 | 0.970 | 0.649 | 1.000 | 1.000 | 0.926 |
| 128 | 0.725 | 1.000 | 1.000 | 0.958 | 0.608 | 1.000 | 1.000 | 0.934 | 0.627 | 1.000 | 1.000 | 0.956 | 0.870 | 0.943 | 0.993 | 0.970 | 0.649 | 1.000 | 1.000 | 0.926 |
| 129 | 0.725 | 1.000 | 1.000 | 0.958 | 0.608 | 1.000 | 1.000 | 0.934 | 0.627 | 1.000 | 1.000 | 0.956 | 0.864 | 0.943 | 0.993 | 0.969 | 0.654 | 1.000 | 1.000 | 0.927 |
| 130 | 0.725 | 1.000 | 1.000 | 0.958 | 0.608 | 1.000 | 1.000 | 0.934 | 0.627 | 1.000 | 1.000 | 0.956 | 0.857 | 0.943 | 0.993 | 0.968 | 0.654 | 1.000 | 1.000 | 0.927 |
| 131 | 0.725 | 1.000 | 1.000 | 0.958 | 0.608 | 1.000 | 1.000 | 0.934 | 0.627 | 1.000 | 1.000 | 0.956 | 0.857 | 0.943 | 0.993 | 0.968 | 0.649 | 1.000 | 1.000 | 0.926 |
| 132 | 0.763 | 1.000 | 1.000 | 0.964 | 0.633 | 1.000 | 1.000 | 0.938 | 0.661 | 1.000 | 1.000 | 0.960 | 0.870 | 0.943 | 0.996 | 0.973 | 0.681 | 1.000 | 1.000 | 0.933 |
| 133 | 0.763 | 1.000 | 1.000 | 0.964 | 0.633 | 1.000 | 1.000 | 0.938 | 0.661 | 1.000 | 1.000 | 0.960 | 0.870 | 0.943 | 0.996 | 0.973 | 0.681 | 1.000 | 1.000 | 0.933 |
| 134 | 0.775 | 1.000 | 1.000 | 0.966 | 0.633 | 1.000 | 1.000 | 0.938 | 0.678 | 1.000 | 1.000 | 0.962 | 0.877 | 0.943 | 0.995 | 0.973 | 0.691 | 1.000 | 1.000 | 0.935 |
| 135 | 0.775 | 1.000 | 1.000 | 0.966 | 0.633 | 1.000 | 1.000 | 0.938 | 0.661 | 1.000 | 1.000 | 0.960 | 0.883 | 0.943 | 0.996 | 0.975 | 0.686 | 1.000 | 1.000 | 0.934 |
| 136 | 0.788 | 1.000 | 1.000 | 0.968 | 0.646 | 1.000 | 1.000 | 0.940 | 0.678 | 1.000 | 1.000 | 0.962 | 0.883 | 0.943 | 0.996 | 0.975 | 0.691 | 1.000 | 1.000 | 0.935 |
| 137 | 0.788 | 1.000 | 1.000 | 0.968 | 0.646 | 1.000 | 1.000 | 0.940 | 0.661 | 1.000 | 1.000 | 0.960 | 0.877 | 0.943 | 0.996 | 0.974 | 0.691 | 1.000 | 1.000 | 0.935 |
| 138 | 0.788 | 1.000 | 1.000 | 0.968 | 0.646 | 1.000 | 1.000 | 0.940 | 0.661 | 1.000 | 1.000 | 0.960 | 0.877 | 0.943 | 0.996 | 0.974 | 0.697 | 1.000 | 1.000 | 0.936 |
| 139 | 0.800 | 1.000 | 1.000 | 0.970 | 0.646 | 1.000 | 1.000 | 0.940 | 0.678 | 1.000 | 1.000 | 0.962 | 0.883 | 0.943 | 0.996 | 0.975 | 0.697 | 1.000 | 1.000 | 0.936 |
| 140 | 0.788 | 1.000 | 1.000 | 0.968 | 0.646 | 1.000 | 1.000 | 0.940 | 0.661 | 1.000 | 1.000 | 0.960 | 0.883 | 0.943 | 0.996 | 0.975 | 0.702 | 1.000 | 1.000 | 0.937 |
| 141 | 0.775 | 1.000 | 1.000 | 0.966 | 0.646 | 1.000 | 1.000 | 0.940 | 0.678 | 1.000 | 1.000 | 0.962 | 0.883 | 0.943 | 0.996 | 0.975 | 0.697 | 1.000 | 1.000 | 0.936 |
| 142 | 0.775 | 1.000 | 1.000 | 0.966 | 0.646 | 1.000 | 1.000 | 0.940 | 0.678 | 1.000 | 1.000 | 0.962 | 0.877 | 0.943 | 0.995 | 0.973 | 0.702 | 1.000 | 1.000 | 0.937 |
| 143 | 0.775 | 1.000 | 1.000 | 0.966 | 0.646 | 1.000 | 1.000 | 0.940 | 0.661 | 1.000 | 1.000 | 0.960 | 0.877 | 0.943 | 0.995 | 0.973 | 0.707 | 1.000 | 1.000 | 0.938 |
| 144 | 0.775 | 1.000 | 1.000 | 0.966 | 0.646 | 1.000 | 1.000 | 0.940 | 0.661 | 1.000 | 1.000 | 0.960 | 0.877 | 0.943 | 0.995 | 0.973 | 0.707 | 1.000 | 1.000 | 0.938 |
| 145 | 0.775 | 1.000 | 1.000 | 0.966 | 0.646 | 1.000 | 1.000 | 0.940 | 0.661 | 1.000 | 1.000 | 0.960 | 0.877 | 0.943 | 0.995 | 0.973 | 0.713 | 1.000 | 1.000 | 0.940 |
| 146 | 0.775 | 1.000 | 1.000 | 0.966 | 0.646 | 1.000 | 1.000 | 0.940 | 0.661 | 1.000 | 1.000 | 0.960 | 0.877 | 0.943 | 0.995 | 0.973 | 0.707 | 1.000 | 1.000 | 0.938 |
| 147 | 0.763 | 1.000 | 1.000 | 0.964 | 0.620 | 1.000 | 1.000 | 0.936 | 0.661 | 1.000 | 1.000 | 0.960 | 0.883 | 0.943 | 0.995 | 0.974 | 0.702 | 1.000 | 1.000 | 0.937 |
| 148 | 0.775 | 1.000 | 1.000 | 0.966 | 0.620 | 1.000 | 1.000 | 0.936 | 0.678 | 1.000 | 1.000 | 0.962 | 0.877 | 0.943 | 0.995 | 0.973 | 0.702 | 1.000 | 1.000 | 0.937 |
| 149 | 0.775 | 1.000 | 1.000 | 0.966 | 0.620 | 1.000 | 1.000 | 0.936 | 0.678 | 1.000 | 1.000 | 0.962 | 0.877 | 0.943 | 0.995 | 0.973 | 0.702 | 1.000 | 1.000 | 0.937 |
| 150 | 0.775 | 1.000 | 1.000 | 0.966 | 0.620 | 1.000 | 1.000 | 0.936 | 0.678 | 1.000 | 1.000 | 0.962 | 0.877 | 0.943 | 0.995 | 0.973 | 0.702 | 1.000 | 1.000 | 0.937 |
| 151 | 0.775 | 1.000 | 1.000 | 0.966 | 0.620 | 1.000 | 1.000 | 0.936 | 0.678 | 1.000 | 1.000 | 0.962 | 0.877 | 0.943 | 0.995 | 0.973 | 0.702 | 1.000 | 1.000 | 0.937 |
| 152 | 0.775 | 1.000 | 1.000 | 0.966 | 0.646 | 1.000 | 1.000 | 0.940 | 0.678 | 1.000 | 1.000 | 0.962 | 0.896 | 0.943 | 0.996 | 0.977 | 0.707 | 1.000 | 1.000 | 0.938 |
| 153 | 0.775 | 1.000 | 1.000 | 0.966 | 0.646 | 1.000 | 1.000 | 0.940 | 0.678 | 1.000 | 1.000 | 0.962 | 0.896 | 0.943 | 0.996 | 0.977 | 0.707 | 1.000 | 1.000 | 0.938 |
| 154 | 0.788 | 1.000 | 1.000 | 0.968 | 0.633 | 1.000 | 1.000 | 0.938 | 0.678 | 1.000 | 1.000 | 0.962 | 0.890 | 0.943 | 0.996 | 0.976 | 0.691 | 1.000 | 1.000 | 0.935 |
| 155 | 0.788 | 1.000 | 1.000 | 0.968 | 0.633 | 1.000 | 1.000 | 0.938 | 0.678 | 1.000 | 1.000 | 0.962 | 0.890 | 0.943 | 0.996 | 0.976 | 0.697 | 1.000 | 1.000 | 0.936 |
| 156 | 0.788 | 1.000 | 1.000 | 0.968 | 0.633 | 1.000 | 1.000 | 0.938 | 0.678 | 1.000 | 1.000 | 0.962 | 0.890 | 0.943 | 0.996 | 0.976 | 0.691 | 1.000 | 1.000 | 0.935 |
| 157 | 0.788 | 1.000 | 1.000 | 0.968 | 0.646 | 1.000 | 1.000 | 0.940 | 0.678 | 1.000 | 1.000 | 0.962 | 0.883 | 0.943 | 0.996 | 0.975 | 0.676 | 1.000 | 1.000 | 0.932 |
| 158 | 0.788 | 1.000 | 1.000 | 0.968 | 0.646 | 1.000 | 1.000 | 0.940 | 0.695 | 1.000 | 1.000 | 0.964 | 0.883 | 0.943 | 0.996 | 0.975 | 0.681 | 1.000 | 1.000 | 0.933 |
| 159 | 0.800 | 1.000 | 1.000 | 0.970 | 0.646 | 1.000 | 1.000 | 0.940 | 0.695 | 1.000 | 1.000 | 0.964 | 0.896 | 0.943 | 0.993 | 0.975 | 0.686 | 1.000 | 1.000 | 0.934 |
| 160 | 0.800 | 1.000 | 1.000 | 0.970 | 0.646 | 1.000 | 1.000 | 0.940 | 0.695 | 1.000 | 1.000 | 0.964 | 0.890 | 0.943 | 0.995 | 0.975 | 0.691 | 1.000 | 1.000 | 0.935 |
| 161 | 0.788 | 1.000 | 1.000 | 0.968 | 0.646 | 1.000 | 1.000 | 0.940 | 0.695 | 1.000 | 1.000 | 0.964 | 0.890 | 0.943 | 0.995 | 0.975 | 0.697 | 1.000 | 1.000 | 0.936 |
| 162 | 0.800 | 1.000 | 1.000 | 0.970 | 0.646 | 1.000 | 1.000 | 0.940 | 0.695 | 0.988 | 1.000 | 0.962 | 0.890 | 0.906 | 0.995 | 0.973 | 0.702 | 1.000 | 1.000 | 0.937 |
| 163 | 0.788 | 1.000 | 1.000 | 0.968 | 0.646 | 1.000 | 1.000 | 0.940 | 0.695 | 0.988 | 1.000 | 0.962 | 0.896 | 0.906 | 0.993 | 0.973 | 0.697 | 1.000 | 1.000 | 0.936 |
| 164 | 0.775 | 1.000 | 1.000 | 0.966 | 0.646 | 1.000 | 1.000 | 0.940 | 0.695 | 0.988 | 1.000 | 0.962 | 0.896 | 0.906 | 0.993 | 0.973 | 0.697 | 1.000 | 1.000 | 0.936 |
| 165 | 0.775 | 1.000 | 1.000 | 0.966 | 0.646 | 1.000 | 1.000 | 0.940 | 0.678 | 0.988 | 1.000 | 0.960 | 0.896 | 0.906 | 0.993 | 0.973 | 0.697 | 1.000 | 1.000 | 0.936 |
| 166 | 0.775 | 1.000 | 1.000 | 0.966 | 0.646 | 1.000 | 1.000 | 0.940 | 0.678 | 0.988 | 1.000 | 0.960 | 0.896 | 0.906 | 0.993 | 0.973 | 0.697 | 1.000 | 1.000 | 0.936 |
| 167 | 0.775 | 1.000 | 1.000 | 0.966 | 0.646 | 1.000 | 1.000 | 0.940 | 0.678 | 0.988 | 1.000 | 0.960 | 0.896 | 0.906 | 0.993 | 0.973 | 0.697 | 1.000 | 1.000 | 0.936 |
| 168 | 0.775 | 1.000 | 1.000 | 0.966 | 0.633 | 1.000 | 1.000 | 0.938 | 0.678 | 0.988 | 1.000 | 0.960 | 0.909 | 0.906 | 0.992 | 0.974 | 0.702 | 1.000 | 1.000 | 0.937 |
| 169 | 0.775 | 1.000 | 1.000 | 0.966 | 0.646 | 1.000 | 1.000 | 0.940 | 0.678 | 0.988 | 1.000 | 0.960 | 0.896 | 0.906 | 0.992 | 0.971 | 0.697 | 1.000 | 1.000 | 0.936 |
| 170 | 0.775 | 1.000 | 1.000 | 0.966 | 0.646 | 1.000 | 1.000 | 0.940 | 0.678 | 0.988 | 1.000 | 0.960 | 0.896 | 0.906 | 0.992 | 0.971 | 0.697 | 1.000 | 1.000 | 0.936 |
| 171 | 0.788 | 1.000 | 1.000 | 0.968 | 0.646 | 1.000 | 1.000 | 0.940 | 0.678 | 0.988 | 1.000 | 0.960 | 0.903 | 0.906 | 0.992 | 0.973 | 0.702 | 1.000 | 1.000 | 0.937 |
| 172 | 0.775 | 1.000 | 1.000 | 0.966 | 0.646 | 1.000 | 1.000 | 0.940 | 0.678 | 1.000 | 1.000 | 0.962 | 0.896 | 0.906 | 0.992 | 0.971 | 0.707 | 1.000 | 1.000 | 0.938 |
| 173 | 0.775 | 1.000 | 1.000 | 0.966 | 0.646 | 1.000 | 1.000 | 0.940 | 0.678 | 1.000 | 1.000 | 0.962 | 0.896 | 0.906 | 0.992 | 0.971 | 0.707 | 1.000 | 1.000 | 0.938 |
| 174 | 0.775 | 1.000 | 1.000 | 0.966 | 0.646 | 1.000 | 1.000 | 0.940 | 0.678 | 1.000 | 1.000 | 0.962 | 0.896 | 0.906 | 0.992 | 0.971 | 0.713 | 1.000 | 1.000 | 0.940 |
| 175 | 0.788 | 1.000 | 1.000 | 0.968 | 0.646 | 1.000 | 1.000 | 0.940 | 0.678 | 1.000 | 1.000 | 0.962 | 0.896 | 0.906 | 0.992 | 0.971 | 0.713 | 1.000 | 1.000 | 0.940 |
| 176 | 0.788 | 1.000 | 1.000 | 0.968 | 0.646 | 1.000 | 1.000 | 0.940 | 0.678 | 1.000 | 1.000 | 0.962 | 0.890 | 0.906 | 0.992 | 0.970 | 0.718 | 1.000 | 1.000 | 0.941 |
| 177 | 0.800 | 1.000 | 1.000 | 0.970 | 0.646 | 1.000 | 1.000 | 0.940 | 0.678 | 0.988 | 1.000 | 0.960 | 0.890 | 0.906 | 0.992 | 0.970 | 0.713 | 1.000 | 1.000 | 0.940 |
| 178 | 0.788 | 1.000 | 1.000 | 0.968 | 0.646 | 1.000 | 1.000 | 0.940 | 0.661 | 0.988 | 1.000 | 0.958 | 0.896 | 0.906 | 0.992 | 0.971 | 0.707 | 1.000 | 1.000 | 0.938 |
| 179 | 0.788 | 1.000 | 1.000 | 0.968 | 0.646 | 1.000 | 1.000 | 0.940 | 0.678 | 0.988 | 1.000 | 0.960 | 0.896 | 0.906 | 0.992 | 0.971 | 0.707 | 1.000 | 1.000 | 0.938 |
| 180 | 0.788 | 1.000 | 1.000 | 0.968 | 0.608 | 1.000 | 1.000 | 0.934 | 0.644 | 0.988 | 1.000 | 0.956 | 0.896 | 0.906 | 0.993 | 0.973 | 0.691 | 1.000 | 1.000 | 0.935 |
| 181 | 0.788 | 1.000 | 1.000 | 0.968 | 0.633 | 1.000 | 1.000 | 0.938 | 0.644 | 1.000 | 1.000 | 0.958 | 0.896 | 0.906 | 0.995 | 0.974 | 0.676 | 1.000 | 1.000 | 0.932 |
| 182 | 0.788 | 1.000 | 1.000 | 0.968 | 0.633 | 1.000 | 1.000 | 0.938 | 0.644 | 1.000 | 1.000 | 0.958 | 0.890 | 0.906 | 0.995 | 0.973 | 0.691 | 1.000 | 1.000 | 0.935 |
| 183 | 0.788 | 1.000 | 1.000 | 0.968 | 0.633 | 1.000 | 1.000 | 0.938 | 0.644 | 1.000 | 1.000 | 0.958 | 0.890 | 0.906 | 0.995 | 0.973 | 0.686 | 1.000 | 1.000 | 0.934 |
| 184 | 0.788 | 1.000 | 1.000 | 0.968 | 0.633 | 1.000 | 1.000 | 0.938 | 0.644 | 1.000 | 1.000 | 0.958 | 0.896 | 0.906 | 0.995 | 0.974 | 0.691 | 1.000 | 1.000 | 0.935 |
| 185 | 0.788 | 1.000 | 1.000 | 0.968 | 0.633 | 1.000 | 1.000 | 0.938 | 0.627 | 1.000 | 1.000 | 0.956 | 0.890 | 0.906 | 0.995 | 0.973 | 0.686 | 1.000 | 1.000 | 0.934 |
| 186 | 0.788 | 1.000 | 1.000 | 0.968 | 0.633 | 1.000 | 1.000 | 0.938 | 0.627 | 1.000 | 1.000 | 0.956 | 0.890 | 0.906 | 0.995 | 0.973 | 0.691 | 1.000 | 1.000 | 0.935 |
| 187 | 0.788 | 1.000 | 1.000 | 0.968 | 0.633 | 1.000 | 1.000 | 0.938 | 0.627 | 1.000 | 1.000 | 0.956 | 0.896 | 0.906 | 0.995 | 0.974 | 0.686 | 1.000 | 1.000 | 0.934 |
| 188 | 0.788 | 1.000 | 1.000 | 0.968 | 0.633 | 1.000 | 1.000 | 0.938 | 0.627 | 1.000 | 1.000 | 0.956 | 0.890 | 0.906 | 0.995 | 0.973 | 0.697 | 1.000 | 1.000 | 0.936 |
| 189 | 0.788 | 1.000 | 1.000 | 0.968 | 0.620 | 1.000 | 1.000 | 0.936 | 0.627 | 1.000 | 1.000 | 0.956 | 0.896 | 0.906 | 0.995 | 0.974 | 0.681 | 1.000 | 1.000 | 0.933 |
| 190 | 0.775 | 1.000 | 1.000 | 0.966 | 0.620 | 1.000 | 1.000 | 0.936 | 0.644 | 0.988 | 1.000 | 0.956 | 0.896 | 0.906 | 0.995 | 0.974 | 0.681 | 1.000 | 1.000 | 0.933 |
| 191 | 0.775 | 1.000 | 1.000 | 0.966 | 0.608 | 1.000 | 1.000 | 0.934 | 0.644 | 0.988 | 1.000 | 0.956 | 0.896 | 0.906 | 0.995 | 0.974 | 0.686 | 1.000 | 1.000 | 0.934 |
| 192 | 0.775 | 1.000 | 1.000 | 0.966 | 0.608 | 1.000 | 1.000 | 0.934 | 0.627 | 1.000 | 1.000 | 0.956 | 0.883 | 0.906 | 0.995 | 0.971 | 0.660 | 1.000 | 1.000 | 0.928 |
| 193 | 0.775 | 1.000 | 1.000 | 0.966 | 0.608 | 1.000 | 1.000 | 0.934 | 0.627 | 1.000 | 1.000 | 0.956 | 0.883 | 0.906 | 0.995 | 0.971 | 0.660 | 1.000 | 1.000 | 0.928 |
| 194 | 0.775 | 1.000 | 1.000 | 0.966 | 0.608 | 1.000 | 1.000 | 0.934 | 0.627 | 1.000 | 1.000 | 0.956 | 0.883 | 0.906 | 0.995 | 0.971 | 0.660 | 1.000 | 1.000 | 0.928 |
| 195 | 0.775 | 1.000 | 1.000 | 0.966 | 0.608 | 1.000 | 1.000 | 0.934 | 0.644 | 1.000 | 1.000 | 0.958 | 0.883 | 0.906 | 0.995 | 0.971 | 0.670 | 1.000 | 1.000 | 0.931 |
| 196 | 0.775 | 1.000 | 1.000 | 0.966 | 0.608 | 1.000 | 1.000 | 0.934 | 0.644 | 1.000 | 1.000 | 0.958 | 0.883 | 0.906 | 0.995 | 0.971 | 0.670 | 1.000 | 1.000 | 0.931 |
| 197 | 0.800 | 1.000 | 1.000 | 0.970 | 0.608 | 1.000 | 1.000 | 0.934 | 0.644 | 1.000 | 1.000 | 0.958 | 0.877 | 0.906 | 0.995 | 0.970 | 0.686 | 1.000 | 1.000 | 0.934 |
| 198 | 0.800 | 1.000 | 1.000 | 0.970 | 0.608 | 1.000 | 1.000 | 0.934 | 0.644 | 1.000 | 1.000 | 0.958 | 0.877 | 0.906 | 0.995 | 0.970 | 0.691 | 1.000 | 1.000 | 0.935 |
| 199 | 0.813 | 1.000 | 1.000 | 0.972 | 0.608 | 1.000 | 1.000 | 0.934 | 0.644 | 1.000 | 1.000 | 0.958 | 0.890 | 0.906 | 0.995 | 0.973 | 0.697 | 1.000 | 1.000 | 0.936 |
| 200 | 0.800 | 1.000 | 1.000 | 0.970 | 0.608 | 1.000 | 1.000 | 0.934 | 0.644 | 1.000 | 1.000 | 0.958 | 0.896 | 0.906 | 0.995 | 0.974 | 0.713 | 1.000 | 1.000 | 0.940 |
| 201 | 0.800 | 1.000 | 1.000 | 0.970 | 0.608 | 1.000 | 1.000 | 0.934 | 0.644 | 1.000 | 1.000 | 0.958 | 0.896 | 0.906 | 0.995 | 0.974 | 0.702 | 1.000 | 1.000 | 0.937 |
| 202 | 0.800 | 1.000 | 1.000 | 0.970 | 0.608 | 1.000 | 1.000 | 0.934 | 0.644 | 1.000 | 1.000 | 0.958 | 0.896 | 0.906 | 0.995 | 0.974 | 0.686 | 1.000 | 1.000 | 0.934 |
| 203 | 0.800 | 1.000 | 1.000 | 0.970 | 0.608 | 1.000 | 1.000 | 0.934 | 0.644 | 1.000 | 1.000 | 0.958 | 0.903 | 0.906 | 0.995 | 0.975 | 0.697 | 1.000 | 1.000 | 0.936 |
| 204 | 0.800 | 1.000 | 1.000 | 0.970 | 0.608 | 1.000 | 1.000 | 0.934 | 0.610 | 1.000 | 1.000 | 0.954 | 0.883 | 0.906 | 0.993 | 0.970 | 0.707 | 1.000 | 1.000 | 0.938 |
| 205 | 0.813 | 1.000 | 1.000 | 0.972 | 0.608 | 1.000 | 1.000 | 0.934 | 0.610 | 1.000 | 1.000 | 0.954 | 0.883 | 0.906 | 0.993 | 0.970 | 0.707 | 1.000 | 1.000 | 0.938 |
| 206 | 0.813 | 1.000 | 1.000 | 0.972 | 0.608 | 1.000 | 1.000 | 0.934 | 0.610 | 1.000 | 1.000 | 0.954 | 0.883 | 0.906 | 0.993 | 0.970 | 0.707 | 1.000 | 1.000 | 0.938 |
| 207 | 0.813 | 1.000 | 1.000 | 0.972 | 0.608 | 1.000 | 1.000 | 0.934 | 0.610 | 1.000 | 1.000 | 0.954 | 0.883 | 0.906 | 0.993 | 0.970 | 0.702 | 1.000 | 1.000 | 0.937 |
| 208 | 0.813 | 1.000 | 1.000 | 0.972 | 0.608 | 1.000 | 1.000 | 0.934 | 0.610 | 1.000 | 1.000 | 0.954 | 0.883 | 0.906 | 0.993 | 0.970 | 0.697 | 1.000 | 1.000 | 0.936 |
| 209 | 0.800 | 1.000 | 1.000 | 0.970 | 0.608 | 1.000 | 1.000 | 0.934 | 0.610 | 1.000 | 1.000 | 0.954 | 0.883 | 0.906 | 0.995 | 0.971 | 0.681 | 1.000 | 1.000 | 0.933 |
| 210 | 0.775 | 1.000 | 1.000 | 0.966 | 0.608 | 1.000 | 1.000 | 0.934 | 0.610 | 1.000 | 1.000 | 0.954 | 0.883 | 0.906 | 0.995 | 0.971 | 0.665 | 1.000 | 1.000 | 0.929 |
| 211 | 0.775 | 1.000 | 1.000 | 0.966 | 0.608 | 1.000 | 1.000 | 0.934 | 0.610 | 1.000 | 1.000 | 0.954 | 0.883 | 0.906 | 0.995 | 0.971 | 0.670 | 1.000 | 1.000 | 0.931 |
| 212 | 0.775 | 1.000 | 1.000 | 0.966 | 0.595 | 1.000 | 1.000 | 0.932 | 0.610 | 1.000 | 1.000 | 0.954 | 0.883 | 0.906 | 0.995 | 0.971 | 0.665 | 1.000 | 1.000 | 0.929 |
| 213 | 0.775 | 1.000 | 1.000 | 0.966 | 0.595 | 1.000 | 1.000 | 0.932 | 0.593 | 1.000 | 1.000 | 0.952 | 0.883 | 0.906 | 0.995 | 0.971 | 0.665 | 1.000 | 1.000 | 0.929 |
| 214 | 0.775 | 1.000 | 1.000 | 0.966 | 0.608 | 1.000 | 1.000 | 0.934 | 0.627 | 0.988 | 1.000 | 0.954 | 0.883 | 0.906 | 0.995 | 0.971 | 0.676 | 1.000 | 1.000 | 0.932 |
| 215 | 0.775 | 1.000 | 1.000 | 0.966 | 0.608 | 1.000 | 1.000 | 0.934 | 0.627 | 0.988 | 1.000 | 0.954 | 0.883 | 0.906 | 0.995 | 0.971 | 0.670 | 1.000 | 1.000 | 0.931 |
| 216 | 0.775 | 1.000 | 1.000 | 0.966 | 0.608 | 1.000 | 1.000 | 0.934 | 0.627 | 0.988 | 1.000 | 0.954 | 0.883 | 0.906 | 0.995 | 0.971 | 0.654 | 1.000 | 1.000 | 0.927 |
| 217 | 0.775 | 1.000 | 1.000 | 0.966 | 0.608 | 1.000 | 1.000 | 0.934 | 0.627 | 0.988 | 1.000 | 0.954 | 0.883 | 0.906 | 0.995 | 0.971 | 0.660 | 1.000 | 1.000 | 0.928 |
| 218 | 0.775 | 1.000 | 1.000 | 0.966 | 0.608 | 1.000 | 1.000 | 0.934 | 0.627 | 0.988 | 1.000 | 0.954 | 0.883 | 0.906 | 0.995 | 0.971 | 0.670 | 1.000 | 1.000 | 0.931 |
| 219 | 0.775 | 1.000 | 1.000 | 0.966 | 0.608 | 1.000 | 1.000 | 0.934 | 0.627 | 0.988 | 1.000 | 0.954 | 0.883 | 0.906 | 0.995 | 0.971 | 0.670 | 1.000 | 1.000 | 0.931 |
| 220 | 0.788 | 1.000 | 1.000 | 0.968 | 0.608 | 1.000 | 1.000 | 0.934 | 0.644 | 0.988 | 1.000 | 0.956 | 0.883 | 0.906 | 0.995 | 0.971 | 0.670 | 1.000 | 1.000 | 0.931 |
| 221 | 0.813 | 1.000 | 1.000 | 0.972 | 0.633 | 1.000 | 1.000 | 0.938 | 0.644 | 0.988 | 1.000 | 0.956 | 0.883 | 0.906 | 0.993 | 0.970 | 0.729 | 1.000 | 1.000 | 0.943 |
| 222 | 0.813 | 1.000 | 1.000 | 0.972 | 0.633 | 1.000 | 1.000 | 0.938 | 0.644 | 0.988 | 1.000 | 0.956 | 0.890 | 0.906 | 0.993 | 0.971 | 0.739 | 1.000 | 1.000 | 0.945 |
| 223 | 0.813 | 1.000 | 1.000 | 0.972 | 0.646 | 1.000 | 1.000 | 0.940 | 0.644 | 1.000 | 1.000 | 0.958 | 0.890 | 0.906 | 0.993 | 0.971 | 0.739 | 1.000 | 1.000 | 0.945 |
| 224 | 0.813 | 1.000 | 1.000 | 0.972 | 0.633 | 1.000 | 1.000 | 0.938 | 0.644 | 1.000 | 1.000 | 0.958 | 0.890 | 0.906 | 0.993 | 0.971 | 0.734 | 1.000 | 1.000 | 0.944 |
| 225 | 0.813 | 1.000 | 1.000 | 0.972 | 0.633 | 1.000 | 1.000 | 0.938 | 0.644 | 1.000 | 1.000 | 0.958 | 0.890 | 0.906 | 0.993 | 0.971 | 0.734 | 1.000 | 1.000 | 0.944 |
| 226 | 0.813 | 1.000 | 1.000 | 0.972 | 0.633 | 1.000 | 1.000 | 0.938 | 0.644 | 1.000 | 1.000 | 0.958 | 0.896 | 0.906 | 0.993 | 0.973 | 0.739 | 1.000 | 1.000 | 0.945 |
| 227 | 0.813 | 1.000 | 1.000 | 0.972 | 0.633 | 1.000 | 1.000 | 0.938 | 0.644 | 1.000 | 1.000 | 0.958 | 0.890 | 0.906 | 0.993 | 0.971 | 0.745 | 1.000 | 1.000 | 0.946 |
| 228 | 0.813 | 1.000 | 1.000 | 0.972 | 0.633 | 1.000 | 1.000 | 0.938 | 0.644 | 1.000 | 1.000 | 0.958 | 0.890 | 0.906 | 0.993 | 0.971 | 0.745 | 1.000 | 1.000 | 0.946 |
| 229 | 0.813 | 1.000 | 1.000 | 0.972 | 0.633 | 1.000 | 1.000 | 0.938 | 0.644 | 1.000 | 1.000 | 0.958 | 0.883 | 0.906 | 0.993 | 0.970 | 0.734 | 1.000 | 1.000 | 0.944 |
| 230 | 0.813 | 1.000 | 1.000 | 0.972 | 0.633 | 1.000 | 1.000 | 0.938 | 0.644 | 1.000 | 1.000 | 0.958 | 0.877 | 0.906 | 0.993 | 0.969 | 0.713 | 1.000 | 1.000 | 0.940 |
| 231 | 0.813 | 1.000 | 1.000 | 0.972 | 0.633 | 1.000 | 1.000 | 0.938 | 0.644 | 1.000 | 1.000 | 0.958 | 0.870 | 0.906 | 0.993 | 0.968 | 0.707 | 1.000 | 1.000 | 0.938 |
| 232 | 0.813 | 1.000 | 1.000 | 0.972 | 0.633 | 1.000 | 1.000 | 0.938 | 0.644 | 1.000 | 1.000 | 0.958 | 0.877 | 0.906 | 0.993 | 0.969 | 0.707 | 1.000 | 1.000 | 0.938 |
| 233 | 0.813 | 1.000 | 1.000 | 0.972 | 0.620 | 1.000 | 1.000 | 0.936 | 0.644 | 1.000 | 1.000 | 0.958 | 0.870 | 0.906 | 0.993 | 0.968 | 0.702 | 1.000 | 1.000 | 0.937 |
| 234 | 0.800 | 1.000 | 1.000 | 0.970 | 0.620 | 1.000 | 1.000 | 0.936 | 0.644 | 1.000 | 1.000 | 0.958 | 0.864 | 0.906 | 0.993 | 0.967 | 0.691 | 1.000 | 1.000 | 0.935 |
| 235 | 0.800 | 1.000 | 1.000 | 0.970 | 0.620 | 1.000 | 1.000 | 0.936 | 0.644 | 1.000 | 1.000 | 0.958 | 0.864 | 0.906 | 0.993 | 0.967 | 0.691 | 1.000 | 1.000 | 0.935 |
| 236 | 0.800 | 1.000 | 1.000 | 0.970 | 0.620 | 1.000 | 1.000 | 0.936 | 0.644 | 1.000 | 1.000 | 0.958 | 0.864 | 0.906 | 0.993 | 0.967 | 0.691 | 1.000 | 1.000 | 0.935 |
| 237 | 0.800 | 1.000 | 1.000 | 0.970 | 0.620 | 1.000 | 1.000 | 0.936 | 0.644 | 1.000 | 1.000 | 0.958 | 0.864 | 0.906 | 0.993 | 0.967 | 0.691 | 1.000 | 1.000 | 0.935 |
| 238 | 0.800 | 1.000 | 1.000 | 0.970 | 0.608 | 1.000 | 1.000 | 0.934 | 0.644 | 1.000 | 1.000 | 0.958 | 0.857 | 0.906 | 0.993 | 0.966 | 0.665 | 1.000 | 1.000 | 0.929 |
| 239 | 0.800 | 1.000 | 1.000 | 0.970 | 0.608 | 1.000 | 1.000 | 0.934 | 0.661 | 0.988 | 1.000 | 0.958 | 0.857 | 0.906 | 0.993 | 0.966 | 0.660 | 1.000 | 1.000 | 0.928 |
| 240 | 0.800 | 1.000 | 1.000 | 0.970 | 0.582 | 1.000 | 1.000 | 0.930 | 0.644 | 0.988 | 1.000 | 0.956 | 0.857 | 0.906 | 0.995 | 0.967 | 0.660 | 1.000 | 1.000 | 0.928 |
| 241 | 0.775 | 1.000 | 1.000 | 0.966 | 0.582 | 1.000 | 1.000 | 0.930 | 0.644 | 0.988 | 1.000 | 0.956 | 0.857 | 0.906 | 0.995 | 0.967 | 0.660 | 1.000 | 1.000 | 0.928 |
| 242 | 0.775 | 1.000 | 1.000 | 0.966 | 0.582 | 1.000 | 1.000 | 0.930 | 0.644 | 0.988 | 1.000 | 0.956 | 0.864 | 0.906 | 0.995 | 0.968 | 0.660 | 1.000 | 1.000 | 0.928 |
| 243 | 0.800 | 1.000 | 1.000 | 0.970 | 0.595 | 1.000 | 1.000 | 0.932 | 0.644 | 0.988 | 1.000 | 0.956 | 0.857 | 0.906 | 0.995 | 0.967 | 0.660 | 1.000 | 1.000 | 0.928 |
| 244 | 0.775 | 1.000 | 1.000 | 0.966 | 0.595 | 1.000 | 1.000 | 0.932 | 0.644 | 0.988 | 1.000 | 0.956 | 0.857 | 0.906 | 0.995 | 0.967 | 0.654 | 1.000 | 1.000 | 0.927 |
| 245 | 0.775 | 1.000 | 1.000 | 0.966 | 0.595 | 1.000 | 1.000 | 0.932 | 0.644 | 0.988 | 1.000 | 0.956 | 0.864 | 0.906 | 0.995 | 0.968 | 0.654 | 1.000 | 1.000 | 0.927 |
| 246 | 0.775 | 1.000 | 1.000 | 0.966 | 0.595 | 1.000 | 1.000 | 0.932 | 0.644 | 0.988 | 1.000 | 0.956 | 0.864 | 0.906 | 0.995 | 0.968 | 0.654 | 1.000 | 1.000 | 0.927 |
| 247 | 0.775 | 1.000 | 1.000 | 0.966 | 0.595 | 1.000 | 1.000 | 0.932 | 0.644 | 0.988 | 1.000 | 0.956 | 0.864 | 0.906 | 0.995 | 0.968 | 0.654 | 1.000 | 1.000 | 0.927 |
| 248 | 0.788 | 1.000 | 1.000 | 0.968 | 0.582 | 1.000 | 1.000 | 0.930 | 0.644 | 0.988 | 1.000 | 0.956 | 0.844 | 0.906 | 0.995 | 0.965 | 0.649 | 1.000 | 1.000 | 0.926 |
| 249 | 0.788 | 1.000 | 1.000 | 0.968 | 0.582 | 1.000 | 1.000 | 0.930 | 0.644 | 0.988 | 1.000 | 0.956 | 0.844 | 0.906 | 0.995 | 0.965 | 0.649 | 1.000 | 1.000 | 0.926 |
| 250 | 0.788 | 1.000 | 1.000 | 0.968 | 0.582 | 1.000 | 1.000 | 0.930 | 0.644 | 0.988 | 1.000 | 0.956 | 0.838 | 0.906 | 0.995 | 0.964 | 0.649 | 1.000 | 1.000 | 0.926 |
| 251 | 0.788 | 1.000 | 1.000 | 0.968 | 0.582 | 1.000 | 1.000 | 0.930 | 0.644 | 0.988 | 1.000 | 0.956 | 0.838 | 0.906 | 0.995 | 0.964 | 0.649 | 1.000 | 1.000 | 0.926 |
| 252 | 0.800 | 1.000 | 1.000 | 0.970 | 0.582 | 1.000 | 1.000 | 0.930 | 0.644 | 0.988 | 1.000 | 0.956 | 0.844 | 0.906 | 0.995 | 0.965 | 0.654 | 1.000 | 1.000 | 0.927 |
| 253 | 0.800 | 1.000 | 1.000 | 0.970 | 0.582 | 1.000 | 1.000 | 0.930 | 0.644 | 0.988 | 1.000 | 0.956 | 0.844 | 0.906 | 0.995 | 0.965 | 0.654 | 1.000 | 1.000 | 0.927 |
| 254 | 0.800 | 1.000 | 1.000 | 0.970 | 0.582 | 1.000 | 1.000 | 0.930 | 0.644 | 0.988 | 1.000 | 0.956 | 0.844 | 0.906 | 0.995 | 0.965 | 0.660 | 1.000 | 1.000 | 0.928 |
| 255 | 0.800 | 1.000 | 1.000 | 0.970 | 0.582 | 1.000 | 1.000 | 0.930 | 0.644 | 0.988 | 1.000 | 0.956 | 0.844 | 0.906 | 0.995 | 0.965 | 0.654 | 1.000 | 1.000 | 0.927 |
| 256 | 0.788 | 1.000 | 1.000 | 0.968 | 0.570 | 1.000 | 1.000 | 0.928 | 0.644 | 0.988 | 1.000 | 0.956 | 0.838 | 0.906 | 0.995 | 0.964 | 0.649 | 1.000 | 1.000 | 0.926 |
| 257 | 0.788 | 1.000 | 1.000 | 0.968 | 0.582 | 1.000 | 1.000 | 0.930 | 0.644 | 0.988 | 1.000 | 0.956 | 0.831 | 0.906 | 0.995 | 0.963 | 0.649 | 1.000 | 1.000 | 0.926 |
| 258 | 0.788 | 1.000 | 1.000 | 0.968 | 0.582 | 1.000 | 1.000 | 0.930 | 0.661 | 0.988 | 1.000 | 0.958 | 0.838 | 0.906 | 0.995 | 0.964 | 0.649 | 1.000 | 1.000 | 0.926 |
| 259 | 0.788 | 1.000 | 1.000 | 0.968 | 0.582 | 1.000 | 1.000 | 0.930 | 0.661 | 0.988 | 1.000 | 0.958 | 0.838 | 0.906 | 0.995 | 0.964 | 0.649 | 1.000 | 1.000 | 0.926 |
| 260 | 0.788 | 1.000 | 1.000 | 0.968 | 0.582 | 1.000 | 1.000 | 0.930 | 0.661 | 0.988 | 1.000 | 0.958 | 0.838 | 0.906 | 0.995 | 0.964 | 0.654 | 1.000 | 1.000 | 0.927 |
| 261 | 0.788 | 1.000 | 1.000 | 0.968 | 0.582 | 1.000 | 1.000 | 0.930 | 0.661 | 0.988 | 1.000 | 0.958 | 0.831 | 0.906 | 0.995 | 0.963 | 0.654 | 1.000 | 1.000 | 0.927 |
| 262 | 0.788 | 1.000 | 1.000 | 0.968 | 0.570 | 1.000 | 1.000 | 0.928 | 0.661 | 0.988 | 1.000 | 0.958 | 0.831 | 0.906 | 0.995 | 0.963 | 0.649 | 1.000 | 1.000 | 0.926 |
| 263 | 0.788 | 1.000 | 1.000 | 0.968 | 0.570 | 1.000 | 1.000 | 0.928 | 0.661 | 0.988 | 1.000 | 0.958 | 0.831 | 0.906 | 0.995 | 0.963 | 0.654 | 1.000 | 1.000 | 0.927 |
| 264 | 0.788 | 1.000 | 1.000 | 0.968 | 0.570 | 1.000 | 1.000 | 0.928 | 0.661 | 0.988 | 1.000 | 0.958 | 0.831 | 0.887 | 0.995 | 0.962 | 0.670 | 1.000 | 1.000 | 0.931 |
| 265 | 0.788 | 1.000 | 1.000 | 0.968 | 0.570 | 1.000 | 1.000 | 0.928 | 0.661 | 0.988 | 1.000 | 0.958 | 0.831 | 0.887 | 0.995 | 0.962 | 0.670 | 1.000 | 1.000 | 0.931 |
| 266 | 0.788 | 1.000 | 1.000 | 0.968 | 0.570 | 1.000 | 1.000 | 0.928 | 0.661 | 0.988 | 1.000 | 0.958 | 0.831 | 0.906 | 0.995 | 0.963 | 0.660 | 1.000 | 1.000 | 0.928 |
| 267 | 0.788 | 1.000 | 1.000 | 0.968 | 0.570 | 1.000 | 1.000 | 0.928 | 0.661 | 0.988 | 1.000 | 0.958 | 0.818 | 0.906 | 0.995 | 0.961 | 0.633 | 1.000 | 1.000 | 0.923 |
| 268 | 0.788 | 1.000 | 1.000 | 0.968 | 0.570 | 1.000 | 1.000 | 0.928 | 0.661 | 0.988 | 1.000 | 0.958 | 0.825 | 0.906 | 0.995 | 0.962 | 0.633 | 1.000 | 1.000 | 0.923 |
| 269 | 0.788 | 1.000 | 1.000 | 0.968 | 0.570 | 1.000 | 1.000 | 0.928 | 0.661 | 0.988 | 1.000 | 0.958 | 0.818 | 0.906 | 0.995 | 0.961 | 0.638 | 1.000 | 1.000 | 0.924 |
| 270 | 0.788 | 1.000 | 1.000 | 0.968 | 0.570 | 1.000 | 1.000 | 0.928 | 0.661 | 0.988 | 1.000 | 0.958 | 0.825 | 0.906 | 0.995 | 0.962 | 0.649 | 1.000 | 1.000 | 0.926 |
| 271 | 0.788 | 1.000 | 1.000 | 0.968 | 0.570 | 1.000 | 1.000 | 0.928 | 0.661 | 0.988 | 1.000 | 0.958 | 0.818 | 0.906 | 0.995 | 0.961 | 0.649 | 1.000 | 1.000 | 0.926 |
| 272 | 0.788 | 1.000 | 1.000 | 0.968 | 0.582 | 1.000 | 1.000 | 0.930 | 0.661 | 0.988 | 1.000 | 0.958 | 0.825 | 0.906 | 0.995 | 0.962 | 0.649 | 1.000 | 1.000 | 0.926 |
| 273 | 0.788 | 1.000 | 1.000 | 0.968 | 0.582 | 1.000 | 1.000 | 0.930 | 0.661 | 0.988 | 1.000 | 0.958 | 0.825 | 0.906 | 0.995 | 0.962 | 0.649 | 1.000 | 1.000 | 0.926 |
| 274 | 0.775 | 1.000 | 1.000 | 0.966 | 0.582 | 1.000 | 1.000 | 0.930 | 0.661 | 0.988 | 1.000 | 0.958 | 0.825 | 0.906 | 0.995 | 0.962 | 0.649 | 1.000 | 1.000 | 0.926 |
| 275 | 0.788 | 1.000 | 1.000 | 0.968 | 0.570 | 1.000 | 1.000 | 0.928 | 0.678 | 0.988 | 1.000 | 0.960 | 0.812 | 0.906 | 0.995 | 0.960 | 0.649 | 1.000 | 1.000 | 0.926 |
| 276 | 0.788 | 1.000 | 1.000 | 0.968 | 0.570 | 1.000 | 1.000 | 0.928 | 0.678 | 0.988 | 1.000 | 0.960 | 0.812 | 0.906 | 0.995 | 0.960 | 0.649 | 1.000 | 1.000 | 0.926 |
| 277 | 0.788 | 1.000 | 1.000 | 0.968 | 0.582 | 1.000 | 1.000 | 0.930 | 0.678 | 0.988 | 1.000 | 0.960 | 0.812 | 0.906 | 0.995 | 0.960 | 0.644 | 1.000 | 1.000 | 0.925 |
| 278 | 0.788 | 1.000 | 1.000 | 0.968 | 0.595 | 1.000 | 1.000 | 0.932 | 0.678 | 0.988 | 1.000 | 0.960 | 0.812 | 0.906 | 0.995 | 0.960 | 0.660 | 1.000 | 1.000 | 0.928 |
| 279 | 0.788 | 1.000 | 1.000 | 0.968 | 0.608 | 1.000 | 1.000 | 0.934 | 0.678 | 0.988 | 1.000 | 0.960 | 0.812 | 0.906 | 0.996 | 0.961 | 0.665 | 1.000 | 1.000 | 0.929 |
| 280 | 0.788 | 1.000 | 1.000 | 0.968 | 0.595 | 1.000 | 1.000 | 0.932 | 0.678 | 0.988 | 1.000 | 0.960 | 0.812 | 0.906 | 0.996 | 0.961 | 0.660 | 1.000 | 1.000 | 0.928 |
| 281 | 0.788 | 1.000 | 1.000 | 0.968 | 0.595 | 1.000 | 1.000 | 0.932 | 0.678 | 0.988 | 1.000 | 0.960 | 0.812 | 0.906 | 0.996 | 0.961 | 0.665 | 1.000 | 1.000 | 0.929 |
| 282 | 0.813 | 1.000 | 1.000 | 0.972 | 0.595 | 1.000 | 1.000 | 0.932 | 0.678 | 0.988 | 1.000 | 0.960 | 0.812 | 0.887 | 0.996 | 0.960 | 0.670 | 1.000 | 1.000 | 0.931 |
| 283 | 0.813 | 1.000 | 1.000 | 0.972 | 0.608 | 1.000 | 1.000 | 0.934 | 0.678 | 0.988 | 1.000 | 0.960 | 0.812 | 0.906 | 0.996 | 0.961 | 0.670 | 1.000 | 1.000 | 0.931 |
| 284 | 0.813 | 1.000 | 1.000 | 0.972 | 0.608 | 1.000 | 1.000 | 0.934 | 0.678 | 0.988 | 1.000 | 0.960 | 0.812 | 0.906 | 0.996 | 0.961 | 0.670 | 1.000 | 1.000 | 0.931 |
| 285 | 0.813 | 1.000 | 1.000 | 0.972 | 0.608 | 1.000 | 1.000 | 0.934 | 0.678 | 0.988 | 1.000 | 0.960 | 0.812 | 0.887 | 0.996 | 0.960 | 0.665 | 1.000 | 1.000 | 0.929 |
| 286 | 0.813 | 1.000 | 1.000 | 0.972 | 0.595 | 1.000 | 1.000 | 0.932 | 0.678 | 0.988 | 1.000 | 0.960 | 0.812 | 0.906 | 0.996 | 0.961 | 0.665 | 1.000 | 1.000 | 0.929 |
| 287 | 0.813 | 1.000 | 1.000 | 0.972 | 0.608 | 1.000 | 1.000 | 0.934 | 0.695 | 0.988 | 1.000 | 0.962 | 0.812 | 0.906 | 0.996 | 0.961 | 0.665 | 1.000 | 1.000 | 0.929 |
| 288 | 0.813 | 1.000 | 1.000 | 0.972 | 0.608 | 1.000 | 1.000 | 0.934 | 0.695 | 0.988 | 1.000 | 0.962 | 0.831 | 0.906 | 0.995 | 0.963 | 0.707 | 0.988 | 1.000 | 0.937 |
| 289 | 0.800 | 1.000 | 1.000 | 0.970 | 0.595 | 1.000 | 1.000 | 0.932 | 0.695 | 0.988 | 1.000 | 0.962 | 0.831 | 0.906 | 0.995 | 0.963 | 0.702 | 0.988 | 1.000 | 0.936 |
| 290 | 0.800 | 1.000 | 1.000 | 0.970 | 0.595 | 1.000 | 1.000 | 0.932 | 0.695 | 0.988 | 1.000 | 0.962 | 0.831 | 0.906 | 0.995 | 0.963 | 0.702 | 0.988 | 1.000 | 0.936 |
| 291 | 0.800 | 1.000 | 1.000 | 0.970 | 0.595 | 1.000 | 1.000 | 0.932 | 0.695 | 0.988 | 1.000 | 0.962 | 0.838 | 0.906 | 0.995 | 0.964 | 0.707 | 0.988 | 1.000 | 0.937 |
| 292 | 0.800 | 1.000 | 1.000 | 0.970 | 0.595 | 1.000 | 1.000 | 0.932 | 0.695 | 0.988 | 1.000 | 0.962 | 0.838 | 0.906 | 0.995 | 0.964 | 0.707 | 0.988 | 1.000 | 0.937 |
| 293 | 0.800 | 1.000 | 1.000 | 0.970 | 0.595 | 1.000 | 1.000 | 0.932 | 0.695 | 0.988 | 1.000 | 0.962 | 0.838 | 0.906 | 0.995 | 0.964 | 0.702 | 0.988 | 1.000 | 0.936 |
| 294 | 0.800 | 1.000 | 1.000 | 0.970 | 0.608 | 1.000 | 1.000 | 0.934 | 0.695 | 0.988 | 1.000 | 0.962 | 0.831 | 0.887 | 0.995 | 0.962 | 0.702 | 0.988 | 1.000 | 0.936 |
| 295 | 0.800 | 1.000 | 1.000 | 0.970 | 0.595 | 1.000 | 1.000 | 0.932 | 0.695 | 0.988 | 1.000 | 0.962 | 0.831 | 0.887 | 0.995 | 0.962 | 0.702 | 0.988 | 1.000 | 0.936 |
| 296 | 0.800 | 1.000 | 1.000 | 0.970 | 0.608 | 1.000 | 1.000 | 0.934 | 0.695 | 0.988 | 1.000 | 0.962 | 0.825 | 0.887 | 0.995 | 0.961 | 0.707 | 0.988 | 1.000 | 0.937 |
| 297 | 0.800 | 1.000 | 1.000 | 0.970 | 0.595 | 1.000 | 1.000 | 0.932 | 0.695 | 0.988 | 1.000 | 0.962 | 0.831 | 0.887 | 0.995 | 0.962 | 0.707 | 0.988 | 1.000 | 0.937 |
| 298 | 0.800 | 1.000 | 1.000 | 0.970 | 0.608 | 1.000 | 1.000 | 0.934 | 0.695 | 0.988 | 1.000 | 0.962 | 0.825 | 0.887 | 0.995 | 0.961 | 0.697 | 0.988 | 1.000 | 0.935 |
| 299 | 0.800 | 1.000 | 1.000 | 0.970 | 0.608 | 1.000 | 1.000 | 0.934 | 0.695 | 0.988 | 1.000 | 0.962 | 0.818 | 0.887 | 0.995 | 0.960 | 0.691 | 0.988 | 1.000 | 0.934 |
| 300 | 0.800 | 1.000 | 1.000 | 0.970 | 0.608 | 1.000 | 1.000 | 0.934 | 0.695 | 0.988 | 1.000 | 0.962 | 0.818 | 0.887 | 0.995 | 0.960 | 0.691 | 0.988 | 1.000 | 0.934 |
| 301 | 0.800 | 1.000 | 1.000 | 0.970 | 0.608 | 1.000 | 1.000 | 0.934 | 0.695 | 0.988 | 1.000 | 0.962 | 0.825 | 0.887 | 0.995 | 0.961 | 0.697 | 0.988 | 1.000 | 0.935 |
| 302 | 0.800 | 1.000 | 1.000 | 0.970 | 0.608 | 1.000 | 1.000 | 0.934 | 0.695 | 0.988 | 1.000 | 0.962 | 0.818 | 0.887 | 0.995 | 0.960 | 0.697 | 0.988 | 1.000 | 0.935 |
| 303 | 0.800 | 1.000 | 1.000 | 0.970 | 0.608 | 1.000 | 1.000 | 0.934 | 0.695 | 0.988 | 1.000 | 0.962 | 0.818 | 0.887 | 0.995 | 0.960 | 0.702 | 1.000 | 1.000 | 0.937 |
| 304 | 0.800 | 1.000 | 1.000 | 0.970 | 0.608 | 1.000 | 1.000 | 0.934 | 0.695 | 0.988 | 1.000 | 0.962 | 0.818 | 0.887 | 0.995 | 0.960 | 0.702 | 1.000 | 1.000 | 0.937 |
| 305 | 0.800 | 1.000 | 1.000 | 0.970 | 0.608 | 1.000 | 1.000 | 0.934 | 0.695 | 0.988 | 1.000 | 0.962 | 0.818 | 0.887 | 0.995 | 0.960 | 0.702 | 1.000 | 1.000 | 0.937 |
| 306 | 0.800 | 1.000 | 1.000 | 0.970 | 0.608 | 1.000 | 1.000 | 0.934 | 0.695 | 0.988 | 1.000 | 0.962 | 0.818 | 0.887 | 0.995 | 0.960 | 0.702 | 1.000 | 1.000 | 0.937 |
| 307 | 0.800 | 1.000 | 1.000 | 0.970 | 0.608 | 1.000 | 1.000 | 0.934 | 0.695 | 0.988 | 1.000 | 0.962 | 0.818 | 0.887 | 0.995 | 0.960 | 0.702 | 1.000 | 1.000 | 0.937 |
| 308 | 0.800 | 1.000 | 1.000 | 0.970 | 0.608 | 1.000 | 1.000 | 0.934 | 0.695 | 0.988 | 1.000 | 0.962 | 0.825 | 0.887 | 0.995 | 0.961 | 0.702 | 1.000 | 1.000 | 0.937 |
| 309 | 0.800 | 1.000 | 1.000 | 0.970 | 0.608 | 1.000 | 1.000 | 0.934 | 0.695 | 0.988 | 1.000 | 0.962 | 0.818 | 0.887 | 0.995 | 0.960 | 0.702 | 1.000 | 1.000 | 0.937 |
| 310 | 0.800 | 1.000 | 1.000 | 0.970 | 0.608 | 1.000 | 1.000 | 0.934 | 0.695 | 0.988 | 1.000 | 0.962 | 0.825 | 0.887 | 0.995 | 0.961 | 0.702 | 1.000 | 1.000 | 0.937 |
| 311 | 0.800 | 1.000 | 1.000 | 0.970 | 0.608 | 1.000 | 1.000 | 0.934 | 0.695 | 0.988 | 1.000 | 0.962 | 0.818 | 0.887 | 0.995 | 0.960 | 0.702 | 1.000 | 1.000 | 0.937 |
| 312 | 0.800 | 1.000 | 1.000 | 0.970 | 0.608 | 1.000 | 1.000 | 0.934 | 0.695 | 0.988 | 1.000 | 0.962 | 0.825 | 0.887 | 0.995 | 0.961 | 0.702 | 1.000 | 1.000 | 0.937 |
| 313 | 0.800 | 1.000 | 1.000 | 0.970 | 0.608 | 1.000 | 1.000 | 0.934 | 0.695 | 0.988 | 1.000 | 0.962 | 0.818 | 0.887 | 0.995 | 0.960 | 0.707 | 1.000 | 1.000 | 0.938 |
| 314 | 0.800 | 1.000 | 1.000 | 0.970 | 0.608 | 1.000 | 1.000 | 0.934 | 0.695 | 0.988 | 1.000 | 0.962 | 0.825 | 0.887 | 0.995 | 0.961 | 0.707 | 1.000 | 1.000 | 0.938 |
| 315 | 0.800 | 1.000 | 1.000 | 0.970 | 0.608 | 1.000 | 1.000 | 0.934 | 0.695 | 0.988 | 1.000 | 0.962 | 0.818 | 0.887 | 0.995 | 0.960 | 0.702 | 1.000 | 1.000 | 0.937 |
| 316 | 0.800 | 1.000 | 1.000 | 0.970 | 0.608 | 1.000 | 1.000 | 0.934 | 0.695 | 0.988 | 1.000 | 0.962 | 0.825 | 0.887 | 0.995 | 0.961 | 0.702 | 1.000 | 1.000 | 0.937 |
| 317 | 0.788 | 1.000 | 1.000 | 0.968 | 0.582 | 1.000 | 1.000 | 0.930 | 0.695 | 0.988 | 1.000 | 0.962 | 0.818 | 0.887 | 0.995 | 0.960 | 0.681 | 1.000 | 1.000 | 0.933 |
| 318 | 0.800 | 1.000 | 1.000 | 0.970 | 0.570 | 1.000 | 1.000 | 0.928 | 0.695 | 0.988 | 1.000 | 0.962 | 0.818 | 0.887 | 0.995 | 0.960 | 0.686 | 1.000 | 1.000 | 0.934 |
| 319 | 0.775 | 1.000 | 1.000 | 0.966 | 0.595 | 1.000 | 1.000 | 0.932 | 0.695 | 0.988 | 1.000 | 0.962 | 0.818 | 0.887 | 0.995 | 0.960 | 0.670 | 1.000 | 1.000 | 0.931 |
| 320 | 0.763 | 1.000 | 1.000 | 0.964 | 0.557 | 1.000 | 1.000 | 0.926 | 0.678 | 0.988 | 1.000 | 0.960 | 0.818 | 0.887 | 0.995 | 0.960 | 0.670 | 1.000 | 1.000 | 0.931 |
| 321 | 0.788 | 1.000 | 1.000 | 0.968 | 0.582 | 1.000 | 1.000 | 0.930 | 0.695 | 0.988 | 1.000 | 0.962 | 0.818 | 0.887 | 0.995 | 0.960 | 0.681 | 1.000 | 1.000 | 0.933 |
| 322 | 0.788 | 1.000 | 1.000 | 0.968 | 0.595 | 1.000 | 1.000 | 0.932 | 0.695 | 0.988 | 1.000 | 0.962 | 0.812 | 0.887 | 0.995 | 0.959 | 0.681 | 1.000 | 1.000 | 0.933 |
| 323 | 0.788 | 1.000 | 1.000 | 0.968 | 0.595 | 1.000 | 1.000 | 0.932 | 0.712 | 0.988 | 1.000 | 0.964 | 0.818 | 0.887 | 0.995 | 0.960 | 0.686 | 1.000 | 1.000 | 0.934 |
| 324 | 0.775 | 1.000 | 1.000 | 0.966 | 0.595 | 1.000 | 1.000 | 0.932 | 0.695 | 0.988 | 1.000 | 0.962 | 0.812 | 0.887 | 0.995 | 0.959 | 0.681 | 1.000 | 1.000 | 0.933 |
| 325 | 0.763 | 1.000 | 1.000 | 0.964 | 0.570 | 1.000 | 1.000 | 0.928 | 0.695 | 0.988 | 1.000 | 0.962 | 0.805 | 0.887 | 0.995 | 0.958 | 0.676 | 1.000 | 1.000 | 0.932 |
| 326 | 0.763 | 1.000 | 1.000 | 0.964 | 0.582 | 1.000 | 1.000 | 0.930 | 0.695 | 0.988 | 1.000 | 0.962 | 0.812 | 0.887 | 0.995 | 0.959 | 0.681 | 1.000 | 1.000 | 0.933 |
| 327 | 0.763 | 1.000 | 1.000 | 0.964 | 0.582 | 1.000 | 1.000 | 0.930 | 0.695 | 0.988 | 1.000 | 0.962 | 0.812 | 0.887 | 0.995 | 0.959 | 0.681 | 1.000 | 1.000 | 0.933 |
| 328 | 0.763 | 1.000 | 1.000 | 0.964 | 0.582 | 1.000 | 1.000 | 0.930 | 0.695 | 0.988 | 1.000 | 0.962 | 0.805 | 0.887 | 0.995 | 0.958 | 0.686 | 1.000 | 1.000 | 0.934 |
| 329 | 0.788 | 1.000 | 1.000 | 0.968 | 0.570 | 1.000 | 1.000 | 0.928 | 0.712 | 0.988 | 1.000 | 0.964 | 0.805 | 0.887 | 0.995 | 0.958 | 0.697 | 1.000 | 1.000 | 0.936 |
| 330 | 0.788 | 1.000 | 1.000 | 0.968 | 0.582 | 1.000 | 1.000 | 0.930 | 0.695 | 0.988 | 1.000 | 0.962 | 0.805 | 0.887 | 0.995 | 0.958 | 0.697 | 1.000 | 1.000 | 0.936 |
| 331 | 0.800 | 1.000 | 1.000 | 0.970 | 0.570 | 1.000 | 1.000 | 0.928 | 0.695 | 0.988 | 1.000 | 0.962 | 0.805 | 0.887 | 0.995 | 0.958 | 0.681 | 1.000 | 1.000 | 0.933 |
| 332 | 0.800 | 1.000 | 1.000 | 0.970 | 0.595 | 1.000 | 1.000 | 0.932 | 0.695 | 0.988 | 1.000 | 0.962 | 0.805 | 0.887 | 0.995 | 0.958 | 0.676 | 1.000 | 1.000 | 0.932 |
| 333 | 0.775 | 1.000 | 1.000 | 0.966 | 0.595 | 1.000 | 1.000 | 0.932 | 0.695 | 0.988 | 1.000 | 0.962 | 0.812 | 0.887 | 0.995 | 0.959 | 0.691 | 0.988 | 1.000 | 0.934 |
| 334 | 0.788 | 1.000 | 1.000 | 0.968 | 0.582 | 1.000 | 1.000 | 0.930 | 0.695 | 0.988 | 1.000 | 0.962 | 0.805 | 0.887 | 0.995 | 0.958 | 0.686 | 0.988 | 1.000 | 0.933 |
| 335 | 0.775 | 1.000 | 1.000 | 0.966 | 0.582 | 1.000 | 1.000 | 0.930 | 0.695 | 0.988 | 1.000 | 0.962 | 0.805 | 0.887 | 0.995 | 0.958 | 0.676 | 1.000 | 1.000 | 0.932 |
| 336 | 0.775 | 1.000 | 1.000 | 0.966 | 0.582 | 1.000 | 1.000 | 0.930 | 0.695 | 0.988 | 1.000 | 0.962 | 0.805 | 0.887 | 0.995 | 0.958 | 0.676 | 1.000 | 1.000 | 0.932 |
| 337 | 0.775 | 1.000 | 1.000 | 0.966 | 0.582 | 1.000 | 1.000 | 0.930 | 0.695 | 0.988 | 1.000 | 0.962 | 0.805 | 0.887 | 0.995 | 0.958 | 0.676 | 1.000 | 1.000 | 0.932 |
| 338 | 0.775 | 1.000 | 1.000 | 0.966 | 0.582 | 1.000 | 1.000 | 0.930 | 0.695 | 0.988 | 1.000 | 0.962 | 0.805 | 0.887 | 0.995 | 0.958 | 0.676 | 1.000 | 1.000 | 0.932 |
| 339 | 0.775 | 1.000 | 1.000 | 0.966 | 0.595 | 1.000 | 1.000 | 0.932 | 0.695 | 0.988 | 1.000 | 0.962 | 0.799 | 0.887 | 0.995 | 0.957 | 0.676 | 1.000 | 1.000 | 0.932 |
| 340 | 0.775 | 1.000 | 1.000 | 0.966 | 0.595 | 1.000 | 1.000 | 0.932 | 0.695 | 0.988 | 1.000 | 0.962 | 0.799 | 0.887 | 0.995 | 0.957 | 0.676 | 1.000 | 1.000 | 0.932 |
| 341 | 0.775 | 1.000 | 1.000 | 0.966 | 0.595 | 1.000 | 1.000 | 0.932 | 0.695 | 0.988 | 1.000 | 0.962 | 0.799 | 0.887 | 0.995 | 0.957 | 0.670 | 1.000 | 1.000 | 0.931 |
| 342 | 0.775 | 1.000 | 1.000 | 0.966 | 0.595 | 1.000 | 1.000 | 0.932 | 0.695 | 0.988 | 1.000 | 0.962 | 0.799 | 0.887 | 0.995 | 0.957 | 0.670 | 1.000 | 1.000 | 0.931 |
| 343 | 0.788 | 1.000 | 1.000 | 0.968 | 0.595 | 1.000 | 1.000 | 0.932 | 0.695 | 0.988 | 1.000 | 0.962 | 0.799 | 0.887 | 0.996 | 0.958 | 0.686 | 1.000 | 1.000 | 0.934 |
| 344 | 0.788 | 1.000 | 1.000 | 0.968 | 0.595 | 1.000 | 1.000 | 0.932 | 0.695 | 0.988 | 1.000 | 0.962 | 0.792 | 0.887 | 0.996 | 0.957 | 0.686 | 1.000 | 1.000 | 0.934 |
| 345 | 0.788 | 1.000 | 1.000 | 0.968 | 0.595 | 1.000 | 1.000 | 0.932 | 0.695 | 0.988 | 1.000 | 0.962 | 0.792 | 0.887 | 0.996 | 0.957 | 0.681 | 1.000 | 1.000 | 0.933 |
| 346 | 0.788 | 1.000 | 1.000 | 0.968 | 0.595 | 1.000 | 1.000 | 0.932 | 0.695 | 0.988 | 1.000 | 0.962 | 0.792 | 0.887 | 0.996 | 0.957 | 0.681 | 1.000 | 1.000 | 0.933 |
| 347 | 0.788 | 1.000 | 1.000 | 0.968 | 0.595 | 1.000 | 1.000 | 0.932 | 0.695 | 0.988 | 1.000 | 0.962 | 0.792 | 0.887 | 0.996 | 0.957 | 0.686 | 1.000 | 1.000 | 0.934 |
| 348 | 0.788 | 1.000 | 1.000 | 0.968 | 0.595 | 1.000 | 1.000 | 0.932 | 0.695 | 0.988 | 1.000 | 0.962 | 0.799 | 0.887 | 0.995 | 0.957 | 0.686 | 1.000 | 1.000 | 0.934 |
| 349 | 0.788 | 1.000 | 1.000 | 0.968 | 0.595 | 1.000 | 1.000 | 0.932 | 0.695 | 0.988 | 1.000 | 0.962 | 0.805 | 0.887 | 0.995 | 0.958 | 0.686 | 1.000 | 1.000 | 0.934 |
| 350 | 0.788 | 1.000 | 1.000 | 0.968 | 0.595 | 1.000 | 1.000 | 0.932 | 0.695 | 0.988 | 1.000 | 0.962 | 0.805 | 0.887 | 0.995 | 0.958 | 0.686 | 1.000 | 1.000 | 0.934 |
| 351 | 0.788 | 1.000 | 1.000 | 0.968 | 0.595 | 1.000 | 1.000 | 0.932 | 0.695 | 0.988 | 1.000 | 0.962 | 0.799 | 0.887 | 0.995 | 0.957 | 0.686 | 1.000 | 1.000 | 0.934 |
| 352 | 0.788 | 1.000 | 1.000 | 0.968 | 0.595 | 1.000 | 1.000 | 0.932 | 0.695 | 0.988 | 1.000 | 0.962 | 0.805 | 0.887 | 0.995 | 0.958 | 0.691 | 1.000 | 1.000 | 0.935 |
| 353 | 0.788 | 1.000 | 1.000 | 0.968 | 0.582 | 1.000 | 1.000 | 0.930 | 0.695 | 0.988 | 1.000 | 0.962 | 0.805 | 0.887 | 0.995 | 0.958 | 0.681 | 1.000 | 1.000 | 0.933 |
| 354 | 0.788 | 1.000 | 1.000 | 0.968 | 0.595 | 1.000 | 1.000 | 0.932 | 0.695 | 0.988 | 1.000 | 0.962 | 0.792 | 0.887 | 0.995 | 0.956 | 0.686 | 1.000 | 1.000 | 0.934 |
| 355 | 0.788 | 1.000 | 1.000 | 0.968 | 0.582 | 1.000 | 1.000 | 0.930 | 0.695 | 0.988 | 1.000 | 0.962 | 0.792 | 0.887 | 0.995 | 0.956 | 0.686 | 1.000 | 1.000 | 0.934 |
| 356 | 0.788 | 1.000 | 1.000 | 0.968 | 0.582 | 1.000 | 1.000 | 0.930 | 0.695 | 0.988 | 1.000 | 0.962 | 0.792 | 0.887 | 0.995 | 0.956 | 0.686 | 1.000 | 1.000 | 0.934 |
| 357 | 0.788 | 1.000 | 1.000 | 0.968 | 0.582 | 1.000 | 1.000 | 0.930 | 0.695 | 0.988 | 1.000 | 0.962 | 0.792 | 0.887 | 0.995 | 0.956 | 0.686 | 1.000 | 1.000 | 0.934 |
| 358 | 0.788 | 1.000 | 1.000 | 0.968 | 0.582 | 1.000 | 1.000 | 0.930 | 0.678 | 0.988 | 1.000 | 0.960 | 0.786 | 0.887 | 0.996 | 0.956 | 0.686 | 1.000 | 1.000 | 0.934 |
| 359 | 0.788 | 1.000 | 1.000 | 0.968 | 0.582 | 1.000 | 1.000 | 0.930 | 0.678 | 0.988 | 1.000 | 0.960 | 0.786 | 0.887 | 0.996 | 0.956 | 0.686 | 1.000 | 1.000 | 0.934 |
| 360 | 0.788 | 1.000 | 1.000 | 0.968 | 0.582 | 1.000 | 1.000 | 0.930 | 0.695 | 0.988 | 1.000 | 0.962 | 0.792 | 0.887 | 0.995 | 0.956 | 0.686 | 1.000 | 1.000 | 0.934 |
| 361 | 0.788 | 1.000 | 1.000 | 0.968 | 0.582 | 1.000 | 1.000 | 0.930 | 0.695 | 0.988 | 1.000 | 0.962 | 0.792 | 0.887 | 0.996 | 0.957 | 0.686 | 1.000 | 1.000 | 0.934 |
| 362 | 0.788 | 1.000 | 1.000 | 0.968 | 0.582 | 1.000 | 1.000 | 0.930 | 0.678 | 0.988 | 1.000 | 0.960 | 0.792 | 0.887 | 0.995 | 0.956 | 0.686 | 1.000 | 1.000 | 0.934 |
| 363 | 0.788 | 1.000 | 1.000 | 0.968 | 0.582 | 1.000 | 1.000 | 0.930 | 0.678 | 0.988 | 1.000 | 0.960 | 0.792 | 0.887 | 0.995 | 0.956 | 0.686 | 1.000 | 1.000 | 0.934 |
| 364 | 0.788 | 1.000 | 1.000 | 0.968 | 0.582 | 1.000 | 1.000 | 0.930 | 0.678 | 0.988 | 1.000 | 0.960 | 0.792 | 0.887 | 0.995 | 0.956 | 0.686 | 1.000 | 1.000 | 0.934 |
| 365 | 0.788 | 1.000 | 1.000 | 0.968 | 0.582 | 1.000 | 1.000 | 0.930 | 0.678 | 0.988 | 1.000 | 0.960 | 0.792 | 0.887 | 0.995 | 0.956 | 0.681 | 1.000 | 1.000 | 0.933 |
| 366 | 0.788 | 1.000 | 1.000 | 0.968 | 0.582 | 1.000 | 1.000 | 0.930 | 0.678 | 0.988 | 1.000 | 0.960 | 0.792 | 0.887 | 0.995 | 0.956 | 0.686 | 1.000 | 1.000 | 0.934 |
| 367 | 0.800 | 1.000 | 1.000 | 0.970 | 0.582 | 1.000 | 1.000 | 0.930 | 0.678 | 0.988 | 1.000 | 0.960 | 0.792 | 0.887 | 0.995 | 0.956 | 0.691 | 1.000 | 1.000 | 0.935 |
| 368 | 0.788 | 1.000 | 1.000 | 0.968 | 0.582 | 1.000 | 1.000 | 0.930 | 0.678 | 0.988 | 1.000 | 0.960 | 0.792 | 0.887 | 0.995 | 0.956 | 0.676 | 1.000 | 1.000 | 0.932 |
| 369 | 0.788 | 1.000 | 1.000 | 0.968 | 0.582 | 1.000 | 1.000 | 0.930 | 0.678 | 0.988 | 1.000 | 0.960 | 0.792 | 0.887 | 0.995 | 0.956 | 0.681 | 1.000 | 1.000 | 0.933 |
| 370 | 0.788 | 1.000 | 1.000 | 0.968 | 0.582 | 1.000 | 1.000 | 0.930 | 0.678 | 0.988 | 1.000 | 0.960 | 0.792 | 0.887 | 0.995 | 0.956 | 0.670 | 1.000 | 1.000 | 0.931 |
| 371 | 0.775 | 1.000 | 1.000 | 0.966 | 0.582 | 1.000 | 1.000 | 0.930 | 0.678 | 0.988 | 1.000 | 0.960 | 0.792 | 0.887 | 0.995 | 0.956 | 0.660 | 1.000 | 1.000 | 0.928 |
| 372 | 0.763 | 1.000 | 1.000 | 0.964 | 0.595 | 1.000 | 1.000 | 0.932 | 0.678 | 0.988 | 1.000 | 0.960 | 0.792 | 0.887 | 0.995 | 0.956 | 0.660 | 1.000 | 1.000 | 0.928 |
| 373 | 0.763 | 1.000 | 1.000 | 0.964 | 0.595 | 1.000 | 1.000 | 0.932 | 0.678 | 0.988 | 1.000 | 0.960 | 0.792 | 0.887 | 0.995 | 0.956 | 0.660 | 1.000 | 1.000 | 0.928 |
| 374 | 0.775 | 1.000 | 1.000 | 0.966 | 0.595 | 1.000 | 1.000 | 0.932 | 0.678 | 0.988 | 1.000 | 0.960 | 0.792 | 0.887 | 0.995 | 0.956 | 0.660 | 1.000 | 1.000 | 0.928 |
| 375 | 0.775 | 1.000 | 1.000 | 0.966 | 0.582 | 1.000 | 1.000 | 0.930 | 0.678 | 0.988 | 1.000 | 0.960 | 0.792 | 0.887 | 0.995 | 0.956 | 0.665 | 1.000 | 1.000 | 0.929 |
| 376 | 0.775 | 1.000 | 1.000 | 0.966 | 0.582 | 1.000 | 1.000 | 0.930 | 0.678 | 0.988 | 1.000 | 0.960 | 0.792 | 0.887 | 0.995 | 0.956 | 0.665 | 1.000 | 1.000 | 0.929 |
| 377 | 0.775 | 1.000 | 1.000 | 0.966 | 0.582 | 1.000 | 1.000 | 0.930 | 0.678 | 0.988 | 1.000 | 0.960 | 0.792 | 0.887 | 0.995 | 0.956 | 0.670 | 1.000 | 1.000 | 0.931 |
| 378 | 0.775 | 1.000 | 1.000 | 0.966 | 0.582 | 1.000 | 1.000 | 0.930 | 0.678 | 0.988 | 1.000 | 0.960 | 0.792 | 0.887 | 0.995 | 0.956 | 0.670 | 1.000 | 1.000 | 0.931 |
| 379 | 0.775 | 1.000 | 1.000 | 0.966 | 0.582 | 1.000 | 1.000 | 0.930 | 0.695 | 0.988 | 1.000 | 0.962 | 0.792 | 0.868 | 0.995 | 0.955 | 0.670 | 1.000 | 1.000 | 0.931 |
| 380 | 0.775 | 1.000 | 1.000 | 0.966 | 0.595 | 1.000 | 1.000 | 0.932 | 0.695 | 0.988 | 1.000 | 0.962 | 0.792 | 0.868 | 0.996 | 0.956 | 0.670 | 1.000 | 1.000 | 0.931 |
| 381 | 0.775 | 1.000 | 1.000 | 0.966 | 0.595 | 1.000 | 1.000 | 0.932 | 0.695 | 0.988 | 1.000 | 0.962 | 0.792 | 0.868 | 0.996 | 0.956 | 0.670 | 1.000 | 1.000 | 0.931 |
| 382 | 0.775 | 1.000 | 1.000 | 0.966 | 0.595 | 1.000 | 1.000 | 0.932 | 0.695 | 0.988 | 1.000 | 0.962 | 0.792 | 0.868 | 0.996 | 0.956 | 0.670 | 1.000 | 1.000 | 0.931 |
| 383 | 0.775 | 1.000 | 1.000 | 0.966 | 0.570 | 1.000 | 1.000 | 0.928 | 0.695 | 0.988 | 1.000 | 0.962 | 0.792 | 0.868 | 0.997 | 0.957 | 0.665 | 1.000 | 1.000 | 0.929 |
| 384 | 0.775 | 1.000 | 1.000 | 0.966 | 0.582 | 1.000 | 1.000 | 0.930 | 0.695 | 0.988 | 1.000 | 0.962 | 0.792 | 0.868 | 0.995 | 0.955 | 0.665 | 1.000 | 1.000 | 0.929 |
| 385 | 0.775 | 1.000 | 1.000 | 0.966 | 0.582 | 1.000 | 1.000 | 0.930 | 0.678 | 0.988 | 1.000 | 0.960 | 0.792 | 0.868 | 0.995 | 0.955 | 0.670 | 1.000 | 1.000 | 0.931 |
| 386 | 0.775 | 1.000 | 1.000 | 0.966 | 0.582 | 1.000 | 1.000 | 0.930 | 0.695 | 0.988 | 1.000 | 0.962 | 0.792 | 0.868 | 0.995 | 0.955 | 0.670 | 1.000 | 1.000 | 0.931 |
| 387 | 0.775 | 1.000 | 1.000 | 0.966 | 0.582 | 1.000 | 1.000 | 0.930 | 0.695 | 0.988 | 1.000 | 0.962 | 0.792 | 0.849 | 0.995 | 0.953 | 0.670 | 1.000 | 1.000 | 0.931 |
| 388 | 0.775 | 1.000 | 1.000 | 0.966 | 0.570 | 1.000 | 1.000 | 0.928 | 0.695 | 0.988 | 1.000 | 0.962 | 0.792 | 0.849 | 0.996 | 0.955 | 0.665 | 1.000 | 1.000 | 0.929 |
| 389 | 0.775 | 1.000 | 1.000 | 0.966 | 0.570 | 1.000 | 1.000 | 0.928 | 0.695 | 0.988 | 1.000 | 0.962 | 0.792 | 0.849 | 0.996 | 0.955 | 0.670 | 1.000 | 1.000 | 0.931 |
| 390 | 0.775 | 1.000 | 1.000 | 0.966 | 0.570 | 1.000 | 1.000 | 0.928 | 0.695 | 0.988 | 1.000 | 0.962 | 0.792 | 0.849 | 0.996 | 0.955 | 0.665 | 1.000 | 1.000 | 0.929 |
| 391 | 0.763 | 1.000 | 1.000 | 0.964 | 0.557 | 1.000 | 1.000 | 0.926 | 0.695 | 0.988 | 1.000 | 0.962 | 0.792 | 0.849 | 0.996 | 0.955 | 0.670 | 1.000 | 1.000 | 0.931 |
| 392 | 0.763 | 1.000 | 1.000 | 0.964 | 0.557 | 1.000 | 1.000 | 0.926 | 0.695 | 0.988 | 1.000 | 0.962 | 0.792 | 0.849 | 0.996 | 0.955 | 0.670 | 1.000 | 1.000 | 0.931 |
| 393 | 0.763 | 1.000 | 1.000 | 0.964 | 0.557 | 1.000 | 1.000 | 0.926 | 0.695 | 0.988 | 1.000 | 0.962 | 0.786 | 0.849 | 0.996 | 0.953 | 0.654 | 1.000 | 1.000 | 0.927 |
| 394 | 0.800 | 1.000 | 1.000 | 0.970 | 0.570 | 1.000 | 1.000 | 0.928 | 0.695 | 0.988 | 1.000 | 0.962 | 0.792 | 0.849 | 0.996 | 0.955 | 0.660 | 1.000 | 1.000 | 0.928 |
| 395 | 0.800 | 1.000 | 1.000 | 0.970 | 0.570 | 1.000 | 1.000 | 0.928 | 0.695 | 0.988 | 1.000 | 0.962 | 0.792 | 0.849 | 0.996 | 0.955 | 0.660 | 1.000 | 1.000 | 0.928 |
| 396 | 0.800 | 1.000 | 1.000 | 0.970 | 0.557 | 1.000 | 1.000 | 0.926 | 0.695 | 0.988 | 1.000 | 0.962 | 0.792 | 0.849 | 0.996 | 0.955 | 0.660 | 1.000 | 1.000 | 0.928 |
| 397 | 0.788 | 1.000 | 1.000 | 0.968 | 0.557 | 1.000 | 1.000 | 0.926 | 0.695 | 0.988 | 1.000 | 0.962 | 0.792 | 0.849 | 0.996 | 0.955 | 0.660 | 1.000 | 1.000 | 0.928 |
| 398 | 0.788 | 1.000 | 1.000 | 0.968 | 0.570 | 1.000 | 1.000 | 0.928 | 0.695 | 0.988 | 1.000 | 0.962 | 0.792 | 0.849 | 0.997 | 0.956 | 0.654 | 1.000 | 1.000 | 0.927 |
| 399 | 0.788 | 1.000 | 1.000 | 0.968 | 0.570 | 1.000 | 1.000 | 0.928 | 0.695 | 0.988 | 1.000 | 0.962 | 0.792 | 0.849 | 0.997 | 0.956 | 0.654 | 1.000 | 1.000 | 0.927 |
| 400 | 0.775 | 1.000 | 1.000 | 0.966 | 0.557 | 1.000 | 1.000 | 0.926 | 0.695 | 0.988 | 1.000 | 0.962 | 0.799 | 0.849 | 0.997 | 0.957 | 0.654 | 1.000 | 1.000 | 0.927 |
| 401 | 0.775 | 1.000 | 1.000 | 0.966 | 0.557 | 1.000 | 1.000 | 0.926 | 0.695 | 0.988 | 1.000 | 0.962 | 0.799 | 0.849 | 0.997 | 0.957 | 0.654 | 1.000 | 1.000 | 0.927 |
| 402 | 0.750 | 1.000 | 1.000 | 0.962 | 0.544 | 1.000 | 1.000 | 0.923 | 0.695 | 0.988 | 1.000 | 0.962 | 0.799 | 0.849 | 0.996 | 0.956 | 0.654 | 1.000 | 1.000 | 0.927 |
| 403 | 0.763 | 1.000 | 1.000 | 0.964 | 0.557 | 1.000 | 1.000 | 0.926 | 0.678 | 0.988 | 1.000 | 0.960 | 0.799 | 0.849 | 0.995 | 0.955 | 0.654 | 1.000 | 1.000 | 0.927 |
| 404 | 0.750 | 1.000 | 1.000 | 0.962 | 0.570 | 1.000 | 1.000 | 0.928 | 0.695 | 0.988 | 1.000 | 0.962 | 0.792 | 0.849 | 0.995 | 0.953 | 0.654 | 1.000 | 1.000 | 0.927 |
| 405 | 0.763 | 1.000 | 1.000 | 0.964 | 0.570 | 1.000 | 1.000 | 0.928 | 0.678 | 0.988 | 1.000 | 0.960 | 0.792 | 0.849 | 0.995 | 0.953 | 0.654 | 1.000 | 1.000 | 0.927 |
| 406 | 0.763 | 1.000 | 1.000 | 0.964 | 0.570 | 1.000 | 1.000 | 0.928 | 0.695 | 0.988 | 1.000 | 0.962 | 0.792 | 0.849 | 0.995 | 0.953 | 0.660 | 1.000 | 1.000 | 0.928 |
| 407 | 0.738 | 1.000 | 1.000 | 0.960 | 0.570 | 1.000 | 1.000 | 0.928 | 0.695 | 0.988 | 1.000 | 0.962 | 0.792 | 0.849 | 0.995 | 0.953 | 0.660 | 1.000 | 1.000 | 0.928 |
| 408 | 0.750 | 1.000 | 1.000 | 0.962 | 0.557 | 1.000 | 1.000 | 0.926 | 0.695 | 0.988 | 1.000 | 0.962 | 0.792 | 0.849 | 0.995 | 0.953 | 0.665 | 1.000 | 1.000 | 0.929 |
| 409 | 0.750 | 1.000 | 1.000 | 0.962 | 0.557 | 1.000 | 1.000 | 0.926 | 0.695 | 0.988 | 1.000 | 0.962 | 0.792 | 0.849 | 0.997 | 0.956 | 0.660 | 1.000 | 1.000 | 0.928 |
| 410 | 0.738 | 1.000 | 1.000 | 0.960 | 0.557 | 1.000 | 1.000 | 0.926 | 0.678 | 0.988 | 1.000 | 0.960 | 0.792 | 0.849 | 0.996 | 0.955 | 0.660 | 1.000 | 1.000 | 0.928 |
| 411 | 0.738 | 1.000 | 1.000 | 0.960 | 0.557 | 1.000 | 1.000 | 0.926 | 0.678 | 0.988 | 1.000 | 0.960 | 0.792 | 0.849 | 0.996 | 0.955 | 0.660 | 1.000 | 1.000 | 0.928 |
| 412 | 0.725 | 1.000 | 1.000 | 0.958 | 0.557 | 1.000 | 1.000 | 0.926 | 0.678 | 0.988 | 1.000 | 0.960 | 0.792 | 0.849 | 0.996 | 0.955 | 0.654 | 1.000 | 1.000 | 0.927 |
| 413 | 0.725 | 1.000 | 1.000 | 0.958 | 0.557 | 1.000 | 1.000 | 0.926 | 0.678 | 0.988 | 1.000 | 0.960 | 0.792 | 0.849 | 0.996 | 0.955 | 0.660 | 1.000 | 1.000 | 0.928 |
| 414 | 0.725 | 1.000 | 1.000 | 0.958 | 0.557 | 1.000 | 1.000 | 0.926 | 0.661 | 0.988 | 1.000 | 0.958 | 0.792 | 0.849 | 0.996 | 0.955 | 0.660 | 1.000 | 1.000 | 0.928 |
| 415 | 0.725 | 1.000 | 1.000 | 0.958 | 0.557 | 1.000 | 1.000 | 0.926 | 0.661 | 0.988 | 1.000 | 0.958 | 0.792 | 0.849 | 0.996 | 0.955 | 0.660 | 1.000 | 1.000 | 0.928 |
| 416 | 0.738 | 1.000 | 1.000 | 0.960 | 0.557 | 1.000 | 1.000 | 0.926 | 0.678 | 0.988 | 1.000 | 0.960 | 0.792 | 0.849 | 0.996 | 0.955 | 0.665 | 1.000 | 1.000 | 0.929 |
| 417 | 0.750 | 1.000 | 1.000 | 0.962 | 0.544 | 1.000 | 1.000 | 0.923 | 0.678 | 0.988 | 1.000 | 0.960 | 0.792 | 0.849 | 0.996 | 0.955 | 0.676 | 1.000 | 1.000 | 0.932 |
| 418 | 0.763 | 1.000 | 1.000 | 0.964 | 0.544 | 1.000 | 1.000 | 0.923 | 0.678 | 0.988 | 1.000 | 0.960 | 0.792 | 0.849 | 0.997 | 0.956 | 0.676 | 1.000 | 1.000 | 0.932 |
| 419 | 0.763 | 1.000 | 1.000 | 0.964 | 0.557 | 1.000 | 1.000 | 0.926 | 0.678 | 0.988 | 1.000 | 0.960 | 0.799 | 0.849 | 0.997 | 0.957 | 0.676 | 1.000 | 1.000 | 0.932 |
| 420 | 0.750 | 1.000 | 1.000 | 0.962 | 0.544 | 1.000 | 1.000 | 0.923 | 0.678 | 0.988 | 1.000 | 0.960 | 0.799 | 0.849 | 0.997 | 0.957 | 0.670 | 1.000 | 1.000 | 0.931 |
| 421 | 0.750 | 1.000 | 1.000 | 0.962 | 0.544 | 1.000 | 1.000 | 0.923 | 0.678 | 0.988 | 1.000 | 0.960 | 0.786 | 0.849 | 0.997 | 0.955 | 0.681 | 0.988 | 1.000 | 0.932 |
| 422 | 0.763 | 1.000 | 1.000 | 0.964 | 0.544 | 1.000 | 1.000 | 0.923 | 0.678 | 0.988 | 1.000 | 0.960 | 0.792 | 0.849 | 0.997 | 0.956 | 0.691 | 0.988 | 1.000 | 0.934 |
| 423 | 0.775 | 1.000 | 1.000 | 0.966 | 0.544 | 1.000 | 1.000 | 0.923 | 0.678 | 0.988 | 1.000 | 0.960 | 0.792 | 0.849 | 0.996 | 0.955 | 0.691 | 1.000 | 1.000 | 0.935 |
| 424 | 0.763 | 1.000 | 1.000 | 0.964 | 0.544 | 1.000 | 1.000 | 0.923 | 0.661 | 0.988 | 1.000 | 0.958 | 0.792 | 0.849 | 0.996 | 0.955 | 0.686 | 1.000 | 1.000 | 0.934 |
| 425 | 0.763 | 1.000 | 1.000 | 0.964 | 0.544 | 1.000 | 1.000 | 0.923 | 0.661 | 0.988 | 1.000 | 0.958 | 0.792 | 0.849 | 0.996 | 0.955 | 0.681 | 1.000 | 1.000 | 0.933 |
| 426 | 0.738 | 1.000 | 1.000 | 0.960 | 0.544 | 1.000 | 1.000 | 0.923 | 0.627 | 0.988 | 1.000 | 0.954 | 0.786 | 0.849 | 0.996 | 0.953 | 0.676 | 1.000 | 1.000 | 0.932 |
| 427 | 0.750 | 1.000 | 1.000 | 0.962 | 0.544 | 1.000 | 1.000 | 0.923 | 0.661 | 0.988 | 1.000 | 0.958 | 0.779 | 0.849 | 0.996 | 0.952 | 0.681 | 1.000 | 1.000 | 0.933 |
| 428 | 0.750 | 1.000 | 1.000 | 0.962 | 0.544 | 1.000 | 1.000 | 0.923 | 0.661 | 0.988 | 1.000 | 0.958 | 0.779 | 0.849 | 0.996 | 0.952 | 0.681 | 1.000 | 1.000 | 0.933 |
| 429 | 0.750 | 1.000 | 1.000 | 0.962 | 0.544 | 1.000 | 1.000 | 0.923 | 0.661 | 0.988 | 1.000 | 0.958 | 0.779 | 0.849 | 0.996 | 0.952 | 0.681 | 1.000 | 1.000 | 0.933 |
| 430 | 0.750 | 1.000 | 1.000 | 0.962 | 0.532 | 1.000 | 1.000 | 0.921 | 0.661 | 0.988 | 1.000 | 0.958 | 0.779 | 0.849 | 0.996 | 0.952 | 0.676 | 1.000 | 1.000 | 0.932 |
| 431 | 0.750 | 1.000 | 1.000 | 0.962 | 0.532 | 1.000 | 1.000 | 0.921 | 0.661 | 0.988 | 1.000 | 0.958 | 0.779 | 0.849 | 0.996 | 0.952 | 0.681 | 1.000 | 1.000 | 0.933 |
| 432 | 0.750 | 1.000 | 1.000 | 0.962 | 0.532 | 1.000 | 1.000 | 0.921 | 0.661 | 0.988 | 1.000 | 0.958 | 0.779 | 0.849 | 0.996 | 0.952 | 0.681 | 1.000 | 1.000 | 0.933 |
| 433 | 0.738 | 1.000 | 1.000 | 0.960 | 0.544 | 1.000 | 1.000 | 0.923 | 0.661 | 0.975 | 1.000 | 0.956 | 0.779 | 0.849 | 0.996 | 0.952 | 0.670 | 1.000 | 1.000 | 0.931 |
| 434 | 0.738 | 1.000 | 1.000 | 0.960 | 0.544 | 1.000 | 1.000 | 0.923 | 0.661 | 0.975 | 1.000 | 0.956 | 0.779 | 0.849 | 0.996 | 0.952 | 0.670 | 1.000 | 1.000 | 0.931 |
| 435 | 0.738 | 1.000 | 1.000 | 0.960 | 0.544 | 1.000 | 1.000 | 0.923 | 0.661 | 0.975 | 1.000 | 0.956 | 0.773 | 0.849 | 0.996 | 0.951 | 0.670 | 1.000 | 1.000 | 0.931 |
| 436 | 0.738 | 1.000 | 1.000 | 0.960 | 0.544 | 1.000 | 1.000 | 0.923 | 0.661 | 0.975 | 1.000 | 0.956 | 0.773 | 0.849 | 0.996 | 0.951 | 0.681 | 1.000 | 1.000 | 0.933 |
| 437 | 0.738 | 1.000 | 1.000 | 0.960 | 0.544 | 1.000 | 1.000 | 0.923 | 0.661 | 0.975 | 1.000 | 0.956 | 0.773 | 0.849 | 0.996 | 0.951 | 0.676 | 1.000 | 1.000 | 0.932 |
| 438 | 0.738 | 1.000 | 1.000 | 0.960 | 0.544 | 1.000 | 1.000 | 0.923 | 0.661 | 0.975 | 1.000 | 0.956 | 0.779 | 0.849 | 0.996 | 0.952 | 0.681 | 1.000 | 1.000 | 0.933 |
| 439 | 0.738 | 1.000 | 1.000 | 0.960 | 0.544 | 1.000 | 1.000 | 0.923 | 0.661 | 0.975 | 1.000 | 0.956 | 0.779 | 0.849 | 0.996 | 0.952 | 0.681 | 1.000 | 1.000 | 0.933 |
| 440 | 0.738 | 1.000 | 1.000 | 0.960 | 0.544 | 1.000 | 1.000 | 0.923 | 0.661 | 0.975 | 1.000 | 0.956 | 0.779 | 0.849 | 0.997 | 0.953 | 0.670 | 1.000 | 1.000 | 0.931 |
| 441 | 0.738 | 1.000 | 1.000 | 0.960 | 0.544 | 1.000 | 1.000 | 0.923 | 0.644 | 0.975 | 1.000 | 0.954 | 0.779 | 0.849 | 0.997 | 0.953 | 0.665 | 1.000 | 1.000 | 0.929 |
| 442 | 0.738 | 1.000 | 1.000 | 0.960 | 0.544 | 1.000 | 1.000 | 0.923 | 0.644 | 0.975 | 1.000 | 0.954 | 0.779 | 0.849 | 0.997 | 0.953 | 0.665 | 1.000 | 1.000 | 0.929 |
| 443 | 0.738 | 1.000 | 1.000 | 0.960 | 0.544 | 1.000 | 1.000 | 0.923 | 0.644 | 0.975 | 1.000 | 0.954 | 0.779 | 0.849 | 0.995 | 0.951 | 0.665 | 1.000 | 1.000 | 0.929 |
| 444 | 0.738 | 1.000 | 1.000 | 0.960 | 0.544 | 1.000 | 1.000 | 0.923 | 0.661 | 0.975 | 1.000 | 0.956 | 0.779 | 0.849 | 0.995 | 0.951 | 0.665 | 1.000 | 1.000 | 0.929 |
| 445 | 0.738 | 1.000 | 1.000 | 0.960 | 0.544 | 1.000 | 1.000 | 0.923 | 0.661 | 0.975 | 1.000 | 0.956 | 0.779 | 0.849 | 0.995 | 0.951 | 0.660 | 1.000 | 1.000 | 0.928 |
| 446 | 0.738 | 1.000 | 1.000 | 0.960 | 0.532 | 1.000 | 1.000 | 0.921 | 0.661 | 0.975 | 1.000 | 0.956 | 0.779 | 0.849 | 0.995 | 0.951 | 0.660 | 1.000 | 1.000 | 0.928 |
| 447 | 0.738 | 1.000 | 1.000 | 0.960 | 0.544 | 1.000 | 1.000 | 0.923 | 0.661 | 0.975 | 1.000 | 0.956 | 0.779 | 0.849 | 0.995 | 0.951 | 0.660 | 1.000 | 1.000 | 0.928 |
| 448 | 0.738 | 1.000 | 1.000 | 0.960 | 0.544 | 1.000 | 1.000 | 0.923 | 0.661 | 0.975 | 1.000 | 0.956 | 0.779 | 0.849 | 0.995 | 0.951 | 0.654 | 0.988 | 1.000 | 0.926 |
| 449 | 0.738 | 1.000 | 1.000 | 0.960 | 0.544 | 1.000 | 1.000 | 0.923 | 0.661 | 0.975 | 1.000 | 0.956 | 0.779 | 0.849 | 0.995 | 0.951 | 0.660 | 1.000 | 1.000 | 0.928 |
| 450 | 0.738 | 1.000 | 1.000 | 0.960 | 0.519 | 1.000 | 1.000 | 0.919 | 0.661 | 0.975 | 1.000 | 0.956 | 0.779 | 0.849 | 0.995 | 0.951 | 0.638 | 1.000 | 1.000 | 0.924 |
| 451 | 0.738 | 1.000 | 1.000 | 0.960 | 0.506 | 1.000 | 1.000 | 0.917 | 0.661 | 0.975 | 1.000 | 0.956 | 0.779 | 0.849 | 0.995 | 0.951 | 0.638 | 1.000 | 1.000 | 0.924 |
| 452 | 0.725 | 1.000 | 1.000 | 0.958 | 0.494 | 1.000 | 1.000 | 0.915 | 0.644 | 0.975 | 1.000 | 0.954 | 0.779 | 0.849 | 0.995 | 0.951 | 0.638 | 1.000 | 1.000 | 0.924 |
| 453 | 0.725 | 1.000 | 1.000 | 0.958 | 0.494 | 1.000 | 1.000 | 0.915 | 0.644 | 0.975 | 1.000 | 0.954 | 0.779 | 0.849 | 0.996 | 0.952 | 0.638 | 1.000 | 1.000 | 0.924 |
| 454 | 0.725 | 1.000 | 1.000 | 0.958 | 0.494 | 1.000 | 1.000 | 0.915 | 0.644 | 0.975 | 1.000 | 0.954 | 0.779 | 0.849 | 0.996 | 0.952 | 0.638 | 1.000 | 1.000 | 0.924 |
| 455 | 0.738 | 1.000 | 1.000 | 0.960 | 0.481 | 1.000 | 1.000 | 0.913 | 0.644 | 0.975 | 1.000 | 0.954 | 0.779 | 0.849 | 0.996 | 0.952 | 0.638 | 1.000 | 1.000 | 0.924 |
| 456 | 0.738 | 1.000 | 1.000 | 0.960 | 0.481 | 1.000 | 1.000 | 0.913 | 0.644 | 0.975 | 1.000 | 0.954 | 0.779 | 0.849 | 0.996 | 0.952 | 0.638 | 1.000 | 1.000 | 0.924 |
| 457 | 0.738 | 1.000 | 1.000 | 0.960 | 0.481 | 1.000 | 1.000 | 0.913 | 0.644 | 0.975 | 1.000 | 0.954 | 0.773 | 0.849 | 0.996 | 0.951 | 0.638 | 1.000 | 1.000 | 0.924 |
| 458 | 0.725 | 1.000 | 1.000 | 0.958 | 0.481 | 1.000 | 1.000 | 0.913 | 0.644 | 0.975 | 1.000 | 0.954 | 0.779 | 0.849 | 0.997 | 0.953 | 0.649 | 1.000 | 1.000 | 0.926 |
| 459 | 0.700 | 1.000 | 1.000 | 0.955 | 0.481 | 1.000 | 1.000 | 0.913 | 0.627 | 0.975 | 1.000 | 0.952 | 0.773 | 0.849 | 0.997 | 0.952 | 0.649 | 1.000 | 1.000 | 0.926 |
| 460 | 0.688 | 1.000 | 1.000 | 0.953 | 0.468 | 1.000 | 1.000 | 0.911 | 0.644 | 0.975 | 1.000 | 0.954 | 0.779 | 0.849 | 0.996 | 0.952 | 0.644 | 1.000 | 1.000 | 0.925 |
| 461 | 0.688 | 1.000 | 1.000 | 0.953 | 0.456 | 1.000 | 1.000 | 0.909 | 0.644 | 0.975 | 1.000 | 0.954 | 0.773 | 0.849 | 0.996 | 0.951 | 0.644 | 1.000 | 1.000 | 0.925 |
| 462 | 0.688 | 1.000 | 1.000 | 0.953 | 0.456 | 1.000 | 1.000 | 0.909 | 0.644 | 0.975 | 1.000 | 0.954 | 0.773 | 0.849 | 0.996 | 0.951 | 0.644 | 1.000 | 1.000 | 0.925 |
| 463 | 0.688 | 1.000 | 1.000 | 0.953 | 0.443 | 1.000 | 1.000 | 0.906 | 0.627 | 0.975 | 1.000 | 0.952 | 0.773 | 0.849 | 0.996 | 0.951 | 0.644 | 1.000 | 1.000 | 0.925 |
| 464 | 0.688 | 1.000 | 1.000 | 0.953 | 0.443 | 1.000 | 1.000 | 0.906 | 0.627 | 0.975 | 1.000 | 0.952 | 0.773 | 0.849 | 0.996 | 0.951 | 0.644 | 0.988 | 1.000 | 0.924 |
| 465 | 0.688 | 1.000 | 1.000 | 0.953 | 0.443 | 1.000 | 1.000 | 0.906 | 0.627 | 0.975 | 1.000 | 0.952 | 0.773 | 0.849 | 0.996 | 0.951 | 0.644 | 0.988 | 1.000 | 0.924 |
| 466 | 0.688 | 1.000 | 1.000 | 0.953 | 0.443 | 1.000 | 1.000 | 0.906 | 0.627 | 0.975 | 1.000 | 0.952 | 0.773 | 0.849 | 0.996 | 0.951 | 0.644 | 0.988 | 1.000 | 0.924 |
| 467 | 0.688 | 1.000 | 1.000 | 0.953 | 0.443 | 1.000 | 1.000 | 0.906 | 0.610 | 0.975 | 1.000 | 0.950 | 0.773 | 0.849 | 0.996 | 0.951 | 0.644 | 0.988 | 1.000 | 0.924 |
| 468 | 0.675 | 1.000 | 1.000 | 0.951 | 0.443 | 1.000 | 1.000 | 0.906 | 0.610 | 0.975 | 1.000 | 0.950 | 0.773 | 0.849 | 0.996 | 0.951 | 0.649 | 0.988 | 1.000 | 0.925 |
| 469 | 0.688 | 1.000 | 1.000 | 0.953 | 0.443 | 1.000 | 1.000 | 0.906 | 0.627 | 0.975 | 1.000 | 0.952 | 0.773 | 0.849 | 0.996 | 0.951 | 0.649 | 0.988 | 1.000 | 0.925 |
| 470 | 0.675 | 1.000 | 1.000 | 0.951 | 0.456 | 1.000 | 1.000 | 0.909 | 0.627 | 0.975 | 1.000 | 0.952 | 0.773 | 0.849 | 0.996 | 0.951 | 0.644 | 0.988 | 1.000 | 0.924 |
| 471 | 0.688 | 1.000 | 1.000 | 0.953 | 0.456 | 1.000 | 1.000 | 0.909 | 0.627 | 0.975 | 1.000 | 0.952 | 0.773 | 0.849 | 0.996 | 0.951 | 0.644 | 0.988 | 1.000 | 0.924 |
| 472 | 0.688 | 1.000 | 1.000 | 0.953 | 0.456 | 1.000 | 1.000 | 0.909 | 0.627 | 0.975 | 1.000 | 0.952 | 0.773 | 0.849 | 0.996 | 0.951 | 0.644 | 0.988 | 1.000 | 0.924 |
| 473 | 0.700 | 1.000 | 1.000 | 0.955 | 0.456 | 1.000 | 1.000 | 0.909 | 0.627 | 0.975 | 1.000 | 0.952 | 0.779 | 0.849 | 0.996 | 0.952 | 0.649 | 0.988 | 1.000 | 0.925 |
| 474 | 0.700 | 1.000 | 1.000 | 0.955 | 0.456 | 1.000 | 1.000 | 0.909 | 0.627 | 0.975 | 1.000 | 0.952 | 0.779 | 0.849 | 0.996 | 0.952 | 0.644 | 0.988 | 1.000 | 0.924 |
| 475 | 0.713 | 1.000 | 1.000 | 0.957 | 0.468 | 1.000 | 1.000 | 0.911 | 0.627 | 0.975 | 1.000 | 0.952 | 0.779 | 0.849 | 0.997 | 0.953 | 0.649 | 0.988 | 1.000 | 0.925 |
| 476 | 0.713 | 1.000 | 1.000 | 0.957 | 0.456 | 1.000 | 1.000 | 0.909 | 0.627 | 0.975 | 1.000 | 0.952 | 0.779 | 0.849 | 0.997 | 0.953 | 0.654 | 0.988 | 1.000 | 0.926 |
| 477 | 0.700 | 1.000 | 1.000 | 0.955 | 0.456 | 1.000 | 1.000 | 0.909 | 0.627 | 0.975 | 1.000 | 0.952 | 0.779 | 0.849 | 0.996 | 0.952 | 0.649 | 0.988 | 1.000 | 0.925 |
| 478 | 0.700 | 1.000 | 1.000 | 0.955 | 0.456 | 1.000 | 1.000 | 0.909 | 0.627 | 0.975 | 1.000 | 0.952 | 0.779 | 0.849 | 0.996 | 0.952 | 0.649 | 0.988 | 1.000 | 0.925 |
| 479 | 0.713 | 1.000 | 1.000 | 0.957 | 0.456 | 1.000 | 1.000 | 0.909 | 0.627 | 0.975 | 1.000 | 0.952 | 0.779 | 0.849 | 0.996 | 0.952 | 0.649 | 0.988 | 1.000 | 0.925 |
| 480 | 0.725 | 1.000 | 1.000 | 0.958 | 0.456 | 1.000 | 1.000 | 0.909 | 0.644 | 0.975 | 1.000 | 0.954 | 0.779 | 0.849 | 0.996 | 0.952 | 0.628 | 0.988 | 1.000 | 0.920 |
| 481 | 0.725 | 1.000 | 1.000 | 0.958 | 0.456 | 1.000 | 1.000 | 0.909 | 0.644 | 0.975 | 1.000 | 0.954 | 0.779 | 0.849 | 0.996 | 0.952 | 0.628 | 0.988 | 1.000 | 0.920 |
| 482 | 0.725 | 1.000 | 1.000 | 0.958 | 0.456 | 1.000 | 1.000 | 0.909 | 0.644 | 0.975 | 1.000 | 0.954 | 0.779 | 0.849 | 0.997 | 0.953 | 0.628 | 0.988 | 1.000 | 0.920 |
| 483 | 0.725 | 1.000 | 1.000 | 0.958 | 0.456 | 1.000 | 1.000 | 0.909 | 0.644 | 0.975 | 1.000 | 0.954 | 0.779 | 0.849 | 0.997 | 0.953 | 0.628 | 0.988 | 1.000 | 0.920 |
| 484 | 0.725 | 1.000 | 1.000 | 0.958 | 0.456 | 1.000 | 1.000 | 0.909 | 0.644 | 0.975 | 1.000 | 0.954 | 0.779 | 0.849 | 0.997 | 0.953 | 0.628 | 0.988 | 1.000 | 0.920 |
| 485 | 0.738 | 1.000 | 1.000 | 0.960 | 0.481 | 1.000 | 1.000 | 0.913 | 0.661 | 0.975 | 1.000 | 0.956 | 0.786 | 0.849 | 0.996 | 0.953 | 0.638 | 0.988 | 1.000 | 0.923 |
| 486 | 0.738 | 1.000 | 1.000 | 0.960 | 0.481 | 1.000 | 1.000 | 0.913 | 0.661 | 0.975 | 1.000 | 0.956 | 0.786 | 0.849 | 0.996 | 0.953 | 0.638 | 0.988 | 1.000 | 0.923 |
| 487 | 0.738 | 1.000 | 1.000 | 0.960 | 0.481 | 1.000 | 1.000 | 0.913 | 0.661 | 0.975 | 1.000 | 0.956 | 0.786 | 0.849 | 0.996 | 0.953 | 0.638 | 0.988 | 1.000 | 0.923 |
| 488 | 0.738 | 1.000 | 1.000 | 0.960 | 0.481 | 1.000 | 1.000 | 0.913 | 0.661 | 0.975 | 1.000 | 0.956 | 0.773 | 0.849 | 0.997 | 0.952 | 0.628 | 0.988 | 1.000 | 0.920 |
| 489 | 0.738 | 1.000 | 1.000 | 0.960 | 0.481 | 1.000 | 1.000 | 0.913 | 0.661 | 0.975 | 1.000 | 0.956 | 0.766 | 0.849 | 0.996 | 0.950 | 0.622 | 0.988 | 1.000 | 0.919 |
| 490 | 0.738 | 1.000 | 1.000 | 0.960 | 0.468 | 1.000 | 1.000 | 0.911 | 0.661 | 0.988 | 1.000 | 0.958 | 0.766 | 0.849 | 0.996 | 0.950 | 0.628 | 0.988 | 1.000 | 0.920 |
| 491 | 0.738 | 1.000 | 1.000 | 0.960 | 0.494 | 1.000 | 1.000 | 0.915 | 0.661 | 0.988 | 1.000 | 0.958 | 0.786 | 0.849 | 0.996 | 0.953 | 0.644 | 0.988 | 1.000 | 0.924 |
| 492 | 0.738 | 1.000 | 1.000 | 0.960 | 0.494 | 1.000 | 1.000 | 0.915 | 0.661 | 0.988 | 1.000 | 0.958 | 0.779 | 0.849 | 0.996 | 0.952 | 0.638 | 0.988 | 1.000 | 0.923 |
| 493 | 0.738 | 1.000 | 1.000 | 0.960 | 0.494 | 1.000 | 1.000 | 0.915 | 0.661 | 0.988 | 1.000 | 0.958 | 0.786 | 0.849 | 0.996 | 0.953 | 0.638 | 0.988 | 1.000 | 0.923 |
| 494 | 0.763 | 1.000 | 1.000 | 0.964 | 0.494 | 1.000 | 1.000 | 0.915 | 0.661 | 0.988 | 1.000 | 0.958 | 0.766 | 0.849 | 0.996 | 0.950 | 0.617 | 0.988 | 1.000 | 0.918 |
| 495 | 0.763 | 1.000 | 1.000 | 0.964 | 0.494 | 1.000 | 1.000 | 0.915 | 0.661 | 0.988 | 1.000 | 0.958 | 0.766 | 0.849 | 0.996 | 0.950 | 0.633 | 0.988 | 1.000 | 0.922 |
| 496 | 0.763 | 1.000 | 1.000 | 0.964 | 0.494 | 1.000 | 1.000 | 0.915 | 0.661 | 0.988 | 1.000 | 0.958 | 0.766 | 0.849 | 0.996 | 0.950 | 0.633 | 0.988 | 1.000 | 0.922 |
| 497 | 0.763 | 1.000 | 1.000 | 0.964 | 0.494 | 1.000 | 1.000 | 0.915 | 0.661 | 0.988 | 1.000 | 0.958 | 0.766 | 0.849 | 0.996 | 0.950 | 0.633 | 0.988 | 1.000 | 0.922 |
| 498 | 0.763 | 1.000 | 1.000 | 0.964 | 0.494 | 1.000 | 1.000 | 0.915 | 0.661 | 0.988 | 1.000 | 0.958 | 0.766 | 0.849 | 0.996 | 0.950 | 0.633 | 0.988 | 1.000 | 0.922 |
| 499 | 0.763 | 1.000 | 1.000 | 0.964 | 0.494 | 1.000 | 1.000 | 0.915 | 0.661 | 0.975 | 1.000 | 0.956 | 0.766 | 0.849 | 0.996 | 0.950 | 0.628 | 0.988 | 1.000 | 0.920 |
| 500 | 0.750 | 1.000 | 1.000 | 0.962 | 0.481 | 1.000 | 1.000 | 0.913 | 0.661 | 0.975 | 1.000 | 0.956 | 0.766 | 0.849 | 0.996 | 0.950 | 0.628 | 0.988 | 1.000 | 0.920 |

1. The dataset of H0351.1012 was selected as the training dataset

| Number of features | H0351.1009 | | | | H0351.1015 | | | | H0351.1016 | | | | H0351.2001 | | | | H0351.2002 | | | |
| --- | --- | --- | --- | --- | --- | --- | --- | --- | --- | --- | --- | --- | --- | --- | --- | --- | --- | --- | --- | --- |
|  | class1 | class2 | class3 | total | class1 | class2 | class3 | total | class1 | class2 | class3 | total | class1 | class2 | class3 | total | class1 | class2 | class3 | total |
| 4 | 0.96154 | 1 | 1 | 0.99725 | 0.88608 | 1 | 1 | 0.98085 | 0.91525 | 1 | 1 | 0.99002 | 0.90909 | 1 | 1 | 0.9852 | 0.88298 | 1 | 1 | 0.975364 |
| 5 | 0.96154 | 1 | 1 | 0.99725 | 0.88608 | 1 | 1 | 0.98085 | 0.9322 | 1 | 1 | 0.99202 | 0.9026 | 1 | 1 | 0.98414 | 0.8883 | 1 | 1 | 0.976484 |
| 6 | 0.96154 | 1 | 1 | 0.99725 | 0.88608 | 1 | 1 | 0.98085 | 0.9322 | 1 | 1 | 0.99202 | 0.9026 | 1 | 1 | 0.98414 | 0.89362 | 1 | 1 | 0.977604 |
| 7 | 0.96154 | 1 | 1 | 0.99725 | 0.89873 | 1 | 1 | 0.98298 | 0.94915 | 1 | 1 | 0.99401 | 0.90909 | 1 | 1 | 0.9852 | 0.90426 | 1 | 1 | 0.979843 |
| 8 | 1 | 1 | 1 | 1 | 0.89873 | 1 | 1 | 0.98298 | 0.9322 | 1 | 1 | 0.99202 | 0.9026 | 1 | 1 | 0.98414 | 0.89894 | 1 | 1 | 0.978723 |
| 9 | 1 | 1 | 1 | 1 | 0.92405 | 1 | 1 | 0.98723 | 0.9661 | 1 | 1 | 0.99601 | 0.92857 | 1 | 0.99865 | 0.98732 | 0.87766 | 1 | 1 | 0.974244 |
| 10 | 1 | 1 | 1 | 1 | 0.92405 | 1 | 1 | 0.98723 | 0.98305 | 1 | 1 | 0.998 | 0.95455 | 1 | 0.99865 | 0.99154 | 0.89362 | 1 | 1 | 0.977604 |
| 11 | 1 | 1 | 1 | 1 | 0.89873 | 1 | 1 | 0.98298 | 0.9661 | 1 | 1 | 0.99601 | 0.94805 | 1 | 1 | 0.99154 | 0.87234 | 1 | 1 | 0.973124 |
| 12 | 1 | 1 | 1 | 1 | 0.91139 | 1 | 1 | 0.98511 | 0.98305 | 1 | 1 | 0.998 | 0.94805 | 1 | 0.99865 | 0.99049 | 0.88298 | 1 | 1 | 0.975364 |
| 13 | 1 | 1 | 1 | 1 | 0.89873 | 1 | 1 | 0.98298 | 0.98305 | 1 | 1 | 0.998 | 0.94156 | 1 | 0.99865 | 0.98943 | 0.8883 | 1 | 1 | 0.976484 |
| 14 | 1 | 1 | 1 | 1 | 0.89873 | 1 | 1 | 0.98298 | 0.98305 | 1 | 1 | 0.998 | 0.95455 | 1 | 0.99865 | 0.99154 | 0.8883 | 1 | 1 | 0.976484 |
| 15 | 1 | 1 | 1 | 1 | 0.91139 | 1 | 1 | 0.98511 | 0.98305 | 1 | 1 | 0.998 | 0.95455 | 1 | 1 | 0.9926 | 0.8883 | 1 | 1 | 0.976484 |
| 16 | 1 | 1 | 1 | 1 | 0.91139 | 1 | 1 | 0.98511 | 0.98305 | 1 | 1 | 0.998 | 0.95455 | 1 | 1 | 0.9926 | 0.88298 | 1 | 1 | 0.975364 |
| 17 | 1 | 1 | 1 | 1 | 0.91139 | 1 | 1 | 0.98511 | 0.98305 | 1 | 1 | 0.998 | 0.96104 | 1 | 1 | 0.99366 | 0.8883 | 1 | 1 | 0.976484 |
| 18 | 0.96154 | 1 | 1 | 0.99725 | 0.91139 | 1 | 1 | 0.98511 | 0.98305 | 1 | 1 | 0.998 | 0.96104 | 1 | 0.99865 | 0.9926 | 0.90426 | 1 | 1 | 0.979843 |
| 19 | 1 | 1 | 1 | 1 | 0.91139 | 1 | 1 | 0.98511 | 0.98305 | 1 | 1 | 0.998 | 0.95455 | 1 | 0.99865 | 0.99154 | 0.90426 | 1 | 1 | 0.979843 |
| 20 | 1 | 1 | 1 | 1 | 0.92405 | 1 | 1 | 0.98723 | 0.98305 | 1 | 1 | 0.998 | 0.95455 | 1 | 0.99865 | 0.99154 | 0.90957 | 1 | 0.99839 | 0.979843 |
| 21 | 1 | 1 | 1 | 1 | 0.92405 | 1 | 1 | 0.98723 | 0.98305 | 1 | 1 | 0.998 | 0.93507 | 1 | 0.99865 | 0.98837 | 0.90957 | 1 | 0.99839 | 0.979843 |
| 22 | 0.96154 | 1 | 1 | 0.99725 | 0.92405 | 1 | 1 | 0.98723 | 0.98305 | 1 | 1 | 0.998 | 0.93507 | 1 | 1 | 0.98943 | 0.89894 | 1 | 0.99839 | 0.977604 |
| 23 | 0.92308 | 1 | 1 | 0.99449 | 0.93671 | 1 | 1 | 0.98936 | 0.98305 | 1 | 1 | 0.998 | 0.93507 | 1 | 0.99729 | 0.98732 | 0.90957 | 1 | 0.99839 | 0.979843 |
| 24 | 0.96154 | 1 | 1 | 0.99725 | 0.93671 | 1 | 1 | 0.98936 | 0.98305 | 1 | 1 | 0.998 | 0.94156 | 1 | 0.99729 | 0.98837 | 0.90957 | 1 | 0.99839 | 0.979843 |
| 25 | 0.92308 | 1 | 1 | 0.99449 | 0.93671 | 1 | 1 | 0.98936 | 0.98305 | 1 | 1 | 0.998 | 0.94156 | 1 | 0.99729 | 0.98837 | 0.90957 | 1 | 0.99839 | 0.979843 |
| 26 | 0.92308 | 1 | 1 | 0.99449 | 0.93671 | 1 | 1 | 0.98936 | 0.98305 | 1 | 1 | 0.998 | 0.94156 | 1 | 0.99729 | 0.98837 | 0.90957 | 1 | 0.99839 | 0.979843 |
| 27 | 0.92308 | 1 | 1 | 0.99449 | 0.93671 | 1 | 1 | 0.98936 | 0.98305 | 1 | 1 | 0.998 | 0.94805 | 1 | 0.99729 | 0.98943 | 0.90957 | 1 | 0.99839 | 0.979843 |
| 28 | 0.96154 | 1 | 1 | 0.99725 | 0.93671 | 1 | 1 | 0.98936 | 0.9661 | 1 | 1 | 0.99601 | 0.93507 | 1 | 0.99729 | 0.98732 | 0.90957 | 1 | 0.99839 | 0.979843 |
| 29 | 0.96154 | 1 | 1 | 0.99725 | 0.93671 | 1 | 1 | 0.98936 | 0.98305 | 1 | 1 | 0.998 | 0.94805 | 1 | 0.99729 | 0.98943 | 0.90957 | 1 | 0.99839 | 0.979843 |
| 30 | 1 | 1 | 1 | 1 | 0.94937 | 1 | 1 | 0.99149 | 0.98305 | 1 | 1 | 0.998 | 0.96104 | 1 | 0.99729 | 0.99154 | 0.90957 | 1 | 0.99839 | 0.979843 |
| 31 | 1 | 1 | 1 | 1 | 0.94937 | 1 | 1 | 0.99149 | 0.98305 | 1 | 1 | 0.998 | 0.95455 | 1 | 0.99729 | 0.99049 | 0.92553 | 1 | 0.99839 | 0.983203 |
| 32 | 1 | 1 | 1 | 1 | 0.96203 | 1 | 1 | 0.99362 | 0.98305 | 1 | 1 | 0.998 | 0.96104 | 1 | 0.99729 | 0.99154 | 0.93617 | 1 | 1 | 0.986562 |
| 33 | 1 | 1 | 1 | 1 | 0.96203 | 1 | 1 | 0.99362 | 0.98305 | 1 | 1 | 0.998 | 0.96104 | 1 | 0.99729 | 0.99154 | 0.93085 | 1 | 1 | 0.985442 |
| 34 | 1 | 1 | 0.99661 | 0.99725 | 0.96203 | 1 | 1 | 0.99362 | 0.98305 | 1 | 1 | 0.998 | 0.96104 | 1 | 0.99729 | 0.99154 | 0.93085 | 1 | 0.99839 | 0.984323 |
| 35 | 1 | 1 | 0.99661 | 0.99725 | 0.96203 | 1 | 1 | 0.99362 | 0.98305 | 1 | 1 | 0.998 | 0.96753 | 1 | 0.99729 | 0.9926 | 0.93085 | 1 | 0.99839 | 0.984323 |
| 36 | 1 | 1 | 0.99661 | 0.99725 | 0.96203 | 1 | 1 | 0.99362 | 0.98305 | 1 | 1 | 0.998 | 0.96753 | 1 | 0.99729 | 0.9926 | 0.93085 | 1 | 0.99839 | 0.984323 |
| 37 | 1 | 1 | 1 | 1 | 0.96203 | 1 | 1 | 0.99362 | 0.9661 | 1 | 1 | 0.99601 | 0.94805 | 1 | 0.99729 | 0.98943 | 0.93085 | 1 | 1 | 0.985442 |
| 38 | 1 | 1 | 1 | 1 | 0.94937 | 1 | 1 | 0.99149 | 0.9661 | 1 | 1 | 0.99601 | 0.94805 | 1 | 0.99729 | 0.98943 | 0.93085 | 1 | 1 | 0.985442 |
| 39 | 1 | 1 | 1 | 1 | 0.96203 | 1 | 1 | 0.99362 | 0.9661 | 1 | 1 | 0.99601 | 0.94805 | 1 | 0.99729 | 0.98943 | 0.93085 | 1 | 1 | 0.985442 |
| 40 | 1 | 1 | 1 | 1 | 0.96203 | 1 | 1 | 0.99362 | 0.9661 | 1 | 1 | 0.99601 | 0.94805 | 1 | 0.99729 | 0.98943 | 0.93085 | 1 | 1 | 0.985442 |
| 41 | 1 | 1 | 1 | 1 | 0.96203 | 1 | 1 | 0.99362 | 0.9661 | 1 | 1 | 0.99601 | 0.95455 | 1 | 0.99729 | 0.99049 | 0.93085 | 1 | 1 | 0.985442 |
| 42 | 1 | 1 | 1 | 1 | 0.94937 | 1 | 1 | 0.99149 | 0.9661 | 1 | 1 | 0.99601 | 0.94805 | 1 | 0.99865 | 0.99049 | 0.91489 | 1 | 1 | 0.982083 |
| 43 | 1 | 1 | 1 | 1 | 0.94937 | 1 | 1 | 0.99149 | 0.9661 | 1 | 1 | 0.99601 | 0.94805 | 1 | 0.99865 | 0.99049 | 0.91489 | 1 | 1 | 0.982083 |
| 44 | 1 | 1 | 1 | 1 | 0.94937 | 1 | 1 | 0.99149 | 0.9661 | 1 | 1 | 0.99601 | 0.94805 | 1 | 0.99865 | 0.99049 | 0.91489 | 1 | 1 | 0.982083 |
| 45 | 1 | 1 | 1 | 1 | 0.94937 | 1 | 1 | 0.99149 | 0.9661 | 1 | 1 | 0.99601 | 0.94805 | 1 | 0.99865 | 0.99049 | 0.91489 | 1 | 1 | 0.982083 |
| 46 | 1 | 1 | 1 | 1 | 0.94937 | 1 | 1 | 0.99149 | 0.9661 | 1 | 1 | 0.99601 | 0.94805 | 1 | 0.99865 | 0.99049 | 0.91489 | 1 | 1 | 0.982083 |
| 47 | 1 | 1 | 1 | 1 | 0.94937 | 1 | 1 | 0.99149 | 0.9661 | 1 | 1 | 0.99601 | 0.94805 | 1 | 0.99865 | 0.99049 | 0.90957 | 1 | 1 | 0.980963 |
| 48 | 1 | 1 | 1 | 1 | 0.94937 | 1 | 1 | 0.99149 | 0.9661 | 1 | 1 | 0.99601 | 0.94805 | 1 | 0.99865 | 0.99049 | 0.91489 | 1 | 1 | 0.982083 |
| 49 | 1 | 1 | 1 | 1 | 0.94937 | 1 | 1 | 0.99149 | 0.9661 | 1 | 1 | 0.99601 | 0.94805 | 1 | 0.99865 | 0.99049 | 0.90957 | 1 | 1 | 0.980963 |
| 50 | 1 | 1 | 1 | 1 | 0.94937 | 1 | 1 | 0.99149 | 0.9661 | 1 | 1 | 0.99601 | 0.94805 | 1 | 0.99865 | 0.99049 | 0.90957 | 1 | 1 | 0.980963 |
| 51 | 1 | 1 | 1 | 1 | 0.94937 | 1 | 1 | 0.99149 | 0.9661 | 1 | 1 | 0.99601 | 0.94805 | 1 | 0.99865 | 0.99049 | 0.90957 | 1 | 1 | 0.980963 |
| 52 | 1 | 1 | 1 | 1 | 0.94937 | 1 | 1 | 0.99149 | 0.9661 | 1 | 1 | 0.99601 | 0.94805 | 1 | 1 | 0.99154 | 0.90957 | 1 | 1 | 0.980963 |
| 53 | 1 | 1 | 0.99661 | 0.99725 | 0.94937 | 1 | 1 | 0.99149 | 0.9661 | 1 | 0.99171 | 0.99002 | 0.96104 | 1 | 0.99729 | 0.99154 | 0.94681 | 1 | 0.99518 | 0.985442 |
| 54 | 1 | 1 | 0.99661 | 0.99725 | 0.94937 | 1 | 1 | 0.99149 | 0.98305 | 1 | 0.99448 | 0.99401 | 0.98052 | 1 | 0.99729 | 0.99472 | 0.95745 | 1 | 0.99357 | 0.986562 |
| 55 | 1 | 1 | 0.99661 | 0.99725 | 0.94937 | 1 | 1 | 0.99149 | 0.98305 | 1 | 0.99448 | 0.99401 | 0.96753 | 1 | 0.99729 | 0.9926 | 0.94149 | 1 | 0.99518 | 0.984323 |
| 56 | 1 | 1 | 0.99661 | 0.99725 | 0.94937 | 1 | 1 | 0.99149 | 0.98305 | 1 | 0.99448 | 0.99401 | 0.98701 | 1 | 0.99729 | 0.99577 | 0.94149 | 1 | 0.99357 | 0.983203 |
| 57 | 1 | 1 | 0.99661 | 0.99725 | 0.94937 | 1 | 1 | 0.99149 | 0.98305 | 1 | 0.99171 | 0.99202 | 0.97403 | 1 | 0.99729 | 0.99366 | 0.94681 | 1 | 0.99357 | 0.984323 |
| 58 | 1 | 1 | 0.99661 | 0.99725 | 0.94937 | 1 | 1 | 0.99149 | 0.98305 | 1 | 0.99171 | 0.99202 | 0.98052 | 1 | 0.99729 | 0.99472 | 0.94681 | 1 | 0.99357 | 0.984323 |
| 59 | 1 | 1 | 1 | 1 | 0.96203 | 1 | 1 | 0.99362 | 1 | 1 | 0.99724 | 0.998 | 0.96104 | 1 | 0.99729 | 0.99154 | 0.94681 | 1 | 0.99357 | 0.984323 |
| 60 | 1 | 1 | 1 | 1 | 0.96203 | 1 | 1 | 0.99362 | 1 | 1 | 0.99724 | 0.998 | 0.94805 | 1 | 0.99865 | 0.99049 | 0.94149 | 1 | 0.99357 | 0.983203 |
| 61 | 1 | 1 | 1 | 1 | 0.96203 | 1 | 1 | 0.99362 | 0.98305 | 1 | 0.99724 | 0.99601 | 0.96104 | 1 | 0.99729 | 0.99154 | 0.94681 | 1 | 0.99357 | 0.984323 |
| 62 | 1 | 1 | 1 | 1 | 0.96203 | 1 | 1 | 0.99362 | 0.98305 | 1 | 0.99724 | 0.99601 | 0.96104 | 1 | 0.99729 | 0.99154 | 0.95213 | 1 | 0.99357 | 0.985442 |
| 63 | 1 | 1 | 1 | 1 | 0.96203 | 1 | 1 | 0.99362 | 0.98305 | 1 | 0.99724 | 0.99601 | 0.96104 | 1 | 0.99729 | 0.99154 | 0.95213 | 1 | 0.99357 | 0.985442 |
| 64 | 1 | 1 | 1 | 1 | 0.94937 | 1 | 1 | 0.99149 | 0.98305 | 1 | 0.99724 | 0.99601 | 0.96104 | 1 | 0.99729 | 0.99154 | 0.94681 | 1 | 0.99357 | 0.984323 |
| 65 | 1 | 1 | 1 | 1 | 0.94937 | 1 | 1 | 0.99149 | 0.9661 | 1 | 0.99724 | 0.99401 | 0.93507 | 1 | 0.99865 | 0.98837 | 0.94681 | 1 | 0.99357 | 0.984323 |
| 66 | 1 | 1 | 1 | 1 | 0.94937 | 1 | 1 | 0.99149 | 0.9661 | 1 | 0.99724 | 0.99401 | 0.93507 | 1 | 0.99865 | 0.98837 | 0.94681 | 1 | 0.99357 | 0.984323 |
| 67 | 1 | 1 | 1 | 1 | 0.94937 | 1 | 1 | 0.99149 | 0.9661 | 1 | 0.99724 | 0.99401 | 0.93507 | 1 | 0.99865 | 0.98837 | 0.94681 | 1 | 0.99357 | 0.984323 |
| 68 | 1 | 1 | 1 | 1 | 0.94937 | 1 | 1 | 0.99149 | 0.9661 | 1 | 0.99724 | 0.99401 | 0.93507 | 1 | 0.99865 | 0.98837 | 0.94681 | 1 | 0.99357 | 0.984323 |
| 69 | 1 | 1 | 1 | 1 | 0.94937 | 1 | 1 | 0.99149 | 0.9661 | 1 | 0.99724 | 0.99401 | 0.93507 | 1 | 0.99865 | 0.98837 | 0.94681 | 1 | 0.99357 | 0.984323 |
| 70 | 1 | 1 | 1 | 1 | 0.94937 | 1 | 1 | 0.99149 | 0.9661 | 1 | 0.99724 | 0.99401 | 0.93507 | 1 | 0.99865 | 0.98837 | 0.94681 | 1 | 0.99357 | 0.984323 |
| 71 | 0.96154 | 1 | 1 | 0.99725 | 0.92405 | 1 | 1 | 0.98723 | 0.9661 | 1 | 0.99724 | 0.99401 | 0.96104 | 1 | 0.99729 | 0.99154 | 0.96277 | 1 | 0.99357 | 0.987682 |
| 72 | 0.96154 | 1 | 1 | 0.99725 | 0.92405 | 1 | 1 | 0.98723 | 0.9661 | 1 | 0.99724 | 0.99401 | 0.96104 | 1 | 0.99729 | 0.99154 | 0.95745 | 1 | 0.99357 | 0.986562 |
| 73 | 0.96154 | 1 | 1 | 0.99725 | 0.92405 | 1 | 1 | 0.98723 | 0.9661 | 1 | 0.99724 | 0.99401 | 0.96753 | 1 | 0.99729 | 0.9926 | 0.95745 | 0.9759 | 0.99357 | 0.984323 |
| 74 | 0.96154 | 1 | 1 | 0.99725 | 0.92405 | 1 | 1 | 0.98723 | 0.9661 | 1 | 0.99724 | 0.99401 | 0.96104 | 1 | 0.99865 | 0.9926 | 0.95745 | 0.9759 | 0.99357 | 0.984323 |
| 75 | 0.96154 | 1 | 1 | 0.99725 | 0.92405 | 1 | 1 | 0.98723 | 0.9661 | 1 | 0.99724 | 0.99401 | 0.96104 | 1 | 0.99865 | 0.9926 | 0.95745 | 0.9759 | 0.99357 | 0.984323 |
| 76 | 0.96154 | 1 | 1 | 0.99725 | 0.92405 | 1 | 1 | 0.98723 | 0.9661 | 1 | 0.99724 | 0.99401 | 0.96753 | 1 | 0.99865 | 0.99366 | 0.96277 | 0.9759 | 0.99357 | 0.985442 |
| 77 | 0.96154 | 1 | 1 | 0.99725 | 0.92405 | 1 | 1 | 0.98723 | 0.9661 | 1 | 0.99724 | 0.99401 | 0.96104 | 1 | 0.99729 | 0.99154 | 0.96277 | 0.9759 | 0.99357 | 0.985442 |
| 78 | 1 | 1 | 1 | 1 | 0.92405 | 1 | 1 | 0.98723 | 0.9661 | 1 | 1 | 0.99601 | 0.98052 | 0.98113 | 0.99594 | 0.9926 | 0.96277 | 0.9759 | 0.99357 | 0.985442 |
| 79 | 1 | 1 | 1 | 1 | 0.93671 | 1 | 1 | 0.98936 | 0.9661 | 1 | 1 | 0.99601 | 0.98052 | 0.98113 | 0.99594 | 0.9926 | 0.96277 | 0.9759 | 0.99196 | 0.984323 |
| 80 | 1 | 1 | 1 | 1 | 0.93671 | 1 | 1 | 0.98936 | 0.9661 | 1 | 1 | 0.99601 | 0.98052 | 0.98113 | 0.99594 | 0.9926 | 0.95745 | 0.9759 | 0.99196 | 0.983203 |
| 81 | 1 | 1 | 1 | 1 | 0.93671 | 1 | 1 | 0.98936 | 0.9661 | 1 | 1 | 0.99601 | 0.98052 | 0.98113 | 0.99594 | 0.9926 | 0.96277 | 0.9759 | 0.99196 | 0.984323 |
| 82 | 0.96154 | 1 | 1 | 0.99725 | 0.94937 | 1 | 1 | 0.99149 | 0.9661 | 1 | 1 | 0.99601 | 0.98052 | 0.98113 | 0.99594 | 0.9926 | 0.96277 | 0.9759 | 0.99196 | 0.984323 |
| 83 | 0.96154 | 1 | 1 | 0.99725 | 0.92405 | 1 | 1 | 0.98723 | 0.9661 | 1 | 1 | 0.99601 | 0.98052 | 0.98113 | 0.99594 | 0.9926 | 0.96277 | 0.9759 | 0.99196 | 0.984323 |
| 84 | 0.96154 | 1 | 1 | 0.99725 | 0.93671 | 1 | 1 | 0.98936 | 0.9661 | 1 | 1 | 0.99601 | 0.98701 | 0.98113 | 0.99594 | 0.99366 | 0.96277 | 0.9759 | 0.99196 | 0.984323 |
| 85 | 0.96154 | 1 | 1 | 0.99725 | 0.93671 | 1 | 1 | 0.98936 | 0.9661 | 1 | 1 | 0.99601 | 0.98701 | 0.98113 | 0.99594 | 0.99366 | 0.96277 | 0.9759 | 0.99196 | 0.984323 |
| 86 | 0.96154 | 1 | 1 | 0.99725 | 0.93671 | 1 | 1 | 0.98936 | 0.9661 | 1 | 1 | 0.99601 | 0.98701 | 0.98113 | 0.99594 | 0.99366 | 0.96277 | 0.9759 | 0.99196 | 0.984323 |
| 87 | 0.96154 | 1 | 1 | 0.99725 | 0.93671 | 1 | 1 | 0.98936 | 0.9661 | 1 | 1 | 0.99601 | 0.98701 | 0.98113 | 0.99594 | 0.99366 | 0.96277 | 0.9759 | 0.99196 | 0.984323 |
| 88 | 0.96154 | 1 | 1 | 0.99725 | 0.94937 | 1 | 1 | 0.99149 | 0.9661 | 0.9875 | 1 | 0.99401 | 0.98701 | 0.9434 | 0.99594 | 0.99154 | 0.96277 | 0.95181 | 0.99196 | 0.982083 |
| 89 | 0.96154 | 1 | 1 | 0.99725 | 0.93671 | 1 | 1 | 0.98936 | 0.9661 | 0.9875 | 1 | 0.99401 | 0.97403 | 0.9434 | 0.99594 | 0.98943 | 0.95745 | 0.95181 | 0.99357 | 0.982083 |
| 90 | 0.96154 | 1 | 1 | 0.99725 | 0.93671 | 1 | 1 | 0.98936 | 0.9661 | 0.9875 | 1 | 0.99401 | 0.97403 | 0.9434 | 0.99594 | 0.98943 | 0.95745 | 0.95181 | 0.99357 | 0.982083 |
| 91 | 0.96154 | 1 | 1 | 0.99725 | 0.93671 | 1 | 1 | 0.98936 | 0.9661 | 0.9875 | 0.99724 | 0.99202 | 0.97403 | 0.9434 | 0.99594 | 0.98943 | 0.95745 | 0.95181 | 0.99357 | 0.982083 |
| 92 | 0.96154 | 1 | 1 | 0.99725 | 0.94937 | 1 | 1 | 0.99149 | 0.9661 | 0.975 | 1 | 0.99202 | 0.97403 | 0.9434 | 0.99594 | 0.98943 | 0.95213 | 0.95181 | 0.99357 | 0.980963 |
| 93 | 0.96154 | 1 | 1 | 0.99725 | 0.94937 | 1 | 1 | 0.99149 | 0.9661 | 0.975 | 1 | 0.99202 | 0.97403 | 0.9434 | 0.99594 | 0.98943 | 0.95213 | 0.95181 | 0.99357 | 0.980963 |
| 94 | 0.96154 | 1 | 1 | 0.99725 | 0.94937 | 1 | 1 | 0.99149 | 0.9661 | 0.975 | 1 | 0.99202 | 0.97403 | 0.9434 | 0.99594 | 0.98943 | 0.95213 | 0.95181 | 0.99357 | 0.980963 |
| 95 | 0.96154 | 1 | 1 | 0.99725 | 0.94937 | 1 | 1 | 0.99149 | 0.94915 | 0.975 | 1 | 0.99002 | 0.97403 | 0.9434 | 0.99729 | 0.99049 | 0.94681 | 0.95181 | 0.99357 | 0.979843 |
| 96 | 0.96154 | 1 | 1 | 0.99725 | 0.94937 | 1 | 1 | 0.99149 | 0.94915 | 0.975 | 1 | 0.99002 | 0.97403 | 0.9434 | 0.99729 | 0.99049 | 0.94681 | 0.95181 | 0.99357 | 0.979843 |
| 97 | 0.96154 | 1 | 1 | 0.99725 | 0.94937 | 1 | 1 | 0.99149 | 0.94915 | 0.9875 | 1 | 0.99202 | 0.97403 | 0.9434 | 0.99729 | 0.99049 | 0.95213 | 0.95181 | 0.99357 | 0.980963 |
| 98 | 0.96154 | 1 | 1 | 0.99725 | 0.93671 | 1 | 1 | 0.98936 | 0.94915 | 1 | 1 | 0.99401 | 0.97403 | 0.9434 | 0.99729 | 0.99049 | 0.94149 | 0.95181 | 0.99357 | 0.978723 |
| 99 | 0.96154 | 1 | 1 | 0.99725 | 0.92405 | 1 | 1 | 0.98723 | 0.94915 | 1 | 1 | 0.99401 | 0.97403 | 0.9434 | 0.99594 | 0.98943 | 0.94681 | 0.95181 | 0.99357 | 0.979843 |
| 100 | 0.96154 | 1 | 1 | 0.99725 | 0.92405 | 1 | 1 | 0.98723 | 0.9661 | 1 | 1 | 0.99601 | 0.97403 | 0.9434 | 0.99594 | 0.98943 | 0.95213 | 0.95181 | 0.99357 | 0.980963 |
| 101 | 0.96154 | 1 | 1 | 0.99725 | 0.92405 | 1 | 1 | 0.98723 | 0.9661 | 1 | 1 | 0.99601 | 0.97403 | 0.9434 | 0.99729 | 0.99049 | 0.95213 | 0.95181 | 0.99357 | 0.980963 |
| 102 | 0.96154 | 1 | 1 | 0.99725 | 0.92405 | 1 | 1 | 0.98723 | 0.9661 | 0.9875 | 1 | 0.99401 | 0.97403 | 0.9434 | 0.99729 | 0.99049 | 0.95213 | 0.95181 | 0.99357 | 0.980963 |
| 103 | 0.96154 | 1 | 1 | 0.99725 | 0.92405 | 1 | 1 | 0.98723 | 0.9661 | 0.975 | 1 | 0.99202 | 0.97403 | 0.9434 | 0.99865 | 0.99154 | 0.95213 | 0.95181 | 0.99357 | 0.980963 |
| 104 | 1 | 1 | 1 | 1 | 0.93671 | 1 | 1 | 0.98936 | 0.9661 | 0.9875 | 1 | 0.99401 | 0.98052 | 0.9434 | 0.99729 | 0.99154 | 0.94681 | 0.95181 | 0.99357 | 0.979843 |
| 105 | 1 | 1 | 1 | 1 | 0.93671 | 1 | 1 | 0.98936 | 0.9661 | 0.9875 | 1 | 0.99401 | 0.98052 | 0.9434 | 0.99729 | 0.99154 | 0.94681 | 0.95181 | 0.99357 | 0.979843 |
| 106 | 1 | 1 | 1 | 1 | 0.93671 | 1 | 1 | 0.98936 | 0.9661 | 0.975 | 1 | 0.99202 | 0.98052 | 0.9434 | 0.99729 | 0.99154 | 0.94681 | 0.95181 | 0.99357 | 0.979843 |
| 107 | 1 | 1 | 1 | 1 | 0.93671 | 1 | 1 | 0.98936 | 0.9661 | 0.975 | 1 | 0.99202 | 0.98052 | 0.9434 | 0.99729 | 0.99154 | 0.94681 | 0.95181 | 0.99357 | 0.979843 |
| 108 | 1 | 1 | 1 | 1 | 0.92405 | 1 | 1 | 0.98723 | 0.9661 | 0.9875 | 1 | 0.99401 | 0.98052 | 0.96226 | 0.99594 | 0.99154 | 0.95213 | 0.96386 | 0.99357 | 0.982083 |
| 109 | 1 | 1 | 1 | 1 | 0.92405 | 1 | 1 | 0.98723 | 0.9661 | 0.9875 | 1 | 0.99401 | 0.98052 | 0.96226 | 0.99594 | 0.99154 | 0.95213 | 0.96386 | 0.99357 | 0.982083 |
| 110 | 1 | 1 | 1 | 1 | 0.93671 | 1 | 1 | 0.98936 | 0.9661 | 0.9875 | 1 | 0.99401 | 0.98052 | 0.96226 | 0.99459 | 0.99049 | 0.95213 | 0.96386 | 0.99357 | 0.982083 |
| 111 | 1 | 1 | 1 | 1 | 0.93671 | 1 | 1 | 0.98936 | 0.9661 | 0.975 | 1 | 0.99202 | 0.98052 | 0.96226 | 0.99459 | 0.99049 | 0.95213 | 0.95181 | 0.99357 | 0.980963 |
| 112 | 1 | 1 | 1 | 1 | 0.93671 | 1 | 1 | 0.98936 | 0.9661 | 0.9875 | 1 | 0.99401 | 0.98052 | 0.98113 | 0.99459 | 0.99154 | 0.95213 | 0.96386 | 0.99357 | 0.982083 |
| 113 | 1 | 1 | 1 | 1 | 0.93671 | 1 | 1 | 0.98936 | 0.9661 | 0.9875 | 1 | 0.99401 | 0.98052 | 0.98113 | 0.99459 | 0.99154 | 0.95213 | 0.96386 | 0.99357 | 0.982083 |
| 114 | 1 | 1 | 1 | 1 | 0.92405 | 1 | 1 | 0.98723 | 0.9661 | 0.9875 | 1 | 0.99401 | 0.98052 | 0.98113 | 0.99459 | 0.99154 | 0.95213 | 0.96386 | 0.99357 | 0.982083 |
| 115 | 1 | 1 | 1 | 1 | 0.93671 | 1 | 1 | 0.98936 | 0.9661 | 0.9875 | 1 | 0.99401 | 0.98052 | 0.98113 | 0.99459 | 0.99154 | 0.95745 | 0.96386 | 0.99357 | 0.983203 |
| 116 | 1 | 1 | 1 | 1 | 0.93671 | 1 | 1 | 0.98936 | 0.9661 | 0.9625 | 1 | 0.99002 | 0.98052 | 0.96226 | 0.99459 | 0.99049 | 0.95745 | 0.96386 | 0.99357 | 0.983203 |
| 117 | 1 | 1 | 1 | 1 | 0.92405 | 1 | 1 | 0.98723 | 0.9661 | 0.9625 | 1 | 0.99002 | 0.98052 | 0.96226 | 0.99459 | 0.99049 | 0.95213 | 0.96386 | 0.99357 | 0.982083 |
| 118 | 1 | 1 | 1 | 1 | 0.92405 | 1 | 1 | 0.98723 | 0.9661 | 0.9625 | 1 | 0.99002 | 0.98052 | 0.96226 | 0.99459 | 0.99049 | 0.95213 | 0.96386 | 0.99357 | 0.982083 |
| 119 | 1 | 1 | 1 | 1 | 0.92405 | 1 | 1 | 0.98723 | 0.9661 | 0.9625 | 1 | 0.99002 | 0.98052 | 0.96226 | 0.99459 | 0.99049 | 0.95213 | 0.95181 | 0.99357 | 0.980963 |
| 120 | 1 | 1 | 1 | 1 | 0.92405 | 1 | 1 | 0.98723 | 0.9661 | 0.9625 | 1 | 0.99002 | 0.98052 | 0.96226 | 0.99459 | 0.99049 | 0.95213 | 0.95181 | 0.99357 | 0.980963 |
| 121 | 1 | 1 | 1 | 1 | 0.92405 | 1 | 1 | 0.98723 | 0.9661 | 0.9625 | 1 | 0.99002 | 0.98052 | 0.96226 | 0.99459 | 0.99049 | 0.95745 | 0.95181 | 0.99357 | 0.982083 |
| 122 | 1 | 1 | 1 | 1 | 0.93671 | 1 | 1 | 0.98936 | 0.9661 | 0.95 | 1 | 0.98802 | 0.98052 | 0.96226 | 0.99459 | 0.99049 | 0.95213 | 0.93976 | 0.99357 | 0.979843 |
| 123 | 1 | 1 | 1 | 1 | 0.94937 | 1 | 1 | 0.99149 | 0.9661 | 0.95 | 1 | 0.98802 | 0.98052 | 0.96226 | 0.99459 | 0.99049 | 0.95213 | 0.93976 | 0.99357 | 0.979843 |
| 124 | 1 | 1 | 1 | 1 | 0.94937 | 1 | 1 | 0.99149 | 0.9661 | 0.95 | 1 | 0.98802 | 0.98052 | 0.96226 | 0.99459 | 0.99049 | 0.95213 | 0.93976 | 0.99357 | 0.979843 |
| 125 | 1 | 1 | 1 | 1 | 0.93671 | 1 | 1 | 0.98936 | 0.9661 | 0.95 | 1 | 0.98802 | 0.98052 | 0.96226 | 0.99459 | 0.99049 | 0.95213 | 0.95181 | 0.99357 | 0.980963 |
| 126 | 1 | 1 | 1 | 1 | 0.93671 | 1 | 1 | 0.98936 | 0.9661 | 0.95 | 1 | 0.98802 | 0.98052 | 0.96226 | 0.99459 | 0.99049 | 0.95213 | 0.93976 | 0.99357 | 0.979843 |
| 127 | 1 | 1 | 1 | 1 | 0.92405 | 1 | 1 | 0.98723 | 0.9661 | 0.95 | 1 | 0.98802 | 0.98052 | 0.96226 | 0.99459 | 0.99049 | 0.95213 | 0.93976 | 0.99357 | 0.979843 |
| 128 | 1 | 1 | 1 | 1 | 0.91139 | 1 | 1 | 0.98511 | 0.9661 | 0.95 | 1 | 0.98802 | 0.98052 | 0.96226 | 0.99594 | 0.99154 | 0.95213 | 0.93976 | 0.99357 | 0.979843 |
| 129 | 1 | 1 | 1 | 1 | 0.91139 | 1 | 1 | 0.98511 | 0.9661 | 0.95 | 1 | 0.98802 | 0.98052 | 0.96226 | 0.99459 | 0.99049 | 0.95745 | 0.95181 | 0.99357 | 0.982083 |
| 130 | 1 | 1 | 1 | 1 | 0.91139 | 1 | 1 | 0.98511 | 0.9661 | 0.9625 | 1 | 0.99002 | 0.98052 | 0.96226 | 0.99459 | 0.99049 | 0.95213 | 0.95181 | 0.99357 | 0.980963 |
| 131 | 1 | 1 | 1 | 1 | 0.92405 | 1 | 1 | 0.98723 | 0.9661 | 0.9625 | 1 | 0.99002 | 0.98052 | 0.96226 | 0.99459 | 0.99049 | 0.95213 | 0.95181 | 0.99357 | 0.980963 |
| 132 | 1 | 1 | 1 | 1 | 0.93671 | 1 | 1 | 0.98936 | 0.9661 | 0.9625 | 1 | 0.99002 | 0.98052 | 0.96226 | 0.99459 | 0.99049 | 0.96277 | 0.96386 | 0.99357 | 0.984323 |
| 133 | 1 | 1 | 1 | 1 | 0.93671 | 1 | 1 | 0.98936 | 0.9661 | 0.9625 | 1 | 0.99002 | 0.98052 | 0.96226 | 0.99459 | 0.99049 | 0.96277 | 0.96386 | 0.99357 | 0.984323 |
| 134 | 0.96154 | 1 | 1 | 0.99725 | 0.93671 | 1 | 1 | 0.98936 | 0.9661 | 0.9625 | 1 | 0.99002 | 0.98052 | 0.96226 | 0.99729 | 0.9926 | 0.95745 | 0.96386 | 0.99357 | 0.983203 |
| 135 | 0.96154 | 1 | 1 | 0.99725 | 0.93671 | 1 | 1 | 0.98936 | 0.9661 | 0.9625 | 1 | 0.99002 | 0.98052 | 0.96226 | 0.99729 | 0.9926 | 0.96277 | 0.96386 | 0.99357 | 0.984323 |
| 136 | 0.96154 | 1 | 1 | 0.99725 | 0.93671 | 1 | 1 | 0.98936 | 0.9661 | 0.9625 | 1 | 0.99002 | 0.98052 | 0.96226 | 0.99729 | 0.9926 | 0.95745 | 0.96386 | 0.99357 | 0.983203 |
| 137 | 0.96154 | 1 | 1 | 0.99725 | 0.93671 | 1 | 1 | 0.98936 | 0.9661 | 0.9625 | 1 | 0.99002 | 0.98701 | 0.96226 | 0.99729 | 0.99366 | 0.96277 | 0.96386 | 0.99357 | 0.984323 |
| 138 | 0.96154 | 1 | 1 | 0.99725 | 0.93671 | 1 | 1 | 0.98936 | 0.9661 | 0.9625 | 1 | 0.99002 | 0.98701 | 0.96226 | 0.99729 | 0.99366 | 0.96277 | 0.96386 | 0.99357 | 0.984323 |
| 139 | 0.96154 | 1 | 1 | 0.99725 | 0.92405 | 1 | 1 | 0.98723 | 0.9661 | 0.9625 | 1 | 0.99002 | 0.98052 | 0.96226 | 0.99729 | 0.9926 | 0.96277 | 0.96386 | 0.99357 | 0.984323 |
| 140 | 0.96154 | 1 | 1 | 0.99725 | 0.92405 | 1 | 1 | 0.98723 | 0.9661 | 0.9625 | 1 | 0.99002 | 0.98052 | 0.96226 | 0.99729 | 0.9926 | 0.96277 | 0.96386 | 0.99357 | 0.984323 |
| 141 | 1 | 1 | 1 | 1 | 0.92405 | 1 | 1 | 0.98723 | 0.9322 | 0.9625 | 1 | 0.98603 | 0.98052 | 0.96226 | 0.99729 | 0.9926 | 0.95745 | 0.96386 | 0.99357 | 0.983203 |
| 142 | 1 | 1 | 1 | 1 | 0.92405 | 1 | 1 | 0.98723 | 0.9322 | 0.9625 | 1 | 0.98603 | 0.98052 | 0.96226 | 0.99729 | 0.9926 | 0.95745 | 0.96386 | 0.99357 | 0.983203 |
| 143 | 1 | 1 | 1 | 1 | 0.92405 | 1 | 1 | 0.98723 | 0.9322 | 0.9625 | 1 | 0.98603 | 0.98052 | 0.96226 | 0.99729 | 0.9926 | 0.95745 | 0.96386 | 0.99357 | 0.983203 |
| 144 | 1 | 1 | 1 | 1 | 0.92405 | 1 | 1 | 0.98723 | 0.9322 | 0.9625 | 1 | 0.98603 | 0.98052 | 0.96226 | 0.99729 | 0.9926 | 0.95745 | 0.96386 | 0.99357 | 0.983203 |
| 145 | 1 | 1 | 0.99661 | 0.99725 | 0.91139 | 1 | 1 | 0.98511 | 0.9322 | 0.9625 | 1 | 0.98603 | 0.97403 | 0.96226 | 0.99729 | 0.99154 | 0.95213 | 0.96386 | 0.99357 | 0.982083 |
| 146 | 1 | 1 | 0.99661 | 0.99725 | 0.91139 | 1 | 1 | 0.98511 | 0.9322 | 0.9625 | 1 | 0.98603 | 0.97403 | 0.96226 | 0.99729 | 0.99154 | 0.95213 | 0.96386 | 0.99357 | 0.982083 |
| 147 | 1 | 1 | 0.99661 | 0.99725 | 0.91139 | 1 | 1 | 0.98511 | 0.9322 | 0.975 | 1 | 0.98802 | 0.98052 | 0.96226 | 0.99729 | 0.9926 | 0.95213 | 0.96386 | 0.99357 | 0.982083 |
| 148 | 1 | 1 | 0.99661 | 0.99725 | 0.91139 | 1 | 1 | 0.98511 | 0.9322 | 0.975 | 1 | 0.98802 | 0.98052 | 0.96226 | 0.99729 | 0.9926 | 0.95213 | 0.96386 | 0.99357 | 0.982083 |
| 149 | 1 | 1 | 0.99661 | 0.99725 | 0.91139 | 1 | 1 | 0.98511 | 0.9322 | 0.975 | 1 | 0.98802 | 0.98052 | 0.96226 | 0.99729 | 0.9926 | 0.95213 | 0.96386 | 0.99357 | 0.982083 |
| 150 | 0.96154 | 1 | 0.99661 | 0.99449 | 0.91139 | 1 | 1 | 0.98511 | 0.9322 | 0.975 | 1 | 0.98802 | 0.98052 | 0.96226 | 0.99729 | 0.9926 | 0.95213 | 0.96386 | 0.99357 | 0.982083 |
| 151 | 1 | 1 | 0.99661 | 0.99725 | 0.91139 | 1 | 1 | 0.98511 | 0.9322 | 0.975 | 1 | 0.98802 | 0.97403 | 0.96226 | 0.99729 | 0.99154 | 0.95213 | 0.96386 | 0.99518 | 0.983203 |
| 152 | 1 | 1 | 0.99661 | 0.99725 | 0.89873 | 1 | 1 | 0.98298 | 0.9322 | 0.975 | 1 | 0.98802 | 0.97403 | 0.96226 | 0.99729 | 0.99154 | 0.95213 | 0.96386 | 0.99518 | 0.983203 |
| 153 | 1 | 1 | 0.99661 | 0.99725 | 0.91139 | 1 | 1 | 0.98511 | 0.9322 | 0.975 | 1 | 0.98802 | 0.97403 | 0.96226 | 0.99729 | 0.99154 | 0.95213 | 0.96386 | 0.99679 | 0.984323 |
| 154 | 1 | 1 | 0.99661 | 0.99725 | 0.89873 | 1 | 1 | 0.98298 | 0.9322 | 0.975 | 1 | 0.98802 | 0.98052 | 0.96226 | 0.99729 | 0.9926 | 0.95213 | 0.96386 | 0.99679 | 0.984323 |
| 155 | 1 | 1 | 1 | 1 | 0.91139 | 1 | 1 | 0.98511 | 0.9322 | 0.975 | 1 | 0.98802 | 0.97403 | 0.96226 | 0.99729 | 0.99154 | 0.95213 | 0.96386 | 0.99679 | 0.984323 |
| 156 | 1 | 1 | 1 | 1 | 0.91139 | 1 | 1 | 0.98511 | 0.9322 | 0.975 | 1 | 0.98802 | 0.97403 | 0.96226 | 0.99729 | 0.99154 | 0.95213 | 0.96386 | 0.99679 | 0.984323 |
| 157 | 1 | 1 | 1 | 1 | 0.89873 | 1 | 1 | 0.98298 | 0.9322 | 0.975 | 1 | 0.98802 | 0.98052 | 0.96226 | 0.99729 | 0.9926 | 0.95213 | 0.96386 | 0.99679 | 0.984323 |
| 158 | 1 | 1 | 1 | 1 | 0.91139 | 1 | 1 | 0.98511 | 0.9322 | 0.975 | 1 | 0.98802 | 0.98052 | 0.96226 | 0.99729 | 0.9926 | 0.95213 | 0.96386 | 0.99679 | 0.984323 |
| 159 | 1 | 1 | 1 | 1 | 0.89873 | 1 | 1 | 0.98298 | 0.9322 | 0.975 | 1 | 0.98802 | 0.98052 | 0.96226 | 0.99729 | 0.9926 | 0.95213 | 0.96386 | 0.99679 | 0.984323 |
| 160 | 1 | 1 | 1 | 1 | 0.89873 | 1 | 1 | 0.98298 | 0.9322 | 0.975 | 1 | 0.98802 | 0.98052 | 0.96226 | 0.99729 | 0.9926 | 0.95213 | 0.96386 | 0.99679 | 0.984323 |
| 161 | 1 | 1 | 1 | 1 | 0.89873 | 1 | 1 | 0.98298 | 0.9322 | 0.975 | 1 | 0.98802 | 0.98052 | 0.96226 | 0.99729 | 0.9926 | 0.95213 | 0.96386 | 0.99679 | 0.984323 |
| 162 | 1 | 1 | 1 | 1 | 0.89873 | 1 | 1 | 0.98298 | 0.9322 | 0.975 | 1 | 0.98802 | 0.98052 | 0.96226 | 0.99729 | 0.9926 | 0.95213 | 0.96386 | 0.99679 | 0.984323 |
| 163 | 1 | 1 | 1 | 1 | 0.89873 | 1 | 1 | 0.98298 | 0.9322 | 0.9625 | 1 | 0.98603 | 0.98052 | 0.96226 | 0.99729 | 0.9926 | 0.95213 | 0.96386 | 0.99679 | 0.984323 |
| 164 | 1 | 1 | 1 | 1 | 0.89873 | 1 | 1 | 0.98298 | 0.9322 | 0.9625 | 1 | 0.98603 | 0.98052 | 0.96226 | 0.99729 | 0.9926 | 0.95213 | 0.96386 | 0.99518 | 0.983203 |
| 165 | 1 | 1 | 1 | 1 | 0.91139 | 1 | 1 | 0.98511 | 0.9322 | 0.9625 | 1 | 0.98603 | 0.98052 | 0.96226 | 0.99729 | 0.9926 | 0.95213 | 0.96386 | 0.99518 | 0.983203 |
| 166 | 1 | 1 | 1 | 1 | 0.91139 | 1 | 1 | 0.98511 | 0.9322 | 0.9625 | 1 | 0.98603 | 0.98052 | 0.96226 | 0.99729 | 0.9926 | 0.95213 | 0.96386 | 0.99518 | 0.983203 |
| 167 | 1 | 1 | 1 | 1 | 0.91139 | 1 | 1 | 0.98511 | 0.9322 | 0.9625 | 1 | 0.98603 | 0.98701 | 0.96226 | 0.99729 | 0.99366 | 0.95213 | 0.96386 | 0.99518 | 0.983203 |
| 168 | 1 | 1 | 1 | 1 | 0.91139 | 1 | 1 | 0.98511 | 0.94915 | 0.9625 | 1 | 0.98802 | 0.98052 | 0.96226 | 0.99729 | 0.9926 | 0.95213 | 0.95181 | 0.99679 | 0.983203 |
| 169 | 1 | 1 | 1 | 1 | 0.91139 | 1 | 1 | 0.98511 | 0.94915 | 0.9625 | 1 | 0.98802 | 0.98052 | 0.96226 | 0.99729 | 0.9926 | 0.95213 | 0.95181 | 0.99679 | 0.983203 |
| 170 | 1 | 1 | 1 | 1 | 0.91139 | 1 | 1 | 0.98511 | 0.94915 | 0.9625 | 1 | 0.98802 | 0.98052 | 0.96226 | 0.99729 | 0.9926 | 0.95213 | 0.95181 | 0.99679 | 0.983203 |
| 171 | 1 | 1 | 1 | 1 | 0.91139 | 1 | 1 | 0.98511 | 0.94915 | 0.9625 | 1 | 0.98802 | 0.98052 | 0.96226 | 0.99729 | 0.9926 | 0.96277 | 0.95181 | 0.99518 | 0.984323 |
| 172 | 1 | 1 | 1 | 1 | 0.91139 | 1 | 1 | 0.98511 | 0.94915 | 0.9625 | 1 | 0.98802 | 0.98052 | 0.96226 | 0.99729 | 0.9926 | 0.96277 | 0.95181 | 0.99518 | 0.984323 |
| 173 | 1 | 1 | 1 | 1 | 0.91139 | 1 | 1 | 0.98511 | 0.94915 | 0.9625 | 1 | 0.98802 | 0.98052 | 0.96226 | 0.99729 | 0.9926 | 0.96277 | 0.95181 | 0.99518 | 0.984323 |
| 174 | 1 | 1 | 1 | 1 | 0.91139 | 1 | 1 | 0.98511 | 0.94915 | 0.9625 | 1 | 0.98802 | 0.98052 | 0.96226 | 0.99729 | 0.9926 | 0.96277 | 0.95181 | 0.99518 | 0.984323 |
| 175 | 1 | 1 | 1 | 1 | 0.91139 | 1 | 1 | 0.98511 | 0.94915 | 0.9625 | 1 | 0.98802 | 0.98052 | 0.96226 | 0.99729 | 0.9926 | 0.96277 | 0.95181 | 0.99357 | 0.983203 |
| 176 | 1 | 1 | 1 | 1 | 0.91139 | 1 | 1 | 0.98511 | 0.94915 | 0.9625 | 1 | 0.98802 | 0.98052 | 0.96226 | 0.99729 | 0.9926 | 0.96277 | 0.95181 | 0.99357 | 0.983203 |
| 177 | 1 | 1 | 1 | 1 | 0.91139 | 1 | 1 | 0.98511 | 0.94915 | 0.975 | 1 | 0.99002 | 0.98052 | 0.96226 | 0.99729 | 0.9926 | 0.95745 | 0.96386 | 0.99357 | 0.983203 |
| 178 | 1 | 1 | 1 | 1 | 0.91139 | 1 | 1 | 0.98511 | 0.94915 | 0.975 | 1 | 0.99002 | 0.98052 | 0.96226 | 0.99729 | 0.9926 | 0.96277 | 0.95181 | 0.99357 | 0.983203 |
| 179 | 1 | 1 | 1 | 1 | 0.91139 | 1 | 1 | 0.98511 | 0.94915 | 0.975 | 1 | 0.99002 | 0.98052 | 0.96226 | 0.99729 | 0.9926 | 0.95745 | 0.95181 | 0.99196 | 0.980963 |
| 180 | 1 | 1 | 1 | 1 | 0.91139 | 1 | 1 | 0.98511 | 0.94915 | 0.975 | 1 | 0.99002 | 0.98052 | 0.96226 | 0.99729 | 0.9926 | 0.96277 | 0.95181 | 0.99196 | 0.982083 |
| 181 | 1 | 1 | 1 | 1 | 0.91139 | 1 | 1 | 0.98511 | 0.94915 | 0.975 | 1 | 0.99002 | 0.98052 | 0.96226 | 0.99729 | 0.9926 | 0.96277 | 0.96386 | 0.99196 | 0.983203 |
| 182 | 1 | 1 | 1 | 1 | 0.91139 | 1 | 1 | 0.98511 | 0.94915 | 0.975 | 1 | 0.99002 | 0.98052 | 0.96226 | 0.99729 | 0.9926 | 0.95745 | 0.96386 | 0.99196 | 0.982083 |
| 183 | 1 | 1 | 1 | 1 | 0.91139 | 1 | 1 | 0.98511 | 0.9661 | 0.975 | 1 | 0.99202 | 0.98052 | 0.96226 | 0.99729 | 0.9926 | 0.95745 | 0.96386 | 0.99357 | 0.983203 |
| 184 | 1 | 1 | 1 | 1 | 0.91139 | 1 | 1 | 0.98511 | 0.9661 | 0.975 | 1 | 0.99202 | 0.98052 | 0.96226 | 0.99729 | 0.9926 | 0.95213 | 0.96386 | 0.99357 | 0.982083 |
| 185 | 1 | 1 | 1 | 1 | 0.91139 | 1 | 1 | 0.98511 | 0.9661 | 0.95 | 1 | 0.98802 | 0.98052 | 0.96226 | 0.99729 | 0.9926 | 0.95745 | 0.96386 | 0.99518 | 0.984323 |
| 186 | 1 | 1 | 1 | 1 | 0.91139 | 1 | 1 | 0.98511 | 0.9661 | 0.95 | 1 | 0.98802 | 0.98052 | 0.96226 | 0.99729 | 0.9926 | 0.95213 | 0.96386 | 0.99518 | 0.983203 |
| 187 | 1 | 1 | 1 | 1 | 0.91139 | 1 | 1 | 0.98511 | 0.9661 | 0.95 | 1 | 0.98802 | 0.98052 | 0.96226 | 0.99729 | 0.9926 | 0.95213 | 0.96386 | 0.99518 | 0.983203 |
| 188 | 1 | 1 | 1 | 1 | 0.91139 | 1 | 1 | 0.98511 | 0.9661 | 0.95 | 1 | 0.98802 | 0.98052 | 0.96226 | 0.99729 | 0.9926 | 0.95213 | 0.96386 | 0.99518 | 0.983203 |
| 189 | 1 | 1 | 1 | 1 | 0.91139 | 1 | 1 | 0.98511 | 0.9661 | 0.95 | 1 | 0.98802 | 0.98052 | 0.96226 | 0.99729 | 0.9926 | 0.95213 | 0.96386 | 0.99518 | 0.983203 |
| 190 | 1 | 1 | 1 | 1 | 0.91139 | 1 | 1 | 0.98511 | 0.9661 | 0.95 | 1 | 0.98802 | 0.98052 | 0.96226 | 0.99729 | 0.9926 | 0.95213 | 0.96386 | 0.99518 | 0.983203 |
| 191 | 1 | 1 | 1 | 1 | 0.91139 | 1 | 1 | 0.98511 | 0.9661 | 0.95 | 1 | 0.98802 | 0.98052 | 0.96226 | 0.99729 | 0.9926 | 0.95213 | 0.96386 | 0.99518 | 0.983203 |
| 192 | 1 | 1 | 1 | 1 | 0.91139 | 1 | 1 | 0.98511 | 0.9661 | 0.95 | 1 | 0.98802 | 0.98052 | 0.96226 | 0.99729 | 0.9926 | 0.95745 | 0.96386 | 0.99518 | 0.984323 |
| 193 | 1 | 1 | 1 | 1 | 0.91139 | 1 | 1 | 0.98511 | 0.94915 | 0.9625 | 1 | 0.98802 | 0.98052 | 0.96226 | 0.99729 | 0.9926 | 0.95745 | 0.95181 | 0.99518 | 0.983203 |
| 194 | 1 | 1 | 1 | 1 | 0.91139 | 1 | 1 | 0.98511 | 0.9661 | 0.9625 | 1 | 0.99002 | 0.98052 | 0.96226 | 0.99729 | 0.9926 | 0.95745 | 0.95181 | 0.99518 | 0.983203 |
| 195 | 1 | 1 | 1 | 1 | 0.91139 | 1 | 1 | 0.98511 | 0.9661 | 0.9625 | 1 | 0.99002 | 0.98052 | 0.9434 | 0.99729 | 0.99154 | 0.96277 | 0.95181 | 0.99357 | 0.983203 |
| 196 | 1 | 1 | 1 | 1 | 0.91139 | 1 | 1 | 0.98511 | 0.9661 | 0.9625 | 1 | 0.99002 | 0.98052 | 0.9434 | 0.99729 | 0.99154 | 0.95745 | 0.95181 | 0.99518 | 0.983203 |
| 197 | 1 | 1 | 1 | 1 | 0.91139 | 1 | 1 | 0.98511 | 0.94915 | 0.9625 | 1 | 0.98802 | 0.98052 | 0.96226 | 0.99729 | 0.9926 | 0.95213 | 0.95181 | 0.99518 | 0.982083 |
| 198 | 1 | 1 | 1 | 1 | 0.91139 | 1 | 1 | 0.98511 | 0.94915 | 0.9625 | 1 | 0.98802 | 0.98052 | 0.96226 | 0.99729 | 0.9926 | 0.95213 | 0.95181 | 0.99518 | 0.982083 |
| 199 | 1 | 1 | 1 | 1 | 0.91139 | 1 | 1 | 0.98511 | 0.9661 | 0.9625 | 1 | 0.99002 | 0.98052 | 0.96226 | 0.99729 | 0.9926 | 0.95213 | 0.95181 | 0.99357 | 0.980963 |
| 200 | 1 | 1 | 1 | 1 | 0.91139 | 1 | 1 | 0.98511 | 0.9661 | 0.95 | 1 | 0.98802 | 0.98052 | 0.96226 | 0.99729 | 0.9926 | 0.95213 | 0.95181 | 0.99357 | 0.980963 |
| 201 | 1 | 1 | 1 | 1 | 0.91139 | 1 | 1 | 0.98511 | 0.9661 | 0.95 | 1 | 0.98802 | 0.98052 | 0.96226 | 0.99729 | 0.9926 | 0.95213 | 0.95181 | 0.99357 | 0.980963 |
| 202 | 1 | 1 | 1 | 1 | 0.91139 | 1 | 1 | 0.98511 | 0.9661 | 0.95 | 1 | 0.98802 | 0.98052 | 0.96226 | 0.99729 | 0.9926 | 0.95213 | 0.95181 | 0.99357 | 0.980963 |
| 203 | 1 | 1 | 1 | 1 | 0.91139 | 1 | 1 | 0.98511 | 0.94915 | 0.95 | 1 | 0.98603 | 0.98052 | 0.9434 | 0.99729 | 0.99154 | 0.95213 | 0.95181 | 0.99679 | 0.983203 |
| 204 | 1 | 1 | 1 | 1 | 0.91139 | 1 | 1 | 0.98511 | 0.94915 | 0.95 | 1 | 0.98603 | 0.98052 | 0.96226 | 0.99729 | 0.9926 | 0.95213 | 0.95181 | 0.99679 | 0.983203 |
| 205 | 1 | 1 | 1 | 1 | 0.91139 | 1 | 1 | 0.98511 | 0.94915 | 0.95 | 1 | 0.98603 | 0.98052 | 0.9434 | 0.99729 | 0.99154 | 0.95213 | 0.95181 | 0.99679 | 0.983203 |
| 206 | 1 | 1 | 1 | 1 | 0.91139 | 1 | 1 | 0.98511 | 0.94915 | 0.95 | 1 | 0.98603 | 0.98052 | 0.9434 | 0.99729 | 0.99154 | 0.95213 | 0.95181 | 0.99679 | 0.983203 |
| 207 | 1 | 1 | 1 | 1 | 0.91139 | 1 | 1 | 0.98511 | 0.94915 | 0.95 | 1 | 0.98603 | 0.98052 | 0.96226 | 0.99729 | 0.9926 | 0.95745 | 0.95181 | 0.99518 | 0.983203 |
| 208 | 1 | 1 | 1 | 1 | 0.91139 | 1 | 1 | 0.98511 | 0.94915 | 0.95 | 1 | 0.98603 | 0.98052 | 0.96226 | 0.99729 | 0.9926 | 0.95745 | 0.95181 | 0.99679 | 0.984323 |
| 209 | 1 | 1 | 1 | 1 | 0.91139 | 1 | 1 | 0.98511 | 0.94915 | 0.95 | 1 | 0.98603 | 0.98052 | 0.96226 | 0.99729 | 0.9926 | 0.95745 | 0.95181 | 0.99679 | 0.984323 |
| 210 | 1 | 1 | 1 | 1 | 0.91139 | 1 | 1 | 0.98511 | 0.94915 | 0.95 | 1 | 0.98603 | 0.98052 | 0.96226 | 0.99729 | 0.9926 | 0.95745 | 0.95181 | 0.99679 | 0.984323 |
| 211 | 1 | 1 | 1 | 1 | 0.91139 | 1 | 1 | 0.98511 | 0.9322 | 0.95 | 1 | 0.98403 | 0.98052 | 0.96226 | 0.99729 | 0.9926 | 0.95745 | 0.95181 | 0.99679 | 0.984323 |
| 212 | 1 | 1 | 1 | 1 | 0.91139 | 1 | 1 | 0.98511 | 0.9322 | 0.9625 | 1 | 0.98603 | 0.98052 | 0.96226 | 0.99729 | 0.9926 | 0.95745 | 0.95181 | 0.99679 | 0.984323 |
| 213 | 1 | 1 | 1 | 1 | 0.91139 | 1 | 1 | 0.98511 | 0.94915 | 0.9625 | 1 | 0.98802 | 0.98052 | 0.96226 | 0.99729 | 0.9926 | 0.95745 | 0.95181 | 0.99518 | 0.983203 |
| 214 | 1 | 1 | 1 | 1 | 0.91139 | 1 | 1 | 0.98511 | 0.94915 | 0.9625 | 1 | 0.98802 | 0.98052 | 0.96226 | 0.99729 | 0.9926 | 0.95745 | 0.96386 | 0.99518 | 0.984323 |
| 215 | 1 | 1 | 1 | 1 | 0.91139 | 1 | 1 | 0.98511 | 0.9322 | 0.9625 | 1 | 0.98603 | 0.98052 | 0.96226 | 0.99729 | 0.9926 | 0.95745 | 0.96386 | 0.99518 | 0.984323 |
| 216 | 1 | 1 | 1 | 1 | 0.91139 | 1 | 1 | 0.98511 | 0.9322 | 0.95 | 1 | 0.98403 | 0.98052 | 0.96226 | 0.99729 | 0.9926 | 0.95745 | 0.95181 | 0.99518 | 0.983203 |
| 217 | 1 | 1 | 1 | 1 | 0.91139 | 1 | 1 | 0.98511 | 0.9322 | 0.95 | 1 | 0.98403 | 0.98052 | 0.96226 | 0.99729 | 0.9926 | 0.95745 | 0.95181 | 0.99518 | 0.983203 |
| 218 | 1 | 1 | 1 | 1 | 0.91139 | 1 | 1 | 0.98511 | 0.9322 | 0.95 | 1 | 0.98403 | 0.98052 | 0.96226 | 0.99729 | 0.9926 | 0.95745 | 0.95181 | 0.99679 | 0.984323 |
| 219 | 1 | 1 | 1 | 1 | 0.91139 | 1 | 1 | 0.98511 | 0.9322 | 0.95 | 1 | 0.98403 | 0.98052 | 0.96226 | 0.99729 | 0.9926 | 0.95745 | 0.95181 | 0.99679 | 0.984323 |
| 220 | 1 | 1 | 1 | 1 | 0.91139 | 1 | 1 | 0.98511 | 0.9322 | 0.9625 | 1 | 0.98603 | 0.98052 | 0.9434 | 0.99729 | 0.99154 | 0.95745 | 0.95181 | 0.99518 | 0.983203 |
| 221 | 1 | 1 | 1 | 1 | 0.91139 | 1 | 1 | 0.98511 | 0.9322 | 0.9625 | 1 | 0.98603 | 0.98052 | 0.9434 | 0.99729 | 0.99154 | 0.95745 | 0.95181 | 0.99518 | 0.983203 |
| 222 | 1 | 1 | 1 | 1 | 0.91139 | 1 | 1 | 0.98511 | 0.9322 | 0.9625 | 1 | 0.98603 | 0.98052 | 0.9434 | 0.99729 | 0.99154 | 0.95745 | 0.95181 | 0.99518 | 0.983203 |
| 223 | 1 | 1 | 1 | 1 | 0.91139 | 1 | 1 | 0.98511 | 0.9322 | 0.9625 | 1 | 0.98603 | 0.98052 | 0.9434 | 0.99729 | 0.99154 | 0.95745 | 0.95181 | 0.99518 | 0.983203 |
| 224 | 1 | 1 | 1 | 1 | 0.91139 | 1 | 1 | 0.98511 | 0.9322 | 0.9625 | 1 | 0.98603 | 0.98052 | 0.9434 | 0.99729 | 0.99154 | 0.95745 | 0.95181 | 0.99518 | 0.983203 |
| 225 | 1 | 1 | 1 | 1 | 0.91139 | 1 | 1 | 0.98511 | 0.9322 | 0.9625 | 1 | 0.98603 | 0.98052 | 0.9434 | 0.99729 | 0.99154 | 0.95745 | 0.96386 | 0.99518 | 0.984323 |
| 226 | 1 | 1 | 1 | 1 | 0.91139 | 1 | 1 | 0.98511 | 0.9322 | 0.9625 | 1 | 0.98603 | 0.98052 | 0.9434 | 0.99594 | 0.99049 | 0.95745 | 0.96386 | 0.99518 | 0.984323 |
| 227 | 1 | 1 | 1 | 1 | 0.91139 | 1 | 1 | 0.98511 | 0.9322 | 0.9625 | 1 | 0.98603 | 0.98052 | 0.9434 | 0.99729 | 0.99154 | 0.95745 | 0.96386 | 0.99518 | 0.984323 |
| 228 | 1 | 1 | 1 | 1 | 0.91139 | 1 | 1 | 0.98511 | 0.9322 | 0.9625 | 1 | 0.98603 | 0.98052 | 0.9434 | 0.99729 | 0.99154 | 0.95745 | 0.96386 | 0.99518 | 0.984323 |
| 229 | 1 | 1 | 1 | 1 | 0.91139 | 1 | 1 | 0.98511 | 0.9322 | 0.9625 | 1 | 0.98603 | 0.98052 | 0.9434 | 0.99729 | 0.99154 | 0.95745 | 0.96386 | 0.99518 | 0.984323 |
| 230 | 1 | 1 | 1 | 1 | 0.91139 | 1 | 1 | 0.98511 | 0.9322 | 0.95 | 1 | 0.98403 | 0.98052 | 0.9434 | 0.99594 | 0.99049 | 0.95745 | 0.96386 | 0.99518 | 0.984323 |
| 231 | 1 | 1 | 1 | 1 | 0.91139 | 1 | 1 | 0.98511 | 0.9322 | 0.95 | 1 | 0.98403 | 0.98052 | 0.9434 | 0.99594 | 0.99049 | 0.95745 | 0.96386 | 0.99518 | 0.984323 |
| 232 | 1 | 1 | 1 | 1 | 0.91139 | 1 | 1 | 0.98511 | 0.9322 | 0.95 | 1 | 0.98403 | 0.98052 | 0.92453 | 0.99729 | 0.99049 | 0.95745 | 0.96386 | 1 | 0.987682 |
| 233 | 1 | 1 | 1 | 1 | 0.91139 | 1 | 1 | 0.98511 | 0.9322 | 0.95 | 1 | 0.98403 | 0.98052 | 0.9434 | 0.99594 | 0.99049 | 0.96277 | 0.96386 | 1 | 0.988802 |
| 234 | 1 | 1 | 1 | 1 | 0.91139 | 1 | 1 | 0.98511 | 0.9322 | 0.95 | 1 | 0.98403 | 0.98052 | 0.9434 | 0.99865 | 0.9926 | 0.95213 | 0.96386 | 1 | 0.986562 |
| 235 | 1 | 1 | 1 | 1 | 0.89873 | 1 | 1 | 0.98298 | 0.9322 | 0.95 | 1 | 0.98403 | 0.98052 | 0.9434 | 0.99865 | 0.9926 | 0.95213 | 0.96386 | 1 | 0.986562 |
| 236 | 1 | 1 | 1 | 1 | 0.89873 | 1 | 1 | 0.98298 | 0.9322 | 0.95 | 1 | 0.98403 | 0.98052 | 0.9434 | 0.99865 | 0.9926 | 0.95213 | 0.96386 | 1 | 0.986562 |
| 237 | 1 | 1 | 1 | 1 | 0.89873 | 1 | 1 | 0.98298 | 0.9322 | 0.95 | 1 | 0.98403 | 0.98052 | 0.9434 | 0.99865 | 0.9926 | 0.95213 | 0.96386 | 1 | 0.986562 |
| 238 | 1 | 1 | 1 | 1 | 0.89873 | 1 | 1 | 0.98298 | 0.9322 | 0.95 | 1 | 0.98403 | 0.98052 | 0.9434 | 0.99865 | 0.9926 | 0.95213 | 0.9759 | 1 | 0.987682 |
| 239 | 1 | 1 | 1 | 1 | 0.89873 | 1 | 1 | 0.98298 | 0.9322 | 0.95 | 1 | 0.98403 | 0.98052 | 0.9434 | 0.99865 | 0.9926 | 0.95213 | 0.9759 | 1 | 0.987682 |
| 240 | 1 | 1 | 1 | 1 | 0.89873 | 1 | 1 | 0.98298 | 0.9322 | 0.95 | 1 | 0.98403 | 0.98052 | 0.9434 | 0.99865 | 0.9926 | 0.95213 | 0.9759 | 1 | 0.987682 |
| 241 | 1 | 1 | 1 | 1 | 0.89873 | 1 | 1 | 0.98298 | 0.9322 | 0.95 | 1 | 0.98403 | 0.98052 | 0.9434 | 0.99865 | 0.9926 | 0.95745 | 0.9759 | 1 | 0.988802 |
| 242 | 1 | 1 | 1 | 1 | 0.89873 | 1 | 1 | 0.98298 | 0.9322 | 0.95 | 1 | 0.98403 | 0.98052 | 0.9434 | 0.99865 | 0.9926 | 0.95745 | 0.9759 | 1 | 0.988802 |
| 243 | 1 | 1 | 1 | 1 | 0.89873 | 1 | 1 | 0.98298 | 0.9322 | 0.95 | 1 | 0.98403 | 0.98701 | 0.9434 | 0.99729 | 0.9926 | 0.95745 | 0.9759 | 1 | 0.988802 |
| 244 | 1 | 1 | 1 | 1 | 0.89873 | 1 | 1 | 0.98298 | 0.9322 | 0.95 | 1 | 0.98403 | 0.98701 | 0.9434 | 0.99729 | 0.9926 | 0.95745 | 0.9759 | 1 | 0.988802 |
| 245 | 1 | 1 | 1 | 1 | 0.89873 | 1 | 1 | 0.98298 | 0.9322 | 0.95 | 1 | 0.98403 | 0.98701 | 0.9434 | 0.99729 | 0.9926 | 0.95745 | 0.9759 | 1 | 0.988802 |
| 246 | 1 | 1 | 1 | 1 | 0.89873 | 1 | 1 | 0.98298 | 0.9322 | 0.95 | 1 | 0.98403 | 0.98701 | 0.9434 | 0.99729 | 0.9926 | 0.95745 | 0.96386 | 1 | 0.987682 |
| 247 | 1 | 1 | 1 | 1 | 0.89873 | 1 | 1 | 0.98298 | 0.9322 | 0.95 | 1 | 0.98403 | 0.98701 | 0.9434 | 0.99729 | 0.9926 | 0.95745 | 0.96386 | 1 | 0.987682 |
| 248 | 1 | 1 | 1 | 1 | 0.89873 | 1 | 1 | 0.98298 | 0.9322 | 0.95 | 1 | 0.98403 | 0.98701 | 0.9434 | 0.99729 | 0.9926 | 0.95745 | 0.9759 | 1 | 0.988802 |
| 249 | 1 | 1 | 1 | 1 | 0.89873 | 1 | 1 | 0.98298 | 0.9322 | 0.95 | 1 | 0.98403 | 0.98701 | 0.9434 | 0.99729 | 0.9926 | 0.95213 | 0.9759 | 1 | 0.987682 |
| 250 | 1 | 1 | 1 | 1 | 0.93671 | 1 | 1 | 0.98936 | 0.94915 | 0.95 | 1 | 0.98603 | 0.98701 | 0.9434 | 0.99323 | 0.98943 | 0.96277 | 0.9759 | 1 | 0.989922 |
| 251 | 1 | 1 | 1 | 1 | 0.93671 | 1 | 1 | 0.98936 | 0.9322 | 0.95 | 1 | 0.98403 | 0.98701 | 0.9434 | 0.99459 | 0.99049 | 0.95745 | 0.9759 | 0.99839 | 0.987682 |
| 252 | 1 | 1 | 1 | 1 | 0.93671 | 1 | 1 | 0.98936 | 0.9322 | 0.95 | 1 | 0.98403 | 0.98701 | 0.9434 | 0.99323 | 0.98943 | 0.95745 | 0.9759 | 0.99839 | 0.987682 |
| 253 | 1 | 1 | 1 | 1 | 0.93671 | 1 | 1 | 0.98936 | 0.9322 | 0.95 | 1 | 0.98403 | 0.98701 | 0.9434 | 0.99323 | 0.98943 | 0.95745 | 0.9759 | 0.99839 | 0.987682 |
| 254 | 1 | 1 | 1 | 1 | 0.93671 | 1 | 1 | 0.98936 | 0.9322 | 0.95 | 1 | 0.98403 | 0.98701 | 0.9434 | 0.99323 | 0.98943 | 0.95745 | 0.9759 | 0.99839 | 0.987682 |
| 255 | 1 | 1 | 1 | 1 | 0.93671 | 1 | 1 | 0.98936 | 0.9322 | 0.95 | 1 | 0.98403 | 0.98701 | 0.9434 | 0.99323 | 0.98943 | 0.96277 | 0.9759 | 0.99839 | 0.988802 |
| 256 | 1 | 1 | 1 | 1 | 0.93671 | 1 | 1 | 0.98936 | 0.94915 | 0.95 | 1 | 0.98603 | 0.98701 | 0.9434 | 0.99323 | 0.98943 | 0.96277 | 0.9759 | 0.99839 | 0.988802 |
| 257 | 1 | 1 | 1 | 1 | 0.93671 | 1 | 1 | 0.98936 | 0.94915 | 0.95 | 1 | 0.98603 | 0.98701 | 0.9434 | 0.99459 | 0.99049 | 0.96277 | 0.9759 | 0.99839 | 0.988802 |
| 258 | 1 | 1 | 1 | 1 | 0.93671 | 1 | 1 | 0.98936 | 0.94915 | 0.95 | 1 | 0.98603 | 0.98701 | 0.9434 | 0.99459 | 0.99049 | 0.96277 | 0.9759 | 0.99839 | 0.988802 |
| 259 | 1 | 1 | 1 | 1 | 0.93671 | 1 | 1 | 0.98936 | 0.94915 | 0.95 | 1 | 0.98603 | 0.98701 | 0.9434 | 0.99459 | 0.99049 | 0.96277 | 0.9759 | 0.99839 | 0.988802 |
| 260 | 1 | 1 | 1 | 1 | 0.93671 | 1 | 1 | 0.98936 | 0.94915 | 0.95 | 1 | 0.98603 | 0.98701 | 0.9434 | 0.99459 | 0.99049 | 0.96277 | 0.9759 | 0.99839 | 0.988802 |
| 261 | 1 | 1 | 1 | 1 | 0.93671 | 1 | 1 | 0.98936 | 0.94915 | 0.95 | 1 | 0.98603 | 0.98701 | 0.9434 | 0.99459 | 0.99049 | 0.96809 | 0.96386 | 0.99839 | 0.988802 |
| 262 | 1 | 1 | 1 | 1 | 0.93671 | 1 | 1 | 0.98936 | 0.94915 | 0.95 | 1 | 0.98603 | 0.98052 | 0.9434 | 0.99459 | 0.98943 | 0.95213 | 0.96386 | 1 | 0.986562 |
| 263 | 1 | 1 | 1 | 1 | 0.93671 | 1 | 1 | 0.98936 | 0.94915 | 0.95 | 1 | 0.98603 | 0.98052 | 0.9434 | 0.99459 | 0.98943 | 0.95213 | 0.9759 | 1 | 0.987682 |
| 264 | 1 | 1 | 1 | 1 | 0.93671 | 1 | 1 | 0.98936 | 0.94915 | 0.95 | 1 | 0.98603 | 0.98052 | 0.9434 | 0.99865 | 0.9926 | 0.94681 | 0.96386 | 1 | 0.985442 |
| 265 | 1 | 1 | 1 | 1 | 0.93671 | 1 | 1 | 0.98936 | 0.94915 | 0.95 | 1 | 0.98603 | 0.98052 | 0.9434 | 0.99865 | 0.9926 | 0.94681 | 0.9759 | 1 | 0.986562 |
| 266 | 1 | 1 | 1 | 1 | 0.93671 | 1 | 1 | 0.98936 | 0.94915 | 0.95 | 1 | 0.98603 | 0.98052 | 0.9434 | 0.99865 | 0.9926 | 0.94681 | 0.9759 | 1 | 0.986562 |
| 267 | 1 | 1 | 1 | 1 | 0.93671 | 1 | 1 | 0.98936 | 0.94915 | 0.95 | 1 | 0.98603 | 0.98052 | 0.9434 | 0.99865 | 0.9926 | 0.94681 | 0.9759 | 1 | 0.986562 |
| 268 | 1 | 1 | 1 | 1 | 0.93671 | 1 | 1 | 0.98936 | 0.94915 | 0.95 | 1 | 0.98603 | 0.98052 | 0.9434 | 0.99865 | 0.9926 | 0.95213 | 0.96386 | 1 | 0.986562 |
| 269 | 1 | 1 | 1 | 1 | 0.93671 | 1 | 1 | 0.98936 | 0.94915 | 0.95 | 1 | 0.98603 | 0.98052 | 0.9434 | 0.99865 | 0.9926 | 0.95213 | 0.96386 | 1 | 0.986562 |
| 270 | 0.96154 | 1 | 1 | 0.99725 | 0.93671 | 1 | 1 | 0.98936 | 0.94915 | 0.95 | 1 | 0.98603 | 0.98052 | 0.9434 | 0.99865 | 0.9926 | 0.94681 | 0.96386 | 1 | 0.985442 |
| 271 | 0.96154 | 1 | 1 | 0.99725 | 0.93671 | 1 | 1 | 0.98936 | 0.94915 | 0.95 | 1 | 0.98603 | 0.97403 | 0.9434 | 0.99865 | 0.99154 | 0.94681 | 0.96386 | 1 | 0.985442 |
| 272 | 0.96154 | 1 | 1 | 0.99725 | 0.93671 | 1 | 1 | 0.98936 | 0.94915 | 0.95 | 1 | 0.98603 | 0.97403 | 0.9434 | 0.99865 | 0.99154 | 0.94681 | 0.96386 | 1 | 0.985442 |
| 273 | 0.96154 | 1 | 1 | 0.99725 | 0.93671 | 1 | 1 | 0.98936 | 0.94915 | 0.95 | 1 | 0.98603 | 0.96753 | 0.9434 | 0.99865 | 0.99049 | 0.94681 | 0.96386 | 1 | 0.985442 |
| 274 | 0.96154 | 1 | 1 | 0.99725 | 0.93671 | 1 | 1 | 0.98936 | 0.94915 | 0.95 | 1 | 0.98603 | 0.96753 | 0.9434 | 0.99865 | 0.99049 | 0.94681 | 0.96386 | 1 | 0.985442 |
| 275 | 0.96154 | 1 | 1 | 0.99725 | 0.92405 | 1 | 1 | 0.98723 | 0.94915 | 0.95 | 1 | 0.98603 | 0.97403 | 0.9434 | 0.99865 | 0.99154 | 0.94681 | 0.96386 | 1 | 0.985442 |
| 276 | 0.96154 | 1 | 1 | 0.99725 | 0.92405 | 1 | 1 | 0.98723 | 0.94915 | 0.95 | 1 | 0.98603 | 0.97403 | 0.9434 | 0.99865 | 0.99154 | 0.94681 | 0.96386 | 1 | 0.985442 |
| 277 | 0.96154 | 1 | 1 | 0.99725 | 0.92405 | 1 | 1 | 0.98723 | 0.94915 | 0.95 | 1 | 0.98603 | 0.97403 | 0.9434 | 0.99865 | 0.99154 | 0.94681 | 0.96386 | 1 | 0.985442 |
| 278 | 0.96154 | 1 | 1 | 0.99725 | 0.92405 | 1 | 1 | 0.98723 | 0.94915 | 0.95 | 1 | 0.98603 | 0.97403 | 0.9434 | 0.99865 | 0.99154 | 0.94681 | 0.96386 | 1 | 0.985442 |
| 279 | 0.96154 | 1 | 1 | 0.99725 | 0.92405 | 1 | 1 | 0.98723 | 0.94915 | 0.95 | 1 | 0.98603 | 0.98052 | 0.9434 | 0.99865 | 0.9926 | 0.94681 | 0.96386 | 1 | 0.985442 |
| 280 | 0.96154 | 1 | 1 | 0.99725 | 0.92405 | 1 | 1 | 0.98723 | 0.9322 | 0.95 | 1 | 0.98403 | 0.98052 | 0.9434 | 0.99865 | 0.9926 | 0.94681 | 0.96386 | 1 | 0.985442 |
| 281 | 0.96154 | 1 | 1 | 0.99725 | 0.92405 | 1 | 1 | 0.98723 | 0.9322 | 0.95 | 1 | 0.98403 | 0.98052 | 0.9434 | 0.99865 | 0.9926 | 0.94681 | 0.96386 | 1 | 0.985442 |
| 282 | 0.96154 | 1 | 1 | 0.99725 | 0.92405 | 1 | 1 | 0.98723 | 0.9322 | 0.95 | 1 | 0.98403 | 0.97403 | 0.9434 | 0.99865 | 0.99154 | 0.94681 | 0.96386 | 1 | 0.985442 |
| 283 | 0.96154 | 1 | 1 | 0.99725 | 0.92405 | 1 | 1 | 0.98723 | 0.9322 | 0.95 | 1 | 0.98403 | 0.97403 | 0.9434 | 0.99865 | 0.99154 | 0.94681 | 0.96386 | 1 | 0.985442 |
| 284 | 0.96154 | 1 | 1 | 0.99725 | 0.92405 | 1 | 1 | 0.98723 | 0.9322 | 0.95 | 1 | 0.98403 | 0.97403 | 0.9434 | 0.99865 | 0.99154 | 0.94681 | 0.96386 | 1 | 0.985442 |
| 285 | 0.96154 | 1 | 1 | 0.99725 | 0.92405 | 1 | 1 | 0.98723 | 0.9322 | 0.95 | 1 | 0.98403 | 0.97403 | 0.9434 | 0.99865 | 0.99154 | 0.94681 | 0.96386 | 1 | 0.985442 |
| 286 | 0.96154 | 1 | 1 | 0.99725 | 0.92405 | 1 | 1 | 0.98723 | 0.91525 | 0.95 | 1 | 0.98204 | 0.97403 | 0.9434 | 0.99865 | 0.99154 | 0.94681 | 0.96386 | 1 | 0.985442 |
| 287 | 0.96154 | 1 | 1 | 0.99725 | 0.92405 | 1 | 1 | 0.98723 | 0.91525 | 0.95 | 1 | 0.98204 | 0.97403 | 0.9434 | 0.99865 | 0.99154 | 0.94681 | 0.96386 | 1 | 0.985442 |
| 288 | 0.96154 | 1 | 1 | 0.99725 | 0.92405 | 1 | 1 | 0.98723 | 0.91525 | 0.95 | 1 | 0.98204 | 0.97403 | 0.9434 | 0.99865 | 0.99154 | 0.94681 | 0.96386 | 1 | 0.985442 |
| 289 | 0.96154 | 1 | 1 | 0.99725 | 0.92405 | 1 | 1 | 0.98723 | 0.91525 | 0.95 | 1 | 0.98204 | 0.97403 | 0.9434 | 0.99865 | 0.99154 | 0.94681 | 0.96386 | 1 | 0.985442 |
| 290 | 0.96154 | 1 | 1 | 0.99725 | 0.92405 | 1 | 1 | 0.98723 | 0.91525 | 0.95 | 1 | 0.98204 | 0.97403 | 0.9434 | 0.99865 | 0.99154 | 0.94681 | 0.96386 | 1 | 0.985442 |
| 291 | 0.96154 | 1 | 1 | 0.99725 | 0.92405 | 1 | 1 | 0.98723 | 0.91525 | 0.95 | 1 | 0.98204 | 0.97403 | 0.9434 | 0.99865 | 0.99154 | 0.94681 | 0.96386 | 1 | 0.985442 |
| 292 | 0.96154 | 1 | 1 | 0.99725 | 0.92405 | 1 | 1 | 0.98723 | 0.91525 | 0.95 | 1 | 0.98204 | 0.97403 | 0.9434 | 0.99865 | 0.99154 | 0.94681 | 0.96386 | 1 | 0.985442 |
| 293 | 0.96154 | 1 | 1 | 0.99725 | 0.92405 | 1 | 1 | 0.98723 | 0.9322 | 0.95 | 1 | 0.98403 | 0.97403 | 0.9434 | 0.99865 | 0.99154 | 0.94681 | 0.96386 | 1 | 0.985442 |
| 294 | 0.96154 | 1 | 1 | 0.99725 | 0.92405 | 1 | 1 | 0.98723 | 0.9322 | 0.95 | 1 | 0.98403 | 0.98701 | 0.9434 | 0.99459 | 0.99049 | 0.95745 | 0.96386 | 1 | 0.987682 |
| 295 | 0.96154 | 1 | 1 | 0.99725 | 0.92405 | 1 | 1 | 0.98723 | 0.9322 | 0.95 | 1 | 0.98403 | 0.98701 | 0.9434 | 0.99459 | 0.99049 | 0.95745 | 0.96386 | 1 | 0.987682 |
| 296 | 0.96154 | 1 | 1 | 0.99725 | 0.92405 | 1 | 1 | 0.98723 | 0.9322 | 0.95 | 1 | 0.98403 | 0.98701 | 0.9434 | 0.99459 | 0.99049 | 0.95745 | 0.96386 | 1 | 0.987682 |
| 297 | 1 | 1 | 1 | 1 | 0.92405 | 1 | 1 | 0.98723 | 0.9322 | 0.95 | 1 | 0.98403 | 0.98701 | 0.92453 | 0.99459 | 0.98943 | 0.95745 | 0.95181 | 1 | 0.986562 |
| 298 | 0.96154 | 1 | 1 | 0.99725 | 0.92405 | 1 | 1 | 0.98723 | 0.9322 | 0.95 | 1 | 0.98403 | 0.98701 | 0.92453 | 0.99459 | 0.98943 | 0.95745 | 0.95181 | 1 | 0.986562 |
| 299 | 0.96154 | 1 | 1 | 0.99725 | 0.92405 | 1 | 1 | 0.98723 | 0.9322 | 0.95 | 1 | 0.98403 | 0.98701 | 0.92453 | 0.99459 | 0.98943 | 0.95745 | 0.95181 | 1 | 0.986562 |
| 300 | 0.96154 | 1 | 1 | 0.99725 | 0.92405 | 1 | 1 | 0.98723 | 0.9322 | 0.95 | 1 | 0.98403 | 0.98701 | 0.92453 | 0.99594 | 0.99049 | 0.95745 | 0.95181 | 1 | 0.986562 |
| 301 | 0.96154 | 1 | 1 | 0.99725 | 0.92405 | 1 | 1 | 0.98723 | 0.9322 | 0.95 | 1 | 0.98403 | 0.98701 | 0.92453 | 0.99594 | 0.99049 | 0.95745 | 0.95181 | 1 | 0.986562 |
| 302 | 0.96154 | 1 | 1 | 0.99725 | 0.92405 | 1 | 1 | 0.98723 | 0.9322 | 0.95 | 1 | 0.98403 | 0.98701 | 0.92453 | 0.99594 | 0.99049 | 0.95745 | 0.95181 | 1 | 0.986562 |
| 303 | 0.96154 | 1 | 1 | 0.99725 | 0.92405 | 1 | 1 | 0.98723 | 0.9322 | 0.95 | 1 | 0.98403 | 0.98701 | 0.92453 | 0.99594 | 0.99049 | 0.95745 | 0.95181 | 0.99839 | 0.985442 |
| 304 | 1 | 1 | 1 | 1 | 0.92405 | 1 | 1 | 0.98723 | 0.9322 | 0.95 | 1 | 0.98403 | 0.98701 | 0.92453 | 0.99459 | 0.98943 | 0.95745 | 0.93976 | 0.99839 | 0.984323 |
| 305 | 0.96154 | 1 | 1 | 0.99725 | 0.91139 | 1 | 1 | 0.98511 | 0.9322 | 0.95 | 1 | 0.98403 | 0.98701 | 0.92453 | 0.99459 | 0.98943 | 0.95745 | 0.93976 | 0.99839 | 0.984323 |
| 306 | 0.96154 | 1 | 1 | 0.99725 | 0.91139 | 1 | 1 | 0.98511 | 0.91525 | 0.95 | 1 | 0.98204 | 0.98052 | 0.92453 | 0.99459 | 0.98837 | 0.95745 | 0.95181 | 0.99839 | 0.985442 |
| 307 | 0.96154 | 1 | 1 | 0.99725 | 0.91139 | 1 | 1 | 0.98511 | 0.91525 | 0.95 | 1 | 0.98204 | 0.98052 | 0.92453 | 0.99459 | 0.98837 | 0.95745 | 0.92771 | 0.99839 | 0.983203 |
| 308 | 0.96154 | 1 | 1 | 0.99725 | 0.91139 | 1 | 1 | 0.98511 | 0.91525 | 0.95 | 1 | 0.98204 | 0.98052 | 0.92453 | 0.99459 | 0.98837 | 0.95745 | 0.92771 | 0.99839 | 0.983203 |
| 309 | 0.96154 | 1 | 1 | 0.99725 | 0.91139 | 1 | 1 | 0.98511 | 0.9322 | 0.95 | 1 | 0.98403 | 0.98701 | 0.92453 | 0.99323 | 0.98837 | 0.95745 | 0.92771 | 0.99839 | 0.983203 |
| 310 | 0.96154 | 1 | 1 | 0.99725 | 0.92405 | 1 | 1 | 0.98723 | 0.9322 | 0.95 | 1 | 0.98403 | 0.98052 | 0.92453 | 0.99594 | 0.98943 | 0.95745 | 0.92771 | 1 | 0.984323 |
| 311 | 0.96154 | 1 | 1 | 0.99725 | 0.92405 | 1 | 1 | 0.98723 | 0.9322 | 0.95 | 1 | 0.98403 | 0.98052 | 0.92453 | 0.99594 | 0.98943 | 0.95213 | 0.92771 | 1 | 0.983203 |
| 312 | 0.96154 | 1 | 1 | 0.99725 | 0.92405 | 1 | 1 | 0.98723 | 0.9322 | 0.95 | 1 | 0.98403 | 0.98052 | 0.92453 | 0.99594 | 0.98943 | 0.95213 | 0.92771 | 1 | 0.983203 |
| 313 | 0.96154 | 1 | 1 | 0.99725 | 0.92405 | 1 | 1 | 0.98723 | 0.9322 | 0.95 | 1 | 0.98403 | 0.98052 | 0.92453 | 0.99594 | 0.98943 | 0.95745 | 0.92771 | 1 | 0.984323 |
| 314 | 0.96154 | 1 | 1 | 0.99725 | 0.92405 | 1 | 1 | 0.98723 | 0.9322 | 0.95 | 1 | 0.98403 | 0.98052 | 0.92453 | 0.99594 | 0.98943 | 0.95745 | 0.92771 | 1 | 0.984323 |
| 315 | 0.96154 | 1 | 1 | 0.99725 | 0.92405 | 1 | 1 | 0.98723 | 0.9322 | 0.95 | 1 | 0.98403 | 0.98052 | 0.92453 | 0.99594 | 0.98943 | 0.95745 | 0.92771 | 1 | 0.984323 |
| 316 | 0.96154 | 1 | 1 | 0.99725 | 0.92405 | 1 | 1 | 0.98723 | 0.9322 | 0.95 | 1 | 0.98403 | 0.98052 | 0.92453 | 0.99594 | 0.98943 | 0.95745 | 0.92771 | 1 | 0.984323 |
| 317 | 0.96154 | 1 | 1 | 0.99725 | 0.92405 | 1 | 1 | 0.98723 | 0.9322 | 0.95 | 1 | 0.98403 | 0.98052 | 0.92453 | 0.99594 | 0.98943 | 0.95745 | 0.92771 | 1 | 0.984323 |
| 318 | 0.96154 | 1 | 1 | 0.99725 | 0.92405 | 1 | 1 | 0.98723 | 0.9322 | 0.95 | 1 | 0.98403 | 0.98052 | 0.92453 | 0.99594 | 0.98943 | 0.95745 | 0.92771 | 1 | 0.984323 |
| 319 | 0.96154 | 1 | 1 | 0.99725 | 0.92405 | 1 | 1 | 0.98723 | 0.9322 | 0.95 | 1 | 0.98403 | 0.98052 | 0.92453 | 0.99594 | 0.98943 | 0.95745 | 0.92771 | 1 | 0.984323 |
| 320 | 0.96154 | 1 | 1 | 0.99725 | 0.92405 | 1 | 1 | 0.98723 | 0.9322 | 0.95 | 1 | 0.98403 | 0.98052 | 0.92453 | 0.99594 | 0.98943 | 0.95745 | 0.92771 | 1 | 0.984323 |
| 321 | 0.96154 | 1 | 1 | 0.99725 | 0.92405 | 1 | 1 | 0.98723 | 0.9322 | 0.95 | 1 | 0.98403 | 0.98052 | 0.92453 | 0.99594 | 0.98943 | 0.95745 | 0.92771 | 1 | 0.984323 |
| 322 | 0.96154 | 1 | 1 | 0.99725 | 0.92405 | 1 | 1 | 0.98723 | 0.9322 | 0.95 | 1 | 0.98403 | 0.98052 | 0.92453 | 0.99594 | 0.98943 | 0.95745 | 0.92771 | 1 | 0.984323 |
| 323 | 0.96154 | 1 | 1 | 0.99725 | 0.92405 | 1 | 1 | 0.98723 | 0.9322 | 0.95 | 1 | 0.98403 | 0.98052 | 0.92453 | 0.99594 | 0.98943 | 0.95745 | 0.92771 | 1 | 0.984323 |
| 324 | 0.96154 | 1 | 1 | 0.99725 | 0.92405 | 1 | 1 | 0.98723 | 0.9322 | 0.95 | 1 | 0.98403 | 0.98052 | 0.92453 | 0.99594 | 0.98943 | 0.95745 | 0.93976 | 1 | 0.985442 |
| 325 | 0.96154 | 1 | 1 | 0.99725 | 0.92405 | 1 | 1 | 0.98723 | 0.9322 | 0.95 | 1 | 0.98403 | 0.98052 | 0.92453 | 0.99594 | 0.98943 | 0.95213 | 0.93976 | 1 | 0.984323 |
| 326 | 0.96154 | 1 | 1 | 0.99725 | 0.92405 | 1 | 1 | 0.98723 | 0.9322 | 0.95 | 1 | 0.98403 | 0.98052 | 0.92453 | 0.99594 | 0.98943 | 0.95745 | 0.92771 | 1 | 0.984323 |
| 327 | 0.96154 | 1 | 1 | 0.99725 | 0.92405 | 1 | 1 | 0.98723 | 0.9322 | 0.95 | 1 | 0.98403 | 0.98052 | 0.92453 | 0.99594 | 0.98943 | 0.95213 | 0.93976 | 1 | 0.984323 |
| 328 | 0.96154 | 1 | 1 | 0.99725 | 0.92405 | 1 | 1 | 0.98723 | 0.9322 | 0.95 | 1 | 0.98403 | 0.98052 | 0.92453 | 0.99594 | 0.98943 | 0.95213 | 0.93976 | 1 | 0.984323 |
| 329 | 0.96154 | 1 | 1 | 0.99725 | 0.92405 | 1 | 1 | 0.98723 | 0.9322 | 0.95 | 1 | 0.98403 | 0.98052 | 0.92453 | 0.99594 | 0.98943 | 0.95213 | 0.93976 | 1 | 0.984323 |
| 330 | 0.96154 | 1 | 1 | 0.99725 | 0.92405 | 1 | 1 | 0.98723 | 0.9322 | 0.95 | 1 | 0.98403 | 0.98052 | 0.92453 | 0.99594 | 0.98943 | 0.95213 | 0.93976 | 1 | 0.984323 |
| 331 | 0.96154 | 1 | 1 | 0.99725 | 0.92405 | 1 | 1 | 0.98723 | 0.9322 | 0.95 | 1 | 0.98403 | 0.98052 | 0.92453 | 0.99594 | 0.98943 | 0.95213 | 0.93976 | 1 | 0.984323 |
| 332 | 0.96154 | 1 | 1 | 0.99725 | 0.92405 | 1 | 1 | 0.98723 | 0.9322 | 0.95 | 1 | 0.98403 | 0.98052 | 0.92453 | 0.99594 | 0.98943 | 0.95745 | 0.92771 | 1 | 0.984323 |
| 333 | 0.96154 | 1 | 1 | 0.99725 | 0.91139 | 1 | 1 | 0.98511 | 0.9322 | 0.95 | 1 | 0.98403 | 0.98052 | 0.92453 | 0.99594 | 0.98943 | 0.95213 | 0.95181 | 1 | 0.985442 |
| 334 | 0.96154 | 1 | 1 | 0.99725 | 0.89873 | 1 | 1 | 0.98298 | 0.9322 | 0.95 | 1 | 0.98403 | 0.98052 | 0.92453 | 0.99594 | 0.98943 | 0.95745 | 0.95181 | 1 | 0.986562 |
| 335 | 0.96154 | 1 | 1 | 0.99725 | 0.89873 | 1 | 1 | 0.98298 | 0.9322 | 0.95 | 1 | 0.98403 | 0.98052 | 0.92453 | 0.99594 | 0.98943 | 0.95213 | 0.95181 | 1 | 0.985442 |
| 336 | 0.96154 | 1 | 1 | 0.99725 | 0.89873 | 1 | 1 | 0.98298 | 0.9322 | 0.95 | 1 | 0.98403 | 0.98052 | 0.92453 | 0.99594 | 0.98943 | 0.94681 | 0.93976 | 1 | 0.983203 |
| 337 | 0.96154 | 1 | 1 | 0.99725 | 0.89873 | 1 | 1 | 0.98298 | 0.9322 | 0.95 | 1 | 0.98403 | 0.98052 | 0.92453 | 0.99594 | 0.98943 | 0.94681 | 0.93976 | 1 | 0.983203 |
| 338 | 0.96154 | 1 | 1 | 0.99725 | 0.89873 | 1 | 1 | 0.98298 | 0.9322 | 0.95 | 1 | 0.98403 | 0.98052 | 0.92453 | 0.99594 | 0.98943 | 0.94681 | 0.93976 | 1 | 0.983203 |
| 339 | 0.96154 | 1 | 1 | 0.99725 | 0.89873 | 1 | 1 | 0.98298 | 0.9322 | 0.95 | 1 | 0.98403 | 0.98052 | 0.92453 | 0.99594 | 0.98943 | 0.94681 | 0.95181 | 1 | 0.984323 |
| 340 | 0.96154 | 1 | 1 | 0.99725 | 0.89873 | 1 | 1 | 0.98298 | 0.9322 | 0.95 | 1 | 0.98403 | 0.98052 | 0.92453 | 0.99594 | 0.98943 | 0.94681 | 0.95181 | 1 | 0.984323 |
| 341 | 0.96154 | 1 | 1 | 0.99725 | 0.89873 | 1 | 1 | 0.98298 | 0.9322 | 0.95 | 1 | 0.98403 | 0.98052 | 0.92453 | 0.99594 | 0.98943 | 0.94681 | 0.95181 | 1 | 0.984323 |
| 342 | 0.96154 | 1 | 1 | 0.99725 | 0.89873 | 1 | 1 | 0.98298 | 0.9322 | 0.95 | 1 | 0.98403 | 0.98052 | 0.92453 | 0.99594 | 0.98943 | 0.94681 | 0.93976 | 1 | 0.983203 |
| 343 | 0.96154 | 1 | 1 | 0.99725 | 0.89873 | 1 | 1 | 0.98298 | 0.9322 | 0.95 | 1 | 0.98403 | 0.98052 | 0.92453 | 0.99594 | 0.98943 | 0.94681 | 0.93976 | 1 | 0.983203 |
| 344 | 0.96154 | 1 | 1 | 0.99725 | 0.89873 | 1 | 1 | 0.98298 | 0.9322 | 0.95 | 1 | 0.98403 | 0.98052 | 0.92453 | 0.99594 | 0.98943 | 0.94681 | 0.95181 | 1 | 0.984323 |
| 345 | 0.96154 | 1 | 1 | 0.99725 | 0.89873 | 1 | 1 | 0.98298 | 0.9322 | 0.95 | 1 | 0.98403 | 0.98052 | 0.92453 | 0.99594 | 0.98943 | 0.94681 | 0.93976 | 1 | 0.983203 |
| 346 | 0.96154 | 1 | 1 | 0.99725 | 0.89873 | 1 | 1 | 0.98298 | 0.9322 | 0.95 | 1 | 0.98403 | 0.98052 | 0.92453 | 0.99729 | 0.99049 | 0.94681 | 0.93976 | 1 | 0.983203 |
| 347 | 0.96154 | 1 | 1 | 0.99725 | 0.89873 | 1 | 1 | 0.98298 | 0.9322 | 0.95 | 1 | 0.98403 | 0.98052 | 0.92453 | 0.99594 | 0.98943 | 0.94681 | 0.93976 | 1 | 0.983203 |
| 348 | 0.96154 | 1 | 1 | 0.99725 | 0.89873 | 1 | 1 | 0.98298 | 0.9322 | 0.95 | 1 | 0.98403 | 0.98052 | 0.92453 | 0.99594 | 0.98943 | 0.94681 | 0.93976 | 1 | 0.983203 |
| 349 | 0.96154 | 1 | 1 | 0.99725 | 0.91139 | 1 | 1 | 0.98511 | 0.9322 | 0.95 | 1 | 0.98403 | 0.98052 | 0.92453 | 0.99594 | 0.98943 | 0.94681 | 0.93976 | 1 | 0.983203 |
| 350 | 0.96154 | 1 | 1 | 0.99725 | 0.91139 | 1 | 1 | 0.98511 | 0.9322 | 0.95 | 1 | 0.98403 | 0.98052 | 0.92453 | 0.99594 | 0.98943 | 0.95213 | 0.95181 | 1 | 0.985442 |
| 351 | 0.96154 | 1 | 1 | 0.99725 | 0.91139 | 1 | 1 | 0.98511 | 0.9322 | 0.95 | 1 | 0.98403 | 0.98052 | 0.92453 | 0.99594 | 0.98943 | 0.95213 | 0.95181 | 1 | 0.985442 |
| 352 | 0.96154 | 1 | 1 | 0.99725 | 0.91139 | 1 | 1 | 0.98511 | 0.9322 | 0.95 | 1 | 0.98403 | 0.98052 | 0.92453 | 0.99594 | 0.98943 | 0.95213 | 0.93976 | 1 | 0.984323 |
| 353 | 0.96154 | 1 | 1 | 0.99725 | 0.92405 | 1 | 1 | 0.98723 | 0.9322 | 0.95 | 1 | 0.98403 | 0.98052 | 0.92453 | 0.99594 | 0.98943 | 0.94681 | 0.93976 | 1 | 0.983203 |
| 354 | 0.96154 | 1 | 1 | 0.99725 | 0.93671 | 1 | 1 | 0.98936 | 0.9322 | 0.95 | 1 | 0.98403 | 0.98052 | 0.92453 | 0.99594 | 0.98943 | 0.95213 | 0.93976 | 1 | 0.984323 |
| 355 | 0.96154 | 1 | 1 | 0.99725 | 0.93671 | 1 | 1 | 0.98936 | 0.9322 | 0.95 | 1 | 0.98403 | 0.98052 | 0.92453 | 0.99594 | 0.98943 | 0.94681 | 0.93976 | 1 | 0.983203 |
| 356 | 0.96154 | 1 | 1 | 0.99725 | 0.93671 | 1 | 1 | 0.98936 | 0.9322 | 0.95 | 1 | 0.98403 | 0.98052 | 0.92453 | 0.99594 | 0.98943 | 0.94681 | 0.93976 | 1 | 0.983203 |
| 357 | 0.96154 | 1 | 1 | 0.99725 | 0.93671 | 1 | 1 | 0.98936 | 0.9322 | 0.95 | 1 | 0.98403 | 0.98052 | 0.92453 | 0.99729 | 0.99049 | 0.94681 | 0.95181 | 1 | 0.984323 |
| 358 | 0.96154 | 1 | 1 | 0.99725 | 0.93671 | 1 | 1 | 0.98936 | 0.9322 | 0.95 | 1 | 0.98403 | 0.98052 | 0.92453 | 0.99729 | 0.99049 | 0.94681 | 0.95181 | 1 | 0.984323 |
| 359 | 0.96154 | 1 | 1 | 0.99725 | 0.93671 | 1 | 1 | 0.98936 | 0.9322 | 0.95 | 1 | 0.98403 | 0.98052 | 0.92453 | 0.99729 | 0.99049 | 0.94681 | 0.95181 | 1 | 0.984323 |
| 360 | 0.96154 | 1 | 1 | 0.99725 | 0.89873 | 1 | 1 | 0.98298 | 0.9322 | 0.95 | 1 | 0.98403 | 0.98052 | 0.92453 | 0.99729 | 0.99049 | 0.94681 | 0.95181 | 1 | 0.984323 |
| 361 | 0.96154 | 1 | 1 | 0.99725 | 0.91139 | 1 | 1 | 0.98511 | 0.9322 | 0.95 | 1 | 0.98403 | 0.98052 | 0.92453 | 0.99594 | 0.98943 | 0.94681 | 0.95181 | 1 | 0.984323 |
| 362 | 0.96154 | 1 | 1 | 0.99725 | 0.91139 | 1 | 1 | 0.98511 | 0.9322 | 0.95 | 1 | 0.98403 | 0.98052 | 0.92453 | 0.99865 | 0.99154 | 0.94681 | 0.95181 | 1 | 0.984323 |
| 363 | 0.96154 | 1 | 1 | 0.99725 | 0.91139 | 1 | 1 | 0.98511 | 0.9322 | 0.95 | 1 | 0.98403 | 0.98052 | 0.92453 | 0.99865 | 0.99154 | 0.94681 | 0.95181 | 1 | 0.984323 |
| 364 | 0.96154 | 1 | 1 | 0.99725 | 0.91139 | 1 | 1 | 0.98511 | 0.9322 | 0.95 | 1 | 0.98403 | 0.98052 | 0.92453 | 0.99729 | 0.99049 | 0.94681 | 0.93976 | 1 | 0.983203 |
| 365 | 0.96154 | 1 | 1 | 0.99725 | 0.91139 | 1 | 1 | 0.98511 | 0.9322 | 0.95 | 1 | 0.98403 | 0.98052 | 0.92453 | 0.99729 | 0.99049 | 0.94681 | 0.95181 | 1 | 0.984323 |
| 366 | 0.96154 | 1 | 1 | 0.99725 | 0.91139 | 1 | 1 | 0.98511 | 0.9322 | 0.95 | 1 | 0.98403 | 0.98052 | 0.92453 | 0.99865 | 0.99154 | 0.94681 | 0.93976 | 1 | 0.983203 |
| 367 | 0.96154 | 1 | 1 | 0.99725 | 0.92405 | 1 | 1 | 0.98723 | 0.9322 | 0.95 | 1 | 0.98403 | 0.98052 | 0.92453 | 0.99865 | 0.99154 | 0.94681 | 0.95181 | 1 | 0.984323 |
| 368 | 0.96154 | 1 | 1 | 0.99725 | 0.92405 | 1 | 1 | 0.98723 | 0.9322 | 0.95 | 1 | 0.98403 | 0.98052 | 0.92453 | 0.99865 | 0.99154 | 0.94681 | 0.95181 | 1 | 0.984323 |
| 369 | 0.96154 | 1 | 1 | 0.99725 | 0.92405 | 1 | 1 | 0.98723 | 0.9322 | 0.95 | 1 | 0.98403 | 0.98052 | 0.92453 | 0.99865 | 0.99154 | 0.94681 | 0.95181 | 1 | 0.984323 |
| 370 | 0.96154 | 1 | 1 | 0.99725 | 0.92405 | 1 | 1 | 0.98723 | 0.9322 | 0.95 | 1 | 0.98403 | 0.98052 | 0.92453 | 0.99865 | 0.99154 | 0.94681 | 0.95181 | 1 | 0.984323 |
| 371 | 0.96154 | 1 | 1 | 0.99725 | 0.92405 | 1 | 1 | 0.98723 | 0.9322 | 0.95 | 1 | 0.98403 | 0.98052 | 0.92453 | 0.99865 | 0.99154 | 0.94681 | 0.95181 | 1 | 0.984323 |
| 372 | 0.96154 | 1 | 1 | 0.99725 | 0.92405 | 1 | 1 | 0.98723 | 0.9322 | 0.95 | 1 | 0.98403 | 0.98052 | 0.92453 | 0.99865 | 0.99154 | 0.94681 | 0.95181 | 1 | 0.984323 |
| 373 | 0.96154 | 1 | 1 | 0.99725 | 0.91139 | 1 | 1 | 0.98511 | 0.9322 | 0.95 | 1 | 0.98403 | 0.96753 | 0.92453 | 0.99865 | 0.98943 | 0.94681 | 0.95181 | 1 | 0.984323 |
| 374 | 0.96154 | 1 | 1 | 0.99725 | 0.91139 | 1 | 1 | 0.98511 | 0.9322 | 0.95 | 1 | 0.98403 | 0.97403 | 0.92453 | 0.99865 | 0.99049 | 0.94681 | 0.95181 | 1 | 0.984323 |
| 375 | 0.96154 | 1 | 1 | 0.99725 | 0.91139 | 1 | 1 | 0.98511 | 0.9322 | 0.95 | 1 | 0.98403 | 0.96753 | 0.92453 | 0.99865 | 0.98943 | 0.94681 | 0.93976 | 1 | 0.983203 |
| 376 | 0.96154 | 1 | 1 | 0.99725 | 0.91139 | 1 | 1 | 0.98511 | 0.9322 | 0.95 | 1 | 0.98403 | 0.96753 | 0.92453 | 0.99865 | 0.98943 | 0.94681 | 0.95181 | 1 | 0.984323 |
| 377 | 0.96154 | 1 | 1 | 0.99725 | 0.91139 | 1 | 1 | 0.98511 | 0.9322 | 0.95 | 1 | 0.98403 | 0.96753 | 0.92453 | 0.99865 | 0.98943 | 0.94681 | 0.95181 | 1 | 0.984323 |
| 378 | 0.96154 | 1 | 1 | 0.99725 | 0.91139 | 1 | 1 | 0.98511 | 0.9322 | 0.95 | 1 | 0.98403 | 0.96753 | 0.92453 | 0.99865 | 0.98943 | 0.94681 | 0.95181 | 1 | 0.984323 |
| 379 | 0.96154 | 1 | 1 | 0.99725 | 0.91139 | 1 | 1 | 0.98511 | 0.9322 | 0.95 | 1 | 0.98403 | 0.96753 | 0.92453 | 0.99865 | 0.98943 | 0.94149 | 0.95181 | 1 | 0.983203 |
| 380 | 0.96154 | 1 | 1 | 0.99725 | 0.91139 | 1 | 1 | 0.98511 | 0.9322 | 0.95 | 1 | 0.98403 | 0.96753 | 0.92453 | 0.99865 | 0.98943 | 0.94149 | 0.95181 | 1 | 0.983203 |
| 381 | 0.96154 | 1 | 1 | 0.99725 | 0.89873 | 1 | 1 | 0.98298 | 0.9322 | 0.95 | 1 | 0.98403 | 0.96753 | 0.92453 | 0.99865 | 0.98943 | 0.94149 | 0.95181 | 1 | 0.983203 |
| 382 | 0.96154 | 1 | 1 | 0.99725 | 0.89873 | 1 | 1 | 0.98298 | 0.9322 | 0.95 | 1 | 0.98403 | 0.96753 | 0.92453 | 0.99865 | 0.98943 | 0.94149 | 0.95181 | 1 | 0.983203 |
| 383 | 0.96154 | 1 | 1 | 0.99725 | 0.91139 | 1 | 1 | 0.98511 | 0.9322 | 0.95 | 1 | 0.98403 | 0.96753 | 0.92453 | 0.99865 | 0.98943 | 0.94149 | 0.95181 | 1 | 0.983203 |
| 384 | 0.96154 | 1 | 1 | 0.99725 | 0.91139 | 1 | 1 | 0.98511 | 0.9322 | 0.95 | 1 | 0.98403 | 0.96753 | 0.92453 | 0.99865 | 0.98943 | 0.94149 | 0.95181 | 1 | 0.983203 |
| 385 | 0.96154 | 1 | 1 | 0.99725 | 0.89873 | 1 | 1 | 0.98298 | 0.9322 | 0.95 | 1 | 0.98403 | 0.96753 | 0.92453 | 0.99865 | 0.98943 | 0.94149 | 0.95181 | 1 | 0.983203 |
| 386 | 0.96154 | 1 | 1 | 0.99725 | 0.89873 | 1 | 1 | 0.98298 | 0.9322 | 0.95 | 1 | 0.98403 | 0.96753 | 0.92453 | 0.99865 | 0.98943 | 0.94149 | 0.95181 | 1 | 0.983203 |
| 387 | 0.96154 | 1 | 1 | 0.99725 | 0.89873 | 1 | 1 | 0.98298 | 0.9322 | 0.95 | 1 | 0.98403 | 0.96753 | 0.92453 | 0.99865 | 0.98943 | 0.94149 | 0.95181 | 1 | 0.983203 |
| 388 | 0.96154 | 1 | 1 | 0.99725 | 0.89873 | 1 | 1 | 0.98298 | 0.9322 | 0.95 | 1 | 0.98403 | 0.96753 | 0.92453 | 0.99865 | 0.98943 | 0.94149 | 0.95181 | 1 | 0.983203 |
| 389 | 0.96154 | 1 | 1 | 0.99725 | 0.89873 | 1 | 1 | 0.98298 | 0.9322 | 0.95 | 1 | 0.98403 | 0.96753 | 0.92453 | 0.99865 | 0.98943 | 0.94149 | 0.95181 | 1 | 0.983203 |
| 390 | 0.96154 | 1 | 1 | 0.99725 | 0.89873 | 1 | 1 | 0.98298 | 0.9322 | 0.95 | 1 | 0.98403 | 0.96753 | 0.92453 | 0.99865 | 0.98943 | 0.94149 | 0.95181 | 1 | 0.983203 |
| 391 | 0.96154 | 1 | 1 | 0.99725 | 0.89873 | 1 | 1 | 0.98298 | 0.9322 | 0.95 | 1 | 0.98403 | 0.96753 | 0.92453 | 0.99865 | 0.98943 | 0.94149 | 0.95181 | 1 | 0.983203 |
| 392 | 0.96154 | 1 | 1 | 0.99725 | 0.89873 | 1 | 1 | 0.98298 | 0.9322 | 0.95 | 1 | 0.98403 | 0.96753 | 0.92453 | 0.99865 | 0.98943 | 0.94149 | 0.93976 | 1 | 0.982083 |
| 393 | 0.96154 | 1 | 1 | 0.99725 | 0.88608 | 1 | 1 | 0.98085 | 0.9322 | 0.95 | 1 | 0.98403 | 0.96753 | 0.92453 | 0.99865 | 0.98943 | 0.94149 | 0.93976 | 1 | 0.982083 |
| 394 | 0.96154 | 1 | 1 | 0.99725 | 0.89873 | 1 | 1 | 0.98298 | 0.9322 | 0.95 | 1 | 0.98403 | 0.96753 | 0.90566 | 0.99865 | 0.98837 | 0.94149 | 0.92771 | 1 | 0.980963 |
| 395 | 0.96154 | 1 | 1 | 0.99725 | 0.89873 | 1 | 1 | 0.98298 | 0.9322 | 0.95 | 1 | 0.98403 | 0.97403 | 0.90566 | 0.99865 | 0.98943 | 0.94149 | 0.92771 | 1 | 0.980963 |
| 396 | 0.96154 | 1 | 1 | 0.99725 | 0.89873 | 1 | 1 | 0.98298 | 0.9322 | 0.95 | 1 | 0.98403 | 0.97403 | 0.90566 | 0.99865 | 0.98943 | 0.94149 | 0.92771 | 1 | 0.980963 |
| 397 | 0.96154 | 1 | 1 | 0.99725 | 0.89873 | 1 | 1 | 0.98298 | 0.9322 | 0.95 | 1 | 0.98403 | 0.97403 | 0.90566 | 0.99865 | 0.98943 | 0.94149 | 0.92771 | 1 | 0.980963 |
| 398 | 0.96154 | 1 | 1 | 0.99725 | 0.89873 | 1 | 1 | 0.98298 | 0.9322 | 0.95 | 1 | 0.98403 | 0.97403 | 0.90566 | 0.99865 | 0.98943 | 0.94149 | 0.92771 | 1 | 0.980963 |
| 399 | 0.96154 | 1 | 1 | 0.99725 | 0.89873 | 1 | 1 | 0.98298 | 0.9322 | 0.95 | 1 | 0.98403 | 0.97403 | 0.90566 | 0.99865 | 0.98943 | 0.94149 | 0.92771 | 1 | 0.980963 |
| 400 | 0.96154 | 1 | 1 | 0.99725 | 0.89873 | 1 | 1 | 0.98298 | 0.9322 | 0.95 | 1 | 0.98403 | 0.97403 | 0.90566 | 0.99865 | 0.98943 | 0.94149 | 0.92771 | 1 | 0.980963 |
| 401 | 0.96154 | 1 | 1 | 0.99725 | 0.89873 | 1 | 1 | 0.98298 | 0.9322 | 0.95 | 1 | 0.98403 | 0.97403 | 0.90566 | 0.99865 | 0.98943 | 0.94149 | 0.92771 | 1 | 0.980963 |
| 402 | 0.96154 | 1 | 1 | 0.99725 | 0.89873 | 1 | 1 | 0.98298 | 0.9322 | 0.95 | 1 | 0.98403 | 0.96753 | 0.90566 | 0.99865 | 0.98837 | 0.93617 | 0.92771 | 1 | 0.979843 |
| 403 | 0.96154 | 1 | 1 | 0.99725 | 0.89873 | 1 | 1 | 0.98298 | 0.9322 | 0.95 | 1 | 0.98403 | 0.96753 | 0.90566 | 1 | 0.98943 | 0.92553 | 0.92771 | 1 | 0.977604 |
| 404 | 0.96154 | 1 | 1 | 0.99725 | 0.89873 | 1 | 1 | 0.98298 | 0.9322 | 0.95 | 1 | 0.98403 | 0.96753 | 0.88679 | 1 | 0.98837 | 0.92553 | 0.92771 | 1 | 0.977604 |
| 405 | 0.96154 | 1 | 1 | 0.99725 | 0.89873 | 1 | 1 | 0.98298 | 0.9322 | 0.95 | 1 | 0.98403 | 0.96753 | 0.88679 | 1 | 0.98837 | 0.92021 | 0.92771 | 1 | 0.976484 |
| 406 | 0.96154 | 1 | 1 | 0.99725 | 0.89873 | 1 | 1 | 0.98298 | 0.9322 | 0.95 | 1 | 0.98403 | 0.96753 | 0.90566 | 1 | 0.98943 | 0.92553 | 0.92771 | 1 | 0.977604 |
| 407 | 0.96154 | 1 | 1 | 0.99725 | 0.89873 | 1 | 1 | 0.98298 | 0.9322 | 0.95 | 1 | 0.98403 | 0.96753 | 0.90566 | 1 | 0.98943 | 0.92021 | 0.92771 | 1 | 0.976484 |
| 408 | 0.96154 | 1 | 1 | 0.99725 | 0.89873 | 1 | 1 | 0.98298 | 0.9322 | 0.95 | 1 | 0.98403 | 0.96753 | 0.90566 | 0.99865 | 0.98837 | 0.92021 | 0.92771 | 1 | 0.976484 |
| 409 | 0.96154 | 1 | 1 | 0.99725 | 0.89873 | 1 | 1 | 0.98298 | 0.9322 | 0.95 | 1 | 0.98403 | 0.96753 | 0.90566 | 0.99865 | 0.98837 | 0.92021 | 0.92771 | 1 | 0.976484 |
| 410 | 0.96154 | 1 | 1 | 0.99725 | 0.89873 | 1 | 1 | 0.98298 | 0.9322 | 0.95 | 1 | 0.98403 | 0.96753 | 0.90566 | 0.99865 | 0.98837 | 0.92021 | 0.92771 | 1 | 0.976484 |
| 411 | 0.96154 | 1 | 1 | 0.99725 | 0.89873 | 1 | 1 | 0.98298 | 0.9322 | 0.95 | 1 | 0.98403 | 0.96753 | 0.90566 | 0.99865 | 0.98837 | 0.92021 | 0.92771 | 1 | 0.976484 |
| 412 | 0.96154 | 1 | 1 | 0.99725 | 0.89873 | 1 | 1 | 0.98298 | 0.9322 | 0.95 | 1 | 0.98403 | 0.96753 | 0.90566 | 0.99865 | 0.98837 | 0.92021 | 0.92771 | 1 | 0.976484 |
| 413 | 0.96154 | 1 | 1 | 0.99725 | 0.89873 | 1 | 1 | 0.98298 | 0.9322 | 0.95 | 1 | 0.98403 | 0.96753 | 0.90566 | 1 | 0.98943 | 0.91489 | 0.92771 | 1 | 0.975364 |
| 414 | 0.96154 | 1 | 1 | 0.99725 | 0.89873 | 1 | 1 | 0.98298 | 0.9322 | 0.95 | 1 | 0.98403 | 0.96753 | 0.88679 | 1 | 0.98837 | 0.91489 | 0.92771 | 1 | 0.975364 |
| 415 | 0.96154 | 1 | 1 | 0.99725 | 0.89873 | 1 | 1 | 0.98298 | 0.9322 | 0.95 | 1 | 0.98403 | 0.96753 | 0.88679 | 1 | 0.98837 | 0.91489 | 0.92771 | 1 | 0.975364 |
| 416 | 0.96154 | 1 | 1 | 0.99725 | 0.89873 | 1 | 1 | 0.98298 | 0.9322 | 0.95 | 1 | 0.98403 | 0.96753 | 0.88679 | 1 | 0.98837 | 0.90957 | 0.92771 | 1 | 0.974244 |
| 417 | 0.96154 | 1 | 1 | 0.99725 | 0.89873 | 1 | 1 | 0.98298 | 0.9322 | 0.95 | 1 | 0.98403 | 0.96753 | 0.88679 | 1 | 0.98837 | 0.90957 | 0.92771 | 1 | 0.974244 |
| 418 | 0.96154 | 1 | 1 | 0.99725 | 0.89873 | 1 | 1 | 0.98298 | 0.9322 | 0.95 | 1 | 0.98403 | 0.96753 | 0.88679 | 1 | 0.98837 | 0.90957 | 0.92771 | 1 | 0.974244 |
| 419 | 0.96154 | 1 | 1 | 0.99725 | 0.89873 | 1 | 1 | 0.98298 | 0.9322 | 0.95 | 1 | 0.98403 | 0.96753 | 0.88679 | 1 | 0.98837 | 0.90957 | 0.92771 | 1 | 0.974244 |
| 420 | 0.96154 | 1 | 1 | 0.99725 | 0.89873 | 1 | 1 | 0.98298 | 0.9322 | 0.95 | 1 | 0.98403 | 0.96753 | 0.88679 | 1 | 0.98837 | 0.90426 | 0.92771 | 1 | 0.973124 |
| 421 | 0.96154 | 1 | 1 | 0.99725 | 0.89873 | 1 | 1 | 0.98298 | 0.9322 | 0.95 | 1 | 0.98403 | 0.96753 | 0.88679 | 1 | 0.98837 | 0.90426 | 0.92771 | 1 | 0.973124 |
| 422 | 0.96154 | 1 | 1 | 0.99725 | 0.89873 | 1 | 1 | 0.98298 | 0.9322 | 0.95 | 1 | 0.98403 | 0.96753 | 0.88679 | 1 | 0.98837 | 0.90957 | 0.92771 | 1 | 0.974244 |
| 423 | 0.96154 | 1 | 1 | 0.99725 | 0.89873 | 1 | 1 | 0.98298 | 0.9322 | 0.95 | 1 | 0.98403 | 0.96753 | 0.88679 | 1 | 0.98837 | 0.90957 | 0.92771 | 1 | 0.974244 |
| 424 | 0.96154 | 1 | 1 | 0.99725 | 0.89873 | 1 | 1 | 0.98298 | 0.9322 | 0.95 | 1 | 0.98403 | 0.96753 | 0.88679 | 1 | 0.98837 | 0.90957 | 0.92771 | 1 | 0.974244 |
| 425 | 0.96154 | 1 | 1 | 0.99725 | 0.89873 | 1 | 1 | 0.98298 | 0.9322 | 0.95 | 1 | 0.98403 | 0.96753 | 0.88679 | 1 | 0.98837 | 0.91489 | 0.92771 | 1 | 0.975364 |
| 426 | 0.96154 | 1 | 1 | 0.99725 | 0.89873 | 1 | 1 | 0.98298 | 0.9322 | 0.95 | 1 | 0.98403 | 0.96753 | 0.88679 | 1 | 0.98837 | 0.91489 | 0.92771 | 1 | 0.975364 |
| 427 | 0.96154 | 1 | 1 | 0.99725 | 0.89873 | 1 | 1 | 0.98298 | 0.9322 | 0.95 | 1 | 0.98403 | 0.96753 | 0.88679 | 1 | 0.98837 | 0.91489 | 0.92771 | 1 | 0.975364 |
| 428 | 0.96154 | 1 | 1 | 0.99725 | 0.89873 | 1 | 1 | 0.98298 | 0.9322 | 0.95 | 1 | 0.98403 | 0.96753 | 0.88679 | 1 | 0.98837 | 0.91489 | 0.92771 | 1 | 0.975364 |
| 429 | 0.96154 | 1 | 1 | 0.99725 | 0.89873 | 1 | 1 | 0.98298 | 0.9322 | 0.95 | 1 | 0.98403 | 0.96753 | 0.88679 | 1 | 0.98837 | 0.91489 | 0.92771 | 1 | 0.975364 |
| 430 | 0.96154 | 1 | 1 | 0.99725 | 0.89873 | 1 | 1 | 0.98298 | 0.9322 | 0.95 | 1 | 0.98403 | 0.96753 | 0.88679 | 1 | 0.98837 | 0.91489 | 0.92771 | 1 | 0.975364 |
| 431 | 0.96154 | 1 | 1 | 0.99725 | 0.89873 | 1 | 1 | 0.98298 | 0.9322 | 0.95 | 1 | 0.98403 | 0.96753 | 0.88679 | 1 | 0.98837 | 0.90957 | 0.91566 | 1 | 0.973124 |
| 432 | 0.96154 | 1 | 1 | 0.99725 | 0.89873 | 1 | 1 | 0.98298 | 0.9322 | 0.95 | 1 | 0.98403 | 0.96753 | 0.88679 | 1 | 0.98837 | 0.90957 | 0.91566 | 1 | 0.973124 |
| 433 | 0.96154 | 1 | 1 | 0.99725 | 0.89873 | 1 | 1 | 0.98298 | 0.9322 | 0.95 | 1 | 0.98403 | 0.96753 | 0.88679 | 1 | 0.98837 | 0.90957 | 0.91566 | 1 | 0.973124 |
| 434 | 0.96154 | 1 | 1 | 0.99725 | 0.89873 | 1 | 1 | 0.98298 | 0.9322 | 0.95 | 1 | 0.98403 | 0.96753 | 0.88679 | 1 | 0.98837 | 0.90957 | 0.91566 | 1 | 0.973124 |
| 435 | 0.96154 | 1 | 1 | 0.99725 | 0.89873 | 1 | 1 | 0.98298 | 0.9322 | 0.95 | 1 | 0.98403 | 0.96753 | 0.88679 | 1 | 0.98837 | 0.90957 | 0.91566 | 1 | 0.973124 |
| 436 | 0.96154 | 1 | 1 | 0.99725 | 0.89873 | 1 | 1 | 0.98298 | 0.9322 | 0.95 | 1 | 0.98403 | 0.96753 | 0.88679 | 1 | 0.98837 | 0.90957 | 0.91566 | 1 | 0.973124 |
| 437 | 0.96154 | 1 | 1 | 0.99725 | 0.89873 | 1 | 1 | 0.98298 | 0.9322 | 0.95 | 1 | 0.98403 | 0.96753 | 0.88679 | 1 | 0.98837 | 0.90957 | 0.91566 | 1 | 0.973124 |
| 438 | 0.96154 | 1 | 1 | 0.99725 | 0.89873 | 1 | 1 | 0.98298 | 0.9322 | 0.95 | 1 | 0.98403 | 0.96753 | 0.88679 | 1 | 0.98837 | 0.91489 | 0.91566 | 1 | 0.974244 |
| 439 | 0.96154 | 1 | 1 | 0.99725 | 0.89873 | 1 | 1 | 0.98298 | 0.9322 | 0.95 | 1 | 0.98403 | 0.96753 | 0.88679 | 1 | 0.98837 | 0.92021 | 0.91566 | 1 | 0.975364 |
| 440 | 0.96154 | 1 | 1 | 0.99725 | 0.89873 | 1 | 1 | 0.98298 | 0.9322 | 0.95 | 1 | 0.98403 | 0.96753 | 0.88679 | 1 | 0.98837 | 0.92021 | 0.91566 | 1 | 0.975364 |
| 441 | 0.96154 | 1 | 1 | 0.99725 | 0.89873 | 1 | 1 | 0.98298 | 0.9322 | 0.95 | 1 | 0.98403 | 0.96753 | 0.88679 | 1 | 0.98837 | 0.90957 | 0.91566 | 1 | 0.973124 |
| 442 | 0.96154 | 1 | 1 | 0.99725 | 0.88608 | 1 | 1 | 0.98085 | 0.9322 | 0.95 | 1 | 0.98403 | 0.96753 | 0.88679 | 1 | 0.98837 | 0.92021 | 0.91566 | 1 | 0.975364 |
| 443 | 0.96154 | 1 | 1 | 0.99725 | 0.88608 | 1 | 1 | 0.98085 | 0.9322 | 0.95 | 1 | 0.98403 | 0.96753 | 0.88679 | 1 | 0.98837 | 0.92021 | 0.91566 | 1 | 0.975364 |
| 444 | 0.96154 | 1 | 1 | 0.99725 | 0.88608 | 1 | 1 | 0.98085 | 0.9322 | 0.95 | 1 | 0.98403 | 0.96753 | 0.88679 | 0.99865 | 0.98732 | 0.93085 | 0.91566 | 1 | 0.977604 |
| 445 | 0.96154 | 1 | 1 | 0.99725 | 0.88608 | 1 | 1 | 0.98085 | 0.9322 | 0.95 | 1 | 0.98403 | 0.96753 | 0.88679 | 0.99865 | 0.98732 | 0.93617 | 0.91566 | 1 | 0.978723 |
| 446 | 0.96154 | 1 | 1 | 0.99725 | 0.88608 | 1 | 1 | 0.98085 | 0.9322 | 0.95 | 1 | 0.98403 | 0.96753 | 0.88679 | 0.99865 | 0.98732 | 0.93085 | 0.91566 | 1 | 0.977604 |
| 447 | 0.96154 | 1 | 1 | 0.99725 | 0.87342 | 1 | 1 | 0.97872 | 0.9322 | 0.95 | 1 | 0.98403 | 0.96753 | 0.88679 | 0.99865 | 0.98732 | 0.93085 | 0.91566 | 1 | 0.977604 |
| 448 | 0.96154 | 1 | 1 | 0.99725 | 0.87342 | 1 | 1 | 0.97872 | 0.9322 | 0.95 | 1 | 0.98403 | 0.96753 | 0.88679 | 0.99865 | 0.98732 | 0.93085 | 0.91566 | 1 | 0.977604 |
| 449 | 0.96154 | 1 | 1 | 0.99725 | 0.87342 | 1 | 1 | 0.97872 | 0.9322 | 0.95 | 1 | 0.98403 | 0.96753 | 0.88679 | 0.99865 | 0.98732 | 0.93085 | 0.91566 | 1 | 0.977604 |
| 450 | 0.96154 | 1 | 1 | 0.99725 | 0.88608 | 1 | 1 | 0.98085 | 0.9322 | 0.95 | 1 | 0.98403 | 0.96753 | 0.88679 | 1 | 0.98837 | 0.92021 | 0.91566 | 1 | 0.975364 |
| 451 | 0.96154 | 1 | 1 | 0.99725 | 0.88608 | 1 | 1 | 0.98085 | 0.9322 | 0.95 | 1 | 0.98403 | 0.96753 | 0.88679 | 1 | 0.98837 | 0.92021 | 0.91566 | 1 | 0.975364 |
| 452 | 0.96154 | 1 | 1 | 0.99725 | 0.88608 | 1 | 1 | 0.98085 | 0.9322 | 0.95 | 1 | 0.98403 | 0.96753 | 0.88679 | 1 | 0.98837 | 0.91489 | 0.91566 | 1 | 0.974244 |
| 453 | 0.96154 | 1 | 1 | 0.99725 | 0.88608 | 1 | 1 | 0.98085 | 0.9322 | 0.95 | 1 | 0.98403 | 0.96753 | 0.88679 | 1 | 0.98837 | 0.91489 | 0.91566 | 1 | 0.974244 |
| 454 | 0.96154 | 1 | 1 | 0.99725 | 0.88608 | 1 | 1 | 0.98085 | 0.9322 | 0.95 | 1 | 0.98403 | 0.96753 | 0.88679 | 1 | 0.98837 | 0.91489 | 0.91566 | 1 | 0.974244 |
| 455 | 0.96154 | 1 | 1 | 0.99725 | 0.88608 | 1 | 1 | 0.98085 | 0.9322 | 0.95 | 1 | 0.98403 | 0.96753 | 0.88679 | 1 | 0.98837 | 0.91489 | 0.91566 | 1 | 0.974244 |
| 456 | 0.96154 | 1 | 1 | 0.99725 | 0.88608 | 1 | 1 | 0.98085 | 0.9322 | 0.95 | 1 | 0.98403 | 0.96753 | 0.88679 | 1 | 0.98837 | 0.91489 | 0.91566 | 1 | 0.974244 |
| 457 | 0.96154 | 1 | 1 | 0.99725 | 0.88608 | 1 | 1 | 0.98085 | 0.9322 | 0.95 | 1 | 0.98403 | 0.96753 | 0.88679 | 1 | 0.98837 | 0.91489 | 0.91566 | 1 | 0.974244 |
| 458 | 0.96154 | 1 | 1 | 0.99725 | 0.87342 | 1 | 1 | 0.97872 | 0.9322 | 0.95 | 1 | 0.98403 | 0.96753 | 0.88679 | 1 | 0.98837 | 0.90957 | 0.91566 | 1 | 0.973124 |
| 459 | 0.96154 | 1 | 1 | 0.99725 | 0.87342 | 1 | 1 | 0.97872 | 0.9322 | 0.95 | 1 | 0.98403 | 0.96753 | 0.88679 | 1 | 0.98837 | 0.90957 | 0.91566 | 1 | 0.973124 |
| 460 | 0.96154 | 1 | 1 | 0.99725 | 0.87342 | 1 | 1 | 0.97872 | 0.9322 | 0.95 | 1 | 0.98403 | 0.96753 | 0.88679 | 1 | 0.98837 | 0.92553 | 0.91566 | 1 | 0.976484 |
| 461 | 0.96154 | 1 | 1 | 0.99725 | 0.87342 | 1 | 1 | 0.97872 | 0.9322 | 0.95 | 1 | 0.98403 | 0.96753 | 0.88679 | 1 | 0.98837 | 0.93085 | 0.91566 | 1 | 0.977604 |
| 462 | 0.96154 | 1 | 1 | 0.99725 | 0.87342 | 1 | 1 | 0.97872 | 0.9322 | 0.95 | 1 | 0.98403 | 0.96753 | 0.88679 | 1 | 0.98837 | 0.92553 | 0.91566 | 1 | 0.976484 |
| 463 | 0.96154 | 1 | 1 | 0.99725 | 0.87342 | 1 | 1 | 0.97872 | 0.9322 | 0.95 | 1 | 0.98403 | 0.96753 | 0.88679 | 1 | 0.98837 | 0.90957 | 0.91566 | 1 | 0.973124 |
| 464 | 0.96154 | 1 | 1 | 0.99725 | 0.87342 | 1 | 1 | 0.97872 | 0.9322 | 0.95 | 1 | 0.98403 | 0.96753 | 0.88679 | 1 | 0.98837 | 0.90957 | 0.91566 | 1 | 0.973124 |
| 465 | 0.96154 | 1 | 1 | 0.99725 | 0.87342 | 1 | 1 | 0.97872 | 0.9322 | 0.95 | 1 | 0.98403 | 0.96753 | 0.88679 | 1 | 0.98837 | 0.90957 | 0.91566 | 1 | 0.973124 |
| 466 | 0.96154 | 1 | 1 | 0.99725 | 0.87342 | 1 | 1 | 0.97872 | 0.9322 | 0.95 | 1 | 0.98403 | 0.96753 | 0.88679 | 1 | 0.98837 | 0.90426 | 0.91566 | 1 | 0.972004 |
| 467 | 0.96154 | 1 | 1 | 0.99725 | 0.87342 | 1 | 1 | 0.97872 | 0.9322 | 0.95 | 1 | 0.98403 | 0.96753 | 0.88679 | 1 | 0.98837 | 0.90426 | 0.91566 | 1 | 0.972004 |
| 468 | 0.96154 | 1 | 1 | 0.99725 | 0.86076 | 1 | 1 | 0.9766 | 0.9322 | 0.95 | 1 | 0.98403 | 0.96753 | 0.88679 | 1 | 0.98837 | 0.90426 | 0.91566 | 1 | 0.972004 |
| 469 | 0.96154 | 1 | 1 | 0.99725 | 0.86076 | 1 | 1 | 0.9766 | 0.9322 | 0.95 | 1 | 0.98403 | 0.96753 | 0.88679 | 1 | 0.98837 | 0.90426 | 0.91566 | 1 | 0.972004 |
| 470 | 0.96154 | 1 | 1 | 0.99725 | 0.86076 | 1 | 1 | 0.9766 | 0.9322 | 0.95 | 1 | 0.98403 | 0.96753 | 0.88679 | 1 | 0.98837 | 0.90426 | 0.91566 | 1 | 0.972004 |
| 471 | 0.96154 | 1 | 1 | 0.99725 | 0.86076 | 1 | 1 | 0.9766 | 0.9322 | 0.95 | 1 | 0.98403 | 0.96753 | 0.88679 | 1 | 0.98837 | 0.90426 | 0.91566 | 1 | 0.972004 |
| 472 | 0.96154 | 1 | 1 | 0.99725 | 0.86076 | 1 | 1 | 0.9766 | 0.9322 | 0.95 | 1 | 0.98403 | 0.96753 | 0.88679 | 1 | 0.98837 | 0.89894 | 0.91566 | 1 | 0.970885 |
| 473 | 0.96154 | 1 | 1 | 0.99725 | 0.86076 | 1 | 1 | 0.9766 | 0.9322 | 0.95 | 1 | 0.98403 | 0.96753 | 0.88679 | 1 | 0.98837 | 0.89894 | 0.91566 | 1 | 0.970885 |
| 474 | 0.96154 | 1 | 1 | 0.99725 | 0.86076 | 1 | 1 | 0.9766 | 0.9322 | 0.95 | 1 | 0.98403 | 0.96753 | 0.88679 | 1 | 0.98837 | 0.89894 | 0.91566 | 1 | 0.970885 |
| 475 | 0.96154 | 1 | 1 | 0.99725 | 0.86076 | 1 | 1 | 0.9766 | 0.9322 | 0.95 | 1 | 0.98403 | 0.96753 | 0.88679 | 1 | 0.98837 | 0.89894 | 0.91566 | 1 | 0.970885 |
| 476 | 0.96154 | 1 | 1 | 0.99725 | 0.86076 | 1 | 1 | 0.9766 | 0.9322 | 0.95 | 1 | 0.98403 | 0.96753 | 0.88679 | 1 | 0.98837 | 0.89894 | 0.91566 | 1 | 0.970885 |
| 477 | 0.96154 | 1 | 1 | 0.99725 | 0.86076 | 1 | 1 | 0.9766 | 0.9322 | 0.95 | 1 | 0.98403 | 0.96753 | 0.88679 | 1 | 0.98837 | 0.89894 | 0.91566 | 1 | 0.970885 |
| 478 | 0.96154 | 1 | 1 | 0.99725 | 0.86076 | 1 | 1 | 0.9766 | 0.9322 | 0.95 | 1 | 0.98403 | 0.96753 | 0.88679 | 1 | 0.98837 | 0.89894 | 0.92771 | 1 | 0.972004 |
| 479 | 0.96154 | 1 | 1 | 0.99725 | 0.86076 | 1 | 1 | 0.9766 | 0.9322 | 0.95 | 1 | 0.98403 | 0.96753 | 0.88679 | 1 | 0.98837 | 0.89894 | 0.92771 | 1 | 0.972004 |
| 480 | 0.96154 | 1 | 1 | 0.99725 | 0.86076 | 1 | 1 | 0.9766 | 0.9322 | 0.95 | 1 | 0.98403 | 0.96753 | 0.88679 | 1 | 0.98837 | 0.89894 | 0.92771 | 1 | 0.972004 |
| 481 | 0.96154 | 1 | 1 | 0.99725 | 0.86076 | 1 | 1 | 0.9766 | 0.9322 | 0.95 | 1 | 0.98403 | 0.96753 | 0.88679 | 1 | 0.98837 | 0.89894 | 0.92771 | 1 | 0.972004 |
| 482 | 0.96154 | 1 | 1 | 0.99725 | 0.86076 | 1 | 1 | 0.9766 | 0.9322 | 0.95 | 1 | 0.98403 | 0.96753 | 0.88679 | 1 | 0.98837 | 0.89894 | 0.92771 | 1 | 0.972004 |
| 483 | 0.96154 | 1 | 1 | 0.99725 | 0.86076 | 1 | 1 | 0.9766 | 0.9322 | 0.95 | 1 | 0.98403 | 0.96104 | 0.88679 | 1 | 0.98732 | 0.89894 | 0.92771 | 1 | 0.972004 |
| 484 | 0.96154 | 1 | 1 | 0.99725 | 0.86076 | 1 | 1 | 0.9766 | 0.9322 | 0.95 | 1 | 0.98403 | 0.96104 | 0.88679 | 1 | 0.98732 | 0.89894 | 0.92771 | 1 | 0.972004 |
| 485 | 0.96154 | 1 | 1 | 0.99725 | 0.86076 | 1 | 1 | 0.9766 | 0.9322 | 0.95 | 1 | 0.98403 | 0.96104 | 0.88679 | 1 | 0.98732 | 0.89894 | 0.92771 | 1 | 0.972004 |
| 486 | 0.96154 | 1 | 1 | 0.99725 | 0.86076 | 1 | 1 | 0.9766 | 0.9322 | 0.95 | 1 | 0.98403 | 0.96104 | 0.88679 | 1 | 0.98732 | 0.89894 | 0.92771 | 1 | 0.972004 |
| 487 | 0.96154 | 1 | 1 | 0.99725 | 0.86076 | 1 | 1 | 0.9766 | 0.9322 | 0.95 | 1 | 0.98403 | 0.96104 | 0.88679 | 1 | 0.98732 | 0.89894 | 0.92771 | 1 | 0.972004 |
| 488 | 0.96154 | 1 | 1 | 0.99725 | 0.86076 | 1 | 1 | 0.9766 | 0.9322 | 0.95 | 1 | 0.98403 | 0.96104 | 0.88679 | 1 | 0.98732 | 0.89894 | 0.92771 | 1 | 0.972004 |
| 489 | 0.96154 | 1 | 1 | 0.99725 | 0.86076 | 1 | 1 | 0.9766 | 0.9322 | 0.95 | 1 | 0.98403 | 0.96104 | 0.88679 | 1 | 0.98732 | 0.89894 | 0.92771 | 1 | 0.972004 |
| 490 | 0.96154 | 1 | 1 | 0.99725 | 0.86076 | 1 | 1 | 0.9766 | 0.9322 | 0.95 | 1 | 0.98403 | 0.96104 | 0.88679 | 1 | 0.98732 | 0.89894 | 0.92771 | 1 | 0.972004 |
| 491 | 0.96154 | 1 | 1 | 0.99725 | 0.86076 | 1 | 1 | 0.9766 | 0.9322 | 0.95 | 1 | 0.98403 | 0.96753 | 0.88679 | 1 | 0.98837 | 0.89894 | 0.92771 | 1 | 0.972004 |
| 492 | 0.96154 | 1 | 1 | 0.99725 | 0.86076 | 1 | 1 | 0.9766 | 0.9322 | 0.95 | 1 | 0.98403 | 0.96104 | 0.88679 | 1 | 0.98732 | 0.89894 | 0.92771 | 1 | 0.972004 |
| 493 | 0.96154 | 1 | 1 | 0.99725 | 0.86076 | 1 | 1 | 0.9766 | 0.9322 | 0.95 | 1 | 0.98403 | 0.96104 | 0.88679 | 1 | 0.98732 | 0.89894 | 0.92771 | 1 | 0.972004 |
| 494 | 0.96154 | 1 | 1 | 0.99725 | 0.86076 | 1 | 1 | 0.9766 | 0.9322 | 0.95 | 1 | 0.98403 | 0.96104 | 0.88679 | 1 | 0.98732 | 0.89894 | 0.92771 | 1 | 0.972004 |
| 495 | 0.96154 | 1 | 1 | 0.99725 | 0.86076 | 1 | 1 | 0.9766 | 0.9322 | 0.95 | 1 | 0.98403 | 0.96104 | 0.88679 | 1 | 0.98732 | 0.89894 | 0.92771 | 1 | 0.972004 |
| 496 | 0.96154 | 1 | 1 | 0.99725 | 0.86076 | 1 | 1 | 0.9766 | 0.9322 | 0.95 | 1 | 0.98403 | 0.96753 | 0.88679 | 1 | 0.98837 | 0.90426 | 0.92771 | 1 | 0.973124 |
| 497 | 0.96154 | 1 | 1 | 0.99725 | 0.86076 | 1 | 1 | 0.9766 | 0.9322 | 0.95 | 1 | 0.98403 | 0.96753 | 0.88679 | 1 | 0.98837 | 0.90426 | 0.92771 | 1 | 0.973124 |
| 498 | 0.96154 | 1 | 1 | 0.99725 | 0.86076 | 1 | 1 | 0.9766 | 0.9322 | 0.95 | 1 | 0.98403 | 0.96753 | 0.88679 | 1 | 0.98837 | 0.90426 | 0.92771 | 1 | 0.973124 |
| 499 | 0.96154 | 1 | 1 | 0.99725 | 0.86076 | 1 | 1 | 0.9766 | 0.9322 | 0.95 | 1 | 0.98403 | 0.96753 | 0.88679 | 1 | 0.98837 | 0.89894 | 0.92771 | 1 | 0.972004 |
| 500 | 0.96154 | 1 | 1 | 0.99725 | 0.86076 | 1 | 1 | 0.9766 | 0.9322 | 0.95 | 1 | 0.98403 | 0.96753 | 0.88679 | 1 | 0.98837 | 0.90426 | 0.92771 | 1 | 0.973124 |

1. The dataset of H0351.1015 was selected as the training dataset

| Number of features | H0351.1009 | | | | H0351.1012 | | | | H0351.1016 | | | | H0351.2001 | | | | H0351.2002 | | | |
| --- | --- | --- | --- | --- | --- | --- | --- | --- | --- | --- | --- | --- | --- | --- | --- | --- | --- | --- | --- | --- |
|  | class1 | class2 | class3 | total | class1 | class2 | class3 | total | class1 | class2 | class3 | total | class1 | class2 | class3 | total | class1 | class2 | class3 | total |
| 4 | 1 | 0.952 | 0.949 | 0.953 | 0.925 | 0.917 | 0.97 | 0.958 | 0.983 | 0.95 | 0.994 | 0.986 | 0.916 | 0.774 | 0.968 | 0.948 | 0.606 | 0.916 | 0.992 | 0.9037 |
| 5 | 1 | 0.952 | 0.942 | 0.948 | 0.988 | 0.917 | 0.965 | 0.964 | 1 | 0.95 | 0.992 | 0.986 | 0.955 | 0.774 | 0.957 | 0.946 | 0.729 | 0.916 | 0.992 | 0.9295 |
| 6 | 1 | 0.952 | 0.922 | 0.931 | 0.963 | 0.917 | 0.975 | 0.968 | 0.983 | 0.95 | 0.992 | 0.984 | 0.955 | 0.774 | 0.949 | 0.94 | 0.564 | 0.916 | 0.995 | 0.897 |
| 7 | 1 | 0.952 | 0.939 | 0.945 | 1 | 0.917 | 0.97 | 0.97 | 1 | 0.95 | 0.994 | 0.988 | 0.968 | 0.774 | 0.965 | 0.955 | 0.697 | 0.916 | 0.997 | 0.9261 |
| 8 | 1 | 0.952 | 0.983 | 0.981 | 1 | 0.917 | 0.993 | 0.987 | 1 | 0.95 | 1 | 0.992 | 0.942 | 0.774 | 0.986 | 0.967 | 0.814 | 0.916 | 1 | 0.953 |
| 9 | 1 | 0.952 | 0.976 | 0.975 | 1 | 0.917 | 0.99 | 0.985 | 1 | 0.95 | 1 | 0.992 | 0.968 | 0.774 | 0.981 | 0.967 | 0.856 | 0.916 | 1 | 0.9619 |
| 10 | 1 | 0.952 | 0.976 | 0.975 | 1 | 0.917 | 0.99 | 0.985 | 1 | 0.95 | 1 | 0.992 | 0.961 | 0.774 | 0.981 | 0.966 | 0.856 | 0.916 | 1 | 0.9619 |
| 11 | 1 | 0.952 | 0.976 | 0.975 | 1 | 0.917 | 0.993 | 0.987 | 1 | 0.95 | 1 | 0.992 | 0.961 | 0.774 | 0.978 | 0.964 | 0.84 | 0.916 | 1 | 0.9586 |
| 12 | 1 | 0.952 | 0.969 | 0.97 | 1 | 0.917 | 0.988 | 0.983 | 1 | 0.95 | 1 | 0.992 | 0.974 | 0.774 | 0.978 | 0.966 | 0.872 | 0.916 | 0.998 | 0.9642 |
| 13 | 1 | 0.952 | 0.966 | 0.967 | 1 | 0.917 | 0.985 | 0.981 | 1 | 0.95 | 1 | 0.992 | 0.974 | 0.774 | 0.977 | 0.965 | 0.872 | 0.916 | 0.998 | 0.9642 |
| 14 | 1 | 0.952 | 0.966 | 0.967 | 1 | 0.917 | 0.988 | 0.983 | 1 | 0.95 | 1 | 0.992 | 0.974 | 0.774 | 0.977 | 0.965 | 0.867 | 0.916 | 0.998 | 0.963 |
| 15 | 1 | 0.952 | 0.963 | 0.964 | 1 | 0.917 | 0.988 | 0.983 | 1 | 0.95 | 1 | 0.992 | 0.974 | 0.774 | 0.98 | 0.967 | 0.851 | 0.916 | 0.998 | 0.9597 |
| 16 | 1 | 0.952 | 0.963 | 0.964 | 1 | 0.917 | 0.988 | 0.983 | 1 | 0.95 | 1 | 0.992 | 0.974 | 0.774 | 0.98 | 0.967 | 0.851 | 0.916 | 0.998 | 0.9597 |
| 17 | 1 | 0.952 | 0.973 | 0.972 | 1 | 0.917 | 0.983 | 0.979 | 1 | 0.95 | 1 | 0.992 | 0.968 | 0.774 | 0.974 | 0.962 | 0.862 | 0.916 | 0.998 | 0.9619 |
| 18 | 1 | 0.952 | 0.973 | 0.972 | 1 | 0.917 | 0.985 | 0.981 | 1 | 0.95 | 1 | 0.992 | 0.974 | 0.774 | 0.977 | 0.965 | 0.883 | 0.916 | 0.998 | 0.9664 |
| 19 | 1 | 0.952 | 0.973 | 0.972 | 1 | 0.917 | 0.985 | 0.981 | 1 | 0.95 | 1 | 0.992 | 0.968 | 0.774 | 0.974 | 0.962 | 0.872 | 0.916 | 0.998 | 0.9642 |
| 20 | 1 | 0.952 | 0.959 | 0.961 | 1 | 0.917 | 0.98 | 0.977 | 1 | 0.95 | 0.997 | 0.99 | 0.987 | 0.774 | 0.962 | 0.956 | 0.888 | 0.916 | 0.998 | 0.9675 |
| 21 | 1 | 0.952 | 0.976 | 0.975 | 1 | 0.917 | 0.988 | 0.983 | 1 | 0.95 | 1 | 0.992 | 0.974 | 0.774 | 0.969 | 0.959 | 0.883 | 0.916 | 1 | 0.9675 |
| 22 | 1 | 0.952 | 0.969 | 0.97 | 1 | 0.917 | 0.988 | 0.983 | 1 | 0.95 | 1 | 0.992 | 0.974 | 0.774 | 0.968 | 0.958 | 0.872 | 0.916 | 1 | 0.9653 |
| 23 | 1 | 0.952 | 0.973 | 0.972 | 1 | 0.917 | 0.988 | 0.983 | 1 | 0.95 | 1 | 0.992 | 0.974 | 0.774 | 0.968 | 0.958 | 0.883 | 0.916 | 1 | 0.9675 |
| 24 | 1 | 0.952 | 0.963 | 0.964 | 1 | 0.917 | 0.985 | 0.981 | 1 | 0.95 | 1 | 0.992 | 0.981 | 0.774 | 0.962 | 0.955 | 0.894 | 0.916 | 1 | 0.9698 |
| 25 | 1 | 0.952 | 0.963 | 0.964 | 1 | 0.917 | 0.985 | 0.981 | 1 | 0.95 | 1 | 0.992 | 0.981 | 0.774 | 0.961 | 0.953 | 0.888 | 0.916 | 1 | 0.9686 |
| 26 | 1 | 0.952 | 0.969 | 0.97 | 1 | 0.917 | 0.988 | 0.983 | 1 | 0.95 | 1 | 0.992 | 0.981 | 0.774 | 0.962 | 0.955 | 0.872 | 0.916 | 1 | 0.9653 |
| 27 | 1 | 0.952 | 0.969 | 0.97 | 1 | 0.917 | 0.985 | 0.981 | 1 | 0.95 | 1 | 0.992 | 0.974 | 0.774 | 0.962 | 0.953 | 0.883 | 0.916 | 1 | 0.9675 |
| 28 | 1 | 0.952 | 0.973 | 0.972 | 1 | 0.917 | 0.988 | 0.983 | 1 | 0.95 | 1 | 0.992 | 0.968 | 0.774 | 0.961 | 0.951 | 0.894 | 0.916 | 1 | 0.9698 |
| 29 | 1 | 0.952 | 0.969 | 0.97 | 1 | 0.917 | 0.983 | 0.979 | 1 | 0.95 | 1 | 0.992 | 0.968 | 0.774 | 0.961 | 0.951 | 0.894 | 0.916 | 1 | 0.9698 |
| 30 | 1 | 0.952 | 0.966 | 0.967 | 1 | 0.917 | 0.983 | 0.979 | 1 | 0.95 | 0.997 | 0.99 | 0.968 | 0.774 | 0.959 | 0.95 | 0.894 | 0.916 | 1 | 0.9698 |
| 31 | 1 | 0.952 | 0.963 | 0.964 | 1 | 0.917 | 0.98 | 0.977 | 1 | 0.95 | 0.997 | 0.99 | 0.968 | 0.774 | 0.959 | 0.95 | 0.899 | 0.916 | 1 | 0.9709 |
| 32 | 1 | 0.952 | 0.963 | 0.964 | 1 | 0.917 | 0.98 | 0.977 | 1 | 0.95 | 0.997 | 0.99 | 0.968 | 0.774 | 0.959 | 0.95 | 0.894 | 0.916 | 1 | 0.9698 |
| 33 | 1 | 0.952 | 0.966 | 0.967 | 1 | 0.917 | 0.98 | 0.977 | 1 | 0.95 | 0.997 | 0.99 | 0.968 | 0.774 | 0.959 | 0.95 | 0.894 | 0.916 | 1 | 0.9698 |
| 34 | 1 | 0.952 | 0.966 | 0.967 | 1 | 0.917 | 0.98 | 0.977 | 1 | 0.95 | 0.997 | 0.99 | 0.968 | 0.774 | 0.959 | 0.95 | 0.894 | 1 | 1 | 0.9776 |
| 35 | 1 | 0.952 | 0.966 | 0.967 | 1 | 0.917 | 0.983 | 0.979 | 1 | 0.95 | 0.997 | 0.99 | 0.968 | 0.774 | 0.962 | 0.952 | 0.888 | 1 | 1 | 0.9765 |
| 36 | 1 | 0.952 | 0.966 | 0.967 | 1 | 0.917 | 0.983 | 0.979 | 1 | 0.95 | 0.997 | 0.99 | 0.968 | 0.774 | 0.962 | 0.952 | 0.888 | 1 | 1 | 0.9765 |
| 37 | 1 | 0.952 | 0.966 | 0.967 | 1 | 0.917 | 0.983 | 0.979 | 1 | 0.95 | 0.997 | 0.99 | 0.968 | 0.774 | 0.962 | 0.952 | 0.888 | 1 | 1 | 0.9765 |
| 38 | 1 | 0.952 | 0.963 | 0.964 | 1 | 0.917 | 0.983 | 0.979 | 1 | 0.95 | 0.997 | 0.99 | 0.968 | 0.774 | 0.962 | 0.952 | 0.888 | 1 | 1 | 0.9765 |
| 39 | 1 | 0.952 | 0.963 | 0.964 | 1 | 0.917 | 0.983 | 0.979 | 1 | 0.95 | 0.997 | 0.99 | 0.968 | 0.774 | 0.961 | 0.951 | 0.888 | 1 | 0.998 | 0.9754 |
| 40 | 1 | 0.952 | 0.963 | 0.964 | 1 | 0.917 | 0.983 | 0.979 | 1 | 0.95 | 0.997 | 0.99 | 0.968 | 0.774 | 0.961 | 0.951 | 0.888 | 1 | 0.998 | 0.9754 |
| 41 | 1 | 0.952 | 0.969 | 0.97 | 1 | 0.938 | 0.985 | 0.983 | 0.983 | 0.95 | 1 | 0.99 | 0.968 | 0.774 | 0.963 | 0.953 | 0.899 | 1 | 0.997 | 0.9765 |
| 42 | 1 | 0.952 | 0.969 | 0.97 | 1 | 0.917 | 0.985 | 0.981 | 1 | 0.95 | 1 | 0.992 | 0.961 | 0.774 | 0.963 | 0.952 | 0.894 | 0.976 | 0.997 | 0.9731 |
| 43 | 1 | 0.952 | 0.969 | 0.97 | 1 | 0.917 | 0.985 | 0.981 | 1 | 0.95 | 1 | 0.992 | 0.968 | 0.774 | 0.963 | 0.953 | 0.899 | 0.988 | 0.998 | 0.9765 |
| 44 | 1 | 0.952 | 0.969 | 0.97 | 1 | 0.938 | 0.983 | 0.981 | 0.983 | 0.95 | 1 | 0.99 | 0.961 | 0.792 | 0.966 | 0.956 | 0.867 | 1 | 1 | 0.972 |
| 45 | 1 | 0.952 | 0.969 | 0.97 | 1 | 0.958 | 0.985 | 0.985 | 0.983 | 0.95 | 1 | 0.99 | 0.942 | 0.849 | 0.969 | 0.958 | 0.846 | 1 | 1 | 0.9675 |
| 46 | 1 | 0.952 | 0.966 | 0.967 | 1 | 0.958 | 0.983 | 0.983 | 0.983 | 0.95 | 1 | 0.99 | 0.942 | 0.83 | 0.968 | 0.956 | 0.851 | 1 | 1 | 0.9686 |
| 47 | 1 | 0.952 | 0.969 | 0.97 | 1 | 0.938 | 0.983 | 0.981 | 0.983 | 0.95 | 1 | 0.99 | 0.942 | 0.83 | 0.968 | 0.956 | 0.835 | 1 | 1 | 0.9653 |
| 48 | 1 | 0.952 | 0.966 | 0.967 | 1 | 0.938 | 0.983 | 0.981 | 0.983 | 0.95 | 1 | 0.99 | 0.942 | 0.83 | 0.968 | 0.956 | 0.835 | 1 | 1 | 0.9653 |
| 49 | 1 | 0.952 | 0.966 | 0.967 | 1 | 0.938 | 0.983 | 0.981 | 0.966 | 0.95 | 1 | 0.988 | 0.942 | 0.83 | 0.968 | 0.956 | 0.835 | 1 | 1 | 0.9653 |
| 50 | 1 | 0.952 | 0.966 | 0.967 | 1 | 0.938 | 0.985 | 0.983 | 0.966 | 0.95 | 1 | 0.988 | 0.942 | 0.83 | 0.968 | 0.956 | 0.835 | 1 | 1 | 0.9653 |
| 51 | 1 | 0.952 | 0.959 | 0.961 | 0.988 | 0.938 | 0.983 | 0.979 | 0.966 | 0.95 | 1 | 0.988 | 0.942 | 0.83 | 0.966 | 0.955 | 0.798 | 1 | 1 | 0.9574 |
| 52 | 1 | 0.952 | 0.959 | 0.961 | 0.988 | 0.938 | 0.983 | 0.979 | 0.966 | 0.95 | 1 | 0.988 | 0.942 | 0.83 | 0.966 | 0.955 | 0.787 | 1 | 1 | 0.9552 |
| 53 | 1 | 0.952 | 0.959 | 0.961 | 0.988 | 0.958 | 0.98 | 0.979 | 0.966 | 0.963 | 1 | 0.99 | 0.948 | 0.849 | 0.965 | 0.956 | 0.798 | 1 | 0.998 | 0.9563 |
| 54 | 1 | 0.952 | 0.959 | 0.961 | 0.988 | 0.958 | 0.98 | 0.979 | 0.966 | 0.963 | 1 | 0.99 | 0.948 | 0.849 | 0.965 | 0.956 | 0.798 | 1 | 0.998 | 0.9563 |
| 55 | 1 | 0.952 | 0.959 | 0.961 | 0.988 | 0.958 | 0.978 | 0.977 | 0.966 | 0.963 | 1 | 0.99 | 0.955 | 0.849 | 0.965 | 0.957 | 0.798 | 1 | 0.998 | 0.9563 |
| 56 | 1 | 0.952 | 0.959 | 0.961 | 0.988 | 0.958 | 0.985 | 0.983 | 0.966 | 0.963 | 1 | 0.99 | 0.955 | 0.849 | 0.965 | 0.957 | 0.819 | 1 | 0.998 | 0.9608 |
| 57 | 1 | 0.952 | 0.959 | 0.961 | 0.988 | 0.958 | 0.985 | 0.983 | 0.966 | 0.963 | 1 | 0.99 | 0.955 | 0.849 | 0.965 | 0.957 | 0.819 | 1 | 0.998 | 0.9608 |
| 58 | 1 | 0.952 | 0.946 | 0.95 | 0.988 | 0.958 | 0.98 | 0.979 | 0.966 | 0.963 | 1 | 0.99 | 0.961 | 0.868 | 0.962 | 0.957 | 0.819 | 1 | 0.998 | 0.9608 |
| 59 | 1 | 0.952 | 0.942 | 0.948 | 0.988 | 0.958 | 0.978 | 0.977 | 0.966 | 0.963 | 1 | 0.99 | 0.955 | 0.868 | 0.962 | 0.956 | 0.793 | 1 | 1 | 0.9563 |
| 60 | 1 | 0.952 | 0.942 | 0.948 | 0.988 | 0.958 | 0.978 | 0.977 | 0.966 | 0.963 | 1 | 0.99 | 0.955 | 0.868 | 0.962 | 0.956 | 0.793 | 1 | 1 | 0.9563 |
| 61 | 1 | 0.952 | 0.946 | 0.95 | 0.988 | 0.958 | 0.978 | 0.977 | 0.966 | 0.963 | 1 | 0.99 | 0.955 | 0.868 | 0.962 | 0.956 | 0.798 | 1 | 1 | 0.9574 |
| 62 | 1 | 0.952 | 0.946 | 0.95 | 0.988 | 0.958 | 0.98 | 0.979 | 0.966 | 0.963 | 1 | 0.99 | 0.955 | 0.868 | 0.962 | 0.956 | 0.819 | 1 | 0.998 | 0.9608 |
| 63 | 1 | 0.952 | 0.946 | 0.95 | 0.988 | 0.958 | 0.98 | 0.979 | 0.966 | 0.963 | 1 | 0.99 | 0.955 | 0.868 | 0.962 | 0.956 | 0.819 | 1 | 0.998 | 0.9608 |
| 64 | 1 | 0.952 | 0.946 | 0.95 | 0.988 | 0.958 | 0.98 | 0.979 | 0.966 | 0.963 | 1 | 0.99 | 0.955 | 0.868 | 0.963 | 0.957 | 0.819 | 1 | 0.998 | 0.9608 |
| 65 | 1 | 0.952 | 0.946 | 0.95 | 0.988 | 0.958 | 0.98 | 0.979 | 0.966 | 0.963 | 1 | 0.99 | 0.948 | 0.868 | 0.963 | 0.956 | 0.819 | 1 | 0.997 | 0.9597 |
| 66 | 1 | 0.952 | 0.946 | 0.95 | 0.988 | 0.958 | 0.98 | 0.979 | 0.966 | 0.963 | 1 | 0.99 | 0.948 | 0.868 | 0.963 | 0.956 | 0.819 | 1 | 0.997 | 0.9597 |
| 67 | 1 | 0.952 | 0.946 | 0.95 | 0.988 | 0.958 | 0.978 | 0.977 | 0.966 | 0.963 | 1 | 0.99 | 0.948 | 0.868 | 0.962 | 0.955 | 0.819 | 1 | 0.997 | 0.9597 |
| 68 | 1 | 0.952 | 0.946 | 0.95 | 0.988 | 0.958 | 0.978 | 0.977 | 0.966 | 0.963 | 1 | 0.99 | 0.948 | 0.868 | 0.963 | 0.956 | 0.803 | 1 | 0.997 | 0.9563 |
| 69 | 1 | 0.952 | 0.946 | 0.95 | 0.988 | 0.958 | 0.98 | 0.979 | 0.966 | 0.963 | 1 | 0.99 | 0.948 | 0.868 | 0.963 | 0.956 | 0.814 | 1 | 0.997 | 0.9586 |
| 70 | 1 | 0.952 | 0.953 | 0.956 | 0.988 | 0.958 | 0.98 | 0.979 | 0.966 | 0.963 | 1 | 0.99 | 0.942 | 0.868 | 0.965 | 0.956 | 0.819 | 1 | 0.997 | 0.9597 |
| 71 | 1 | 0.952 | 0.953 | 0.956 | 1 | 0.958 | 0.978 | 0.979 | 0.966 | 0.963 | 1 | 0.99 | 0.942 | 0.868 | 0.963 | 0.955 | 0.824 | 1 | 0.998 | 0.9619 |
| 72 | 1 | 0.952 | 0.953 | 0.956 | 1 | 0.958 | 0.975 | 0.977 | 0.966 | 0.95 | 1 | 0.988 | 0.948 | 0.868 | 0.959 | 0.952 | 0.824 | 1 | 0.997 | 0.9608 |
| 73 | 1 | 0.952 | 0.949 | 0.953 | 0.988 | 0.958 | 0.975 | 0.975 | 0.966 | 0.95 | 1 | 0.988 | 0.961 | 0.868 | 0.959 | 0.955 | 0.846 | 1 | 0.997 | 0.9653 |
| 74 | 1 | 0.952 | 0.949 | 0.953 | 1 | 0.958 | 0.975 | 0.977 | 0.966 | 0.95 | 1 | 0.988 | 0.961 | 0.868 | 0.959 | 0.955 | 0.846 | 1 | 0.997 | 0.9653 |
| 75 | 1 | 0.952 | 0.949 | 0.953 | 1 | 0.958 | 0.975 | 0.977 | 0.966 | 0.963 | 1 | 0.99 | 0.961 | 0.887 | 0.959 | 0.956 | 0.846 | 1 | 0.997 | 0.9653 |
| 76 | 1 | 0.952 | 0.949 | 0.953 | 0.988 | 0.958 | 0.975 | 0.975 | 0.966 | 0.95 | 1 | 0.988 | 0.955 | 0.849 | 0.962 | 0.955 | 0.83 | 1 | 0.997 | 0.9619 |
| 77 | 1 | 0.952 | 0.949 | 0.953 | 0.988 | 0.958 | 0.975 | 0.975 | 0.966 | 0.95 | 1 | 0.988 | 0.955 | 0.849 | 0.959 | 0.952 | 0.824 | 1 | 0.998 | 0.9619 |
| 78 | 1 | 0.952 | 0.932 | 0.939 | 1 | 0.958 | 0.973 | 0.975 | 0.983 | 0.95 | 0.997 | 0.988 | 0.968 | 0.83 | 0.959 | 0.953 | 0.856 | 1 | 0.997 | 0.9675 |
| 79 | 1 | 0.952 | 0.946 | 0.95 | 1 | 0.958 | 0.975 | 0.977 | 0.983 | 0.95 | 0.997 | 0.988 | 0.968 | 0.83 | 0.961 | 0.955 | 0.856 | 1 | 0.997 | 0.9675 |
| 80 | 1 | 0.952 | 0.939 | 0.945 | 1 | 0.958 | 0.975 | 0.977 | 0.983 | 0.95 | 0.997 | 0.988 | 0.961 | 0.849 | 0.961 | 0.955 | 0.835 | 1 | 0.997 | 0.963 |
| 81 | 1 | 0.952 | 0.922 | 0.931 | 1 | 0.958 | 0.975 | 0.977 | 0.983 | 0.95 | 0.994 | 0.986 | 0.968 | 0.849 | 0.957 | 0.952 | 0.851 | 1 | 0.997 | 0.9664 |
| 82 | 1 | 0.952 | 0.922 | 0.931 | 1 | 0.958 | 0.975 | 0.977 | 0.983 | 0.95 | 0.992 | 0.984 | 0.968 | 0.849 | 0.957 | 0.952 | 0.846 | 1 | 0.997 | 0.9653 |
| 83 | 1 | 0.952 | 0.922 | 0.931 | 1 | 0.958 | 0.975 | 0.977 | 0.983 | 0.95 | 0.992 | 0.984 | 0.968 | 0.849 | 0.957 | 0.952 | 0.84 | 1 | 0.997 | 0.9642 |
| 84 | 1 | 0.952 | 0.922 | 0.931 | 1 | 0.958 | 0.975 | 0.977 | 0.983 | 0.95 | 0.992 | 0.984 | 0.968 | 0.849 | 0.955 | 0.951 | 0.856 | 1 | 0.997 | 0.9675 |
| 85 | 1 | 0.952 | 0.919 | 0.928 | 1 | 0.958 | 0.973 | 0.975 | 0.983 | 0.95 | 0.992 | 0.984 | 0.968 | 0.849 | 0.955 | 0.951 | 0.846 | 1 | 0.997 | 0.9653 |
| 86 | 1 | 0.952 | 0.919 | 0.928 | 1 | 0.958 | 0.973 | 0.975 | 0.983 | 0.95 | 0.992 | 0.984 | 0.968 | 0.849 | 0.953 | 0.949 | 0.846 | 1 | 0.997 | 0.9653 |
| 87 | 1 | 0.952 | 0.919 | 0.928 | 1 | 0.958 | 0.973 | 0.975 | 0.983 | 0.95 | 0.992 | 0.984 | 0.968 | 0.849 | 0.951 | 0.948 | 0.851 | 1 | 0.997 | 0.9664 |
| 88 | 1 | 0.952 | 0.912 | 0.923 | 1 | 0.958 | 0.97 | 0.974 | 1 | 0.95 | 0.992 | 0.986 | 0.974 | 0.83 | 0.94 | 0.94 | 0.867 | 1 | 0.997 | 0.9698 |
| 89 | 1 | 0.952 | 0.912 | 0.923 | 1 | 0.958 | 0.97 | 0.974 | 1 | 0.95 | 0.992 | 0.986 | 0.974 | 0.849 | 0.94 | 0.941 | 0.872 | 1 | 0.997 | 0.9709 |
| 90 | 1 | 0.952 | 0.892 | 0.906 | 1 | 0.958 | 0.97 | 0.974 | 1 | 0.95 | 0.981 | 0.978 | 0.974 | 0.849 | 0.919 | 0.924 | 0.91 | 1 | 0.997 | 0.9787 |
| 91 | 1 | 0.952 | 0.892 | 0.906 | 1 | 0.958 | 0.97 | 0.974 | 1 | 0.95 | 0.981 | 0.978 | 0.974 | 0.849 | 0.917 | 0.923 | 0.904 | 1 | 0.997 | 0.9776 |
| 92 | 1 | 0.952 | 0.892 | 0.906 | 1 | 0.958 | 0.97 | 0.974 | 1 | 0.95 | 0.981 | 0.978 | 0.974 | 0.849 | 0.917 | 0.923 | 0.904 | 1 | 0.997 | 0.9776 |
| 93 | 1 | 0.952 | 0.895 | 0.909 | 1 | 0.958 | 0.968 | 0.972 | 1 | 0.95 | 0.983 | 0.98 | 0.974 | 0.849 | 0.917 | 0.923 | 0.947 | 1 | 0.995 | 0.9854 |
| 94 | 1 | 0.952 | 0.895 | 0.909 | 1 | 0.958 | 0.968 | 0.972 | 1 | 0.95 | 0.983 | 0.98 | 0.974 | 0.849 | 0.919 | 0.924 | 0.947 | 1 | 0.995 | 0.9854 |
| 95 | 1 | 0.952 | 0.895 | 0.909 | 1 | 0.958 | 0.97 | 0.974 | 1 | 0.95 | 0.986 | 0.982 | 0.974 | 0.849 | 0.919 | 0.924 | 0.947 | 1 | 0.995 | 0.9854 |
| 96 | 1 | 0.952 | 0.902 | 0.915 | 1 | 0.958 | 0.968 | 0.972 | 1 | 0.95 | 0.983 | 0.98 | 0.974 | 0.849 | 0.926 | 0.929 | 0.926 | 1 | 0.997 | 0.9821 |
| 97 | 1 | 0.952 | 0.902 | 0.915 | 1 | 0.958 | 0.968 | 0.972 | 1 | 0.95 | 0.983 | 0.98 | 0.974 | 0.849 | 0.926 | 0.929 | 0.936 | 1 | 0.997 | 0.9843 |
| 98 | 1 | 0.952 | 0.898 | 0.912 | 1 | 0.958 | 0.968 | 0.972 | 1 | 0.95 | 0.983 | 0.98 | 0.974 | 0.849 | 0.928 | 0.931 | 0.915 | 1 | 0.997 | 0.9798 |
| 99 | 1 | 0.952 | 0.898 | 0.912 | 1 | 0.958 | 0.968 | 0.972 | 1 | 0.95 | 0.983 | 0.98 | 0.974 | 0.849 | 0.928 | 0.931 | 0.915 | 1 | 0.997 | 0.9798 |
| 100 | 1 | 0.952 | 0.898 | 0.912 | 1 | 0.958 | 0.968 | 0.972 | 1 | 0.95 | 0.983 | 0.98 | 0.974 | 0.849 | 0.928 | 0.931 | 0.915 | 1 | 0.997 | 0.9798 |
| 101 | 1 | 0.952 | 0.898 | 0.912 | 1 | 0.958 | 0.968 | 0.972 | 1 | 0.95 | 0.983 | 0.98 | 0.974 | 0.83 | 0.928 | 0.93 | 0.915 | 1 | 0.997 | 0.9798 |
| 102 | 1 | 0.952 | 0.895 | 0.909 | 1 | 0.958 | 0.968 | 0.972 | 1 | 0.95 | 0.983 | 0.98 | 0.974 | 0.83 | 0.928 | 0.93 | 0.915 | 1 | 0.997 | 0.9798 |
| 103 | 1 | 0.952 | 0.895 | 0.909 | 1 | 0.958 | 0.968 | 0.972 | 1 | 0.95 | 0.983 | 0.98 | 0.974 | 0.83 | 0.928 | 0.93 | 0.915 | 1 | 0.997 | 0.9798 |
| 104 | 1 | 0.952 | 0.895 | 0.909 | 1 | 0.958 | 0.97 | 0.974 | 1 | 0.95 | 0.983 | 0.98 | 0.974 | 0.83 | 0.93 | 0.931 | 0.926 | 1 | 0.997 | 0.9821 |
| 105 | 1 | 0.952 | 0.895 | 0.909 | 1 | 0.958 | 0.97 | 0.974 | 1 | 0.95 | 0.983 | 0.98 | 0.974 | 0.83 | 0.927 | 0.929 | 0.92 | 1 | 0.997 | 0.981 |
| 106 | 1 | 0.952 | 0.895 | 0.909 | 1 | 0.958 | 0.97 | 0.974 | 1 | 0.95 | 0.983 | 0.98 | 0.974 | 0.83 | 0.927 | 0.929 | 0.92 | 1 | 0.997 | 0.981 |
| 107 | 1 | 0.952 | 0.908 | 0.92 | 1 | 0.938 | 0.973 | 0.974 | 1 | 0.95 | 0.986 | 0.982 | 0.974 | 0.792 | 0.928 | 0.928 | 0.936 | 0.94 | 0.997 | 0.9787 |
| 108 | 1 | 0.952 | 0.905 | 0.917 | 1 | 0.938 | 0.973 | 0.974 | 1 | 0.95 | 0.989 | 0.984 | 0.974 | 0.792 | 0.928 | 0.928 | 0.936 | 0.94 | 0.997 | 0.9787 |
| 109 | 1 | 0.952 | 0.912 | 0.923 | 1 | 0.938 | 0.973 | 0.974 | 1 | 0.95 | 0.983 | 0.98 | 0.974 | 0.792 | 0.938 | 0.936 | 0.91 | 0.94 | 0.997 | 0.9731 |
| 110 | 1 | 0.952 | 0.912 | 0.923 | 1 | 0.938 | 0.973 | 0.974 | 1 | 0.95 | 0.983 | 0.98 | 0.974 | 0.83 | 0.938 | 0.938 | 0.91 | 0.94 | 0.997 | 0.9731 |
| 111 | 1 | 0.952 | 0.905 | 0.917 | 1 | 0.938 | 0.973 | 0.974 | 1 | 0.95 | 0.986 | 0.982 | 0.974 | 0.792 | 0.938 | 0.936 | 0.91 | 0.94 | 0.997 | 0.9731 |
| 112 | 1 | 0.952 | 0.908 | 0.92 | 1 | 0.938 | 0.973 | 0.974 | 1 | 0.95 | 0.986 | 0.982 | 0.974 | 0.792 | 0.938 | 0.936 | 0.91 | 0.94 | 0.997 | 0.9731 |
| 113 | 1 | 0.952 | 0.908 | 0.92 | 1 | 0.938 | 0.973 | 0.974 | 1 | 0.95 | 0.986 | 0.982 | 0.974 | 0.792 | 0.938 | 0.936 | 0.91 | 0.94 | 0.997 | 0.9731 |
| 114 | 1 | 0.952 | 0.908 | 0.92 | 1 | 0.938 | 0.973 | 0.974 | 1 | 0.95 | 0.986 | 0.982 | 0.974 | 0.811 | 0.938 | 0.937 | 0.91 | 0.94 | 0.997 | 0.9731 |
| 115 | 1 | 0.952 | 0.908 | 0.92 | 1 | 0.938 | 0.973 | 0.974 | 1 | 0.95 | 0.986 | 0.982 | 0.974 | 0.811 | 0.938 | 0.937 | 0.915 | 0.94 | 0.997 | 0.9742 |
| 116 | 1 | 0.952 | 0.905 | 0.917 | 1 | 0.938 | 0.973 | 0.974 | 1 | 0.95 | 0.983 | 0.98 | 0.974 | 0.811 | 0.938 | 0.937 | 0.915 | 0.94 | 0.997 | 0.9742 |
| 117 | 1 | 0.952 | 0.905 | 0.917 | 1 | 0.938 | 0.973 | 0.974 | 1 | 0.95 | 0.983 | 0.98 | 0.974 | 0.792 | 0.938 | 0.936 | 0.915 | 0.928 | 0.997 | 0.9731 |
| 118 | 1 | 0.952 | 0.908 | 0.92 | 1 | 0.958 | 0.973 | 0.975 | 1 | 0.95 | 0.983 | 0.98 | 0.974 | 0.792 | 0.938 | 0.936 | 0.92 | 0.928 | 0.997 | 0.9742 |
| 119 | 1 | 0.952 | 0.905 | 0.917 | 1 | 0.958 | 0.973 | 0.975 | 1 | 0.95 | 0.983 | 0.98 | 0.974 | 0.792 | 0.938 | 0.936 | 0.915 | 0.928 | 0.997 | 0.9731 |
| 120 | 1 | 0.952 | 0.885 | 0.901 | 1 | 0.938 | 0.97 | 0.972 | 1 | 0.95 | 0.983 | 0.98 | 0.974 | 0.774 | 0.911 | 0.913 | 0.957 | 0.916 | 0.995 | 0.9798 |
| 121 | 1 | 0.952 | 0.885 | 0.901 | 1 | 0.938 | 0.97 | 0.972 | 1 | 0.95 | 0.983 | 0.98 | 0.974 | 0.774 | 0.911 | 0.913 | 0.957 | 0.916 | 0.995 | 0.9798 |
| 122 | 1 | 0.952 | 0.895 | 0.909 | 1 | 0.938 | 0.97 | 0.972 | 1 | 0.95 | 0.983 | 0.98 | 0.974 | 0.774 | 0.912 | 0.914 | 0.957 | 0.916 | 0.995 | 0.9798 |
| 123 | 1 | 0.952 | 0.895 | 0.909 | 1 | 0.938 | 0.97 | 0.972 | 1 | 0.95 | 0.983 | 0.98 | 0.974 | 0.774 | 0.912 | 0.914 | 0.957 | 0.916 | 0.995 | 0.9798 |
| 124 | 1 | 0.952 | 0.895 | 0.909 | 1 | 0.938 | 0.97 | 0.972 | 1 | 0.95 | 0.983 | 0.98 | 0.974 | 0.774 | 0.912 | 0.914 | 0.957 | 0.928 | 0.995 | 0.981 |
| 125 | 1 | 0.952 | 0.905 | 0.917 | 1 | 0.938 | 0.973 | 0.974 | 1 | 0.95 | 0.983 | 0.98 | 0.974 | 0.792 | 0.915 | 0.918 | 0.952 | 0.928 | 0.998 | 0.9821 |
| 126 | 1 | 0.952 | 0.905 | 0.917 | 1 | 0.938 | 0.97 | 0.972 | 1 | 0.95 | 0.983 | 0.98 | 0.974 | 0.774 | 0.915 | 0.916 | 0.957 | 0.916 | 0.998 | 0.9821 |
| 127 | 1 | 0.952 | 0.908 | 0.92 | 1 | 0.938 | 0.973 | 0.974 | 1 | 0.95 | 0.983 | 0.98 | 0.974 | 0.774 | 0.915 | 0.916 | 0.957 | 0.916 | 0.998 | 0.9821 |
| 128 | 1 | 0.952 | 0.908 | 0.92 | 1 | 0.938 | 0.973 | 0.974 | 1 | 0.95 | 0.983 | 0.98 | 0.974 | 0.774 | 0.915 | 0.916 | 0.957 | 0.916 | 0.998 | 0.9821 |
| 129 | 1 | 0.952 | 0.908 | 0.92 | 1 | 0.938 | 0.973 | 0.974 | 1 | 0.95 | 0.983 | 0.98 | 0.974 | 0.774 | 0.915 | 0.916 | 0.957 | 0.916 | 0.998 | 0.9821 |
| 130 | 1 | 0.952 | 0.908 | 0.92 | 1 | 0.938 | 0.973 | 0.974 | 1 | 0.95 | 0.981 | 0.978 | 0.974 | 0.774 | 0.916 | 0.918 | 0.957 | 0.916 | 0.998 | 0.9821 |
| 131 | 1 | 0.952 | 0.908 | 0.92 | 1 | 0.938 | 0.973 | 0.974 | 1 | 0.95 | 0.986 | 0.982 | 0.974 | 0.774 | 0.917 | 0.919 | 0.957 | 0.916 | 0.998 | 0.9821 |
| 132 | 1 | 0.952 | 0.905 | 0.917 | 1 | 0.938 | 0.973 | 0.974 | 1 | 0.95 | 0.986 | 0.982 | 0.974 | 0.774 | 0.919 | 0.92 | 0.957 | 0.916 | 0.998 | 0.9821 |
| 133 | 1 | 0.952 | 0.888 | 0.904 | 1 | 0.958 | 0.97 | 0.974 | 1 | 0.95 | 0.981 | 0.978 | 0.974 | 0.792 | 0.909 | 0.913 | 0.963 | 0.916 | 0.997 | 0.9821 |
| 134 | 1 | 0.952 | 0.888 | 0.904 | 1 | 0.958 | 0.97 | 0.974 | 1 | 0.95 | 0.981 | 0.978 | 0.974 | 0.774 | 0.911 | 0.913 | 0.963 | 0.916 | 0.997 | 0.9821 |
| 135 | 1 | 0.952 | 0.888 | 0.904 | 1 | 0.958 | 0.968 | 0.972 | 1 | 0.95 | 0.978 | 0.976 | 0.974 | 0.792 | 0.909 | 0.913 | 0.963 | 0.928 | 0.998 | 0.9843 |
| 136 | 1 | 0.952 | 0.888 | 0.904 | 1 | 0.958 | 0.968 | 0.972 | 1 | 0.95 | 0.978 | 0.976 | 0.974 | 0.792 | 0.909 | 0.913 | 0.963 | 0.928 | 0.998 | 0.9843 |
| 137 | 1 | 0.952 | 0.888 | 0.904 | 1 | 0.958 | 0.968 | 0.972 | 1 | 0.95 | 0.981 | 0.978 | 0.974 | 0.774 | 0.907 | 0.91 | 0.963 | 0.916 | 0.998 | 0.9832 |
| 138 | 1 | 0.952 | 0.895 | 0.909 | 1 | 0.958 | 0.97 | 0.974 | 1 | 0.95 | 0.978 | 0.976 | 0.974 | 0.774 | 0.908 | 0.911 | 0.952 | 0.916 | 0.998 | 0.981 |
| 139 | 1 | 0.952 | 0.905 | 0.917 | 1 | 0.958 | 0.97 | 0.974 | 1 | 0.95 | 0.981 | 0.978 | 0.974 | 0.774 | 0.912 | 0.914 | 0.936 | 0.916 | 0.998 | 0.9776 |
| 140 | 1 | 0.952 | 0.905 | 0.917 | 1 | 0.958 | 0.97 | 0.974 | 1 | 0.95 | 0.981 | 0.978 | 0.974 | 0.774 | 0.916 | 0.918 | 0.936 | 0.916 | 0.998 | 0.9776 |
| 141 | 1 | 0.952 | 0.905 | 0.917 | 1 | 0.958 | 0.97 | 0.974 | 1 | 0.95 | 0.981 | 0.978 | 0.974 | 0.774 | 0.916 | 0.918 | 0.936 | 0.916 | 0.998 | 0.9776 |
| 142 | 1 | 0.952 | 0.905 | 0.917 | 1 | 0.958 | 0.973 | 0.975 | 1 | 0.95 | 0.981 | 0.978 | 0.974 | 0.774 | 0.916 | 0.918 | 0.936 | 0.928 | 0.998 | 0.9787 |
| 143 | 1 | 0.952 | 0.905 | 0.917 | 1 | 0.958 | 0.973 | 0.975 | 1 | 0.95 | 0.981 | 0.978 | 0.974 | 0.774 | 0.916 | 0.918 | 0.936 | 0.928 | 0.998 | 0.9787 |
| 144 | 1 | 0.952 | 0.905 | 0.917 | 1 | 0.958 | 0.973 | 0.975 | 1 | 0.95 | 0.981 | 0.978 | 0.974 | 0.774 | 0.916 | 0.918 | 0.936 | 0.928 | 0.998 | 0.9787 |
| 145 | 1 | 0.952 | 0.905 | 0.917 | 1 | 0.958 | 0.973 | 0.975 | 1 | 0.95 | 0.981 | 0.978 | 0.974 | 0.774 | 0.915 | 0.916 | 0.936 | 0.928 | 0.998 | 0.9787 |
| 146 | 1 | 0.952 | 0.905 | 0.917 | 1 | 0.958 | 0.973 | 0.975 | 1 | 0.95 | 0.981 | 0.978 | 0.974 | 0.774 | 0.916 | 0.918 | 0.931 | 0.928 | 0.998 | 0.9776 |
| 147 | 1 | 0.952 | 0.905 | 0.917 | 1 | 0.958 | 0.973 | 0.975 | 1 | 0.95 | 0.981 | 0.978 | 0.974 | 0.774 | 0.916 | 0.918 | 0.931 | 0.928 | 0.998 | 0.9776 |
| 148 | 1 | 0.952 | 0.902 | 0.915 | 1 | 0.958 | 0.973 | 0.975 | 1 | 0.95 | 0.981 | 0.978 | 0.974 | 0.774 | 0.915 | 0.916 | 0.92 | 0.928 | 0.998 | 0.9754 |
| 149 | 1 | 0.952 | 0.898 | 0.912 | 1 | 0.958 | 0.973 | 0.975 | 1 | 0.95 | 0.981 | 0.978 | 0.974 | 0.774 | 0.915 | 0.916 | 0.92 | 0.928 | 0.998 | 0.9754 |
| 150 | 1 | 0.952 | 0.902 | 0.915 | 1 | 0.958 | 0.973 | 0.975 | 1 | 0.95 | 0.981 | 0.978 | 0.974 | 0.774 | 0.915 | 0.916 | 0.92 | 0.928 | 0.998 | 0.9754 |
| 151 | 1 | 0.952 | 0.908 | 0.92 | 1 | 0.958 | 0.973 | 0.975 | 1 | 0.95 | 0.981 | 0.978 | 0.974 | 0.774 | 0.916 | 0.918 | 0.92 | 0.928 | 0.998 | 0.9754 |
| 152 | 1 | 0.952 | 0.908 | 0.92 | 1 | 0.958 | 0.973 | 0.975 | 1 | 0.95 | 0.981 | 0.978 | 0.974 | 0.774 | 0.916 | 0.918 | 0.92 | 0.928 | 0.998 | 0.9754 |
| 153 | 1 | 0.952 | 0.908 | 0.92 | 1 | 0.958 | 0.97 | 0.974 | 1 | 0.95 | 0.983 | 0.98 | 0.974 | 0.774 | 0.919 | 0.92 | 0.936 | 0.928 | 0.998 | 0.9787 |
| 154 | 1 | 0.952 | 0.908 | 0.92 | 1 | 0.958 | 0.97 | 0.974 | 1 | 0.95 | 0.983 | 0.98 | 0.974 | 0.774 | 0.919 | 0.92 | 0.936 | 0.94 | 0.998 | 0.9798 |
| 155 | 1 | 0.952 | 0.908 | 0.92 | 1 | 0.958 | 0.97 | 0.974 | 1 | 0.95 | 0.986 | 0.982 | 0.974 | 0.774 | 0.922 | 0.922 | 0.936 | 0.94 | 0.998 | 0.9798 |
| 156 | 1 | 0.952 | 0.908 | 0.92 | 1 | 0.958 | 0.97 | 0.974 | 1 | 0.95 | 0.983 | 0.98 | 0.974 | 0.774 | 0.923 | 0.923 | 0.936 | 0.928 | 0.998 | 0.9787 |
| 157 | 1 | 0.952 | 0.908 | 0.92 | 1 | 0.958 | 0.97 | 0.974 | 1 | 0.95 | 0.986 | 0.982 | 0.974 | 0.774 | 0.924 | 0.924 | 0.936 | 0.94 | 0.998 | 0.9798 |
| 158 | 1 | 0.952 | 0.908 | 0.92 | 1 | 0.958 | 0.97 | 0.974 | 1 | 0.95 | 0.986 | 0.982 | 0.974 | 0.774 | 0.924 | 0.924 | 0.936 | 0.94 | 0.998 | 0.9798 |
| 159 | 1 | 0.952 | 0.908 | 0.92 | 1 | 0.958 | 0.97 | 0.974 | 1 | 0.95 | 0.986 | 0.982 | 0.974 | 0.774 | 0.924 | 0.924 | 0.936 | 0.94 | 0.998 | 0.9798 |
| 160 | 1 | 0.952 | 0.908 | 0.92 | 1 | 0.958 | 0.97 | 0.974 | 1 | 0.95 | 0.986 | 0.982 | 0.974 | 0.774 | 0.924 | 0.924 | 0.936 | 0.94 | 0.998 | 0.9798 |
| 161 | 1 | 0.952 | 0.908 | 0.92 | 1 | 0.958 | 0.97 | 0.974 | 1 | 0.95 | 0.986 | 0.982 | 0.974 | 0.774 | 0.924 | 0.924 | 0.936 | 0.94 | 0.998 | 0.9798 |
| 162 | 1 | 0.952 | 0.908 | 0.92 | 1 | 0.958 | 0.97 | 0.974 | 1 | 0.95 | 0.983 | 0.98 | 0.974 | 0.774 | 0.923 | 0.923 | 0.936 | 0.94 | 0.998 | 0.9798 |
| 163 | 1 | 0.952 | 0.908 | 0.92 | 1 | 0.958 | 0.97 | 0.974 | 1 | 0.95 | 0.983 | 0.98 | 0.974 | 0.774 | 0.923 | 0.923 | 0.936 | 0.94 | 0.998 | 0.9798 |
| 164 | 1 | 0.952 | 0.908 | 0.92 | 1 | 0.958 | 0.973 | 0.975 | 1 | 0.95 | 0.983 | 0.98 | 0.974 | 0.774 | 0.923 | 0.923 | 0.931 | 0.94 | 0.998 | 0.9787 |
| 165 | 1 | 0.952 | 0.908 | 0.92 | 1 | 0.958 | 0.973 | 0.975 | 1 | 0.95 | 0.983 | 0.98 | 0.974 | 0.774 | 0.923 | 0.923 | 0.931 | 0.94 | 0.998 | 0.9787 |
| 166 | 1 | 0.952 | 0.908 | 0.92 | 1 | 0.958 | 0.97 | 0.974 | 1 | 0.95 | 0.983 | 0.98 | 0.974 | 0.774 | 0.923 | 0.923 | 0.931 | 0.94 | 0.998 | 0.9787 |
| 167 | 1 | 0.952 | 0.908 | 0.92 | 1 | 0.958 | 0.97 | 0.974 | 1 | 0.95 | 0.983 | 0.98 | 0.974 | 0.774 | 0.923 | 0.923 | 0.931 | 0.928 | 0.998 | 0.9776 |
| 168 | 1 | 0.952 | 0.908 | 0.92 | 1 | 0.958 | 0.97 | 0.974 | 1 | 0.95 | 0.983 | 0.98 | 0.974 | 0.774 | 0.932 | 0.93 | 0.931 | 0.928 | 0.998 | 0.9776 |
| 169 | 1 | 0.952 | 0.908 | 0.92 | 1 | 0.958 | 0.97 | 0.974 | 1 | 0.95 | 0.983 | 0.98 | 0.974 | 0.774 | 0.93 | 0.928 | 0.931 | 0.928 | 0.998 | 0.9776 |
| 170 | 1 | 0.952 | 0.908 | 0.92 | 1 | 0.958 | 0.97 | 0.974 | 1 | 0.95 | 0.983 | 0.98 | 0.974 | 0.774 | 0.931 | 0.929 | 0.931 | 0.928 | 0.998 | 0.9776 |
| 171 | 1 | 0.952 | 0.912 | 0.923 | 1 | 0.958 | 0.97 | 0.974 | 1 | 0.95 | 0.983 | 0.98 | 0.974 | 0.774 | 0.932 | 0.93 | 0.931 | 0.928 | 0.998 | 0.9776 |
| 172 | 1 | 0.952 | 0.912 | 0.923 | 1 | 0.958 | 0.97 | 0.974 | 1 | 0.95 | 0.983 | 0.98 | 0.974 | 0.774 | 0.932 | 0.93 | 0.931 | 0.928 | 0.998 | 0.9776 |
| 173 | 1 | 0.952 | 0.908 | 0.92 | 1 | 0.958 | 0.973 | 0.975 | 1 | 0.95 | 0.983 | 0.98 | 0.974 | 0.774 | 0.93 | 0.928 | 0.931 | 0.928 | 0.998 | 0.9776 |
| 174 | 1 | 0.952 | 0.908 | 0.92 | 1 | 0.958 | 0.973 | 0.975 | 1 | 0.95 | 0.983 | 0.98 | 0.974 | 0.774 | 0.928 | 0.927 | 0.931 | 0.928 | 0.998 | 0.9776 |
| 175 | 1 | 0.952 | 0.908 | 0.92 | 1 | 0.958 | 0.973 | 0.975 | 1 | 0.95 | 0.983 | 0.98 | 0.974 | 0.774 | 0.928 | 0.927 | 0.931 | 0.928 | 0.998 | 0.9776 |
| 176 | 1 | 0.952 | 0.905 | 0.917 | 1 | 0.958 | 0.973 | 0.975 | 1 | 0.95 | 0.983 | 0.98 | 0.974 | 0.774 | 0.934 | 0.931 | 0.926 | 0.928 | 0.998 | 0.9765 |
| 177 | 1 | 0.952 | 0.905 | 0.917 | 1 | 0.958 | 0.973 | 0.975 | 1 | 0.95 | 0.983 | 0.98 | 0.974 | 0.774 | 0.934 | 0.931 | 0.926 | 0.928 | 0.998 | 0.9765 |
| 178 | 1 | 0.952 | 0.905 | 0.917 | 1 | 0.958 | 0.973 | 0.975 | 1 | 0.95 | 0.981 | 0.978 | 0.974 | 0.774 | 0.922 | 0.922 | 0.931 | 0.928 | 0.998 | 0.9776 |
| 179 | 1 | 0.952 | 0.905 | 0.917 | 1 | 0.958 | 0.973 | 0.975 | 1 | 0.95 | 0.981 | 0.978 | 0.974 | 0.774 | 0.922 | 0.922 | 0.931 | 0.928 | 0.998 | 0.9776 |
| 180 | 1 | 0.952 | 0.905 | 0.917 | 1 | 0.958 | 0.973 | 0.975 | 1 | 0.95 | 0.981 | 0.978 | 0.974 | 0.774 | 0.924 | 0.924 | 0.936 | 0.928 | 0.998 | 0.9787 |
| 181 | 1 | 0.952 | 0.905 | 0.917 | 1 | 0.958 | 0.973 | 0.975 | 1 | 0.95 | 0.981 | 0.978 | 0.974 | 0.774 | 0.926 | 0.925 | 0.936 | 0.928 | 0.998 | 0.9787 |
| 182 | 1 | 0.952 | 0.905 | 0.917 | 1 | 0.958 | 0.973 | 0.975 | 1 | 0.95 | 0.981 | 0.978 | 0.974 | 0.774 | 0.92 | 0.921 | 0.936 | 0.928 | 0.998 | 0.9787 |
| 183 | 1 | 0.952 | 0.905 | 0.917 | 1 | 0.958 | 0.973 | 0.975 | 1 | 0.95 | 0.981 | 0.978 | 0.974 | 0.774 | 0.92 | 0.921 | 0.936 | 0.928 | 0.998 | 0.9787 |
| 184 | 1 | 0.952 | 0.905 | 0.917 | 1 | 0.958 | 0.973 | 0.975 | 1 | 0.95 | 0.983 | 0.98 | 0.974 | 0.774 | 0.927 | 0.926 | 0.936 | 0.928 | 0.998 | 0.9787 |
| 185 | 1 | 0.952 | 0.905 | 0.917 | 1 | 0.958 | 0.973 | 0.975 | 1 | 0.95 | 0.983 | 0.98 | 0.974 | 0.774 | 0.926 | 0.925 | 0.936 | 0.928 | 0.998 | 0.9787 |
| 186 | 1 | 0.952 | 0.905 | 0.917 | 1 | 0.958 | 0.973 | 0.975 | 1 | 0.95 | 0.983 | 0.98 | 0.974 | 0.774 | 0.924 | 0.924 | 0.936 | 0.928 | 0.998 | 0.9787 |
| 187 | 1 | 0.952 | 0.905 | 0.917 | 1 | 0.958 | 0.973 | 0.975 | 1 | 0.95 | 0.983 | 0.98 | 0.974 | 0.774 | 0.922 | 0.922 | 0.936 | 0.928 | 0.998 | 0.9787 |
| 188 | 1 | 0.952 | 0.905 | 0.917 | 1 | 0.958 | 0.973 | 0.975 | 1 | 0.95 | 0.983 | 0.98 | 0.974 | 0.774 | 0.92 | 0.921 | 0.936 | 0.928 | 0.998 | 0.9787 |
| 189 | 1 | 0.952 | 0.905 | 0.917 | 1 | 0.958 | 0.973 | 0.975 | 1 | 0.95 | 0.983 | 0.98 | 0.974 | 0.774 | 0.919 | 0.92 | 0.936 | 0.928 | 0.998 | 0.9787 |
| 190 | 1 | 0.952 | 0.898 | 0.912 | 1 | 0.958 | 0.973 | 0.975 | 1 | 0.95 | 0.983 | 0.98 | 0.981 | 0.774 | 0.908 | 0.912 | 0.952 | 0.928 | 0.998 | 0.9821 |
| 191 | 1 | 0.952 | 0.898 | 0.912 | 1 | 0.958 | 0.973 | 0.975 | 1 | 0.95 | 0.983 | 0.98 | 0.974 | 0.774 | 0.907 | 0.91 | 0.947 | 0.928 | 0.998 | 0.981 |
| 192 | 1 | 0.952 | 0.898 | 0.912 | 1 | 0.958 | 0.973 | 0.975 | 1 | 0.95 | 0.983 | 0.98 | 0.974 | 0.774 | 0.907 | 0.91 | 0.947 | 0.928 | 0.998 | 0.981 |
| 193 | 1 | 0.952 | 0.898 | 0.912 | 1 | 0.958 | 0.97 | 0.974 | 1 | 0.95 | 0.983 | 0.98 | 0.981 | 0.774 | 0.905 | 0.91 | 0.941 | 0.928 | 0.998 | 0.9798 |
| 194 | 1 | 0.952 | 0.898 | 0.912 | 1 | 0.958 | 0.97 | 0.974 | 1 | 0.95 | 0.983 | 0.98 | 0.981 | 0.774 | 0.905 | 0.91 | 0.941 | 0.94 | 0.998 | 0.981 |
| 195 | 1 | 0.952 | 0.898 | 0.912 | 1 | 0.958 | 0.973 | 0.975 | 1 | 0.95 | 0.983 | 0.98 | 0.974 | 0.774 | 0.907 | 0.91 | 0.941 | 0.94 | 0.998 | 0.981 |
| 196 | 1 | 0.952 | 0.898 | 0.912 | 1 | 0.958 | 0.973 | 0.975 | 1 | 0.95 | 0.983 | 0.98 | 0.974 | 0.774 | 0.907 | 0.91 | 0.941 | 0.94 | 0.998 | 0.981 |
| 197 | 1 | 0.952 | 0.898 | 0.912 | 1 | 0.958 | 0.973 | 0.975 | 1 | 0.95 | 0.983 | 0.98 | 0.974 | 0.774 | 0.907 | 0.91 | 0.941 | 0.94 | 0.998 | 0.981 |
| 198 | 1 | 0.952 | 0.898 | 0.912 | 1 | 0.958 | 0.973 | 0.975 | 1 | 0.95 | 0.983 | 0.98 | 0.974 | 0.774 | 0.907 | 0.91 | 0.941 | 0.94 | 0.998 | 0.981 |
| 199 | 1 | 0.952 | 0.902 | 0.915 | 1 | 0.958 | 0.973 | 0.975 | 1 | 0.95 | 0.983 | 0.98 | 0.974 | 0.774 | 0.908 | 0.911 | 0.941 | 0.94 | 0.998 | 0.981 |
| 200 | 1 | 0.952 | 0.902 | 0.915 | 1 | 0.958 | 0.973 | 0.975 | 1 | 0.95 | 0.983 | 0.98 | 0.974 | 0.774 | 0.908 | 0.911 | 0.941 | 0.94 | 0.998 | 0.981 |
| 201 | 1 | 0.952 | 0.905 | 0.917 | 1 | 0.958 | 0.973 | 0.975 | 1 | 0.95 | 0.983 | 0.98 | 0.974 | 0.774 | 0.911 | 0.913 | 0.941 | 0.94 | 0.998 | 0.981 |
| 202 | 1 | 0.952 | 0.902 | 0.915 | 1 | 0.958 | 0.97 | 0.974 | 1 | 0.95 | 0.983 | 0.98 | 0.974 | 0.774 | 0.909 | 0.912 | 0.941 | 0.928 | 0.998 | 0.9798 |
| 203 | 1 | 0.952 | 0.902 | 0.915 | 1 | 0.958 | 0.97 | 0.974 | 1 | 0.95 | 0.983 | 0.98 | 0.974 | 0.774 | 0.909 | 0.912 | 0.941 | 0.94 | 0.998 | 0.981 |
| 204 | 1 | 0.952 | 0.898 | 0.912 | 1 | 0.958 | 0.97 | 0.974 | 1 | 0.95 | 0.986 | 0.982 | 0.981 | 0.774 | 0.908 | 0.912 | 0.947 | 0.94 | 0.998 | 0.9821 |
| 205 | 1 | 0.952 | 0.898 | 0.912 | 1 | 0.958 | 0.97 | 0.974 | 1 | 0.95 | 0.986 | 0.982 | 0.974 | 0.774 | 0.908 | 0.911 | 0.947 | 0.94 | 0.998 | 0.9821 |
| 206 | 1 | 0.952 | 0.902 | 0.915 | 1 | 0.958 | 0.973 | 0.975 | 1 | 0.95 | 0.986 | 0.982 | 0.974 | 0.774 | 0.909 | 0.912 | 0.947 | 0.94 | 0.998 | 0.9821 |
| 207 | 1 | 0.952 | 0.895 | 0.909 | 1 | 0.958 | 0.973 | 0.975 | 1 | 0.95 | 0.983 | 0.98 | 0.974 | 0.774 | 0.905 | 0.909 | 0.952 | 0.94 | 0.998 | 0.9832 |
| 208 | 1 | 0.952 | 0.895 | 0.909 | 1 | 0.958 | 0.973 | 0.975 | 1 | 0.95 | 0.983 | 0.98 | 0.974 | 0.774 | 0.905 | 0.909 | 0.952 | 0.94 | 0.998 | 0.9832 |
| 209 | 1 | 0.952 | 0.895 | 0.909 | 1 | 0.958 | 0.973 | 0.975 | 1 | 0.95 | 0.983 | 0.98 | 0.974 | 0.774 | 0.905 | 0.909 | 0.952 | 0.94 | 0.998 | 0.9832 |
| 210 | 1 | 0.952 | 0.895 | 0.909 | 1 | 0.958 | 0.973 | 0.975 | 1 | 0.95 | 0.983 | 0.98 | 0.974 | 0.774 | 0.907 | 0.91 | 0.957 | 0.94 | 0.998 | 0.9843 |
| 211 | 1 | 0.952 | 0.898 | 0.912 | 1 | 0.958 | 0.973 | 0.975 | 1 | 0.95 | 0.983 | 0.98 | 0.974 | 0.774 | 0.908 | 0.911 | 0.963 | 0.94 | 0.998 | 0.9854 |
| 212 | 1 | 0.952 | 0.898 | 0.912 | 1 | 0.958 | 0.973 | 0.975 | 1 | 0.95 | 0.983 | 0.98 | 0.974 | 0.774 | 0.907 | 0.91 | 0.952 | 0.94 | 0.998 | 0.9832 |
| 213 | 1 | 0.952 | 0.888 | 0.904 | 1 | 0.958 | 0.97 | 0.974 | 1 | 0.95 | 0.983 | 0.98 | 0.981 | 0.774 | 0.903 | 0.908 | 0.952 | 0.94 | 0.998 | 0.9832 |
| 214 | 1 | 0.952 | 0.888 | 0.904 | 1 | 0.958 | 0.97 | 0.974 | 1 | 0.95 | 0.983 | 0.98 | 0.981 | 0.774 | 0.903 | 0.908 | 0.957 | 0.94 | 0.998 | 0.9843 |
| 215 | 1 | 0.952 | 0.888 | 0.904 | 1 | 0.958 | 0.965 | 0.97 | 1 | 0.95 | 0.983 | 0.98 | 0.981 | 0.774 | 0.901 | 0.907 | 0.963 | 0.94 | 0.998 | 0.9854 |
| 216 | 1 | 0.952 | 0.892 | 0.906 | 1 | 0.958 | 0.968 | 0.972 | 1 | 0.95 | 0.983 | 0.98 | 0.981 | 0.774 | 0.901 | 0.907 | 0.963 | 0.94 | 0.998 | 0.9854 |
| 217 | 1 | 0.952 | 0.892 | 0.906 | 1 | 0.958 | 0.97 | 0.974 | 1 | 0.95 | 0.983 | 0.98 | 0.981 | 0.774 | 0.899 | 0.905 | 0.963 | 0.94 | 0.998 | 0.9854 |
| 218 | 1 | 0.952 | 0.892 | 0.906 | 1 | 0.958 | 0.97 | 0.974 | 1 | 0.95 | 0.983 | 0.98 | 0.981 | 0.774 | 0.899 | 0.905 | 0.963 | 0.94 | 0.998 | 0.9854 |
| 219 | 1 | 0.952 | 0.888 | 0.904 | 1 | 0.958 | 0.97 | 0.974 | 1 | 0.95 | 0.983 | 0.98 | 0.981 | 0.774 | 0.896 | 0.903 | 0.968 | 0.94 | 0.998 | 0.9866 |
| 220 | 1 | 0.952 | 0.895 | 0.909 | 1 | 0.958 | 0.97 | 0.974 | 1 | 0.95 | 0.983 | 0.98 | 0.981 | 0.774 | 0.899 | 0.905 | 0.968 | 0.94 | 0.998 | 0.9866 |
| 221 | 1 | 0.952 | 0.895 | 0.909 | 1 | 0.958 | 0.97 | 0.974 | 1 | 0.95 | 0.983 | 0.98 | 0.981 | 0.774 | 0.899 | 0.905 | 0.968 | 0.94 | 0.998 | 0.9866 |
| 222 | 1 | 0.952 | 0.895 | 0.909 | 1 | 0.958 | 0.97 | 0.974 | 1 | 0.95 | 0.983 | 0.98 | 0.981 | 0.774 | 0.899 | 0.905 | 0.968 | 0.94 | 0.998 | 0.9866 |
| 223 | 1 | 0.952 | 0.888 | 0.904 | 1 | 0.958 | 0.968 | 0.972 | 1 | 0.95 | 0.981 | 0.978 | 0.981 | 0.774 | 0.897 | 0.904 | 0.968 | 0.94 | 0.998 | 0.9866 |
| 224 | 1 | 0.952 | 0.892 | 0.906 | 1 | 0.958 | 0.97 | 0.974 | 1 | 0.95 | 0.983 | 0.98 | 0.981 | 0.774 | 0.901 | 0.907 | 0.957 | 0.94 | 0.998 | 0.9843 |
| 225 | 1 | 0.952 | 0.892 | 0.906 | 1 | 0.958 | 0.97 | 0.974 | 1 | 0.95 | 0.983 | 0.98 | 0.981 | 0.774 | 0.9 | 0.906 | 0.968 | 0.94 | 0.998 | 0.9866 |
| 226 | 1 | 0.952 | 0.892 | 0.906 | 1 | 0.958 | 0.97 | 0.974 | 1 | 0.95 | 0.983 | 0.98 | 0.981 | 0.774 | 0.9 | 0.906 | 0.968 | 0.94 | 0.998 | 0.9866 |
| 227 | 1 | 0.952 | 0.888 | 0.904 | 1 | 0.958 | 0.97 | 0.974 | 1 | 0.95 | 0.983 | 0.98 | 0.981 | 0.774 | 0.9 | 0.906 | 0.968 | 0.94 | 0.998 | 0.9866 |
| 228 | 1 | 0.952 | 0.892 | 0.906 | 1 | 0.958 | 0.97 | 0.974 | 1 | 0.95 | 0.983 | 0.98 | 0.981 | 0.774 | 0.899 | 0.905 | 0.968 | 0.94 | 0.998 | 0.9866 |
| 229 | 1 | 0.952 | 0.892 | 0.906 | 1 | 0.958 | 0.97 | 0.974 | 1 | 0.95 | 0.983 | 0.98 | 0.981 | 0.774 | 0.9 | 0.906 | 0.957 | 0.94 | 0.998 | 0.9843 |
| 230 | 1 | 0.952 | 0.892 | 0.906 | 1 | 0.958 | 0.97 | 0.974 | 1 | 0.95 | 0.983 | 0.98 | 0.981 | 0.774 | 0.9 | 0.906 | 0.957 | 0.94 | 0.998 | 0.9843 |
| 231 | 1 | 0.952 | 0.888 | 0.904 | 1 | 0.958 | 0.97 | 0.974 | 1 | 0.95 | 0.983 | 0.98 | 0.981 | 0.774 | 0.9 | 0.906 | 0.963 | 0.94 | 0.998 | 0.9854 |
| 232 | 1 | 0.952 | 0.892 | 0.906 | 1 | 0.958 | 0.97 | 0.974 | 1 | 0.95 | 0.983 | 0.98 | 0.981 | 0.774 | 0.903 | 0.908 | 0.957 | 0.94 | 0.998 | 0.9843 |
| 233 | 1 | 0.952 | 0.892 | 0.906 | 1 | 0.958 | 0.97 | 0.974 | 1 | 0.95 | 0.983 | 0.98 | 0.974 | 0.774 | 0.905 | 0.909 | 0.957 | 0.94 | 0.998 | 0.9843 |
| 234 | 1 | 0.952 | 0.895 | 0.909 | 1 | 0.958 | 0.97 | 0.974 | 1 | 0.95 | 0.981 | 0.978 | 0.974 | 0.774 | 0.903 | 0.907 | 0.968 | 0.94 | 0.998 | 0.9866 |
| 235 | 1 | 0.952 | 0.898 | 0.912 | 1 | 0.958 | 0.97 | 0.974 | 1 | 0.95 | 0.983 | 0.98 | 0.974 | 0.774 | 0.905 | 0.909 | 0.963 | 0.94 | 0.998 | 0.9854 |
| 236 | 1 | 0.952 | 0.898 | 0.912 | 1 | 0.958 | 0.97 | 0.974 | 1 | 0.95 | 0.983 | 0.98 | 0.974 | 0.774 | 0.905 | 0.909 | 0.963 | 0.94 | 0.998 | 0.9854 |
| 237 | 1 | 0.952 | 0.898 | 0.912 | 1 | 0.958 | 0.97 | 0.974 | 1 | 0.95 | 0.983 | 0.98 | 0.974 | 0.774 | 0.905 | 0.909 | 0.963 | 0.94 | 0.998 | 0.9854 |
| 238 | 1 | 0.952 | 0.895 | 0.909 | 1 | 0.958 | 0.97 | 0.974 | 1 | 0.95 | 0.983 | 0.98 | 0.981 | 0.774 | 0.904 | 0.909 | 0.963 | 0.94 | 0.998 | 0.9854 |
| 239 | 1 | 0.952 | 0.898 | 0.912 | 1 | 0.958 | 0.97 | 0.974 | 1 | 0.95 | 0.983 | 0.98 | 0.981 | 0.774 | 0.904 | 0.909 | 0.963 | 0.94 | 0.998 | 0.9854 |
| 240 | 1 | 0.952 | 0.898 | 0.912 | 1 | 0.958 | 0.97 | 0.974 | 1 | 0.95 | 0.983 | 0.98 | 0.981 | 0.774 | 0.904 | 0.909 | 0.963 | 0.94 | 0.998 | 0.9854 |
| 241 | 1 | 0.952 | 0.898 | 0.912 | 1 | 0.938 | 0.97 | 0.972 | 1 | 0.95 | 0.983 | 0.98 | 0.981 | 0.774 | 0.904 | 0.909 | 0.963 | 0.94 | 0.998 | 0.9854 |
| 242 | 1 | 0.952 | 0.895 | 0.909 | 1 | 0.938 | 0.97 | 0.972 | 1 | 0.95 | 0.983 | 0.98 | 0.974 | 0.774 | 0.904 | 0.908 | 0.963 | 0.952 | 0.998 | 0.9866 |
| 243 | 1 | 0.952 | 0.892 | 0.906 | 1 | 0.958 | 0.968 | 0.972 | 1 | 0.95 | 0.981 | 0.978 | 0.987 | 0.774 | 0.901 | 0.908 | 0.963 | 0.952 | 0.998 | 0.9866 |
| 244 | 1 | 0.952 | 0.892 | 0.906 | 1 | 0.958 | 0.968 | 0.972 | 1 | 0.95 | 0.981 | 0.978 | 0.987 | 0.774 | 0.901 | 0.908 | 0.963 | 0.952 | 0.998 | 0.9866 |
| 245 | 1 | 0.952 | 0.892 | 0.906 | 1 | 0.958 | 0.968 | 0.972 | 1 | 0.95 | 0.981 | 0.978 | 0.987 | 0.774 | 0.901 | 0.908 | 0.963 | 0.952 | 0.998 | 0.9866 |
| 246 | 1 | 0.952 | 0.892 | 0.906 | 1 | 0.958 | 0.968 | 0.972 | 1 | 0.95 | 0.981 | 0.978 | 0.987 | 0.774 | 0.901 | 0.908 | 0.963 | 0.952 | 0.998 | 0.9866 |
| 247 | 1 | 0.952 | 0.895 | 0.909 | 1 | 0.958 | 0.965 | 0.97 | 1 | 0.95 | 0.983 | 0.98 | 0.987 | 0.774 | 0.901 | 0.908 | 0.963 | 0.952 | 0.998 | 0.9866 |
| 248 | 1 | 0.952 | 0.895 | 0.909 | 1 | 0.958 | 0.965 | 0.97 | 1 | 0.95 | 0.983 | 0.98 | 0.987 | 0.774 | 0.901 | 0.908 | 0.957 | 0.952 | 0.998 | 0.9854 |
| 249 | 1 | 0.952 | 0.898 | 0.912 | 1 | 0.958 | 0.97 | 0.974 | 1 | 0.95 | 0.983 | 0.98 | 0.987 | 0.774 | 0.901 | 0.908 | 0.963 | 0.952 | 0.998 | 0.9866 |
| 250 | 1 | 0.952 | 0.898 | 0.912 | 1 | 0.958 | 0.97 | 0.974 | 1 | 0.95 | 0.983 | 0.98 | 0.987 | 0.774 | 0.901 | 0.908 | 0.963 | 0.94 | 0.998 | 0.9854 |
| 251 | 1 | 0.952 | 0.898 | 0.912 | 1 | 0.958 | 0.97 | 0.974 | 1 | 0.95 | 0.983 | 0.98 | 0.987 | 0.774 | 0.901 | 0.908 | 0.963 | 0.94 | 0.998 | 0.9854 |
| 252 | 1 | 0.952 | 0.898 | 0.912 | 1 | 0.958 | 0.97 | 0.974 | 1 | 0.95 | 0.983 | 0.98 | 0.974 | 0.774 | 0.904 | 0.908 | 0.963 | 0.94 | 0.998 | 0.9854 |
| 253 | 1 | 0.952 | 0.898 | 0.912 | 1 | 0.958 | 0.97 | 0.974 | 1 | 0.95 | 0.983 | 0.98 | 0.981 | 0.774 | 0.903 | 0.908 | 0.963 | 0.94 | 0.998 | 0.9854 |
| 254 | 1 | 0.952 | 0.898 | 0.912 | 1 | 0.958 | 0.97 | 0.974 | 1 | 0.95 | 0.983 | 0.98 | 0.981 | 0.774 | 0.903 | 0.908 | 0.963 | 0.952 | 0.998 | 0.9866 |
| 255 | 1 | 0.952 | 0.898 | 0.912 | 1 | 0.958 | 0.97 | 0.974 | 1 | 0.95 | 0.983 | 0.98 | 0.981 | 0.774 | 0.903 | 0.908 | 0.963 | 0.952 | 0.998 | 0.9866 |
| 256 | 1 | 0.952 | 0.898 | 0.912 | 1 | 0.958 | 0.968 | 0.972 | 1 | 0.95 | 0.983 | 0.98 | 0.981 | 0.774 | 0.904 | 0.909 | 0.963 | 0.952 | 0.998 | 0.9866 |
| 257 | 1 | 0.952 | 0.895 | 0.909 | 1 | 0.958 | 0.968 | 0.972 | 1 | 0.95 | 0.978 | 0.976 | 0.974 | 0.774 | 0.905 | 0.909 | 0.963 | 0.952 | 0.998 | 0.9866 |
| 258 | 1 | 0.952 | 0.895 | 0.909 | 1 | 0.958 | 0.968 | 0.972 | 1 | 0.95 | 0.981 | 0.978 | 0.974 | 0.774 | 0.908 | 0.911 | 0.963 | 0.952 | 0.998 | 0.9866 |
| 259 | 1 | 0.952 | 0.895 | 0.909 | 1 | 0.958 | 0.968 | 0.972 | 1 | 0.95 | 0.978 | 0.976 | 0.974 | 0.774 | 0.907 | 0.91 | 0.963 | 0.952 | 0.998 | 0.9866 |
| 260 | 1 | 0.952 | 0.898 | 0.912 | 1 | 0.958 | 0.968 | 0.972 | 1 | 0.95 | 0.981 | 0.978 | 0.974 | 0.774 | 0.909 | 0.912 | 0.963 | 0.952 | 0.998 | 0.9866 |
| 261 | 1 | 0.952 | 0.895 | 0.909 | 1 | 0.958 | 0.965 | 0.97 | 1 | 0.95 | 0.978 | 0.976 | 0.974 | 0.774 | 0.904 | 0.908 | 0.963 | 0.952 | 0.998 | 0.9866 |
| 262 | 1 | 0.952 | 0.895 | 0.909 | 1 | 0.958 | 0.965 | 0.97 | 1 | 0.95 | 0.978 | 0.976 | 0.974 | 0.774 | 0.904 | 0.908 | 0.963 | 0.94 | 0.998 | 0.9854 |
| 263 | 1 | 0.952 | 0.898 | 0.912 | 1 | 0.958 | 0.965 | 0.97 | 1 | 0.95 | 0.978 | 0.976 | 0.974 | 0.774 | 0.903 | 0.907 | 0.963 | 0.94 | 0.998 | 0.9854 |
| 264 | 1 | 0.952 | 0.898 | 0.912 | 1 | 0.958 | 0.965 | 0.97 | 1 | 0.95 | 0.978 | 0.976 | 0.981 | 0.774 | 0.901 | 0.907 | 0.963 | 0.94 | 0.998 | 0.9854 |
| 265 | 1 | 0.952 | 0.892 | 0.906 | 1 | 0.958 | 0.97 | 0.974 | 1 | 0.95 | 0.978 | 0.976 | 0.974 | 0.774 | 0.903 | 0.907 | 0.963 | 0.94 | 0.998 | 0.9854 |
| 266 | 1 | 0.952 | 0.892 | 0.906 | 1 | 0.958 | 0.97 | 0.974 | 1 | 0.95 | 0.978 | 0.976 | 0.974 | 0.774 | 0.903 | 0.907 | 0.963 | 0.94 | 0.998 | 0.9854 |
| 267 | 1 | 0.952 | 0.895 | 0.909 | 1 | 0.958 | 0.97 | 0.974 | 1 | 0.95 | 0.978 | 0.976 | 0.974 | 0.774 | 0.903 | 0.907 | 0.963 | 0.94 | 0.998 | 0.9854 |
| 268 | 1 | 0.952 | 0.895 | 0.909 | 1 | 0.958 | 0.97 | 0.974 | 1 | 0.95 | 0.978 | 0.976 | 0.981 | 0.774 | 0.903 | 0.908 | 0.963 | 0.94 | 0.998 | 0.9854 |
| 269 | 1 | 0.952 | 0.888 | 0.904 | 1 | 0.958 | 0.963 | 0.968 | 1 | 0.95 | 0.975 | 0.974 | 0.987 | 0.774 | 0.896 | 0.904 | 0.963 | 0.94 | 0.998 | 0.9854 |
| 270 | 1 | 0.952 | 0.888 | 0.904 | 1 | 0.958 | 0.963 | 0.968 | 1 | 0.95 | 0.975 | 0.974 | 0.987 | 0.774 | 0.896 | 0.904 | 0.968 | 0.94 | 0.998 | 0.9866 |
| 271 | 1 | 0.952 | 0.885 | 0.901 | 1 | 0.958 | 0.963 | 0.968 | 1 | 0.95 | 0.975 | 0.974 | 0.987 | 0.774 | 0.896 | 0.904 | 0.968 | 0.94 | 0.998 | 0.9866 |
| 272 | 1 | 0.952 | 0.885 | 0.901 | 1 | 0.958 | 0.963 | 0.968 | 1 | 0.95 | 0.975 | 0.974 | 0.987 | 0.774 | 0.896 | 0.904 | 0.968 | 0.94 | 0.998 | 0.9866 |
| 273 | 1 | 0.952 | 0.885 | 0.901 | 1 | 0.958 | 0.963 | 0.968 | 1 | 0.95 | 0.975 | 0.974 | 0.987 | 0.774 | 0.896 | 0.904 | 0.968 | 0.94 | 0.998 | 0.9866 |
| 274 | 1 | 0.952 | 0.885 | 0.901 | 1 | 0.958 | 0.963 | 0.968 | 1 | 0.95 | 0.975 | 0.974 | 0.987 | 0.774 | 0.896 | 0.904 | 0.963 | 0.94 | 0.998 | 0.9854 |
| 275 | 1 | 0.952 | 0.888 | 0.904 | 1 | 0.958 | 0.963 | 0.968 | 1 | 0.95 | 0.975 | 0.974 | 0.981 | 0.774 | 0.896 | 0.903 | 0.963 | 0.94 | 0.998 | 0.9854 |
| 276 | 1 | 0.952 | 0.888 | 0.904 | 1 | 0.958 | 0.96 | 0.966 | 1 | 0.95 | 0.975 | 0.974 | 0.981 | 0.774 | 0.897 | 0.904 | 0.963 | 0.94 | 0.998 | 0.9854 |
| 277 | 1 | 0.952 | 0.885 | 0.901 | 1 | 0.958 | 0.963 | 0.968 | 1 | 0.95 | 0.975 | 0.974 | 0.981 | 0.774 | 0.896 | 0.903 | 0.963 | 0.94 | 0.998 | 0.9854 |
| 278 | 1 | 0.952 | 0.878 | 0.895 | 1 | 0.938 | 0.958 | 0.962 | 1 | 0.95 | 0.975 | 0.974 | 0.987 | 0.774 | 0.886 | 0.896 | 0.968 | 0.94 | 0.998 | 0.9866 |
| 279 | 1 | 0.952 | 0.878 | 0.895 | 1 | 0.938 | 0.958 | 0.962 | 1 | 0.95 | 0.972 | 0.972 | 0.987 | 0.774 | 0.888 | 0.897 | 0.968 | 0.94 | 0.998 | 0.9866 |
| 280 | 1 | 0.952 | 0.878 | 0.895 | 1 | 0.958 | 0.958 | 0.964 | 1 | 0.95 | 0.972 | 0.972 | 0.987 | 0.774 | 0.888 | 0.897 | 0.968 | 0.94 | 0.998 | 0.9866 |
| 281 | 1 | 0.952 | 0.878 | 0.895 | 1 | 0.958 | 0.955 | 0.962 | 1 | 0.95 | 0.972 | 0.972 | 0.987 | 0.774 | 0.888 | 0.897 | 0.968 | 0.94 | 0.998 | 0.9866 |
| 282 | 1 | 0.952 | 0.878 | 0.895 | 1 | 0.958 | 0.955 | 0.962 | 1 | 0.95 | 0.972 | 0.972 | 0.987 | 0.774 | 0.886 | 0.896 | 0.968 | 0.94 | 0.998 | 0.9866 |
| 283 | 1 | 0.952 | 0.878 | 0.895 | 1 | 0.958 | 0.955 | 0.962 | 1 | 0.95 | 0.972 | 0.972 | 0.987 | 0.774 | 0.889 | 0.899 | 0.968 | 0.94 | 0.998 | 0.9866 |
| 284 | 1 | 0.952 | 0.878 | 0.895 | 1 | 0.958 | 0.955 | 0.962 | 1 | 0.95 | 0.972 | 0.972 | 0.987 | 0.774 | 0.889 | 0.899 | 0.968 | 0.94 | 0.998 | 0.9866 |
| 285 | 1 | 0.952 | 0.878 | 0.895 | 1 | 0.958 | 0.955 | 0.962 | 1 | 0.95 | 0.972 | 0.972 | 0.987 | 0.774 | 0.888 | 0.897 | 0.968 | 0.94 | 0.998 | 0.9866 |
| 286 | 1 | 0.952 | 0.878 | 0.895 | 1 | 0.958 | 0.955 | 0.962 | 1 | 0.95 | 0.972 | 0.972 | 0.987 | 0.774 | 0.885 | 0.895 | 0.968 | 0.94 | 0.998 | 0.9866 |
| 287 | 1 | 0.952 | 0.878 | 0.895 | 1 | 0.958 | 0.958 | 0.964 | 1 | 0.95 | 0.972 | 0.972 | 0.987 | 0.774 | 0.888 | 0.897 | 0.968 | 0.94 | 0.998 | 0.9866 |
| 288 | 1 | 0.952 | 0.878 | 0.895 | 1 | 0.938 | 0.958 | 0.962 | 1 | 0.95 | 0.972 | 0.972 | 0.987 | 0.774 | 0.888 | 0.897 | 0.968 | 0.94 | 0.998 | 0.9866 |
| 289 | 1 | 0.952 | 0.878 | 0.895 | 1 | 0.938 | 0.958 | 0.962 | 1 | 0.95 | 0.972 | 0.972 | 0.987 | 0.774 | 0.888 | 0.897 | 0.968 | 0.94 | 0.998 | 0.9866 |
| 290 | 1 | 0.952 | 0.878 | 0.895 | 1 | 0.938 | 0.96 | 0.964 | 1 | 0.95 | 0.972 | 0.972 | 0.981 | 0.774 | 0.89 | 0.899 | 0.968 | 0.94 | 0.998 | 0.9866 |
| 291 | 1 | 0.952 | 0.878 | 0.895 | 1 | 0.938 | 0.96 | 0.964 | 1 | 0.95 | 0.972 | 0.972 | 0.987 | 0.774 | 0.89 | 0.9 | 0.963 | 0.94 | 0.998 | 0.9854 |
| 292 | 1 | 0.952 | 0.878 | 0.895 | 1 | 0.938 | 0.96 | 0.964 | 1 | 0.95 | 0.972 | 0.972 | 0.981 | 0.774 | 0.89 | 0.899 | 0.963 | 0.94 | 0.998 | 0.9854 |
| 293 | 1 | 0.952 | 0.878 | 0.895 | 1 | 0.938 | 0.96 | 0.964 | 1 | 0.95 | 0.972 | 0.972 | 0.981 | 0.774 | 0.89 | 0.899 | 0.963 | 0.94 | 0.998 | 0.9854 |
| 294 | 1 | 0.952 | 0.878 | 0.895 | 1 | 0.938 | 0.96 | 0.964 | 1 | 0.95 | 0.972 | 0.972 | 0.987 | 0.774 | 0.892 | 0.901 | 0.968 | 0.94 | 0.998 | 0.9866 |
| 295 | 1 | 0.952 | 0.878 | 0.895 | 1 | 0.938 | 0.96 | 0.964 | 1 | 0.95 | 0.972 | 0.972 | 0.987 | 0.774 | 0.892 | 0.901 | 0.968 | 0.94 | 0.998 | 0.9866 |
| 296 | 1 | 0.952 | 0.878 | 0.895 | 1 | 0.938 | 0.96 | 0.964 | 1 | 0.95 | 0.97 | 0.97 | 0.981 | 0.774 | 0.889 | 0.897 | 0.968 | 0.94 | 0.998 | 0.9866 |
| 297 | 1 | 0.952 | 0.875 | 0.893 | 1 | 0.938 | 0.958 | 0.962 | 1 | 0.95 | 0.972 | 0.972 | 0.987 | 0.774 | 0.885 | 0.895 | 0.968 | 0.94 | 0.998 | 0.9866 |
| 298 | 1 | 0.952 | 0.875 | 0.893 | 1 | 0.938 | 0.958 | 0.962 | 1 | 0.95 | 0.972 | 0.972 | 0.981 | 0.774 | 0.885 | 0.894 | 0.968 | 0.94 | 0.998 | 0.9866 |
| 299 | 1 | 0.952 | 0.875 | 0.893 | 1 | 0.938 | 0.958 | 0.962 | 1 | 0.95 | 0.972 | 0.972 | 0.981 | 0.774 | 0.885 | 0.894 | 0.968 | 0.94 | 0.998 | 0.9866 |
| 300 | 1 | 0.952 | 0.878 | 0.895 | 1 | 0.938 | 0.958 | 0.962 | 1 | 0.95 | 0.972 | 0.972 | 0.981 | 0.774 | 0.885 | 0.894 | 0.968 | 0.94 | 0.998 | 0.9866 |
| 301 | 1 | 0.952 | 0.878 | 0.895 | 1 | 0.938 | 0.958 | 0.962 | 1 | 0.95 | 0.972 | 0.972 | 0.981 | 0.774 | 0.885 | 0.894 | 0.968 | 0.94 | 0.998 | 0.9866 |
| 302 | 1 | 0.952 | 0.878 | 0.895 | 1 | 0.938 | 0.958 | 0.962 | 1 | 0.95 | 0.972 | 0.972 | 0.981 | 0.774 | 0.886 | 0.895 | 0.968 | 0.94 | 0.998 | 0.9866 |
| 303 | 1 | 0.952 | 0.878 | 0.895 | 1 | 0.938 | 0.958 | 0.962 | 1 | 0.95 | 0.972 | 0.972 | 0.981 | 0.774 | 0.886 | 0.895 | 0.968 | 0.94 | 0.998 | 0.9866 |
| 304 | 1 | 0.952 | 0.875 | 0.893 | 1 | 0.938 | 0.958 | 0.962 | 1 | 0.95 | 0.972 | 0.972 | 0.981 | 0.774 | 0.885 | 0.894 | 0.968 | 0.928 | 0.998 | 0.9854 |
| 305 | 1 | 0.952 | 0.875 | 0.893 | 1 | 0.938 | 0.958 | 0.962 | 1 | 0.95 | 0.972 | 0.972 | 0.981 | 0.774 | 0.884 | 0.893 | 0.968 | 0.94 | 0.998 | 0.9866 |
| 306 | 1 | 0.952 | 0.875 | 0.893 | 1 | 0.938 | 0.958 | 0.962 | 1 | 0.95 | 0.972 | 0.972 | 0.981 | 0.774 | 0.884 | 0.893 | 0.968 | 0.94 | 0.998 | 0.9866 |
| 307 | 1 | 0.952 | 0.871 | 0.89 | 1 | 0.938 | 0.958 | 0.962 | 1 | 0.95 | 0.972 | 0.972 | 0.981 | 0.774 | 0.882 | 0.892 | 0.968 | 0.94 | 0.998 | 0.9866 |
| 308 | 1 | 0.952 | 0.871 | 0.89 | 1 | 0.938 | 0.958 | 0.962 | 1 | 0.95 | 0.972 | 0.972 | 0.981 | 0.774 | 0.884 | 0.893 | 0.968 | 0.94 | 0.998 | 0.9866 |
| 309 | 1 | 0.952 | 0.875 | 0.893 | 1 | 0.938 | 0.958 | 0.962 | 1 | 0.95 | 0.972 | 0.972 | 0.981 | 0.774 | 0.884 | 0.893 | 0.963 | 0.94 | 0.998 | 0.9854 |
| 310 | 1 | 0.952 | 0.875 | 0.893 | 1 | 0.938 | 0.958 | 0.962 | 1 | 0.95 | 0.972 | 0.972 | 0.981 | 0.774 | 0.884 | 0.893 | 0.968 | 0.94 | 0.998 | 0.9866 |
| 311 | 1 | 0.952 | 0.878 | 0.895 | 1 | 0.938 | 0.958 | 0.962 | 1 | 0.95 | 0.972 | 0.972 | 0.981 | 0.774 | 0.884 | 0.893 | 0.968 | 0.94 | 0.998 | 0.9866 |
| 312 | 1 | 0.952 | 0.878 | 0.895 | 1 | 0.938 | 0.958 | 0.962 | 1 | 0.95 | 0.972 | 0.972 | 0.981 | 0.774 | 0.885 | 0.894 | 0.968 | 0.94 | 0.998 | 0.9866 |
| 313 | 1 | 0.952 | 0.878 | 0.895 | 1 | 0.938 | 0.958 | 0.962 | 1 | 0.95 | 0.972 | 0.972 | 0.981 | 0.774 | 0.882 | 0.892 | 0.968 | 0.94 | 0.998 | 0.9866 |
| 314 | 1 | 0.952 | 0.875 | 0.893 | 1 | 0.938 | 0.958 | 0.962 | 1 | 0.95 | 0.972 | 0.972 | 0.981 | 0.774 | 0.882 | 0.892 | 0.968 | 0.94 | 0.998 | 0.9866 |
| 315 | 1 | 0.952 | 0.875 | 0.893 | 1 | 0.938 | 0.958 | 0.962 | 1 | 0.95 | 0.972 | 0.972 | 0.981 | 0.774 | 0.882 | 0.892 | 0.968 | 0.94 | 0.998 | 0.9866 |
| 316 | 1 | 0.952 | 0.878 | 0.895 | 1 | 0.938 | 0.955 | 0.96 | 1 | 0.95 | 0.972 | 0.972 | 0.981 | 0.774 | 0.882 | 0.892 | 0.963 | 0.94 | 0.998 | 0.9854 |
| 317 | 1 | 0.952 | 0.878 | 0.895 | 1 | 0.938 | 0.955 | 0.96 | 1 | 0.95 | 0.972 | 0.972 | 0.981 | 0.774 | 0.882 | 0.892 | 0.963 | 0.94 | 0.998 | 0.9854 |
| 318 | 1 | 0.952 | 0.878 | 0.895 | 1 | 0.938 | 0.958 | 0.962 | 1 | 0.95 | 0.972 | 0.972 | 0.981 | 0.774 | 0.884 | 0.893 | 0.963 | 0.94 | 0.998 | 0.9854 |
| 319 | 1 | 0.952 | 0.878 | 0.895 | 1 | 0.938 | 0.958 | 0.962 | 1 | 0.95 | 0.972 | 0.972 | 0.981 | 0.774 | 0.885 | 0.894 | 0.963 | 0.94 | 0.998 | 0.9854 |
| 320 | 1 | 0.952 | 0.871 | 0.89 | 1 | 0.938 | 0.955 | 0.96 | 1 | 0.95 | 0.972 | 0.972 | 0.981 | 0.774 | 0.881 | 0.891 | 0.963 | 0.94 | 0.997 | 0.9843 |
| 321 | 1 | 0.952 | 0.871 | 0.89 | 1 | 0.938 | 0.955 | 0.96 | 1 | 0.95 | 0.972 | 0.972 | 0.981 | 0.774 | 0.881 | 0.891 | 0.957 | 0.94 | 0.997 | 0.9832 |
| 322 | 1 | 0.952 | 0.875 | 0.893 | 1 | 0.938 | 0.958 | 0.962 | 1 | 0.95 | 0.972 | 0.972 | 0.981 | 0.774 | 0.882 | 0.892 | 0.957 | 0.94 | 0.997 | 0.9832 |
| 323 | 1 | 0.952 | 0.871 | 0.89 | 1 | 0.938 | 0.953 | 0.958 | 1 | 0.95 | 0.972 | 0.972 | 0.981 | 0.774 | 0.88 | 0.89 | 0.957 | 0.94 | 0.998 | 0.9843 |
| 324 | 1 | 0.952 | 0.871 | 0.89 | 1 | 0.938 | 0.953 | 0.958 | 1 | 0.95 | 0.972 | 0.972 | 0.981 | 0.774 | 0.88 | 0.89 | 0.957 | 0.94 | 0.998 | 0.9843 |
| 325 | 1 | 0.952 | 0.875 | 0.893 | 1 | 0.938 | 0.955 | 0.96 | 1 | 0.95 | 0.972 | 0.972 | 0.981 | 0.774 | 0.88 | 0.89 | 0.957 | 0.94 | 0.998 | 0.9843 |
| 326 | 1 | 0.952 | 0.871 | 0.89 | 1 | 0.938 | 0.955 | 0.96 | 1 | 0.95 | 0.972 | 0.972 | 0.981 | 0.774 | 0.88 | 0.89 | 0.957 | 0.94 | 0.997 | 0.9832 |
| 327 | 1 | 0.952 | 0.875 | 0.893 | 1 | 0.938 | 0.955 | 0.96 | 1 | 0.95 | 0.972 | 0.972 | 0.981 | 0.774 | 0.88 | 0.89 | 0.952 | 0.94 | 0.997 | 0.9821 |
| 328 | 1 | 0.952 | 0.875 | 0.893 | 1 | 0.938 | 0.955 | 0.96 | 1 | 0.95 | 0.972 | 0.972 | 0.981 | 0.774 | 0.88 | 0.89 | 0.952 | 0.94 | 0.997 | 0.9821 |
| 329 | 1 | 0.952 | 0.875 | 0.893 | 1 | 0.938 | 0.955 | 0.96 | 1 | 0.95 | 0.972 | 0.972 | 0.981 | 0.774 | 0.88 | 0.89 | 0.952 | 0.94 | 0.997 | 0.9821 |
| 330 | 1 | 0.952 | 0.875 | 0.893 | 1 | 0.938 | 0.955 | 0.96 | 1 | 0.95 | 0.972 | 0.972 | 0.974 | 0.774 | 0.882 | 0.891 | 0.952 | 0.94 | 0.997 | 0.9821 |
| 331 | 1 | 0.952 | 0.871 | 0.89 | 1 | 0.938 | 0.955 | 0.96 | 1 | 0.95 | 0.972 | 0.972 | 0.974 | 0.774 | 0.885 | 0.893 | 0.947 | 0.94 | 0.997 | 0.981 |
| 332 | 1 | 0.952 | 0.871 | 0.89 | 1 | 0.938 | 0.953 | 0.958 | 1 | 0.95 | 0.972 | 0.972 | 0.974 | 0.774 | 0.885 | 0.893 | 0.947 | 0.94 | 0.997 | 0.981 |
| 333 | 1 | 0.952 | 0.871 | 0.89 | 1 | 0.938 | 0.953 | 0.958 | 1 | 0.95 | 0.972 | 0.972 | 0.974 | 0.774 | 0.884 | 0.892 | 0.947 | 0.94 | 0.997 | 0.981 |
| 334 | 1 | 0.952 | 0.868 | 0.887 | 1 | 0.938 | 0.953 | 0.958 | 1 | 0.95 | 0.97 | 0.97 | 0.974 | 0.774 | 0.88 | 0.889 | 0.947 | 0.94 | 0.997 | 0.981 |
| 335 | 1 | 0.952 | 0.868 | 0.887 | 1 | 0.938 | 0.953 | 0.958 | 1 | 0.95 | 0.97 | 0.97 | 0.974 | 0.774 | 0.88 | 0.889 | 0.947 | 0.94 | 0.997 | 0.981 |
| 336 | 1 | 0.952 | 0.871 | 0.89 | 1 | 0.938 | 0.95 | 0.957 | 1 | 0.95 | 0.97 | 0.97 | 0.974 | 0.774 | 0.881 | 0.89 | 0.952 | 0.94 | 0.997 | 0.9821 |
| 337 | 1 | 0.952 | 0.871 | 0.89 | 1 | 0.938 | 0.95 | 0.957 | 1 | 0.95 | 0.97 | 0.97 | 0.974 | 0.774 | 0.881 | 0.89 | 0.952 | 0.94 | 0.997 | 0.9821 |
| 338 | 1 | 0.952 | 0.871 | 0.89 | 1 | 0.938 | 0.95 | 0.957 | 1 | 0.95 | 0.97 | 0.97 | 0.974 | 0.774 | 0.88 | 0.889 | 0.952 | 0.94 | 0.997 | 0.9821 |
| 339 | 1 | 0.952 | 0.871 | 0.89 | 1 | 0.938 | 0.948 | 0.955 | 1 | 0.95 | 0.97 | 0.97 | 0.974 | 0.774 | 0.88 | 0.889 | 0.947 | 0.94 | 0.997 | 0.981 |
| 340 | 1 | 0.952 | 0.871 | 0.89 | 1 | 0.938 | 0.948 | 0.955 | 1 | 0.95 | 0.97 | 0.97 | 0.974 | 0.774 | 0.88 | 0.889 | 0.952 | 0.94 | 0.998 | 0.9832 |
| 341 | 1 | 0.952 | 0.871 | 0.89 | 1 | 0.938 | 0.95 | 0.957 | 1 | 0.95 | 0.97 | 0.97 | 0.974 | 0.774 | 0.88 | 0.889 | 0.947 | 0.94 | 0.998 | 0.9821 |
| 342 | 1 | 0.952 | 0.871 | 0.89 | 1 | 0.938 | 0.95 | 0.957 | 1 | 0.95 | 0.97 | 0.97 | 0.974 | 0.774 | 0.88 | 0.889 | 0.947 | 0.94 | 0.998 | 0.9821 |
| 343 | 1 | 0.952 | 0.871 | 0.89 | 1 | 0.938 | 0.948 | 0.955 | 1 | 0.95 | 0.97 | 0.97 | 0.974 | 0.774 | 0.88 | 0.889 | 0.947 | 0.94 | 0.998 | 0.9821 |
| 344 | 1 | 0.952 | 0.871 | 0.89 | 1 | 0.938 | 0.948 | 0.955 | 1 | 0.95 | 0.97 | 0.97 | 0.974 | 0.774 | 0.88 | 0.889 | 0.947 | 0.94 | 0.998 | 0.9821 |
| 345 | 1 | 0.952 | 0.871 | 0.89 | 1 | 0.938 | 0.948 | 0.955 | 1 | 0.95 | 0.97 | 0.97 | 0.974 | 0.774 | 0.88 | 0.889 | 0.947 | 0.94 | 0.998 | 0.9821 |
| 346 | 1 | 0.952 | 0.871 | 0.89 | 1 | 0.938 | 0.948 | 0.955 | 1 | 0.95 | 0.97 | 0.97 | 0.974 | 0.774 | 0.88 | 0.889 | 0.952 | 0.94 | 0.997 | 0.9821 |
| 347 | 1 | 0.952 | 0.871 | 0.89 | 1 | 0.938 | 0.948 | 0.955 | 1 | 0.95 | 0.97 | 0.97 | 0.974 | 0.774 | 0.88 | 0.889 | 0.947 | 0.94 | 0.998 | 0.9821 |
| 348 | 1 | 0.952 | 0.871 | 0.89 | 1 | 0.938 | 0.95 | 0.957 | 1 | 0.95 | 0.97 | 0.97 | 0.974 | 0.774 | 0.881 | 0.89 | 0.941 | 0.94 | 0.998 | 0.981 |
| 349 | 1 | 0.952 | 0.871 | 0.89 | 1 | 0.938 | 0.95 | 0.957 | 1 | 0.95 | 0.97 | 0.97 | 0.974 | 0.774 | 0.881 | 0.89 | 0.936 | 0.94 | 0.998 | 0.9798 |
| 350 | 1 | 0.952 | 0.871 | 0.89 | 1 | 0.938 | 0.95 | 0.957 | 1 | 0.95 | 0.97 | 0.97 | 0.974 | 0.774 | 0.881 | 0.89 | 0.936 | 0.94 | 0.998 | 0.9798 |
| 351 | 1 | 0.952 | 0.875 | 0.893 | 1 | 0.938 | 0.953 | 0.958 | 1 | 0.95 | 0.97 | 0.97 | 0.974 | 0.774 | 0.88 | 0.889 | 0.941 | 0.94 | 0.997 | 0.9798 |
| 352 | 1 | 0.952 | 0.875 | 0.893 | 1 | 0.938 | 0.953 | 0.958 | 1 | 0.95 | 0.97 | 0.97 | 0.974 | 0.774 | 0.881 | 0.89 | 0.941 | 0.94 | 0.997 | 0.9798 |
| 353 | 1 | 0.952 | 0.875 | 0.893 | 1 | 0.938 | 0.953 | 0.958 | 1 | 0.95 | 0.97 | 0.97 | 0.974 | 0.774 | 0.88 | 0.889 | 0.941 | 0.94 | 0.997 | 0.9798 |
| 354 | 1 | 0.952 | 0.875 | 0.893 | 1 | 0.938 | 0.953 | 0.958 | 1 | 0.95 | 0.97 | 0.97 | 0.974 | 0.774 | 0.882 | 0.891 | 0.947 | 0.94 | 0.997 | 0.981 |
| 355 | 1 | 0.952 | 0.875 | 0.893 | 1 | 0.938 | 0.953 | 0.958 | 1 | 0.95 | 0.97 | 0.97 | 0.974 | 0.774 | 0.882 | 0.891 | 0.947 | 0.94 | 0.997 | 0.981 |
| 356 | 1 | 0.952 | 0.875 | 0.893 | 1 | 0.938 | 0.953 | 0.958 | 1 | 0.95 | 0.97 | 0.97 | 0.974 | 0.774 | 0.882 | 0.891 | 0.947 | 0.928 | 0.997 | 0.9798 |
| 357 | 1 | 0.952 | 0.875 | 0.893 | 1 | 0.938 | 0.953 | 0.958 | 1 | 0.95 | 0.97 | 0.97 | 0.981 | 0.774 | 0.882 | 0.892 | 0.947 | 0.928 | 0.997 | 0.9798 |
| 358 | 1 | 0.952 | 0.878 | 0.895 | 1 | 0.938 | 0.95 | 0.957 | 1 | 0.95 | 0.97 | 0.97 | 0.981 | 0.774 | 0.882 | 0.892 | 0.947 | 0.928 | 0.997 | 0.9798 |
| 359 | 1 | 0.952 | 0.878 | 0.895 | 1 | 0.938 | 0.95 | 0.957 | 1 | 0.95 | 0.97 | 0.97 | 0.981 | 0.774 | 0.882 | 0.892 | 0.947 | 0.928 | 0.997 | 0.9798 |
| 360 | 1 | 0.952 | 0.878 | 0.895 | 1 | 0.938 | 0.953 | 0.958 | 1 | 0.95 | 0.97 | 0.97 | 0.981 | 0.774 | 0.885 | 0.894 | 0.947 | 0.928 | 0.998 | 0.981 |
| 361 | 1 | 0.952 | 0.878 | 0.895 | 1 | 0.938 | 0.953 | 0.958 | 1 | 0.95 | 0.97 | 0.97 | 0.981 | 0.774 | 0.885 | 0.894 | 0.947 | 0.928 | 0.998 | 0.981 |
| 362 | 1 | 0.952 | 0.878 | 0.895 | 1 | 0.938 | 0.955 | 0.96 | 1 | 0.95 | 0.97 | 0.97 | 0.981 | 0.774 | 0.886 | 0.895 | 0.941 | 0.928 | 0.998 | 0.9798 |
| 363 | 1 | 0.952 | 0.878 | 0.895 | 1 | 0.938 | 0.955 | 0.96 | 1 | 0.95 | 0.97 | 0.97 | 0.981 | 0.774 | 0.886 | 0.895 | 0.947 | 0.928 | 0.998 | 0.981 |
| 364 | 1 | 0.952 | 0.878 | 0.895 | 1 | 0.938 | 0.955 | 0.96 | 1 | 0.95 | 0.97 | 0.97 | 0.981 | 0.774 | 0.886 | 0.895 | 0.947 | 0.928 | 0.997 | 0.9798 |
| 365 | 1 | 0.952 | 0.878 | 0.895 | 1 | 0.938 | 0.955 | 0.96 | 1 | 0.95 | 0.97 | 0.97 | 0.981 | 0.774 | 0.885 | 0.894 | 0.947 | 0.928 | 0.998 | 0.981 |
| 366 | 1 | 0.952 | 0.878 | 0.895 | 1 | 0.938 | 0.955 | 0.96 | 1 | 0.95 | 0.97 | 0.97 | 0.981 | 0.774 | 0.885 | 0.894 | 0.947 | 0.928 | 0.998 | 0.981 |
| 367 | 1 | 0.952 | 0.878 | 0.895 | 1 | 0.938 | 0.955 | 0.96 | 1 | 0.95 | 0.97 | 0.97 | 0.981 | 0.774 | 0.885 | 0.894 | 0.941 | 0.928 | 0.998 | 0.9798 |
| 368 | 1 | 0.952 | 0.878 | 0.895 | 1 | 0.938 | 0.955 | 0.96 | 1 | 0.95 | 0.97 | 0.97 | 0.981 | 0.774 | 0.885 | 0.894 | 0.947 | 0.928 | 0.998 | 0.981 |
| 369 | 1 | 0.952 | 0.878 | 0.895 | 1 | 0.938 | 0.955 | 0.96 | 1 | 0.95 | 0.97 | 0.97 | 0.981 | 0.774 | 0.885 | 0.894 | 0.947 | 0.928 | 0.998 | 0.981 |
| 370 | 1 | 0.952 | 0.878 | 0.895 | 1 | 0.938 | 0.955 | 0.96 | 1 | 0.95 | 0.97 | 0.97 | 0.981 | 0.774 | 0.885 | 0.894 | 0.947 | 0.928 | 0.998 | 0.981 |
| 371 | 1 | 0.952 | 0.878 | 0.895 | 1 | 0.938 | 0.955 | 0.96 | 1 | 0.95 | 0.97 | 0.97 | 0.981 | 0.774 | 0.885 | 0.894 | 0.947 | 0.928 | 0.998 | 0.981 |
| 372 | 1 | 0.952 | 0.881 | 0.898 | 1 | 0.938 | 0.958 | 0.962 | 1 | 0.95 | 0.97 | 0.97 | 0.981 | 0.774 | 0.886 | 0.895 | 0.947 | 0.928 | 0.998 | 0.981 |
| 373 | 1 | 0.952 | 0.881 | 0.898 | 1 | 0.938 | 0.958 | 0.962 | 1 | 0.95 | 0.97 | 0.97 | 0.974 | 0.774 | 0.89 | 0.897 | 0.947 | 0.928 | 0.998 | 0.981 |
| 374 | 1 | 0.952 | 0.881 | 0.898 | 1 | 0.938 | 0.958 | 0.962 | 1 | 0.95 | 0.97 | 0.97 | 0.974 | 0.774 | 0.89 | 0.897 | 0.947 | 0.928 | 0.998 | 0.981 |
| 375 | 1 | 0.952 | 0.881 | 0.898 | 1 | 0.938 | 0.958 | 0.962 | 1 | 0.95 | 0.97 | 0.97 | 0.974 | 0.774 | 0.889 | 0.896 | 0.947 | 0.928 | 0.997 | 0.9798 |
| 376 | 1 | 0.952 | 0.881 | 0.898 | 1 | 0.938 | 0.958 | 0.962 | 1 | 0.95 | 0.97 | 0.97 | 0.974 | 0.774 | 0.889 | 0.896 | 0.947 | 0.928 | 0.997 | 0.9798 |
| 377 | 1 | 0.952 | 0.885 | 0.901 | 1 | 0.938 | 0.958 | 0.962 | 1 | 0.95 | 0.97 | 0.97 | 0.974 | 0.774 | 0.89 | 0.897 | 0.947 | 0.928 | 0.997 | 0.9798 |
| 378 | 1 | 0.952 | 0.881 | 0.898 | 1 | 0.938 | 0.958 | 0.962 | 1 | 0.95 | 0.97 | 0.97 | 0.974 | 0.774 | 0.89 | 0.897 | 0.947 | 0.928 | 0.997 | 0.9798 |
| 379 | 1 | 0.952 | 0.881 | 0.898 | 1 | 0.938 | 0.958 | 0.962 | 1 | 0.95 | 0.97 | 0.97 | 0.974 | 0.774 | 0.89 | 0.897 | 0.947 | 0.928 | 0.997 | 0.9798 |
| 380 | 1 | 0.952 | 0.881 | 0.898 | 1 | 0.938 | 0.953 | 0.958 | 1 | 0.95 | 0.97 | 0.97 | 0.974 | 0.774 | 0.889 | 0.896 | 0.947 | 0.928 | 0.997 | 0.9798 |
| 381 | 1 | 0.952 | 0.881 | 0.898 | 1 | 0.938 | 0.953 | 0.958 | 1 | 0.95 | 0.97 | 0.97 | 0.974 | 0.774 | 0.89 | 0.897 | 0.941 | 0.928 | 0.997 | 0.9787 |
| 382 | 1 | 0.952 | 0.881 | 0.898 | 1 | 0.938 | 0.955 | 0.96 | 1 | 0.95 | 0.97 | 0.97 | 0.974 | 0.774 | 0.89 | 0.897 | 0.947 | 0.928 | 0.997 | 0.9798 |
| 383 | 1 | 0.952 | 0.881 | 0.898 | 1 | 0.938 | 0.955 | 0.96 | 1 | 0.95 | 0.97 | 0.97 | 0.974 | 0.774 | 0.89 | 0.897 | 0.947 | 0.928 | 0.997 | 0.9798 |
| 384 | 1 | 0.952 | 0.881 | 0.898 | 1 | 0.938 | 0.955 | 0.96 | 1 | 0.95 | 0.97 | 0.97 | 0.974 | 0.774 | 0.89 | 0.897 | 0.947 | 0.928 | 0.997 | 0.9798 |
| 385 | 1 | 0.952 | 0.881 | 0.898 | 1 | 0.938 | 0.955 | 0.96 | 1 | 0.95 | 0.97 | 0.97 | 0.974 | 0.774 | 0.89 | 0.897 | 0.947 | 0.928 | 0.997 | 0.9798 |
| 386 | 1 | 0.952 | 0.881 | 0.898 | 1 | 0.938 | 0.953 | 0.958 | 1 | 0.95 | 0.97 | 0.97 | 0.974 | 0.774 | 0.89 | 0.897 | 0.931 | 0.928 | 0.997 | 0.9765 |
| 387 | 1 | 0.952 | 0.881 | 0.898 | 1 | 0.938 | 0.953 | 0.958 | 1 | 0.95 | 0.97 | 0.97 | 0.974 | 0.774 | 0.89 | 0.897 | 0.931 | 0.928 | 0.997 | 0.9765 |
| 388 | 1 | 0.952 | 0.881 | 0.898 | 1 | 0.938 | 0.953 | 0.958 | 1 | 0.95 | 0.97 | 0.97 | 0.974 | 0.774 | 0.89 | 0.897 | 0.931 | 0.928 | 0.997 | 0.9765 |
| 389 | 1 | 0.952 | 0.881 | 0.898 | 1 | 0.938 | 0.953 | 0.958 | 1 | 0.95 | 0.97 | 0.97 | 0.974 | 0.774 | 0.89 | 0.897 | 0.931 | 0.928 | 0.997 | 0.9765 |
| 390 | 1 | 0.952 | 0.881 | 0.898 | 1 | 0.938 | 0.953 | 0.958 | 1 | 0.95 | 0.97 | 0.97 | 0.974 | 0.774 | 0.89 | 0.897 | 0.931 | 0.928 | 0.997 | 0.9765 |
| 391 | 1 | 0.952 | 0.881 | 0.898 | 1 | 0.938 | 0.953 | 0.958 | 1 | 0.95 | 0.97 | 0.97 | 0.974 | 0.774 | 0.89 | 0.897 | 0.931 | 0.928 | 0.997 | 0.9765 |
| 392 | 1 | 0.952 | 0.881 | 0.898 | 1 | 0.938 | 0.953 | 0.958 | 1 | 0.95 | 0.97 | 0.97 | 0.974 | 0.774 | 0.89 | 0.897 | 0.931 | 0.928 | 0.997 | 0.9765 |
| 393 | 1 | 0.952 | 0.881 | 0.898 | 1 | 0.938 | 0.953 | 0.958 | 1 | 0.95 | 0.97 | 0.97 | 0.974 | 0.774 | 0.89 | 0.897 | 0.926 | 0.928 | 0.997 | 0.9754 |
| 394 | 1 | 0.952 | 0.881 | 0.898 | 1 | 0.938 | 0.953 | 0.958 | 1 | 0.95 | 0.97 | 0.97 | 0.974 | 0.774 | 0.89 | 0.897 | 0.926 | 0.928 | 0.997 | 0.9754 |
| 395 | 1 | 0.952 | 0.881 | 0.898 | 1 | 0.938 | 0.953 | 0.958 | 1 | 0.95 | 0.97 | 0.97 | 0.974 | 0.774 | 0.89 | 0.897 | 0.926 | 0.928 | 0.997 | 0.9754 |
| 396 | 1 | 0.952 | 0.881 | 0.898 | 1 | 0.938 | 0.95 | 0.957 | 1 | 0.95 | 0.97 | 0.97 | 0.974 | 0.774 | 0.889 | 0.896 | 0.926 | 0.928 | 0.998 | 0.9765 |
| 397 | 1 | 0.952 | 0.885 | 0.901 | 1 | 0.938 | 0.95 | 0.957 | 1 | 0.95 | 0.97 | 0.97 | 0.968 | 0.774 | 0.889 | 0.895 | 0.926 | 0.928 | 0.998 | 0.9765 |
| 398 | 1 | 0.952 | 0.885 | 0.901 | 1 | 0.938 | 0.95 | 0.957 | 1 | 0.95 | 0.97 | 0.97 | 0.968 | 0.774 | 0.888 | 0.894 | 0.926 | 0.94 | 0.998 | 0.9776 |
| 399 | 1 | 0.952 | 0.888 | 0.904 | 1 | 0.938 | 0.953 | 0.958 | 1 | 0.95 | 0.97 | 0.97 | 0.968 | 0.774 | 0.889 | 0.895 | 0.926 | 0.928 | 0.998 | 0.9765 |
| 400 | 1 | 0.952 | 0.885 | 0.901 | 1 | 0.938 | 0.953 | 0.958 | 1 | 0.95 | 0.97 | 0.97 | 0.968 | 0.774 | 0.889 | 0.895 | 0.926 | 0.94 | 0.998 | 0.9776 |
| 401 | 1 | 0.952 | 0.888 | 0.904 | 1 | 0.938 | 0.953 | 0.958 | 1 | 0.95 | 0.97 | 0.97 | 0.968 | 0.774 | 0.893 | 0.899 | 0.92 | 0.94 | 0.998 | 0.9765 |
| 402 | 1 | 0.952 | 0.888 | 0.904 | 1 | 0.938 | 0.955 | 0.96 | 1 | 0.95 | 0.97 | 0.97 | 0.968 | 0.774 | 0.894 | 0.9 | 0.915 | 0.94 | 0.998 | 0.9754 |
| 403 | 1 | 0.952 | 0.892 | 0.906 | 1 | 0.938 | 0.955 | 0.96 | 1 | 0.95 | 0.97 | 0.97 | 0.968 | 0.774 | 0.894 | 0.9 | 0.915 | 0.94 | 0.998 | 0.9754 |
| 404 | 1 | 0.952 | 0.888 | 0.904 | 1 | 0.938 | 0.955 | 0.96 | 1 | 0.95 | 0.97 | 0.97 | 0.968 | 0.774 | 0.894 | 0.9 | 0.915 | 0.94 | 0.998 | 0.9754 |
| 405 | 1 | 0.952 | 0.888 | 0.904 | 1 | 0.938 | 0.955 | 0.96 | 1 | 0.95 | 0.97 | 0.97 | 0.968 | 0.774 | 0.894 | 0.9 | 0.92 | 0.94 | 0.998 | 0.9765 |
| 406 | 1 | 0.952 | 0.888 | 0.904 | 1 | 0.938 | 0.955 | 0.96 | 1 | 0.95 | 0.97 | 0.97 | 0.968 | 0.774 | 0.893 | 0.899 | 0.92 | 0.94 | 0.998 | 0.9765 |
| 407 | 1 | 0.952 | 0.888 | 0.904 | 1 | 0.938 | 0.955 | 0.96 | 1 | 0.95 | 0.97 | 0.97 | 0.968 | 0.774 | 0.894 | 0.9 | 0.92 | 0.94 | 0.998 | 0.9765 |
| 408 | 1 | 0.952 | 0.888 | 0.904 | 1 | 0.938 | 0.955 | 0.96 | 1 | 0.95 | 0.97 | 0.97 | 0.968 | 0.774 | 0.894 | 0.9 | 0.92 | 0.94 | 0.998 | 0.9765 |
| 409 | 1 | 0.952 | 0.888 | 0.904 | 1 | 0.938 | 0.955 | 0.96 | 1 | 0.95 | 0.97 | 0.97 | 0.968 | 0.774 | 0.893 | 0.899 | 0.92 | 0.94 | 0.998 | 0.9765 |
| 410 | 1 | 0.952 | 0.888 | 0.904 | 1 | 0.938 | 0.955 | 0.96 | 1 | 0.95 | 0.97 | 0.97 | 0.968 | 0.774 | 0.894 | 0.9 | 0.926 | 0.94 | 0.998 | 0.9776 |
| 411 | 1 | 0.952 | 0.885 | 0.901 | 1 | 0.938 | 0.958 | 0.962 | 1 | 0.95 | 0.97 | 0.97 | 0.968 | 0.774 | 0.896 | 0.901 | 0.926 | 0.94 | 0.998 | 0.9776 |
| 412 | 1 | 0.952 | 0.888 | 0.904 | 1 | 0.938 | 0.96 | 0.964 | 1 | 0.95 | 0.97 | 0.97 | 0.968 | 0.774 | 0.896 | 0.901 | 0.926 | 0.94 | 0.998 | 0.9776 |
| 413 | 1 | 0.952 | 0.885 | 0.901 | 1 | 0.938 | 0.96 | 0.964 | 1 | 0.95 | 0.97 | 0.97 | 0.968 | 0.774 | 0.896 | 0.901 | 0.926 | 0.94 | 0.998 | 0.9776 |
| 414 | 1 | 0.952 | 0.885 | 0.901 | 1 | 0.938 | 0.96 | 0.964 | 1 | 0.95 | 0.97 | 0.97 | 0.968 | 0.774 | 0.894 | 0.9 | 0.926 | 0.94 | 0.998 | 0.9776 |
| 415 | 1 | 0.952 | 0.885 | 0.901 | 1 | 0.938 | 0.96 | 0.964 | 1 | 0.95 | 0.97 | 0.97 | 0.968 | 0.774 | 0.894 | 0.9 | 0.926 | 0.94 | 0.998 | 0.9776 |
| 416 | 1 | 0.952 | 0.888 | 0.904 | 1 | 0.938 | 0.96 | 0.964 | 1 | 0.95 | 0.97 | 0.97 | 0.968 | 0.774 | 0.894 | 0.9 | 0.926 | 0.94 | 0.998 | 0.9776 |
| 417 | 1 | 0.952 | 0.885 | 0.901 | 1 | 0.938 | 0.96 | 0.964 | 1 | 0.95 | 0.97 | 0.97 | 0.968 | 0.774 | 0.894 | 0.9 | 0.926 | 0.94 | 0.998 | 0.9776 |
| 418 | 1 | 0.952 | 0.885 | 0.901 | 1 | 0.938 | 0.96 | 0.964 | 1 | 0.95 | 0.97 | 0.97 | 0.968 | 0.774 | 0.896 | 0.901 | 0.926 | 0.94 | 0.998 | 0.9776 |
| 419 | 1 | 0.952 | 0.885 | 0.901 | 1 | 0.938 | 0.958 | 0.962 | 1 | 0.95 | 0.97 | 0.97 | 0.968 | 0.774 | 0.896 | 0.901 | 0.926 | 0.94 | 0.998 | 0.9776 |
| 420 | 1 | 0.952 | 0.885 | 0.901 | 1 | 0.938 | 0.958 | 0.962 | 1 | 0.95 | 0.97 | 0.97 | 0.968 | 0.774 | 0.896 | 0.901 | 0.926 | 0.94 | 0.998 | 0.9776 |
| 421 | 1 | 0.952 | 0.885 | 0.901 | 1 | 0.938 | 0.958 | 0.962 | 1 | 0.95 | 0.97 | 0.97 | 0.968 | 0.774 | 0.896 | 0.901 | 0.926 | 0.94 | 0.998 | 0.9776 |
| 422 | 1 | 0.952 | 0.885 | 0.901 | 1 | 0.938 | 0.958 | 0.962 | 1 | 0.95 | 0.975 | 0.974 | 0.968 | 0.774 | 0.896 | 0.901 | 0.915 | 0.94 | 0.998 | 0.9754 |
| 423 | 1 | 0.952 | 0.885 | 0.901 | 1 | 0.938 | 0.958 | 0.962 | 1 | 0.95 | 0.975 | 0.974 | 0.968 | 0.774 | 0.896 | 0.901 | 0.915 | 0.94 | 0.998 | 0.9754 |
| 424 | 1 | 0.952 | 0.885 | 0.901 | 1 | 0.938 | 0.953 | 0.958 | 1 | 0.95 | 0.975 | 0.974 | 0.968 | 0.774 | 0.896 | 0.901 | 0.915 | 0.94 | 0.998 | 0.9754 |
| 425 | 1 | 0.952 | 0.885 | 0.901 | 1 | 0.938 | 0.955 | 0.96 | 1 | 0.95 | 0.975 | 0.974 | 0.968 | 0.774 | 0.896 | 0.901 | 0.915 | 0.94 | 0.998 | 0.9754 |
| 426 | 1 | 0.952 | 0.885 | 0.901 | 1 | 0.938 | 0.955 | 0.96 | 1 | 0.95 | 0.975 | 0.974 | 0.968 | 0.774 | 0.896 | 0.901 | 0.91 | 0.94 | 0.998 | 0.9742 |
| 427 | 1 | 0.952 | 0.885 | 0.901 | 1 | 0.938 | 0.955 | 0.96 | 1 | 0.95 | 0.975 | 0.974 | 0.968 | 0.774 | 0.896 | 0.901 | 0.91 | 0.94 | 0.998 | 0.9742 |
| 428 | 1 | 0.952 | 0.885 | 0.901 | 1 | 0.938 | 0.955 | 0.96 | 1 | 0.95 | 0.975 | 0.974 | 0.968 | 0.774 | 0.896 | 0.901 | 0.91 | 0.94 | 0.998 | 0.9742 |
| 429 | 1 | 0.952 | 0.885 | 0.901 | 1 | 0.938 | 0.953 | 0.958 | 1 | 0.95 | 0.975 | 0.974 | 0.968 | 0.774 | 0.896 | 0.901 | 0.915 | 0.94 | 0.998 | 0.9754 |
| 430 | 1 | 0.952 | 0.885 | 0.901 | 1 | 0.938 | 0.95 | 0.957 | 1 | 0.95 | 0.975 | 0.974 | 0.968 | 0.774 | 0.894 | 0.9 | 0.915 | 0.94 | 0.998 | 0.9754 |
| 431 | 1 | 0.952 | 0.885 | 0.901 | 1 | 0.938 | 0.95 | 0.957 | 1 | 0.95 | 0.975 | 0.974 | 0.968 | 0.774 | 0.894 | 0.9 | 0.915 | 0.94 | 0.998 | 0.9754 |
| 432 | 1 | 0.952 | 0.885 | 0.901 | 1 | 0.938 | 0.95 | 0.957 | 1 | 0.95 | 0.975 | 0.974 | 0.968 | 0.774 | 0.893 | 0.899 | 0.915 | 0.94 | 0.998 | 0.9754 |
| 433 | 1 | 0.952 | 0.881 | 0.898 | 1 | 0.938 | 0.948 | 0.955 | 1 | 0.95 | 0.975 | 0.974 | 0.968 | 0.774 | 0.893 | 0.899 | 0.915 | 0.94 | 0.998 | 0.9754 |
| 434 | 1 | 0.952 | 0.881 | 0.898 | 1 | 0.938 | 0.948 | 0.955 | 0.983 | 0.95 | 0.975 | 0.972 | 0.968 | 0.774 | 0.896 | 0.901 | 0.915 | 0.94 | 0.998 | 0.9754 |
| 435 | 1 | 0.952 | 0.881 | 0.898 | 1 | 0.938 | 0.948 | 0.955 | 0.983 | 0.95 | 0.975 | 0.972 | 0.968 | 0.774 | 0.894 | 0.9 | 0.915 | 0.94 | 0.998 | 0.9754 |
| 436 | 1 | 0.952 | 0.881 | 0.898 | 1 | 0.938 | 0.948 | 0.955 | 0.983 | 0.95 | 0.975 | 0.972 | 0.968 | 0.774 | 0.894 | 0.9 | 0.915 | 0.94 | 0.998 | 0.9754 |
| 437 | 1 | 0.952 | 0.881 | 0.898 | 1 | 0.938 | 0.948 | 0.955 | 0.983 | 0.95 | 0.975 | 0.972 | 0.968 | 0.774 | 0.893 | 0.899 | 0.915 | 0.94 | 0.998 | 0.9754 |
| 438 | 1 | 0.952 | 0.881 | 0.898 | 1 | 0.938 | 0.948 | 0.955 | 0.983 | 0.95 | 0.975 | 0.972 | 0.968 | 0.774 | 0.893 | 0.899 | 0.915 | 0.94 | 0.998 | 0.9754 |
| 439 | 1 | 0.952 | 0.881 | 0.898 | 1 | 0.938 | 0.948 | 0.955 | 0.983 | 0.95 | 0.975 | 0.972 | 0.968 | 0.774 | 0.893 | 0.899 | 0.915 | 0.94 | 0.998 | 0.9754 |
| 440 | 1 | 0.952 | 0.881 | 0.898 | 1 | 0.938 | 0.948 | 0.955 | 0.983 | 0.95 | 0.975 | 0.972 | 0.968 | 0.774 | 0.893 | 0.899 | 0.915 | 0.94 | 0.998 | 0.9754 |
| 441 | 1 | 0.952 | 0.881 | 0.898 | 1 | 0.938 | 0.948 | 0.955 | 0.983 | 0.95 | 0.975 | 0.972 | 0.968 | 0.774 | 0.893 | 0.899 | 0.915 | 0.94 | 0.998 | 0.9754 |
| 442 | 1 | 0.952 | 0.881 | 0.898 | 1 | 0.938 | 0.948 | 0.955 | 0.983 | 0.95 | 0.975 | 0.972 | 0.968 | 0.774 | 0.893 | 0.899 | 0.92 | 0.94 | 0.998 | 0.9765 |
| 443 | 1 | 0.952 | 0.881 | 0.898 | 1 | 0.938 | 0.948 | 0.955 | 0.983 | 0.95 | 0.975 | 0.972 | 0.968 | 0.774 | 0.894 | 0.9 | 0.92 | 0.94 | 0.998 | 0.9765 |
| 444 | 1 | 0.952 | 0.875 | 0.893 | 1 | 0.938 | 0.948 | 0.955 | 1 | 0.95 | 0.975 | 0.974 | 0.968 | 0.774 | 0.893 | 0.899 | 0.915 | 0.94 | 0.998 | 0.9754 |
| 445 | 1 | 0.952 | 0.875 | 0.893 | 1 | 0.938 | 0.948 | 0.955 | 1 | 0.95 | 0.975 | 0.974 | 0.968 | 0.774 | 0.893 | 0.899 | 0.915 | 0.94 | 0.998 | 0.9754 |
| 446 | 1 | 0.952 | 0.871 | 0.89 | 1 | 0.938 | 0.948 | 0.955 | 1 | 0.95 | 0.975 | 0.974 | 0.968 | 0.774 | 0.892 | 0.897 | 0.915 | 0.94 | 0.998 | 0.9754 |
| 447 | 1 | 0.952 | 0.871 | 0.89 | 1 | 0.938 | 0.948 | 0.955 | 0.983 | 0.95 | 0.975 | 0.972 | 0.968 | 0.774 | 0.892 | 0.897 | 0.915 | 0.94 | 0.998 | 0.9754 |
| 448 | 1 | 0.952 | 0.871 | 0.89 | 1 | 0.938 | 0.948 | 0.955 | 0.983 | 0.95 | 0.975 | 0.972 | 0.968 | 0.774 | 0.892 | 0.897 | 0.915 | 0.94 | 0.998 | 0.9754 |
| 449 | 1 | 0.952 | 0.875 | 0.893 | 1 | 0.938 | 0.948 | 0.955 | 1 | 0.95 | 0.975 | 0.974 | 0.968 | 0.774 | 0.893 | 0.899 | 0.915 | 0.94 | 0.998 | 0.9754 |
| 450 | 1 | 0.952 | 0.878 | 0.895 | 1 | 0.938 | 0.948 | 0.955 | 1 | 0.95 | 0.975 | 0.974 | 0.968 | 0.774 | 0.893 | 0.899 | 0.92 | 0.94 | 0.998 | 0.9765 |
| 451 | 1 | 0.952 | 0.881 | 0.898 | 1 | 0.938 | 0.948 | 0.955 | 1 | 0.95 | 0.975 | 0.974 | 0.968 | 0.774 | 0.893 | 0.899 | 0.915 | 0.94 | 0.998 | 0.9754 |
| 452 | 1 | 0.952 | 0.881 | 0.898 | 1 | 0.938 | 0.95 | 0.957 | 1 | 0.95 | 0.975 | 0.974 | 0.968 | 0.774 | 0.893 | 0.899 | 0.915 | 0.94 | 0.998 | 0.9754 |
| 453 | 1 | 0.952 | 0.881 | 0.898 | 1 | 0.938 | 0.95 | 0.957 | 1 | 0.95 | 0.975 | 0.974 | 0.968 | 0.774 | 0.893 | 0.899 | 0.915 | 0.94 | 0.998 | 0.9754 |
| 454 | 1 | 0.952 | 0.875 | 0.893 | 1 | 0.938 | 0.948 | 0.955 | 1 | 0.95 | 0.975 | 0.974 | 0.968 | 0.774 | 0.893 | 0.899 | 0.915 | 0.94 | 0.998 | 0.9754 |
| 455 | 1 | 0.952 | 0.875 | 0.893 | 1 | 0.938 | 0.945 | 0.953 | 1 | 0.95 | 0.975 | 0.974 | 0.968 | 0.774 | 0.892 | 0.897 | 0.915 | 0.94 | 0.998 | 0.9754 |
| 456 | 1 | 0.952 | 0.871 | 0.89 | 1 | 0.938 | 0.945 | 0.953 | 1 | 0.95 | 0.975 | 0.974 | 0.968 | 0.774 | 0.892 | 0.897 | 0.915 | 0.94 | 0.998 | 0.9754 |
| 457 | 1 | 0.952 | 0.871 | 0.89 | 1 | 0.938 | 0.945 | 0.953 | 1 | 0.95 | 0.975 | 0.974 | 0.968 | 0.774 | 0.89 | 0.896 | 0.915 | 0.94 | 0.998 | 0.9754 |
| 458 | 1 | 0.952 | 0.871 | 0.89 | 1 | 0.938 | 0.945 | 0.953 | 1 | 0.95 | 0.975 | 0.974 | 0.968 | 0.774 | 0.89 | 0.896 | 0.915 | 0.94 | 0.998 | 0.9754 |
| 459 | 1 | 0.952 | 0.871 | 0.89 | 1 | 0.938 | 0.945 | 0.953 | 1 | 0.95 | 0.975 | 0.974 | 0.968 | 0.774 | 0.89 | 0.896 | 0.915 | 0.94 | 0.998 | 0.9754 |
| 460 | 1 | 0.952 | 0.871 | 0.89 | 1 | 0.938 | 0.945 | 0.953 | 1 | 0.95 | 0.975 | 0.974 | 0.968 | 0.774 | 0.89 | 0.896 | 0.915 | 0.94 | 0.998 | 0.9754 |
| 461 | 1 | 0.952 | 0.868 | 0.887 | 1 | 0.938 | 0.945 | 0.953 | 1 | 0.95 | 0.975 | 0.974 | 0.968 | 0.774 | 0.89 | 0.896 | 0.915 | 0.94 | 0.998 | 0.9754 |
| 462 | 1 | 0.952 | 0.868 | 0.887 | 1 | 0.938 | 0.945 | 0.953 | 1 | 0.95 | 0.975 | 0.974 | 0.968 | 0.774 | 0.89 | 0.896 | 0.915 | 0.94 | 0.998 | 0.9754 |
| 463 | 1 | 0.952 | 0.871 | 0.89 | 1 | 0.938 | 0.945 | 0.953 | 1 | 0.95 | 0.975 | 0.974 | 0.968 | 0.774 | 0.89 | 0.896 | 0.915 | 0.94 | 0.998 | 0.9754 |
| 464 | 1 | 0.952 | 0.868 | 0.887 | 1 | 0.938 | 0.945 | 0.953 | 1 | 0.95 | 0.975 | 0.974 | 0.968 | 0.774 | 0.89 | 0.896 | 0.915 | 0.928 | 0.998 | 0.9742 |
| 465 | 1 | 0.952 | 0.871 | 0.89 | 1 | 0.938 | 0.945 | 0.953 | 1 | 0.95 | 0.975 | 0.974 | 0.968 | 0.774 | 0.89 | 0.896 | 0.915 | 0.94 | 0.998 | 0.9754 |
| 466 | 1 | 0.952 | 0.871 | 0.89 | 1 | 0.938 | 0.945 | 0.953 | 0.983 | 0.95 | 0.975 | 0.972 | 0.968 | 0.774 | 0.89 | 0.896 | 0.915 | 0.94 | 0.998 | 0.9754 |
| 467 | 1 | 0.952 | 0.871 | 0.89 | 1 | 0.938 | 0.945 | 0.953 | 0.983 | 0.95 | 0.975 | 0.972 | 0.968 | 0.774 | 0.89 | 0.896 | 0.915 | 0.94 | 0.998 | 0.9754 |
| 468 | 1 | 0.952 | 0.875 | 0.893 | 1 | 0.938 | 0.945 | 0.953 | 0.983 | 0.95 | 0.975 | 0.972 | 0.968 | 0.774 | 0.89 | 0.896 | 0.915 | 0.94 | 0.998 | 0.9754 |
| 469 | 1 | 0.952 | 0.875 | 0.893 | 1 | 0.938 | 0.945 | 0.953 | 0.983 | 0.95 | 0.975 | 0.972 | 0.968 | 0.774 | 0.89 | 0.896 | 0.915 | 0.94 | 0.998 | 0.9754 |
| 470 | 1 | 0.952 | 0.875 | 0.893 | 1 | 0.938 | 0.945 | 0.953 | 0.983 | 0.95 | 0.975 | 0.972 | 0.968 | 0.774 | 0.89 | 0.896 | 0.915 | 0.94 | 0.998 | 0.9754 |
| 471 | 1 | 0.952 | 0.875 | 0.893 | 1 | 0.938 | 0.945 | 0.953 | 0.983 | 0.95 | 0.975 | 0.972 | 0.968 | 0.774 | 0.889 | 0.895 | 0.915 | 0.94 | 0.998 | 0.9754 |
| 472 | 1 | 0.952 | 0.875 | 0.893 | 1 | 0.938 | 0.945 | 0.953 | 0.983 | 0.95 | 0.975 | 0.972 | 0.968 | 0.774 | 0.888 | 0.894 | 0.915 | 0.94 | 0.998 | 0.9754 |
| 473 | 1 | 0.952 | 0.875 | 0.893 | 1 | 0.938 | 0.945 | 0.953 | 0.983 | 0.95 | 0.975 | 0.972 | 0.968 | 0.774 | 0.889 | 0.895 | 0.915 | 0.94 | 0.998 | 0.9754 |
| 474 | 1 | 0.952 | 0.875 | 0.893 | 1 | 0.938 | 0.945 | 0.953 | 0.983 | 0.95 | 0.975 | 0.972 | 0.968 | 0.774 | 0.888 | 0.894 | 0.915 | 0.94 | 0.998 | 0.9754 |
| 475 | 1 | 0.952 | 0.875 | 0.893 | 1 | 0.938 | 0.943 | 0.951 | 0.983 | 0.95 | 0.975 | 0.972 | 0.968 | 0.774 | 0.888 | 0.894 | 0.915 | 0.94 | 0.998 | 0.9754 |
| 476 | 1 | 0.952 | 0.868 | 0.887 | 1 | 0.938 | 0.943 | 0.951 | 0.983 | 0.95 | 0.975 | 0.972 | 0.968 | 0.774 | 0.888 | 0.894 | 0.915 | 0.94 | 0.998 | 0.9754 |
| 477 | 1 | 0.952 | 0.868 | 0.887 | 1 | 0.938 | 0.943 | 0.951 | 0.983 | 0.95 | 0.975 | 0.972 | 0.968 | 0.774 | 0.888 | 0.894 | 0.915 | 0.94 | 0.998 | 0.9754 |
| 478 | 1 | 0.952 | 0.868 | 0.887 | 1 | 0.938 | 0.943 | 0.951 | 0.983 | 0.95 | 0.975 | 0.972 | 0.968 | 0.774 | 0.888 | 0.894 | 0.915 | 0.94 | 0.998 | 0.9754 |
| 479 | 1 | 0.952 | 0.875 | 0.893 | 1 | 0.938 | 0.943 | 0.951 | 0.983 | 0.95 | 0.975 | 0.972 | 0.968 | 0.774 | 0.888 | 0.894 | 0.91 | 0.94 | 0.998 | 0.9742 |
| 480 | 1 | 0.952 | 0.875 | 0.893 | 1 | 0.938 | 0.943 | 0.951 | 0.983 | 0.95 | 0.975 | 0.972 | 0.968 | 0.774 | 0.888 | 0.894 | 0.91 | 0.94 | 0.998 | 0.9742 |
| 481 | 1 | 0.952 | 0.875 | 0.893 | 1 | 0.938 | 0.943 | 0.951 | 0.983 | 0.95 | 0.975 | 0.972 | 0.968 | 0.774 | 0.888 | 0.894 | 0.91 | 0.94 | 0.998 | 0.9742 |
| 482 | 1 | 0.952 | 0.875 | 0.893 | 1 | 0.938 | 0.943 | 0.951 | 0.983 | 0.95 | 0.975 | 0.972 | 0.968 | 0.774 | 0.888 | 0.894 | 0.91 | 0.94 | 0.998 | 0.9742 |
| 483 | 1 | 0.952 | 0.875 | 0.893 | 1 | 0.938 | 0.948 | 0.955 | 0.983 | 0.95 | 0.975 | 0.972 | 0.968 | 0.774 | 0.888 | 0.894 | 0.91 | 0.94 | 0.998 | 0.9742 |
| 484 | 1 | 0.952 | 0.875 | 0.893 | 1 | 0.938 | 0.943 | 0.951 | 0.983 | 0.95 | 0.975 | 0.972 | 0.968 | 0.774 | 0.888 | 0.894 | 0.904 | 0.94 | 0.998 | 0.9731 |
| 485 | 1 | 0.952 | 0.875 | 0.893 | 1 | 0.938 | 0.943 | 0.951 | 0.983 | 0.95 | 0.975 | 0.972 | 0.968 | 0.774 | 0.888 | 0.894 | 0.904 | 0.94 | 0.998 | 0.9731 |
| 486 | 1 | 0.952 | 0.875 | 0.893 | 1 | 0.938 | 0.945 | 0.953 | 0.983 | 0.95 | 0.975 | 0.972 | 0.968 | 0.774 | 0.889 | 0.895 | 0.899 | 0.94 | 0.998 | 0.972 |
| 487 | 1 | 0.952 | 0.875 | 0.893 | 1 | 0.938 | 0.945 | 0.953 | 0.983 | 0.95 | 0.975 | 0.972 | 0.968 | 0.774 | 0.889 | 0.895 | 0.899 | 0.94 | 0.998 | 0.972 |
| 488 | 1 | 0.952 | 0.875 | 0.893 | 1 | 0.938 | 0.945 | 0.953 | 0.983 | 0.95 | 0.975 | 0.972 | 0.968 | 0.774 | 0.888 | 0.894 | 0.899 | 0.94 | 0.998 | 0.972 |
| 489 | 1 | 0.952 | 0.875 | 0.893 | 1 | 0.938 | 0.945 | 0.953 | 0.983 | 0.95 | 0.975 | 0.972 | 0.968 | 0.774 | 0.89 | 0.896 | 0.894 | 0.94 | 0.998 | 0.9709 |
| 490 | 1 | 0.952 | 0.875 | 0.893 | 1 | 0.938 | 0.945 | 0.953 | 0.983 | 0.95 | 0.975 | 0.972 | 0.968 | 0.774 | 0.89 | 0.896 | 0.894 | 0.94 | 0.998 | 0.9709 |
| 491 | 1 | 0.952 | 0.875 | 0.893 | 1 | 0.938 | 0.945 | 0.953 | 0.983 | 0.95 | 0.975 | 0.972 | 0.968 | 0.774 | 0.889 | 0.895 | 0.888 | 0.94 | 0.998 | 0.9698 |
| 492 | 1 | 0.952 | 0.875 | 0.893 | 1 | 0.938 | 0.945 | 0.953 | 0.983 | 0.95 | 0.975 | 0.972 | 0.968 | 0.774 | 0.889 | 0.895 | 0.894 | 0.94 | 0.998 | 0.9709 |
| 493 | 1 | 0.952 | 0.875 | 0.893 | 1 | 0.938 | 0.948 | 0.955 | 0.983 | 0.95 | 0.975 | 0.972 | 0.968 | 0.774 | 0.892 | 0.897 | 0.894 | 0.94 | 0.998 | 0.9709 |
| 494 | 1 | 0.952 | 0.875 | 0.893 | 1 | 0.938 | 0.948 | 0.955 | 0.983 | 0.95 | 0.975 | 0.972 | 0.968 | 0.774 | 0.892 | 0.897 | 0.894 | 0.94 | 0.998 | 0.9709 |
| 495 | 1 | 0.952 | 0.875 | 0.893 | 1 | 0.938 | 0.948 | 0.955 | 0.983 | 0.95 | 0.975 | 0.972 | 0.968 | 0.774 | 0.89 | 0.896 | 0.91 | 0.94 | 0.998 | 0.9742 |
| 496 | 1 | 0.952 | 0.875 | 0.893 | 1 | 0.938 | 0.948 | 0.955 | 0.983 | 0.95 | 0.975 | 0.972 | 0.968 | 0.774 | 0.89 | 0.896 | 0.904 | 0.94 | 0.998 | 0.9731 |
| 497 | 1 | 0.952 | 0.875 | 0.893 | 1 | 0.938 | 0.948 | 0.955 | 0.983 | 0.95 | 0.975 | 0.972 | 0.968 | 0.774 | 0.89 | 0.896 | 0.904 | 0.94 | 0.998 | 0.9731 |
| 498 | 1 | 0.952 | 0.875 | 0.893 | 1 | 0.938 | 0.948 | 0.955 | 0.983 | 0.95 | 0.975 | 0.972 | 0.968 | 0.774 | 0.886 | 0.893 | 0.91 | 0.94 | 0.998 | 0.9742 |
| 499 | 1 | 0.952 | 0.875 | 0.893 | 1 | 0.938 | 0.94 | 0.949 | 0.983 | 0.95 | 0.975 | 0.972 | 0.968 | 0.774 | 0.886 | 0.893 | 0.91 | 0.94 | 0.998 | 0.9742 |
| 500 | 1 | 0.952 | 0.875 | 0.893 | 1 | 0.938 | 0.943 | 0.951 | 0.983 | 0.95 | 0.975 | 0.972 | 0.968 | 0.774 | 0.886 | 0.893 | 0.91 | 0.94 | 0.998 | 0.9742 |

1. The dataset of H0351.1016 was selected as the training dataset

| Number of features | H0351.1009 | | | | H0351.1012 | | | | H0351.1015 | | | | H0351.2001 | | | | H0351.2002 | | | |
| --- | --- | --- | --- | --- | --- | --- | --- | --- | --- | --- | --- | --- | --- | --- | --- | --- | --- | --- | --- | --- |
|  | class1 | class2 | class3 | total | class1 | class2 | class3 | total | class1 | class2 | class3 | total | class1 | class2 | class3 | total | class1 | class2 | class3 | total |
| 4 | 1 | 0.952 | 0.997 | 0.992 | 0.988 | 0.917 | 0.998 | 0.989 | 0.899 | 0.968 | 1 | 0.979 | 0.5 | 0.868 | 1 | 0.911 | 0.803 | 0.904 | 1 | 0.9496 |
| 5 | 1 | 0.976 | 1 | 0.997 | 0.988 | 0.917 | 0.998 | 0.989 | 0.899 | 0.968 | 1 | 0.979 | 0.669 | 0.792 | 1 | 0.934 | 0.622 | 0.916 | 1 | 0.9127 |
| 6 | 1 | 0.976 | 0.997 | 0.994 | 0.988 | 0.917 | 0.998 | 0.989 | 0.949 | 0.968 | 0.997 | 0.985 | 0.805 | 0.849 | 0.995 | 0.956 | 0.601 | 0.94 | 0.998 | 0.9093 |
| 7 | 1 | 0.952 | 0.997 | 0.992 | 0.988 | 0.917 | 0.998 | 0.989 | 0.949 | 0.968 | 0.997 | 0.985 | 0.89 | 0.774 | 0.995 | 0.965 | 0.676 | 0.916 | 0.998 | 0.9227 |
| 8 | 1 | 0.952 | 0.997 | 0.992 | 0.988 | 0.917 | 0.998 | 0.989 | 0.924 | 0.968 | 0.997 | 0.981 | 0.935 | 0.774 | 0.995 | 0.973 | 0.734 | 0.916 | 0.998 | 0.9351 |
| 9 | 1 | 0.952 | 0.997 | 0.992 | 0.988 | 0.958 | 0.998 | 0.992 | 0.937 | 1 | 0.997 | 0.987 | 0.929 | 1 | 0.993 | 0.983 | 0.761 | 1 | 0.998 | 0.9485 |
| 10 | 1 | 0.952 | 0.997 | 0.992 | 0.988 | 0.958 | 0.998 | 0.992 | 0.937 | 1 | 0.997 | 0.987 | 0.903 | 1 | 0.997 | 0.982 | 0.676 | 1 | 1 | 0.9317 |
| 11 | 1 | 0.976 | 0.997 | 0.994 | 0.975 | 0.938 | 0.995 | 0.987 | 0.937 | 0.984 | 0.997 | 0.985 | 0.916 | 0.981 | 0.996 | 0.982 | 0.723 | 1 | 1 | 0.9418 |
| 12 | 1 | 0.976 | 0.997 | 0.994 | 0.988 | 0.938 | 0.998 | 0.991 | 0.924 | 1 | 0.997 | 0.985 | 0.922 | 0.981 | 0.995 | 0.982 | 0.686 | 1 | 1 | 0.9339 |
| 13 | 1 | 0.976 | 0.997 | 0.994 | 0.988 | 0.917 | 0.998 | 0.989 | 0.937 | 0.984 | 0.997 | 0.985 | 0.916 | 0.943 | 0.995 | 0.979 | 0.729 | 1 | 1 | 0.9429 |
| 14 | 1 | 0.976 | 0.993 | 0.992 | 0.988 | 0.979 | 0.998 | 0.994 | 0.937 | 1 | 0.997 | 0.987 | 0.942 | 1 | 0.993 | 0.985 | 0.798 | 1 | 1 | 0.9574 |
| 15 | 1 | 0.976 | 0.993 | 0.992 | 0.988 | 0.979 | 0.998 | 0.994 | 0.937 | 1 | 0.997 | 0.987 | 0.942 | 1 | 0.993 | 0.985 | 0.798 | 1 | 1 | 0.9574 |
| 16 | 1 | 0.976 | 0.997 | 0.994 | 0.988 | 0.979 | 0.998 | 0.994 | 0.975 | 1 | 0.997 | 0.994 | 0.916 | 1 | 0.997 | 0.984 | 0.809 | 1 | 1 | 0.9597 |
| 17 | 1 | 0.976 | 0.997 | 0.994 | 0.988 | 0.979 | 0.998 | 0.994 | 0.975 | 1 | 0.997 | 0.994 | 0.929 | 1 | 0.997 | 0.986 | 0.798 | 1 | 1 | 0.9574 |
| 18 | 1 | 0.976 | 0.997 | 0.994 | 0.988 | 0.979 | 0.998 | 0.994 | 0.987 | 1 | 1 | 0.998 | 0.922 | 1 | 0.997 | 0.985 | 0.819 | 1 | 1 | 0.9619 |
| 19 | 1 | 0.976 | 0.997 | 0.994 | 0.988 | 0.979 | 0.998 | 0.994 | 1 | 1 | 1 | 1 | 0.896 | 1 | 0.997 | 0.981 | 0.835 | 1 | 1 | 0.9653 |
| 20 | 1 | 0.976 | 0.997 | 0.994 | 1 | 0.979 | 0.998 | 0.996 | 1 | 1 | 1 | 1 | 0.903 | 1 | 0.997 | 0.982 | 0.83 | 1 | 1 | 0.9642 |
| 21 | 1 | 0.976 | 0.997 | 0.994 | 0.988 | 0.979 | 0.998 | 0.994 | 1 | 1 | 1 | 1 | 0.909 | 1 | 0.995 | 0.981 | 0.824 | 1 | 1 | 0.963 |
| 22 | 1 | 0.976 | 0.997 | 0.994 | 1 | 0.979 | 0.998 | 0.996 | 1 | 1 | 1 | 1 | 0.916 | 1 | 0.993 | 0.981 | 0.846 | 1 | 1 | 0.9675 |
| 23 | 1 | 0.976 | 0.997 | 0.994 | 1 | 0.979 | 0.998 | 0.996 | 1 | 1 | 1 | 1 | 0.929 | 1 | 0.993 | 0.983 | 0.851 | 1 | 1 | 0.9686 |
| 24 | 1 | 0.976 | 0.993 | 0.992 | 1 | 0.979 | 0.998 | 0.996 | 1 | 1 | 1 | 1 | 0.935 | 1 | 0.993 | 0.984 | 0.851 | 1 | 1 | 0.9686 |
| 25 | 1 | 0.976 | 0.993 | 0.992 | 1 | 0.979 | 0.998 | 0.996 | 1 | 1 | 1 | 1 | 0.942 | 1 | 0.993 | 0.985 | 0.851 | 1 | 1 | 0.9686 |
| 26 | 1 | 0.976 | 0.997 | 0.994 | 1 | 0.979 | 0.993 | 0.992 | 1 | 1 | 1 | 1 | 0.929 | 1 | 0.993 | 0.983 | 0.83 | 1 | 1 | 0.9642 |
| 27 | 1 | 0.976 | 0.997 | 0.994 | 1 | 0.979 | 0.993 | 0.992 | 1 | 1 | 1 | 1 | 0.929 | 1 | 0.993 | 0.983 | 0.83 | 1 | 1 | 0.9642 |
| 28 | 1 | 0.976 | 0.997 | 0.994 | 1 | 0.979 | 0.993 | 0.992 | 1 | 1 | 1 | 1 | 0.929 | 1 | 0.993 | 0.983 | 0.83 | 1 | 1 | 0.9642 |
| 29 | 1 | 0.976 | 0.997 | 0.994 | 1 | 0.979 | 0.995 | 0.994 | 1 | 1 | 1 | 1 | 0.922 | 1 | 0.993 | 0.982 | 0.824 | 1 | 1 | 0.963 |
| 30 | 1 | 0.976 | 0.993 | 0.992 | 1 | 0.979 | 0.993 | 0.992 | 1 | 1 | 1 | 1 | 0.929 | 1 | 0.993 | 0.983 | 0.846 | 1 | 1 | 0.9675 |
| 31 | 1 | 0.976 | 0.997 | 0.994 | 1 | 0.979 | 0.995 | 0.994 | 1 | 1 | 1 | 1 | 0.922 | 1 | 0.993 | 0.982 | 0.846 | 1 | 1 | 0.9675 |
| 32 | 1 | 0.976 | 0.997 | 0.994 | 1 | 0.979 | 0.995 | 0.994 | 1 | 1 | 1 | 1 | 0.922 | 1 | 0.993 | 0.982 | 0.846 | 1 | 1 | 0.9675 |
| 33 | 1 | 0.976 | 0.997 | 0.994 | 1 | 0.979 | 0.995 | 0.994 | 1 | 1 | 1 | 1 | 0.922 | 1 | 0.993 | 0.982 | 0.846 | 1 | 1 | 0.9675 |
| 34 | 1 | 0.976 | 0.997 | 0.994 | 1 | 0.979 | 0.995 | 0.994 | 1 | 1 | 1 | 1 | 0.922 | 1 | 0.993 | 0.982 | 0.851 | 1 | 1 | 0.9686 |
| 35 | 1 | 0.976 | 0.997 | 0.994 | 1 | 0.979 | 0.995 | 0.994 | 1 | 1 | 1 | 1 | 0.922 | 1 | 0.993 | 0.982 | 0.846 | 1 | 1 | 0.9675 |
| 36 | 1 | 0.976 | 0.997 | 0.994 | 1 | 0.979 | 0.995 | 0.994 | 1 | 1 | 1 | 1 | 0.922 | 1 | 0.993 | 0.982 | 0.851 | 1 | 1 | 0.9686 |
| 37 | 1 | 0.976 | 0.997 | 0.994 | 1 | 0.979 | 0.995 | 0.994 | 1 | 1 | 1 | 1 | 0.922 | 1 | 0.993 | 0.982 | 0.846 | 1 | 1 | 0.9675 |
| 38 | 1 | 0.976 | 1 | 0.997 | 1 | 0.979 | 0.998 | 0.996 | 1 | 1 | 1 | 1 | 0.922 | 1 | 0.993 | 0.982 | 0.856 | 1 | 1 | 0.9698 |
| 39 | 1 | 0.976 | 1 | 0.997 | 1 | 0.979 | 0.998 | 0.996 | 1 | 1 | 1 | 1 | 0.922 | 1 | 0.993 | 0.982 | 0.851 | 1 | 1 | 0.9686 |
| 40 | 1 | 0.976 | 1 | 0.997 | 1 | 0.979 | 0.995 | 0.994 | 1 | 1 | 1 | 1 | 0.922 | 1 | 0.993 | 0.982 | 0.856 | 1 | 1 | 0.9698 |
| 41 | 1 | 0.976 | 1 | 0.997 | 1 | 0.979 | 0.993 | 0.992 | 1 | 1 | 1 | 1 | 0.922 | 1 | 0.993 | 0.982 | 0.84 | 1 | 1 | 0.9664 |
| 42 | 1 | 0.976 | 1 | 0.997 | 1 | 0.979 | 0.995 | 0.994 | 1 | 1 | 1 | 1 | 0.922 | 1 | 0.993 | 0.982 | 0.824 | 1 | 1 | 0.963 |
| 43 | 1 | 0.976 | 1 | 0.997 | 1 | 0.979 | 0.995 | 0.994 | 1 | 1 | 1 | 1 | 0.922 | 1 | 0.992 | 0.981 | 0.824 | 1 | 1 | 0.963 |
| 44 | 1 | 0.976 | 1 | 0.997 | 1 | 0.979 | 0.995 | 0.994 | 1 | 1 | 1 | 1 | 0.922 | 1 | 0.991 | 0.98 | 0.835 | 1 | 1 | 0.9653 |
| 45 | 1 | 0.976 | 0.997 | 0.994 | 1 | 0.979 | 0.995 | 0.994 | 1 | 1 | 1 | 1 | 0.929 | 1 | 0.991 | 0.981 | 0.835 | 1 | 1 | 0.9653 |
| 46 | 1 | 0.976 | 1 | 0.997 | 1 | 0.979 | 0.995 | 0.994 | 1 | 1 | 1 | 1 | 0.929 | 1 | 0.989 | 0.98 | 0.835 | 1 | 1 | 0.9653 |
| 47 | 1 | 0.976 | 1 | 0.997 | 1 | 0.979 | 0.995 | 0.994 | 1 | 1 | 1 | 1 | 0.929 | 1 | 0.989 | 0.98 | 0.83 | 1 | 1 | 0.9642 |
| 48 | 1 | 0.976 | 1 | 0.997 | 1 | 0.979 | 0.993 | 0.992 | 0.987 | 1 | 1 | 0.998 | 0.922 | 1 | 0.986 | 0.977 | 0.809 | 1 | 1 | 0.9597 |
| 49 | 1 | 0.976 | 1 | 0.997 | 1 | 0.979 | 0.993 | 0.992 | 0.975 | 1 | 1 | 0.996 | 0.922 | 1 | 0.986 | 0.977 | 0.809 | 1 | 1 | 0.9597 |
| 50 | 1 | 0.976 | 1 | 0.997 | 1 | 0.979 | 0.993 | 0.992 | 1 | 1 | 1 | 1 | 0.922 | 1 | 0.992 | 0.981 | 0.803 | 1 | 1 | 0.9586 |
| 51 | 1 | 0.976 | 1 | 0.997 | 1 | 0.979 | 0.993 | 0.992 | 1 | 1 | 1 | 1 | 0.922 | 1 | 0.992 | 0.981 | 0.809 | 1 | 1 | 0.9597 |
| 52 | 1 | 0.976 | 1 | 0.997 | 1 | 0.979 | 0.993 | 0.992 | 1 | 1 | 1 | 1 | 0.922 | 1 | 0.992 | 0.981 | 0.803 | 1 | 1 | 0.9586 |
| 53 | 1 | 0.976 | 1 | 0.997 | 1 | 0.979 | 0.993 | 0.992 | 1 | 1 | 1 | 1 | 0.916 | 1 | 0.992 | 0.98 | 0.809 | 1 | 1 | 0.9597 |
| 54 | 1 | 0.976 | 1 | 0.997 | 1 | 0.979 | 0.993 | 0.992 | 1 | 1 | 1 | 1 | 0.922 | 1 | 0.991 | 0.98 | 0.809 | 1 | 1 | 0.9597 |
| 55 | 1 | 0.976 | 1 | 0.997 | 1 | 0.979 | 0.993 | 0.992 | 1 | 1 | 1 | 1 | 0.922 | 1 | 0.992 | 0.981 | 0.803 | 1 | 1 | 0.9586 |
| 56 | 1 | 0.976 | 1 | 0.997 | 1 | 0.979 | 0.993 | 0.992 | 1 | 1 | 1 | 1 | 0.922 | 1 | 0.992 | 0.981 | 0.803 | 1 | 1 | 0.9586 |
| 57 | 1 | 0.976 | 1 | 0.997 | 1 | 0.979 | 0.993 | 0.992 | 1 | 1 | 1 | 1 | 0.922 | 1 | 0.992 | 0.981 | 0.803 | 1 | 1 | 0.9586 |
| 58 | 1 | 0.976 | 1 | 0.997 | 1 | 0.979 | 0.993 | 0.992 | 1 | 1 | 1 | 1 | 0.922 | 1 | 0.992 | 0.981 | 0.809 | 1 | 1 | 0.9597 |
| 59 | 1 | 0.976 | 1 | 0.997 | 1 | 0.979 | 0.993 | 0.992 | 1 | 1 | 1 | 1 | 0.922 | 1 | 0.992 | 0.981 | 0.809 | 1 | 1 | 0.9597 |
| 60 | 1 | 0.976 | 1 | 0.997 | 1 | 0.979 | 0.993 | 0.992 | 1 | 1 | 1 | 1 | 0.916 | 1 | 0.992 | 0.98 | 0.809 | 1 | 1 | 0.9597 |
| 61 | 1 | 0.976 | 1 | 0.997 | 1 | 0.979 | 0.993 | 0.992 | 1 | 1 | 1 | 1 | 0.916 | 1 | 0.992 | 0.98 | 0.809 | 1 | 1 | 0.9597 |
| 62 | 1 | 1 | 1 | 1 | 1 | 0.979 | 0.993 | 0.992 | 1 | 1 | 1 | 1 | 0.916 | 1 | 0.991 | 0.979 | 0.809 | 1 | 1 | 0.9597 |
| 63 | 1 | 1 | 1 | 1 | 1 | 0.979 | 0.993 | 0.992 | 1 | 1 | 1 | 1 | 0.916 | 1 | 0.991 | 0.979 | 0.809 | 1 | 1 | 0.9597 |
| 64 | 1 | 1 | 1 | 1 | 1 | 0.979 | 0.993 | 0.992 | 1 | 1 | 1 | 1 | 0.916 | 1 | 0.991 | 0.979 | 0.824 | 1 | 1 | 0.963 |
| 65 | 1 | 1 | 1 | 1 | 1 | 0.979 | 0.988 | 0.989 | 1 | 1 | 0.997 | 0.998 | 0.942 | 1 | 0.985 | 0.979 | 0.867 | 1 | 1 | 0.972 |
| 66 | 1 | 1 | 1 | 1 | 1 | 0.979 | 0.988 | 0.989 | 1 | 1 | 0.997 | 0.998 | 0.929 | 1 | 0.985 | 0.977 | 0.867 | 1 | 1 | 0.972 |
| 67 | 1 | 0.976 | 0.997 | 0.994 | 1 | 0.979 | 0.988 | 0.989 | 1 | 1 | 0.997 | 0.998 | 0.922 | 1 | 0.985 | 0.976 | 0.862 | 1 | 1 | 0.9709 |
| 68 | 1 | 0.976 | 0.997 | 0.994 | 1 | 0.979 | 0.988 | 0.989 | 1 | 1 | 0.997 | 0.998 | 0.922 | 1 | 0.985 | 0.976 | 0.856 | 1 | 1 | 0.9698 |
| 69 | 1 | 0.976 | 0.997 | 0.994 | 1 | 0.979 | 0.988 | 0.989 | 1 | 1 | 0.997 | 0.998 | 0.922 | 1 | 0.986 | 0.977 | 0.862 | 1 | 1 | 0.9709 |
| 70 | 1 | 0.976 | 0.997 | 0.994 | 1 | 0.979 | 0.988 | 0.989 | 1 | 1 | 0.997 | 0.998 | 0.929 | 1 | 0.986 | 0.978 | 0.867 | 1 | 1 | 0.972 |
| 71 | 1 | 0.976 | 0.997 | 0.994 | 1 | 0.979 | 0.988 | 0.989 | 1 | 1 | 0.997 | 0.998 | 0.929 | 1 | 0.985 | 0.977 | 0.867 | 1 | 1 | 0.972 |
| 72 | 1 | 0.976 | 0.99 | 0.989 | 1 | 0.958 | 0.98 | 0.981 | 1 | 1 | 0.997 | 0.998 | 0.948 | 1 | 0.976 | 0.973 | 0.878 | 1 | 1 | 0.9742 |
| 73 | 1 | 0.976 | 0.99 | 0.989 | 1 | 0.958 | 0.98 | 0.981 | 1 | 1 | 0.997 | 0.998 | 0.948 | 1 | 0.976 | 0.973 | 0.872 | 1 | 1 | 0.9731 |
| 74 | 1 | 0.976 | 0.99 | 0.989 | 1 | 0.958 | 0.98 | 0.981 | 1 | 1 | 0.997 | 0.998 | 0.948 | 1 | 0.976 | 0.973 | 0.862 | 1 | 1 | 0.9709 |
| 75 | 1 | 0.976 | 0.99 | 0.989 | 1 | 0.958 | 0.978 | 0.979 | 1 | 1 | 0.997 | 0.998 | 0.948 | 1 | 0.976 | 0.973 | 0.867 | 1 | 1 | 0.972 |
| 76 | 1 | 0.976 | 0.99 | 0.989 | 1 | 0.958 | 0.98 | 0.981 | 1 | 1 | 1 | 1 | 0.942 | 1 | 0.977 | 0.973 | 0.856 | 1 | 1 | 0.9698 |
| 77 | 1 | 0.976 | 0.993 | 0.992 | 1 | 0.958 | 0.978 | 0.979 | 1 | 1 | 0.997 | 0.998 | 0.935 | 1 | 0.976 | 0.97 | 0.851 | 1 | 1 | 0.9686 |
| 78 | 1 | 0.976 | 0.99 | 0.989 | 1 | 0.958 | 0.978 | 0.979 | 1 | 1 | 0.997 | 0.998 | 0.942 | 1 | 0.976 | 0.971 | 0.856 | 1 | 1 | 0.9698 |
| 79 | 1 | 0.976 | 0.993 | 0.992 | 1 | 0.958 | 0.978 | 0.979 | 1 | 1 | 0.997 | 0.998 | 0.935 | 0.981 | 0.976 | 0.969 | 0.851 | 1 | 1 | 0.9686 |
| 80 | 1 | 0.976 | 0.99 | 0.989 | 1 | 0.958 | 0.978 | 0.979 | 1 | 1 | 0.997 | 0.998 | 0.942 | 0.981 | 0.976 | 0.97 | 0.851 | 1 | 1 | 0.9686 |
| 81 | 1 | 0.976 | 0.99 | 0.989 | 1 | 0.958 | 0.978 | 0.979 | 1 | 1 | 0.997 | 0.998 | 0.935 | 1 | 0.976 | 0.97 | 0.851 | 1 | 1 | 0.9686 |
| 82 | 1 | 0.976 | 0.993 | 0.992 | 1 | 0.958 | 0.978 | 0.979 | 1 | 1 | 0.997 | 0.998 | 0.942 | 1 | 0.976 | 0.971 | 0.851 | 1 | 1 | 0.9686 |
| 83 | 1 | 0.976 | 0.99 | 0.989 | 1 | 0.958 | 0.98 | 0.981 | 1 | 1 | 0.997 | 0.998 | 0.948 | 1 | 0.976 | 0.973 | 0.856 | 1 | 1 | 0.9698 |
| 84 | 1 | 0.976 | 0.99 | 0.989 | 1 | 0.958 | 0.98 | 0.981 | 1 | 1 | 0.997 | 0.998 | 0.935 | 1 | 0.976 | 0.97 | 0.872 | 1 | 1 | 0.9731 |
| 85 | 1 | 1 | 0.99 | 0.992 | 1 | 0.979 | 0.98 | 0.983 | 1 | 1 | 0.997 | 0.998 | 0.948 | 1 | 0.976 | 0.973 | 0.878 | 1 | 1 | 0.9742 |
| 86 | 1 | 1 | 0.99 | 0.992 | 1 | 0.979 | 0.98 | 0.983 | 1 | 1 | 0.997 | 0.998 | 0.948 | 1 | 0.976 | 0.973 | 0.872 | 1 | 1 | 0.9731 |
| 87 | 1 | 1 | 0.99 | 0.992 | 1 | 0.979 | 0.98 | 0.983 | 1 | 1 | 0.997 | 0.998 | 0.948 | 1 | 0.976 | 0.973 | 0.872 | 1 | 1 | 0.9731 |
| 88 | 1 | 1 | 0.99 | 0.992 | 1 | 0.979 | 0.98 | 0.983 | 1 | 1 | 0.997 | 0.998 | 0.948 | 1 | 0.976 | 0.973 | 0.872 | 1 | 1 | 0.9731 |
| 89 | 1 | 1 | 0.99 | 0.992 | 1 | 0.979 | 0.98 | 0.983 | 1 | 1 | 0.997 | 0.998 | 0.948 | 1 | 0.976 | 0.973 | 0.878 | 1 | 1 | 0.9742 |
| 90 | 1 | 1 | 0.99 | 0.992 | 1 | 0.979 | 0.98 | 0.983 | 1 | 1 | 0.997 | 0.998 | 0.955 | 1 | 0.976 | 0.974 | 0.878 | 1 | 1 | 0.9742 |
| 91 | 1 | 1 | 0.99 | 0.992 | 1 | 0.979 | 0.98 | 0.983 | 1 | 1 | 0.997 | 0.998 | 0.955 | 1 | 0.976 | 0.974 | 0.872 | 1 | 1 | 0.9731 |
| 92 | 1 | 1 | 0.99 | 0.992 | 1 | 0.979 | 0.98 | 0.983 | 1 | 1 | 0.997 | 0.998 | 0.955 | 1 | 0.976 | 0.974 | 0.878 | 1 | 1 | 0.9742 |
| 93 | 1 | 1 | 0.99 | 0.992 | 1 | 0.979 | 0.98 | 0.983 | 1 | 1 | 0.997 | 0.998 | 0.955 | 1 | 0.976 | 0.974 | 0.872 | 1 | 1 | 0.9731 |
| 94 | 1 | 1 | 0.99 | 0.992 | 1 | 0.979 | 0.978 | 0.981 | 1 | 1 | 0.997 | 0.998 | 0.955 | 1 | 0.976 | 0.974 | 0.867 | 1 | 1 | 0.972 |
| 95 | 1 | 1 | 0.99 | 0.992 | 1 | 0.979 | 0.978 | 0.981 | 1 | 1 | 1 | 1 | 0.955 | 1 | 0.974 | 0.973 | 0.904 | 1 | 1 | 0.9798 |
| 96 | 1 | 1 | 0.99 | 0.992 | 1 | 0.979 | 0.978 | 0.981 | 1 | 1 | 1 | 1 | 0.955 | 1 | 0.974 | 0.973 | 0.904 | 1 | 1 | 0.9798 |
| 97 | 1 | 1 | 0.99 | 0.992 | 1 | 0.979 | 0.978 | 0.981 | 1 | 1 | 1 | 1 | 0.955 | 1 | 0.974 | 0.973 | 0.904 | 1 | 1 | 0.9798 |
| 98 | 1 | 1 | 0.99 | 0.992 | 1 | 0.979 | 0.98 | 0.983 | 1 | 1 | 1 | 1 | 0.955 | 1 | 0.976 | 0.974 | 0.894 | 1 | 1 | 0.9776 |
| 99 | 1 | 1 | 0.99 | 0.992 | 1 | 0.979 | 0.98 | 0.983 | 1 | 1 | 1 | 1 | 0.955 | 1 | 0.973 | 0.971 | 0.91 | 1 | 1 | 0.981 |
| 100 | 1 | 1 | 0.99 | 0.992 | 1 | 0.979 | 0.98 | 0.983 | 1 | 1 | 0.997 | 0.998 | 0.955 | 1 | 0.981 | 0.978 | 0.888 | 1 | 1 | 0.9765 |
| 101 | 1 | 1 | 0.99 | 0.992 | 1 | 0.979 | 0.98 | 0.983 | 1 | 1 | 0.997 | 0.998 | 0.955 | 1 | 0.981 | 0.978 | 0.888 | 1 | 1 | 0.9765 |
| 102 | 1 | 1 | 0.99 | 0.992 | 1 | 0.979 | 0.98 | 0.983 | 1 | 1 | 1 | 1 | 0.961 | 1 | 0.982 | 0.98 | 0.883 | 1 | 1 | 0.9754 |
| 103 | 1 | 1 | 0.99 | 0.992 | 1 | 0.979 | 0.98 | 0.983 | 1 | 1 | 1 | 1 | 0.961 | 1 | 0.982 | 0.98 | 0.883 | 1 | 1 | 0.9754 |
| 104 | 1 | 1 | 0.99 | 0.992 | 1 | 0.979 | 0.98 | 0.983 | 1 | 1 | 0.997 | 0.998 | 0.961 | 1 | 0.982 | 0.98 | 0.883 | 1 | 1 | 0.9754 |
| 105 | 1 | 1 | 0.99 | 0.992 | 1 | 0.979 | 0.98 | 0.983 | 1 | 1 | 0.997 | 0.998 | 0.955 | 1 | 0.982 | 0.979 | 0.883 | 1 | 1 | 0.9754 |
| 106 | 1 | 1 | 0.99 | 0.992 | 1 | 0.958 | 0.98 | 0.981 | 1 | 1 | 0.997 | 0.998 | 0.961 | 1 | 0.981 | 0.979 | 0.91 | 1 | 1 | 0.981 |
| 107 | 1 | 1 | 0.99 | 0.992 | 1 | 0.979 | 0.98 | 0.983 | 1 | 1 | 0.997 | 0.998 | 0.961 | 0.981 | 0.981 | 0.978 | 0.904 | 1 | 1 | 0.9798 |
| 108 | 1 | 1 | 0.99 | 0.992 | 1 | 0.979 | 0.98 | 0.983 | 0.987 | 1 | 1 | 0.998 | 0.961 | 0.981 | 0.981 | 0.978 | 0.904 | 1 | 1 | 0.9798 |
| 109 | 1 | 1 | 0.99 | 0.992 | 1 | 0.979 | 0.98 | 0.983 | 0.987 | 1 | 1 | 0.998 | 0.955 | 0.981 | 0.981 | 0.977 | 0.904 | 1 | 1 | 0.9798 |
| 110 | 1 | 1 | 0.99 | 0.992 | 1 | 0.979 | 0.978 | 0.981 | 0.987 | 1 | 1 | 0.998 | 0.961 | 0.981 | 0.981 | 0.978 | 0.904 | 1 | 1 | 0.9798 |
| 111 | 1 | 1 | 0.99 | 0.992 | 1 | 0.979 | 0.978 | 0.981 | 0.987 | 1 | 1 | 0.998 | 0.961 | 1 | 0.981 | 0.979 | 0.904 | 1 | 1 | 0.9798 |
| 112 | 1 | 1 | 0.99 | 0.992 | 1 | 0.979 | 0.978 | 0.981 | 0.987 | 1 | 1 | 0.998 | 0.961 | 1 | 0.981 | 0.979 | 0.899 | 1 | 1 | 0.9787 |
| 113 | 1 | 1 | 0.99 | 0.992 | 1 | 1 | 0.978 | 0.983 | 0.987 | 1 | 1 | 0.998 | 0.961 | 1 | 0.981 | 0.979 | 0.904 | 1 | 1 | 0.9798 |
| 114 | 1 | 1 | 0.99 | 0.992 | 1 | 1 | 0.978 | 0.983 | 0.987 | 1 | 1 | 0.998 | 0.961 | 1 | 0.981 | 0.979 | 0.894 | 1 | 1 | 0.9776 |
| 115 | 1 | 1 | 0.99 | 0.992 | 1 | 1 | 0.978 | 0.983 | 0.987 | 1 | 1 | 0.998 | 0.955 | 1 | 0.98 | 0.977 | 0.899 | 1 | 1 | 0.9787 |
| 116 | 1 | 1 | 0.99 | 0.992 | 1 | 1 | 0.978 | 0.983 | 0.987 | 1 | 1 | 0.998 | 0.955 | 1 | 0.98 | 0.977 | 0.894 | 1 | 1 | 0.9776 |
| 117 | 1 | 1 | 0.99 | 0.992 | 1 | 1 | 0.978 | 0.983 | 0.987 | 1 | 1 | 0.998 | 0.955 | 1 | 0.98 | 0.977 | 0.894 | 1 | 1 | 0.9776 |
| 118 | 1 | 1 | 0.99 | 0.992 | 1 | 1 | 0.975 | 0.981 | 0.987 | 1 | 1 | 0.998 | 0.961 | 1 | 0.981 | 0.979 | 0.894 | 1 | 1 | 0.9776 |
| 119 | 1 | 1 | 0.99 | 0.992 | 1 | 0.979 | 0.97 | 0.975 | 0.987 | 1 | 1 | 0.998 | 0.955 | 1 | 0.984 | 0.98 | 0.888 | 1 | 1 | 0.9765 |
| 120 | 1 | 1 | 0.99 | 0.992 | 1 | 1 | 0.97 | 0.977 | 0.987 | 1 | 1 | 0.998 | 0.961 | 1 | 0.984 | 0.981 | 0.894 | 1 | 1 | 0.9776 |
| 121 | 1 | 1 | 0.99 | 0.992 | 1 | 1 | 0.978 | 0.983 | 0.987 | 1 | 1 | 0.998 | 0.961 | 1 | 0.984 | 0.981 | 0.894 | 1 | 1 | 0.9776 |
| 122 | 1 | 1 | 0.99 | 0.992 | 1 | 0.958 | 0.975 | 0.977 | 0.987 | 1 | 1 | 0.998 | 0.961 | 1 | 0.984 | 0.981 | 0.899 | 1 | 1 | 0.9787 |
| 123 | 1 | 1 | 0.99 | 0.992 | 1 | 0.958 | 0.973 | 0.975 | 0.987 | 1 | 1 | 0.998 | 0.955 | 1 | 0.984 | 0.98 | 0.888 | 1 | 1 | 0.9765 |
| 124 | 1 | 1 | 0.99 | 0.992 | 1 | 0.958 | 0.97 | 0.974 | 0.987 | 1 | 1 | 0.998 | 0.955 | 1 | 0.984 | 0.98 | 0.888 | 1 | 1 | 0.9765 |
| 125 | 1 | 1 | 0.99 | 0.992 | 1 | 0.958 | 0.98 | 0.981 | 0.987 | 1 | 1 | 0.998 | 0.955 | 1 | 0.985 | 0.981 | 0.888 | 1 | 1 | 0.9765 |
| 126 | 1 | 1 | 0.99 | 0.992 | 1 | 0.958 | 0.98 | 0.981 | 0.987 | 1 | 1 | 0.998 | 0.955 | 1 | 0.985 | 0.981 | 0.888 | 1 | 1 | 0.9765 |
| 127 | 1 | 1 | 0.99 | 0.992 | 1 | 0.958 | 0.98 | 0.981 | 0.987 | 1 | 1 | 0.998 | 0.955 | 1 | 0.985 | 0.981 | 0.894 | 1 | 1 | 0.9776 |
| 128 | 1 | 1 | 0.99 | 0.992 | 1 | 0.958 | 0.98 | 0.981 | 0.987 | 1 | 1 | 0.998 | 0.955 | 1 | 0.985 | 0.981 | 0.894 | 1 | 1 | 0.9776 |
| 129 | 1 | 1 | 0.99 | 0.992 | 1 | 0.958 | 0.978 | 0.979 | 0.987 | 1 | 1 | 0.998 | 0.955 | 1 | 0.985 | 0.981 | 0.894 | 1 | 1 | 0.9776 |
| 130 | 1 | 1 | 0.99 | 0.992 | 1 | 0.958 | 0.978 | 0.979 | 0.987 | 1 | 1 | 0.998 | 0.955 | 1 | 0.984 | 0.98 | 0.894 | 1 | 1 | 0.9776 |
| 131 | 1 | 1 | 0.99 | 0.992 | 1 | 0.958 | 0.98 | 0.981 | 0.987 | 1 | 1 | 0.998 | 0.955 | 1 | 0.988 | 0.983 | 0.894 | 1 | 1 | 0.9776 |
| 132 | 1 | 1 | 0.99 | 0.992 | 1 | 0.958 | 0.98 | 0.981 | 0.987 | 1 | 1 | 0.998 | 0.955 | 1 | 0.989 | 0.984 | 0.894 | 1 | 1 | 0.9776 |
| 133 | 1 | 1 | 0.99 | 0.992 | 1 | 0.958 | 0.98 | 0.981 | 0.987 | 1 | 1 | 0.998 | 0.955 | 1 | 0.989 | 0.984 | 0.894 | 1 | 1 | 0.9776 |
| 134 | 1 | 1 | 0.99 | 0.992 | 1 | 0.958 | 0.98 | 0.981 | 0.987 | 1 | 1 | 0.998 | 0.955 | 1 | 0.989 | 0.984 | 0.888 | 1 | 1 | 0.9765 |
| 135 | 1 | 1 | 0.99 | 0.992 | 1 | 0.958 | 0.98 | 0.981 | 0.987 | 1 | 1 | 0.998 | 0.955 | 1 | 0.988 | 0.983 | 0.888 | 1 | 1 | 0.9765 |
| 136 | 1 | 1 | 0.99 | 0.992 | 1 | 0.958 | 0.98 | 0.981 | 0.987 | 1 | 1 | 0.998 | 0.955 | 1 | 0.988 | 0.983 | 0.888 | 1 | 1 | 0.9765 |
| 137 | 1 | 1 | 0.99 | 0.992 | 1 | 0.958 | 0.98 | 0.981 | 0.987 | 1 | 1 | 0.998 | 0.955 | 1 | 0.989 | 0.984 | 0.883 | 1 | 1 | 0.9754 |
| 138 | 1 | 1 | 0.99 | 0.992 | 1 | 0.958 | 0.98 | 0.981 | 0.987 | 1 | 1 | 0.998 | 0.955 | 1 | 0.988 | 0.983 | 0.883 | 1 | 1 | 0.9754 |
| 139 | 1 | 1 | 0.99 | 0.992 | 1 | 0.958 | 0.983 | 0.983 | 0.987 | 1 | 1 | 0.998 | 0.955 | 1 | 0.988 | 0.983 | 0.883 | 1 | 1 | 0.9754 |
| 140 | 1 | 1 | 0.99 | 0.992 | 1 | 0.958 | 0.983 | 0.983 | 0.987 | 1 | 1 | 0.998 | 0.955 | 1 | 0.988 | 0.983 | 0.883 | 1 | 1 | 0.9754 |
| 141 | 1 | 1 | 0.99 | 0.992 | 1 | 0.958 | 0.983 | 0.983 | 0.987 | 1 | 1 | 0.998 | 0.955 | 1 | 0.988 | 0.983 | 0.883 | 1 | 1 | 0.9754 |
| 142 | 1 | 1 | 0.99 | 0.992 | 1 | 0.958 | 0.983 | 0.983 | 0.987 | 1 | 1 | 0.998 | 0.955 | 1 | 0.986 | 0.982 | 0.883 | 1 | 1 | 0.9754 |
| 143 | 1 | 1 | 0.99 | 0.992 | 1 | 0.958 | 0.983 | 0.983 | 0.987 | 1 | 1 | 0.998 | 0.955 | 1 | 0.986 | 0.982 | 0.883 | 1 | 1 | 0.9754 |
| 144 | 1 | 1 | 0.99 | 0.992 | 1 | 0.958 | 0.983 | 0.983 | 0.987 | 1 | 1 | 0.998 | 0.955 | 1 | 0.988 | 0.983 | 0.883 | 1 | 1 | 0.9754 |
| 145 | 1 | 1 | 0.99 | 0.992 | 1 | 0.958 | 0.983 | 0.983 | 0.987 | 1 | 1 | 0.998 | 0.948 | 1 | 0.988 | 0.982 | 0.883 | 1 | 1 | 0.9754 |
| 146 | 1 | 1 | 0.99 | 0.992 | 1 | 0.958 | 0.983 | 0.983 | 0.987 | 1 | 1 | 0.998 | 0.948 | 1 | 0.988 | 0.982 | 0.883 | 1 | 1 | 0.9754 |
| 147 | 1 | 1 | 0.99 | 0.992 | 1 | 0.958 | 0.98 | 0.981 | 0.987 | 1 | 1 | 0.998 | 0.948 | 1 | 0.986 | 0.981 | 0.883 | 1 | 1 | 0.9754 |
| 148 | 1 | 1 | 0.99 | 0.992 | 1 | 0.958 | 0.983 | 0.983 | 0.987 | 1 | 1 | 0.998 | 0.948 | 1 | 0.986 | 0.981 | 0.883 | 1 | 1 | 0.9754 |
| 149 | 1 | 1 | 0.99 | 0.992 | 1 | 0.958 | 0.983 | 0.983 | 0.987 | 1 | 1 | 0.998 | 0.955 | 1 | 0.986 | 0.982 | 0.883 | 1 | 1 | 0.9754 |
| 150 | 1 | 1 | 0.99 | 0.992 | 1 | 0.958 | 0.983 | 0.983 | 0.987 | 1 | 1 | 0.998 | 0.955 | 1 | 0.986 | 0.982 | 0.883 | 1 | 1 | 0.9754 |
| 151 | 1 | 1 | 0.99 | 0.992 | 1 | 0.958 | 0.983 | 0.983 | 0.987 | 1 | 1 | 0.998 | 0.955 | 1 | 0.986 | 0.982 | 0.883 | 1 | 1 | 0.9754 |
| 152 | 1 | 1 | 0.99 | 0.992 | 1 | 0.958 | 0.983 | 0.983 | 0.987 | 1 | 1 | 0.998 | 0.955 | 1 | 0.986 | 0.982 | 0.883 | 1 | 1 | 0.9754 |
| 153 | 1 | 1 | 0.99 | 0.992 | 1 | 0.958 | 0.983 | 0.983 | 0.987 | 1 | 1 | 0.998 | 0.955 | 1 | 0.988 | 0.983 | 0.883 | 1 | 1 | 0.9754 |
| 154 | 1 | 1 | 0.99 | 0.992 | 1 | 0.958 | 0.983 | 0.983 | 0.987 | 1 | 1 | 0.998 | 0.955 | 1 | 0.988 | 0.983 | 0.883 | 1 | 1 | 0.9754 |
| 155 | 1 | 1 | 0.99 | 0.992 | 1 | 0.958 | 0.983 | 0.983 | 0.987 | 1 | 1 | 0.998 | 0.955 | 1 | 0.989 | 0.984 | 0.883 | 1 | 1 | 0.9754 |
| 156 | 1 | 1 | 0.99 | 0.992 | 1 | 0.958 | 0.983 | 0.983 | 0.987 | 1 | 1 | 0.998 | 0.955 | 1 | 0.991 | 0.985 | 0.883 | 1 | 1 | 0.9754 |
| 157 | 1 | 1 | 0.99 | 0.992 | 1 | 0.958 | 0.983 | 0.983 | 0.987 | 1 | 1 | 0.998 | 0.955 | 1 | 0.986 | 0.982 | 0.883 | 1 | 1 | 0.9754 |
| 158 | 1 | 1 | 0.99 | 0.992 | 1 | 0.958 | 0.983 | 0.983 | 0.987 | 1 | 1 | 0.998 | 0.955 | 1 | 0.986 | 0.982 | 0.883 | 1 | 1 | 0.9754 |
| 159 | 1 | 1 | 0.99 | 0.992 | 1 | 0.958 | 0.983 | 0.983 | 0.987 | 1 | 1 | 0.998 | 0.955 | 1 | 0.986 | 0.982 | 0.883 | 1 | 1 | 0.9754 |
| 160 | 1 | 1 | 0.99 | 0.992 | 1 | 0.958 | 0.983 | 0.983 | 1 | 1 | 1 | 1 | 0.955 | 1 | 0.986 | 0.982 | 0.883 | 1 | 1 | 0.9754 |
| 161 | 1 | 1 | 0.99 | 0.992 | 1 | 0.958 | 0.983 | 0.983 | 1 | 1 | 1 | 1 | 0.948 | 1 | 0.989 | 0.983 | 0.878 | 1 | 1 | 0.9742 |
| 162 | 1 | 1 | 0.99 | 0.992 | 1 | 0.958 | 0.983 | 0.983 | 1 | 1 | 1 | 1 | 0.948 | 1 | 0.988 | 0.982 | 0.878 | 1 | 1 | 0.9742 |
| 163 | 1 | 1 | 0.99 | 0.992 | 1 | 0.958 | 0.983 | 0.983 | 1 | 1 | 1 | 1 | 0.948 | 1 | 0.991 | 0.984 | 0.878 | 1 | 1 | 0.9742 |
| 164 | 1 | 1 | 0.99 | 0.992 | 1 | 0.958 | 0.985 | 0.985 | 0.987 | 1 | 1 | 0.998 | 0.942 | 1 | 0.989 | 0.982 | 0.872 | 1 | 1 | 0.9731 |
| 165 | 1 | 1 | 0.99 | 0.992 | 1 | 0.958 | 0.985 | 0.985 | 0.987 | 1 | 1 | 0.998 | 0.942 | 1 | 0.991 | 0.983 | 0.872 | 1 | 1 | 0.9731 |
| 166 | 1 | 1 | 0.99 | 0.992 | 1 | 0.958 | 0.983 | 0.983 | 0.987 | 1 | 1 | 0.998 | 0.942 | 1 | 0.991 | 0.983 | 0.872 | 1 | 1 | 0.9731 |
| 167 | 1 | 1 | 0.99 | 0.992 | 1 | 0.958 | 0.983 | 0.983 | 0.987 | 1 | 1 | 0.998 | 0.942 | 1 | 0.991 | 0.983 | 0.872 | 1 | 1 | 0.9731 |
| 168 | 1 | 1 | 0.99 | 0.992 | 1 | 0.958 | 0.983 | 0.983 | 0.987 | 1 | 1 | 0.998 | 0.942 | 1 | 0.989 | 0.982 | 0.872 | 1 | 1 | 0.9731 |
| 169 | 1 | 1 | 0.99 | 0.992 | 1 | 0.958 | 0.983 | 0.983 | 0.987 | 1 | 1 | 0.998 | 0.948 | 1 | 0.992 | 0.985 | 0.872 | 1 | 1 | 0.9731 |
| 170 | 1 | 1 | 0.99 | 0.992 | 1 | 0.958 | 0.985 | 0.985 | 0.987 | 1 | 1 | 0.998 | 0.948 | 1 | 0.989 | 0.983 | 0.867 | 1 | 1 | 0.972 |
| 171 | 1 | 1 | 0.99 | 0.992 | 1 | 0.979 | 0.983 | 0.985 | 0.987 | 1 | 1 | 0.998 | 0.948 | 1 | 0.991 | 0.984 | 0.872 | 1 | 1 | 0.9731 |
| 172 | 1 | 1 | 0.99 | 0.992 | 1 | 0.979 | 0.983 | 0.985 | 0.987 | 1 | 1 | 0.998 | 0.942 | 1 | 0.991 | 0.983 | 0.867 | 1 | 1 | 0.972 |
| 173 | 1 | 1 | 0.99 | 0.992 | 1 | 0.979 | 0.985 | 0.987 | 0.987 | 1 | 1 | 0.998 | 0.942 | 1 | 0.991 | 0.983 | 0.851 | 1 | 1 | 0.9686 |
| 174 | 1 | 1 | 0.99 | 0.992 | 1 | 0.979 | 0.985 | 0.987 | 0.987 | 1 | 1 | 0.998 | 0.942 | 1 | 0.991 | 0.983 | 0.851 | 1 | 1 | 0.9686 |
| 175 | 1 | 1 | 0.99 | 0.992 | 1 | 0.979 | 0.985 | 0.987 | 0.987 | 1 | 1 | 0.998 | 0.942 | 1 | 0.991 | 0.983 | 0.851 | 1 | 1 | 0.9686 |
| 176 | 1 | 1 | 0.99 | 0.992 | 1 | 0.979 | 0.985 | 0.987 | 0.987 | 1 | 1 | 0.998 | 0.942 | 1 | 0.991 | 0.983 | 0.851 | 1 | 1 | 0.9686 |
| 177 | 1 | 1 | 0.99 | 0.992 | 1 | 0.979 | 0.983 | 0.985 | 0.987 | 1 | 1 | 0.998 | 0.942 | 1 | 0.991 | 0.983 | 0.856 | 1 | 1 | 0.9698 |
| 178 | 1 | 1 | 0.99 | 0.992 | 1 | 0.979 | 0.98 | 0.983 | 0.987 | 1 | 1 | 0.998 | 0.942 | 1 | 0.988 | 0.981 | 0.856 | 1 | 1 | 0.9698 |
| 179 | 1 | 1 | 0.99 | 0.992 | 1 | 0.979 | 0.98 | 0.983 | 0.987 | 1 | 1 | 0.998 | 0.942 | 1 | 0.988 | 0.981 | 0.856 | 1 | 1 | 0.9698 |
| 180 | 1 | 1 | 0.99 | 0.992 | 1 | 0.979 | 0.978 | 0.981 | 0.987 | 1 | 1 | 0.998 | 0.942 | 1 | 0.988 | 0.981 | 0.856 | 1 | 1 | 0.9698 |
| 181 | 1 | 1 | 0.99 | 0.992 | 1 | 0.979 | 0.978 | 0.981 | 0.987 | 1 | 1 | 0.998 | 0.942 | 1 | 0.988 | 0.981 | 0.856 | 1 | 1 | 0.9698 |
| 182 | 1 | 1 | 0.99 | 0.992 | 1 | 1 | 0.978 | 0.983 | 0.987 | 1 | 1 | 0.998 | 0.942 | 1 | 0.989 | 0.982 | 0.856 | 1 | 1 | 0.9698 |
| 183 | 1 | 1 | 0.99 | 0.992 | 1 | 1 | 0.978 | 0.983 | 0.987 | 1 | 1 | 0.998 | 0.942 | 1 | 0.989 | 0.982 | 0.856 | 1 | 1 | 0.9698 |
| 184 | 1 | 1 | 0.99 | 0.992 | 1 | 1 | 0.978 | 0.983 | 1 | 1 | 1 | 1 | 0.942 | 1 | 0.991 | 0.983 | 0.856 | 1 | 1 | 0.9698 |
| 185 | 1 | 1 | 0.99 | 0.992 | 1 | 1 | 0.978 | 0.983 | 1 | 1 | 1 | 1 | 0.942 | 1 | 0.991 | 0.983 | 0.856 | 1 | 1 | 0.9698 |
| 186 | 1 | 1 | 0.99 | 0.992 | 1 | 1 | 0.978 | 0.983 | 1 | 1 | 1 | 1 | 0.942 | 1 | 0.991 | 0.983 | 0.856 | 1 | 1 | 0.9698 |
| 187 | 1 | 1 | 0.99 | 0.992 | 1 | 1 | 0.978 | 0.983 | 1 | 1 | 0.997 | 0.998 | 0.942 | 1 | 0.989 | 0.982 | 0.856 | 1 | 1 | 0.9698 |
| 188 | 1 | 1 | 0.99 | 0.992 | 1 | 1 | 0.978 | 0.983 | 1 | 1 | 0.997 | 0.998 | 0.942 | 1 | 0.991 | 0.983 | 0.856 | 1 | 1 | 0.9698 |
| 189 | 1 | 1 | 0.99 | 0.992 | 1 | 1 | 0.978 | 0.983 | 1 | 1 | 0.997 | 0.998 | 0.942 | 1 | 0.991 | 0.983 | 0.856 | 1 | 1 | 0.9698 |
| 190 | 1 | 1 | 0.99 | 0.992 | 1 | 1 | 0.978 | 0.983 | 1 | 1 | 0.997 | 0.998 | 0.942 | 1 | 0.991 | 0.983 | 0.856 | 1 | 1 | 0.9698 |
| 191 | 1 | 1 | 0.99 | 0.992 | 1 | 1 | 0.978 | 0.983 | 1 | 1 | 0.997 | 0.998 | 0.942 | 1 | 0.989 | 0.982 | 0.856 | 1 | 1 | 0.9698 |
| 192 | 1 | 1 | 0.99 | 0.992 | 1 | 1 | 0.975 | 0.981 | 1 | 1 | 1 | 1 | 0.942 | 1 | 0.988 | 0.981 | 0.851 | 1 | 1 | 0.9686 |
| 193 | 1 | 1 | 0.99 | 0.992 | 1 | 1 | 0.97 | 0.977 | 1 | 1 | 1 | 1 | 0.942 | 1 | 0.989 | 0.982 | 0.851 | 1 | 1 | 0.9686 |
| 194 | 1 | 1 | 0.99 | 0.992 | 1 | 1 | 0.968 | 0.975 | 1 | 1 | 1 | 1 | 0.935 | 1 | 0.989 | 0.981 | 0.851 | 1 | 1 | 0.9686 |
| 195 | 1 | 1 | 0.99 | 0.992 | 1 | 1 | 0.968 | 0.975 | 1 | 1 | 1 | 1 | 0.935 | 1 | 0.989 | 0.981 | 0.851 | 1 | 1 | 0.9686 |
| 196 | 1 | 1 | 0.99 | 0.992 | 1 | 1 | 0.968 | 0.975 | 1 | 1 | 1 | 1 | 0.935 | 1 | 0.988 | 0.98 | 0.851 | 1 | 1 | 0.9686 |
| 197 | 1 | 1 | 0.99 | 0.992 | 1 | 1 | 0.968 | 0.975 | 1 | 1 | 1 | 1 | 0.935 | 1 | 0.988 | 0.98 | 0.851 | 1 | 1 | 0.9686 |
| 198 | 1 | 1 | 0.99 | 0.992 | 1 | 1 | 0.97 | 0.977 | 1 | 1 | 1 | 1 | 0.935 | 1 | 0.989 | 0.981 | 0.851 | 1 | 1 | 0.9686 |
| 199 | 1 | 1 | 0.99 | 0.992 | 1 | 1 | 0.975 | 0.981 | 1 | 1 | 1 | 1 | 0.935 | 1 | 0.989 | 0.981 | 0.851 | 1 | 1 | 0.9686 |
| 200 | 1 | 1 | 0.99 | 0.992 | 1 | 1 | 0.975 | 0.981 | 1 | 1 | 1 | 1 | 0.935 | 1 | 0.989 | 0.981 | 0.851 | 1 | 1 | 0.9686 |
| 201 | 1 | 1 | 0.99 | 0.992 | 1 | 1 | 0.973 | 0.979 | 1 | 1 | 1 | 1 | 0.935 | 1 | 0.989 | 0.981 | 0.856 | 1 | 1 | 0.9698 |
| 202 | 1 | 1 | 0.99 | 0.992 | 1 | 1 | 0.973 | 0.979 | 1 | 1 | 1 | 1 | 0.935 | 1 | 0.989 | 0.981 | 0.851 | 1 | 1 | 0.9686 |
| 203 | 1 | 1 | 0.99 | 0.992 | 1 | 1 | 0.973 | 0.979 | 1 | 1 | 1 | 1 | 0.935 | 1 | 0.989 | 0.981 | 0.851 | 1 | 1 | 0.9686 |
| 204 | 1 | 1 | 0.99 | 0.992 | 1 | 1 | 0.973 | 0.979 | 0.987 | 1 | 1 | 0.998 | 0.935 | 1 | 0.989 | 0.981 | 0.851 | 1 | 1 | 0.9686 |
| 205 | 1 | 1 | 0.99 | 0.992 | 1 | 1 | 0.973 | 0.979 | 0.987 | 1 | 1 | 0.998 | 0.935 | 1 | 0.989 | 0.981 | 0.851 | 1 | 1 | 0.9686 |
| 206 | 1 | 1 | 0.99 | 0.992 | 1 | 1 | 0.973 | 0.979 | 0.987 | 1 | 1 | 0.998 | 0.942 | 1 | 0.989 | 0.982 | 0.851 | 1 | 1 | 0.9686 |
| 207 | 1 | 1 | 0.99 | 0.992 | 1 | 1 | 0.975 | 0.981 | 0.987 | 1 | 1 | 0.998 | 0.935 | 1 | 0.991 | 0.982 | 0.856 | 1 | 1 | 0.9698 |
| 208 | 1 | 1 | 0.99 | 0.992 | 1 | 1 | 0.983 | 0.987 | 1 | 1 | 1 | 1 | 0.935 | 1 | 0.991 | 0.982 | 0.856 | 1 | 1 | 0.9698 |
| 209 | 1 | 1 | 0.99 | 0.992 | 1 | 1 | 0.983 | 0.987 | 1 | 1 | 1 | 1 | 0.935 | 1 | 0.991 | 0.982 | 0.856 | 1 | 1 | 0.9698 |
| 210 | 1 | 1 | 0.99 | 0.992 | 1 | 1 | 0.983 | 0.987 | 1 | 1 | 1 | 1 | 0.935 | 1 | 0.991 | 0.982 | 0.856 | 1 | 1 | 0.9698 |
| 211 | 1 | 1 | 0.99 | 0.992 | 1 | 1 | 0.983 | 0.987 | 1 | 1 | 1 | 1 | 0.935 | 1 | 0.991 | 0.982 | 0.851 | 1 | 1 | 0.9686 |
| 212 | 1 | 1 | 0.99 | 0.992 | 1 | 1 | 0.983 | 0.987 | 0.987 | 1 | 1 | 0.998 | 0.929 | 1 | 0.992 | 0.982 | 0.851 | 1 | 1 | 0.9686 |
| 213 | 1 | 1 | 0.99 | 0.992 | 1 | 1 | 0.983 | 0.987 | 0.987 | 1 | 1 | 0.998 | 0.929 | 1 | 0.992 | 0.982 | 0.851 | 1 | 1 | 0.9686 |
| 214 | 1 | 1 | 0.99 | 0.992 | 1 | 1 | 0.983 | 0.987 | 0.987 | 1 | 1 | 0.998 | 0.929 | 1 | 0.989 | 0.98 | 0.851 | 1 | 1 | 0.9686 |
| 215 | 1 | 1 | 0.99 | 0.992 | 1 | 1 | 0.983 | 0.987 | 0.987 | 1 | 1 | 0.998 | 0.929 | 1 | 0.991 | 0.981 | 0.851 | 1 | 1 | 0.9686 |
| 216 | 1 | 1 | 0.99 | 0.992 | 1 | 1 | 0.983 | 0.987 | 0.987 | 1 | 1 | 0.998 | 0.929 | 1 | 0.991 | 0.981 | 0.851 | 1 | 1 | 0.9686 |
| 217 | 1 | 1 | 0.99 | 0.992 | 1 | 1 | 0.983 | 0.987 | 0.987 | 1 | 1 | 0.998 | 0.929 | 1 | 0.991 | 0.981 | 0.851 | 1 | 1 | 0.9686 |
| 218 | 1 | 1 | 0.99 | 0.992 | 1 | 1 | 0.983 | 0.987 | 0.987 | 1 | 1 | 0.998 | 0.929 | 1 | 0.991 | 0.981 | 0.851 | 1 | 1 | 0.9686 |
| 219 | 1 | 1 | 0.99 | 0.992 | 1 | 1 | 0.983 | 0.987 | 1 | 1 | 1 | 1 | 0.929 | 1 | 0.986 | 0.978 | 0.851 | 1 | 1 | 0.9686 |
| 220 | 1 | 1 | 0.99 | 0.992 | 1 | 1 | 0.983 | 0.987 | 0.987 | 1 | 1 | 0.998 | 0.929 | 1 | 0.986 | 0.978 | 0.851 | 1 | 1 | 0.9686 |
| 221 | 1 | 1 | 0.99 | 0.992 | 1 | 1 | 0.983 | 0.987 | 0.987 | 1 | 1 | 0.998 | 0.929 | 1 | 0.986 | 0.978 | 0.851 | 1 | 1 | 0.9686 |
| 222 | 1 | 1 | 0.99 | 0.992 | 1 | 1 | 0.983 | 0.987 | 0.987 | 1 | 1 | 0.998 | 0.929 | 1 | 0.988 | 0.979 | 0.851 | 1 | 1 | 0.9686 |
| 223 | 1 | 1 | 0.99 | 0.992 | 1 | 1 | 0.98 | 0.985 | 1 | 1 | 1 | 1 | 0.929 | 1 | 0.988 | 0.979 | 0.851 | 1 | 1 | 0.9686 |
| 224 | 1 | 1 | 0.99 | 0.992 | 1 | 1 | 0.98 | 0.985 | 0.987 | 1 | 1 | 0.998 | 0.929 | 1 | 0.989 | 0.98 | 0.851 | 1 | 1 | 0.9686 |
| 225 | 1 | 1 | 0.99 | 0.992 | 1 | 1 | 0.98 | 0.985 | 0.987 | 1 | 1 | 0.998 | 0.929 | 1 | 0.989 | 0.98 | 0.851 | 1 | 1 | 0.9686 |
| 226 | 1 | 1 | 0.99 | 0.992 | 1 | 1 | 0.98 | 0.985 | 1 | 1 | 1 | 1 | 0.929 | 1 | 0.988 | 0.979 | 0.851 | 1 | 1 | 0.9686 |
| 227 | 1 | 1 | 0.99 | 0.992 | 1 | 1 | 0.98 | 0.985 | 1 | 1 | 1 | 1 | 0.929 | 1 | 0.988 | 0.979 | 0.856 | 1 | 1 | 0.9698 |
| 228 | 1 | 1 | 0.99 | 0.992 | 1 | 1 | 0.98 | 0.985 | 1 | 1 | 1 | 1 | 0.929 | 1 | 0.989 | 0.98 | 0.856 | 1 | 1 | 0.9698 |
| 229 | 1 | 1 | 0.99 | 0.992 | 1 | 1 | 0.98 | 0.985 | 1 | 1 | 1 | 1 | 0.929 | 1 | 0.989 | 0.98 | 0.851 | 1 | 1 | 0.9686 |
| 230 | 1 | 1 | 0.99 | 0.992 | 1 | 1 | 0.98 | 0.985 | 1 | 1 | 1 | 1 | 0.929 | 1 | 0.989 | 0.98 | 0.846 | 1 | 1 | 0.9675 |
| 231 | 1 | 1 | 0.99 | 0.992 | 1 | 1 | 0.978 | 0.983 | 1 | 1 | 1 | 1 | 0.929 | 1 | 0.989 | 0.98 | 0.846 | 1 | 1 | 0.9675 |
| 232 | 1 | 1 | 0.99 | 0.992 | 1 | 1 | 0.98 | 0.985 | 1 | 1 | 1 | 1 | 0.929 | 1 | 0.989 | 0.98 | 0.846 | 1 | 1 | 0.9675 |
| 233 | 1 | 1 | 0.99 | 0.992 | 1 | 1 | 0.98 | 0.985 | 1 | 1 | 1 | 1 | 0.929 | 1 | 0.989 | 0.98 | 0.846 | 1 | 1 | 0.9675 |
| 234 | 1 | 1 | 0.99 | 0.992 | 1 | 1 | 0.978 | 0.983 | 1 | 1 | 1 | 1 | 0.929 | 1 | 0.989 | 0.98 | 0.851 | 1 | 1 | 0.9686 |
| 235 | 1 | 1 | 0.99 | 0.992 | 1 | 1 | 0.978 | 0.983 | 1 | 1 | 1 | 1 | 0.922 | 1 | 0.991 | 0.98 | 0.846 | 1 | 1 | 0.9675 |
| 236 | 1 | 1 | 0.99 | 0.992 | 1 | 1 | 0.978 | 0.983 | 1 | 1 | 1 | 1 | 0.922 | 1 | 0.991 | 0.98 | 0.846 | 1 | 1 | 0.9675 |
| 237 | 1 | 1 | 0.99 | 0.992 | 1 | 1 | 0.975 | 0.981 | 1 | 1 | 1 | 1 | 0.922 | 1 | 0.992 | 0.981 | 0.846 | 1 | 1 | 0.9675 |
| 238 | 1 | 1 | 0.99 | 0.992 | 1 | 1 | 0.978 | 0.983 | 1 | 1 | 1 | 1 | 0.922 | 1 | 0.991 | 0.98 | 0.846 | 1 | 1 | 0.9675 |
| 239 | 1 | 1 | 0.99 | 0.992 | 1 | 1 | 0.978 | 0.983 | 1 | 1 | 1 | 1 | 0.922 | 1 | 0.991 | 0.98 | 0.846 | 1 | 1 | 0.9675 |
| 240 | 1 | 1 | 0.993 | 0.994 | 1 | 1 | 0.978 | 0.983 | 1 | 1 | 1 | 1 | 0.922 | 1 | 0.992 | 0.981 | 0.846 | 1 | 1 | 0.9675 |
| 241 | 1 | 1 | 0.99 | 0.992 | 1 | 1 | 0.978 | 0.983 | 1 | 1 | 1 | 1 | 0.922 | 1 | 0.991 | 0.98 | 0.846 | 1 | 1 | 0.9675 |
| 242 | 1 | 1 | 0.99 | 0.992 | 1 | 1 | 0.978 | 0.983 | 1 | 1 | 1 | 1 | 0.922 | 1 | 0.989 | 0.979 | 0.846 | 1 | 1 | 0.9675 |
| 243 | 1 | 1 | 0.99 | 0.992 | 1 | 1 | 0.975 | 0.981 | 0.987 | 1 | 1 | 0.998 | 0.929 | 1 | 0.985 | 0.977 | 0.846 | 1 | 1 | 0.9675 |
| 244 | 1 | 1 | 0.99 | 0.992 | 1 | 1 | 0.975 | 0.981 | 0.987 | 1 | 1 | 0.998 | 0.929 | 1 | 0.986 | 0.978 | 0.846 | 1 | 1 | 0.9675 |
| 245 | 1 | 1 | 0.99 | 0.992 | 1 | 1 | 0.975 | 0.981 | 0.987 | 1 | 1 | 0.998 | 0.929 | 1 | 0.986 | 0.978 | 0.851 | 1 | 1 | 0.9686 |
| 246 | 1 | 1 | 0.99 | 0.992 | 1 | 1 | 0.975 | 0.981 | 0.987 | 1 | 1 | 0.998 | 0.929 | 1 | 0.986 | 0.978 | 0.851 | 1 | 1 | 0.9686 |
| 247 | 1 | 1 | 0.99 | 0.992 | 1 | 1 | 0.975 | 0.981 | 0.987 | 1 | 1 | 0.998 | 0.929 | 1 | 0.985 | 0.977 | 0.851 | 1 | 1 | 0.9686 |
| 248 | 1 | 1 | 0.99 | 0.992 | 1 | 1 | 0.973 | 0.979 | 0.987 | 1 | 1 | 0.998 | 0.929 | 1 | 0.991 | 0.981 | 0.851 | 1 | 1 | 0.9686 |
| 249 | 1 | 1 | 0.99 | 0.992 | 1 | 1 | 0.973 | 0.979 | 0.987 | 1 | 1 | 0.998 | 0.929 | 1 | 0.991 | 0.981 | 0.851 | 1 | 1 | 0.9686 |
| 250 | 1 | 1 | 0.99 | 0.992 | 1 | 1 | 0.973 | 0.979 | 0.987 | 1 | 1 | 0.998 | 0.929 | 1 | 0.991 | 0.981 | 0.851 | 1 | 1 | 0.9686 |
| 251 | 1 | 1 | 0.99 | 0.992 | 1 | 1 | 0.973 | 0.979 | 0.987 | 1 | 1 | 0.998 | 0.929 | 1 | 0.991 | 0.981 | 0.851 | 1 | 1 | 0.9686 |
| 252 | 1 | 1 | 0.99 | 0.992 | 1 | 1 | 0.973 | 0.979 | 0.987 | 1 | 1 | 0.998 | 0.929 | 1 | 0.989 | 0.98 | 0.851 | 1 | 1 | 0.9686 |
| 253 | 1 | 1 | 0.99 | 0.992 | 1 | 1 | 0.973 | 0.979 | 0.987 | 1 | 1 | 0.998 | 0.929 | 1 | 0.989 | 0.98 | 0.851 | 1 | 1 | 0.9686 |
| 254 | 1 | 1 | 0.99 | 0.992 | 1 | 1 | 0.973 | 0.979 | 0.987 | 1 | 1 | 0.998 | 0.929 | 1 | 0.989 | 0.98 | 0.851 | 1 | 1 | 0.9686 |
| 255 | 1 | 1 | 0.99 | 0.992 | 1 | 1 | 0.973 | 0.979 | 0.987 | 1 | 1 | 0.998 | 0.929 | 1 | 0.989 | 0.98 | 0.851 | 1 | 1 | 0.9686 |
| 256 | 1 | 1 | 0.99 | 0.992 | 1 | 1 | 0.973 | 0.979 | 0.987 | 1 | 1 | 0.998 | 0.929 | 1 | 0.991 | 0.981 | 0.851 | 1 | 1 | 0.9686 |
| 257 | 1 | 1 | 0.99 | 0.992 | 1 | 1 | 0.973 | 0.979 | 0.987 | 1 | 1 | 0.998 | 0.929 | 1 | 0.992 | 0.982 | 0.851 | 1 | 1 | 0.9686 |
| 258 | 1 | 1 | 0.99 | 0.992 | 1 | 1 | 0.973 | 0.979 | 0.987 | 1 | 1 | 0.998 | 0.929 | 1 | 0.992 | 0.982 | 0.851 | 1 | 1 | 0.9686 |
| 259 | 1 | 1 | 0.99 | 0.992 | 1 | 1 | 0.973 | 0.979 | 0.987 | 1 | 1 | 0.998 | 0.929 | 1 | 0.992 | 0.982 | 0.851 | 1 | 1 | 0.9686 |
| 260 | 1 | 1 | 0.99 | 0.992 | 1 | 1 | 0.973 | 0.979 | 0.987 | 1 | 1 | 0.998 | 0.929 | 1 | 0.992 | 0.982 | 0.851 | 1 | 1 | 0.9686 |
| 261 | 1 | 1 | 0.99 | 0.992 | 1 | 1 | 0.973 | 0.979 | 0.987 | 1 | 1 | 0.998 | 0.929 | 1 | 0.992 | 0.982 | 0.851 | 1 | 1 | 0.9686 |
| 262 | 1 | 1 | 0.993 | 0.994 | 1 | 1 | 0.973 | 0.979 | 0.987 | 1 | 1 | 0.998 | 0.935 | 1 | 0.985 | 0.978 | 0.856 | 1 | 1 | 0.9698 |
| 263 | 1 | 1 | 0.993 | 0.994 | 1 | 1 | 0.973 | 0.979 | 0.987 | 1 | 1 | 0.998 | 0.942 | 1 | 0.985 | 0.979 | 0.856 | 1 | 1 | 0.9698 |
| 264 | 1 | 1 | 0.993 | 0.994 | 1 | 1 | 0.973 | 0.979 | 0.987 | 1 | 1 | 0.998 | 0.935 | 1 | 0.985 | 0.978 | 0.862 | 1 | 1 | 0.9709 |
| 265 | 1 | 1 | 0.993 | 0.994 | 1 | 1 | 0.973 | 0.979 | 0.987 | 1 | 1 | 0.998 | 0.935 | 1 | 0.988 | 0.98 | 0.851 | 1 | 1 | 0.9686 |
| 266 | 1 | 1 | 0.993 | 0.994 | 1 | 1 | 0.973 | 0.979 | 0.987 | 1 | 1 | 0.998 | 0.935 | 1 | 0.989 | 0.981 | 0.851 | 1 | 1 | 0.9686 |
| 267 | 1 | 1 | 0.993 | 0.994 | 1 | 1 | 0.978 | 0.983 | 0.987 | 1 | 1 | 0.998 | 0.929 | 1 | 0.986 | 0.978 | 0.851 | 1 | 1 | 0.9686 |
| 268 | 1 | 1 | 0.993 | 0.994 | 1 | 1 | 0.978 | 0.983 | 0.987 | 1 | 1 | 0.998 | 0.929 | 1 | 0.986 | 0.978 | 0.856 | 1 | 1 | 0.9698 |
| 269 | 1 | 1 | 0.993 | 0.994 | 1 | 1 | 0.978 | 0.983 | 0.987 | 1 | 1 | 0.998 | 0.929 | 1 | 0.986 | 0.978 | 0.862 | 1 | 1 | 0.9709 |
| 270 | 1 | 1 | 0.993 | 0.994 | 1 | 1 | 0.978 | 0.983 | 0.987 | 1 | 1 | 0.998 | 0.929 | 1 | 0.988 | 0.979 | 0.862 | 1 | 1 | 0.9709 |
| 271 | 1 | 1 | 0.993 | 0.994 | 1 | 1 | 0.978 | 0.983 | 0.987 | 1 | 1 | 0.998 | 0.929 | 1 | 0.986 | 0.978 | 0.862 | 1 | 1 | 0.9709 |
| 272 | 1 | 1 | 0.993 | 0.994 | 1 | 1 | 0.978 | 0.983 | 0.987 | 1 | 1 | 0.998 | 0.929 | 1 | 0.986 | 0.978 | 0.862 | 1 | 1 | 0.9709 |
| 273 | 1 | 1 | 0.993 | 0.994 | 1 | 1 | 0.978 | 0.983 | 0.987 | 1 | 1 | 0.998 | 0.929 | 1 | 0.985 | 0.977 | 0.867 | 1 | 1 | 0.972 |
| 274 | 1 | 1 | 0.993 | 0.994 | 1 | 1 | 0.978 | 0.983 | 0.987 | 1 | 1 | 0.998 | 0.929 | 1 | 0.988 | 0.979 | 0.862 | 1 | 1 | 0.9709 |
| 275 | 1 | 1 | 0.993 | 0.994 | 1 | 1 | 0.978 | 0.983 | 0.987 | 1 | 1 | 0.998 | 0.929 | 1 | 0.986 | 0.978 | 0.856 | 1 | 1 | 0.9698 |
| 276 | 1 | 1 | 0.993 | 0.994 | 1 | 1 | 0.978 | 0.983 | 0.987 | 1 | 1 | 0.998 | 0.929 | 1 | 0.986 | 0.978 | 0.862 | 1 | 1 | 0.9709 |
| 277 | 1 | 1 | 0.993 | 0.994 | 1 | 1 | 0.978 | 0.983 | 0.987 | 1 | 1 | 0.998 | 0.929 | 1 | 0.986 | 0.978 | 0.862 | 1 | 1 | 0.9709 |
| 278 | 1 | 1 | 0.993 | 0.994 | 1 | 1 | 0.978 | 0.983 | 0.987 | 1 | 1 | 0.998 | 0.929 | 1 | 0.985 | 0.977 | 0.862 | 1 | 1 | 0.9709 |
| 279 | 1 | 1 | 0.993 | 0.994 | 1 | 1 | 0.978 | 0.983 | 0.987 | 1 | 1 | 0.998 | 0.929 | 1 | 0.985 | 0.977 | 0.862 | 1 | 1 | 0.9709 |
| 280 | 1 | 1 | 0.993 | 0.994 | 1 | 1 | 0.978 | 0.983 | 0.987 | 1 | 1 | 0.998 | 0.929 | 1 | 0.984 | 0.976 | 0.856 | 1 | 1 | 0.9698 |
| 281 | 1 | 1 | 0.993 | 0.994 | 1 | 1 | 0.978 | 0.983 | 0.987 | 1 | 1 | 0.998 | 0.929 | 1 | 0.984 | 0.976 | 0.856 | 1 | 1 | 0.9698 |
| 282 | 1 | 1 | 0.993 | 0.994 | 1 | 1 | 0.978 | 0.983 | 0.987 | 1 | 1 | 0.998 | 0.929 | 1 | 0.984 | 0.976 | 0.856 | 1 | 1 | 0.9698 |
| 283 | 1 | 1 | 0.993 | 0.994 | 1 | 1 | 0.978 | 0.983 | 0.987 | 1 | 1 | 0.998 | 0.929 | 1 | 0.984 | 0.976 | 0.856 | 1 | 1 | 0.9698 |
| 284 | 1 | 1 | 0.993 | 0.994 | 1 | 1 | 0.978 | 0.983 | 0.987 | 1 | 1 | 0.998 | 0.929 | 1 | 0.984 | 0.976 | 0.846 | 1 | 1 | 0.9675 |
| 285 | 1 | 1 | 0.993 | 0.994 | 1 | 1 | 0.978 | 0.983 | 0.987 | 1 | 1 | 0.998 | 0.929 | 1 | 0.984 | 0.976 | 0.862 | 1 | 1 | 0.9709 |
| 286 | 1 | 1 | 0.993 | 0.994 | 1 | 1 | 0.978 | 0.983 | 0.987 | 1 | 1 | 0.998 | 0.929 | 1 | 0.984 | 0.976 | 0.862 | 1 | 1 | 0.9709 |
| 287 | 1 | 1 | 0.993 | 0.994 | 1 | 1 | 0.978 | 0.983 | 0.987 | 1 | 1 | 0.998 | 0.929 | 1 | 0.985 | 0.977 | 0.856 | 1 | 1 | 0.9698 |
| 288 | 1 | 1 | 0.993 | 0.994 | 1 | 1 | 0.978 | 0.983 | 0.987 | 1 | 1 | 0.998 | 0.929 | 1 | 0.985 | 0.977 | 0.856 | 1 | 1 | 0.9698 |
| 289 | 1 | 1 | 0.993 | 0.994 | 1 | 1 | 0.978 | 0.983 | 0.987 | 1 | 1 | 0.998 | 0.929 | 1 | 0.985 | 0.977 | 0.856 | 1 | 1 | 0.9698 |
| 290 | 1 | 1 | 0.993 | 0.994 | 1 | 1 | 0.978 | 0.983 | 0.987 | 1 | 1 | 0.998 | 0.929 | 1 | 0.985 | 0.977 | 0.856 | 1 | 1 | 0.9698 |
| 291 | 1 | 1 | 0.993 | 0.994 | 1 | 1 | 0.978 | 0.983 | 0.987 | 1 | 1 | 0.998 | 0.929 | 1 | 0.985 | 0.977 | 0.856 | 1 | 1 | 0.9698 |
| 292 | 1 | 1 | 0.993 | 0.994 | 1 | 1 | 0.978 | 0.983 | 0.987 | 1 | 1 | 0.998 | 0.929 | 1 | 0.985 | 0.977 | 0.851 | 1 | 1 | 0.9686 |
| 293 | 1 | 1 | 0.993 | 0.994 | 1 | 1 | 0.978 | 0.983 | 0.987 | 1 | 1 | 0.998 | 0.929 | 1 | 0.985 | 0.977 | 0.856 | 1 | 1 | 0.9698 |
| 294 | 1 | 1 | 0.993 | 0.994 | 1 | 1 | 0.978 | 0.983 | 0.987 | 1 | 1 | 0.998 | 0.929 | 1 | 0.984 | 0.976 | 0.856 | 1 | 1 | 0.9698 |
| 295 | 1 | 1 | 0.993 | 0.994 | 1 | 1 | 0.975 | 0.981 | 0.987 | 1 | 1 | 0.998 | 0.929 | 1 | 0.984 | 0.976 | 0.856 | 1 | 1 | 0.9698 |
| 296 | 1 | 1 | 0.993 | 0.994 | 1 | 1 | 0.975 | 0.981 | 0.987 | 1 | 1 | 0.998 | 0.929 | 1 | 0.984 | 0.976 | 0.856 | 1 | 1 | 0.9698 |
| 297 | 1 | 1 | 0.993 | 0.994 | 1 | 1 | 0.968 | 0.975 | 0.987 | 1 | 1 | 0.998 | 0.922 | 1 | 0.981 | 0.973 | 0.851 | 1 | 1 | 0.9686 |
| 298 | 1 | 1 | 0.993 | 0.994 | 1 | 1 | 0.968 | 0.975 | 0.987 | 1 | 1 | 0.998 | 0.922 | 1 | 0.978 | 0.97 | 0.851 | 1 | 1 | 0.9686 |
| 299 | 1 | 1 | 0.993 | 0.994 | 1 | 1 | 0.965 | 0.974 | 0.987 | 1 | 1 | 0.998 | 0.922 | 1 | 0.978 | 0.97 | 0.851 | 1 | 1 | 0.9686 |
| 300 | 1 | 1 | 0.993 | 0.994 | 1 | 1 | 0.968 | 0.975 | 0.987 | 1 | 1 | 0.998 | 0.922 | 1 | 0.98 | 0.971 | 0.851 | 1 | 1 | 0.9686 |
| 301 | 1 | 1 | 0.993 | 0.994 | 1 | 1 | 0.965 | 0.974 | 0.987 | 1 | 1 | 0.998 | 0.922 | 1 | 0.98 | 0.971 | 0.851 | 1 | 1 | 0.9686 |
| 302 | 1 | 1 | 0.993 | 0.994 | 1 | 1 | 0.965 | 0.974 | 0.987 | 1 | 1 | 0.998 | 0.929 | 1 | 0.98 | 0.973 | 0.851 | 1 | 1 | 0.9686 |
| 303 | 1 | 1 | 0.993 | 0.994 | 1 | 1 | 0.965 | 0.974 | 0.987 | 1 | 1 | 0.998 | 0.922 | 1 | 0.98 | 0.971 | 0.851 | 1 | 1 | 0.9686 |
| 304 | 1 | 1 | 0.993 | 0.994 | 1 | 1 | 0.965 | 0.974 | 0.987 | 1 | 1 | 0.998 | 0.929 | 1 | 0.978 | 0.971 | 0.851 | 1 | 1 | 0.9686 |
| 305 | 1 | 1 | 0.993 | 0.994 | 1 | 1 | 0.965 | 0.974 | 0.987 | 1 | 1 | 0.998 | 0.929 | 1 | 0.978 | 0.971 | 0.851 | 1 | 1 | 0.9686 |
| 306 | 1 | 1 | 0.993 | 0.994 | 1 | 1 | 0.965 | 0.974 | 0.987 | 1 | 1 | 0.998 | 0.929 | 1 | 0.978 | 0.971 | 0.851 | 1 | 1 | 0.9686 |
| 307 | 1 | 1 | 0.993 | 0.994 | 1 | 1 | 0.965 | 0.974 | 0.987 | 1 | 1 | 0.998 | 0.929 | 1 | 0.978 | 0.971 | 0.851 | 1 | 1 | 0.9686 |
| 308 | 1 | 1 | 0.993 | 0.994 | 1 | 1 | 0.965 | 0.974 | 0.987 | 1 | 1 | 0.998 | 0.935 | 1 | 0.978 | 0.973 | 0.851 | 1 | 1 | 0.9686 |
| 309 | 1 | 1 | 0.993 | 0.994 | 1 | 1 | 0.965 | 0.974 | 0.987 | 1 | 1 | 0.998 | 0.929 | 1 | 0.978 | 0.971 | 0.851 | 1 | 1 | 0.9686 |
| 310 | 1 | 1 | 0.993 | 0.994 | 1 | 1 | 0.965 | 0.974 | 0.987 | 1 | 1 | 0.998 | 0.935 | 1 | 0.978 | 0.973 | 0.851 | 1 | 1 | 0.9686 |
| 311 | 1 | 1 | 0.993 | 0.994 | 1 | 1 | 0.965 | 0.974 | 0.987 | 1 | 1 | 0.998 | 0.929 | 1 | 0.978 | 0.971 | 0.851 | 1 | 1 | 0.9686 |
| 312 | 1 | 1 | 0.993 | 0.994 | 1 | 1 | 0.968 | 0.975 | 0.987 | 1 | 1 | 0.998 | 0.929 | 1 | 0.978 | 0.971 | 0.851 | 1 | 1 | 0.9686 |
| 313 | 1 | 1 | 0.993 | 0.994 | 1 | 1 | 0.968 | 0.975 | 0.987 | 1 | 1 | 0.998 | 0.929 | 1 | 0.978 | 0.971 | 0.851 | 1 | 1 | 0.9686 |
| 314 | 1 | 1 | 0.993 | 0.994 | 1 | 1 | 0.968 | 0.975 | 0.987 | 1 | 1 | 0.998 | 0.929 | 1 | 0.978 | 0.971 | 0.851 | 1 | 1 | 0.9686 |
| 315 | 1 | 1 | 0.993 | 0.994 | 1 | 1 | 0.968 | 0.975 | 0.987 | 1 | 1 | 0.998 | 0.929 | 1 | 0.978 | 0.971 | 0.851 | 1 | 1 | 0.9686 |
| 316 | 1 | 1 | 0.993 | 0.994 | 1 | 1 | 0.968 | 0.975 | 0.987 | 1 | 1 | 0.998 | 0.929 | 1 | 0.978 | 0.971 | 0.851 | 1 | 1 | 0.9686 |
| 317 | 1 | 1 | 0.993 | 0.994 | 1 | 1 | 0.968 | 0.975 | 0.987 | 1 | 1 | 0.998 | 0.929 | 1 | 0.978 | 0.971 | 0.851 | 1 | 1 | 0.9686 |
| 318 | 1 | 1 | 0.993 | 0.994 | 1 | 1 | 0.968 | 0.975 | 0.987 | 1 | 1 | 0.998 | 0.929 | 1 | 0.978 | 0.971 | 0.851 | 1 | 1 | 0.9686 |
| 319 | 1 | 1 | 0.993 | 0.994 | 1 | 1 | 0.968 | 0.975 | 0.987 | 1 | 1 | 0.998 | 0.929 | 1 | 0.978 | 0.971 | 0.851 | 1 | 1 | 0.9686 |
| 320 | 1 | 1 | 0.993 | 0.994 | 1 | 1 | 0.968 | 0.975 | 0.987 | 1 | 1 | 0.998 | 0.929 | 1 | 0.978 | 0.971 | 0.851 | 1 | 1 | 0.9686 |
| 321 | 1 | 1 | 0.993 | 0.994 | 1 | 1 | 0.973 | 0.979 | 0.987 | 1 | 1 | 0.998 | 0.929 | 1 | 0.98 | 0.973 | 0.846 | 1 | 1 | 0.9675 |
| 322 | 1 | 1 | 0.993 | 0.994 | 1 | 1 | 0.973 | 0.979 | 0.987 | 1 | 1 | 0.998 | 0.929 | 1 | 0.98 | 0.973 | 0.846 | 1 | 1 | 0.9675 |
| 323 | 1 | 1 | 0.993 | 0.994 | 1 | 1 | 0.973 | 0.979 | 0.987 | 1 | 1 | 0.998 | 0.922 | 1 | 0.98 | 0.971 | 0.846 | 1 | 1 | 0.9675 |
| 324 | 1 | 1 | 0.993 | 0.994 | 1 | 1 | 0.97 | 0.977 | 0.987 | 1 | 1 | 0.998 | 0.922 | 1 | 0.981 | 0.973 | 0.846 | 1 | 1 | 0.9675 |
| 325 | 1 | 1 | 0.993 | 0.994 | 1 | 1 | 0.97 | 0.977 | 0.987 | 1 | 1 | 0.998 | 0.922 | 1 | 0.978 | 0.97 | 0.851 | 1 | 1 | 0.9686 |
| 326 | 1 | 1 | 0.993 | 0.994 | 1 | 1 | 0.97 | 0.977 | 0.987 | 1 | 1 | 0.998 | 0.922 | 1 | 0.976 | 0.968 | 0.851 | 1 | 1 | 0.9686 |
| 327 | 1 | 1 | 0.993 | 0.994 | 1 | 1 | 0.97 | 0.977 | 0.987 | 1 | 1 | 0.998 | 0.929 | 1 | 0.976 | 0.969 | 0.846 | 1 | 1 | 0.9675 |
| 328 | 1 | 1 | 0.993 | 0.994 | 1 | 1 | 0.97 | 0.977 | 0.987 | 1 | 1 | 0.998 | 0.929 | 1 | 0.976 | 0.969 | 0.846 | 1 | 1 | 0.9675 |
| 329 | 1 | 1 | 0.993 | 0.994 | 1 | 1 | 0.97 | 0.977 | 0.987 | 1 | 1 | 0.998 | 0.929 | 1 | 0.977 | 0.97 | 0.846 | 1 | 1 | 0.9675 |
| 330 | 1 | 1 | 0.993 | 0.994 | 1 | 1 | 0.97 | 0.977 | 0.987 | 1 | 1 | 0.998 | 0.929 | 1 | 0.977 | 0.97 | 0.846 | 1 | 1 | 0.9675 |
| 331 | 1 | 1 | 0.997 | 0.997 | 1 | 1 | 0.97 | 0.977 | 0.987 | 1 | 1 | 0.998 | 0.929 | 1 | 0.976 | 0.969 | 0.846 | 1 | 1 | 0.9675 |
| 332 | 1 | 1 | 0.993 | 0.994 | 1 | 1 | 0.97 | 0.977 | 0.987 | 1 | 1 | 0.998 | 0.929 | 1 | 0.976 | 0.969 | 0.846 | 1 | 1 | 0.9675 |
| 333 | 1 | 1 | 0.993 | 0.994 | 1 | 1 | 0.97 | 0.977 | 0.987 | 1 | 1 | 0.998 | 0.929 | 1 | 0.976 | 0.969 | 0.846 | 1 | 1 | 0.9675 |
| 334 | 1 | 1 | 0.993 | 0.994 | 1 | 1 | 0.97 | 0.977 | 0.987 | 1 | 1 | 0.998 | 0.929 | 1 | 0.976 | 0.969 | 0.846 | 1 | 1 | 0.9675 |
| 335 | 1 | 1 | 0.993 | 0.994 | 1 | 1 | 0.968 | 0.975 | 0.987 | 1 | 1 | 0.998 | 0.929 | 1 | 0.976 | 0.969 | 0.846 | 1 | 1 | 0.9675 |
| 336 | 1 | 1 | 0.993 | 0.994 | 1 | 1 | 0.968 | 0.975 | 0.987 | 1 | 1 | 0.998 | 0.929 | 1 | 0.976 | 0.969 | 0.846 | 1 | 1 | 0.9675 |
| 337 | 1 | 1 | 0.993 | 0.994 | 1 | 1 | 0.968 | 0.975 | 0.987 | 1 | 1 | 0.998 | 0.929 | 1 | 0.976 | 0.969 | 0.846 | 1 | 1 | 0.9675 |
| 338 | 1 | 1 | 0.993 | 0.994 | 1 | 1 | 0.968 | 0.975 | 0.987 | 1 | 1 | 0.998 | 0.929 | 1 | 0.976 | 0.969 | 0.846 | 1 | 1 | 0.9675 |
| 339 | 1 | 1 | 0.993 | 0.994 | 1 | 1 | 0.968 | 0.975 | 0.987 | 1 | 1 | 0.998 | 0.929 | 1 | 0.976 | 0.969 | 0.846 | 1 | 1 | 0.9675 |
| 340 | 1 | 1 | 0.993 | 0.994 | 1 | 1 | 0.968 | 0.975 | 0.987 | 1 | 1 | 0.998 | 0.929 | 1 | 0.974 | 0.968 | 0.84 | 1 | 1 | 0.9664 |
| 341 | 1 | 1 | 0.993 | 0.994 | 1 | 1 | 0.968 | 0.975 | 0.987 | 1 | 1 | 0.998 | 0.929 | 1 | 0.974 | 0.968 | 0.84 | 1 | 1 | 0.9664 |
| 342 | 1 | 1 | 0.993 | 0.994 | 1 | 1 | 0.968 | 0.975 | 0.987 | 1 | 1 | 0.998 | 0.929 | 1 | 0.976 | 0.969 | 0.84 | 1 | 1 | 0.9664 |
| 343 | 1 | 1 | 0.993 | 0.994 | 1 | 1 | 0.968 | 0.975 | 0.987 | 1 | 1 | 0.998 | 0.929 | 1 | 0.974 | 0.968 | 0.84 | 1 | 1 | 0.9664 |
| 344 | 1 | 1 | 0.993 | 0.994 | 1 | 1 | 0.968 | 0.975 | 0.987 | 1 | 1 | 0.998 | 0.929 | 1 | 0.974 | 0.968 | 0.84 | 1 | 1 | 0.9664 |
| 345 | 1 | 1 | 0.993 | 0.994 | 1 | 1 | 0.968 | 0.975 | 0.987 | 1 | 1 | 0.998 | 0.929 | 1 | 0.974 | 0.968 | 0.84 | 1 | 1 | 0.9664 |
| 346 | 1 | 1 | 0.993 | 0.994 | 1 | 1 | 0.968 | 0.975 | 0.987 | 1 | 1 | 0.998 | 0.929 | 1 | 0.974 | 0.968 | 0.84 | 1 | 1 | 0.9664 |
| 347 | 1 | 1 | 0.993 | 0.994 | 1 | 1 | 0.968 | 0.975 | 0.987 | 1 | 1 | 0.998 | 0.929 | 1 | 0.974 | 0.968 | 0.84 | 1 | 1 | 0.9664 |
| 348 | 1 | 1 | 0.993 | 0.994 | 1 | 1 | 0.968 | 0.975 | 0.987 | 1 | 1 | 0.998 | 0.929 | 1 | 0.974 | 0.968 | 0.846 | 1 | 1 | 0.9675 |
| 349 | 1 | 1 | 0.993 | 0.994 | 1 | 1 | 0.968 | 0.975 | 0.987 | 1 | 1 | 0.998 | 0.929 | 1 | 0.977 | 0.97 | 0.846 | 1 | 1 | 0.9675 |
| 350 | 1 | 1 | 0.993 | 0.994 | 1 | 1 | 0.968 | 0.975 | 0.987 | 1 | 1 | 0.998 | 0.929 | 1 | 0.977 | 0.97 | 0.835 | 1 | 1 | 0.9653 |
| 351 | 1 | 1 | 0.993 | 0.994 | 1 | 1 | 0.968 | 0.975 | 0.987 | 1 | 1 | 0.998 | 0.929 | 1 | 0.977 | 0.97 | 0.835 | 1 | 1 | 0.9653 |
| 352 | 1 | 1 | 0.993 | 0.994 | 1 | 1 | 0.968 | 0.975 | 0.987 | 1 | 1 | 0.998 | 0.922 | 1 | 0.977 | 0.969 | 0.835 | 1 | 1 | 0.9653 |
| 353 | 1 | 1 | 0.993 | 0.994 | 1 | 1 | 0.968 | 0.975 | 0.987 | 1 | 1 | 0.998 | 0.922 | 1 | 0.977 | 0.969 | 0.835 | 1 | 1 | 0.9653 |
| 354 | 1 | 1 | 0.993 | 0.994 | 1 | 1 | 0.968 | 0.975 | 0.987 | 1 | 1 | 0.998 | 0.922 | 1 | 0.977 | 0.969 | 0.835 | 1 | 1 | 0.9653 |
| 355 | 1 | 1 | 0.993 | 0.994 | 1 | 1 | 0.968 | 0.975 | 0.987 | 1 | 1 | 0.998 | 0.922 | 1 | 0.977 | 0.969 | 0.835 | 1 | 1 | 0.9653 |
| 356 | 1 | 1 | 0.993 | 0.994 | 1 | 1 | 0.968 | 0.975 | 0.987 | 1 | 1 | 0.998 | 0.922 | 1 | 0.977 | 0.969 | 0.835 | 1 | 1 | 0.9653 |
| 357 | 1 | 1 | 0.993 | 0.994 | 1 | 1 | 0.968 | 0.975 | 0.987 | 1 | 1 | 0.998 | 0.922 | 1 | 0.977 | 0.969 | 0.835 | 1 | 1 | 0.9653 |
| 358 | 1 | 1 | 0.993 | 0.994 | 1 | 1 | 0.968 | 0.975 | 0.987 | 1 | 1 | 0.998 | 0.922 | 1 | 0.977 | 0.969 | 0.835 | 1 | 1 | 0.9653 |
| 359 | 1 | 1 | 0.993 | 0.994 | 1 | 1 | 0.968 | 0.975 | 0.987 | 1 | 1 | 0.998 | 0.922 | 1 | 0.977 | 0.969 | 0.835 | 1 | 1 | 0.9653 |
| 360 | 1 | 1 | 0.993 | 0.994 | 1 | 1 | 0.968 | 0.975 | 0.987 | 1 | 1 | 0.998 | 0.922 | 1 | 0.978 | 0.97 | 0.835 | 1 | 1 | 0.9653 |
| 361 | 1 | 1 | 0.993 | 0.994 | 1 | 1 | 0.968 | 0.975 | 0.987 | 1 | 1 | 0.998 | 0.929 | 1 | 0.978 | 0.971 | 0.835 | 1 | 1 | 0.9653 |
| 362 | 1 | 1 | 0.993 | 0.994 | 1 | 1 | 0.968 | 0.975 | 0.987 | 1 | 1 | 0.998 | 0.929 | 1 | 0.977 | 0.97 | 0.835 | 1 | 1 | 0.9653 |
| 363 | 1 | 1 | 0.993 | 0.994 | 1 | 1 | 0.968 | 0.975 | 0.987 | 1 | 1 | 0.998 | 0.929 | 1 | 0.978 | 0.971 | 0.84 | 1 | 1 | 0.9664 |
| 364 | 1 | 1 | 0.993 | 0.994 | 1 | 1 | 0.968 | 0.975 | 0.987 | 1 | 1 | 0.998 | 0.929 | 1 | 0.977 | 0.97 | 0.835 | 1 | 1 | 0.9653 |
| 365 | 1 | 1 | 0.993 | 0.994 | 1 | 1 | 0.968 | 0.975 | 0.987 | 1 | 1 | 0.998 | 0.929 | 1 | 0.977 | 0.97 | 0.835 | 1 | 1 | 0.9653 |
| 366 | 1 | 1 | 0.993 | 0.994 | 1 | 1 | 0.968 | 0.975 | 0.987 | 1 | 1 | 0.998 | 0.929 | 1 | 0.977 | 0.97 | 0.835 | 1 | 1 | 0.9653 |
| 367 | 1 | 1 | 0.993 | 0.994 | 1 | 1 | 0.968 | 0.975 | 0.987 | 1 | 1 | 0.998 | 0.929 | 1 | 0.977 | 0.97 | 0.835 | 1 | 1 | 0.9653 |
| 368 | 1 | 1 | 0.993 | 0.994 | 1 | 1 | 0.968 | 0.975 | 0.987 | 1 | 1 | 0.998 | 0.929 | 1 | 0.976 | 0.969 | 0.835 | 1 | 1 | 0.9653 |
| 369 | 1 | 1 | 0.993 | 0.994 | 1 | 1 | 0.968 | 0.975 | 0.987 | 1 | 1 | 0.998 | 0.929 | 1 | 0.976 | 0.969 | 0.835 | 1 | 1 | 0.9653 |
| 370 | 1 | 1 | 0.993 | 0.994 | 1 | 1 | 0.968 | 0.975 | 0.987 | 1 | 1 | 0.998 | 0.929 | 1 | 0.976 | 0.969 | 0.84 | 1 | 1 | 0.9664 |
| 371 | 1 | 1 | 0.993 | 0.994 | 1 | 1 | 0.968 | 0.975 | 0.987 | 1 | 1 | 0.998 | 0.929 | 1 | 0.977 | 0.97 | 0.84 | 1 | 1 | 0.9664 |
| 372 | 1 | 1 | 0.993 | 0.994 | 1 | 1 | 0.968 | 0.975 | 0.987 | 1 | 1 | 0.998 | 0.929 | 1 | 0.977 | 0.97 | 0.84 | 1 | 1 | 0.9664 |
| 373 | 1 | 1 | 0.993 | 0.994 | 1 | 1 | 0.968 | 0.975 | 0.987 | 1 | 1 | 0.998 | 0.929 | 1 | 0.976 | 0.969 | 0.84 | 1 | 1 | 0.9664 |
| 374 | 1 | 1 | 0.993 | 0.994 | 1 | 1 | 0.963 | 0.972 | 0.987 | 1 | 1 | 0.998 | 0.929 | 1 | 0.976 | 0.969 | 0.84 | 1 | 1 | 0.9664 |
| 375 | 1 | 1 | 0.993 | 0.994 | 1 | 1 | 0.963 | 0.972 | 0.987 | 1 | 1 | 0.998 | 0.929 | 1 | 0.976 | 0.969 | 0.84 | 1 | 1 | 0.9664 |
| 376 | 1 | 1 | 0.993 | 0.994 | 1 | 1 | 0.963 | 0.972 | 0.987 | 1 | 1 | 0.998 | 0.929 | 1 | 0.976 | 0.969 | 0.84 | 1 | 1 | 0.9664 |
| 377 | 1 | 1 | 0.993 | 0.994 | 1 | 1 | 0.963 | 0.972 | 0.987 | 1 | 1 | 0.998 | 0.929 | 1 | 0.976 | 0.969 | 0.84 | 1 | 1 | 0.9664 |
| 378 | 1 | 1 | 0.993 | 0.994 | 1 | 1 | 0.963 | 0.972 | 0.987 | 1 | 1 | 0.998 | 0.929 | 1 | 0.976 | 0.969 | 0.835 | 1 | 1 | 0.9653 |
| 379 | 1 | 1 | 0.993 | 0.994 | 1 | 1 | 0.963 | 0.972 | 0.987 | 1 | 1 | 0.998 | 0.929 | 1 | 0.976 | 0.969 | 0.84 | 1 | 1 | 0.9664 |
| 380 | 1 | 1 | 0.993 | 0.994 | 1 | 1 | 0.96 | 0.97 | 0.987 | 1 | 1 | 0.998 | 0.929 | 1 | 0.974 | 0.968 | 0.84 | 1 | 1 | 0.9664 |
| 381 | 1 | 1 | 0.993 | 0.994 | 1 | 1 | 0.958 | 0.968 | 0.987 | 1 | 1 | 0.998 | 0.929 | 1 | 0.974 | 0.968 | 0.84 | 1 | 1 | 0.9664 |
| 382 | 1 | 1 | 0.993 | 0.994 | 1 | 1 | 0.958 | 0.968 | 0.987 | 1 | 1 | 0.998 | 0.929 | 1 | 0.974 | 0.968 | 0.84 | 1 | 1 | 0.9664 |
| 383 | 1 | 1 | 0.993 | 0.994 | 1 | 1 | 0.958 | 0.968 | 0.987 | 1 | 1 | 0.998 | 0.929 | 1 | 0.974 | 0.968 | 0.84 | 1 | 1 | 0.9664 |
| 384 | 1 | 1 | 0.993 | 0.994 | 1 | 1 | 0.958 | 0.968 | 0.987 | 1 | 1 | 0.998 | 0.929 | 1 | 0.974 | 0.968 | 0.84 | 1 | 1 | 0.9664 |
| 385 | 1 | 1 | 0.993 | 0.994 | 1 | 1 | 0.958 | 0.968 | 0.987 | 1 | 1 | 0.998 | 0.929 | 1 | 0.974 | 0.968 | 0.84 | 1 | 1 | 0.9664 |
| 386 | 1 | 1 | 0.993 | 0.994 | 1 | 1 | 0.955 | 0.966 | 0.987 | 1 | 1 | 0.998 | 0.929 | 1 | 0.974 | 0.968 | 0.84 | 1 | 1 | 0.9664 |
| 387 | 1 | 1 | 0.993 | 0.994 | 1 | 1 | 0.955 | 0.966 | 0.987 | 1 | 1 | 0.998 | 0.929 | 1 | 0.974 | 0.968 | 0.84 | 1 | 1 | 0.9664 |
| 388 | 1 | 1 | 0.993 | 0.994 | 1 | 1 | 0.955 | 0.966 | 0.987 | 1 | 1 | 0.998 | 0.929 | 1 | 0.976 | 0.969 | 0.835 | 1 | 1 | 0.9653 |
| 389 | 1 | 1 | 0.993 | 0.994 | 1 | 1 | 0.955 | 0.966 | 0.987 | 1 | 1 | 0.998 | 0.929 | 1 | 0.976 | 0.969 | 0.835 | 1 | 1 | 0.9653 |
| 390 | 1 | 1 | 0.993 | 0.994 | 1 | 1 | 0.958 | 0.968 | 0.987 | 1 | 1 | 0.998 | 0.929 | 1 | 0.976 | 0.969 | 0.835 | 1 | 1 | 0.9653 |
| 391 | 1 | 1 | 0.993 | 0.994 | 1 | 1 | 0.958 | 0.968 | 0.987 | 1 | 1 | 0.998 | 0.929 | 1 | 0.976 | 0.969 | 0.835 | 1 | 1 | 0.9653 |
| 392 | 1 | 1 | 0.993 | 0.994 | 1 | 1 | 0.953 | 0.964 | 0.987 | 1 | 1 | 0.998 | 0.929 | 1 | 0.976 | 0.969 | 0.835 | 1 | 1 | 0.9653 |
| 393 | 1 | 1 | 0.993 | 0.994 | 1 | 1 | 0.953 | 0.964 | 0.987 | 1 | 1 | 0.998 | 0.929 | 1 | 0.976 | 0.969 | 0.83 | 1 | 1 | 0.9642 |
| 394 | 1 | 1 | 0.993 | 0.994 | 1 | 1 | 0.953 | 0.964 | 0.987 | 1 | 1 | 0.998 | 0.929 | 1 | 0.976 | 0.969 | 0.824 | 1 | 1 | 0.963 |
| 395 | 1 | 1 | 0.993 | 0.994 | 1 | 1 | 0.953 | 0.964 | 0.987 | 1 | 1 | 0.998 | 0.929 | 1 | 0.976 | 0.969 | 0.83 | 1 | 1 | 0.9642 |
| 396 | 1 | 1 | 0.993 | 0.994 | 1 | 1 | 0.953 | 0.964 | 0.987 | 1 | 1 | 0.998 | 0.929 | 1 | 0.976 | 0.969 | 0.824 | 1 | 1 | 0.963 |
| 397 | 1 | 1 | 0.993 | 0.994 | 1 | 1 | 0.953 | 0.964 | 0.987 | 1 | 1 | 0.998 | 0.929 | 1 | 0.976 | 0.969 | 0.83 | 1 | 1 | 0.9642 |
| 398 | 1 | 1 | 0.993 | 0.994 | 1 | 1 | 0.953 | 0.964 | 0.987 | 1 | 1 | 0.998 | 0.929 | 1 | 0.976 | 0.969 | 0.83 | 1 | 1 | 0.9642 |
| 399 | 1 | 1 | 0.993 | 0.994 | 1 | 1 | 0.953 | 0.964 | 0.987 | 1 | 1 | 0.998 | 0.929 | 1 | 0.976 | 0.969 | 0.83 | 1 | 1 | 0.9642 |
| 400 | 1 | 1 | 0.993 | 0.994 | 1 | 1 | 0.953 | 0.964 | 0.987 | 1 | 1 | 0.998 | 0.929 | 1 | 0.977 | 0.97 | 0.83 | 1 | 1 | 0.9642 |
| 401 | 1 | 1 | 0.993 | 0.994 | 1 | 1 | 0.953 | 0.964 | 0.987 | 1 | 1 | 0.998 | 0.929 | 1 | 0.977 | 0.97 | 0.83 | 1 | 1 | 0.9642 |
| 402 | 1 | 1 | 0.993 | 0.994 | 1 | 1 | 0.95 | 0.962 | 0.987 | 1 | 1 | 0.998 | 0.929 | 1 | 0.976 | 0.969 | 0.835 | 1 | 1 | 0.9653 |
| 403 | 1 | 1 | 0.993 | 0.994 | 1 | 1 | 0.95 | 0.962 | 0.987 | 1 | 1 | 0.998 | 0.929 | 1 | 0.976 | 0.969 | 0.835 | 1 | 1 | 0.9653 |
| 404 | 1 | 1 | 0.993 | 0.994 | 1 | 1 | 0.95 | 0.962 | 0.987 | 1 | 1 | 0.998 | 0.929 | 1 | 0.976 | 0.969 | 0.835 | 1 | 1 | 0.9653 |
| 405 | 1 | 1 | 0.993 | 0.994 | 1 | 1 | 0.95 | 0.962 | 0.987 | 1 | 1 | 0.998 | 0.929 | 1 | 0.976 | 0.969 | 0.835 | 1 | 1 | 0.9653 |
| 406 | 1 | 1 | 0.993 | 0.994 | 1 | 1 | 0.95 | 0.962 | 0.987 | 1 | 1 | 0.998 | 0.929 | 1 | 0.976 | 0.969 | 0.83 | 1 | 1 | 0.9642 |
| 407 | 1 | 1 | 0.993 | 0.994 | 1 | 1 | 0.95 | 0.962 | 0.987 | 1 | 1 | 0.998 | 0.929 | 1 | 0.974 | 0.968 | 0.835 | 1 | 1 | 0.9653 |
| 408 | 1 | 1 | 0.993 | 0.994 | 1 | 1 | 0.95 | 0.962 | 0.987 | 1 | 1 | 0.998 | 0.929 | 1 | 0.974 | 0.968 | 0.83 | 1 | 1 | 0.9642 |
| 409 | 1 | 1 | 0.993 | 0.994 | 1 | 1 | 0.95 | 0.962 | 0.987 | 1 | 1 | 0.998 | 0.929 | 1 | 0.974 | 0.968 | 0.83 | 1 | 1 | 0.9642 |
| 410 | 1 | 1 | 0.993 | 0.994 | 1 | 1 | 0.95 | 0.962 | 0.987 | 1 | 1 | 0.998 | 0.929 | 1 | 0.976 | 0.969 | 0.83 | 1 | 1 | 0.9642 |
| 411 | 1 | 1 | 0.993 | 0.994 | 1 | 1 | 0.95 | 0.962 | 0.987 | 1 | 1 | 0.998 | 0.929 | 1 | 0.976 | 0.969 | 0.83 | 1 | 1 | 0.9642 |
| 412 | 1 | 1 | 0.993 | 0.994 | 1 | 1 | 0.95 | 0.962 | 0.987 | 1 | 1 | 0.998 | 0.929 | 1 | 0.973 | 0.967 | 0.83 | 1 | 1 | 0.9642 |
| 413 | 1 | 1 | 0.993 | 0.994 | 1 | 1 | 0.95 | 0.962 | 0.987 | 1 | 1 | 0.998 | 0.929 | 1 | 0.973 | 0.967 | 0.83 | 1 | 1 | 0.9642 |
| 414 | 1 | 1 | 0.993 | 0.994 | 1 | 1 | 0.95 | 0.962 | 0.987 | 1 | 1 | 0.998 | 0.929 | 1 | 0.973 | 0.967 | 0.83 | 1 | 1 | 0.9642 |
| 415 | 1 | 1 | 0.993 | 0.994 | 1 | 1 | 0.95 | 0.962 | 0.987 | 1 | 1 | 0.998 | 0.929 | 1 | 0.973 | 0.967 | 0.835 | 1 | 1 | 0.9653 |
| 416 | 1 | 1 | 0.993 | 0.994 | 1 | 1 | 0.95 | 0.962 | 0.987 | 1 | 1 | 0.998 | 0.929 | 1 | 0.973 | 0.967 | 0.835 | 1 | 1 | 0.9653 |
| 417 | 1 | 1 | 0.993 | 0.994 | 1 | 1 | 0.95 | 0.962 | 0.987 | 1 | 1 | 0.998 | 0.929 | 1 | 0.973 | 0.967 | 0.835 | 1 | 1 | 0.9653 |
| 418 | 1 | 1 | 0.993 | 0.994 | 1 | 1 | 0.95 | 0.962 | 0.987 | 1 | 1 | 0.998 | 0.929 | 1 | 0.973 | 0.967 | 0.835 | 1 | 1 | 0.9653 |
| 419 | 1 | 1 | 0.993 | 0.994 | 1 | 1 | 0.95 | 0.962 | 0.987 | 1 | 1 | 0.998 | 0.929 | 1 | 0.973 | 0.967 | 0.835 | 1 | 1 | 0.9653 |
| 420 | 1 | 1 | 0.993 | 0.994 | 1 | 1 | 0.953 | 0.964 | 0.987 | 1 | 1 | 0.998 | 0.929 | 1 | 0.976 | 0.969 | 0.83 | 1 | 1 | 0.9642 |
| 421 | 1 | 1 | 0.993 | 0.994 | 1 | 1 | 0.953 | 0.964 | 0.987 | 1 | 1 | 0.998 | 0.929 | 1 | 0.977 | 0.97 | 0.83 | 1 | 1 | 0.9642 |
| 422 | 1 | 1 | 0.993 | 0.994 | 1 | 1 | 0.95 | 0.962 | 0.987 | 1 | 1 | 0.998 | 0.922 | 1 | 0.977 | 0.969 | 0.819 | 1 | 1 | 0.9619 |
| 423 | 1 | 1 | 0.993 | 0.994 | 1 | 1 | 0.953 | 0.964 | 0.987 | 1 | 1 | 0.998 | 0.922 | 1 | 0.977 | 0.969 | 0.819 | 1 | 1 | 0.9619 |
| 424 | 1 | 1 | 0.993 | 0.994 | 1 | 1 | 0.953 | 0.964 | 0.987 | 1 | 1 | 0.998 | 0.922 | 1 | 0.977 | 0.969 | 0.819 | 1 | 1 | 0.9619 |
| 425 | 1 | 1 | 0.993 | 0.994 | 1 | 1 | 0.953 | 0.964 | 0.987 | 1 | 1 | 0.998 | 0.929 | 1 | 0.976 | 0.969 | 0.819 | 1 | 1 | 0.9619 |
| 426 | 1 | 1 | 0.993 | 0.994 | 1 | 1 | 0.953 | 0.964 | 0.987 | 1 | 1 | 0.998 | 0.929 | 1 | 0.977 | 0.97 | 0.814 | 1 | 1 | 0.9608 |
| 427 | 1 | 1 | 0.993 | 0.994 | 1 | 1 | 0.953 | 0.964 | 0.987 | 1 | 1 | 0.998 | 0.929 | 1 | 0.977 | 0.97 | 0.814 | 1 | 1 | 0.9608 |
| 428 | 1 | 1 | 0.993 | 0.994 | 1 | 1 | 0.953 | 0.964 | 0.987 | 1 | 1 | 0.998 | 0.929 | 1 | 0.977 | 0.97 | 0.814 | 1 | 1 | 0.9608 |
| 429 | 1 | 1 | 0.993 | 0.994 | 1 | 1 | 0.953 | 0.964 | 0.987 | 1 | 1 | 0.998 | 0.929 | 1 | 0.977 | 0.97 | 0.814 | 1 | 1 | 0.9608 |
| 430 | 1 | 1 | 0.993 | 0.994 | 1 | 1 | 0.948 | 0.96 | 0.987 | 1 | 1 | 0.998 | 0.922 | 1 | 0.978 | 0.97 | 0.809 | 1 | 1 | 0.9597 |
| 431 | 1 | 1 | 0.993 | 0.994 | 1 | 1 | 0.948 | 0.96 | 0.987 | 1 | 1 | 0.998 | 0.922 | 1 | 0.978 | 0.97 | 0.809 | 1 | 1 | 0.9597 |
| 432 | 1 | 1 | 0.993 | 0.994 | 1 | 1 | 0.948 | 0.96 | 0.987 | 1 | 1 | 0.998 | 0.922 | 1 | 0.978 | 0.97 | 0.809 | 1 | 1 | 0.9597 |
| 433 | 1 | 1 | 0.993 | 0.994 | 1 | 1 | 0.948 | 0.96 | 0.987 | 1 | 1 | 0.998 | 0.922 | 1 | 0.978 | 0.97 | 0.809 | 1 | 1 | 0.9597 |
| 434 | 1 | 1 | 0.993 | 0.994 | 1 | 1 | 0.945 | 0.958 | 0.987 | 1 | 1 | 0.998 | 0.922 | 1 | 0.978 | 0.97 | 0.809 | 1 | 1 | 0.9597 |
| 435 | 1 | 1 | 0.993 | 0.994 | 1 | 1 | 0.945 | 0.958 | 0.987 | 1 | 1 | 0.998 | 0.922 | 1 | 0.978 | 0.97 | 0.809 | 1 | 1 | 0.9597 |
| 436 | 1 | 1 | 0.993 | 0.994 | 1 | 1 | 0.945 | 0.958 | 0.987 | 1 | 1 | 0.998 | 0.922 | 1 | 0.978 | 0.97 | 0.809 | 1 | 1 | 0.9597 |
| 437 | 1 | 1 | 0.993 | 0.994 | 1 | 1 | 0.945 | 0.958 | 0.987 | 1 | 1 | 0.998 | 0.922 | 1 | 0.978 | 0.97 | 0.809 | 1 | 1 | 0.9597 |
| 438 | 1 | 1 | 0.993 | 0.994 | 1 | 1 | 0.945 | 0.958 | 0.987 | 1 | 1 | 0.998 | 0.929 | 1 | 0.977 | 0.97 | 0.809 | 1 | 1 | 0.9597 |
| 439 | 1 | 1 | 0.993 | 0.994 | 1 | 1 | 0.945 | 0.958 | 0.987 | 1 | 1 | 0.998 | 0.929 | 1 | 0.977 | 0.97 | 0.809 | 1 | 1 | 0.9597 |
| 440 | 1 | 1 | 0.993 | 0.994 | 1 | 1 | 0.945 | 0.958 | 0.987 | 1 | 1 | 0.998 | 0.929 | 1 | 0.977 | 0.97 | 0.809 | 1 | 1 | 0.9597 |
| 441 | 1 | 1 | 0.993 | 0.994 | 1 | 1 | 0.945 | 0.958 | 0.987 | 1 | 1 | 0.998 | 0.929 | 1 | 0.977 | 0.97 | 0.809 | 1 | 1 | 0.9597 |
| 442 | 1 | 1 | 0.993 | 0.994 | 1 | 1 | 0.945 | 0.958 | 0.987 | 1 | 1 | 0.998 | 0.929 | 1 | 0.977 | 0.97 | 0.809 | 1 | 1 | 0.9597 |
| 443 | 1 | 1 | 0.993 | 0.994 | 1 | 1 | 0.945 | 0.958 | 0.987 | 1 | 1 | 0.998 | 0.929 | 1 | 0.977 | 0.97 | 0.809 | 1 | 1 | 0.9597 |
| 444 | 1 | 1 | 0.993 | 0.994 | 1 | 1 | 0.945 | 0.958 | 0.987 | 1 | 1 | 0.998 | 0.929 | 1 | 0.977 | 0.97 | 0.809 | 1 | 1 | 0.9597 |
| 445 | 1 | 1 | 0.993 | 0.994 | 1 | 1 | 0.948 | 0.96 | 0.987 | 1 | 1 | 0.998 | 0.929 | 1 | 0.977 | 0.97 | 0.809 | 1 | 1 | 0.9597 |
| 446 | 1 | 1 | 0.993 | 0.994 | 1 | 1 | 0.945 | 0.958 | 0.987 | 1 | 1 | 0.998 | 0.929 | 1 | 0.977 | 0.97 | 0.809 | 1 | 1 | 0.9597 |
| 447 | 1 | 1 | 0.993 | 0.994 | 1 | 1 | 0.945 | 0.958 | 0.987 | 1 | 1 | 0.998 | 0.929 | 1 | 0.977 | 0.97 | 0.809 | 1 | 1 | 0.9597 |
| 448 | 1 | 1 | 0.993 | 0.994 | 1 | 1 | 0.948 | 0.96 | 0.987 | 1 | 1 | 0.998 | 0.922 | 1 | 0.977 | 0.969 | 0.809 | 1 | 1 | 0.9597 |
| 449 | 1 | 1 | 0.993 | 0.994 | 1 | 1 | 0.945 | 0.958 | 0.987 | 1 | 1 | 0.998 | 0.922 | 1 | 0.977 | 0.969 | 0.809 | 1 | 1 | 0.9597 |
| 450 | 1 | 1 | 0.993 | 0.994 | 1 | 1 | 0.945 | 0.958 | 0.987 | 1 | 1 | 0.998 | 0.922 | 1 | 0.977 | 0.969 | 0.809 | 1 | 1 | 0.9597 |
| 451 | 1 | 1 | 0.993 | 0.994 | 1 | 1 | 0.945 | 0.958 | 0.987 | 1 | 1 | 0.998 | 0.922 | 1 | 0.977 | 0.969 | 0.809 | 1 | 1 | 0.9597 |
| 452 | 1 | 1 | 0.993 | 0.994 | 1 | 1 | 0.945 | 0.958 | 0.987 | 1 | 1 | 0.998 | 0.922 | 1 | 0.977 | 0.969 | 0.809 | 1 | 1 | 0.9597 |
| 453 | 1 | 1 | 0.993 | 0.994 | 1 | 1 | 0.945 | 0.958 | 0.987 | 1 | 1 | 0.998 | 0.922 | 1 | 0.978 | 0.97 | 0.809 | 1 | 1 | 0.9597 |
| 454 | 1 | 1 | 0.993 | 0.994 | 1 | 1 | 0.945 | 0.958 | 0.987 | 1 | 1 | 0.998 | 0.922 | 1 | 0.978 | 0.97 | 0.809 | 1 | 1 | 0.9597 |
| 455 | 1 | 1 | 0.993 | 0.994 | 1 | 1 | 0.945 | 0.958 | 0.987 | 1 | 1 | 0.998 | 0.922 | 1 | 0.977 | 0.969 | 0.809 | 1 | 1 | 0.9597 |
| 456 | 1 | 1 | 0.993 | 0.994 | 1 | 1 | 0.945 | 0.958 | 0.987 | 1 | 1 | 0.998 | 0.922 | 1 | 0.977 | 0.969 | 0.809 | 1 | 1 | 0.9597 |
| 457 | 1 | 1 | 0.993 | 0.994 | 1 | 1 | 0.945 | 0.958 | 0.987 | 1 | 1 | 0.998 | 0.922 | 1 | 0.977 | 0.969 | 0.809 | 1 | 1 | 0.9597 |
| 458 | 1 | 1 | 0.993 | 0.994 | 1 | 1 | 0.945 | 0.958 | 0.987 | 1 | 1 | 0.998 | 0.922 | 1 | 0.976 | 0.968 | 0.809 | 1 | 1 | 0.9597 |
| 459 | 1 | 1 | 0.993 | 0.994 | 1 | 1 | 0.945 | 0.958 | 0.987 | 1 | 1 | 0.998 | 0.922 | 1 | 0.976 | 0.968 | 0.809 | 1 | 1 | 0.9597 |
| 460 | 1 | 1 | 0.993 | 0.994 | 1 | 1 | 0.945 | 0.958 | 0.987 | 1 | 1 | 0.998 | 0.922 | 1 | 0.976 | 0.968 | 0.814 | 1 | 1 | 0.9608 |
| 461 | 1 | 1 | 0.993 | 0.994 | 1 | 1 | 0.945 | 0.958 | 0.987 | 1 | 1 | 0.998 | 0.922 | 1 | 0.976 | 0.968 | 0.814 | 1 | 1 | 0.9608 |
| 462 | 1 | 1 | 0.993 | 0.994 | 1 | 1 | 0.945 | 0.958 | 0.987 | 1 | 1 | 0.998 | 0.922 | 1 | 0.976 | 0.968 | 0.814 | 1 | 1 | 0.9608 |
| 463 | 1 | 1 | 0.993 | 0.994 | 1 | 1 | 0.945 | 0.958 | 0.987 | 1 | 1 | 0.998 | 0.922 | 1 | 0.976 | 0.968 | 0.814 | 1 | 1 | 0.9608 |
| 464 | 1 | 1 | 0.993 | 0.994 | 1 | 1 | 0.945 | 0.958 | 0.987 | 1 | 1 | 0.998 | 0.922 | 1 | 0.976 | 0.968 | 0.814 | 1 | 1 | 0.9608 |
| 465 | 1 | 1 | 0.993 | 0.994 | 1 | 1 | 0.945 | 0.958 | 0.987 | 1 | 1 | 0.998 | 0.922 | 1 | 0.976 | 0.968 | 0.814 | 1 | 1 | 0.9608 |
| 466 | 1 | 1 | 0.993 | 0.994 | 1 | 1 | 0.945 | 0.958 | 0.987 | 1 | 1 | 0.998 | 0.922 | 1 | 0.976 | 0.968 | 0.814 | 1 | 1 | 0.9608 |
| 467 | 1 | 1 | 0.993 | 0.994 | 1 | 1 | 0.945 | 0.958 | 0.987 | 1 | 1 | 0.998 | 0.922 | 1 | 0.976 | 0.968 | 0.803 | 1 | 1 | 0.9586 |
| 468 | 1 | 1 | 0.993 | 0.994 | 1 | 1 | 0.945 | 0.958 | 0.987 | 1 | 1 | 0.998 | 0.922 | 1 | 0.976 | 0.968 | 0.809 | 1 | 1 | 0.9597 |
| 469 | 1 | 1 | 0.993 | 0.994 | 1 | 1 | 0.945 | 0.958 | 0.987 | 1 | 1 | 0.998 | 0.922 | 1 | 0.976 | 0.968 | 0.809 | 1 | 1 | 0.9597 |
| 470 | 1 | 1 | 0.993 | 0.994 | 1 | 1 | 0.945 | 0.958 | 0.987 | 1 | 1 | 0.998 | 0.922 | 1 | 0.976 | 0.968 | 0.809 | 1 | 1 | 0.9597 |
| 471 | 1 | 1 | 0.993 | 0.994 | 1 | 1 | 0.945 | 0.958 | 0.987 | 1 | 1 | 0.998 | 0.922 | 1 | 0.976 | 0.968 | 0.809 | 1 | 1 | 0.9597 |
| 472 | 1 | 1 | 0.993 | 0.994 | 1 | 1 | 0.945 | 0.958 | 0.987 | 1 | 1 | 0.998 | 0.922 | 1 | 0.976 | 0.968 | 0.814 | 1 | 1 | 0.9608 |
| 473 | 1 | 1 | 0.993 | 0.994 | 1 | 1 | 0.945 | 0.958 | 0.987 | 1 | 1 | 0.998 | 0.922 | 1 | 0.976 | 0.968 | 0.809 | 1 | 1 | 0.9597 |
| 474 | 1 | 1 | 0.993 | 0.994 | 1 | 1 | 0.943 | 0.957 | 0.987 | 1 | 1 | 0.998 | 0.922 | 1 | 0.976 | 0.968 | 0.814 | 1 | 1 | 0.9608 |
| 475 | 1 | 1 | 0.993 | 0.994 | 1 | 1 | 0.943 | 0.957 | 0.987 | 1 | 1 | 0.998 | 0.922 | 1 | 0.976 | 0.968 | 0.809 | 1 | 1 | 0.9597 |
| 476 | 1 | 1 | 0.99 | 0.992 | 1 | 1 | 0.943 | 0.957 | 0.987 | 1 | 1 | 0.998 | 0.922 | 1 | 0.974 | 0.967 | 0.809 | 1 | 1 | 0.9597 |
| 477 | 1 | 1 | 0.99 | 0.992 | 1 | 1 | 0.943 | 0.957 | 0.987 | 1 | 1 | 0.998 | 0.929 | 1 | 0.974 | 0.968 | 0.809 | 1 | 1 | 0.9597 |
| 478 | 1 | 1 | 0.99 | 0.992 | 1 | 1 | 0.943 | 0.957 | 0.987 | 1 | 1 | 0.998 | 0.929 | 1 | 0.974 | 0.968 | 0.809 | 1 | 1 | 0.9597 |
| 479 | 1 | 1 | 0.99 | 0.992 | 1 | 1 | 0.943 | 0.957 | 0.987 | 1 | 1 | 0.998 | 0.922 | 1 | 0.974 | 0.967 | 0.809 | 1 | 1 | 0.9597 |
| 480 | 1 | 1 | 0.99 | 0.992 | 1 | 1 | 0.943 | 0.957 | 0.987 | 1 | 1 | 0.998 | 0.922 | 1 | 0.974 | 0.967 | 0.809 | 1 | 1 | 0.9597 |
| 481 | 1 | 1 | 0.99 | 0.992 | 1 | 1 | 0.943 | 0.957 | 0.987 | 1 | 1 | 0.998 | 0.922 | 1 | 0.974 | 0.967 | 0.803 | 1 | 1 | 0.9586 |
| 482 | 1 | 1 | 0.99 | 0.992 | 1 | 1 | 0.943 | 0.957 | 0.987 | 1 | 1 | 0.998 | 0.922 | 1 | 0.973 | 0.966 | 0.803 | 1 | 1 | 0.9586 |
| 483 | 1 | 1 | 0.99 | 0.992 | 1 | 1 | 0.945 | 0.958 | 0.975 | 1 | 1 | 0.996 | 0.929 | 1 | 0.973 | 0.967 | 0.809 | 1 | 1 | 0.9597 |
| 484 | 1 | 1 | 0.99 | 0.992 | 1 | 1 | 0.945 | 0.958 | 0.975 | 1 | 1 | 0.996 | 0.922 | 1 | 0.973 | 0.966 | 0.809 | 1 | 1 | 0.9597 |
| 485 | 1 | 1 | 0.99 | 0.992 | 1 | 1 | 0.945 | 0.958 | 0.987 | 1 | 1 | 0.998 | 0.929 | 1 | 0.974 | 0.968 | 0.803 | 1 | 1 | 0.9586 |
| 486 | 1 | 1 | 0.99 | 0.992 | 1 | 1 | 0.945 | 0.958 | 0.987 | 1 | 1 | 0.998 | 0.929 | 1 | 0.973 | 0.967 | 0.803 | 1 | 1 | 0.9586 |
| 487 | 1 | 1 | 0.99 | 0.992 | 1 | 1 | 0.945 | 0.958 | 0.987 | 1 | 1 | 0.998 | 0.929 | 1 | 0.973 | 0.967 | 0.803 | 1 | 1 | 0.9586 |
| 488 | 1 | 1 | 0.99 | 0.992 | 1 | 1 | 0.945 | 0.958 | 0.987 | 1 | 1 | 0.998 | 0.929 | 1 | 0.973 | 0.967 | 0.803 | 1 | 1 | 0.9586 |
| 489 | 1 | 1 | 0.99 | 0.992 | 1 | 1 | 0.945 | 0.958 | 0.987 | 1 | 1 | 0.998 | 0.929 | 1 | 0.973 | 0.967 | 0.814 | 1 | 1 | 0.9608 |
| 490 | 1 | 1 | 0.99 | 0.992 | 1 | 1 | 0.945 | 0.958 | 0.987 | 1 | 1 | 0.998 | 0.929 | 1 | 0.973 | 0.967 | 0.798 | 1 | 1 | 0.9574 |
| 491 | 1 | 1 | 0.99 | 0.992 | 1 | 1 | 0.943 | 0.957 | 0.987 | 1 | 1 | 0.998 | 0.929 | 1 | 0.974 | 0.968 | 0.798 | 1 | 1 | 0.9574 |
| 492 | 1 | 1 | 0.99 | 0.992 | 1 | 1 | 0.943 | 0.957 | 0.987 | 1 | 1 | 0.998 | 0.929 | 1 | 0.974 | 0.968 | 0.798 | 1 | 1 | 0.9574 |
| 493 | 1 | 1 | 0.99 | 0.992 | 1 | 1 | 0.943 | 0.957 | 0.987 | 1 | 1 | 0.998 | 0.929 | 1 | 0.974 | 0.968 | 0.798 | 1 | 1 | 0.9574 |
| 494 | 1 | 1 | 0.99 | 0.992 | 1 | 1 | 0.943 | 0.957 | 0.987 | 1 | 1 | 0.998 | 0.929 | 1 | 0.974 | 0.968 | 0.798 | 1 | 1 | 0.9574 |
| 495 | 1 | 1 | 0.99 | 0.992 | 1 | 1 | 0.943 | 0.957 | 0.987 | 1 | 1 | 0.998 | 0.929 | 1 | 0.974 | 0.968 | 0.798 | 1 | 1 | 0.9574 |
| 496 | 1 | 1 | 0.99 | 0.992 | 1 | 1 | 0.943 | 0.957 | 0.987 | 1 | 1 | 0.998 | 0.929 | 1 | 0.974 | 0.968 | 0.798 | 1 | 1 | 0.9574 |
| 497 | 1 | 1 | 0.99 | 0.992 | 1 | 1 | 0.943 | 0.957 | 0.987 | 1 | 1 | 0.998 | 0.929 | 1 | 0.974 | 0.968 | 0.798 | 1 | 1 | 0.9574 |
| 498 | 1 | 1 | 0.99 | 0.992 | 1 | 1 | 0.943 | 0.957 | 0.987 | 1 | 1 | 0.998 | 0.929 | 1 | 0.974 | 0.968 | 0.798 | 1 | 1 | 0.9574 |
| 499 | 1 | 1 | 0.99 | 0.992 | 1 | 1 | 0.943 | 0.957 | 0.987 | 1 | 1 | 0.998 | 0.929 | 1 | 0.974 | 0.968 | 0.798 | 1 | 1 | 0.9574 |
| 500 | 1 | 1 | 0.99 | 0.992 | 1 | 1 | 0.943 | 0.957 | 0.987 | 1 | 1 | 0.998 | 0.929 | 1 | 0.974 | 0.968 | 0.798 | 1 | 1 | 0.9574 |

1. The dataset of H0351.2001 was selected as the training dataset

| Number of features | H0351.1009 | | | | H0351.1012 | | | | H0351.1015 | | | | H0351.1016 | | | | H0351.2002 | | | |
| --- | --- | --- | --- | --- | --- | --- | --- | --- | --- | --- | --- | --- | --- | --- | --- | --- | --- | --- | --- | --- |
|  | class1 | class2 | class3 | total | class1 | class2 | class3 | total | class1 | class2 | class3 | total | class1 | class2 | class3 | total | class1 | class2 | class3 | total |
| 4 | 1 | 0.929 | 0.976 | 0.972 | 0.988 | 0.958 | 0.993 | 0.989 | 0.924 | 0.984 | 0.991 | 0.979 | 0.983 | 1 | 0.997 | 0.996 | 0.926 | 0.976 | 0.998 | 0.981 |
| 5 | 1 | 0.976 | 0.976 | 0.978 | 0.988 | 0.938 | 0.993 | 0.987 | 0.924 | 0.984 | 0.991 | 0.979 | 1 | 0.988 | 0.997 | 0.996 | 0.926 | 0.988 | 0.998 | 0.9821 |
| 6 | 1 | 0.976 | 0.98 | 0.981 | 1 | 1 | 0.995 | 0.996 | 0.937 | 1 | 0.994 | 0.985 | 1 | 1 | 1 | 1 | 0.926 | 1 | 0.998 | 0.9832 |
| 7 | 1 | 0.952 | 0.973 | 0.972 | 0.988 | 1 | 0.995 | 0.994 | 0.949 | 1 | 0.985 | 0.981 | 0.983 | 1 | 0.997 | 0.996 | 0.926 | 1 | 0.997 | 0.9821 |
| 8 | 1 | 0.976 | 0.98 | 0.981 | 0.988 | 1 | 0.995 | 0.994 | 0.949 | 1 | 0.985 | 0.981 | 0.983 | 1 | 0.997 | 0.996 | 0.92 | 1 | 0.995 | 0.9798 |
| 9 | 1 | 1 | 0.99 | 0.992 | 0.988 | 1 | 0.995 | 0.994 | 0.962 | 1 | 0.991 | 0.987 | 1 | 1 | 0.997 | 0.998 | 0.894 | 1 | 0.998 | 0.9765 |
| 10 | 1 | 0.952 | 0.99 | 0.986 | 0.988 | 1 | 0.998 | 0.996 | 0.949 | 1 | 0.994 | 0.987 | 0.983 | 1 | 0.997 | 0.996 | 0.904 | 1 | 1 | 0.9798 |
| 11 | 1 | 0.929 | 0.99 | 0.983 | 0.988 | 0.979 | 0.995 | 0.992 | 0.949 | 1 | 0.994 | 0.987 | 0.983 | 1 | 0.997 | 0.996 | 0.904 | 1 | 0.998 | 0.9787 |
| 12 | 1 | 0.929 | 0.997 | 0.989 | 0.975 | 0.979 | 0.998 | 0.992 | 0.924 | 0.984 | 0.994 | 0.981 | 0.983 | 0.988 | 1 | 0.996 | 0.904 | 0.988 | 0.998 | 0.9776 |
| 13 | 1 | 0.952 | 0.997 | 0.992 | 0.975 | 1 | 0.998 | 0.994 | 0.924 | 1 | 0.997 | 0.985 | 0.983 | 1 | 1 | 0.998 | 0.904 | 1 | 0.998 | 0.9787 |
| 14 | 1 | 0.976 | 0.993 | 0.992 | 0.988 | 1 | 0.998 | 0.996 | 0.924 | 1 | 0.991 | 0.981 | 0.983 | 1 | 1 | 0.998 | 0.904 | 1 | 0.998 | 0.9787 |
| 15 | 1 | 0.952 | 0.983 | 0.981 | 1 | 1 | 0.995 | 0.996 | 0.949 | 1 | 0.985 | 0.981 | 1 | 1 | 0.981 | 0.986 | 0.904 | 1 | 0.998 | 0.9787 |
| 16 | 1 | 0.952 | 0.976 | 0.975 | 1 | 1 | 0.995 | 0.996 | 0.949 | 1 | 0.985 | 0.981 | 1 | 1 | 0.983 | 0.988 | 0.899 | 1 | 0.997 | 0.9765 |
| 17 | 1 | 0.952 | 0.983 | 0.981 | 0.988 | 1 | 0.995 | 0.994 | 0.937 | 1 | 0.985 | 0.979 | 1 | 1 | 0.981 | 0.986 | 0.915 | 1 | 0.994 | 0.9776 |
| 18 | 1 | 0.952 | 0.986 | 0.983 | 1 | 1 | 0.998 | 0.998 | 0.949 | 1 | 0.988 | 0.983 | 1 | 1 | 0.981 | 0.986 | 0.92 | 1 | 0.994 | 0.9787 |
| 19 | 1 | 0.976 | 0.986 | 0.986 | 1 | 0.979 | 0.998 | 0.996 | 0.949 | 1 | 0.988 | 0.983 | 1 | 1 | 0.981 | 0.986 | 0.92 | 1 | 0.995 | 0.9798 |
| 20 | 1 | 0.976 | 0.99 | 0.989 | 1 | 0.979 | 0.998 | 0.996 | 0.949 | 1 | 0.985 | 0.981 | 1 | 1 | 0.981 | 0.986 | 0.91 | 1 | 1 | 0.981 |
| 21 | 1 | 0.952 | 0.986 | 0.983 | 0.988 | 1 | 0.993 | 0.992 | 0.937 | 1 | 0.982 | 0.977 | 1 | 1 | 0.972 | 0.98 | 0.926 | 1 | 0.987 | 0.9754 |
| 22 | 1 | 0.952 | 0.993 | 0.989 | 0.988 | 1 | 0.995 | 0.994 | 0.949 | 1 | 0.982 | 0.979 | 1 | 1 | 0.975 | 0.982 | 0.92 | 1 | 0.987 | 0.9742 |
| 23 | 1 | 0.952 | 0.993 | 0.989 | 0.988 | 1 | 0.995 | 0.994 | 0.937 | 1 | 0.982 | 0.977 | 0.983 | 1 | 0.975 | 0.98 | 0.92 | 1 | 0.987 | 0.9742 |
| 24 | 1 | 0.976 | 0.993 | 0.992 | 0.988 | 1 | 0.998 | 0.996 | 0.924 | 1 | 0.982 | 0.974 | 0.966 | 1 | 0.978 | 0.98 | 0.926 | 1 | 0.989 | 0.9765 |
| 25 | 1 | 0.976 | 0.993 | 0.992 | 0.988 | 1 | 0.998 | 0.996 | 0.937 | 1 | 0.985 | 0.979 | 1 | 1 | 0.978 | 0.984 | 0.926 | 1 | 0.994 | 0.9798 |
| 26 | 1 | 0.976 | 0.993 | 0.992 | 0.988 | 1 | 0.998 | 0.996 | 0.937 | 1 | 0.985 | 0.979 | 1 | 1 | 0.978 | 0.984 | 0.926 | 1 | 0.994 | 0.9798 |
| 27 | 1 | 0.976 | 0.993 | 0.992 | 0.988 | 1 | 0.998 | 0.996 | 0.937 | 1 | 0.982 | 0.977 | 1 | 1 | 0.978 | 0.984 | 0.926 | 1 | 0.994 | 0.9798 |
| 28 | 1 | 1 | 0.993 | 0.994 | 0.988 | 1 | 0.998 | 0.996 | 0.937 | 1 | 0.985 | 0.979 | 0.983 | 0.988 | 0.975 | 0.978 | 0.926 | 1 | 0.994 | 0.9798 |
| 29 | 1 | 1 | 0.993 | 0.994 | 0.988 | 1 | 0.998 | 0.996 | 0.937 | 1 | 0.985 | 0.979 | 0.983 | 0.988 | 0.975 | 0.978 | 0.926 | 1 | 0.994 | 0.9798 |
| 30 | 1 | 1 | 0.993 | 0.994 | 0.988 | 1 | 0.998 | 0.996 | 0.937 | 1 | 0.985 | 0.979 | 0.983 | 1 | 0.978 | 0.982 | 0.926 | 1 | 0.994 | 0.9798 |
| 31 | 1 | 1 | 0.993 | 0.994 | 0.988 | 1 | 0.998 | 0.996 | 0.937 | 1 | 0.985 | 0.979 | 0.983 | 1 | 0.978 | 0.982 | 0.926 | 1 | 0.994 | 0.9798 |
| 32 | 1 | 1 | 0.993 | 0.994 | 0.988 | 1 | 0.998 | 0.996 | 0.962 | 1 | 0.979 | 0.979 | 1 | 1 | 0.975 | 0.982 | 0.931 | 1 | 0.989 | 0.9776 |
| 33 | 1 | 1 | 0.993 | 0.994 | 0.988 | 1 | 0.995 | 0.994 | 0.962 | 1 | 0.979 | 0.979 | 0.983 | 1 | 0.981 | 0.984 | 0.941 | 1 | 0.994 | 0.9832 |
| 34 | 1 | 1 | 0.993 | 0.994 | 0.988 | 1 | 0.995 | 0.994 | 0.962 | 1 | 0.979 | 0.979 | 0.983 | 1 | 0.981 | 0.984 | 0.941 | 1 | 0.994 | 0.9832 |
| 35 | 1 | 1 | 0.993 | 0.994 | 0.988 | 1 | 0.995 | 0.994 | 0.975 | 1 | 0.976 | 0.979 | 0.983 | 1 | 0.981 | 0.984 | 0.952 | 1 | 0.992 | 0.9843 |
| 36 | 1 | 1 | 0.993 | 0.994 | 0.988 | 1 | 0.995 | 0.994 | 0.962 | 1 | 0.976 | 0.977 | 0.966 | 1 | 0.983 | 0.984 | 0.952 | 1 | 0.994 | 0.9854 |
| 37 | 1 | 1 | 0.993 | 0.994 | 0.988 | 1 | 0.995 | 0.994 | 0.975 | 1 | 0.979 | 0.981 | 0.983 | 1 | 0.981 | 0.984 | 0.952 | 1 | 0.992 | 0.9843 |
| 38 | 1 | 1 | 0.993 | 0.994 | 0.988 | 1 | 0.995 | 0.994 | 0.975 | 1 | 0.976 | 0.979 | 0.983 | 1 | 0.983 | 0.986 | 0.952 | 1 | 0.99 | 0.9832 |
| 39 | 1 | 1 | 0.993 | 0.994 | 0.988 | 1 | 0.998 | 0.996 | 0.962 | 1 | 0.982 | 0.981 | 0.966 | 1 | 0.983 | 0.984 | 0.952 | 1 | 0.992 | 0.9843 |
| 40 | 1 | 1 | 0.993 | 0.994 | 1 | 1 | 0.993 | 0.994 | 0.975 | 1 | 0.982 | 0.983 | 0.966 | 1 | 0.983 | 0.984 | 0.947 | 1 | 0.995 | 0.9854 |
| 41 | 1 | 1 | 0.993 | 0.994 | 1 | 1 | 0.993 | 0.994 | 0.975 | 1 | 0.982 | 0.983 | 0.966 | 1 | 0.983 | 0.984 | 0.947 | 1 | 0.995 | 0.9854 |
| 42 | 1 | 1 | 0.993 | 0.994 | 1 | 1 | 0.993 | 0.994 | 0.975 | 1 | 0.982 | 0.983 | 0.966 | 1 | 0.986 | 0.986 | 0.947 | 1 | 0.995 | 0.9854 |
| 43 | 1 | 1 | 0.997 | 0.997 | 1 | 1 | 0.99 | 0.992 | 0.975 | 1 | 0.982 | 0.983 | 0.949 | 1 | 0.983 | 0.982 | 0.968 | 1 | 0.989 | 0.9854 |
| 44 | 1 | 1 | 0.997 | 0.997 | 1 | 1 | 0.99 | 0.992 | 0.975 | 1 | 0.985 | 0.985 | 0.932 | 1 | 0.981 | 0.978 | 0.968 | 1 | 0.992 | 0.9877 |
| 45 | 1 | 1 | 0.997 | 0.997 | 1 | 1 | 0.99 | 0.992 | 0.987 | 1 | 0.985 | 0.987 | 0.949 | 1 | 0.983 | 0.982 | 0.973 | 1 | 0.986 | 0.9843 |
| 46 | 1 | 1 | 0.997 | 0.997 | 1 | 1 | 0.99 | 0.992 | 0.987 | 1 | 0.988 | 0.989 | 0.949 | 1 | 0.981 | 0.98 | 0.968 | 1 | 0.989 | 0.9854 |
| 47 | 1 | 1 | 0.997 | 0.997 | 1 | 1 | 0.99 | 0.992 | 0.975 | 1 | 0.988 | 0.987 | 0.949 | 1 | 0.983 | 0.982 | 0.968 | 1 | 0.989 | 0.9854 |
| 48 | 1 | 1 | 0.993 | 0.994 | 1 | 1 | 0.983 | 0.987 | 0.987 | 1 | 0.988 | 0.989 | 0.966 | 1 | 0.981 | 0.982 | 0.963 | 1 | 0.997 | 0.9899 |
| 49 | 1 | 1 | 0.993 | 0.994 | 1 | 1 | 0.983 | 0.987 | 0.987 | 1 | 0.988 | 0.989 | 0.966 | 1 | 0.981 | 0.982 | 0.963 | 1 | 0.997 | 0.9899 |
| 50 | 1 | 1 | 0.993 | 0.994 | 1 | 1 | 0.983 | 0.987 | 0.975 | 1 | 0.988 | 0.987 | 0.966 | 1 | 0.981 | 0.982 | 0.963 | 1 | 0.995 | 0.9888 |
| 51 | 1 | 1 | 0.993 | 0.994 | 1 | 1 | 0.983 | 0.987 | 0.987 | 1 | 0.985 | 0.987 | 0.966 | 1 | 0.981 | 0.982 | 0.963 | 1 | 0.994 | 0.9877 |
| 52 | 1 | 1 | 0.993 | 0.994 | 1 | 1 | 0.983 | 0.987 | 0.987 | 1 | 0.985 | 0.987 | 0.966 | 1 | 0.981 | 0.982 | 0.963 | 1 | 0.994 | 0.9877 |
| 53 | 1 | 1 | 0.99 | 0.992 | 1 | 1 | 0.98 | 0.985 | 0.987 | 1 | 0.985 | 0.987 | 0.966 | 1 | 0.981 | 0.982 | 0.963 | 1 | 0.994 | 0.9877 |
| 54 | 1 | 1 | 0.993 | 0.994 | 1 | 1 | 0.98 | 0.985 | 0.987 | 1 | 0.985 | 0.987 | 0.966 | 1 | 0.981 | 0.982 | 0.963 | 1 | 0.995 | 0.9888 |
| 55 | 1 | 1 | 0.99 | 0.992 | 1 | 1 | 0.98 | 0.985 | 0.987 | 1 | 0.985 | 0.987 | 0.966 | 1 | 0.981 | 0.982 | 0.963 | 1 | 0.995 | 0.9888 |
| 56 | 1 | 1 | 0.99 | 0.992 | 1 | 1 | 0.983 | 0.987 | 0.987 | 1 | 0.988 | 0.989 | 0.966 | 1 | 0.981 | 0.982 | 0.963 | 1 | 0.995 | 0.9888 |
| 57 | 1 | 1 | 0.993 | 0.994 | 1 | 1 | 0.985 | 0.989 | 0.987 | 1 | 0.985 | 0.987 | 0.966 | 1 | 0.983 | 0.984 | 0.963 | 1 | 0.995 | 0.9888 |
| 58 | 1 | 1 | 0.99 | 0.992 | 1 | 1 | 0.993 | 0.994 | 0.987 | 1 | 0.988 | 0.989 | 0.966 | 1 | 0.983 | 0.984 | 0.963 | 1 | 0.995 | 0.9888 |
| 59 | 1 | 1 | 0.993 | 0.994 | 1 | 1 | 0.993 | 0.994 | 0.975 | 1 | 0.988 | 0.987 | 0.966 | 1 | 0.983 | 0.984 | 0.963 | 1 | 0.997 | 0.9899 |
| 60 | 1 | 1 | 0.993 | 0.994 | 1 | 1 | 0.988 | 0.991 | 0.987 | 1 | 0.988 | 0.989 | 0.966 | 1 | 0.983 | 0.984 | 0.963 | 1 | 0.997 | 0.9899 |
| 61 | 1 | 1 | 0.993 | 0.994 | 1 | 1 | 0.983 | 0.987 | 0.987 | 1 | 0.985 | 0.987 | 0.966 | 1 | 0.981 | 0.982 | 0.957 | 1 | 0.994 | 0.9866 |
| 62 | 1 | 1 | 0.993 | 0.994 | 1 | 1 | 0.98 | 0.985 | 0.987 | 1 | 0.985 | 0.987 | 0.966 | 1 | 0.983 | 0.984 | 0.957 | 1 | 0.992 | 0.9854 |
| 63 | 1 | 1 | 0.99 | 0.992 | 1 | 1 | 0.98 | 0.985 | 0.987 | 1 | 0.982 | 0.985 | 0.983 | 1 | 0.981 | 0.984 | 0.957 | 1 | 0.992 | 0.9854 |
| 64 | 1 | 1 | 0.986 | 0.989 | 1 | 1 | 0.98 | 0.985 | 0.987 | 1 | 0.982 | 0.985 | 0.966 | 1 | 0.983 | 0.984 | 0.957 | 1 | 0.992 | 0.9854 |
| 65 | 1 | 1 | 0.986 | 0.989 | 1 | 1 | 0.98 | 0.985 | 0.987 | 1 | 0.985 | 0.987 | 0.983 | 1 | 0.983 | 0.986 | 0.957 | 1 | 0.992 | 0.9854 |
| 66 | 1 | 1 | 0.986 | 0.989 | 1 | 1 | 0.98 | 0.985 | 0.987 | 1 | 0.985 | 0.987 | 0.966 | 1 | 0.981 | 0.982 | 0.957 | 1 | 0.992 | 0.9854 |
| 67 | 1 | 1 | 0.993 | 0.994 | 1 | 1 | 0.993 | 0.994 | 0.975 | 1 | 0.988 | 0.987 | 1 | 1 | 0.986 | 0.99 | 0.957 | 1 | 0.995 | 0.9877 |
| 68 | 1 | 1 | 0.997 | 0.997 | 1 | 1 | 0.993 | 0.994 | 0.975 | 1 | 0.988 | 0.987 | 1 | 1 | 0.986 | 0.99 | 0.957 | 1 | 0.995 | 0.9877 |
| 69 | 1 | 1 | 0.997 | 0.997 | 1 | 1 | 0.99 | 0.992 | 0.975 | 1 | 0.994 | 0.991 | 1 | 1 | 0.986 | 0.99 | 0.973 | 1 | 0.99 | 0.9877 |
| 70 | 1 | 1 | 0.997 | 0.997 | 1 | 1 | 0.99 | 0.992 | 0.975 | 1 | 0.994 | 0.991 | 1 | 1 | 0.986 | 0.99 | 0.973 | 1 | 0.99 | 0.9877 |
| 71 | 1 | 1 | 0.997 | 0.997 | 1 | 1 | 0.988 | 0.991 | 0.975 | 1 | 0.991 | 0.989 | 1 | 1 | 0.989 | 0.992 | 0.973 | 1 | 0.99 | 0.9877 |
| 72 | 1 | 1 | 0.997 | 0.997 | 1 | 1 | 0.988 | 0.991 | 0.975 | 1 | 0.991 | 0.989 | 1 | 1 | 0.989 | 0.992 | 0.973 | 1 | 0.992 | 0.9888 |
| 73 | 1 | 1 | 1 | 1 | 1 | 1 | 0.993 | 0.994 | 0.987 | 1 | 0.982 | 0.985 | 1 | 1 | 0.986 | 0.99 | 0.973 | 1 | 0.989 | 0.9866 |
| 74 | 1 | 1 | 1 | 1 | 1 | 1 | 0.993 | 0.994 | 0.987 | 1 | 0.979 | 0.983 | 1 | 1 | 0.986 | 0.99 | 0.973 | 1 | 0.989 | 0.9866 |
| 75 | 1 | 1 | 1 | 1 | 1 | 1 | 0.993 | 0.994 | 0.987 | 1 | 0.979 | 0.983 | 1 | 1 | 0.986 | 0.99 | 0.973 | 1 | 0.992 | 0.9888 |
| 76 | 1 | 1 | 1 | 1 | 1 | 1 | 0.993 | 0.994 | 0.987 | 1 | 0.988 | 0.989 | 1 | 1 | 0.989 | 0.992 | 0.979 | 1 | 0.992 | 0.9899 |
| 77 | 1 | 1 | 1 | 1 | 1 | 1 | 0.993 | 0.994 | 0.987 | 1 | 0.988 | 0.989 | 1 | 1 | 0.989 | 0.992 | 0.979 | 1 | 0.992 | 0.9899 |
| 78 | 1 | 1 | 1 | 1 | 1 | 1 | 0.993 | 0.994 | 0.987 | 1 | 0.988 | 0.989 | 1 | 1 | 0.989 | 0.992 | 0.973 | 1 | 0.992 | 0.9888 |
| 79 | 1 | 1 | 1 | 1 | 1 | 1 | 0.993 | 0.994 | 0.987 | 1 | 0.991 | 0.991 | 1 | 1 | 0.989 | 0.992 | 0.963 | 1 | 0.992 | 0.9866 |
| 80 | 1 | 1 | 1 | 1 | 1 | 1 | 0.993 | 0.994 | 0.987 | 1 | 0.985 | 0.987 | 1 | 1 | 0.989 | 0.992 | 0.968 | 1 | 0.992 | 0.9877 |
| 81 | 1 | 1 | 1 | 1 | 1 | 1 | 0.993 | 0.994 | 0.987 | 1 | 0.985 | 0.987 | 1 | 1 | 0.989 | 0.992 | 0.968 | 1 | 0.992 | 0.9877 |
| 82 | 1 | 1 | 1 | 1 | 1 | 1 | 0.993 | 0.994 | 0.987 | 1 | 0.985 | 0.987 | 1 | 1 | 0.989 | 0.992 | 0.979 | 1 | 0.992 | 0.9899 |
| 83 | 1 | 1 | 1 | 1 | 1 | 1 | 0.993 | 0.994 | 0.987 | 1 | 0.985 | 0.987 | 1 | 1 | 0.989 | 0.992 | 0.973 | 1 | 0.994 | 0.9899 |
| 84 | 1 | 1 | 1 | 1 | 1 | 1 | 0.993 | 0.994 | 0.987 | 1 | 0.985 | 0.987 | 1 | 1 | 0.989 | 0.992 | 0.973 | 1 | 0.992 | 0.9888 |
| 85 | 1 | 1 | 1 | 1 | 1 | 1 | 0.993 | 0.994 | 0.987 | 1 | 0.985 | 0.987 | 1 | 1 | 0.989 | 0.992 | 0.968 | 1 | 0.99 | 0.9866 |
| 86 | 1 | 1 | 1 | 1 | 1 | 1 | 0.993 | 0.994 | 0.987 | 1 | 0.985 | 0.987 | 1 | 1 | 0.989 | 0.992 | 0.973 | 1 | 0.99 | 0.9877 |
| 87 | 1 | 1 | 1 | 1 | 1 | 1 | 0.993 | 0.994 | 0.975 | 1 | 0.988 | 0.987 | 1 | 1 | 0.989 | 0.992 | 0.968 | 1 | 0.992 | 0.9877 |
| 88 | 1 | 1 | 1 | 1 | 1 | 1 | 0.993 | 0.994 | 0.975 | 1 | 0.985 | 0.985 | 1 | 1 | 0.986 | 0.99 | 0.963 | 1 | 0.994 | 0.9877 |
| 89 | 1 | 1 | 1 | 1 | 1 | 1 | 0.993 | 0.994 | 0.949 | 1 | 0.991 | 0.985 | 0.949 | 1 | 0.989 | 0.986 | 0.979 | 1 | 0.987 | 0.9866 |
| 90 | 1 | 1 | 1 | 1 | 1 | 1 | 0.995 | 0.996 | 0.949 | 1 | 0.991 | 0.985 | 0.932 | 1 | 0.989 | 0.984 | 0.973 | 1 | 0.989 | 0.9866 |
| 91 | 1 | 1 | 1 | 1 | 1 | 1 | 0.995 | 0.996 | 0.949 | 1 | 0.994 | 0.987 | 0.932 | 1 | 0.989 | 0.984 | 0.973 | 1 | 0.989 | 0.9866 |
| 92 | 1 | 1 | 1 | 1 | 1 | 1 | 0.995 | 0.996 | 0.949 | 1 | 0.994 | 0.987 | 0.932 | 1 | 0.989 | 0.984 | 0.973 | 1 | 0.989 | 0.9866 |
| 93 | 1 | 1 | 1 | 1 | 1 | 1 | 0.995 | 0.996 | 0.949 | 1 | 0.991 | 0.985 | 0.949 | 1 | 0.986 | 0.984 | 0.957 | 1 | 0.992 | 0.9854 |
| 94 | 1 | 1 | 1 | 1 | 1 | 1 | 0.995 | 0.996 | 0.962 | 1 | 0.991 | 0.987 | 1 | 1 | 0.986 | 0.99 | 0.957 | 1 | 0.994 | 0.9866 |
| 95 | 1 | 1 | 1 | 1 | 1 | 1 | 0.995 | 0.996 | 0.962 | 1 | 0.991 | 0.987 | 1 | 1 | 0.986 | 0.99 | 0.963 | 1 | 0.995 | 0.9888 |
| 96 | 1 | 1 | 1 | 1 | 1 | 1 | 0.995 | 0.996 | 0.962 | 1 | 0.991 | 0.987 | 1 | 1 | 0.986 | 0.99 | 0.968 | 1 | 0.994 | 0.9888 |
| 97 | 1 | 1 | 1 | 1 | 1 | 1 | 0.995 | 0.996 | 0.962 | 1 | 0.991 | 0.987 | 1 | 1 | 0.986 | 0.99 | 0.968 | 1 | 0.994 | 0.9888 |
| 98 | 1 | 1 | 1 | 1 | 1 | 1 | 0.995 | 0.996 | 0.962 | 1 | 0.991 | 0.987 | 1 | 1 | 0.986 | 0.99 | 0.968 | 1 | 0.995 | 0.9899 |
| 99 | 1 | 1 | 1 | 1 | 1 | 1 | 0.995 | 0.996 | 0.962 | 1 | 0.991 | 0.987 | 1 | 1 | 0.986 | 0.99 | 0.973 | 1 | 0.994 | 0.9899 |
| 100 | 1 | 1 | 1 | 1 | 1 | 1 | 0.995 | 0.996 | 0.962 | 1 | 0.991 | 0.987 | 1 | 1 | 0.986 | 0.99 | 0.973 | 1 | 0.992 | 0.9888 |
| 101 | 1 | 1 | 1 | 1 | 1 | 1 | 0.995 | 0.996 | 0.949 | 1 | 0.994 | 0.987 | 0.983 | 1 | 0.986 | 0.988 | 0.957 | 1 | 0.995 | 0.9877 |
| 102 | 1 | 1 | 1 | 1 | 1 | 1 | 0.995 | 0.996 | 0.949 | 1 | 0.994 | 0.987 | 1 | 1 | 0.986 | 0.99 | 0.963 | 1 | 0.995 | 0.9888 |
| 103 | 1 | 1 | 1 | 1 | 1 | 1 | 0.995 | 0.996 | 0.949 | 1 | 0.994 | 0.987 | 0.983 | 1 | 0.986 | 0.988 | 0.968 | 1 | 0.994 | 0.9888 |
| 104 | 1 | 1 | 1 | 1 | 1 | 1 | 0.995 | 0.996 | 0.949 | 1 | 0.994 | 0.987 | 0.983 | 1 | 0.986 | 0.988 | 0.968 | 1 | 0.992 | 0.9877 |
| 105 | 1 | 1 | 1 | 1 | 1 | 1 | 0.995 | 0.996 | 0.949 | 1 | 0.994 | 0.987 | 0.983 | 1 | 0.986 | 0.988 | 0.968 | 1 | 0.992 | 0.9877 |
| 106 | 1 | 1 | 1 | 1 | 1 | 1 | 0.995 | 0.996 | 0.949 | 1 | 0.994 | 0.987 | 0.983 | 1 | 0.986 | 0.988 | 0.973 | 1 | 0.992 | 0.9888 |
| 107 | 1 | 1 | 1 | 1 | 1 | 1 | 0.995 | 0.996 | 0.949 | 1 | 0.994 | 0.987 | 0.983 | 1 | 0.986 | 0.988 | 0.963 | 1 | 0.994 | 0.9877 |
| 108 | 1 | 1 | 1 | 1 | 1 | 1 | 0.995 | 0.996 | 0.949 | 1 | 0.994 | 0.987 | 0.983 | 1 | 0.986 | 0.988 | 0.968 | 1 | 0.994 | 0.9888 |
| 109 | 1 | 1 | 1 | 1 | 1 | 1 | 0.995 | 0.996 | 0.949 | 1 | 0.994 | 0.987 | 0.983 | 1 | 0.986 | 0.988 | 0.979 | 1 | 0.992 | 0.9899 |
| 110 | 1 | 1 | 1 | 1 | 1 | 1 | 0.995 | 0.996 | 0.949 | 1 | 0.994 | 0.987 | 1 | 1 | 0.986 | 0.99 | 0.973 | 1 | 0.994 | 0.9899 |
| 111 | 1 | 1 | 1 | 1 | 1 | 1 | 0.995 | 0.996 | 0.949 | 1 | 0.994 | 0.987 | 1 | 1 | 0.986 | 0.99 | 0.963 | 1 | 0.994 | 0.9877 |
| 112 | 1 | 1 | 1 | 1 | 1 | 1 | 0.995 | 0.996 | 0.924 | 1 | 0.994 | 0.983 | 0.983 | 1 | 0.992 | 0.992 | 0.957 | 1 | 0.99 | 0.9843 |
| 113 | 1 | 1 | 1 | 1 | 1 | 1 | 0.995 | 0.996 | 0.924 | 1 | 0.994 | 0.983 | 0.983 | 1 | 0.986 | 0.988 | 0.957 | 1 | 0.992 | 0.9854 |
| 114 | 1 | 1 | 1 | 1 | 0.988 | 1 | 0.995 | 0.994 | 0.949 | 1 | 0.994 | 0.987 | 0.983 | 1 | 0.986 | 0.988 | 0.968 | 1 | 0.992 | 0.9877 |
| 115 | 1 | 1 | 1 | 1 | 0.988 | 1 | 0.995 | 0.994 | 0.949 | 1 | 0.994 | 0.987 | 0.983 | 1 | 0.989 | 0.99 | 0.968 | 1 | 0.992 | 0.9877 |
| 116 | 1 | 1 | 1 | 1 | 0.988 | 1 | 0.995 | 0.994 | 0.949 | 1 | 0.994 | 0.987 | 0.983 | 1 | 0.992 | 0.992 | 0.963 | 1 | 0.99 | 0.9854 |
| 117 | 1 | 1 | 1 | 1 | 0.988 | 1 | 0.995 | 0.994 | 0.949 | 1 | 0.994 | 0.987 | 0.983 | 1 | 0.989 | 0.99 | 0.963 | 1 | 0.99 | 0.9854 |
| 118 | 1 | 1 | 1 | 1 | 0.988 | 1 | 0.995 | 0.994 | 0.949 | 1 | 0.994 | 0.987 | 0.983 | 1 | 0.989 | 0.99 | 0.963 | 1 | 0.99 | 0.9854 |
| 119 | 1 | 1 | 1 | 1 | 0.988 | 1 | 0.995 | 0.994 | 0.949 | 1 | 0.994 | 0.987 | 0.983 | 1 | 0.989 | 0.99 | 0.963 | 1 | 0.99 | 0.9854 |
| 120 | 1 | 1 | 1 | 1 | 0.988 | 1 | 0.995 | 0.994 | 0.949 | 1 | 0.994 | 0.987 | 0.983 | 1 | 0.989 | 0.99 | 0.963 | 1 | 0.99 | 0.9854 |
| 121 | 1 | 1 | 1 | 1 | 0.988 | 1 | 0.995 | 0.994 | 0.937 | 1 | 0.991 | 0.983 | 0.966 | 1 | 0.992 | 0.99 | 0.968 | 1 | 0.992 | 0.9877 |
| 122 | 1 | 1 | 1 | 1 | 0.988 | 1 | 0.995 | 0.994 | 0.949 | 1 | 0.991 | 0.985 | 0.966 | 1 | 0.992 | 0.99 | 0.968 | 1 | 0.992 | 0.9877 |
| 123 | 1 | 1 | 1 | 1 | 0.988 | 1 | 0.995 | 0.994 | 0.949 | 1 | 0.991 | 0.985 | 0.966 | 1 | 0.992 | 0.99 | 0.968 | 1 | 0.992 | 0.9877 |
| 124 | 1 | 1 | 1 | 1 | 0.988 | 1 | 0.995 | 0.994 | 0.949 | 1 | 0.991 | 0.985 | 0.966 | 1 | 0.989 | 0.988 | 0.968 | 1 | 0.992 | 0.9877 |
| 125 | 1 | 1 | 1 | 1 | 0.988 | 1 | 0.995 | 0.994 | 0.949 | 1 | 0.991 | 0.985 | 0.966 | 1 | 0.989 | 0.988 | 0.968 | 1 | 0.992 | 0.9877 |
| 126 | 1 | 1 | 1 | 1 | 0.988 | 1 | 0.995 | 0.994 | 0.949 | 1 | 0.991 | 0.985 | 0.966 | 1 | 0.989 | 0.988 | 0.968 | 1 | 0.99 | 0.9866 |
| 127 | 1 | 1 | 1 | 1 | 0.988 | 1 | 0.995 | 0.994 | 0.949 | 1 | 0.991 | 0.985 | 0.966 | 1 | 0.989 | 0.988 | 0.968 | 1 | 0.992 | 0.9877 |
| 128 | 1 | 1 | 1 | 1 | 0.988 | 1 | 0.995 | 0.994 | 0.949 | 1 | 0.991 | 0.985 | 0.966 | 1 | 0.992 | 0.99 | 0.968 | 1 | 0.99 | 0.9866 |
| 129 | 1 | 1 | 1 | 1 | 0.988 | 1 | 0.995 | 0.994 | 0.949 | 1 | 0.994 | 0.987 | 0.966 | 1 | 0.992 | 0.99 | 0.968 | 1 | 0.992 | 0.9877 |
| 130 | 1 | 1 | 1 | 1 | 0.988 | 1 | 0.995 | 0.994 | 0.949 | 1 | 0.994 | 0.987 | 0.966 | 1 | 0.989 | 0.988 | 0.968 | 1 | 0.992 | 0.9877 |
| 131 | 1 | 1 | 1 | 1 | 0.988 | 1 | 0.995 | 0.994 | 0.937 | 1 | 0.994 | 0.985 | 0.966 | 1 | 0.989 | 0.988 | 0.968 | 1 | 0.994 | 0.9888 |
| 132 | 1 | 1 | 1 | 1 | 0.988 | 1 | 0.995 | 0.994 | 0.937 | 1 | 0.991 | 0.983 | 0.966 | 1 | 0.989 | 0.988 | 0.968 | 1 | 0.994 | 0.9888 |
| 133 | 1 | 1 | 1 | 1 | 0.988 | 1 | 0.995 | 0.994 | 0.949 | 1 | 0.991 | 0.985 | 0.966 | 1 | 0.986 | 0.986 | 0.968 | 1 | 0.99 | 0.9866 |
| 134 | 1 | 1 | 1 | 1 | 0.988 | 1 | 0.995 | 0.994 | 0.949 | 1 | 0.991 | 0.985 | 0.966 | 1 | 0.986 | 0.986 | 0.968 | 1 | 0.992 | 0.9877 |
| 135 | 1 | 1 | 1 | 1 | 0.988 | 1 | 0.995 | 0.994 | 0.924 | 1 | 0.991 | 0.981 | 0.966 | 1 | 0.986 | 0.986 | 0.968 | 1 | 0.994 | 0.9888 |
| 136 | 1 | 1 | 0.997 | 0.997 | 0.988 | 1 | 0.995 | 0.994 | 0.937 | 1 | 0.991 | 0.983 | 0.966 | 1 | 0.986 | 0.986 | 0.968 | 1 | 0.992 | 0.9877 |
| 137 | 1 | 1 | 0.997 | 0.997 | 0.988 | 1 | 0.993 | 0.992 | 0.949 | 1 | 0.991 | 0.985 | 0.966 | 1 | 0.986 | 0.986 | 0.968 | 1 | 0.992 | 0.9877 |
| 138 | 1 | 1 | 0.997 | 0.997 | 0.988 | 1 | 0.998 | 0.996 | 0.937 | 1 | 0.988 | 0.981 | 0.966 | 1 | 0.981 | 0.982 | 0.968 | 1 | 0.99 | 0.9866 |
| 139 | 1 | 1 | 0.997 | 0.997 | 0.988 | 1 | 0.998 | 0.996 | 0.937 | 1 | 0.988 | 0.981 | 0.966 | 1 | 0.981 | 0.982 | 0.968 | 1 | 0.99 | 0.9866 |
| 140 | 1 | 1 | 0.997 | 0.997 | 0.988 | 1 | 0.998 | 0.996 | 0.937 | 1 | 0.988 | 0.981 | 0.966 | 1 | 0.981 | 0.982 | 0.968 | 1 | 0.99 | 0.9866 |
| 141 | 1 | 1 | 0.997 | 0.997 | 0.988 | 1 | 0.998 | 0.996 | 0.937 | 1 | 0.991 | 0.983 | 0.949 | 1 | 0.983 | 0.982 | 0.968 | 1 | 0.992 | 0.9877 |
| 142 | 1 | 1 | 0.997 | 0.997 | 0.988 | 1 | 0.998 | 0.996 | 0.937 | 1 | 0.991 | 0.983 | 0.949 | 1 | 0.983 | 0.982 | 0.968 | 1 | 0.992 | 0.9877 |
| 143 | 1 | 1 | 0.997 | 0.997 | 0.988 | 1 | 0.998 | 0.996 | 0.937 | 1 | 0.991 | 0.983 | 0.949 | 1 | 0.983 | 0.982 | 0.968 | 1 | 0.99 | 0.9866 |
| 144 | 1 | 1 | 0.997 | 0.997 | 0.988 | 1 | 0.998 | 0.996 | 0.937 | 1 | 0.991 | 0.983 | 0.949 | 1 | 0.983 | 0.982 | 0.968 | 1 | 0.99 | 0.9866 |
| 145 | 1 | 1 | 0.997 | 0.997 | 0.988 | 1 | 0.995 | 0.994 | 0.899 | 1 | 0.988 | 0.974 | 0.915 | 1 | 0.983 | 0.978 | 0.968 | 1 | 0.989 | 0.9854 |
| 146 | 1 | 1 | 0.997 | 0.997 | 0.988 | 1 | 0.995 | 0.994 | 0.899 | 1 | 0.991 | 0.977 | 0.915 | 1 | 0.983 | 0.978 | 0.963 | 1 | 0.989 | 0.9843 |
| 147 | 1 | 1 | 0.997 | 0.997 | 0.988 | 1 | 0.995 | 0.994 | 0.899 | 1 | 0.991 | 0.977 | 0.915 | 1 | 0.983 | 0.978 | 0.968 | 1 | 0.989 | 0.9854 |
| 148 | 1 | 1 | 0.997 | 0.997 | 0.988 | 1 | 0.998 | 0.996 | 0.899 | 1 | 0.991 | 0.977 | 0.915 | 1 | 0.986 | 0.98 | 0.963 | 1 | 0.99 | 0.9854 |
| 149 | 1 | 1 | 0.997 | 0.997 | 0.988 | 1 | 0.998 | 0.996 | 0.924 | 1 | 0.988 | 0.979 | 0.932 | 1 | 0.986 | 0.982 | 0.963 | 1 | 0.99 | 0.9854 |
| 150 | 1 | 1 | 0.997 | 0.997 | 0.988 | 1 | 0.998 | 0.996 | 0.924 | 1 | 0.988 | 0.979 | 0.932 | 1 | 0.986 | 0.982 | 0.963 | 1 | 0.99 | 0.9854 |
| 151 | 1 | 1 | 0.997 | 0.997 | 0.988 | 1 | 0.998 | 0.996 | 0.911 | 1 | 0.988 | 0.977 | 0.915 | 1 | 0.986 | 0.98 | 0.963 | 1 | 0.99 | 0.9854 |
| 152 | 1 | 1 | 0.997 | 0.997 | 0.988 | 1 | 0.998 | 0.996 | 0.911 | 1 | 0.988 | 0.977 | 0.932 | 1 | 0.986 | 0.982 | 0.963 | 1 | 0.992 | 0.9866 |
| 153 | 1 | 1 | 0.997 | 0.997 | 0.988 | 1 | 0.998 | 0.996 | 0.911 | 1 | 0.988 | 0.977 | 0.932 | 0.988 | 0.986 | 0.98 | 0.968 | 1 | 0.992 | 0.9877 |
| 154 | 1 | 1 | 0.997 | 0.997 | 0.988 | 1 | 0.998 | 0.996 | 0.899 | 1 | 0.994 | 0.979 | 0.898 | 1 | 0.986 | 0.978 | 0.963 | 1 | 0.992 | 0.9866 |
| 155 | 1 | 1 | 0.997 | 0.997 | 0.988 | 1 | 0.998 | 0.996 | 0.886 | 1 | 0.994 | 0.977 | 0.898 | 1 | 0.986 | 0.978 | 0.963 | 1 | 0.992 | 0.9866 |
| 156 | 1 | 1 | 0.997 | 0.997 | 0.988 | 1 | 0.998 | 0.996 | 0.886 | 1 | 0.994 | 0.977 | 0.915 | 1 | 0.986 | 0.98 | 0.963 | 1 | 0.992 | 0.9866 |
| 157 | 1 | 1 | 0.997 | 0.997 | 0.988 | 1 | 0.998 | 0.996 | 0.886 | 1 | 0.997 | 0.979 | 0.915 | 0.988 | 0.986 | 0.978 | 0.963 | 1 | 0.992 | 0.9866 |
| 158 | 1 | 1 | 0.997 | 0.997 | 0.988 | 1 | 0.998 | 0.996 | 0.873 | 1 | 0.997 | 0.977 | 0.898 | 0.988 | 0.986 | 0.976 | 0.968 | 1 | 0.992 | 0.9877 |
| 159 | 1 | 1 | 0.997 | 0.997 | 0.988 | 1 | 0.995 | 0.994 | 0.886 | 1 | 0.997 | 0.979 | 0.898 | 0.988 | 0.986 | 0.976 | 0.968 | 1 | 0.992 | 0.9877 |
| 160 | 1 | 1 | 0.997 | 0.997 | 0.988 | 1 | 0.995 | 0.994 | 0.873 | 1 | 0.997 | 0.977 | 0.898 | 0.988 | 0.986 | 0.976 | 0.968 | 1 | 0.992 | 0.9877 |
| 161 | 1 | 1 | 0.997 | 0.997 | 0.988 | 1 | 0.995 | 0.994 | 0.873 | 1 | 0.997 | 0.977 | 0.898 | 0.988 | 0.986 | 0.976 | 0.968 | 1 | 0.992 | 0.9877 |
| 162 | 1 | 1 | 0.997 | 0.997 | 0.988 | 1 | 0.995 | 0.994 | 0.873 | 1 | 0.997 | 0.977 | 0.898 | 0.988 | 0.986 | 0.976 | 0.968 | 1 | 0.992 | 0.9877 |
| 163 | 1 | 1 | 0.997 | 0.997 | 0.988 | 1 | 0.995 | 0.994 | 0.861 | 1 | 0.997 | 0.974 | 0.881 | 0.988 | 0.986 | 0.974 | 0.968 | 1 | 0.992 | 0.9877 |
| 164 | 1 | 1 | 0.997 | 0.997 | 0.988 | 1 | 0.995 | 0.994 | 0.873 | 1 | 0.997 | 0.977 | 0.881 | 0.988 | 0.986 | 0.974 | 0.968 | 1 | 0.992 | 0.9877 |
| 165 | 1 | 1 | 0.997 | 0.997 | 0.988 | 1 | 0.995 | 0.994 | 0.861 | 1 | 0.997 | 0.974 | 0.881 | 0.988 | 0.986 | 0.974 | 0.968 | 1 | 0.992 | 0.9877 |
| 166 | 1 | 1 | 0.997 | 0.997 | 0.988 | 1 | 0.995 | 0.994 | 0.873 | 1 | 0.997 | 0.977 | 0.881 | 0.988 | 0.986 | 0.974 | 0.957 | 1 | 0.992 | 0.9854 |
| 167 | 1 | 1 | 0.997 | 0.997 | 0.988 | 1 | 0.995 | 0.994 | 0.873 | 1 | 0.997 | 0.977 | 0.881 | 0.988 | 0.986 | 0.974 | 0.952 | 1 | 0.992 | 0.9843 |
| 168 | 1 | 1 | 0.997 | 0.997 | 0.988 | 1 | 0.995 | 0.994 | 0.861 | 1 | 0.997 | 0.974 | 0.881 | 0.988 | 0.983 | 0.972 | 0.963 | 1 | 0.992 | 0.9866 |
| 169 | 1 | 1 | 0.997 | 0.997 | 0.988 | 1 | 0.995 | 0.994 | 0.861 | 1 | 0.997 | 0.974 | 0.881 | 0.988 | 0.983 | 0.972 | 0.968 | 1 | 0.992 | 0.9877 |
| 170 | 1 | 1 | 0.997 | 0.997 | 0.988 | 1 | 0.998 | 0.996 | 0.861 | 1 | 0.997 | 0.974 | 0.881 | 0.988 | 0.986 | 0.974 | 0.968 | 1 | 0.992 | 0.9877 |
| 171 | 1 | 1 | 0.997 | 0.997 | 0.988 | 1 | 0.998 | 0.996 | 0.861 | 1 | 0.997 | 0.974 | 0.881 | 0.988 | 0.986 | 0.974 | 0.968 | 1 | 0.992 | 0.9877 |
| 172 | 1 | 1 | 0.997 | 0.997 | 0.988 | 1 | 0.998 | 0.996 | 0.861 | 1 | 0.997 | 0.974 | 0.881 | 0.988 | 0.986 | 0.974 | 0.957 | 1 | 0.992 | 0.9854 |
| 173 | 1 | 1 | 0.997 | 0.997 | 0.988 | 1 | 0.998 | 0.996 | 0.861 | 1 | 0.997 | 0.974 | 0.881 | 0.988 | 0.986 | 0.974 | 0.963 | 1 | 0.992 | 0.9866 |
| 174 | 1 | 1 | 0.997 | 0.997 | 0.988 | 1 | 0.998 | 0.996 | 0.861 | 1 | 0.997 | 0.974 | 0.881 | 0.988 | 0.986 | 0.974 | 0.963 | 1 | 0.992 | 0.9866 |
| 175 | 1 | 1 | 0.997 | 0.997 | 0.988 | 1 | 0.998 | 0.996 | 0.861 | 1 | 0.997 | 0.974 | 0.881 | 0.988 | 0.986 | 0.974 | 0.957 | 1 | 0.992 | 0.9854 |
| 176 | 1 | 1 | 0.997 | 0.997 | 0.988 | 1 | 0.998 | 0.996 | 0.861 | 1 | 0.997 | 0.974 | 0.881 | 0.988 | 0.986 | 0.974 | 0.957 | 1 | 0.992 | 0.9854 |
| 177 | 1 | 1 | 0.997 | 0.997 | 0.988 | 1 | 0.998 | 0.996 | 0.861 | 1 | 0.997 | 0.974 | 0.881 | 0.988 | 0.986 | 0.974 | 0.957 | 1 | 0.992 | 0.9854 |
| 178 | 1 | 1 | 0.997 | 0.997 | 0.988 | 1 | 0.998 | 0.996 | 0.873 | 1 | 0.994 | 0.974 | 0.881 | 0.988 | 0.986 | 0.974 | 0.963 | 1 | 0.994 | 0.9877 |
| 179 | 1 | 1 | 0.997 | 0.997 | 0.988 | 1 | 0.998 | 0.996 | 0.873 | 1 | 0.994 | 0.974 | 0.864 | 0.988 | 0.989 | 0.974 | 0.968 | 1 | 0.994 | 0.9888 |
| 180 | 1 | 1 | 0.997 | 0.997 | 0.988 | 1 | 0.998 | 0.996 | 0.873 | 1 | 0.994 | 0.974 | 0.847 | 0.988 | 0.989 | 0.972 | 0.968 | 1 | 0.992 | 0.9877 |
| 181 | 1 | 1 | 0.997 | 0.997 | 0.988 | 1 | 0.998 | 0.996 | 0.873 | 1 | 0.994 | 0.974 | 0.847 | 0.988 | 0.989 | 0.972 | 0.968 | 1 | 0.992 | 0.9877 |
| 182 | 1 | 1 | 0.997 | 0.997 | 0.988 | 1 | 0.998 | 0.996 | 0.873 | 1 | 0.994 | 0.974 | 0.864 | 0.988 | 0.989 | 0.974 | 0.963 | 1 | 0.995 | 0.9888 |
| 183 | 1 | 1 | 0.997 | 0.997 | 0.988 | 1 | 0.998 | 0.996 | 0.873 | 1 | 0.994 | 0.974 | 0.864 | 0.988 | 0.989 | 0.974 | 0.963 | 1 | 0.995 | 0.9888 |
| 184 | 1 | 1 | 0.997 | 0.997 | 0.988 | 1 | 0.998 | 0.996 | 0.873 | 1 | 0.994 | 0.974 | 0.864 | 0.988 | 0.989 | 0.974 | 0.963 | 1 | 0.995 | 0.9888 |
| 185 | 1 | 1 | 0.997 | 0.997 | 0.988 | 1 | 0.998 | 0.996 | 0.873 | 1 | 0.994 | 0.974 | 0.864 | 0.988 | 0.989 | 0.974 | 0.963 | 1 | 0.995 | 0.9888 |
| 186 | 1 | 1 | 0.997 | 0.997 | 0.988 | 1 | 0.998 | 0.996 | 0.873 | 1 | 0.994 | 0.974 | 0.864 | 0.988 | 0.989 | 0.974 | 0.963 | 1 | 0.995 | 0.9888 |
| 187 | 1 | 1 | 0.997 | 0.997 | 0.988 | 1 | 0.998 | 0.996 | 0.873 | 1 | 0.994 | 0.974 | 0.864 | 0.988 | 0.989 | 0.974 | 0.963 | 1 | 0.995 | 0.9888 |
| 188 | 1 | 1 | 0.997 | 0.997 | 0.988 | 1 | 0.998 | 0.996 | 0.873 | 1 | 0.994 | 0.974 | 0.864 | 0.988 | 0.989 | 0.974 | 0.963 | 1 | 0.995 | 0.9888 |
| 189 | 1 | 1 | 0.997 | 0.997 | 0.988 | 1 | 0.998 | 0.996 | 0.873 | 1 | 0.994 | 0.974 | 0.864 | 0.988 | 0.989 | 0.974 | 0.963 | 1 | 0.995 | 0.9888 |
| 190 | 1 | 1 | 0.997 | 0.997 | 0.988 | 1 | 0.998 | 0.996 | 0.873 | 1 | 0.994 | 0.974 | 0.864 | 0.988 | 0.989 | 0.974 | 0.963 | 1 | 0.995 | 0.9888 |
| 191 | 1 | 1 | 0.997 | 0.997 | 0.988 | 1 | 0.998 | 0.996 | 0.911 | 1 | 0.994 | 0.981 | 0.864 | 0.988 | 0.989 | 0.974 | 0.968 | 1 | 0.994 | 0.9888 |
| 192 | 1 | 1 | 0.997 | 0.997 | 0.988 | 1 | 0.995 | 0.994 | 0.886 | 1 | 0.991 | 0.974 | 0.881 | 0.988 | 0.986 | 0.974 | 0.973 | 1 | 0.992 | 0.9888 |
| 193 | 1 | 1 | 0.997 | 0.997 | 0.988 | 1 | 0.995 | 0.994 | 0.911 | 1 | 0.991 | 0.979 | 0.881 | 0.988 | 0.989 | 0.976 | 0.973 | 1 | 0.992 | 0.9888 |
| 194 | 1 | 1 | 0.997 | 0.997 | 0.988 | 1 | 0.998 | 0.996 | 0.899 | 1 | 0.991 | 0.977 | 0.881 | 0.988 | 0.989 | 0.976 | 0.957 | 1 | 0.994 | 0.9866 |
| 195 | 1 | 1 | 0.997 | 0.997 | 0.988 | 1 | 0.998 | 0.996 | 0.899 | 1 | 0.991 | 0.977 | 0.881 | 0.988 | 0.989 | 0.976 | 0.957 | 1 | 0.995 | 0.9877 |
| 196 | 1 | 1 | 0.997 | 0.997 | 0.988 | 1 | 0.995 | 0.994 | 0.886 | 1 | 0.991 | 0.974 | 0.864 | 0.988 | 0.989 | 0.974 | 0.957 | 1 | 0.995 | 0.9877 |
| 197 | 1 | 1 | 0.997 | 0.997 | 0.988 | 1 | 0.995 | 0.994 | 0.886 | 1 | 0.991 | 0.974 | 0.864 | 0.988 | 0.989 | 0.974 | 0.957 | 1 | 0.995 | 0.9877 |
| 198 | 1 | 1 | 0.997 | 0.997 | 0.988 | 1 | 0.995 | 0.994 | 0.886 | 1 | 0.991 | 0.974 | 0.864 | 0.988 | 0.989 | 0.974 | 0.957 | 1 | 0.994 | 0.9866 |
| 199 | 1 | 1 | 0.997 | 0.997 | 0.988 | 1 | 0.995 | 0.994 | 0.886 | 1 | 0.991 | 0.974 | 0.864 | 0.988 | 0.989 | 0.974 | 0.957 | 1 | 0.994 | 0.9866 |
| 200 | 1 | 1 | 0.997 | 0.997 | 0.988 | 1 | 0.995 | 0.994 | 0.873 | 1 | 0.991 | 0.972 | 0.864 | 0.988 | 0.989 | 0.974 | 0.957 | 1 | 0.994 | 0.9866 |
| 201 | 1 | 1 | 0.997 | 0.997 | 0.988 | 1 | 0.995 | 0.994 | 0.873 | 1 | 0.991 | 0.972 | 0.864 | 0.988 | 0.989 | 0.974 | 0.957 | 1 | 0.994 | 0.9866 |
| 202 | 1 | 1 | 0.997 | 0.997 | 0.988 | 1 | 0.995 | 0.994 | 0.886 | 1 | 0.994 | 0.977 | 0.864 | 0.988 | 0.989 | 0.974 | 0.957 | 1 | 0.995 | 0.9877 |
| 203 | 1 | 1 | 0.997 | 0.997 | 0.988 | 1 | 0.995 | 0.994 | 0.886 | 1 | 0.994 | 0.977 | 0.864 | 0.988 | 0.989 | 0.974 | 0.957 | 1 | 0.995 | 0.9877 |
| 204 | 1 | 1 | 0.997 | 0.997 | 0.988 | 1 | 0.998 | 0.996 | 0.886 | 1 | 0.994 | 0.977 | 0.864 | 0.988 | 0.989 | 0.974 | 0.963 | 1 | 0.995 | 0.9888 |
| 205 | 1 | 1 | 0.997 | 0.997 | 0.988 | 1 | 0.998 | 0.996 | 0.873 | 1 | 0.994 | 0.974 | 0.864 | 0.988 | 0.989 | 0.974 | 0.963 | 1 | 0.995 | 0.9888 |
| 206 | 1 | 1 | 0.997 | 0.997 | 0.988 | 1 | 0.998 | 0.996 | 0.873 | 1 | 0.991 | 0.972 | 0.864 | 0.988 | 0.989 | 0.974 | 0.973 | 1 | 0.995 | 0.991 |
| 207 | 1 | 1 | 0.997 | 0.997 | 0.988 | 1 | 0.995 | 0.994 | 0.873 | 1 | 0.991 | 0.972 | 0.864 | 0.988 | 0.989 | 0.974 | 0.973 | 1 | 0.995 | 0.991 |
| 208 | 1 | 1 | 0.997 | 0.997 | 0.988 | 1 | 0.995 | 0.994 | 0.886 | 1 | 0.991 | 0.974 | 0.864 | 0.988 | 0.989 | 0.974 | 0.973 | 1 | 0.995 | 0.991 |
| 209 | 1 | 1 | 0.997 | 0.997 | 0.988 | 1 | 0.995 | 0.994 | 0.873 | 1 | 0.991 | 0.972 | 0.864 | 0.988 | 0.989 | 0.974 | 0.973 | 1 | 0.995 | 0.991 |
| 210 | 1 | 1 | 0.997 | 0.997 | 0.988 | 1 | 0.995 | 0.994 | 0.886 | 1 | 0.991 | 0.974 | 0.864 | 0.988 | 0.989 | 0.974 | 0.973 | 1 | 0.995 | 0.991 |
| 211 | 1 | 1 | 0.997 | 0.997 | 0.988 | 1 | 0.995 | 0.994 | 0.899 | 1 | 0.991 | 0.977 | 0.864 | 0.988 | 0.989 | 0.974 | 0.973 | 1 | 0.995 | 0.991 |
| 212 | 1 | 1 | 0.997 | 0.997 | 0.988 | 1 | 0.998 | 0.996 | 0.899 | 1 | 0.991 | 0.977 | 0.864 | 0.975 | 0.989 | 0.972 | 0.973 | 1 | 0.995 | 0.991 |
| 213 | 1 | 1 | 0.997 | 0.997 | 0.988 | 1 | 0.995 | 0.994 | 0.911 | 1 | 0.991 | 0.979 | 0.864 | 0.975 | 0.989 | 0.972 | 0.973 | 1 | 0.995 | 0.991 |
| 214 | 1 | 1 | 0.997 | 0.997 | 0.988 | 1 | 0.995 | 0.994 | 0.911 | 1 | 0.991 | 0.979 | 0.864 | 0.975 | 0.989 | 0.972 | 0.973 | 1 | 0.995 | 0.991 |
| 215 | 1 | 1 | 0.997 | 0.997 | 0.988 | 1 | 0.995 | 0.994 | 0.911 | 1 | 0.991 | 0.979 | 0.864 | 0.975 | 0.989 | 0.972 | 0.973 | 1 | 0.995 | 0.991 |
| 216 | 1 | 1 | 0.997 | 0.997 | 0.988 | 1 | 0.995 | 0.994 | 0.911 | 1 | 0.991 | 0.979 | 0.864 | 0.975 | 0.989 | 0.972 | 0.973 | 1 | 0.995 | 0.991 |
| 217 | 1 | 1 | 0.997 | 0.997 | 0.988 | 1 | 0.995 | 0.994 | 0.911 | 1 | 0.991 | 0.979 | 0.881 | 0.975 | 0.989 | 0.974 | 0.973 | 1 | 0.995 | 0.991 |
| 218 | 1 | 1 | 0.997 | 0.997 | 0.988 | 1 | 0.995 | 0.994 | 0.911 | 1 | 0.991 | 0.979 | 0.881 | 0.975 | 0.989 | 0.974 | 0.973 | 1 | 0.995 | 0.991 |
| 219 | 1 | 1 | 0.997 | 0.997 | 0.988 | 1 | 0.995 | 0.994 | 0.911 | 1 | 0.991 | 0.979 | 0.881 | 0.975 | 0.989 | 0.974 | 0.973 | 1 | 0.994 | 0.9899 |
| 220 | 1 | 1 | 0.997 | 0.997 | 0.988 | 1 | 0.995 | 0.994 | 0.911 | 1 | 0.991 | 0.979 | 0.881 | 0.975 | 0.989 | 0.974 | 0.979 | 1 | 0.994 | 0.991 |
| 221 | 1 | 1 | 0.997 | 0.997 | 0.988 | 1 | 1 | 0.998 | 0.911 | 1 | 0.991 | 0.979 | 0.881 | 0.975 | 0.989 | 0.974 | 0.979 | 1 | 0.994 | 0.991 |
| 222 | 1 | 1 | 0.997 | 0.997 | 0.988 | 1 | 0.998 | 0.996 | 0.911 | 1 | 0.991 | 0.979 | 0.881 | 0.975 | 0.989 | 0.974 | 0.979 | 1 | 0.994 | 0.991 |
| 223 | 1 | 1 | 0.997 | 0.997 | 0.988 | 1 | 0.998 | 0.996 | 0.911 | 1 | 0.991 | 0.979 | 0.881 | 0.988 | 0.989 | 0.976 | 0.979 | 1 | 0.994 | 0.991 |
| 224 | 1 | 1 | 0.997 | 0.997 | 0.988 | 1 | 0.998 | 0.996 | 0.911 | 1 | 0.994 | 0.981 | 0.881 | 0.988 | 0.989 | 0.976 | 0.979 | 1 | 0.995 | 0.9922 |
| 225 | 1 | 1 | 0.997 | 0.997 | 0.988 | 1 | 0.998 | 0.996 | 0.911 | 1 | 0.994 | 0.981 | 0.881 | 0.988 | 0.989 | 0.976 | 0.979 | 1 | 0.995 | 0.9922 |
| 226 | 1 | 1 | 0.997 | 0.997 | 0.988 | 1 | 0.998 | 0.996 | 0.911 | 1 | 0.994 | 0.981 | 0.881 | 0.988 | 0.989 | 0.976 | 0.979 | 1 | 0.995 | 0.9922 |
| 227 | 1 | 1 | 0.997 | 0.997 | 0.988 | 1 | 0.998 | 0.996 | 0.911 | 1 | 0.994 | 0.981 | 0.881 | 0.988 | 0.989 | 0.976 | 0.979 | 1 | 0.995 | 0.9922 |
| 228 | 1 | 1 | 0.997 | 0.997 | 0.988 | 1 | 0.998 | 0.996 | 0.911 | 1 | 0.994 | 0.981 | 0.881 | 0.988 | 0.989 | 0.976 | 0.979 | 1 | 0.995 | 0.9922 |
| 229 | 1 | 1 | 0.997 | 0.997 | 0.988 | 1 | 1 | 0.998 | 0.911 | 1 | 0.994 | 0.981 | 0.881 | 0.988 | 0.989 | 0.976 | 0.979 | 1 | 0.995 | 0.9922 |
| 230 | 1 | 1 | 0.997 | 0.997 | 0.988 | 1 | 1 | 0.998 | 0.911 | 1 | 0.994 | 0.981 | 0.881 | 0.988 | 0.989 | 0.976 | 0.979 | 1 | 0.995 | 0.9922 |
| 231 | 1 | 1 | 0.997 | 0.997 | 0.988 | 1 | 1 | 0.998 | 0.911 | 1 | 0.994 | 0.981 | 0.881 | 0.988 | 0.992 | 0.978 | 0.979 | 1 | 0.995 | 0.9922 |
| 232 | 1 | 1 | 0.997 | 0.997 | 0.988 | 1 | 1 | 0.998 | 0.911 | 1 | 0.994 | 0.981 | 0.864 | 0.988 | 0.997 | 0.98 | 0.963 | 1 | 0.995 | 0.9888 |
| 233 | 1 | 1 | 0.997 | 0.997 | 0.988 | 1 | 1 | 0.998 | 0.911 | 1 | 0.994 | 0.981 | 0.864 | 0.988 | 0.994 | 0.978 | 0.968 | 1 | 0.995 | 0.9899 |
| 234 | 1 | 1 | 0.997 | 0.997 | 0.988 | 1 | 1 | 0.998 | 0.911 | 1 | 0.994 | 0.981 | 0.881 | 0.988 | 0.994 | 0.98 | 0.968 | 1 | 0.995 | 0.9899 |
| 235 | 1 | 1 | 0.997 | 0.997 | 0.988 | 1 | 1 | 0.998 | 0.911 | 1 | 0.994 | 0.981 | 0.881 | 0.988 | 0.994 | 0.98 | 0.963 | 1 | 0.995 | 0.9888 |
| 236 | 1 | 1 | 0.997 | 0.997 | 0.988 | 1 | 1 | 0.998 | 0.911 | 1 | 0.994 | 0.981 | 0.881 | 0.988 | 0.997 | 0.982 | 0.957 | 1 | 0.995 | 0.9877 |
| 237 | 1 | 1 | 0.997 | 0.997 | 0.988 | 1 | 1 | 0.998 | 0.911 | 1 | 0.994 | 0.981 | 0.881 | 0.988 | 0.994 | 0.98 | 0.952 | 1 | 0.995 | 0.9866 |
| 238 | 1 | 1 | 0.997 | 0.997 | 0.988 | 1 | 1 | 0.998 | 0.886 | 1 | 0.994 | 0.977 | 0.864 | 0.988 | 0.997 | 0.98 | 0.957 | 1 | 0.995 | 0.9877 |
| 239 | 1 | 1 | 0.997 | 0.997 | 0.988 | 1 | 1 | 0.998 | 0.886 | 1 | 0.994 | 0.977 | 0.864 | 0.988 | 0.997 | 0.98 | 0.957 | 1 | 0.995 | 0.9877 |
| 240 | 1 | 1 | 0.997 | 0.997 | 0.988 | 1 | 1 | 0.998 | 0.886 | 1 | 0.994 | 0.977 | 0.864 | 0.988 | 0.997 | 0.98 | 0.957 | 1 | 0.995 | 0.9877 |
| 241 | 1 | 1 | 0.997 | 0.997 | 0.988 | 1 | 1 | 0.998 | 0.886 | 1 | 0.994 | 0.977 | 0.864 | 0.988 | 0.997 | 0.98 | 0.957 | 1 | 0.995 | 0.9877 |
| 242 | 1 | 1 | 0.997 | 0.997 | 0.988 | 1 | 1 | 0.998 | 0.911 | 1 | 0.994 | 0.981 | 0.881 | 0.988 | 0.997 | 0.982 | 0.973 | 1 | 0.995 | 0.991 |
| 243 | 1 | 1 | 0.997 | 0.997 | 0.988 | 1 | 1 | 0.998 | 0.911 | 1 | 0.994 | 0.981 | 0.881 | 0.988 | 0.994 | 0.98 | 0.968 | 1 | 0.995 | 0.9899 |
| 244 | 1 | 1 | 0.997 | 0.997 | 0.988 | 1 | 1 | 0.998 | 0.886 | 1 | 0.994 | 0.977 | 0.881 | 0.988 | 0.992 | 0.978 | 0.968 | 1 | 0.995 | 0.9899 |
| 245 | 1 | 1 | 0.997 | 0.997 | 0.988 | 1 | 1 | 0.998 | 0.886 | 1 | 0.994 | 0.977 | 0.881 | 0.988 | 0.997 | 0.982 | 0.968 | 1 | 0.995 | 0.9899 |
| 246 | 1 | 1 | 0.997 | 0.997 | 0.988 | 1 | 1 | 0.998 | 0.886 | 1 | 0.994 | 0.977 | 0.881 | 0.988 | 0.994 | 0.98 | 0.968 | 1 | 0.995 | 0.9899 |
| 247 | 1 | 1 | 0.997 | 0.997 | 0.988 | 1 | 1 | 0.998 | 0.899 | 1 | 0.994 | 0.979 | 0.881 | 0.988 | 0.994 | 0.98 | 0.952 | 1 | 0.997 | 0.9877 |
| 248 | 1 | 1 | 0.997 | 0.997 | 0.988 | 1 | 1 | 0.998 | 0.911 | 1 | 0.994 | 0.981 | 0.881 | 0.988 | 0.994 | 0.98 | 0.957 | 1 | 0.997 | 0.9888 |
| 249 | 1 | 1 | 0.997 | 0.997 | 0.988 | 1 | 1 | 0.998 | 0.911 | 1 | 0.994 | 0.981 | 0.881 | 0.988 | 0.994 | 0.98 | 0.957 | 1 | 0.997 | 0.9888 |
| 250 | 1 | 1 | 0.997 | 0.997 | 0.988 | 1 | 1 | 0.998 | 0.911 | 1 | 0.994 | 0.981 | 0.881 | 0.988 | 0.994 | 0.98 | 0.957 | 1 | 0.997 | 0.9888 |
| 251 | 1 | 1 | 0.997 | 0.997 | 0.988 | 1 | 1 | 0.998 | 0.911 | 1 | 0.994 | 0.981 | 0.881 | 0.988 | 0.992 | 0.978 | 0.957 | 1 | 0.997 | 0.9888 |
| 252 | 1 | 1 | 0.997 | 0.997 | 0.988 | 1 | 0.998 | 0.996 | 0.911 | 1 | 0.994 | 0.981 | 0.898 | 0.988 | 0.986 | 0.976 | 0.968 | 1 | 0.997 | 0.991 |
| 253 | 1 | 1 | 0.997 | 0.997 | 0.988 | 1 | 0.998 | 0.996 | 0.911 | 1 | 0.994 | 0.981 | 0.898 | 0.988 | 0.986 | 0.976 | 0.963 | 1 | 0.997 | 0.9899 |
| 254 | 1 | 1 | 0.997 | 0.997 | 0.988 | 1 | 0.998 | 0.996 | 0.911 | 1 | 0.994 | 0.981 | 0.898 | 0.988 | 0.986 | 0.976 | 0.963 | 1 | 0.997 | 0.9899 |
| 255 | 1 | 1 | 0.997 | 0.997 | 0.988 | 1 | 0.998 | 0.996 | 0.911 | 1 | 0.994 | 0.981 | 0.898 | 0.988 | 0.986 | 0.976 | 0.963 | 1 | 0.997 | 0.9899 |
| 256 | 1 | 1 | 0.997 | 0.997 | 0.988 | 1 | 0.998 | 0.996 | 0.911 | 1 | 0.994 | 0.981 | 0.898 | 0.988 | 0.986 | 0.976 | 0.963 | 1 | 0.997 | 0.9899 |
| 257 | 1 | 1 | 0.997 | 0.997 | 0.988 | 1 | 0.998 | 0.996 | 0.911 | 1 | 0.994 | 0.981 | 0.898 | 0.975 | 0.986 | 0.974 | 0.968 | 1 | 0.995 | 0.9899 |
| 258 | 1 | 1 | 0.997 | 0.997 | 0.988 | 1 | 0.998 | 0.996 | 0.911 | 1 | 0.994 | 0.981 | 0.898 | 0.988 | 0.983 | 0.974 | 0.973 | 1 | 0.997 | 0.9922 |
| 259 | 1 | 1 | 0.997 | 0.997 | 0.988 | 1 | 1 | 0.998 | 0.911 | 1 | 0.994 | 0.981 | 0.898 | 0.975 | 0.983 | 0.972 | 0.973 | 1 | 0.997 | 0.9922 |
| 260 | 1 | 1 | 0.997 | 0.997 | 0.988 | 1 | 1 | 0.998 | 0.911 | 1 | 0.994 | 0.981 | 0.898 | 0.975 | 0.983 | 0.972 | 0.973 | 1 | 0.997 | 0.9922 |
| 261 | 1 | 1 | 0.997 | 0.997 | 0.988 | 1 | 1 | 0.998 | 0.911 | 1 | 0.994 | 0.981 | 0.898 | 0.975 | 0.983 | 0.972 | 0.968 | 1 | 0.997 | 0.991 |
| 262 | 1 | 1 | 0.997 | 0.997 | 0.988 | 1 | 1 | 0.998 | 0.911 | 1 | 0.994 | 0.981 | 0.898 | 0.988 | 0.983 | 0.974 | 0.968 | 1 | 0.997 | 0.991 |
| 263 | 1 | 1 | 0.997 | 0.997 | 0.988 | 1 | 1 | 0.998 | 0.911 | 1 | 0.994 | 0.981 | 0.898 | 0.988 | 0.983 | 0.974 | 0.968 | 1 | 0.997 | 0.991 |
| 264 | 1 | 1 | 0.997 | 0.997 | 0.988 | 1 | 1 | 0.998 | 0.911 | 1 | 0.994 | 0.981 | 0.898 | 0.988 | 0.986 | 0.976 | 0.968 | 1 | 0.997 | 0.991 |
| 265 | 1 | 1 | 0.997 | 0.997 | 0.988 | 1 | 0.998 | 0.996 | 0.911 | 1 | 0.994 | 0.981 | 0.898 | 0.988 | 0.981 | 0.972 | 0.963 | 1 | 0.997 | 0.9899 |
| 266 | 1 | 1 | 0.997 | 0.997 | 0.988 | 1 | 0.998 | 0.996 | 0.911 | 1 | 0.994 | 0.981 | 0.898 | 0.988 | 0.981 | 0.972 | 0.963 | 1 | 0.997 | 0.9899 |
| 267 | 1 | 1 | 0.997 | 0.997 | 0.988 | 1 | 0.998 | 0.996 | 0.911 | 1 | 0.994 | 0.981 | 0.898 | 0.988 | 0.983 | 0.974 | 0.963 | 1 | 0.997 | 0.9899 |
| 268 | 1 | 1 | 0.997 | 0.997 | 0.988 | 1 | 1 | 0.998 | 0.911 | 1 | 0.994 | 0.981 | 0.898 | 0.988 | 0.986 | 0.976 | 0.963 | 1 | 0.995 | 0.9888 |
| 269 | 1 | 1 | 0.997 | 0.997 | 0.988 | 1 | 1 | 0.998 | 0.911 | 1 | 0.994 | 0.981 | 0.898 | 0.988 | 0.986 | 0.976 | 0.963 | 1 | 0.995 | 0.9888 |
| 270 | 1 | 1 | 0.997 | 0.997 | 0.988 | 1 | 1 | 0.998 | 0.911 | 1 | 0.994 | 0.981 | 0.898 | 0.988 | 0.986 | 0.976 | 0.963 | 1 | 0.995 | 0.9888 |
| 271 | 1 | 1 | 0.997 | 0.997 | 0.988 | 1 | 1 | 0.998 | 0.911 | 1 | 0.994 | 0.981 | 0.898 | 0.988 | 0.986 | 0.976 | 0.963 | 1 | 0.995 | 0.9888 |
| 272 | 1 | 1 | 1 | 1 | 0.988 | 1 | 1 | 0.998 | 0.911 | 1 | 0.994 | 0.981 | 0.915 | 0.988 | 0.983 | 0.976 | 0.957 | 1 | 0.995 | 0.9877 |
| 273 | 1 | 1 | 1 | 1 | 0.988 | 1 | 1 | 0.998 | 0.911 | 1 | 0.994 | 0.981 | 0.915 | 0.988 | 0.983 | 0.976 | 0.957 | 1 | 0.995 | 0.9877 |
| 274 | 1 | 1 | 1 | 1 | 0.988 | 1 | 1 | 0.998 | 0.911 | 1 | 0.994 | 0.981 | 0.915 | 0.988 | 0.983 | 0.976 | 0.957 | 1 | 0.995 | 0.9877 |
| 275 | 1 | 1 | 1 | 1 | 0.988 | 1 | 1 | 0.998 | 0.911 | 1 | 0.994 | 0.981 | 0.915 | 0.988 | 0.983 | 0.976 | 0.957 | 1 | 0.995 | 0.9877 |
| 276 | 1 | 1 | 1 | 1 | 0.988 | 1 | 1 | 0.998 | 0.911 | 1 | 0.994 | 0.981 | 0.915 | 0.988 | 0.983 | 0.976 | 0.973 | 1 | 0.995 | 0.991 |
| 277 | 1 | 1 | 1 | 1 | 0.988 | 1 | 1 | 0.998 | 0.911 | 1 | 0.994 | 0.981 | 0.915 | 0.988 | 0.983 | 0.976 | 0.973 | 1 | 0.995 | 0.991 |
| 278 | 1 | 1 | 1 | 1 | 0.988 | 1 | 1 | 0.998 | 0.911 | 1 | 0.994 | 0.981 | 0.915 | 0.988 | 0.983 | 0.976 | 0.973 | 1 | 0.995 | 0.991 |
| 279 | 1 | 1 | 1 | 1 | 0.988 | 1 | 1 | 0.998 | 0.911 | 1 | 0.994 | 0.981 | 0.915 | 0.988 | 0.983 | 0.976 | 0.973 | 1 | 0.995 | 0.991 |
| 280 | 1 | 1 | 1 | 1 | 0.988 | 1 | 1 | 0.998 | 0.911 | 1 | 0.994 | 0.981 | 0.915 | 0.988 | 0.983 | 0.976 | 0.968 | 1 | 0.995 | 0.9899 |
| 281 | 1 | 1 | 1 | 1 | 0.988 | 1 | 1 | 0.998 | 0.911 | 1 | 0.994 | 0.981 | 0.915 | 0.988 | 0.983 | 0.976 | 0.963 | 1 | 0.995 | 0.9888 |
| 282 | 1 | 1 | 1 | 1 | 0.988 | 1 | 1 | 0.998 | 0.911 | 1 | 0.994 | 0.981 | 0.915 | 0.988 | 0.983 | 0.976 | 0.963 | 1 | 0.995 | 0.9888 |
| 283 | 1 | 1 | 1 | 1 | 0.988 | 1 | 1 | 0.998 | 0.911 | 1 | 0.994 | 0.981 | 0.915 | 0.988 | 0.983 | 0.976 | 0.968 | 1 | 0.995 | 0.9899 |
| 284 | 1 | 1 | 1 | 1 | 0.988 | 1 | 1 | 0.998 | 0.911 | 1 | 0.994 | 0.981 | 0.915 | 0.988 | 0.983 | 0.976 | 0.973 | 1 | 0.995 | 0.991 |
| 285 | 1 | 1 | 1 | 1 | 0.988 | 1 | 1 | 0.998 | 0.911 | 1 | 0.994 | 0.981 | 0.915 | 0.988 | 0.983 | 0.976 | 0.957 | 1 | 0.997 | 0.9888 |
| 286 | 1 | 1 | 1 | 1 | 0.988 | 1 | 1 | 0.998 | 0.911 | 1 | 0.994 | 0.981 | 0.915 | 0.988 | 0.983 | 0.976 | 0.963 | 1 | 0.997 | 0.9899 |
| 287 | 1 | 1 | 1 | 1 | 0.988 | 1 | 1 | 0.998 | 0.911 | 1 | 0.994 | 0.981 | 0.915 | 0.988 | 0.983 | 0.976 | 0.963 | 1 | 0.997 | 0.9899 |
| 288 | 1 | 1 | 1 | 1 | 0.988 | 1 | 1 | 0.998 | 0.911 | 1 | 0.994 | 0.981 | 0.915 | 0.988 | 0.983 | 0.976 | 0.963 | 1 | 0.997 | 0.9899 |
| 289 | 1 | 1 | 1 | 1 | 0.988 | 1 | 1 | 0.998 | 0.911 | 1 | 0.994 | 0.981 | 0.915 | 0.988 | 0.983 | 0.976 | 0.957 | 1 | 0.997 | 0.9888 |
| 290 | 1 | 1 | 1 | 1 | 0.988 | 1 | 1 | 0.998 | 0.911 | 1 | 0.994 | 0.981 | 0.915 | 0.988 | 0.983 | 0.976 | 0.957 | 1 | 0.997 | 0.9888 |
| 291 | 1 | 1 | 1 | 1 | 0.988 | 1 | 1 | 0.998 | 0.911 | 1 | 0.994 | 0.981 | 0.915 | 0.988 | 0.983 | 0.976 | 0.957 | 1 | 0.997 | 0.9888 |
| 292 | 1 | 1 | 1 | 1 | 0.988 | 1 | 1 | 0.998 | 0.911 | 1 | 0.994 | 0.981 | 0.915 | 0.988 | 0.983 | 0.976 | 0.957 | 1 | 0.997 | 0.9888 |
| 293 | 1 | 1 | 1 | 1 | 0.988 | 1 | 1 | 0.998 | 0.911 | 1 | 0.994 | 0.981 | 0.915 | 0.988 | 0.983 | 0.976 | 0.957 | 1 | 0.997 | 0.9888 |
| 294 | 1 | 1 | 1 | 1 | 0.988 | 1 | 1 | 0.998 | 0.911 | 1 | 0.994 | 0.981 | 0.915 | 0.988 | 0.983 | 0.976 | 0.963 | 1 | 0.997 | 0.9899 |
| 295 | 1 | 1 | 1 | 1 | 0.988 | 1 | 1 | 0.998 | 0.911 | 1 | 0.994 | 0.981 | 0.915 | 0.988 | 0.983 | 0.976 | 0.957 | 1 | 0.997 | 0.9888 |
| 296 | 1 | 1 | 1 | 1 | 0.988 | 1 | 1 | 0.998 | 0.911 | 1 | 0.994 | 0.981 | 0.915 | 0.988 | 0.983 | 0.976 | 0.957 | 1 | 0.997 | 0.9888 |
| 297 | 1 | 1 | 1 | 1 | 0.988 | 1 | 1 | 0.998 | 0.911 | 1 | 0.994 | 0.981 | 0.915 | 0.988 | 0.983 | 0.976 | 0.957 | 1 | 0.997 | 0.9888 |
| 298 | 1 | 1 | 1 | 1 | 0.988 | 1 | 1 | 0.998 | 0.911 | 1 | 0.994 | 0.981 | 0.915 | 0.988 | 0.983 | 0.976 | 0.957 | 1 | 0.997 | 0.9888 |
| 299 | 1 | 1 | 1 | 1 | 0.988 | 1 | 1 | 0.998 | 0.911 | 1 | 0.994 | 0.981 | 0.898 | 0.988 | 0.983 | 0.974 | 0.957 | 1 | 0.997 | 0.9888 |
| 300 | 1 | 1 | 1 | 1 | 0.988 | 1 | 1 | 0.998 | 0.911 | 1 | 0.994 | 0.981 | 0.898 | 0.988 | 0.983 | 0.974 | 0.957 | 1 | 0.997 | 0.9888 |
| 301 | 1 | 1 | 1 | 1 | 0.988 | 1 | 1 | 0.998 | 0.911 | 1 | 0.994 | 0.981 | 0.898 | 0.988 | 0.983 | 0.974 | 0.957 | 1 | 0.997 | 0.9888 |
| 302 | 1 | 1 | 1 | 1 | 0.988 | 1 | 1 | 0.998 | 0.911 | 1 | 0.994 | 0.981 | 0.898 | 0.988 | 0.983 | 0.974 | 0.957 | 1 | 0.997 | 0.9888 |
| 303 | 1 | 1 | 1 | 1 | 0.988 | 1 | 1 | 0.998 | 0.911 | 1 | 0.994 | 0.981 | 0.898 | 0.988 | 0.989 | 0.978 | 0.957 | 1 | 0.997 | 0.9888 |
| 304 | 1 | 1 | 1 | 1 | 0.988 | 1 | 1 | 0.998 | 0.911 | 1 | 0.994 | 0.981 | 0.898 | 0.988 | 0.989 | 0.978 | 0.957 | 1 | 0.997 | 0.9888 |
| 305 | 1 | 1 | 1 | 1 | 0.988 | 1 | 1 | 0.998 | 0.911 | 1 | 0.994 | 0.981 | 0.898 | 0.988 | 0.989 | 0.978 | 0.957 | 1 | 0.997 | 0.9888 |
| 306 | 1 | 1 | 1 | 1 | 0.988 | 1 | 1 | 0.998 | 0.911 | 1 | 0.994 | 0.981 | 0.898 | 0.988 | 0.989 | 0.978 | 0.957 | 1 | 0.997 | 0.9888 |
| 307 | 1 | 1 | 1 | 1 | 0.988 | 1 | 1 | 0.998 | 0.911 | 1 | 0.994 | 0.981 | 0.898 | 0.988 | 0.989 | 0.978 | 0.957 | 1 | 0.997 | 0.9888 |
| 308 | 1 | 1 | 1 | 1 | 0.988 | 1 | 1 | 0.998 | 0.911 | 1 | 0.994 | 0.981 | 0.898 | 0.988 | 0.989 | 0.978 | 0.957 | 1 | 0.997 | 0.9888 |
| 309 | 1 | 1 | 1 | 1 | 0.988 | 1 | 1 | 0.998 | 0.911 | 1 | 0.994 | 0.981 | 0.898 | 0.988 | 0.989 | 0.978 | 0.957 | 1 | 0.997 | 0.9888 |
| 310 | 1 | 1 | 1 | 1 | 0.988 | 1 | 1 | 0.998 | 0.911 | 1 | 0.994 | 0.981 | 0.898 | 0.988 | 0.989 | 0.978 | 0.957 | 1 | 0.997 | 0.9888 |
| 311 | 1 | 1 | 1 | 1 | 0.988 | 1 | 1 | 0.998 | 0.911 | 1 | 0.994 | 0.981 | 0.898 | 0.988 | 0.986 | 0.976 | 0.957 | 1 | 0.997 | 0.9888 |
| 312 | 1 | 1 | 1 | 1 | 0.988 | 1 | 1 | 0.998 | 0.911 | 1 | 0.994 | 0.981 | 0.898 | 0.988 | 0.986 | 0.976 | 0.957 | 1 | 0.997 | 0.9888 |
| 313 | 1 | 1 | 1 | 1 | 0.988 | 1 | 1 | 0.998 | 0.911 | 1 | 0.994 | 0.981 | 0.898 | 0.988 | 0.986 | 0.976 | 0.957 | 1 | 0.997 | 0.9888 |
| 314 | 1 | 1 | 1 | 1 | 0.988 | 1 | 1 | 0.998 | 0.911 | 1 | 0.994 | 0.981 | 0.898 | 0.988 | 0.986 | 0.976 | 0.957 | 1 | 0.997 | 0.9888 |
| 315 | 1 | 1 | 1 | 1 | 0.988 | 1 | 1 | 0.998 | 0.911 | 1 | 0.994 | 0.981 | 0.898 | 0.988 | 0.986 | 0.976 | 0.957 | 1 | 0.997 | 0.9888 |
| 316 | 1 | 1 | 1 | 1 | 0.988 | 1 | 1 | 0.998 | 0.911 | 1 | 0.994 | 0.981 | 0.898 | 0.988 | 0.986 | 0.976 | 0.957 | 1 | 0.997 | 0.9888 |
| 317 | 1 | 1 | 1 | 1 | 0.988 | 1 | 1 | 0.998 | 0.911 | 1 | 0.994 | 0.981 | 0.898 | 1 | 0.986 | 0.978 | 0.957 | 1 | 0.997 | 0.9888 |
| 318 | 1 | 1 | 1 | 1 | 0.988 | 1 | 1 | 0.998 | 0.911 | 1 | 0.994 | 0.981 | 0.898 | 1 | 0.986 | 0.978 | 0.957 | 1 | 0.997 | 0.9888 |
| 319 | 1 | 1 | 1 | 1 | 0.988 | 1 | 1 | 0.998 | 0.911 | 1 | 0.994 | 0.981 | 0.898 | 1 | 0.986 | 0.978 | 0.957 | 1 | 0.997 | 0.9888 |
| 320 | 1 | 1 | 1 | 1 | 0.988 | 1 | 1 | 0.998 | 0.911 | 1 | 0.994 | 0.981 | 0.898 | 1 | 0.986 | 0.978 | 0.957 | 1 | 0.997 | 0.9888 |
| 321 | 1 | 1 | 1 | 1 | 0.988 | 1 | 1 | 0.998 | 0.911 | 1 | 0.994 | 0.981 | 0.898 | 1 | 0.986 | 0.978 | 0.957 | 1 | 0.997 | 0.9888 |
| 322 | 1 | 1 | 1 | 1 | 0.988 | 1 | 1 | 0.998 | 0.911 | 1 | 0.994 | 0.981 | 0.898 | 1 | 0.986 | 0.978 | 0.957 | 1 | 0.997 | 0.9888 |
| 323 | 1 | 1 | 1 | 1 | 0.988 | 1 | 1 | 0.998 | 0.911 | 1 | 0.994 | 0.981 | 0.898 | 1 | 0.986 | 0.978 | 0.957 | 1 | 0.997 | 0.9888 |
| 324 | 1 | 1 | 1 | 1 | 0.988 | 1 | 1 | 0.998 | 0.899 | 1 | 0.994 | 0.979 | 0.898 | 1 | 0.986 | 0.978 | 0.957 | 1 | 0.997 | 0.9888 |
| 325 | 1 | 1 | 1 | 1 | 0.988 | 1 | 1 | 0.998 | 0.899 | 1 | 0.994 | 0.979 | 0.898 | 1 | 0.986 | 0.978 | 0.957 | 1 | 0.997 | 0.9888 |
| 326 | 1 | 1 | 1 | 1 | 0.988 | 1 | 1 | 0.998 | 0.911 | 1 | 0.994 | 0.981 | 0.898 | 1 | 0.986 | 0.978 | 0.957 | 1 | 0.997 | 0.9888 |
| 327 | 1 | 1 | 1 | 1 | 0.988 | 1 | 1 | 0.998 | 0.911 | 1 | 0.994 | 0.981 | 0.898 | 1 | 0.986 | 0.978 | 0.957 | 1 | 0.997 | 0.9888 |
| 328 | 1 | 1 | 1 | 1 | 0.988 | 1 | 1 | 0.998 | 0.911 | 1 | 0.994 | 0.981 | 0.898 | 1 | 0.986 | 0.978 | 0.957 | 1 | 0.997 | 0.9888 |
| 329 | 1 | 1 | 1 | 1 | 0.988 | 1 | 1 | 0.998 | 0.911 | 1 | 0.994 | 0.981 | 0.898 | 1 | 0.983 | 0.976 | 0.957 | 1 | 0.998 | 0.9899 |
| 330 | 1 | 1 | 1 | 1 | 0.988 | 1 | 1 | 0.998 | 0.911 | 1 | 0.994 | 0.981 | 0.898 | 1 | 0.983 | 0.976 | 0.957 | 1 | 0.998 | 0.9899 |
| 331 | 1 | 1 | 1 | 1 | 0.988 | 1 | 1 | 0.998 | 0.911 | 1 | 0.994 | 0.981 | 0.898 | 1 | 0.983 | 0.976 | 0.957 | 1 | 0.998 | 0.9899 |
| 332 | 1 | 1 | 1 | 1 | 0.988 | 1 | 1 | 0.998 | 0.911 | 1 | 0.994 | 0.981 | 0.898 | 1 | 0.983 | 0.976 | 0.957 | 1 | 0.998 | 0.9899 |
| 333 | 1 | 1 | 1 | 1 | 0.988 | 1 | 1 | 0.998 | 0.911 | 1 | 0.994 | 0.981 | 0.898 | 1 | 0.986 | 0.978 | 0.957 | 1 | 0.997 | 0.9888 |
| 334 | 1 | 1 | 1 | 1 | 0.988 | 1 | 1 | 0.998 | 0.911 | 1 | 0.994 | 0.981 | 0.898 | 1 | 0.986 | 0.978 | 0.957 | 1 | 0.997 | 0.9888 |
| 335 | 1 | 1 | 1 | 1 | 0.988 | 1 | 1 | 0.998 | 0.911 | 1 | 0.994 | 0.981 | 0.898 | 1 | 0.986 | 0.978 | 0.957 | 1 | 0.997 | 0.9888 |
| 336 | 1 | 1 | 1 | 1 | 0.988 | 1 | 1 | 0.998 | 0.911 | 1 | 0.994 | 0.981 | 0.898 | 1 | 0.986 | 0.978 | 0.957 | 1 | 0.997 | 0.9888 |
| 337 | 1 | 1 | 1 | 1 | 0.988 | 1 | 1 | 0.998 | 0.899 | 1 | 0.994 | 0.979 | 0.898 | 1 | 0.986 | 0.978 | 0.957 | 1 | 0.997 | 0.9888 |
| 338 | 1 | 1 | 1 | 1 | 0.988 | 1 | 1 | 0.998 | 0.911 | 1 | 0.994 | 0.981 | 0.898 | 1 | 0.986 | 0.978 | 0.957 | 1 | 0.997 | 0.9888 |
| 339 | 1 | 1 | 1 | 1 | 0.988 | 1 | 1 | 0.998 | 0.911 | 1 | 0.994 | 0.981 | 0.898 | 1 | 0.986 | 0.978 | 0.957 | 1 | 0.997 | 0.9888 |
| 340 | 1 | 1 | 1 | 1 | 0.988 | 1 | 1 | 0.998 | 0.911 | 1 | 0.994 | 0.981 | 0.898 | 1 | 0.986 | 0.978 | 0.957 | 1 | 0.997 | 0.9888 |
| 341 | 1 | 1 | 1 | 1 | 0.988 | 1 | 1 | 0.998 | 0.911 | 1 | 0.994 | 0.981 | 0.898 | 1 | 0.986 | 0.978 | 0.957 | 1 | 0.997 | 0.9888 |
| 342 | 1 | 1 | 1 | 1 | 0.988 | 1 | 1 | 0.998 | 0.911 | 1 | 0.994 | 0.981 | 0.898 | 1 | 0.981 | 0.974 | 0.984 | 1 | 0.995 | 0.9933 |
| 343 | 1 | 1 | 1 | 1 | 0.988 | 1 | 1 | 0.998 | 0.911 | 1 | 0.994 | 0.981 | 0.898 | 1 | 0.981 | 0.974 | 0.984 | 1 | 0.995 | 0.9933 |
| 344 | 1 | 1 | 1 | 1 | 0.988 | 1 | 1 | 0.998 | 0.911 | 1 | 0.994 | 0.981 | 0.898 | 1 | 0.981 | 0.974 | 0.984 | 1 | 0.994 | 0.9922 |
| 345 | 1 | 1 | 1 | 1 | 0.988 | 1 | 1 | 0.998 | 0.911 | 1 | 0.994 | 0.981 | 0.898 | 1 | 0.981 | 0.974 | 0.984 | 1 | 0.994 | 0.9922 |
| 346 | 1 | 1 | 1 | 1 | 0.988 | 1 | 1 | 0.998 | 0.911 | 1 | 0.994 | 0.981 | 0.915 | 1 | 0.981 | 0.976 | 0.984 | 1 | 0.994 | 0.9922 |
| 347 | 1 | 1 | 1 | 1 | 0.988 | 1 | 1 | 0.998 | 0.911 | 1 | 0.994 | 0.981 | 0.915 | 1 | 0.981 | 0.976 | 0.984 | 1 | 0.995 | 0.9933 |
| 348 | 1 | 1 | 1 | 1 | 0.988 | 1 | 1 | 0.998 | 0.911 | 1 | 0.994 | 0.981 | 0.898 | 1 | 0.981 | 0.974 | 0.984 | 1 | 0.994 | 0.9922 |
| 349 | 1 | 1 | 1 | 1 | 0.988 | 1 | 1 | 0.998 | 0.911 | 1 | 0.994 | 0.981 | 0.898 | 1 | 0.981 | 0.974 | 0.984 | 1 | 0.994 | 0.9922 |
| 350 | 1 | 1 | 1 | 1 | 0.988 | 1 | 1 | 0.998 | 0.911 | 1 | 0.994 | 0.981 | 0.898 | 1 | 0.981 | 0.974 | 0.984 | 1 | 0.994 | 0.9922 |
| 351 | 1 | 1 | 1 | 1 | 0.988 | 1 | 1 | 0.998 | 0.911 | 1 | 0.994 | 0.981 | 0.898 | 1 | 0.981 | 0.974 | 0.984 | 1 | 0.995 | 0.9933 |
| 352 | 1 | 1 | 1 | 1 | 0.988 | 1 | 1 | 0.998 | 0.911 | 1 | 0.994 | 0.981 | 0.898 | 1 | 0.981 | 0.974 | 0.984 | 1 | 0.995 | 0.9933 |
| 353 | 1 | 1 | 1 | 1 | 0.988 | 1 | 1 | 0.998 | 0.911 | 1 | 0.994 | 0.981 | 0.898 | 1 | 0.981 | 0.974 | 0.984 | 1 | 0.995 | 0.9933 |
| 354 | 1 | 1 | 1 | 1 | 0.988 | 1 | 1 | 0.998 | 0.911 | 1 | 0.994 | 0.981 | 0.898 | 1 | 0.981 | 0.974 | 0.984 | 1 | 0.995 | 0.9933 |
| 355 | 1 | 1 | 1 | 1 | 0.988 | 1 | 1 | 0.998 | 0.911 | 1 | 0.994 | 0.981 | 0.898 | 1 | 0.981 | 0.974 | 0.984 | 1 | 0.995 | 0.9933 |
| 356 | 1 | 1 | 1 | 1 | 0.988 | 1 | 1 | 0.998 | 0.911 | 1 | 0.994 | 0.981 | 0.898 | 1 | 0.981 | 0.974 | 0.984 | 1 | 0.995 | 0.9933 |
| 357 | 1 | 1 | 1 | 1 | 0.988 | 1 | 1 | 0.998 | 0.911 | 1 | 0.994 | 0.981 | 0.898 | 1 | 0.981 | 0.974 | 0.984 | 1 | 0.995 | 0.9933 |
| 358 | 1 | 1 | 1 | 1 | 0.988 | 1 | 1 | 0.998 | 0.911 | 1 | 0.994 | 0.981 | 0.898 | 1 | 0.981 | 0.974 | 0.979 | 1 | 0.995 | 0.9922 |
| 359 | 1 | 1 | 1 | 1 | 0.988 | 1 | 1 | 0.998 | 0.911 | 1 | 0.994 | 0.981 | 0.898 | 0.988 | 0.981 | 0.972 | 0.979 | 1 | 0.995 | 0.9922 |
| 360 | 1 | 1 | 1 | 1 | 0.988 | 1 | 1 | 0.998 | 0.911 | 1 | 0.994 | 0.981 | 0.898 | 0.988 | 0.981 | 0.972 | 0.979 | 1 | 0.995 | 0.9922 |
| 361 | 1 | 1 | 1 | 1 | 0.988 | 1 | 1 | 0.998 | 0.911 | 1 | 0.994 | 0.981 | 0.898 | 1 | 0.981 | 0.974 | 0.973 | 1 | 0.995 | 0.991 |
| 362 | 1 | 1 | 1 | 1 | 0.988 | 1 | 1 | 0.998 | 0.911 | 1 | 0.994 | 0.981 | 0.898 | 1 | 0.981 | 0.974 | 0.973 | 1 | 0.995 | 0.991 |
| 363 | 1 | 1 | 1 | 1 | 0.988 | 1 | 1 | 0.998 | 0.911 | 1 | 0.994 | 0.981 | 0.898 | 1 | 0.981 | 0.974 | 0.973 | 1 | 0.995 | 0.991 |
| 364 | 1 | 1 | 1 | 1 | 0.988 | 1 | 1 | 0.998 | 0.911 | 1 | 0.994 | 0.981 | 0.898 | 0.988 | 0.981 | 0.972 | 0.979 | 1 | 0.995 | 0.9922 |
| 365 | 1 | 1 | 1 | 1 | 0.988 | 1 | 1 | 0.998 | 0.911 | 1 | 0.994 | 0.981 | 0.898 | 0.988 | 0.981 | 0.972 | 0.984 | 1 | 0.995 | 0.9933 |
| 366 | 1 | 1 | 1 | 1 | 0.988 | 1 | 1 | 0.998 | 0.911 | 1 | 0.994 | 0.981 | 0.898 | 0.988 | 0.983 | 0.974 | 0.979 | 1 | 0.995 | 0.9922 |
| 367 | 1 | 1 | 1 | 1 | 0.988 | 1 | 1 | 0.998 | 0.911 | 1 | 0.994 | 0.981 | 0.898 | 0.988 | 0.983 | 0.974 | 0.979 | 1 | 0.995 | 0.9922 |
| 368 | 1 | 1 | 1 | 1 | 0.988 | 1 | 1 | 0.998 | 0.911 | 1 | 0.994 | 0.981 | 0.898 | 0.988 | 0.983 | 0.974 | 0.984 | 1 | 0.995 | 0.9933 |
| 369 | 1 | 1 | 1 | 1 | 0.988 | 1 | 1 | 0.998 | 0.911 | 1 | 0.994 | 0.981 | 0.898 | 1 | 0.983 | 0.976 | 0.984 | 1 | 0.995 | 0.9933 |
| 370 | 1 | 1 | 1 | 1 | 0.988 | 1 | 1 | 0.998 | 0.911 | 1 | 0.994 | 0.981 | 0.898 | 1 | 0.983 | 0.976 | 0.979 | 1 | 0.995 | 0.9922 |
| 371 | 1 | 1 | 1 | 1 | 0.988 | 1 | 1 | 0.998 | 0.911 | 1 | 0.994 | 0.981 | 0.898 | 1 | 0.983 | 0.976 | 0.968 | 1 | 0.995 | 0.9899 |
| 372 | 0.962 | 1 | 1 | 0.997 | 0.988 | 1 | 1 | 0.998 | 0.911 | 1 | 0.994 | 0.981 | 0.898 | 0.988 | 0.983 | 0.974 | 0.968 | 1 | 0.997 | 0.991 |
| 373 | 0.962 | 1 | 1 | 0.997 | 0.988 | 1 | 1 | 0.998 | 0.911 | 1 | 0.994 | 0.981 | 0.898 | 0.988 | 0.983 | 0.974 | 0.968 | 1 | 0.997 | 0.991 |
| 374 | 0.962 | 1 | 1 | 0.997 | 0.988 | 1 | 1 | 0.998 | 0.911 | 1 | 0.994 | 0.981 | 0.898 | 0.988 | 0.981 | 0.972 | 0.984 | 1 | 0.997 | 0.9944 |
| 375 | 0.962 | 1 | 1 | 0.997 | 0.988 | 1 | 1 | 0.998 | 0.911 | 1 | 0.994 | 0.981 | 0.898 | 0.988 | 0.981 | 0.972 | 0.984 | 1 | 0.995 | 0.9933 |
| 376 | 0.962 | 1 | 1 | 0.997 | 0.988 | 1 | 1 | 0.998 | 0.924 | 1 | 0.994 | 0.983 | 0.898 | 0.988 | 0.981 | 0.972 | 0.984 | 1 | 0.995 | 0.9933 |
| 377 | 0.962 | 1 | 1 | 0.997 | 0.988 | 1 | 1 | 0.998 | 0.924 | 1 | 0.994 | 0.983 | 0.898 | 0.988 | 0.981 | 0.972 | 0.984 | 1 | 0.995 | 0.9933 |
| 378 | 0.962 | 1 | 1 | 0.997 | 0.988 | 1 | 1 | 0.998 | 0.924 | 1 | 0.994 | 0.983 | 0.915 | 0.988 | 0.981 | 0.974 | 0.984 | 1 | 0.995 | 0.9933 |
| 379 | 0.962 | 1 | 1 | 0.997 | 0.988 | 1 | 1 | 0.998 | 0.924 | 1 | 0.994 | 0.983 | 0.915 | 0.988 | 0.981 | 0.974 | 0.984 | 1 | 0.995 | 0.9933 |
| 380 | 0.962 | 1 | 1 | 0.997 | 0.988 | 1 | 1 | 0.998 | 0.924 | 1 | 0.994 | 0.983 | 0.915 | 0.988 | 0.981 | 0.974 | 0.984 | 1 | 0.995 | 0.9933 |
| 381 | 0.962 | 1 | 1 | 0.997 | 0.988 | 1 | 1 | 0.998 | 0.924 | 1 | 0.994 | 0.983 | 0.915 | 0.988 | 0.981 | 0.974 | 0.984 | 1 | 0.995 | 0.9933 |
| 382 | 0.962 | 1 | 1 | 0.997 | 0.988 | 1 | 1 | 0.998 | 0.924 | 1 | 0.994 | 0.983 | 0.915 | 0.988 | 0.981 | 0.974 | 0.984 | 1 | 0.995 | 0.9933 |
| 383 | 0.962 | 1 | 1 | 0.997 | 0.988 | 1 | 1 | 0.998 | 0.924 | 1 | 0.994 | 0.983 | 0.915 | 0.988 | 0.981 | 0.974 | 0.984 | 1 | 0.995 | 0.9933 |
| 384 | 0.962 | 1 | 1 | 0.997 | 0.988 | 1 | 1 | 0.998 | 0.924 | 1 | 0.994 | 0.983 | 0.915 | 0.988 | 0.978 | 0.972 | 0.984 | 1 | 0.995 | 0.9933 |
| 385 | 1 | 1 | 1 | 1 | 0.988 | 1 | 1 | 0.998 | 0.924 | 1 | 0.991 | 0.981 | 0.915 | 0.988 | 0.978 | 0.972 | 0.984 | 1 | 0.995 | 0.9933 |
| 386 | 1 | 1 | 1 | 1 | 0.988 | 1 | 1 | 0.998 | 0.924 | 1 | 0.991 | 0.981 | 0.915 | 0.988 | 0.978 | 0.972 | 0.984 | 1 | 0.994 | 0.9922 |
| 387 | 1 | 1 | 1 | 1 | 0.988 | 1 | 1 | 0.998 | 0.924 | 1 | 0.994 | 0.983 | 0.915 | 0.988 | 0.978 | 0.972 | 0.984 | 1 | 0.995 | 0.9933 |
| 388 | 1 | 1 | 1 | 1 | 0.988 | 1 | 1 | 0.998 | 0.924 | 1 | 0.994 | 0.983 | 0.915 | 0.988 | 0.978 | 0.972 | 0.984 | 1 | 0.995 | 0.9933 |
| 389 | 1 | 1 | 1 | 1 | 0.988 | 1 | 1 | 0.998 | 0.924 | 1 | 0.994 | 0.983 | 0.915 | 0.988 | 0.981 | 0.974 | 0.984 | 1 | 0.995 | 0.9933 |
| 390 | 1 | 1 | 1 | 1 | 0.988 | 1 | 1 | 0.998 | 0.924 | 1 | 0.994 | 0.983 | 0.915 | 0.988 | 0.981 | 0.974 | 0.984 | 1 | 0.995 | 0.9933 |
| 391 | 1 | 1 | 1 | 1 | 0.988 | 1 | 1 | 0.998 | 0.924 | 1 | 0.991 | 0.981 | 0.915 | 0.988 | 0.981 | 0.974 | 0.984 | 1 | 0.995 | 0.9933 |
| 392 | 1 | 1 | 1 | 1 | 0.988 | 1 | 1 | 0.998 | 0.924 | 1 | 0.991 | 0.981 | 0.915 | 0.988 | 0.981 | 0.974 | 0.984 | 1 | 0.995 | 0.9933 |
| 393 | 1 | 1 | 1 | 1 | 0.988 | 1 | 1 | 0.998 | 0.924 | 1 | 0.994 | 0.983 | 0.915 | 0.988 | 0.981 | 0.974 | 0.984 | 1 | 0.995 | 0.9933 |
| 394 | 0.962 | 1 | 1 | 0.997 | 0.988 | 1 | 1 | 0.998 | 0.924 | 1 | 0.991 | 0.981 | 0.915 | 0.988 | 0.981 | 0.974 | 0.968 | 1 | 0.995 | 0.9899 |
| 395 | 0.962 | 1 | 1 | 0.997 | 0.988 | 1 | 1 | 0.998 | 0.924 | 1 | 0.991 | 0.981 | 0.915 | 0.988 | 0.981 | 0.974 | 0.979 | 1 | 0.995 | 0.9922 |
| 396 | 0.962 | 1 | 1 | 0.997 | 0.988 | 1 | 1 | 0.998 | 0.924 | 1 | 0.991 | 0.981 | 0.915 | 0.988 | 0.981 | 0.974 | 0.984 | 1 | 0.995 | 0.9933 |
| 397 | 0.962 | 1 | 1 | 0.997 | 0.988 | 1 | 1 | 0.998 | 0.924 | 1 | 0.991 | 0.981 | 0.898 | 0.988 | 0.981 | 0.972 | 0.973 | 1 | 0.995 | 0.991 |
| 398 | 0.962 | 1 | 1 | 0.997 | 0.988 | 1 | 1 | 0.998 | 0.924 | 1 | 0.994 | 0.983 | 0.898 | 0.988 | 0.981 | 0.972 | 0.979 | 1 | 0.995 | 0.9922 |
| 399 | 0.962 | 1 | 1 | 0.997 | 0.988 | 1 | 1 | 0.998 | 0.924 | 1 | 0.994 | 0.983 | 0.898 | 0.988 | 0.981 | 0.972 | 0.973 | 1 | 0.995 | 0.991 |
| 400 | 0.962 | 1 | 1 | 0.997 | 0.988 | 1 | 1 | 0.998 | 0.924 | 1 | 0.994 | 0.983 | 0.898 | 0.988 | 0.981 | 0.972 | 0.973 | 1 | 0.995 | 0.991 |
| 401 | 0.962 | 1 | 1 | 0.997 | 0.988 | 1 | 1 | 0.998 | 0.924 | 1 | 0.994 | 0.983 | 0.898 | 0.988 | 0.981 | 0.972 | 0.973 | 1 | 0.995 | 0.991 |
| 402 | 0.962 | 1 | 1 | 0.997 | 0.988 | 1 | 1 | 0.998 | 0.924 | 1 | 0.994 | 0.983 | 0.898 | 0.988 | 0.981 | 0.972 | 0.973 | 1 | 0.995 | 0.991 |
| 403 | 0.962 | 1 | 1 | 0.997 | 0.988 | 1 | 1 | 0.998 | 0.924 | 1 | 0.994 | 0.983 | 0.898 | 0.988 | 0.981 | 0.972 | 0.973 | 1 | 0.995 | 0.991 |
| 404 | 0.962 | 1 | 1 | 0.997 | 0.988 | 1 | 1 | 0.998 | 0.924 | 1 | 0.994 | 0.983 | 0.898 | 0.988 | 0.981 | 0.972 | 0.973 | 1 | 0.995 | 0.991 |
| 405 | 0.962 | 1 | 1 | 0.997 | 0.988 | 1 | 1 | 0.998 | 0.924 | 1 | 0.994 | 0.983 | 0.898 | 0.988 | 0.981 | 0.972 | 0.973 | 1 | 0.995 | 0.991 |
| 406 | 0.962 | 1 | 1 | 0.997 | 0.988 | 1 | 1 | 0.998 | 0.924 | 1 | 0.991 | 0.981 | 0.898 | 0.988 | 0.981 | 0.972 | 0.979 | 1 | 0.995 | 0.9922 |
| 407 | 0.962 | 1 | 1 | 0.997 | 0.988 | 1 | 1 | 0.998 | 0.924 | 1 | 0.991 | 0.981 | 0.898 | 0.988 | 0.981 | 0.972 | 0.979 | 1 | 0.995 | 0.9922 |
| 408 | 0.962 | 1 | 1 | 0.997 | 0.988 | 1 | 1 | 0.998 | 0.924 | 1 | 0.991 | 0.981 | 0.898 | 0.988 | 0.981 | 0.972 | 0.979 | 1 | 0.995 | 0.9922 |
| 409 | 0.962 | 1 | 1 | 0.997 | 0.988 | 1 | 1 | 0.998 | 0.924 | 1 | 0.991 | 0.981 | 0.898 | 0.988 | 0.981 | 0.972 | 0.968 | 1 | 0.995 | 0.9899 |
| 410 | 0.962 | 1 | 1 | 0.997 | 0.988 | 1 | 1 | 0.998 | 0.924 | 1 | 0.991 | 0.981 | 0.898 | 0.988 | 0.978 | 0.97 | 0.968 | 1 | 0.997 | 0.991 |
| 411 | 0.962 | 1 | 1 | 0.997 | 0.988 | 1 | 1 | 0.998 | 0.924 | 1 | 0.991 | 0.981 | 0.898 | 0.988 | 0.981 | 0.972 | 0.968 | 1 | 0.995 | 0.9899 |
| 412 | 0.962 | 1 | 1 | 0.997 | 0.988 | 1 | 1 | 0.998 | 0.924 | 1 | 0.991 | 0.981 | 0.898 | 0.988 | 0.981 | 0.972 | 0.968 | 1 | 0.997 | 0.991 |
| 413 | 0.962 | 1 | 1 | 0.997 | 0.988 | 1 | 1 | 0.998 | 0.924 | 1 | 0.994 | 0.983 | 0.898 | 0.988 | 0.981 | 0.972 | 0.973 | 1 | 0.997 | 0.9922 |
| 414 | 0.962 | 1 | 1 | 0.997 | 0.988 | 1 | 1 | 0.998 | 0.924 | 1 | 0.994 | 0.983 | 0.898 | 0.988 | 0.981 | 0.972 | 0.963 | 1 | 0.997 | 0.9899 |
| 415 | 0.962 | 1 | 1 | 0.997 | 0.988 | 1 | 1 | 0.998 | 0.924 | 1 | 0.994 | 0.983 | 0.898 | 0.988 | 0.981 | 0.972 | 0.963 | 1 | 0.995 | 0.9888 |
| 416 | 0.962 | 1 | 1 | 0.997 | 0.988 | 1 | 1 | 0.998 | 0.924 | 1 | 0.994 | 0.983 | 0.898 | 0.988 | 0.981 | 0.972 | 0.963 | 1 | 0.997 | 0.9899 |
| 417 | 0.962 | 1 | 1 | 0.997 | 0.988 | 1 | 1 | 0.998 | 0.924 | 1 | 0.994 | 0.983 | 0.898 | 0.988 | 0.981 | 0.972 | 0.963 | 1 | 0.997 | 0.9899 |
| 418 | 0.962 | 1 | 1 | 0.997 | 0.988 | 1 | 1 | 0.998 | 0.924 | 1 | 0.994 | 0.983 | 0.898 | 0.988 | 0.981 | 0.972 | 0.963 | 1 | 0.997 | 0.9899 |
| 419 | 0.962 | 1 | 1 | 0.997 | 0.988 | 1 | 1 | 0.998 | 0.924 | 1 | 0.994 | 0.983 | 0.898 | 0.988 | 0.981 | 0.972 | 0.973 | 1 | 0.995 | 0.991 |
| 420 | 0.962 | 1 | 1 | 0.997 | 0.988 | 1 | 1 | 0.998 | 0.924 | 1 | 0.994 | 0.983 | 0.898 | 0.988 | 0.981 | 0.972 | 0.973 | 1 | 0.995 | 0.991 |
| 421 | 0.962 | 1 | 1 | 0.997 | 0.988 | 1 | 1 | 0.998 | 0.924 | 1 | 0.994 | 0.983 | 0.898 | 0.988 | 0.981 | 0.972 | 0.968 | 1 | 0.997 | 0.991 |
| 422 | 0.962 | 1 | 1 | 0.997 | 0.988 | 1 | 1 | 0.998 | 0.924 | 1 | 0.994 | 0.983 | 0.898 | 0.988 | 0.981 | 0.972 | 0.968 | 1 | 0.997 | 0.991 |
| 423 | 0.962 | 1 | 1 | 0.997 | 0.988 | 1 | 1 | 0.998 | 0.924 | 1 | 0.994 | 0.983 | 0.898 | 0.988 | 0.981 | 0.972 | 0.968 | 1 | 0.995 | 0.9899 |
| 424 | 0.962 | 1 | 1 | 0.997 | 0.988 | 1 | 1 | 0.998 | 0.924 | 1 | 0.994 | 0.983 | 0.898 | 0.988 | 0.981 | 0.972 | 0.968 | 1 | 0.995 | 0.9899 |
| 425 | 0.962 | 1 | 1 | 0.997 | 0.988 | 1 | 1 | 0.998 | 0.924 | 1 | 0.994 | 0.983 | 0.898 | 0.988 | 0.981 | 0.972 | 0.968 | 1 | 0.997 | 0.991 |
| 426 | 0.962 | 1 | 1 | 0.997 | 0.988 | 1 | 1 | 0.998 | 0.924 | 1 | 0.994 | 0.983 | 0.898 | 0.988 | 0.981 | 0.972 | 0.968 | 1 | 0.997 | 0.991 |
| 427 | 0.962 | 1 | 1 | 0.997 | 0.988 | 1 | 1 | 0.998 | 0.924 | 1 | 0.994 | 0.983 | 0.898 | 0.988 | 0.978 | 0.97 | 0.968 | 1 | 0.997 | 0.991 |
| 428 | 0.962 | 1 | 1 | 0.997 | 0.988 | 1 | 1 | 0.998 | 0.924 | 1 | 0.994 | 0.983 | 0.898 | 0.988 | 0.981 | 0.972 | 0.968 | 1 | 0.997 | 0.991 |
| 429 | 0.962 | 1 | 1 | 0.997 | 0.988 | 1 | 1 | 0.998 | 0.924 | 1 | 0.994 | 0.983 | 0.898 | 0.988 | 0.981 | 0.972 | 0.968 | 1 | 0.997 | 0.991 |
| 430 | 0.962 | 1 | 1 | 0.997 | 0.988 | 1 | 1 | 0.998 | 0.924 | 1 | 0.994 | 0.983 | 0.898 | 0.988 | 0.978 | 0.97 | 0.968 | 1 | 0.997 | 0.991 |
| 431 | 0.962 | 1 | 1 | 0.997 | 0.988 | 1 | 1 | 0.998 | 0.924 | 1 | 0.994 | 0.983 | 0.898 | 0.988 | 0.978 | 0.97 | 0.968 | 1 | 0.997 | 0.991 |
| 432 | 0.962 | 1 | 1 | 0.997 | 0.988 | 1 | 1 | 0.998 | 0.911 | 1 | 0.994 | 0.981 | 0.898 | 0.988 | 0.978 | 0.97 | 0.968 | 1 | 0.997 | 0.991 |
| 433 | 0.962 | 1 | 1 | 0.997 | 0.988 | 1 | 1 | 0.998 | 0.924 | 1 | 0.994 | 0.983 | 0.898 | 0.988 | 0.983 | 0.974 | 0.963 | 1 | 0.997 | 0.9899 |
| 434 | 0.962 | 1 | 1 | 0.997 | 0.988 | 1 | 1 | 0.998 | 0.924 | 1 | 0.994 | 0.983 | 0.898 | 0.988 | 0.983 | 0.974 | 0.963 | 1 | 0.997 | 0.9899 |
| 435 | 0.962 | 1 | 1 | 0.997 | 0.988 | 1 | 1 | 0.998 | 0.924 | 1 | 0.994 | 0.983 | 0.898 | 0.988 | 0.983 | 0.974 | 0.963 | 1 | 0.997 | 0.9899 |
| 436 | 0.962 | 1 | 1 | 0.997 | 0.988 | 1 | 1 | 0.998 | 0.924 | 1 | 0.994 | 0.983 | 0.898 | 0.988 | 0.983 | 0.974 | 0.963 | 1 | 0.997 | 0.9899 |
| 437 | 0.962 | 1 | 1 | 0.997 | 0.988 | 1 | 1 | 0.998 | 0.924 | 1 | 0.994 | 0.983 | 0.898 | 0.988 | 0.983 | 0.974 | 0.968 | 1 | 0.997 | 0.991 |
| 438 | 0.962 | 1 | 1 | 0.997 | 0.988 | 1 | 1 | 0.998 | 0.924 | 1 | 0.991 | 0.981 | 0.898 | 0.988 | 0.981 | 0.972 | 0.973 | 1 | 0.997 | 0.9922 |
| 439 | 0.962 | 1 | 1 | 0.997 | 0.988 | 1 | 1 | 0.998 | 0.924 | 1 | 0.991 | 0.981 | 0.898 | 0.988 | 0.981 | 0.972 | 0.968 | 1 | 0.997 | 0.991 |
| 440 | 0.962 | 1 | 1 | 0.997 | 0.988 | 1 | 1 | 0.998 | 0.924 | 1 | 0.991 | 0.981 | 0.898 | 0.988 | 0.981 | 0.972 | 0.973 | 1 | 0.997 | 0.9922 |
| 441 | 0.962 | 1 | 1 | 0.997 | 0.988 | 1 | 1 | 0.998 | 0.924 | 1 | 0.991 | 0.981 | 0.898 | 0.988 | 0.981 | 0.972 | 0.973 | 1 | 0.997 | 0.9922 |
| 442 | 0.962 | 1 | 1 | 0.997 | 0.988 | 1 | 1 | 0.998 | 0.924 | 1 | 0.991 | 0.981 | 0.898 | 0.988 | 0.981 | 0.972 | 0.973 | 1 | 0.997 | 0.9922 |
| 443 | 0.962 | 1 | 1 | 0.997 | 0.988 | 1 | 1 | 0.998 | 0.924 | 1 | 0.991 | 0.981 | 0.898 | 0.988 | 0.981 | 0.972 | 0.973 | 1 | 0.997 | 0.9922 |
| 444 | 0.962 | 1 | 1 | 0.997 | 0.988 | 1 | 1 | 0.998 | 0.924 | 1 | 0.991 | 0.981 | 0.898 | 0.988 | 0.981 | 0.972 | 0.973 | 1 | 0.997 | 0.9922 |
| 445 | 0.962 | 1 | 1 | 0.997 | 0.988 | 1 | 1 | 0.998 | 0.924 | 1 | 0.991 | 0.981 | 0.898 | 0.988 | 0.981 | 0.972 | 0.973 | 1 | 0.997 | 0.9922 |
| 446 | 0.962 | 1 | 1 | 0.997 | 0.988 | 1 | 1 | 0.998 | 0.924 | 1 | 0.991 | 0.981 | 0.898 | 0.988 | 0.981 | 0.972 | 0.979 | 1 | 0.997 | 0.9933 |
| 447 | 0.962 | 1 | 1 | 0.997 | 0.988 | 1 | 1 | 0.998 | 0.924 | 1 | 0.991 | 0.981 | 0.898 | 0.988 | 0.981 | 0.972 | 0.979 | 1 | 0.997 | 0.9933 |
| 448 | 0.962 | 1 | 1 | 0.997 | 0.988 | 1 | 1 | 0.998 | 0.924 | 1 | 0.991 | 0.981 | 0.898 | 0.988 | 0.983 | 0.974 | 0.979 | 1 | 0.997 | 0.9933 |
| 449 | 0.962 | 1 | 1 | 0.997 | 0.988 | 1 | 1 | 0.998 | 0.924 | 1 | 0.991 | 0.981 | 0.898 | 0.988 | 0.983 | 0.974 | 0.973 | 1 | 0.997 | 0.9922 |
| 450 | 0.962 | 1 | 1 | 0.997 | 0.988 | 1 | 1 | 0.998 | 0.924 | 1 | 0.991 | 0.981 | 0.898 | 0.988 | 0.983 | 0.974 | 0.973 | 1 | 0.997 | 0.9922 |
| 451 | 0.962 | 1 | 1 | 0.997 | 0.988 | 1 | 1 | 0.998 | 0.924 | 1 | 0.991 | 0.981 | 0.898 | 0.988 | 0.983 | 0.974 | 0.973 | 1 | 0.997 | 0.9922 |
| 452 | 0.962 | 1 | 1 | 0.997 | 0.988 | 1 | 1 | 0.998 | 0.924 | 1 | 0.991 | 0.981 | 0.898 | 0.988 | 0.983 | 0.974 | 0.973 | 1 | 0.997 | 0.9922 |
| 453 | 0.962 | 1 | 1 | 0.997 | 0.988 | 1 | 1 | 0.998 | 0.924 | 1 | 0.991 | 0.981 | 0.898 | 0.988 | 0.983 | 0.974 | 0.973 | 1 | 0.997 | 0.9922 |
| 454 | 0.962 | 1 | 1 | 0.997 | 0.988 | 1 | 1 | 0.998 | 0.924 | 1 | 0.991 | 0.981 | 0.898 | 0.988 | 0.983 | 0.974 | 0.979 | 1 | 0.995 | 0.9922 |
| 455 | 0.962 | 1 | 1 | 0.997 | 0.988 | 1 | 1 | 0.998 | 0.924 | 1 | 0.991 | 0.981 | 0.898 | 0.988 | 0.983 | 0.974 | 0.968 | 1 | 0.997 | 0.991 |
| 456 | 0.962 | 1 | 1 | 0.997 | 0.988 | 1 | 1 | 0.998 | 0.924 | 1 | 0.991 | 0.981 | 0.898 | 0.988 | 0.983 | 0.974 | 0.968 | 1 | 0.997 | 0.991 |
| 457 | 0.962 | 1 | 1 | 0.997 | 0.988 | 1 | 1 | 0.998 | 0.924 | 1 | 0.991 | 0.981 | 0.898 | 0.988 | 0.983 | 0.974 | 0.968 | 1 | 0.995 | 0.9899 |
| 458 | 0.962 | 1 | 1 | 0.997 | 0.988 | 1 | 1 | 0.998 | 0.924 | 1 | 0.991 | 0.981 | 0.898 | 0.988 | 0.983 | 0.974 | 0.968 | 1 | 0.995 | 0.9899 |
| 459 | 0.962 | 1 | 1 | 0.997 | 0.988 | 1 | 1 | 0.998 | 0.924 | 1 | 0.991 | 0.981 | 0.898 | 0.988 | 0.983 | 0.974 | 0.968 | 1 | 0.995 | 0.9899 |
| 460 | 0.962 | 1 | 1 | 0.997 | 0.988 | 1 | 1 | 0.998 | 0.924 | 1 | 0.991 | 0.981 | 0.898 | 0.988 | 0.983 | 0.974 | 0.968 | 1 | 0.995 | 0.9899 |
| 461 | 0.962 | 1 | 1 | 0.997 | 0.988 | 1 | 1 | 0.998 | 0.924 | 1 | 0.991 | 0.981 | 0.898 | 0.988 | 0.983 | 0.974 | 0.968 | 1 | 0.995 | 0.9899 |
| 462 | 0.962 | 1 | 1 | 0.997 | 0.988 | 1 | 1 | 0.998 | 0.924 | 1 | 0.991 | 0.981 | 0.898 | 0.988 | 0.983 | 0.974 | 0.963 | 1 | 0.995 | 0.9888 |
| 463 | 0.962 | 1 | 1 | 0.997 | 0.988 | 1 | 1 | 0.998 | 0.924 | 1 | 0.991 | 0.981 | 0.898 | 0.988 | 0.983 | 0.974 | 0.973 | 1 | 0.995 | 0.991 |
| 464 | 0.962 | 1 | 1 | 0.997 | 0.988 | 1 | 1 | 0.998 | 0.924 | 1 | 0.991 | 0.981 | 0.898 | 0.988 | 0.983 | 0.974 | 0.968 | 1 | 0.995 | 0.9899 |
| 465 | 0.962 | 1 | 1 | 0.997 | 0.988 | 1 | 1 | 0.998 | 0.924 | 1 | 0.991 | 0.981 | 0.898 | 0.988 | 0.983 | 0.974 | 0.963 | 1 | 0.997 | 0.9899 |
| 466 | 0.962 | 1 | 1 | 0.997 | 0.988 | 1 | 1 | 0.998 | 0.924 | 1 | 0.991 | 0.981 | 0.898 | 0.988 | 0.983 | 0.974 | 0.963 | 1 | 0.997 | 0.9899 |
| 467 | 0.962 | 1 | 1 | 0.997 | 0.988 | 1 | 1 | 0.998 | 0.924 | 1 | 0.991 | 0.981 | 0.898 | 0.988 | 0.983 | 0.974 | 0.963 | 1 | 0.997 | 0.9899 |
| 468 | 0.962 | 1 | 1 | 0.997 | 0.988 | 1 | 1 | 0.998 | 0.924 | 1 | 0.994 | 0.983 | 0.898 | 0.988 | 0.983 | 0.974 | 0.968 | 1 | 0.997 | 0.991 |
| 469 | 0.962 | 1 | 1 | 0.997 | 0.988 | 1 | 1 | 0.998 | 0.924 | 1 | 0.994 | 0.983 | 0.898 | 0.988 | 0.983 | 0.974 | 0.968 | 1 | 0.997 | 0.991 |
| 470 | 0.962 | 1 | 1 | 0.997 | 0.988 | 1 | 1 | 0.998 | 0.924 | 1 | 0.994 | 0.983 | 0.898 | 0.988 | 0.983 | 0.974 | 0.968 | 1 | 0.997 | 0.991 |
| 471 | 0.962 | 1 | 1 | 0.997 | 0.988 | 1 | 1 | 0.998 | 0.924 | 1 | 0.994 | 0.983 | 0.898 | 0.988 | 0.983 | 0.974 | 0.968 | 1 | 0.997 | 0.991 |
| 472 | 0.962 | 1 | 1 | 0.997 | 0.988 | 1 | 1 | 0.998 | 0.924 | 1 | 0.994 | 0.983 | 0.898 | 0.988 | 0.983 | 0.974 | 0.968 | 1 | 0.997 | 0.991 |
| 473 | 0.962 | 1 | 1 | 0.997 | 0.988 | 1 | 1 | 0.998 | 0.924 | 1 | 0.994 | 0.983 | 0.898 | 0.988 | 0.983 | 0.974 | 0.968 | 1 | 0.997 | 0.991 |
| 474 | 0.962 | 1 | 1 | 0.997 | 0.988 | 1 | 1 | 0.998 | 0.924 | 1 | 0.994 | 0.983 | 0.898 | 0.988 | 0.983 | 0.974 | 0.968 | 1 | 0.997 | 0.991 |
| 475 | 0.962 | 1 | 1 | 0.997 | 0.988 | 1 | 1 | 0.998 | 0.924 | 1 | 0.994 | 0.983 | 0.898 | 0.988 | 0.983 | 0.974 | 0.968 | 1 | 0.997 | 0.991 |
| 476 | 0.962 | 1 | 1 | 0.997 | 0.988 | 1 | 1 | 0.998 | 0.924 | 1 | 0.994 | 0.983 | 0.898 | 0.988 | 0.983 | 0.974 | 0.968 | 1 | 0.997 | 0.991 |
| 477 | 0.962 | 1 | 1 | 0.997 | 0.988 | 1 | 1 | 0.998 | 0.924 | 1 | 0.994 | 0.983 | 0.898 | 0.988 | 0.983 | 0.974 | 0.968 | 1 | 0.997 | 0.991 |
| 478 | 0.962 | 1 | 1 | 0.997 | 0.988 | 1 | 1 | 0.998 | 0.924 | 1 | 0.994 | 0.983 | 0.898 | 0.988 | 0.983 | 0.974 | 0.968 | 1 | 0.997 | 0.991 |
| 479 | 0.962 | 1 | 1 | 0.997 | 0.988 | 1 | 1 | 0.998 | 0.924 | 1 | 0.994 | 0.983 | 0.898 | 0.988 | 0.983 | 0.974 | 0.968 | 1 | 0.997 | 0.991 |
| 480 | 0.962 | 1 | 1 | 0.997 | 0.988 | 1 | 1 | 0.998 | 0.924 | 1 | 0.991 | 0.981 | 0.898 | 0.988 | 0.983 | 0.974 | 0.973 | 1 | 0.997 | 0.9922 |
| 481 | 0.962 | 1 | 1 | 0.997 | 0.988 | 1 | 1 | 0.998 | 0.924 | 1 | 0.991 | 0.981 | 0.898 | 0.988 | 0.983 | 0.974 | 0.973 | 1 | 0.997 | 0.9922 |
| 482 | 0.962 | 1 | 1 | 0.997 | 0.988 | 1 | 1 | 0.998 | 0.924 | 1 | 0.991 | 0.981 | 0.898 | 0.988 | 0.983 | 0.974 | 0.973 | 1 | 0.997 | 0.9922 |
| 483 | 0.962 | 1 | 1 | 0.997 | 0.988 | 1 | 1 | 0.998 | 0.924 | 1 | 0.994 | 0.983 | 0.898 | 0.988 | 0.983 | 0.974 | 0.973 | 1 | 0.995 | 0.991 |
| 484 | 0.962 | 1 | 1 | 0.997 | 0.988 | 1 | 1 | 0.998 | 0.924 | 1 | 0.994 | 0.983 | 0.898 | 0.988 | 0.983 | 0.974 | 0.973 | 1 | 0.995 | 0.991 |
| 485 | 0.962 | 1 | 1 | 0.997 | 0.988 | 1 | 1 | 0.998 | 0.924 | 1 | 0.994 | 0.983 | 0.898 | 0.988 | 0.983 | 0.974 | 0.968 | 1 | 0.997 | 0.991 |
| 486 | 0.962 | 1 | 1 | 0.997 | 0.988 | 1 | 1 | 0.998 | 0.924 | 1 | 0.994 | 0.983 | 0.898 | 0.988 | 0.983 | 0.974 | 0.968 | 1 | 0.997 | 0.991 |
| 487 | 0.962 | 1 | 1 | 0.997 | 0.988 | 1 | 1 | 0.998 | 0.924 | 1 | 0.994 | 0.983 | 0.898 | 0.988 | 0.983 | 0.974 | 0.968 | 1 | 0.997 | 0.991 |
| 488 | 0.962 | 1 | 1 | 0.997 | 0.988 | 1 | 1 | 0.998 | 0.924 | 1 | 0.994 | 0.983 | 0.898 | 0.988 | 0.983 | 0.974 | 0.968 | 1 | 0.997 | 0.991 |
| 489 | 0.962 | 1 | 1 | 0.997 | 0.988 | 1 | 1 | 0.998 | 0.924 | 1 | 0.994 | 0.983 | 0.898 | 0.988 | 0.983 | 0.974 | 0.968 | 1 | 0.997 | 0.991 |
| 490 | 0.962 | 1 | 1 | 0.997 | 0.988 | 1 | 1 | 0.998 | 0.924 | 1 | 0.994 | 0.983 | 0.898 | 0.988 | 0.983 | 0.974 | 0.973 | 1 | 0.997 | 0.9922 |
| 491 | 0.962 | 1 | 1 | 0.997 | 0.988 | 1 | 1 | 0.998 | 0.924 | 1 | 0.994 | 0.983 | 0.898 | 0.988 | 0.983 | 0.974 | 0.973 | 1 | 0.997 | 0.9922 |
| 492 | 0.962 | 1 | 1 | 0.997 | 0.988 | 1 | 1 | 0.998 | 0.924 | 1 | 0.994 | 0.983 | 0.898 | 0.988 | 0.983 | 0.974 | 0.973 | 1 | 0.997 | 0.9922 |
| 493 | 0.962 | 1 | 1 | 0.997 | 0.988 | 1 | 1 | 0.998 | 0.924 | 1 | 0.994 | 0.983 | 0.898 | 0.988 | 0.983 | 0.974 | 0.973 | 1 | 0.997 | 0.9922 |
| 494 | 0.962 | 1 | 1 | 0.997 | 0.988 | 1 | 1 | 0.998 | 0.924 | 1 | 0.994 | 0.983 | 0.898 | 0.988 | 0.983 | 0.974 | 0.973 | 1 | 0.997 | 0.9922 |
| 495 | 0.962 | 1 | 1 | 0.997 | 0.988 | 1 | 1 | 0.998 | 0.924 | 1 | 0.994 | 0.983 | 0.898 | 0.988 | 0.983 | 0.974 | 0.973 | 1 | 0.997 | 0.9922 |
| 496 | 0.962 | 1 | 1 | 0.997 | 0.988 | 1 | 1 | 0.998 | 0.924 | 1 | 0.994 | 0.983 | 0.898 | 0.988 | 0.983 | 0.974 | 0.968 | 1 | 0.997 | 0.991 |
| 497 | 0.962 | 1 | 1 | 0.997 | 0.988 | 1 | 1 | 0.998 | 0.924 | 1 | 0.988 | 0.979 | 0.898 | 0.988 | 0.981 | 0.972 | 0.973 | 1 | 0.997 | 0.9922 |
| 498 | 0.962 | 1 | 1 | 0.997 | 0.988 | 1 | 1 | 0.998 | 0.924 | 1 | 0.988 | 0.979 | 0.898 | 0.988 | 0.981 | 0.972 | 0.973 | 1 | 0.997 | 0.9922 |
| 499 | 0.962 | 1 | 1 | 0.997 | 0.988 | 1 | 1 | 0.998 | 0.924 | 1 | 0.988 | 0.979 | 0.898 | 0.988 | 0.981 | 0.972 | 0.973 | 1 | 0.997 | 0.9922 |
| 500 | 0.962 | 1 | 1 | 0.997 | 0.988 | 1 | 1 | 0.998 | 0.924 | 1 | 0.988 | 0.979 | 0.898 | 0.988 | 0.981 | 0.972 | 0.973 | 1 | 0.997 | 0.9922 |

1. The dataset of H0351.2002 was selected as the training dataset

| Number of features | H0351.1009 | | | | H0351.1012 | | | | H0351.1015 | | | | H0351.1016 | | | | H0351.2001 | | | |
| --- | --- | --- | --- | --- | --- | --- | --- | --- | --- | --- | --- | --- | --- | --- | --- | --- | --- | --- | --- | --- |
|  | class1 | class2 | class3 | total | class1 | class2 | class3 | total | class1 | class2 | class3 | total | class1 | class2 | class3 | total | class1 | class2 | class3 | total |
| 4 | 1 | 0.952 | 0.98 | 0.978 | 0.988 | 0.667 | 0.985 | 0.957 | 0.962 | 0.952 | 0.951 | 0.953 | 0.983 | 0.85 | 0.978 | 0.958 | 0.883 | 0.717 | 0.997 | 0.963 |
| 5 | 1 | 0.952 | 0.98 | 0.978 | 1 | 0.875 | 0.98 | 0.974 | 0.962 | 0.968 | 0.948 | 0.953 | 0.983 | 0.938 | 0.983 | 0.976 | 0.903 | 0.774 | 0.993 | 0.9662 |
| 6 | 1 | 0.952 | 0.966 | 0.967 | 1 | 0.896 | 0.97 | 0.968 | 0.987 | 0.968 | 0.964 | 0.968 | 1 | 0.95 | 0.981 | 0.978 | 0.955 | 0.792 | 0.992 | 0.9746 |
| 7 | 1 | 0.952 | 0.973 | 0.972 | 1 | 0.896 | 0.978 | 0.974 | 0.975 | 0.968 | 0.982 | 0.979 | 1 | 0.95 | 0.986 | 0.982 | 0.974 | 0.755 | 0.978 | 0.9651 |
| 8 | 1 | 0.952 | 0.976 | 0.975 | 1 | 0.896 | 0.98 | 0.975 | 0.987 | 0.968 | 0.982 | 0.981 | 1 | 0.938 | 0.989 | 0.982 | 0.981 | 0.755 | 0.98 | 0.9672 |
| 9 | 1 | 0.952 | 0.976 | 0.975 | 1 | 0.896 | 0.98 | 0.975 | 0.987 | 0.968 | 0.982 | 0.981 | 1 | 0.95 | 0.992 | 0.986 | 0.974 | 0.774 | 0.98 | 0.9672 |
| 10 | 1 | 0.952 | 0.976 | 0.975 | 1 | 0.896 | 0.97 | 0.968 | 1 | 0.968 | 0.982 | 0.983 | 1 | 0.95 | 0.978 | 0.976 | 0.987 | 0.774 | 0.969 | 0.9609 |
| 11 | 1 | 0.952 | 0.973 | 0.972 | 1 | 0.917 | 0.97 | 0.97 | 1 | 0.968 | 0.982 | 0.983 | 1 | 0.95 | 0.986 | 0.982 | 0.987 | 0.774 | 0.965 | 0.9577 |
| 12 | 1 | 0.952 | 0.969 | 0.97 | 1 | 0.896 | 0.963 | 0.962 | 1 | 0.968 | 0.982 | 0.983 | 1 | 0.925 | 0.983 | 0.976 | 0.974 | 0.792 | 0.966 | 0.9577 |
| 13 | 1 | 0.952 | 0.969 | 0.97 | 1 | 0.917 | 0.965 | 0.966 | 1 | 0.968 | 0.982 | 0.983 | 1 | 0.925 | 0.983 | 0.976 | 0.968 | 0.774 | 0.966 | 0.9556 |
| 14 | 1 | 0.952 | 0.959 | 0.961 | 1 | 0.917 | 0.955 | 0.958 | 1 | 0.968 | 0.979 | 0.981 | 1 | 0.925 | 0.986 | 0.978 | 0.961 | 0.774 | 0.969 | 0.9567 |
| 15 | 1 | 0.952 | 0.966 | 0.967 | 1 | 0.896 | 0.963 | 0.962 | 1 | 0.968 | 0.979 | 0.981 | 1 | 0.925 | 0.989 | 0.98 | 0.961 | 0.774 | 0.97 | 0.9577 |
| 16 | 1 | 0.952 | 0.963 | 0.964 | 1 | 0.896 | 0.96 | 0.96 | 1 | 0.968 | 0.976 | 0.979 | 1 | 0.925 | 0.992 | 0.982 | 0.955 | 0.774 | 0.97 | 0.9567 |
| 17 | 1 | 0.952 | 0.969 | 0.97 | 1 | 0.917 | 0.968 | 0.968 | 1 | 0.968 | 0.976 | 0.979 | 1 | 0.95 | 0.992 | 0.986 | 0.961 | 0.792 | 0.972 | 0.9598 |
| 18 | 1 | 0.952 | 0.966 | 0.967 | 1 | 0.917 | 0.96 | 0.962 | 1 | 0.968 | 0.973 | 0.977 | 1 | 0.95 | 0.992 | 0.986 | 0.961 | 0.83 | 0.972 | 0.9619 |
| 19 | 1 | 0.952 | 0.976 | 0.975 | 1 | 0.917 | 0.965 | 0.966 | 1 | 0.968 | 0.973 | 0.977 | 1 | 0.95 | 0.994 | 0.988 | 0.955 | 0.83 | 0.972 | 0.9609 |
| 20 | 1 | 0.952 | 0.976 | 0.975 | 1 | 0.917 | 0.963 | 0.964 | 1 | 0.968 | 0.976 | 0.979 | 1 | 0.95 | 0.994 | 0.988 | 0.961 | 0.83 | 0.97 | 0.9609 |
| 21 | 1 | 0.952 | 0.973 | 0.972 | 1 | 0.917 | 0.963 | 0.964 | 1 | 0.968 | 0.976 | 0.979 | 1 | 0.95 | 0.994 | 0.988 | 0.948 | 0.868 | 0.97 | 0.9609 |
| 22 | 1 | 0.952 | 0.973 | 0.972 | 1 | 0.917 | 0.965 | 0.966 | 1 | 0.968 | 0.976 | 0.979 | 1 | 0.95 | 0.994 | 0.988 | 0.948 | 0.887 | 0.972 | 0.963 |
| 23 | 1 | 0.952 | 0.976 | 0.975 | 1 | 0.917 | 0.965 | 0.966 | 1 | 0.968 | 0.976 | 0.979 | 1 | 0.95 | 0.994 | 0.988 | 0.948 | 0.887 | 0.969 | 0.9609 |
| 24 | 1 | 0.952 | 0.973 | 0.972 | 1 | 0.917 | 0.965 | 0.966 | 1 | 0.968 | 0.979 | 0.981 | 1 | 0.95 | 0.994 | 0.988 | 0.942 | 0.887 | 0.969 | 0.9598 |
| 25 | 1 | 0.952 | 0.973 | 0.972 | 1 | 0.917 | 0.965 | 0.966 | 1 | 0.968 | 0.982 | 0.983 | 1 | 0.95 | 0.994 | 0.988 | 0.948 | 0.887 | 0.969 | 0.9609 |
| 26 | 1 | 0.952 | 0.973 | 0.972 | 1 | 0.917 | 0.965 | 0.966 | 1 | 0.968 | 0.982 | 0.983 | 1 | 0.95 | 0.994 | 0.988 | 0.948 | 0.906 | 0.969 | 0.9619 |
| 27 | 1 | 0.952 | 0.946 | 0.95 | 1 | 0.917 | 0.958 | 0.96 | 1 | 0.968 | 0.979 | 0.981 | 1 | 0.95 | 0.986 | 0.982 | 0.955 | 0.887 | 0.962 | 0.9567 |
| 28 | 1 | 0.952 | 0.959 | 0.961 | 1 | 0.938 | 0.96 | 0.964 | 1 | 0.968 | 0.979 | 0.981 | 1 | 0.95 | 0.989 | 0.984 | 0.948 | 0.906 | 0.963 | 0.9577 |
| 29 | 1 | 0.952 | 0.963 | 0.964 | 1 | 0.917 | 0.965 | 0.966 | 1 | 0.968 | 0.982 | 0.983 | 1 | 0.95 | 0.992 | 0.986 | 0.955 | 0.887 | 0.965 | 0.9588 |
| 30 | 1 | 0.952 | 0.956 | 0.959 | 1 | 0.938 | 0.965 | 0.968 | 1 | 0.968 | 0.982 | 0.983 | 1 | 0.95 | 0.989 | 0.984 | 0.955 | 0.887 | 0.966 | 0.9598 |
| 31 | 1 | 0.952 | 0.953 | 0.956 | 1 | 0.938 | 0.963 | 0.966 | 1 | 0.968 | 0.976 | 0.979 | 1 | 0.95 | 0.989 | 0.984 | 0.955 | 0.887 | 0.962 | 0.9567 |
| 32 | 1 | 0.952 | 0.953 | 0.956 | 1 | 0.938 | 0.96 | 0.964 | 1 | 0.968 | 0.979 | 0.981 | 1 | 0.95 | 0.989 | 0.984 | 0.955 | 0.887 | 0.963 | 0.9577 |
| 33 | 1 | 0.952 | 0.953 | 0.956 | 1 | 0.938 | 0.963 | 0.966 | 1 | 0.968 | 0.979 | 0.981 | 1 | 0.95 | 0.989 | 0.984 | 0.955 | 0.906 | 0.963 | 0.9588 |
| 34 | 1 | 0.952 | 0.959 | 0.961 | 1 | 0.938 | 0.968 | 0.97 | 1 | 0.968 | 0.985 | 0.985 | 1 | 0.95 | 0.989 | 0.984 | 0.955 | 0.887 | 0.965 | 0.9588 |
| 35 | 1 | 0.952 | 0.963 | 0.964 | 1 | 0.938 | 0.968 | 0.97 | 1 | 0.968 | 0.988 | 0.987 | 1 | 0.95 | 0.992 | 0.986 | 0.955 | 0.887 | 0.966 | 0.9598 |
| 36 | 1 | 0.952 | 0.98 | 0.978 | 1 | 0.938 | 0.97 | 0.972 | 1 | 0.968 | 0.988 | 0.987 | 1 | 0.95 | 0.994 | 0.988 | 0.942 | 0.925 | 0.965 | 0.9588 |
| 37 | 1 | 0.952 | 0.976 | 0.975 | 1 | 0.938 | 0.968 | 0.97 | 1 | 0.968 | 0.988 | 0.987 | 1 | 0.95 | 0.994 | 0.988 | 0.942 | 0.925 | 0.965 | 0.9588 |
| 38 | 1 | 0.952 | 0.976 | 0.975 | 1 | 0.938 | 0.968 | 0.97 | 1 | 0.968 | 0.988 | 0.987 | 1 | 0.95 | 0.994 | 0.988 | 0.942 | 0.906 | 0.966 | 0.9588 |
| 39 | 1 | 0.952 | 0.969 | 0.97 | 1 | 0.938 | 0.97 | 0.972 | 1 | 0.968 | 0.988 | 0.987 | 1 | 0.95 | 0.994 | 0.988 | 0.948 | 0.906 | 0.966 | 0.9598 |
| 40 | 1 | 0.952 | 0.969 | 0.97 | 1 | 0.938 | 0.968 | 0.97 | 1 | 0.968 | 0.988 | 0.987 | 1 | 0.95 | 0.994 | 0.988 | 0.948 | 0.906 | 0.966 | 0.9598 |
| 41 | 1 | 0.952 | 0.969 | 0.97 | 1 | 0.938 | 0.97 | 0.972 | 1 | 0.968 | 0.988 | 0.987 | 1 | 0.95 | 0.994 | 0.988 | 0.935 | 0.906 | 0.966 | 0.9577 |
| 42 | 1 | 0.952 | 0.969 | 0.97 | 1 | 0.938 | 0.97 | 0.972 | 1 | 0.968 | 0.988 | 0.987 | 1 | 0.95 | 0.994 | 0.988 | 0.935 | 0.906 | 0.963 | 0.9556 |
| 43 | 1 | 0.952 | 0.98 | 0.978 | 1 | 0.938 | 0.97 | 0.972 | 1 | 0.968 | 0.988 | 0.987 | 0.983 | 0.95 | 0.994 | 0.986 | 0.935 | 0.906 | 0.962 | 0.9545 |
| 44 | 1 | 0.952 | 0.986 | 0.983 | 1 | 0.938 | 0.97 | 0.972 | 1 | 0.968 | 0.988 | 0.987 | 0.983 | 0.95 | 0.994 | 0.986 | 0.942 | 0.906 | 0.965 | 0.9577 |
| 45 | 1 | 0.952 | 0.986 | 0.983 | 1 | 0.938 | 0.97 | 0.972 | 1 | 0.968 | 0.991 | 0.989 | 0.983 | 0.95 | 0.994 | 0.986 | 0.942 | 0.906 | 0.966 | 0.9588 |
| 46 | 1 | 0.952 | 0.983 | 0.981 | 1 | 0.938 | 0.97 | 0.972 | 1 | 0.968 | 0.991 | 0.989 | 0.983 | 0.95 | 0.994 | 0.986 | 0.942 | 0.906 | 0.965 | 0.9577 |
| 47 | 1 | 0.952 | 0.98 | 0.978 | 1 | 0.917 | 0.97 | 0.97 | 1 | 0.968 | 0.991 | 0.989 | 0.983 | 0.95 | 0.994 | 0.986 | 0.942 | 0.868 | 0.966 | 0.9567 |
| 48 | 1 | 0.952 | 0.983 | 0.981 | 1 | 0.938 | 0.973 | 0.974 | 1 | 0.968 | 0.994 | 0.991 | 0.983 | 0.95 | 0.994 | 0.986 | 0.955 | 0.849 | 0.965 | 0.9567 |
| 49 | 1 | 0.952 | 0.986 | 0.983 | 1 | 0.938 | 0.973 | 0.974 | 1 | 0.968 | 0.994 | 0.991 | 0.983 | 0.95 | 0.997 | 0.988 | 0.955 | 0.849 | 0.966 | 0.9577 |
| 50 | 1 | 0.952 | 0.986 | 0.983 | 1 | 0.938 | 0.973 | 0.974 | 1 | 0.968 | 0.994 | 0.991 | 0.983 | 0.95 | 0.997 | 0.988 | 0.955 | 0.849 | 0.966 | 0.9577 |
| 51 | 1 | 0.952 | 0.986 | 0.983 | 1 | 0.938 | 0.973 | 0.974 | 1 | 0.968 | 0.994 | 0.991 | 0.983 | 0.95 | 0.997 | 0.988 | 0.955 | 0.849 | 0.968 | 0.9588 |
| 52 | 1 | 0.952 | 0.983 | 0.981 | 1 | 0.938 | 0.973 | 0.974 | 1 | 0.968 | 0.994 | 0.991 | 0.983 | 0.95 | 0.997 | 0.988 | 0.961 | 0.849 | 0.972 | 0.963 |
| 53 | 1 | 0.952 | 0.983 | 0.981 | 1 | 0.938 | 0.973 | 0.974 | 1 | 0.968 | 0.991 | 0.989 | 0.983 | 0.95 | 0.997 | 0.988 | 0.948 | 0.868 | 0.973 | 0.963 |
| 54 | 1 | 0.952 | 0.983 | 0.981 | 1 | 0.917 | 0.973 | 0.972 | 1 | 0.968 | 0.991 | 0.989 | 1 | 0.95 | 0.997 | 0.99 | 0.955 | 0.849 | 0.973 | 0.963 |
| 55 | 1 | 0.952 | 0.98 | 0.978 | 1 | 0.917 | 0.973 | 0.972 | 1 | 0.968 | 0.991 | 0.989 | 1 | 0.95 | 0.997 | 0.99 | 0.955 | 0.849 | 0.972 | 0.9619 |
| 56 | 1 | 0.952 | 0.983 | 0.981 | 1 | 0.917 | 0.973 | 0.972 | 1 | 0.968 | 0.991 | 0.989 | 0.983 | 0.963 | 0.997 | 0.99 | 0.955 | 0.849 | 0.972 | 0.9619 |
| 57 | 1 | 0.952 | 0.949 | 0.953 | 1 | 0.917 | 0.97 | 0.97 | 1 | 0.968 | 0.991 | 0.989 | 1 | 0.963 | 0.994 | 0.99 | 0.942 | 0.849 | 0.957 | 0.9482 |
| 58 | 1 | 0.952 | 0.956 | 0.959 | 1 | 0.917 | 0.97 | 0.97 | 1 | 0.968 | 0.991 | 0.989 | 1 | 0.963 | 0.994 | 0.99 | 0.948 | 0.849 | 0.957 | 0.9493 |
| 59 | 1 | 0.952 | 0.956 | 0.959 | 1 | 0.917 | 0.97 | 0.97 | 1 | 0.968 | 0.991 | 0.989 | 1 | 0.963 | 0.994 | 0.99 | 0.955 | 0.849 | 0.957 | 0.9503 |
| 60 | 1 | 0.952 | 0.956 | 0.959 | 1 | 0.917 | 0.97 | 0.97 | 1 | 0.968 | 0.991 | 0.989 | 1 | 0.975 | 0.994 | 0.992 | 0.955 | 0.868 | 0.957 | 0.9514 |
| 61 | 1 | 0.952 | 0.956 | 0.959 | 1 | 0.917 | 0.97 | 0.97 | 1 | 0.968 | 0.991 | 0.989 | 1 | 0.975 | 0.994 | 0.992 | 0.961 | 0.868 | 0.951 | 0.9482 |
| 62 | 1 | 0.952 | 0.949 | 0.953 | 1 | 0.938 | 0.968 | 0.97 | 1 | 0.968 | 0.991 | 0.989 | 1 | 0.975 | 0.992 | 0.99 | 0.961 | 0.868 | 0.957 | 0.9524 |
| 63 | 1 | 0.952 | 0.953 | 0.956 | 1 | 0.938 | 0.97 | 0.972 | 1 | 0.984 | 0.991 | 0.991 | 1 | 0.975 | 0.992 | 0.99 | 0.961 | 0.887 | 0.959 | 0.9556 |
| 64 | 1 | 0.952 | 0.956 | 0.959 | 1 | 0.917 | 0.97 | 0.97 | 1 | 0.968 | 0.991 | 0.989 | 1 | 0.975 | 0.992 | 0.99 | 0.961 | 0.887 | 0.958 | 0.9545 |
| 65 | 1 | 0.952 | 0.956 | 0.959 | 1 | 0.917 | 0.97 | 0.97 | 1 | 0.968 | 0.991 | 0.989 | 1 | 0.975 | 0.989 | 0.988 | 0.961 | 0.887 | 0.957 | 0.9535 |
| 66 | 1 | 0.952 | 0.959 | 0.961 | 1 | 0.917 | 0.97 | 0.97 | 1 | 0.968 | 0.991 | 0.989 | 1 | 0.975 | 0.989 | 0.988 | 0.955 | 0.887 | 0.957 | 0.9524 |
| 67 | 1 | 0.952 | 0.969 | 0.97 | 1 | 0.917 | 0.973 | 0.972 | 1 | 0.968 | 0.991 | 0.989 | 1 | 0.975 | 0.997 | 0.994 | 0.948 | 0.887 | 0.963 | 0.9567 |
| 68 | 1 | 0.952 | 0.973 | 0.972 | 1 | 0.917 | 0.975 | 0.974 | 1 | 0.968 | 0.991 | 0.989 | 1 | 0.975 | 0.994 | 0.992 | 0.948 | 0.887 | 0.968 | 0.9598 |
| 69 | 1 | 0.952 | 0.969 | 0.97 | 1 | 0.917 | 0.975 | 0.974 | 1 | 0.968 | 0.991 | 0.989 | 1 | 0.975 | 0.992 | 0.99 | 0.935 | 0.887 | 0.966 | 0.9567 |
| 70 | 1 | 0.952 | 0.969 | 0.97 | 1 | 0.917 | 0.975 | 0.974 | 1 | 0.968 | 0.991 | 0.989 | 1 | 0.975 | 0.992 | 0.99 | 0.935 | 0.887 | 0.965 | 0.9556 |
| 71 | 1 | 0.952 | 0.969 | 0.97 | 1 | 0.917 | 0.973 | 0.972 | 1 | 0.984 | 0.991 | 0.991 | 1 | 0.963 | 0.992 | 0.988 | 0.935 | 0.887 | 0.965 | 0.9556 |
| 72 | 1 | 0.952 | 0.969 | 0.97 | 1 | 0.917 | 0.973 | 0.972 | 1 | 0.984 | 0.991 | 0.991 | 1 | 0.963 | 0.992 | 0.988 | 0.935 | 0.868 | 0.966 | 0.9556 |
| 73 | 1 | 0.952 | 0.969 | 0.97 | 1 | 0.917 | 0.973 | 0.972 | 1 | 0.984 | 0.991 | 0.991 | 1 | 0.963 | 0.992 | 0.988 | 0.935 | 0.868 | 0.966 | 0.9556 |
| 74 | 1 | 0.952 | 0.969 | 0.97 | 1 | 0.917 | 0.973 | 0.972 | 1 | 0.984 | 0.991 | 0.991 | 1 | 0.963 | 0.992 | 0.988 | 0.935 | 0.887 | 0.968 | 0.9577 |
| 75 | 1 | 0.952 | 0.969 | 0.97 | 1 | 0.917 | 0.973 | 0.972 | 1 | 0.984 | 0.991 | 0.991 | 1 | 0.963 | 0.992 | 0.988 | 0.935 | 0.887 | 0.968 | 0.9577 |
| 76 | 1 | 0.952 | 0.969 | 0.97 | 1 | 0.917 | 0.973 | 0.972 | 1 | 0.984 | 0.991 | 0.991 | 1 | 0.963 | 0.992 | 0.988 | 0.935 | 0.887 | 0.969 | 0.9588 |
| 77 | 1 | 0.952 | 0.969 | 0.97 | 1 | 0.917 | 0.973 | 0.972 | 1 | 0.984 | 0.991 | 0.991 | 1 | 0.963 | 0.992 | 0.988 | 0.935 | 0.887 | 0.968 | 0.9577 |
| 78 | 1 | 0.952 | 0.969 | 0.97 | 1 | 0.917 | 0.97 | 0.97 | 1 | 0.984 | 0.991 | 0.991 | 1 | 0.963 | 0.994 | 0.99 | 0.935 | 0.868 | 0.968 | 0.9567 |
| 79 | 1 | 0.952 | 0.969 | 0.97 | 1 | 0.917 | 0.97 | 0.97 | 1 | 0.984 | 0.991 | 0.991 | 1 | 0.963 | 0.994 | 0.99 | 0.935 | 0.868 | 0.968 | 0.9567 |
| 80 | 1 | 0.976 | 0.973 | 0.975 | 1 | 0.917 | 0.97 | 0.97 | 1 | 0.984 | 0.991 | 0.991 | 1 | 0.963 | 0.994 | 0.99 | 0.935 | 0.887 | 0.969 | 0.9588 |
| 81 | 1 | 0.976 | 0.973 | 0.975 | 1 | 0.917 | 0.97 | 0.97 | 1 | 1 | 0.991 | 0.994 | 1 | 0.988 | 0.994 | 0.994 | 0.935 | 0.962 | 0.969 | 0.963 |
| 82 | 1 | 1 | 0.969 | 0.975 | 1 | 0.917 | 0.968 | 0.968 | 1 | 1 | 0.991 | 0.994 | 1 | 0.988 | 0.992 | 0.992 | 0.935 | 0.981 | 0.962 | 0.9588 |
| 83 | 1 | 1 | 0.969 | 0.975 | 1 | 0.917 | 0.968 | 0.968 | 1 | 1 | 0.991 | 0.994 | 1 | 0.988 | 0.992 | 0.992 | 0.935 | 0.981 | 0.962 | 0.9588 |
| 84 | 1 | 0.976 | 0.969 | 0.972 | 1 | 0.917 | 0.968 | 0.968 | 1 | 1 | 0.991 | 0.994 | 1 | 0.988 | 0.992 | 0.992 | 0.942 | 0.962 | 0.962 | 0.9588 |
| 85 | 1 | 1 | 0.969 | 0.975 | 1 | 0.917 | 0.968 | 0.968 | 1 | 1 | 0.991 | 0.994 | 1 | 0.988 | 0.992 | 0.992 | 0.942 | 0.981 | 0.962 | 0.9598 |
| 86 | 1 | 1 | 0.969 | 0.975 | 0.988 | 0.917 | 0.968 | 0.966 | 1 | 1 | 0.991 | 0.994 | 1 | 0.988 | 0.992 | 0.992 | 0.935 | 0.981 | 0.963 | 0.9598 |
| 87 | 1 | 1 | 0.969 | 0.975 | 0.988 | 0.917 | 0.968 | 0.966 | 1 | 1 | 0.991 | 0.994 | 0.983 | 0.988 | 0.994 | 0.992 | 0.929 | 0.981 | 0.962 | 0.9577 |
| 88 | 1 | 1 | 0.969 | 0.975 | 0.988 | 0.917 | 0.968 | 0.966 | 1 | 1 | 0.991 | 0.994 | 0.983 | 0.988 | 0.994 | 0.992 | 0.922 | 0.981 | 0.962 | 0.9567 |
| 89 | 1 | 1 | 0.98 | 0.983 | 0.988 | 0.917 | 0.978 | 0.974 | 1 | 1 | 0.994 | 0.996 | 0.983 | 0.988 | 0.994 | 0.992 | 0.935 | 0.981 | 0.974 | 0.9683 |
| 90 | 1 | 1 | 0.98 | 0.983 | 0.988 | 0.917 | 0.98 | 0.975 | 1 | 1 | 0.994 | 0.996 | 0.983 | 0.988 | 0.994 | 0.992 | 0.935 | 0.981 | 0.976 | 0.9693 |
| 91 | 1 | 1 | 0.98 | 0.983 | 0.988 | 0.917 | 0.98 | 0.975 | 1 | 1 | 0.994 | 0.996 | 0.983 | 0.988 | 0.994 | 0.992 | 0.929 | 0.981 | 0.974 | 0.9672 |
| 92 | 1 | 1 | 0.976 | 0.981 | 0.988 | 0.917 | 0.98 | 0.975 | 1 | 1 | 0.991 | 0.994 | 0.983 | 0.988 | 0.994 | 0.992 | 0.935 | 0.981 | 0.974 | 0.9683 |
| 93 | 1 | 1 | 0.976 | 0.981 | 0.988 | 0.917 | 0.98 | 0.975 | 1 | 1 | 0.991 | 0.994 | 0.983 | 0.988 | 0.994 | 0.992 | 0.929 | 0.981 | 0.974 | 0.9672 |
| 94 | 1 | 1 | 0.976 | 0.981 | 0.988 | 0.917 | 0.98 | 0.975 | 0.987 | 1 | 0.994 | 0.994 | 0.983 | 0.988 | 0.994 | 0.992 | 0.942 | 0.981 | 0.972 | 0.9672 |
| 95 | 1 | 1 | 0.976 | 0.981 | 0.988 | 0.917 | 0.978 | 0.974 | 0.987 | 1 | 0.994 | 0.994 | 0.983 | 0.988 | 0.994 | 0.992 | 0.942 | 0.981 | 0.972 | 0.9672 |
| 96 | 1 | 1 | 0.98 | 0.983 | 0.988 | 0.917 | 0.973 | 0.97 | 1 | 1 | 0.994 | 0.996 | 0.983 | 0.988 | 0.994 | 0.992 | 0.942 | 0.981 | 0.972 | 0.9672 |
| 97 | 1 | 1 | 0.98 | 0.983 | 0.988 | 0.917 | 0.973 | 0.97 | 1 | 1 | 0.991 | 0.994 | 0.983 | 0.988 | 0.994 | 0.992 | 0.942 | 0.981 | 0.969 | 0.9651 |
| 98 | 1 | 1 | 0.98 | 0.983 | 0.988 | 0.917 | 0.975 | 0.972 | 0.987 | 1 | 0.994 | 0.994 | 0.983 | 0.988 | 0.994 | 0.992 | 0.929 | 0.981 | 0.973 | 0.9662 |
| 99 | 1 | 1 | 0.98 | 0.983 | 0.988 | 0.917 | 0.983 | 0.977 | 0.987 | 1 | 0.994 | 0.994 | 0.983 | 0.975 | 0.994 | 0.99 | 0.948 | 0.981 | 0.97 | 0.9672 |
| 100 | 1 | 1 | 0.98 | 0.983 | 0.988 | 0.917 | 0.975 | 0.972 | 1 | 1 | 0.994 | 0.996 | 0.983 | 0.988 | 0.994 | 0.992 | 0.948 | 0.981 | 0.965 | 0.963 |
| 101 | 1 | 1 | 0.976 | 0.981 | 1 | 0.917 | 0.973 | 0.972 | 1 | 1 | 0.994 | 0.996 | 0.983 | 0.988 | 0.994 | 0.992 | 0.955 | 0.981 | 0.963 | 0.963 |
| 102 | 1 | 1 | 0.976 | 0.981 | 1 | 0.917 | 0.973 | 0.972 | 1 | 1 | 0.994 | 0.996 | 0.983 | 0.988 | 0.994 | 0.992 | 0.955 | 0.981 | 0.963 | 0.963 |
| 103 | 1 | 1 | 0.973 | 0.978 | 1 | 0.917 | 0.973 | 0.972 | 1 | 1 | 0.988 | 0.991 | 0.983 | 0.988 | 0.994 | 0.992 | 0.955 | 0.981 | 0.955 | 0.9567 |
| 104 | 1 | 1 | 0.973 | 0.978 | 1 | 0.917 | 0.973 | 0.972 | 1 | 1 | 0.991 | 0.994 | 0.983 | 0.975 | 0.994 | 0.99 | 0.955 | 0.943 | 0.953 | 0.9524 |
| 105 | 1 | 1 | 0.969 | 0.975 | 1 | 0.917 | 0.973 | 0.972 | 1 | 1 | 0.991 | 0.994 | 0.983 | 0.963 | 0.994 | 0.988 | 0.955 | 0.943 | 0.955 | 0.9545 |
| 106 | 1 | 1 | 0.966 | 0.972 | 1 | 0.917 | 0.97 | 0.97 | 1 | 1 | 0.988 | 0.991 | 0.983 | 0.963 | 0.994 | 0.988 | 0.955 | 0.943 | 0.953 | 0.9524 |
| 107 | 1 | 1 | 0.966 | 0.972 | 1 | 0.917 | 0.97 | 0.97 | 1 | 1 | 0.988 | 0.991 | 0.983 | 0.963 | 0.994 | 0.988 | 0.955 | 0.943 | 0.953 | 0.9524 |
| 108 | 1 | 1 | 0.969 | 0.975 | 1 | 0.917 | 0.965 | 0.966 | 1 | 1 | 0.988 | 0.991 | 0.983 | 0.975 | 0.994 | 0.99 | 0.948 | 0.943 | 0.954 | 0.9524 |
| 109 | 1 | 1 | 0.966 | 0.972 | 1 | 0.917 | 0.963 | 0.964 | 1 | 1 | 0.985 | 0.989 | 0.983 | 0.988 | 0.994 | 0.992 | 0.955 | 0.943 | 0.951 | 0.9514 |
| 110 | 1 | 1 | 0.969 | 0.975 | 1 | 0.917 | 0.963 | 0.964 | 1 | 1 | 0.988 | 0.991 | 0.983 | 0.975 | 0.994 | 0.99 | 0.948 | 0.943 | 0.953 | 0.9514 |
| 111 | 1 | 1 | 0.969 | 0.975 | 1 | 0.917 | 0.963 | 0.964 | 1 | 1 | 0.988 | 0.991 | 0.983 | 0.975 | 0.994 | 0.99 | 0.948 | 0.943 | 0.953 | 0.9514 |
| 112 | 1 | 1 | 0.966 | 0.972 | 1 | 0.917 | 0.965 | 0.966 | 1 | 1 | 0.988 | 0.991 | 0.983 | 0.975 | 0.997 | 0.992 | 0.942 | 0.943 | 0.957 | 0.9535 |
| 113 | 1 | 1 | 0.966 | 0.972 | 1 | 0.917 | 0.963 | 0.964 | 1 | 1 | 0.988 | 0.991 | 0.983 | 0.975 | 0.997 | 0.992 | 0.942 | 0.943 | 0.958 | 0.9545 |
| 114 | 1 | 1 | 0.966 | 0.972 | 1 | 0.917 | 0.963 | 0.964 | 1 | 1 | 0.988 | 0.991 | 0.983 | 0.975 | 0.997 | 0.992 | 0.942 | 0.943 | 0.959 | 0.9556 |
| 115 | 1 | 1 | 0.966 | 0.972 | 1 | 0.917 | 0.965 | 0.966 | 1 | 1 | 0.988 | 0.991 | 0.983 | 0.975 | 0.992 | 0.988 | 0.935 | 0.943 | 0.961 | 0.9556 |
| 116 | 1 | 1 | 0.966 | 0.972 | 1 | 0.917 | 0.965 | 0.966 | 1 | 1 | 0.988 | 0.991 | 0.983 | 0.975 | 0.992 | 0.988 | 0.935 | 0.943 | 0.961 | 0.9556 |
| 117 | 1 | 1 | 0.983 | 0.986 | 1 | 0.917 | 0.963 | 0.964 | 1 | 1 | 0.991 | 0.994 | 1 | 0.975 | 0.997 | 0.994 | 0.935 | 0.943 | 0.968 | 0.9609 |
| 118 | 1 | 1 | 0.983 | 0.986 | 1 | 0.917 | 0.973 | 0.972 | 1 | 1 | 0.991 | 0.994 | 0.983 | 0.988 | 0.997 | 0.994 | 0.935 | 0.943 | 0.966 | 0.9598 |
| 119 | 1 | 1 | 0.983 | 0.986 | 1 | 0.917 | 0.973 | 0.972 | 1 | 1 | 0.991 | 0.994 | 0.983 | 0.988 | 0.997 | 0.994 | 0.935 | 0.943 | 0.966 | 0.9598 |
| 120 | 1 | 1 | 0.983 | 0.986 | 1 | 0.917 | 0.968 | 0.968 | 1 | 1 | 0.991 | 0.994 | 0.983 | 0.975 | 0.997 | 0.992 | 0.935 | 0.943 | 0.966 | 0.9598 |
| 121 | 1 | 1 | 0.983 | 0.986 | 1 | 0.917 | 0.965 | 0.966 | 1 | 1 | 0.991 | 0.994 | 0.983 | 0.975 | 0.997 | 0.992 | 0.935 | 0.925 | 0.966 | 0.9588 |
| 122 | 1 | 1 | 0.983 | 0.986 | 1 | 0.917 | 0.968 | 0.968 | 1 | 1 | 0.991 | 0.994 | 0.983 | 0.988 | 0.997 | 0.994 | 0.935 | 0.925 | 0.966 | 0.9588 |
| 123 | 1 | 0.976 | 0.976 | 0.978 | 1 | 0.917 | 0.965 | 0.966 | 1 | 0.984 | 0.994 | 0.994 | 0.983 | 0.95 | 0.997 | 0.988 | 0.916 | 0.811 | 0.97 | 0.9524 |
| 124 | 1 | 0.976 | 0.976 | 0.978 | 1 | 0.917 | 0.965 | 0.966 | 1 | 0.984 | 0.994 | 0.994 | 0.983 | 0.95 | 0.997 | 0.988 | 0.916 | 0.811 | 0.969 | 0.9514 |
| 125 | 1 | 0.976 | 0.976 | 0.978 | 1 | 0.917 | 0.965 | 0.966 | 1 | 0.984 | 0.994 | 0.994 | 0.983 | 0.95 | 0.997 | 0.988 | 0.916 | 0.792 | 0.97 | 0.9514 |
| 126 | 1 | 0.976 | 0.976 | 0.978 | 1 | 0.917 | 0.965 | 0.966 | 1 | 0.984 | 0.994 | 0.994 | 0.983 | 0.95 | 0.997 | 0.988 | 0.916 | 0.792 | 0.969 | 0.9503 |
| 127 | 1 | 0.976 | 0.98 | 0.981 | 1 | 0.917 | 0.968 | 0.968 | 1 | 0.984 | 0.994 | 0.994 | 0.983 | 0.95 | 0.997 | 0.988 | 0.916 | 0.792 | 0.97 | 0.9514 |
| 128 | 1 | 0.976 | 0.976 | 0.978 | 1 | 0.917 | 0.965 | 0.966 | 1 | 0.984 | 0.994 | 0.994 | 0.983 | 0.95 | 0.997 | 0.988 | 0.916 | 0.792 | 0.97 | 0.9514 |
| 129 | 1 | 0.976 | 0.98 | 0.981 | 1 | 0.917 | 0.968 | 0.968 | 1 | 0.984 | 0.994 | 0.994 | 0.983 | 0.95 | 0.997 | 0.988 | 0.916 | 0.792 | 0.97 | 0.9514 |
| 130 | 1 | 0.976 | 0.98 | 0.981 | 1 | 0.917 | 0.965 | 0.966 | 1 | 0.984 | 0.994 | 0.994 | 0.983 | 0.95 | 0.997 | 0.988 | 0.922 | 0.792 | 0.969 | 0.9514 |
| 131 | 1 | 0.976 | 0.983 | 0.983 | 1 | 0.917 | 0.963 | 0.964 | 1 | 0.984 | 0.994 | 0.994 | 0.983 | 0.95 | 0.997 | 0.988 | 0.916 | 0.83 | 0.969 | 0.9524 |
| 132 | 1 | 0.976 | 0.983 | 0.983 | 1 | 0.917 | 0.963 | 0.964 | 1 | 0.984 | 0.994 | 0.994 | 0.983 | 0.95 | 0.997 | 0.988 | 0.922 | 0.811 | 0.969 | 0.9524 |
| 133 | 1 | 0.976 | 0.983 | 0.983 | 1 | 0.917 | 0.963 | 0.964 | 1 | 0.984 | 0.994 | 0.994 | 0.983 | 0.975 | 0.997 | 0.992 | 0.922 | 0.83 | 0.969 | 0.9535 |
| 134 | 1 | 0.976 | 0.98 | 0.981 | 1 | 0.917 | 0.963 | 0.964 | 1 | 0.984 | 0.994 | 0.994 | 0.983 | 0.975 | 0.997 | 0.992 | 0.922 | 0.849 | 0.969 | 0.9545 |
| 135 | 1 | 0.976 | 0.976 | 0.978 | 1 | 0.917 | 0.96 | 0.962 | 1 | 0.984 | 0.991 | 0.991 | 0.983 | 0.975 | 0.997 | 0.992 | 0.922 | 0.83 | 0.965 | 0.9503 |
| 136 | 1 | 0.976 | 0.98 | 0.981 | 1 | 0.917 | 0.968 | 0.968 | 1 | 0.984 | 0.991 | 0.991 | 0.983 | 0.975 | 0.997 | 0.992 | 0.916 | 0.83 | 0.968 | 0.9514 |
| 137 | 1 | 0.976 | 0.983 | 0.983 | 1 | 0.917 | 0.968 | 0.968 | 1 | 0.984 | 0.991 | 0.991 | 0.983 | 0.963 | 0.997 | 0.99 | 0.916 | 0.83 | 0.969 | 0.9524 |
[truncated: 54,896 more chars]
