# Supplementary material for: Analysis of Gene Expression Profiles in the Human Brain Stem, Cerebellum and Cerebral Cortex
Source: PLoS One. 2016 Jul 19;11(7):e0159395. doi: 10.1371/journal.pone.0159395 (PMC4951119; doi:10.1371/journal.pone.0159395)
Supplement: S3 Table — (DOCX) [file pone.0159395.s009.docx]

**S3 Table.** The numbers of features (genes) yielding the maximum total prediction accuracy for pairs of training and test datasets ^a^

| **Code of people** | H0351.1009 | H0351.1012 | H0351.1015 | H0351.1016 | H0351.2001 | H0351.2002 |
| --- | --- | --- | --- | --- | --- | --- |
| H0351.1009 | --- | 32, 0.994 | 30, 0.972 | 34, 0.982 | 11, 0.978 | 5, 0.951 |
| H0351.1012 | 8, 1.000 | --- | 32, 0.994 | 10, 0.998 | 56, 0.996 | 250, 0.990 |
| H0351.1015 | 8, 0.981 | 8, 0.987 | --- | 8, 0.992 | 8, 0.967 | 219, 0.987 |
| H0351.1016 | 62, 1.000 | 20, 0.996 | 19, 1.000 | --- | 17, 0.986 | 99, 0.981 |
| H0351.2001 | 73, 1.000 | 18, 0.998 | 69, 0.991 | 6, 1.000 | --- | 374, 0.994 |
| H0351.2002 | 152, 0.989 | 99, 0.977 | 155, 0.998 | 179, 0.996 | 6, 0.975 | --- |

a: the row represent the corresponding test dataset, the column represent the corresponding training dataset. For example, ‘32, 0.994’ means that using the dataset of H0351.1009 as the training dataset and the dataset of H0351.1012 as the test dataset, the feature set containing the first 32 features in the mRMR feature list of dataset of H0351.1009 can yield the maximum total prediction accuracy 0.994 on the test dataset.
