# Supplementary material for: Analysis of Gene Expression Profiles in the Human Brain Stem, Cerebellum and Cerebral Cortex
Source: PLoS One. 2016 Jul 19;11(7):e0159395. doi: 10.1371/journal.pone.0159395 (PMC4951119; doi:10.1371/journal.pone.0159395)
Supplement: S4 Table — (DOCX) [file pone.0159395.s010.docx]

**S4 Table.** The inflection point for each IFS curve and its corresponding total prediction accuracy ^a^

| **Code of people** | H0351.1009 | H0351.1012 | H0351.1015 | H0351.1016 | H0351.2001 | H0351.2002 |
| --- | --- | --- | --- | --- | --- | --- |
| H0351.1009 | --- | 5, 0.979 | 5, 0.953 | 5, 0.978 | 5, 0.973 | 5, 0.951 |
| H0351.1012 | 17, 1.000 | --- | 10, 0.987 | 7, 0.994 | 7, 0.985 | 7, 0.980 |
| H0351.1015 | 8, 0.981 | 8, 0.987 | --- | 5, 0.986 | 9, 0.967 | 5, 0.929 |
| H0351.1016 | 5, 0.997 | 10, 0.992 | 7, 0.985 | --- | 9, 0.983 | 9, 0.948 |
| H0351.2001 | 6, 0.981 | 6, 0.996 | 6, 0.985 | 6, 1.000 | --- | 6, 0.983 |
| H0351.2002 | 5, 0.978 | 5, 0.974 | 13, 0.983 | 9, 0.986 | 6, 0.975 | --- |

a: the row represent the corresponding test dataset, the column represent the corresponding training dataset.
